# Stereochemical significance of O to N atom interchanges within cationic helicenes: experimental and computational evidence of near racemization to remarkable enantiospecificity†
†Electronic supplementary information (ESI) available. CCDC 1908256 and 1908257. For ESI and crystallographic data in CIF or other electronic format see DOI: 10.1039/c9sc02127b


**DOI:** 10.1039/c9sc02127b

**Published:** 2019-06-18

**Authors:** Geraldine M. Labrador, Céline Besnard, Thomas Bürgi, Amalia I. Poblador-Bahamonde, Johann Bosson, Jérôme Lacour

**Affiliations:** a Department of Organic Chemistry , University of Geneva , Switzerland . Email: Amalia.PobladorBahamonde@unige.ch ; Email: johann.bosson@unige.ch ; Email: jerome.lacour@unige.ch; b Laboratory of Crystallography , University of Geneva , Switzerland; c Department of Physical Chemistry , University of Geneva , Switzerland

## Abstract

Oxygen atoms of cationic dioxa and azaoxa [6]helicenes can be exchanged by amino groups to form azaoxa and diaza [6]helicenes respectively. The mild reaction conditions developed herein allow the construction of libraries of derivatives with sensitive and/or functionalized side chains. Using enantioenriched dioxa or azaoxa helicene precursors, these exchanges lead to either near racemization (es 3%) or to a remarkable enantiospecificity (es up to 97%). This unusual behavior is fully characterized *via* experimental and computational mechanistic evidence. Based on these investigations, the enantiospecificity of the first transformation can be improved to 57–61%.

## Abstract 2

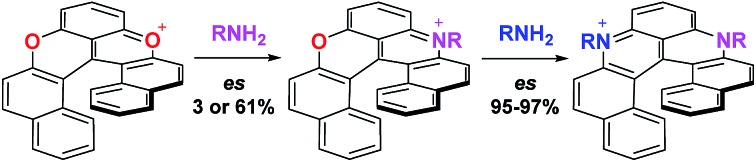
Oxygen atoms of cationic dioxa and azaoxa [6]helicenes can be exchanged by amino groups and these reactions lead to either near racemization (es 3%) or to a remarkable enantiospecificity (es up to 97%). Based on detailed investigations, the chirality transfer of the first transformation can be improved to 61%.

## Introduction

**Fig. 1:**
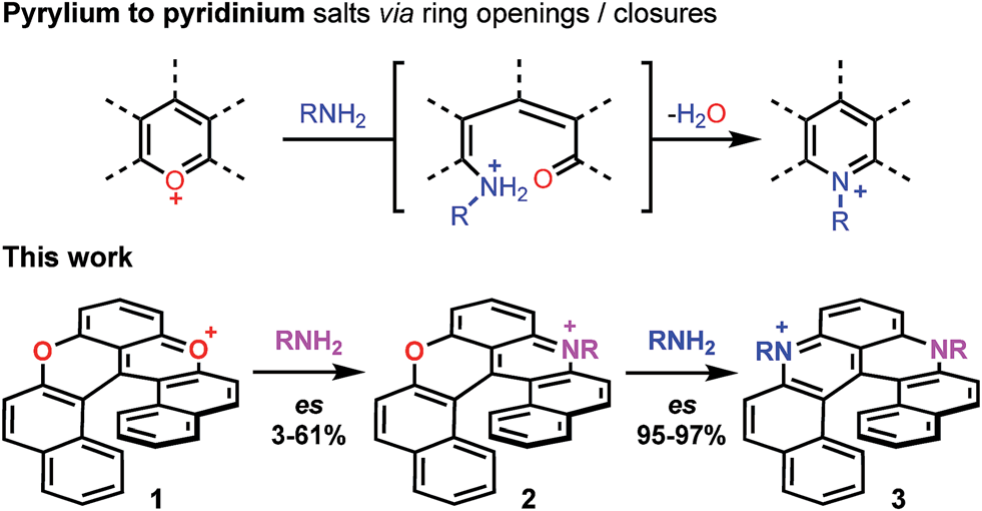
Top: conversion of pyrylium to pyridinium ions *via* sequences of ring openings and ring closures. Bottom: enantiospecific transformations of cationic dioxa and mono oxa [6]helicenes to the corresponding aza derivatives.

Pioneered by Baeyer and Piccard more than a century ago,^1^–^3^ the formation of pyridinium ions by treatment of pyrylium salts with primary amines is recognized as a method of choice for the construction of cationic aza aromatics (Fig. 1, top).^4^–^6^ The three step reaction mechanism, *i.e.*, (i) the nucleophilic attack of the amine, (ii) the O-ring opening yielding an intermediate ketone and (iii) the N-ring closure on the carbonyl,^7^–^10^ classes this reaction as an ANRORC process.^11^ The driving force of the transformation is the better stabilization of the positive charge by the more electron-donating amino group. When the pyrylium unit is embedded in a polyaromatic scaffold, the reaction suffers from a breaking of the overall aromaticity upon O-ring opening. The transformation of xanthylium salts to acridinium ions is thus more challenging but it remains possible as exemplified by the groups of Laursen, Müllen or recently Nicewicz, usually in moderate yields.^12^–^16^ The amenability of this transformation to non-planar chiral polyaromatic systems, and helicenes in particular, was questionable. In fact, with strained substrates like helicenes, the occurrence of more stable (sterically less-demanding) ring-opened biaryl intermediates might disfavor the final ring closure. Conversely, extension of this strategy to cationic oxa helicenes would allow the rapid construction of libraries of aza helicenes,^17^–^19^ compounds that are attractive in regard of their applications in chemistry and at the interface of biology and physics.^20^–^25^ This methodology would also be an interesting addition to the field of late-stage functionalization that remains challenging in polyaromatics.^26^–^33^ Finally, enantiospecific substitutions of O by N atoms within the helicene outer rim would be a novelty in the helicene field. Herein the pyrylium to pyridinium route is extended to cationic [6]helicenes,^34^,^35^ and the direct transformation of dioxa **1** into azaoxa **2** and ultimately into diaza **3** is demonstrated (Fig. 1, bottom). The reaction conditions are sufficiently mild to introduce tailored and/or chemically sensitive functional groups (24 examples). From **2** to **3**, the reaction is enantiospecific (es up to 97%, retention of configuration), which is in stark contrast to the conversion of **1** into **2** for which an almost racemization occurs (es 3%). In-depth experimental and computational investigations of the reaction profiles reveal, in this latter case, the occurrence of propeller intermediates that can racemize through a series of two ring flip processes.^36^ After adaptation of the experimental conditions to limit such deleterious pathways, reaction **1** to **2** then occurs with an effective transfer of the stereochemical information (es 57–61%).

## Results and discussion

In the initial report,^34^ hetero helicenes **1**, **2** and **3** were prepared using a common acyclic triarylcarbenium precursor and then three different ring closing routes, each at very high temperatures (>170 °C, Schemes S1 and S2†). The synthesis of dioxa **1** was particularly efficient as it was obtained in 95% yield in 5 min in molted Pyr·HCl. For the construction of aza helicenes **2** and **3**, it was only possible to introduce amines with linear alkyl chains in low to moderate yields; amines substituted with (sensitive) functional groups were incompatible. In this context, the possibility to use dioxa **1** as a unique precursor to prepare derivatives **2** and **3** in one step *via* a “pyrylium to pyridinium” strategy was deemed not only interesting but also advantageous considering the ready availability of **1** in large scale and high yield by the previous method. This was put to the test. Experimentally the addition of an excess of *n*-propyl amine (25 equiv.) to a solution of dioxa **1** in NMP resulted in an immediate color change from red to blue. Upon heating at 70 °C for 7 h, the formation of diaza **3a** was evidenced (ESI-MS monitoring, 13% isolated yield). After this result, reaction conditions were optimized (Tables S1–S3†). A reduction of the amount of amine (3 equiv.) and the use of benzoic acid as additive (1.5 equiv.) afforded the preferential formation of azaoxa **2a** in 3 h at 60 °C in 38% yield.^37^ Upon increasing the reagents' stoichiometry (25 equiv. of amine and 12.5 equiv. of benzoic acid) along with a prolonged reaction time (7 h) at 70 °C, dioxa **1** was converted into diaza **3a** in 47% yield. The same reaction conditions also afforded diaza **3a** from azaoxa **2a** in 36% yield (Fig. 1, R = *n*-propyl).

**Fig. 2:**
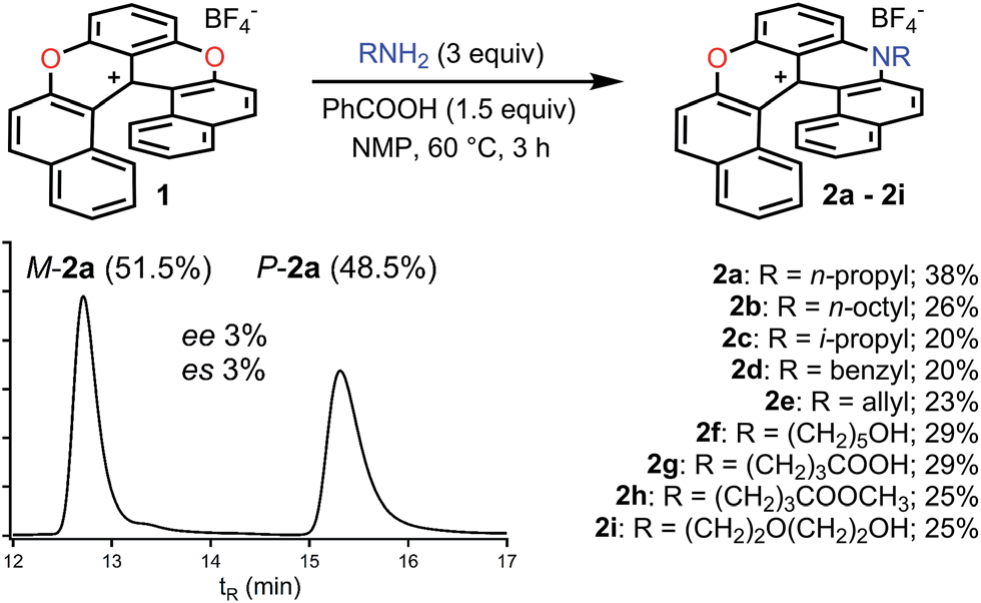
Transformation of dioxa **1** into azaoxa **2a** to **2i**. Inset: CSP HPLC trace of **2a** (enantiospecificity es 3% starting from (–)-*M*-**1**, ee 98%).

With the mild conditions developed above, the scope of the reaction was extended. The preparation of azaoxa derivatives **2b** to **2i** was investigated (Fig. 2). A focus was given to the introduction of residues which were not tolerated before. For instance, *n*-octyl **2b** was obtained in 26% yield. More sterically demanding i-propyl amine yielded **2c** (20% yield). Redox sensitive benzyl and allyl amines were readily introduced (compounds **2d** and **2e**, 20% and 23% respectively).^15^,^38^,^39^ Alcohol, carboxylic acid and ester functional groups were also compatible with the reaction conditions (compounds **2f–2h**, 29%, 29% and 25% respectively). Finally, PEG functionalized azaoxa helicene **2i** was obtained in 25% yield.

**Fig. 3:**
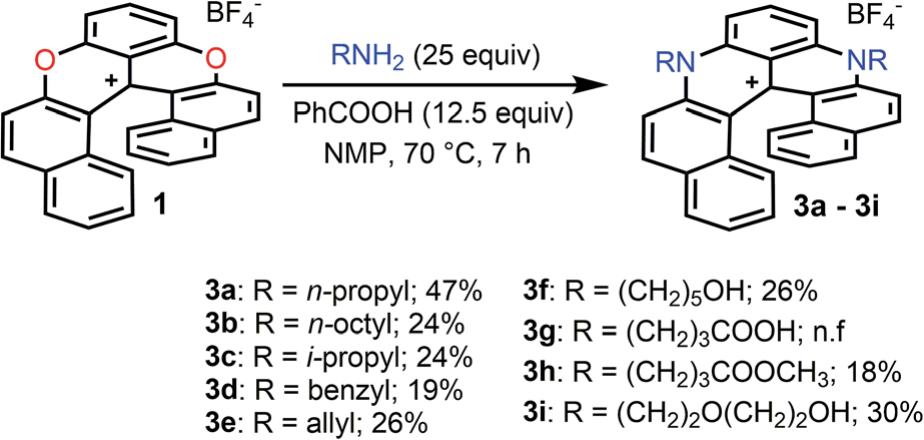
Transformation of dioxa **1** into diaza **3a** to **3i**. n.f.: not formed.

Next, the preparation of symmetrical diaza [6]helicenes **3b** to **3i** using dioxa **1** as substrate was studied (Fig. 3). Introduction of linear alkyl or alcohol chains proceeded unsurprisingly as **3b** or **3f** were isolated in moderate yields (24 and 26%, respectively). As for compound **2c**, it was possible to introduce an isopropyl residue (**3c**, 24% yield). Interestingly, diaza [6]helicenes **3d** and **3e** with dibenzyl and diallyl side chains were also accessible (19% and 26%). While, to our surprise, a simple carboxylic acid moiety could not be added (**3g**), the methyl ester analogue was integrated to the helical scaffold (**3h**, 18%). Bis PEGylated helicene **3i** was also isolated in 30% yield.

**Fig. 4:**
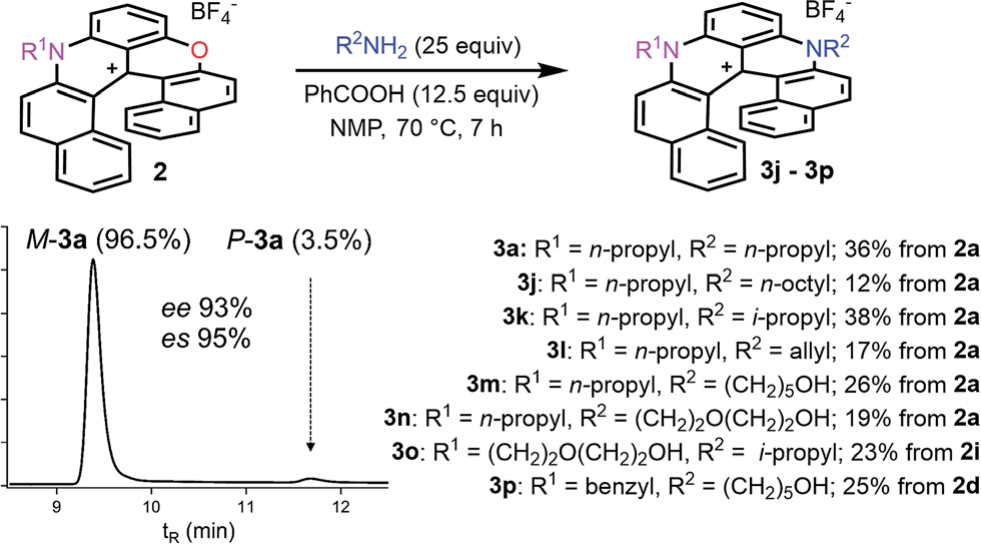
Transformation of azaoxa **2** into diaza **3**. Inset: CSP HPLC trace of **3a** (es 95% starting from (–)-*M*-**2a**, ee 98%).

Finally, compounds **2** were also transformed into diaza **3** giving access to unsymmetrically substituted analogues **3j** to **3p** (Fig. 4). For instance, *n*-propyl azaoxa helicene **2a** was easily converted into diaza helicenes **3j** (12%), **3k** (38%) and **3l** (17%) by reactions with aliphatic *n*-octyl, sterically hindered i-propyl and allyl amines respectively. Hydroxyl functionalized diaza helicenes **3m** and **3n** were also readily prepared from **2a** (26% and 19% yield respectively). PEG functionalized azaoxa **2i** was converted into diaza helicene **3o** bearing an isopropyl group (23%) while substrate **2d** led to the formation of diaza **3p** bearing both sensitive benzyl and hydroxypentyl moieties (25%).

Importantly, reactions **1** → **2a**, **1** → **3a** and **2a** → **3a** were then performed with enantioenriched substrates to determine, as mentioned earlier, if the substitution(s) of the O by N atoms could occur enantiospecifically. This was in fact debatable as the S_N_(ANRORC) mechanism offers *a priori* many possibilities for a loss of enantiomeric purity after ring opening steps in particular. Practically, *rac*-**1**, *rac*-**2a** and *rac*-**3a** were resolved by chiral stationary phase (CSP) HPLC into the corresponding *M* and *P* enantiomers (ee 97–99%). Compounds **1**, **2a** and **3a** are configurationally stable at elevated temperatures, up to 110, 150 and >180 °C specifically.^34^,^40^ The absolute configuration of the enantiomers of **1** and **2a** was unknown prior to this study. It was established by vibrational circular dichroism (VCD) thanks to the conformational rigidity of the compounds that helped the determination (see ESI, Fig. S18–S23†). Interestingly, in this series which also includes **3a**,^40^ the dextro and levorotatory enantiomers are of *P* and *M* configurations irrespectively of their dioxa, azaoxa or diaza nature.

**Fig. 5:**
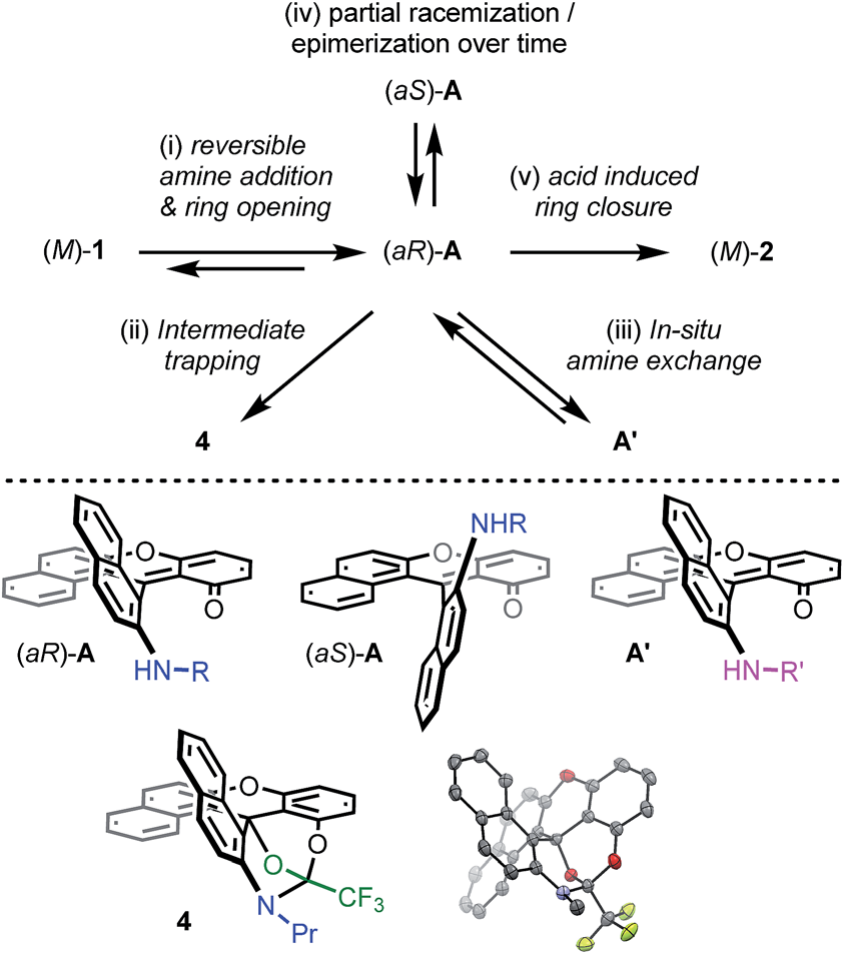
Top: summary of mechanistic investigations. (i) RNH_2_ (3 equiv.), 20 °C, CH_3_CN; then work-up with water to establish the reversibility. (ii) F_3_CCOCl (12 equiv.), CH_3_CN, 20 °C, 16 h, **4** (57%). (iii) Evaporation then R′NH_2_ (3 equiv.), 20 °C, CH_3_CN. (iv) Addition of (*S*)-methyl-*tert*-butylamine and ^1^H NMR monitoring (see Fig. 6). (v) Evaporation then benzoic acid (1.5 equiv.), NMP, 60 °C, 3 h. Bottom: chemical structures of intermediates (*aR*)-**A**, (*aS*)-**A** and **A′**. Drawing and ORTEP view of the crystal structure of **4**: hydrogen atoms are omitted and the propyl chain is truncated for clarity reasons.

Then, attempts to prepare **2a** starting from enantiopure (+) and (–)-**1** led unfortunately to the formation of (+)-*P*- and (–)-*M*-**2a** in only marginal enantiomeric excesses (ee 3%). Similarly, **3a** was obtained from enantiopure (+)- and (–)-**1** in almost racemic form (4% and 0% ee for the (+)- and (–)-enantiomers respectively). In striking contrast, conversion of azaoxa (+)-*P*- and (–)-*M*-**2a** (ee 99 and 98%) provided diaza (+)-*P*- and (–)-*M*-**3a** in 96 and 93% ee, indicating this time a very high level of enantiospecificity for the O to N exchange (es 97–95%). The three transformations detailed above, and **2a** → **3a** in particular, occur hence with retention of configuration.^41^ With these results in hand, experimental and theoretical investigations were performed to determine the mechanism. The aim was to understand the reactivity pathways including their stereochemistry, measure the importance of additives like benzoic acid or offer prospects on how to limit the racemization. Various intermediates, products of trapping experiments and reaction processes were identified. They are summarized in Fig. 5 and specific details are given the following paragraphs.

## Mechanistic evidence

First, dioxa helicene **1** was treated at ambient temperature with an excess of ^*n*^PrNH_2_ which led to one intermediate, named **A**, almost instantly and quantitatively. In the absence of benzoic acid and in the given reaction time (3 h), the reaction did not proceed further. To our surprise, any attempt to isolate this intermediate after aqueous workup led to the complete recovery of starting dioxa **1**. The reaction **1** ⇆ **A** is thus fully reversible in the presence of water.^42^ Detailed *in situ* NMR spectroscopy and MS analyses revealed that **A** possesses a biaryl structure with the N atom connected to the naphthyl ring as a result of a regioselective O-ring opening (see ESI, Fig. S1†). The structure of **A** was indirectly confirmed by the isolation and X-ray structural analysis of *leuco* compound **4** (see ESI and CCDC ; 1908256 †); this derivative results from the addition of an excess of trifluoroacetyl chloride to **A** (Fig. 5). Also, if a different amine is added to **A**, then full substitution of the first amine by the second is evidenced in ^1^H NMR spectroscopy. This exchange forming intermediate **A′** shows (again) the reversibility of amine additions as observed experimentally by the replacement of ^i^Pr by ^*n*^Pr residues (**A** ⇆ **A′**, Fig. S2 and S3†).

**Fig. 6:**
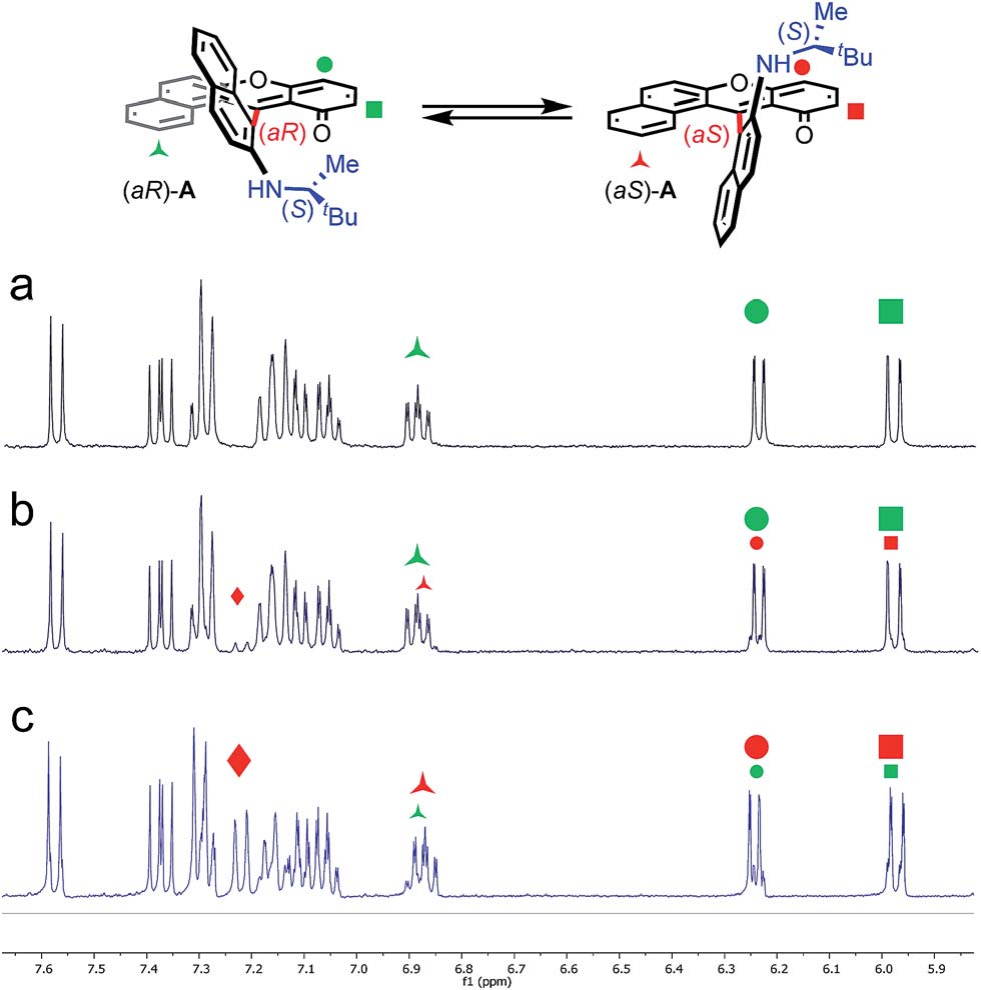
Diastereoisomers of **A** prepared with (*S*)-methyl-*tert*-butylamine. ^1^H NMR spectroscopy performed at 400 MHz in CD_3_CN. Spectrum (a): (–)-*M*-**1** with excess (*S*)-methyl-*tert*-butylamine after 5 min at 20 °C, d.r. > 49 : 1. Spectrum (b): (–)-*M*-**1** with excess (*S*)-methyl-*tert*-butylamine after 3 h at 60 °C, d.r. 88 : 12. Spectrum (c): (+)-*P*-**1** with excess (*S*)-methyl-*tert*-butylamine after 3 h at 60 °C, d.r. 21 : 79. 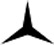 = H_2_, 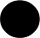 = H_7_, 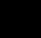 = H_9_, 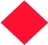 not attributed.

Of particular interest for the stereochemistry of the process, (*S*)-methyl-*tert*-butylamine was added to enantiopure (–)-*M*-dioxa [6]helicene **1**. Upon addition, a single set of NMR signals was observed for intermediate **A** (Fig. 6, spectrum (a), see also Fig. S4 in the ESI†). For instance, signals of protons H_7_ and H_9_ appeared as simple doublets at 6.23 and 5.98 ppm respectively. However, after 3 h at 60 °C (Fig. 6, spectrum (b)), all the mentioned doublets, and some other signals, were split in two. The crude, which was composed initially of a single species (spectrum (a)) has now become a mixture of two stereoisomers in a 88 : 12 ratio.^43^ Importantly, spectrum (b) is similar and opposite to spectrum (c) that is obtained upon the reaction of (*S*)-methyl-*tert*-butylamine with (+)-*P*-**1**. In this latter case, the presence of two diastereoisomers also occurs after some time at 60 °C. The diastereomeric ratio of 88 : 12 (spectrum (b)) is now inverted to a 21 : 79 value (spectrum (c), see also Fig. S5 in the ESI†). Clearly, in the span of the reaction, an epimerization/racemization process occurs at the stage of intermediate **A** but only to a minimal extent; some further steps must happen to account for the almost complete racemization observed at the end of the process. In Fig. 6 are represented the geometry of the stereoisomers of **A** obtained upon addition of the chiral amine onto (–)-*M*-**1**; the configuration of the epimers was determined in the course of the study (*vide infra*).

Finally, it was established that **A** is a productive intermediate as, in the presence of benzoic acid (1.5 equiv.) at 60 °C (3 h), **A** transforms itself into the corresponding helicene **2a**. The presence of the carboxylic acid is essential for the way forward and its role in the promotion of the aza ring closure will be clearly demonstrated by the theoretical analysis (*vide infra*). Conversely the use of sodium benzoate alone provided **2a** in only 4% yield (see Table S3†). Other acids like ^*n*^PrNH_3_·Cl (3 equiv.) did not induce the formation of the azaoxa [6]helicene.

**Fig. 7:**
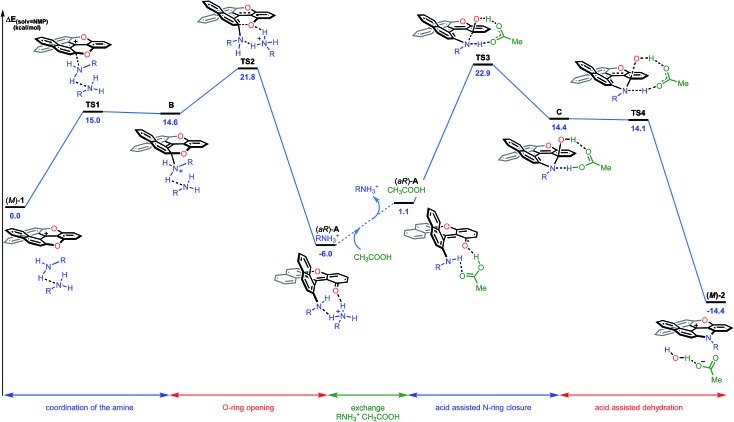
Reaction energy profile of the transformation of (–)-(*M*)-dioxa **1** into (–)- (*M*)-azaoxa **2** (R = methyl). The reported PCM energies (solvent = NMP) are given in kcal mol^–1^.

In conclusion for this section, experimental evidence was found to shed some light on the mechanism of the transformation of **1** to **2a**, and on the existence of intermediates of type **A** or **A′**. Structural and energetical aspects of the process were however hard to apprehend. Care was thus taken to perform an extensive computational survey of the reaction pathway which is depicted in Fig. 7 starting from (*M*)-**1** (energy profile).^44^

## Theoretical study

Computationally, the experimental excess of amine (3 equiv.) was modelled using a bis-amine cluster. The nucleophilic attack, which occurs exclusively on the naphthyl subunits, can happen on the *Re* and *Si* faces of **1**, and both approaches were simulated. The reaction that occurs on the *Re* face of (*M*)-dioxa **1** proceeds through a late transition state **TS1** (Δ*E*^‡^ = +15.0 kcal mol^–1^) which models the dearomatization of the naphthyl moiety and leads to the unstable ammonium adduct **B** (Δ*E* = +14.6 kcal mol^–1^). The following carbon oxygen bond cleavage occurs *via* a 6-membered transition state **TS2** at 21.8 kcal mol^–1^ and it generates stabilized intermediate (*aR*)-**A** that possesses an *ortho* quino methide character (Δ*E* = –6.0 kcal mol^–1^).^45^ Importantly, a strong association with the bis-amine cluster occurs and the positive charge is now localized on the H-bonded ammonium adduct. The attack on the *Si* face follows a similar behavior although in this case, intermediate **A** is highly distorted and clearly unfavorable energetically (Δ*E*(**A**_*Re*_) = –6.0 kcal mol^–1^*vs.* Δ*E*(**A**_*Si*_) = +5.7 kcal mol^–1^, Fig. S24†). These observations are in adequacy with the experimental results and the existence of a highly stereospecific formation of **A**, at the beginning of the reaction in particular. It is rooted in the facial *Re* selectivity of the O-ring opening of dioxa (*M*)-**1** and, hence, in the stereocontroled formation of biaryl (*aR*)-**A**.

For the subsequent conversion of intermediate **A** to azaoxa **2**, an explicit carboxylic acid molecule is required computationally to reproduce well the experimental observations. Calculations show that the replacement of the ammonium ion by the carboxylic acid destabilizes the supramolecular adduct, from –6.0 to 1.1 kcal mol^–1^. Once the acid coordinated, the desired N-ring closure happens *via***TS3** (Δ*E*^‡^ = +22.9 kcal mol^–1^) and forms **C**. During the C–N bond formation, the carboxylic acid acts as a proton shuttle helping the approach of the nucleophile by promoting simultaneously the deprotonation of the amino group and the protonation of the oxygen atom. Then, dehydration of **C** occurs in a barrier-less process and provides azaoxa (*M*)-**2** that is more stable than the starting dioxa (*M*)-**1** by 14.4 kcal mol^–1^.^46^ Overall, the transformation from the ammonium adduct (*aR*)-**A·^+^**H_3_NR to **C** constitutes the rate determining step (Δ*E*^‡^ = +28.9 kcal mol^–1^). An analogous computational study was performed starting from (*P*)-**1** and leading to the formation of (*P*)-**2**; throughout the study, an opposite *aS* configuration for intermediate **A** was evidenced (see ESI, Fig. S25†).

**Fig. 8:**
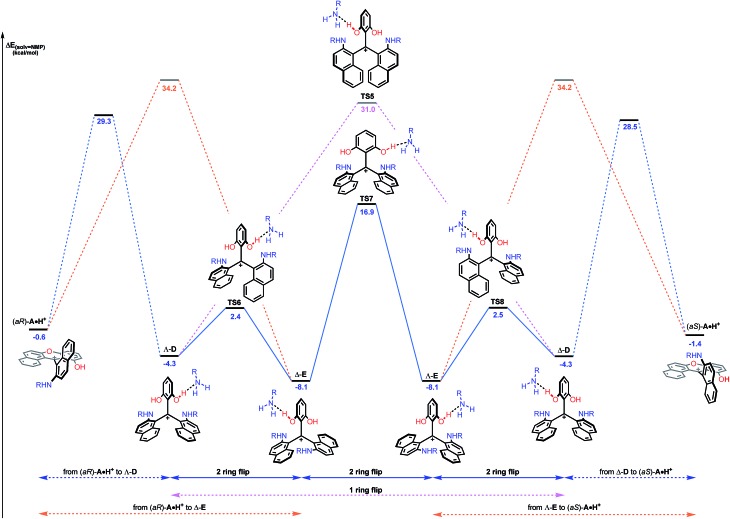
Reaction energy profile of the enantiomerization from (*aR*)-**A·H^+^** to (*aS*)-**A·H^+^**. The ring opening of (*aR*)-**A·H^+^** from the *Re* face leading to Λ-**D** (blue dashed lines) and from the *Si* face leading to Δ-**E** (orange dashed lines) are detailed in the ESI.† Direct isomerization of Λ-**D** into Δ-**D** (pink dashed lines) is clearly not favored. The reported PCM energies (solvent = NMP, R = methyl) are given in kcal mol^–1^.

**Fig. 9:**
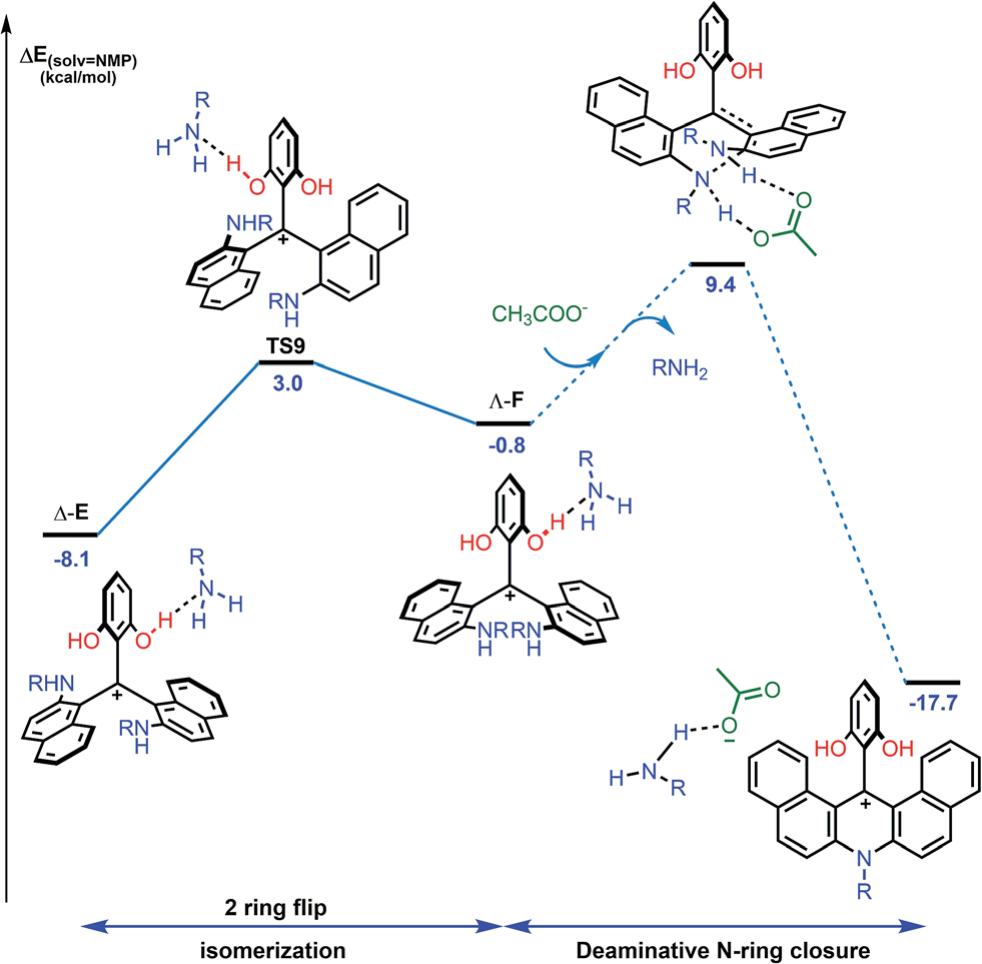
Reaction energy profile for the isomerization from Δ-**E** to Λ-**F** and subsequent formation of dibenzoacridinium **5***via* an acid-promoted cyclization/deamination sequence. The details of the pathway from Λ-**F** to **5** (dashed lines) are depicted in the ESI, Fig. S33.† The reported PCM energies (solvent = NMP, R = methyl) are given in kcal mol^–1^.

While this theoretical approach (Fig. 7) accounted well for the reactivity profile of the reaction, the above calculations did not rationalize the isolation of **2** in almost racemic form starting from either (*M*)-**1** or (*P*)-**1**. In fact, as detailed above, the O-ring opening of dioxa (*M*) or (*P*)-**1** is enantiospecific and leads to intermediates (*aR*) and (*aS*)-**A** which in turn are converted to azaoxa (*M*) and (*P*)-**2** respectively. Also, as shown in the experiments with (*S*)-methyl-*tert*-butylamine (Fig. 6), intermediates of type **A** are quite configurationally stable in basic medium at 60 °C. The origin of the racemization was thus debatable and the possibility of an equilibrium between intermediates (*aR*)-**A** and (*aS*)-**A** induced by an acidification was considered (Fig. 8). As a matter of fact, upon protonation, (*aR*)-**A** is transformed into (*aR*)-**A·H^+^** in which the positive charge is localized on the xanthenium core. This species is highly electrophilic and, as a consequence, it reacts further with the excess of amine cleaving the second ether linkage. Again, this attack can formally occur from either *Re* and *Si* faces of (*aR*)-**A·H^+^** leading to the formation of two three-bladed propellers, named Λ-**D** and Δ-**E**; the Λ and Δ prefixes corresponding to the left and right handed propeller configurations respectively as the additions behave stereoselectively. In **D**, the two amino groups point inward, *i.e.* toward the central phenyl ring while in **E**, one amino group is directed toward the center and the other outward. The global energy profiles, depictured in Fig. 8, clearly indicate the preferential kinetic formation of Λ-**D** over Δ-**E** (see the ESI for more details, Fig. S26 and S27†).^47^ However, the enantiomerization of Λ-**D** into Δ-**D**, through **TS5**, is highly disfavored (Δ*E* = +35.3 kcal mol^–1^) and follows an unusual one ring flip route in which the transition state deviates from ideality; the distortion is brought by the steric hindrance of the naphthyl units.^36^,^48^ Alternatively, stepwise isomerization of Λ-**D** into Δ-**D** was computed through a first facile isomerization of Λ-**D** into Δ-**E** that follows a two ring flip mechanism, *via***TS6** (Δ*E*^‡^ = +6.7 kcal mol^–1^), in which one of the larger naphthyl group remains in the main plane. Subsequent enantiomerization from Δ-**E** to Λ-**E**, also modeled through a two ring flip mechanism with **TS7** located at +16.9 kcal mol^–1^, then accounts for the racemization observed under acid conditions. In fact, (*aS*)-**A·H^+^** is readily obtained *via* the antipodal route from Λ-**E** and Δ-**D** leading to a globally facile (*aR*)-**A** ⇆ (*aS*)-**A** equilibrium under acidic conditions.^49^ Noteworthy, the isomerizations of Λ-**D** into Δ-**D** and of Δ-**E** into Λ-**E** were also computed in the absence of the extra H-bonded amine; quite higher energies for the associated TS were obtained in both cases (see ESI, Fig. S28–S30†).^50^ It is also worth to mention that propeller intermediates **D** and **E** were detected by *in situ* mass spectrometry and their presence explain as well the formation of dibenzoacridinium **5** which can be isolated as a side product (10–30% yield, Fig. 9).^51^ In fact, a relatively simple pathway was computed for its formation from Δ-**E***via* intermediate **F** and an acid-promoted cyclization/deamination (Fig. 9); the overall kinetic barrier is compatible with the experimental observation of **5** (Δ*E*^‡^ = +17.5 kcal mol^–1^).

## Toward higher level of enantiospecificity

**Fig. 10:**
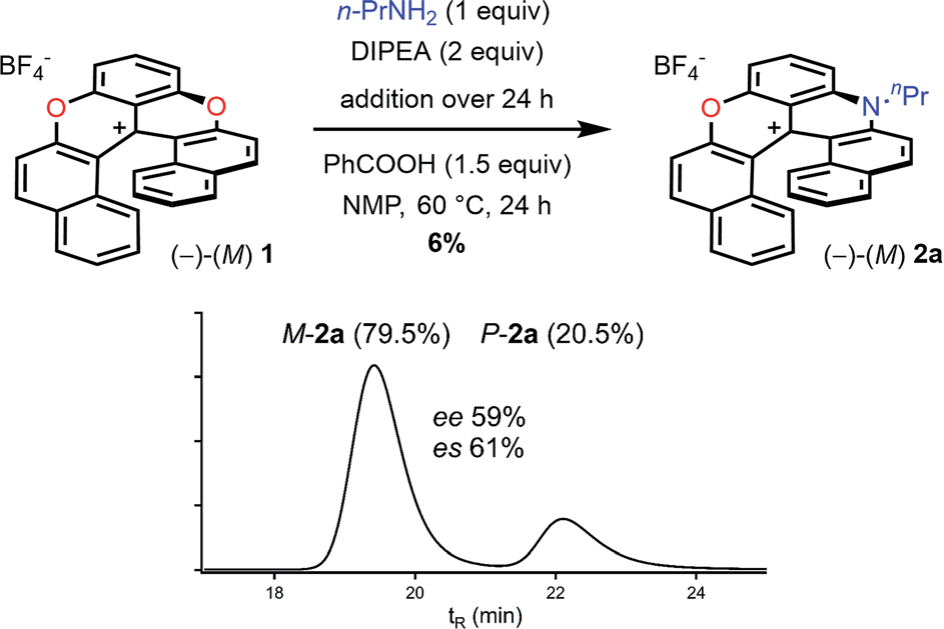
Transformation of dioxa (–)-*M*-**1** into azaoxa (–)-*M*-**2a**. Inset: CSP HPLC trace of **2a** (enantiospecificity es 61% starting from (–)-*M*-**1**, ee 97%).

According to the mechanistic scheme detailed above (Fig. 8), the racemization observed during the formation of azaoxa **2** occurs *via* three-bladed propeller intermediates of type **D** and **E** that necessitates both an acid activation and an excess of primary amine to cleave all ether linkages. To overcome this deleterious effect, it was decided to employ a strict stoichiometric amount of primary amine (for the O to N ring exchange) along with an excess of non-nucleophilic Hunig's base to buffer the medium. Furthermore, to avoid the build-up of species **A** (responsible for the racemization), a slow addition of this combination of nucleophilic and non-nucleophilic amines was considered. With this set of conditions (Fig. 10), the enantiospecificity of the transformation from **1** to **2a** was in part recovered (es 61% and 57% with (*M*)-**1** and (*P*) –**1** respectively, Fig. S12 and S13†). The reactions occurred with retention of the configuration as it could be expected from the computational study. Unfortunately, the yield of (*M*) or (*P*)-**2a** was drastically lowered to 6% only.

**Fig. 11:**
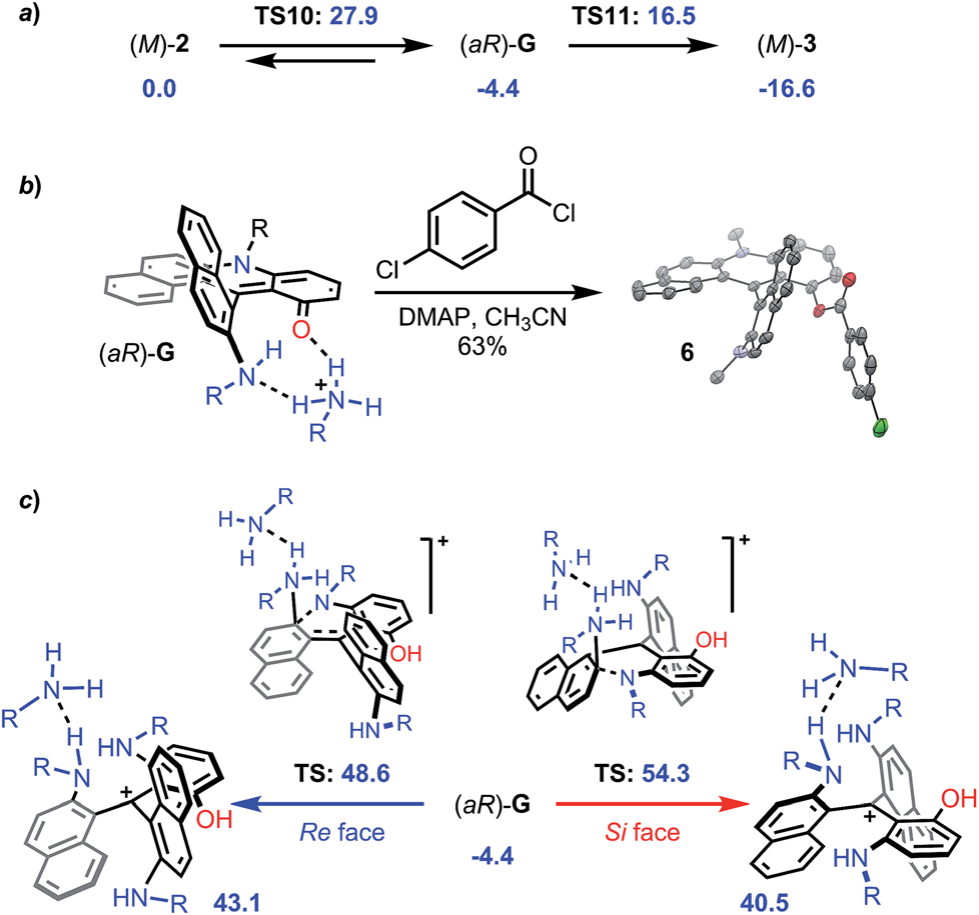
The reported PCM energies (R = methyl, solvent = NMP) are given in kcal mol^–1^. (a) Energies of the general transformation from (*M*)-**2** to (*M*)-**3***via* intermediate (*aR*)-**G**. (b). Trapping of intermediate **G** yielding **6**; ORTEP view of the crystal structure of **6**; hydrogen atoms are omitted and the propyl chains are truncated for clarity reasons. (c) Energies of the N-ring openings of (*aR*)-**G** from the *Re* and the *Si* faces. DMAP = 4-*N*,*N*-dimethylaminopyridine.

In addition, as mentioned earlier, conversion of (+)-*P*- and (–)-*M*-**2a** provides diaza (+)-*P*- and (–)-*M*-**3a** with very high enantiospecificity (es 97–95%) for the O to N exchange. Computationally, the reaction proceeds in a similar fashion to that from **1** to **2**. Ring opening of *M*-**2** with an excess of primary amine leads to the formation of intermediate (*aR*)-**G** located at –4.4 kcal mol^–1^ (Fig. 11a and S34† for details).^52^ This ring opening is regioselective (Fig. S8†) and it was evidenced experimentally by the trapping of **G** with 4-chlorobenzoyl chloride to afford **6** (63%, Fig. 11b and CCDC ; 1908257 †). This step is clearly stereoselective as evidenced by ^1^H-NMR monitoring upon addition of (*S*)-methyl-*tert*-butylamine acting as stereochemical probe. As detailed in Fig. S9,† the NMR spectrum of the addition adduct displays signals for a single stereoisomer which is stable at 70 °C for 7 h. Not surprisingly, the subsequent aza ring closure from (*aR*)-**G** to *M*-**3** (located at –16.6 kcal mol^–1^) is also facilitated by the presence of benzoic acid that promotes the proton transfer and the final dehydration. Yet, the **2** → **3** transformation differs from **1** → **2** in two aspects. First, the O-ring opening is rate determining this time (**TS10** +27.9 kcal mol^–1^). Second, and of utmost importance for the enantiospecificity, protonated intermediate **G·H^+^** does not react with an excess of primary amine (Fig. 11c). Both *Re* and *Si* face approaches and subsequent reactivities would proceed with very high activation energies (+48.6 and +54.3 kcal mol^–1^, respectively) and would be thermodynamically disfavored with corresponding propeller adducts located at +43.1 and +40.5 kcal mol^–1^ (see ESI, Fig. S36 and S37†). This explains the large stereochemical difference between reactions **1** → **2** and **2** → **3** – something that was hard to predict at the onset of the project.

## Conclusions

Herein the transformation of pyrylium rings into pyridinium cycles has been extended to the field of cationic helicenes. The replacement of oxygen atom(s) at the outer rim by stabilizing amino groups proceeds *via* S_N_Ar mechanisms. The reported reaction conditions are sufficiently mild to be beneficial for the introduction of functional groups that were not tolerated previously (24 examples). In-depth mechanistic studies, experimental and theoretical, demonstrate that the process is enantiospecific (es up to 97% for **2** → **3**) and occurs with retention of configuration. Near racemization can however happen *via* three-bladed propeller intermediates, if starting from dioxa **1** in particular. Based on the mechanistic understanding, corrective measures were applied to recover a large amount of the enantiospecificity for the **1** → **2** process (es 57–61%). Care will be taken to utilize this late-stage functionalization strategy for future developments and applications of these original chromophores and fluorophores.^53^–^61^

## Conflicts of interest

There are no conflicts to declare.

## Supplementary Material

Supplementary information

Crystal structure data

## References

[cit1] Baeyer A. (1910). Ber. Dtsch. Chem. Ges..

[cit2] Baeyer A., Piccard J. (1911). Justus Liebigs Ann. Chem..

[cit3] Conlon D. A., in Name Reactions in Heterocyclic Chemistry II, John Wiley & Sons, Inc., 2011, ch. 6.1, pp. 338–346.

[cit4] Aarons R. J., Afarinkia K., Balaban A. T., Balaban T. S., Camp N., Faulkner S., Murphy P. J., Nelson A., Nógrádi M., Rudorf W.-D., Vinader V., Whitehead R. C. and Williams A. C., in Category 2, Hetarenes and Related Ring Systems, ed. E. J. Thomas, Georg Thieme Verlag, Stuttgart, 2003 edn, 2003, vol. 14.1, pp. 11–200.

[cit5] Sowmiah S., Esperanca J. M. S. S., Rebelo L. P. N., Afonso C. A. M. (2018). Org. Chem. Front..

[cit6] For recent applications, see: Wang M. Wang K. Wang C. Huang M. Hao X.-Q. Shen M.-Z. Shi G.-Q. Zhang Z. Song B. Cisneros A. Song M.-P. Xu B. Li X., J. Am. Chem. Soc., 2016, 138 , 9258 –9268 Moser D. Duan Y. Wang F. Ma Y. O'Neill M. J. Cornella J., Angew. Chem., Int. Ed., 2018, 57 , 11035 –11039 Klauck F. J. R. Yoon H. James M. J. Lautens M. Glorius F., ACS Catal., 2019, 9 , 236 –241 . 27379457 10.1021/jacs.6b04959

[cit7] Balaban A. T., Toma C. (1966). Tetrahedron.

[cit8] Katritzky A. R., Manzo R. H. (1981). J. Chem. Soc., Perkin Trans. 2.

[cit9] Katritzky A. R., Lloyd J. M., Patel R. C. (1982). J. Chem. Soc., Perkin Trans. 1.

[cit10] Katritzky A. R., Manzo R. H., Lloyd J. M., Patel R. C. (1980). Angew. Chem., Int. Ed..

[cit11] Van der Plas H. C. (1978). Acc. Chem. Res..

[cit12] Rosenberg M., Rostgaard K. R., Liao Z., Madsen A. Ø., Martinez K. L., Vosch T., Laursen B. W. (2018). Chem. Sci..

[cit13] Bora I., Bogh S. A., Rosenberg M., Santella M., Soerensen T. J., Laursen B. W. (2016). Org. Biomol. Chem..

[cit14] Wu D., Feng X., Takase M., Haberecht M. C., Müllen K. (2008). Tetrahedron.

[cit15] Laursen B. W., Krebs F. C. (2001). Chem.–Eur. J..

[cit16] White A. R., Wang L., Nicewicz D. A. (2019). Synlett.

[cit17] Dumitrascu F., Dumitrascu D. G., Aaron I. (2010). ARKIVOC.

[cit18] Adriaenssens L., Severa L., Šálová T., Císařová I., Pohl R., Šaman D., Rocha S. V., Finney N. S., Pospíšil L., Slavíček P., Teplý F. (2009). Chem.–Eur. J..

[cit19] Arai S., Yafune T., Ōkubo M., Hida M. (1989). Tetrahedron Lett..

[cit20] Stara I. G., Stary I. (2010). Sci. Synth..

[cit21] Shen Y., Chen C.-F. (2011). Chem. Rev..

[cit22] Gingras M. (2013). Chem. Soc. Rev..

[cit23] Bosson J., Gouin J., Lacour J. (2014). Chem. Soc. Rev..

[cit24] Hoffmann N. (2014). J. Photochem. Photobiol., C.

[cit25] Chen C.-F. and Shen Y., Helicene Chemistry: From Synthesis to Applications, Springer, Berlin, Heidelberg, 2017.

[cit26] Schweinfurth D., Mazzolini M., Neshchadin D., Hoyer C., Geier R., Gatterer K., Trapp N., Gescheidt G., Diederich F. (2016). Chem.–Eur. J..

[cit27] Delgado I. H., Pascal S., Wallabregue A., Duwald R., Besnard C., Guenee L., Nancoz C., Vauthey E., Tovar R. C., Lunkley J. L., Muller G., Lacour J. (2016). Chem. Sci..

[cit28] Hasan M., Pandey A. D., Khose V. N., Mirgane N. A., Karnik A. V. (2015). Eur. J. Org. Chem..

[cit29] Bucinskas A., Waghray D., Bagdziunas G., Thomas J., Grazulevicius J. V., Dehaen W. (2015). J. Org. Chem..

[cit30] Weimar M., Correa da Costa R., Lee F.-H., Fuchter M. J. (2013). Org. Lett..

[cit31] Surampudi S. K., Nagarjuna G., Okamoto D., Chaudhuri P. D., Venkataraman D. (2012). J. Org. Chem..

[cit32] Rajca A., Pink M., Xiao S., Miyasaka M., Rajca S., Das K., Plessel K. (2009). J. Org. Chem..

[cit33] Ruch A. A., Handa S., Kong F., Nesterov V. N., Pahls D. R., Cundari T. R., Slaughter L. M. (2016). Org. Biomol. Chem..

[cit34] Torricelli F., Bosson J., Besnard C., Chekini M., Bürgi T., Lacour J. (2013). Angew. Chem., Int. Ed..

[cit35] Bosson J., Labrador G. M., Pascal S., Miannay F.-A., Yushchenko O., Li H., Bouffier L., Sojic N., Tovar R. C., Muller G., Jacquemin D., Laurent A. D., Le Guennic B., Vauthey E., Lacour J. (2016). Chem.–Eur. J..

[cit36] Mislow K. (1976). Acc. Chem. Res..

[cit37] The beneficial role of benzoic acid in S_N_Ar reactions has been already observed by Laursen and co-workers. See for instance ref. 12

[cit38] Nicolas C., Herse C., Lacour J. (2005). Tetrahedron Lett..

[cit39] Laleu B., Herse C., Laursen B. W., Bernardinelli G., Lacour J. (2003). J. Org. Chem..

[cit40] Labrador G. M., Bosson J., Breitbach Z. S., Lim Y., Francotte E. R., Sabia R., Villani C., Armstrong D. W., Lacour J. (2016). Chirality.

[cit41] Later in the manuscript, it will be shown that the transformation of (+)-**1a** → (+)-**2a** and of (–)-**1a** → (–)-**2a** can occur with an improved enantiospecificity of 57–61%; this result establishes firmly the retention of configuration upon heteroatom exchange

[cit42] It will be later shown that the observation of **A** is driven by the formation of an ammonium adduct (Δ*E* = –6.0 kcal mol^-1^). Removal of the ammonium interaction destabilizes the structure by at least +12.6 kcal mol^–1^ and this explains the reversibility upon hydrolysis

[cit43] A related experiment was performed in ECD (CH_3_CN, 2 × 10^–5^ M) upon the addition of *n*-propylamine onto enantiopure (+)-**1**. While an immediate transformation of the ECD spectrum was noticed upon the formation of **A**, the Cotton effects did not evolve over time upon heating at 50 °C for 1 h 30. This indicates that the nature of the amine probably influences the kinetics of epimerization of **A** and that less-sterically demanding amines such PrNH_2_ might favor enantiospecific pathways. See the ESI, Fig. S6 and S7.

[cit44] For examples of DFT calculations on related compounds, see for instance: Lemke S. Ulrich S. Claußen F. Bloedorn A. Jung U. Herges R. Magnussen O. M., Surf. Sci., 2015, 632 , 71 –76 Jacob H. Ulrich S. Jung U. Lemke S. Rusch T. Schütt C. Petersen F. Strunskus T. Magnussen O. Herges R. Tuczek F., Phys. Chem. Chem. Phys., 2014, 16 , 22643 –22650 .

[cit45] Kwan E. E., Zeng Y., Besser H. A., Jacobsen E. N. (2018). Nat. Chem..

[cit46] For examples of chirality transfer from biaryl species to ring closed helicenes, see Murai M. Okada R. Nishiyama A. Takai K., Org. Lett., 2016, 18 , 4380 –4383 Nakano K. Hidehira Y. Takahashi K. Hiyama T. Nozaki K., Angew. Chem., Int. Ed., 2005, 44 , 7136 –7138 .27513028

[cit47] After the coordination of the amine, the O-ring opening requires a high activation energy of +29.3 kcal mol^–1^. In addition, the formation of Δ-**D** is thermodynamically favored starting from (*aR*)-**A·H^+^** (Δ*E* = –4.3 kcal mol^–1^)

[cit48] Traditionally, the enantiomerization of Ar_3_Z propellers containing two identical rings with local *C*_1_-symmetry and one *C*_2_-symmetrical ring occurs preferentially through two ring flip processes. See Gust D. Mislow K., J. Am. Chem. Soc., 1973, 95 , 1535 –1547 .

[cit49] The reaction pathway from (*aS*)-**A·H^+^** to dioxa **1** of (*P*) configuration is detailed in the ESI.

[cit50] Ito H., Abe T., Saigo K. (2011). Angew. Chem., Int. Ed..

[cit51] For the formation of related molecules *via* a pyrylium into pyridinium transformation, see ref. 14

[cit52] For the transformation from **2** to **3**, the energy of **2** is set at 0.0 kcal mol^–1^

[cit53] Isla H., Crassous J. (2016). C. R. Chim..

[cit54] Saleh N., Shen C., Crassous J. (2014). Chem. Sci..

[cit55] Aillard P., Voituriez A., Marinetti A. (2014). Dalton Trans..

[cit56] Jarolímová Z., Bosson J., Labrador G. M., Lacour J., Bakker E. (2018). Electroanalysis.

[cit57] Jarolímová Z., Bosson J., Labrador G. M., Lacour J., Bakker E. (2018). Electroanalysis.

[cit58] Bauer C., Duwald R., Labrador G. M., Pascal S., Moneva Lorente P., Bosson J., Lacour J., Rochaix J.-D. (2018). Org. Biomol. Chem..

[cit59] Li H., Wallabregue A., Adam C., Labrador G. M., Bosson J., Bouffier L., Lacour J., Sojic N. (2017). J. Phys. Chem. C.

[cit60] Li H., Voci S., Wallabregue A., Adam C., Labrador G. M., Duwald R., Hernández Delgado I., Pascal S., Bosson J., Lacour J., Bouffier L., Sojic N. (2017). ChemElectroChem.

[cit61] Duwald R., Pascal S., Bosson J., Grass S., Besnard C., Bürgi T., Lacour J. (2017). Chem.–Eur. J..

