# Supplementary material for: Stereochemical significance of O to N atom interchanges within cationic helicenes: experimental and computational evidence of near racemization to remarkable enantiospecificity
Source: Chem Sci. 2019 Jun 18;10(29):7059–67. doi: 10.1039/c9sc02127b (PMC6676467; doi:10.1039/c9sc02127b)
Supplement: Supplementary file 1 [file SC-010-C9SC02127B-s001.pdf]

Electronic Supplementary Material (ESI) for Chemical Science.
This journal is © The Royal Society of Chemistry 2019

Supporting Information

Stereochemical significance of O to N atom interchanges within cationic helicenes: experimental and computational evidences of near racemization to remarkable enantiospecificity

Geraldine M. Labrador,[a] Celine Besnard,[b] Thomas Bürgi,[c] Amalia I. Poblador-Bahamonde,*[a] Johann Bosson,*[a] and Jérôme Lacour*[a]

The dataset for this article can be found at the following DOI:

10.26037/yareta:ikmm2hq375e75ebqq35mem5dsa

[a] Department of Organic Chemistry, University of Geneva, Switzerland.
[b] Laboratory of Crystallography, University of Geneva, Switzerland.
[c] Department of Physical Chemistry, University of Geneva, Switzerland.
* E-mails: amalia.pobladorbahamonde@unige.ch; johann.bosson@unige.ch; jerome.lacour@unige.ch

Contents

1. General remarks 4
2. Preparation of starting materials 5
3. Optimization of the reaction conditions 6
From dioxa **1** to diaza **3a** 6
From azaoxa **2a** to diaza **3a**. 7
From dioxa **1** to azaoxa **2a**. 8
4. Experimental evidences 10
From dioxa **1** to azaoxa **2** 10
From azaoxa **2** to diaza **3** 20
5. Transformations from 1 to 2 to 3 in enantio-enriched series 25
From dioxa **1** to azaoxa **2a** 25
From dioxa **1** to diaza **3a** 30
From azaoxa **2** to diaza **3** 33
6. Attribution of the absolute configuration of (+)- and (–)-1 and 2a 36
Experiment 36
Calculations 36
7. Discussion about the theoretical rationalization of the mechanism 41
General remarks 41
From dioxa **1** to azaoxa **2** 41
Access to three-bladed propellers **D** and **E** 43
Influence of the extra amine on the relative energies of the isomerizations (Λ)-**D** → (Δ)-**D**, (Δ)-**E** → (Λ)-**E** and (Λ)-**D** → (Δ)-**E** 45
O-ring closure of propeller (Δ)-**D** or (Λ)-**E** leading to cationic (*aS*)-**A·H⁺** 48
Formation of Dibenzoacridinium **5**. 50
From azaoxa **2** to diaza **3** 51
8. Synthesis and characterization of new derivatives 56
Synthesis azaoxa [6]Helicene of type **2** from dioxa [6]Helicene **1** 56
Synthesis diaza [6]Helicene of type **3** from dioxa [6]Helicene **1** 60
Synthesis diaza [6]Helicene of type **3** from azaoxa [6]Helicene of type **2** 63
Synthesis of trapped intermediates 4 and **6** 67
Isolation of side product **5** 68
9. Crystallographic data 70
Compound **4** 70
Compound **6** 72

10. ^{1}H NMR, ^{13}C NMR, ^{19}F NMR and UV/Visible spectra 74
11. Energies and Cartesian coordinates for all the intermediates and transition states 126
From Dioxa (*M*)-**1** to Azaoxa (*M*)-**2** *via* intermediate (*aR*)-**A** 126
From Dioxa (*P*)-**1** to Azaoxa (*P*)-**2** via intermediate (*aS*)-**A**, Attack on the *Si* face. 143
Isomerization from (*aR*)-**A** to (*aS*)-**A** 154
Isomerization without extra amine 185
From Azaoxa (*M*)-**2** to Diaza (*M*)-**3** *via* intermediate (*aR*)-**G**. 195
N-ring opening of cationic (*aR*)-**G·H**$^{+}$ 213
Formation of dibenzoacridinium **5** 223
Miscellaneous structures 236

1. General remarks

Reagents and apparatus:

Acetonitrile, NMP (*N*-methyl-2-pyrrolidinone), DMF (dimethylformamide) CH_2Cl_2, Et_2O, *n*-propylamine was distilled over Zn powder; primary amines were purchased from Aldrich® and used without any further purification.

NMR spectra were recorded on Brucker AMX-500 or ARX-300 at room temperature. **^{1}H NMR**: chemical shifts are given in ppm relative to Me_4Si with solvent resonances used as internal standards (5.32 ppm for CD_2Cl_2). Data were reported as follows: chemical shift (δ) in ppm on the δ scale, multiplicity (s = singulet, brs = broad singulet, d = doublet, t = triplet, dd = doublet of doublet and m = multiplet), coupling constant (Hz) and integration. For **^{13}C NMR**: chemicals shifts were given in ppm relative to Me_4Si with solvent resonances used as internal standards (53.8 ppm for CD_2Cl_2). **IR spectra** were recorded with a Perkin-Elmer 1650. FT-IR spectrometer using a diamond ATR Golden Gate sampling. **R*f*** was measured on TLC Silica gel 60 F254 plates purchased from Merck. **Electrospray** mass spectra were obtained on a Finnigan SSQ 7000 spectrometer by the Department of Mass Spectroscopy of the University of Geneva. **UV/Visible spectra** were obtained using a JASCO-650 spectrometer and were recorded in acetonitrile at a concentration of 2.0 10^{-5} M (unless otherwise stated) in 1 cm quartz cell. λ_{max} are given in nm based on the lowest energy transition and molar absorption coefficient ε ($L.cm^{-1}.mol^{-1}$) in log(ε). **Flash Chromatography** was done with a Combi*Flash*® Rf 200 on SiO_2 4 g cartridge. **HPLC** were performed on Agilent analytical LC 1200 (binary high pressure solvent mixer, automatic sampler, two-column heating-chilling Oven, diode-array detector + ORD detector) or Agilent semi-preparative LC 1100 (quaternary low pressure solvent mixer, automatic sampler, 2-column heating-chilling Oven, diode-array detector, automatic collector); all LC machines are coupled to PC (Analysis program: ChemStation).

2. **Preparation of starting materials**

In the initial report, dioxa **1**, azaoxa **2** and diaza **3** [6]helicenes were prepared from a common precursor.[1] This common precursor is accessible in five synthetic steps on gram scale.

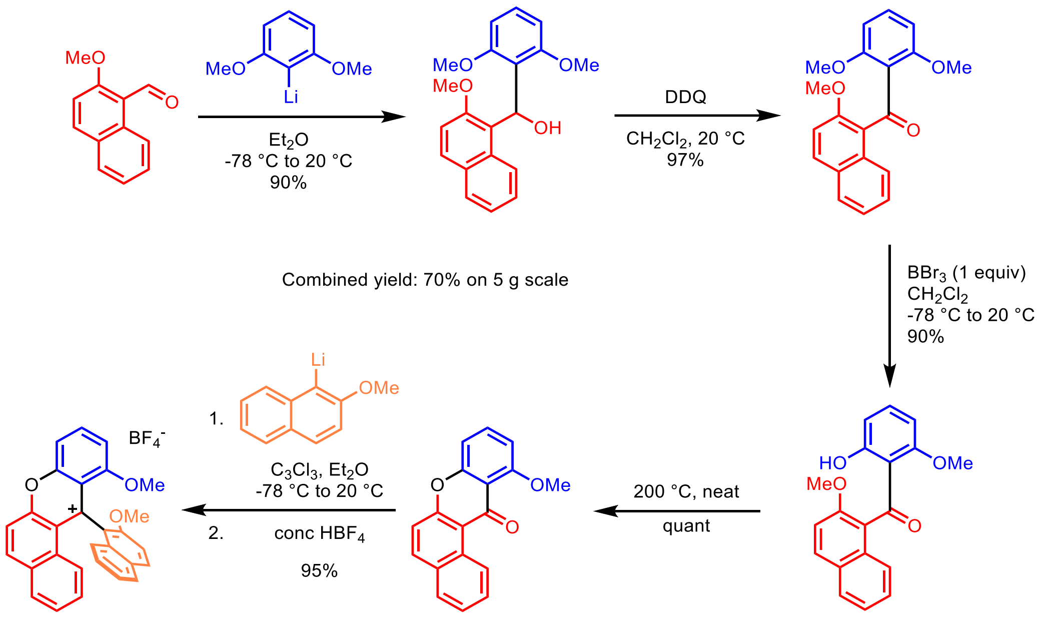

Scheme S1. Five step sequence for the access of a common precursor.

The common precursor is then used for the access to the dioxa **1**, azaoxa **2** and diaza **3** [6]helicenes.

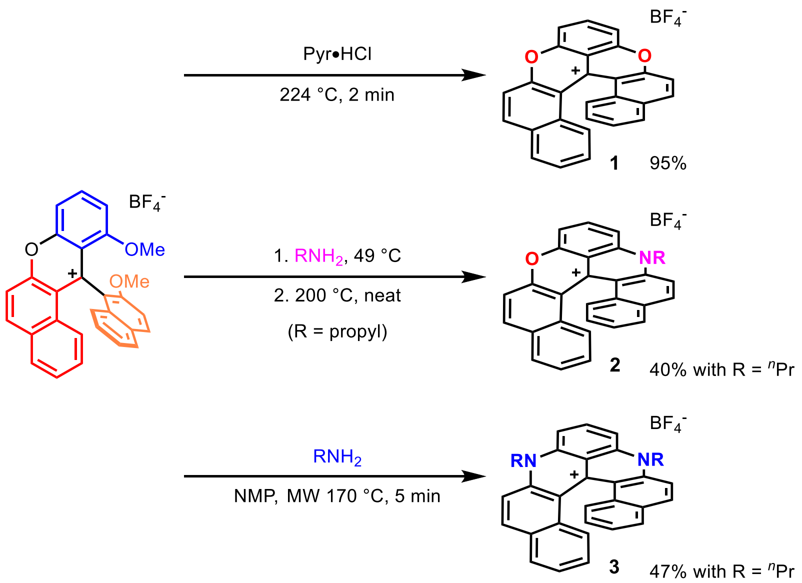

Scheme S2. Access to dioxa **1**, azaoxa **2** and diaza **3** [6]helicenes from a common precursor.

3. **Optimization of the reaction conditions**

The transformations of dioxa **1** to azaoxa **2** and diaza **3** were optimized using *n*-propylamine as reacting partner. The direct formation of diaza **3a** was first studied. With *rac*-dioxa [6]helicene **1** as starting material and 25 equivalents of *n*-propylamine, it was observed the formation of diaza **3a** was possible at 70 °C after 7 h in different solvents (Table S1, entries 1-6). The higher isolated yields were obtained using DMF (26 %) or CH_3CN (22 %). With NMP, THF, DCE and 1-4 dioxane lower isolated yields were obtained (12 - 15 %). However, using NMP, cleaner crude mixtures were evidenced by ^{1}H NMR spectroscopy.

<u>From dioxa **1** to diaza **3a**.</u>

Different combinations of solvents and additives were next screened. First, reactions were performed using different ratios and amounts of benzoic acid and amines in NMP Table S1 (Table S1, entries 7-10). The best and cleaner conditions involve the use of 12.5 equivalents of benzoic acid and 25 equivalents of *n*-propylamine, **3a** being obtained in 47 % yield (Table S1, entry 8). With this ratio (25 equiv of amine and 12.5 equiv of acid) the use of other solvents such as CH_3CN, DMF or THF has a negative impact and lower yields are obtained (Table S1, entries 11, 12, 13). Other carboxylic acids such as trifluoroacetic acid, bromoacetic acid and pivalic acid were also teste with, however, no improvement on the yields (Table S1, entries 14-16).

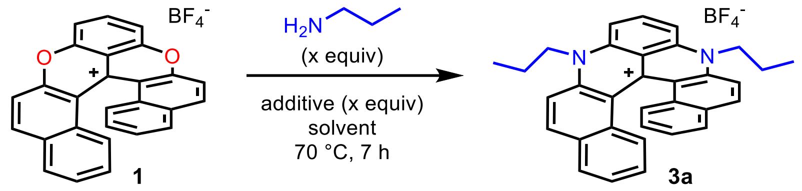

Entry	Solvent	Equiv amine	Additive	Yield[a]
1	NMP	25	-	13 %
2	CH_3CN	25	-	22 %
3	DMF	25	-	26 %
4	THF	25	-	15 %
5	1,4 Dioxane	25	-	14 %
6	DCE	25	-	12 %
7	NMP	25	Benzoic Acid [2.5 equiv]	11 %
**8**	**NMP**	**25**	**Benzoic Acid [12.5 equiv]**	**47 %**

9	NMP	25	Benzoic Acid [25 equiv]	42 %
10	NMP	25	Benzoic Acid [50 equiv]	36 %
11	CH_3CN	25	Benzoic Acid [12.5 equiv]	32 %
12	DMF	25	Benzoic Acid [12.5 equiv]	27 %
13	THF	25	Benzoic Acid [12.5 equiv]	13 %
14	NMP	25	Trifluoracetic Acid [12.5 equiv]	21 %
15	NMP	25	Bromoacetic Acid [12.5 equiv]	24 %
16	NMP	25	Pivalic Acid [12.5 equiv]	15 %

Table S1. Optimization of the transformation of dioxa **1** to diaza **3a**. [a] Average yield of two reproducible reactions.

Representative procedure **A** for the preparation of diaza **3** from dioxa **1**:

To a solution of dioxa [6]helicene **1** (0.1 mmol) in NMP (0.5 mL) were added benzoic acid (12.5 equiv) amine (25 equiv). The mixture was stirred at 70 °C while conversion of starting material was monitored by TLC and MS-ESI. After completion of reaction, the reaction mixture was then cooled to 20 °C. Et_2O (*ca*. 10 mL) was added leading to the precipitation of the crude material. The resulting solid was dissolved in CH_2Cl_2 (*ca.* 5 mL) and washed with aqueous 1 M HBF_4 solution (3 x 10 mL). The organic layer was dried over Na_2SO_4, filtrated and evaporated under reduced pressure. The solid obtained was dissolved in CH_2Cl_2 (*ca.* 1 mL) and precipitated by addition of Et_2O (*ca.* 10 mL). The precipitate was separated from the mother liquor by centrifugation. The product was then purified by flash chromatography (CombiFlash, SiO_2 4 g cartridge, CH_2Cl_2/MeOH, 100:0 to 95:5 over 30 min) yielding the corresponding diaza [6]helicene as a blue powder.

From azaoxa **2a** to diaza **3a**.

The same reaction conditions 25 equivalents of *n*-propylamine and (12.5 equivalents of benzoic acid) revealed to promote efficiently the transformation from azaoxa **2a** to diaza **3a** (Table S2). In this case, slightly better results were obtained using NMP (36 % yield) than CH3CN (32 % yield).

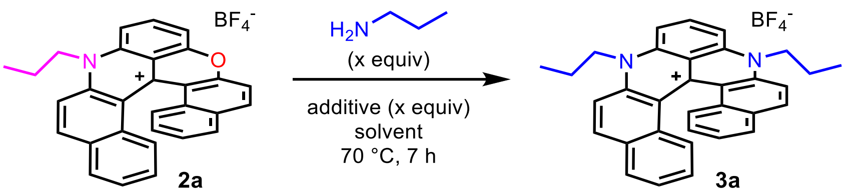

Entry	Solvent	Equiv amine	Additive	Yield[a]
**1**	**NMP**	**25**	**Benzoic Acid [12.5 equiv]**	**36 %**
2	CH_3CN	25	Benzoic Acid [12.5 equiv]	32 %

Table S2. Optimization of the transformation of dioxa **2a** to diaza **3a**. [a] Average yield of two reproducible reactions.

<u>Representative procedure **B** for the preparation of diaza **3** from azaoxa **2**:</u>

To a solution of azaoxa [6]helicene **2** (0.1 mmol) in NMP (0.5 mL)) were added benzoic acid (12.5 equiv) amine (25 equiv). The mixture was stirred at 70 °C while conversion of starting material was monitored by TLC and MS-ESI. After completion of reaction, the reaction mixture was cooled to 20 °C. Et_2O (*ca.* 10 mL) was added leading to the precipitation of the crude material. The resulting solid was dissolved in CH_2Cl_2 (*ca.* 5 mL) and washed with aqueous 1 M HBF_4 solution (3 x 10 mL). The organic layer was dried over Na_2SO_4, filtrated and evaporated under reduced pressure. The solid obtained was dissolved in CH_2Cl_2 (*ca.* 1 mL) and precipitated by addition of Et_2O (*ca.* 10 mL). The precipitate was separated from the mother liquor by centrifugation. The product was then purified by flash chromatography (CombiFlash, SiO_2 4 g cartridge, CH_2Cl_2/MeOH, 100:0 to 95:5 over 30 min) yielding the corresponding diaza [6]helicene **3** as a blue powder.

<u>From dioxa **1** to azaoxa **2a**.</u>

The transformation of dioxa **1** to azaoxa **2a** requires milder reaction conditions (Table S3). Reducing the amounts of amine (3 equiv) and benzoic acid (1.5 equiv) allows the formation of the azaoxa **2a** in only 3 h at 60 °C with 38 % yield in NMP and 30 % in CH_3CN. (Table S3, entries 1-2). No difference was observed using 5 equivalents of amine and 2.5 equivalent of benzoic acid in both solvents (Table S3, entries 3-4). Control experiments with the *n*-propylamine hydrochloride salt or sodium benzoate (Table S3, entries 5-6) clearly highlight the beneficial role of the carboxylic acid.

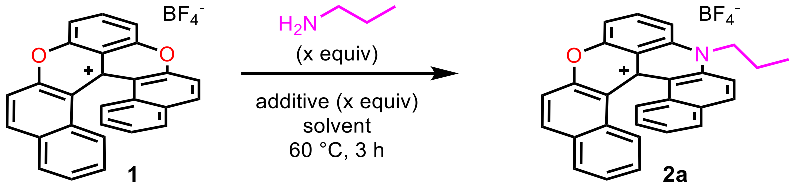

Entry	Solvent	Equiv amine	Additive	Yield [a]
**1**	**NMP**	**3**	**Benzoic Acid [1.5 equiv]**	**38 %**
2	CH_3CN	3	Benzoic Acid [1.5 equiv]	30 %
3	NMP	5	Benzoic Acid [2.5 equiv]	39 %
4	CH_3CN	5	Benzoic Acid [2.5 equiv]	22 %
5[b]	NMP	3	HCl [3 equiv]	13 %
6	NMP	3	Sodium Benzoate [1.5 equiv]	4 %

Table S3. Optimization of the transformation of dioxa **1** to azaoxa **2a**. [a] Average yield of two reproducible reactions. [b] In this case 3 equiv of *n*-propylamine hydrochloride salt were used.

Representative procedure **C** for the preparation of azaoxa **2** from dioxa **1**:

To a solution of dioxa [6]helicene **1** (0.1 mmol) in NMP (0.5 mL) were added benzoic acid (1.5 equiv) amine (3 equiv). The mixture was stirred at 60 °C while conversion of starting material was monitored by TLC and MS-ESI. After completion of reaction, the reaction mixture was cooled to 20 °C. Et_2O (*ca.* 10 mL) was added leading to the precipitation of the crude material. The resulting solid was dissolved in CH_2Cl_2 (*ca.* 5 mL) and washed with aqueous 1M HBF_4 solution (3 x 10 mL). The organic layer was dried over Na_2SO_4, filtrated and evaporated under reduced pressure. The solid obtained was dissolved in CH_2Cl_2 (*ca.* 1 mL) and precipitated by addition of Et_2O (*ca.* 10 mL). The precipitate was separated from the mother liquor by centrifugation. The product was then purified by flash chromatography (CombiFlash, SiO_2 4 g cartridge, CH_2Cl_2/MeOH, 100:0 to 95:5 over 30 min) yielding the corresponding azaoxa [6]helicene **2** as a pink powder.

4. **Experimental evidences**

<u>From dioxa **1** to azaoxa **2**</u>

<u>*In situ* NMR analysis of intermediate **A** (R = *n*-propyl)</u>

To a solution of dioxa **1** (9 mg, 0.02 mmol) in CD_3CN (500 µL) in a NMR tube was added *n*-propylamine (5 µL, 3 equiv). The solution turned to a deep blue color almost instantly. Full NMR analysis was then performed (^1H, ^{13}C, DEPT 135, COSY, HSQC, HMBC, NOESY) at 500 MHz. The ^1H and ^{13}C NMR spectra are depicted below along with the attribution of the signals (Figure S1). **^{1}H NMR (500 MHz, CD_3CN) δ** 8.22 (d, *J* = 9.5 Hz, 1H), 7.97 (d, *J* = 9.6 Hz, 1H), 7.91 (d, *J* = 8.9 Hz, 1H), 7.83 (d, *J* = 9.5 Hz, 1H), 7.79 (d, *J* = 8.5 Hz, 1H), 7.53 (dd, *J* = 8.9, 7.9 Hz, 1H), 7.30 (t, *J* = 7.9 Hz, 1H), 7.25 (d, *J* = 8.9 Hz, 1H), 7.21 (d, *J* = 9.0 Hz, 1H), 7.11 (t, *J* = 8.0 Hz, 1H), 7.01 (t, *J* = 8.5 Hz 1H), 6.95 (d, *J* = 8.5 Hz, 1H), 6.83 (t, *J* = 8.7 Hz, 1H), 6.28 (d, *J* = 8.9 Hz, 1H), 5.99 (d, *J* = 8.9 Hz, 1H), 4.61 (m, 2H), 3.01 (m, 2H), 2.17 (m, 2H), 0.74 (t, *J* = 7.4 Hz, 3H), 0.55 (t, *J* = 7.4 Hz, 3H). **^{13}C NMR (126 MHz, CD_3CN) δ** 181.6 (C), 158.2 (C), 155.8 (C^+), 154.1 (C), 140.2 (C*H*), 139.5 (C*H*), 132.6 (C), 132.1 (C), 131.9 (C), 130.8 (C*H*), 130.3 (C*H*), 129.2 (C*H*), 129.1 (C*H*), 128.6 (C), 127.4 (C*H*), 126.6 (C*H*), 125.8 (C), 125.2 (C*H*), 122.8 (C*H*), 122.7 (C*H*), 121.6 (C*H*), 118.4 (C*H*), 117.4 (C) 116.0 (C), 115.3 (C*H*), 96.2 (C*H*), 45.80 (C*H_2*), 23.5 (C*H_2*), 11.3 (C*H_3*).

^{1}H NMR (500 MHz, CD_3CN):

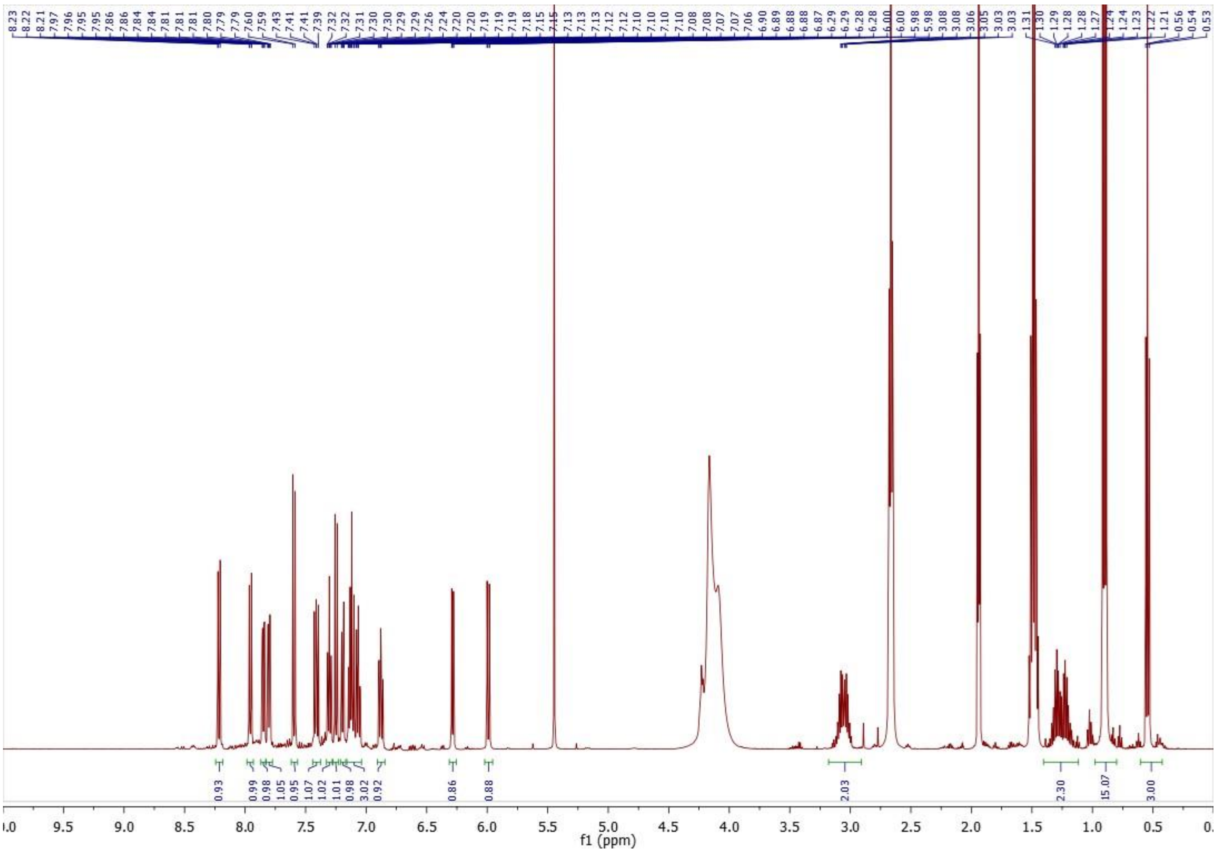

^{13}C NMR (126 MHz, CD_3CN):

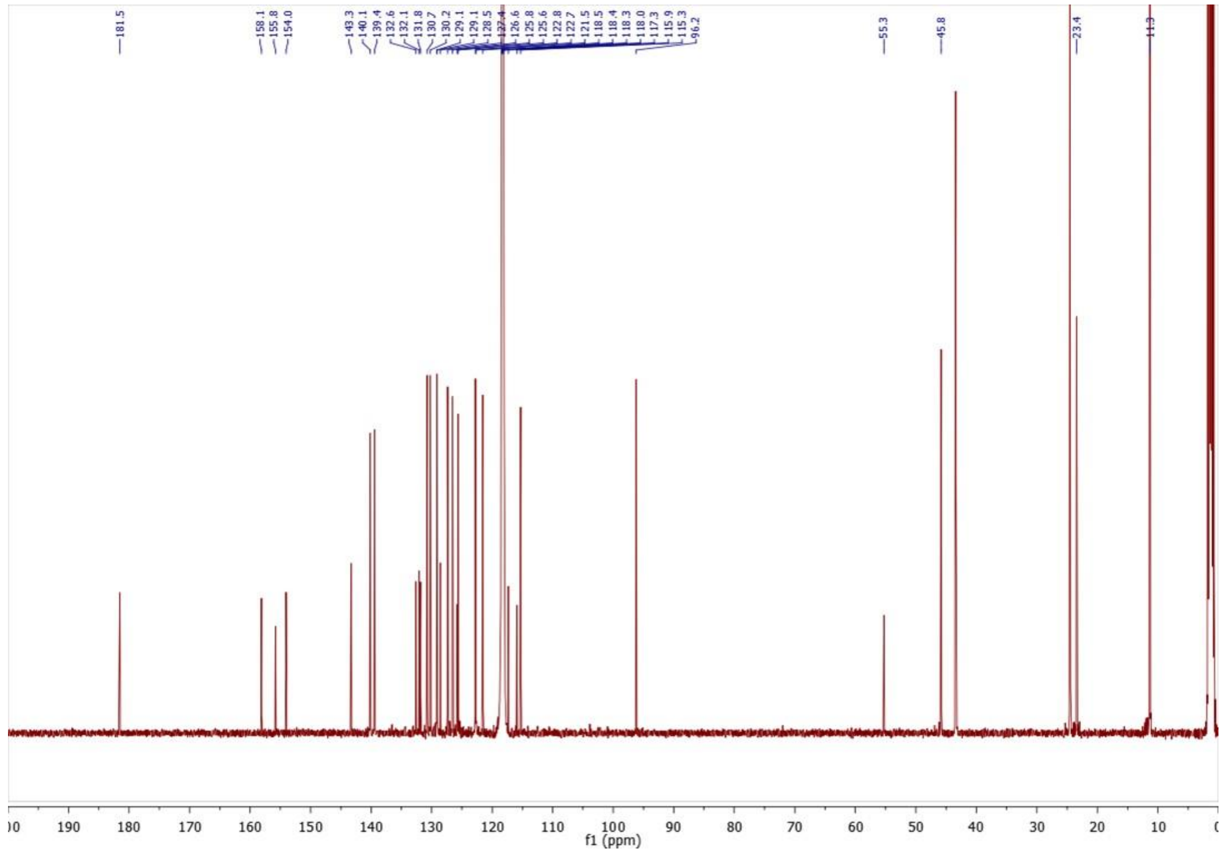

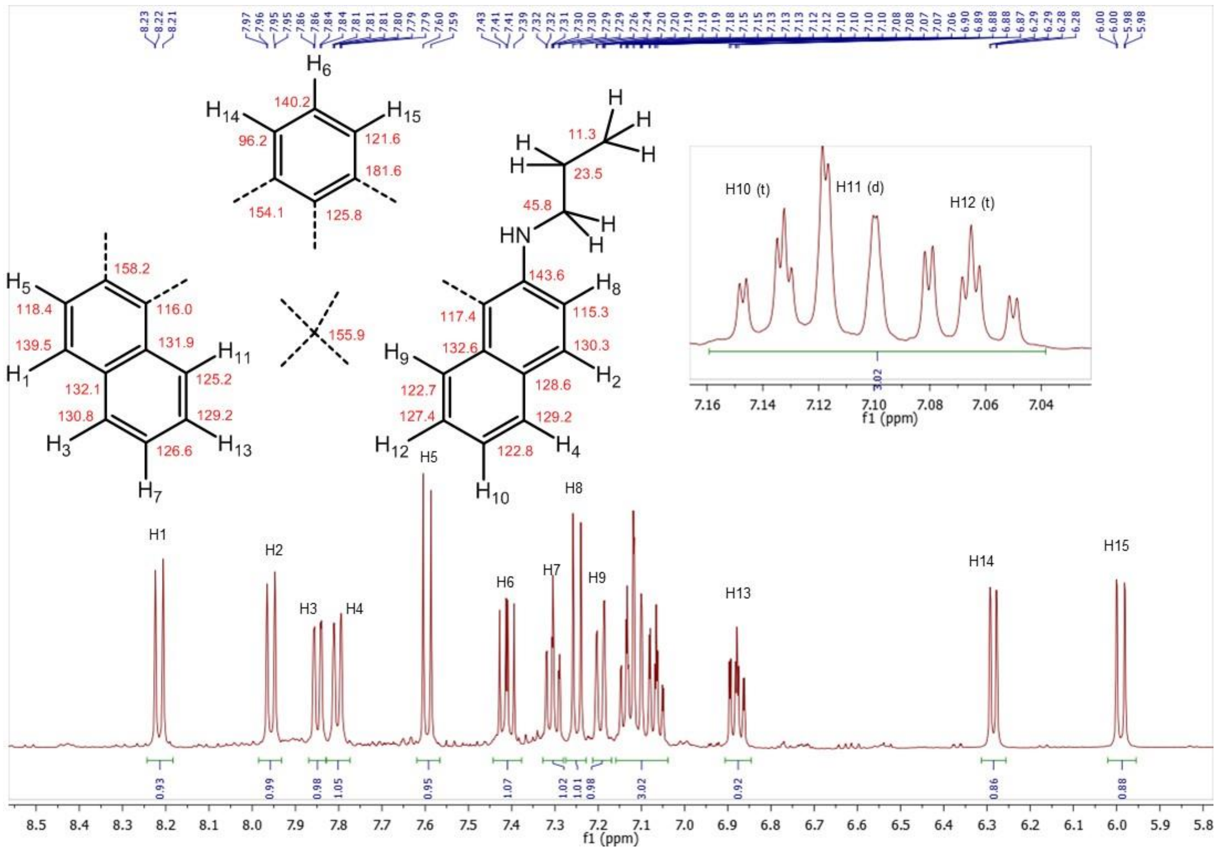

Figure S1: Attribution of the ^{1}H and ^{13}C NMR signals of intermediate **A** (R = *n*-propyl)

Trapping of intermediate **A** with trifluoroacetyl chloride and generation of **4**

13-propyl-12-(trifluoromethyl)-12,13-dihydro-12,19c-epoxybenzo[7,8]xantheno[9,1-fg]naphtho[2,1-d][1,3]oxazocine 4.

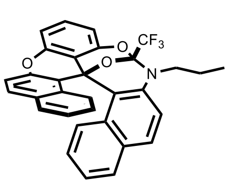

To a solution of dioxa[6]helicene **1** (92 mg, 0.20 mmol) in acetonitrile (2 mL) under nitrogen atmosphere was added *n*-propylamine (50 µL, 0.60 mmol, 3 equiv.). This reaction mixture was stirred 30 min at 20 °C while gradually evolving from a red color to a deep blue color. After this time, trifluoroacetyl chloride solution (4.3 M in dichloromethane, 560 µL, 2.40 mmol, 12 equiv.) was added to the reaction mixture, which immediately turned brown and was further stirred at 20 °C for 16 hours. After this time, the reaction mixture was diluted with dichloromethane, washed with a 1 M aqueous $NaBF_4$ solution (once) and a saturated aqueous NaCl solution (twice). The organic layer was dried over Na_2SO_4, filtered and evaporated. The crude material was purified by flash chromatography on silica gel (cyclohexane / EtOAc, from 100 : 00 to 80 : 20) affording 102 mg of the title compound as an off-white solid in *ca.* 90% purity. Second flash chromatography on silica gel (pentane / Et_2O, from 100 : 00 to 90 : 10) afforded 60 mg of the title compound as a white solid. Slow evaporation of a dichloromethane solution of **5** led to the formation of white crystals. (57 % yield). **MP** 173-175 °C. **Rf:** (pentane / Et_2O 90 : 10): 0.48. **^{1}H NMR (500 MHz, CD_2Cl_2)** δ 8.01 (dd, *J* = 8.0, 1.0 Hz, 1H), 7.88 (dd, *J* = 9.0, 0.7 Hz, 1H), 7.78 (dd, *J* = 10.0, 1.0 Hz, 1H), 7.75 – 7.66 (m, 2H), 7.63 – 7.55 (m, 1H), 7.50 (d, *J* = 9.0 Hz, 1H), 7.41 (d, *J* = 9.2 Hz, 1H), 7.39 – 7.30 (m, 2H), 7.29 – 7.18 (m, 2H), 7.12 – 7.04 (m, 3H), 6.86 (dd, *J* = 8.1, 0.9 Hz, 1H), 4.06 (ddd, *J* = 16.5, 12.0, 4.9 Hz, 1H), 3.68 – 3.55 (m, 1H), 2.12 – 1.99 (m, 1H), 1.93 (tdd, *J* = 12.4, 7.4, 4.9 Hz, 1H), 1.09 (t, *J* = 7.4 Hz, 3H). **^{13}C NMR (126 MHz, CD_2Cl_2)** δ 152.3 (C), 151.3 (C), 147.9 (C), 139.2 (C), 132.6 (CH), 131.6 (C), 131.1 (C), 131.0 (CH), 130.6 (C), 129.9 (CH), 129.3 (C), 129.0 (CH), 128.8 (CH), 127.2 (CH), 126.9 (CH), 125.4 (CH), 124.9 (CH), 123.5 (CH), 122.3 (C), 121.8 (CH), 120.0 (C), 118.9 (C), 117.8 (CH), 115.3 (CH), 114.5 (C), 113.0 (C), 110.5 (CH), 109.6 (CH), 102.3 (q, *J* = 34.0 Hz, CF_3), 71.4 (C), 49.1 (CH_2), 22.5 (CH_2), 11.4 (CH_3). **^{19}F NMR (282 MHz, CD_2Cl_2)** δ -78.62. **IR (neat, cm^{-1}) υ** 3327, 3059, 2966, 2931, 2876, 2255, 2032, 1944, 1694, 1620, 1559, 1480, 1450, 1390, 1363, 1304, 1210, 1139, 1093, 1018, 946, 928, 887, 866, 817, 748, 714, 624, 602. **HRMS** (**ESI**) (M^+) calculated for ($C_{32}H_{22}F_3NO_3$) 526.1630. Found: 526.1625.

Reversibility of the formation of intermediate **A** (**A** ⇆ **A'**)

To a solution of dioxa [6]helicene **1** (4.6 mg, 0.01 mmol, 1 equiv.) in CD_3CN (0.5 mL) in a J-Young NMR tube is added *iso*-propylamine (*i*$PrNH_2$, 1 µL, 0.01 mmol, 1 equiv.). After 5 min, ^1H NMR spectrum is recorded (CD_3CN, 400 MHz, Figures S2 and S3, spectrum A). Then the mixture is transferred in a 5 mL flask under N_2 atmosphere and is evaporated using schlenk technics. The residue is next dissolved in CD_3CN (0.5 mL) under N_2 atmosphere and *n*-propylamine (*n*$PrNH_2$, 5 µL, 0.05 mmol, 5 equiv.) is added. After 15 min, the flask content is evaporated using schlenk technics. The residue is next dissolved in CD_3CN (0.5 mL) and ^1H NMR spectrum is recorded (400 MHz, Figure S2 and S3, spectrum B).

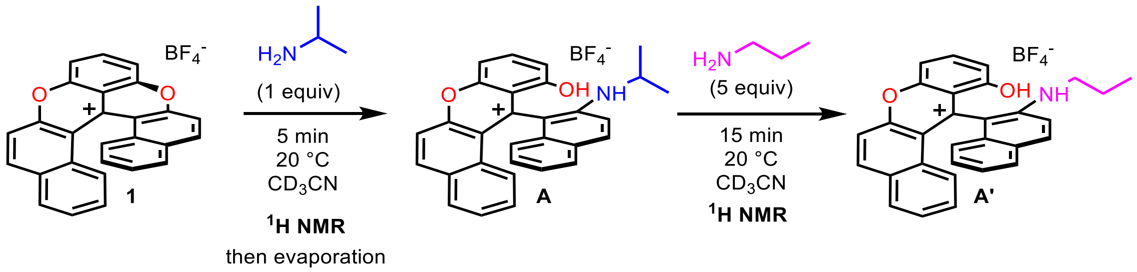

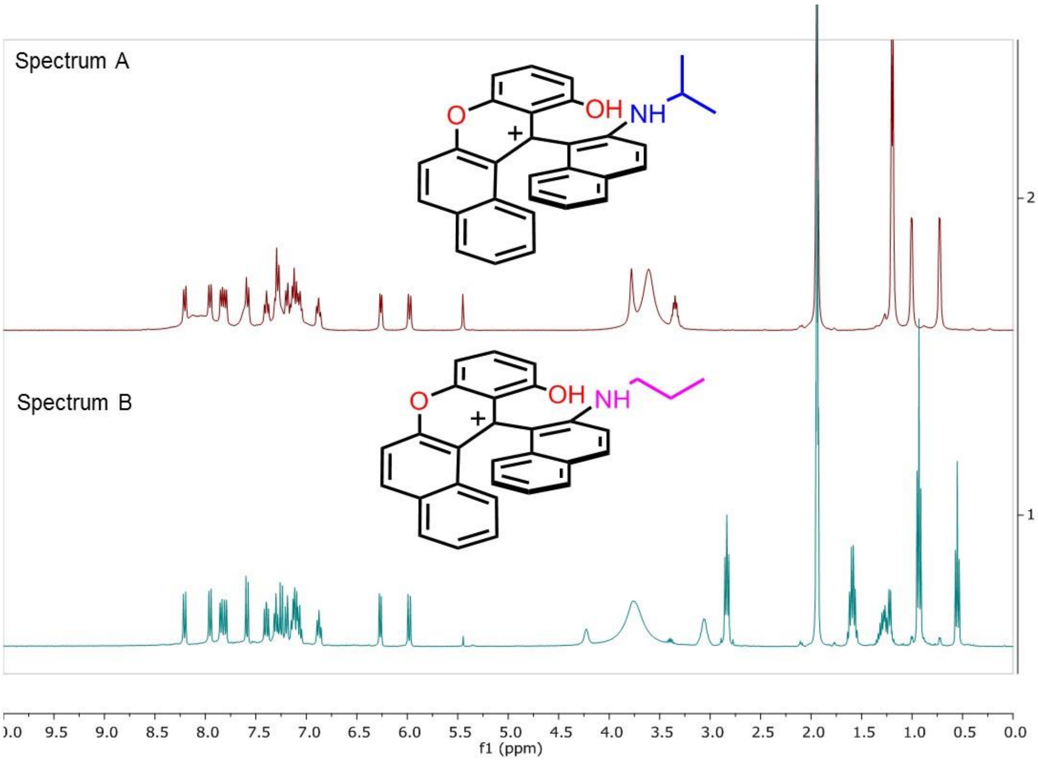

Figure S2. Spectrum A: Full ^1H NMR spectrum (CD_3CN, 400 MHz) of dioxa [6]helicene **1** and $^iPrNH_2$ (intermediate **A**) Spectrum B: Full ^1H NMR spectrum (CD_3CN, 400 MHz) after evaporation of $^iPrNH_2$ and treatment with $^nPrNH_2$ (intermediate **A'**).

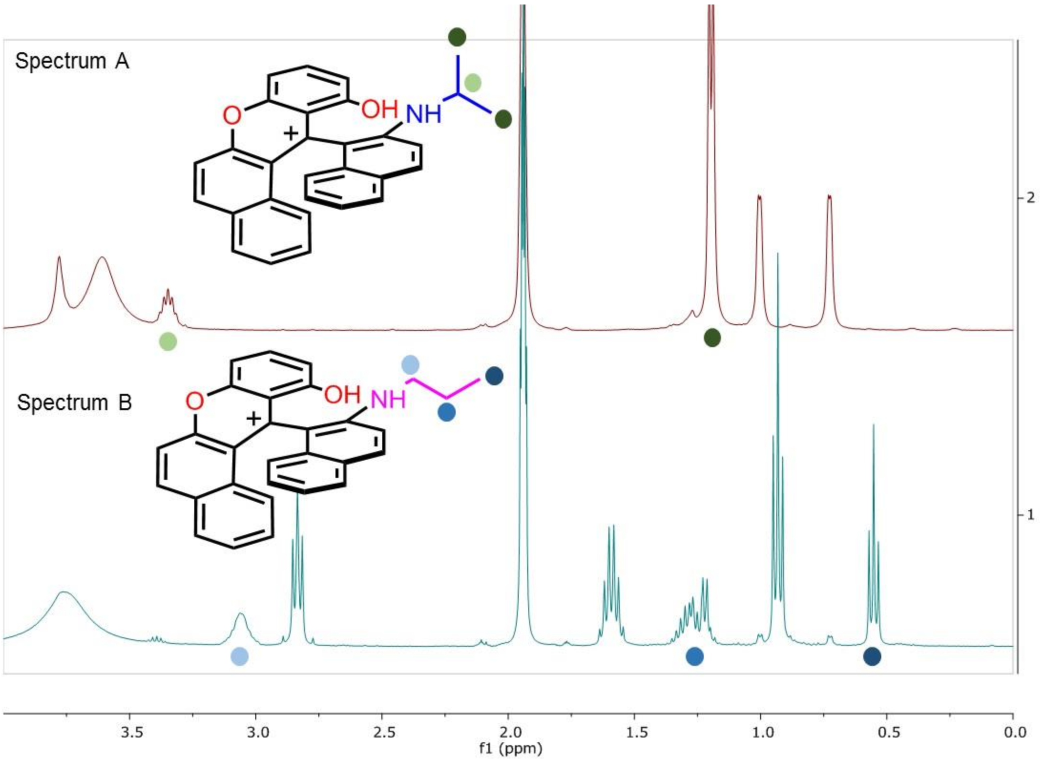

Figure S3. Spectrum A: Zoom in the ^{1}H NMR spectrum (CD_3CN, 400 MHz) of dioxa [6]helicene **1** and $^{i}PrNH_2$. Spectrum B: Zoom in ^{1}H NMR spectrum (CD_3CN, 400 MHz) after evaporation of $^{i}PrNH_2$ and treatment with $^{n}PrNH_2$.

Experimental evidence that **A** (R = *n*-propyl) is a productive intermediate from **1** to **2**

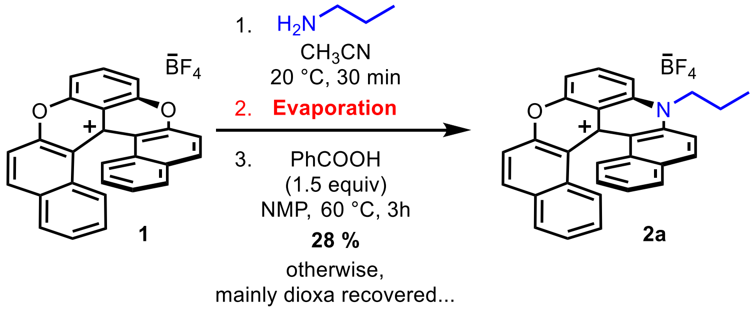

Scheme S3. Formation of **2a** from intermediate **A**.

To a solution of dioxa [6]helicene **1** (23 mg, 0.05 mmol, 1 equiv.) in CH_3CN (1 mL) was added $^{n}PrNH_2$ (12 µL, 0.15 mmol, 3 equiv.). The reaction mixture was stirred for 1 hour at 20 °C. After this time, the reaction mixture was evaporated using schlenk technics affording intermediate **A**. Intermediate **A** was next dissolved in dry NMP (1 mL) and benzoic acid (9 mg, 0.075 mmol, 1.5 equiv.) was added to the reaction mixture which was stirred at 60 °C for 3 hours. After this time, the reaction mixture was allowed to cool back to 20 °C and was diluted with CH_2Cl_2 (5 mL). Addition of Et_2O (40 mL) led to the precipitation of the products which were separated from the mother liquor by centrifugation. The precipitate was next dissolved in CH_2Cl_2 and washed with a 1 M aq. HBF_4 solution. The organic layer was dried over Na_2SO_4, filtered and evaporated affording 13 mg of crude material that were further purified by flash chromatography (CombiFlash, SiO_2 4 g, CH_2Cl_2 / CH_3OH, 100:00 to 95:05). 7 mg of **2a** were isolated (Yield: 28%).

Stereoselectivity of the O-ring opening during the formation of **A** (R = *enantiopure amine*)

The formation of intermediate of type **A** was performed using enantiopure (–)-(*M*)- and (+)-(*P*)-dioxa [6]helicene **1** and enantiopure (*S*)-methyl-*tert*butylamine acting as a stereogenic probe. The resulting biarylic system possesses thus two stereogenic elements.

To a solution of (–)-(*M*)-dioxa [6]helicene **1** (4.6 mg, 0.01 mmol, 1 equiv.) in CD_3CN (0.5 mL) in a J-Young NMR tube under N_2 atmosphere and protected from light was added (*S*)-methyl-*tert*butylamine (4 µL, 0.03 mmol, 3 equiv.). After 5 min at 20 °C, ^{1}H NMR spectrum was recorded (CD_3CN, 400 MHz, Figures S4, spectrum A). The NMR tube was heated for 4 h at 60°C and ^{1}H NMR spectra were recorded after 1 h, 2 h, 3 h and 4 h (CD_3CN, 400 MHz, spectrum B-E). Spectrum F corresponds to the addition of (*S*)-methyl-*tert*-butylamine on racemic dioxa **1**.

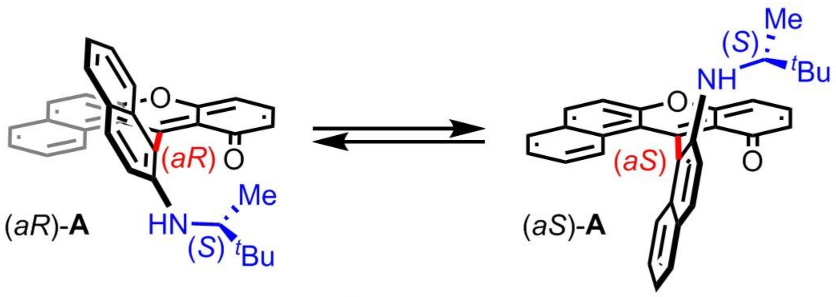

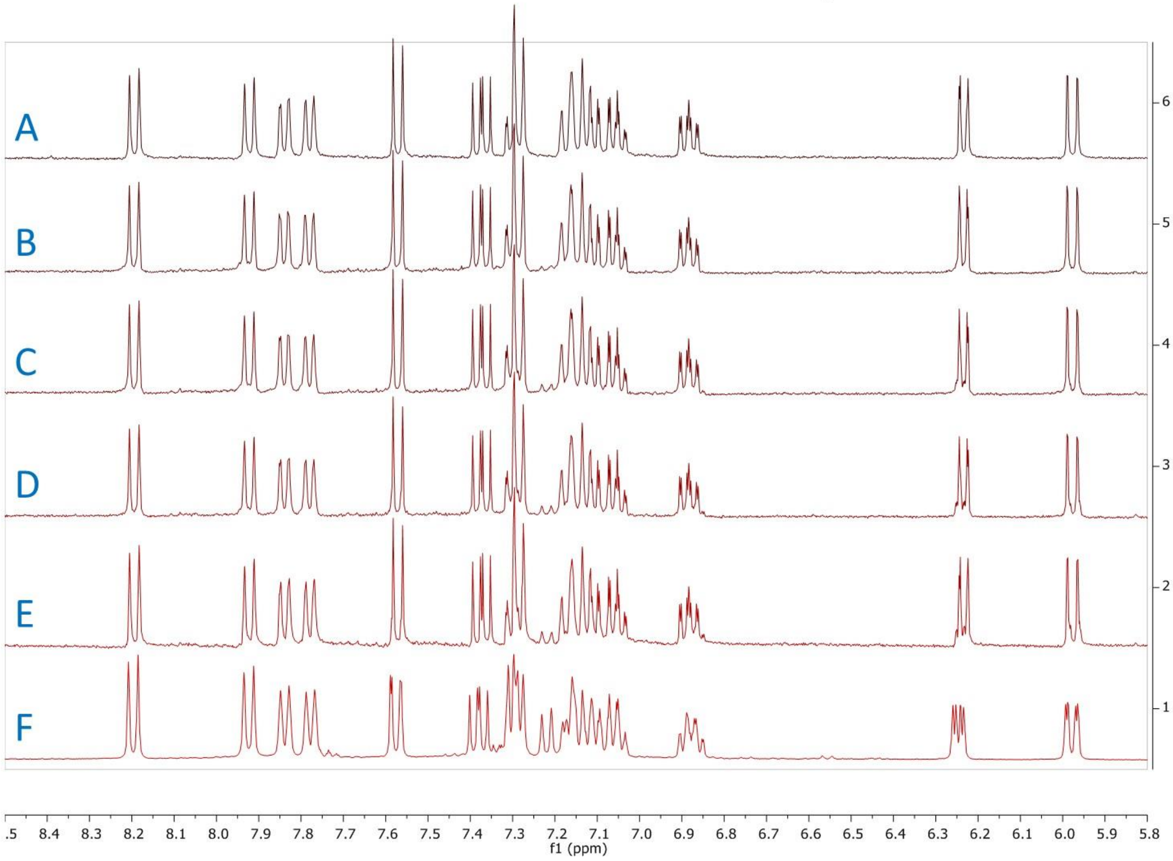

Figure S4. ^{1}H NMR (CD_3CN, 400 MHz) monitoring of the addition of (*S*)-methyl-*tert*-butyl amine on (–)-(*M*)-dioxa **1**. A: 5 min after addition, B: after 1 h at 60 °C, C: after 2 h at 60 °C, D: after 3 h at 60 °C, E: after 4 h at 60 °C, F: (*S*)-methyl-*tert*-butyl amine added on racemic dioxa **1**, after 5 min.

To a solution of (+)-(*P*)-dioxa [6]helicene **1** (4.6 mg, 0.01 mmol, 1 equiv.) in CD_3CN (0.5 mL) in a J-Young NMR tube under N_2 atmosphere and protected from light was added (*S*)-methyl-*tert*butylamine (4 µL, 0.03 mmol, 3 equiv.). After 5 min at 20 °C, ^1H NMR spectrum was recorded (CD_3CN, 400 MHz, Figures S5, spectrum A). The NMR tube was heated for 4 h at 60°C and ^1H NMR spectra were recorded after 1 h, 2 h, 3 h and 4 h (CD_3CN, 400 MHz, spectrum B-E). Spectrum F corresponds to the addition of (*S*)-methyl-*tert*-butylamine on racemic dioxa **1**.

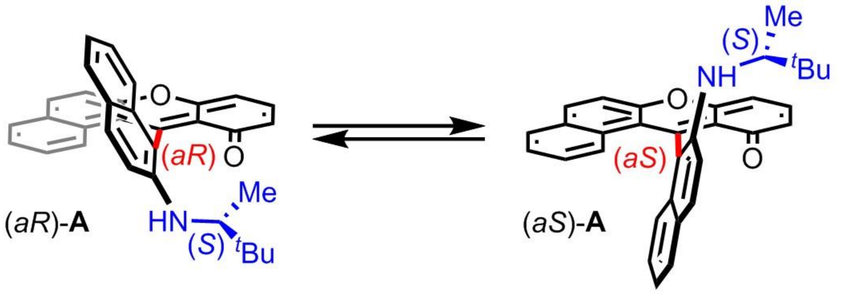

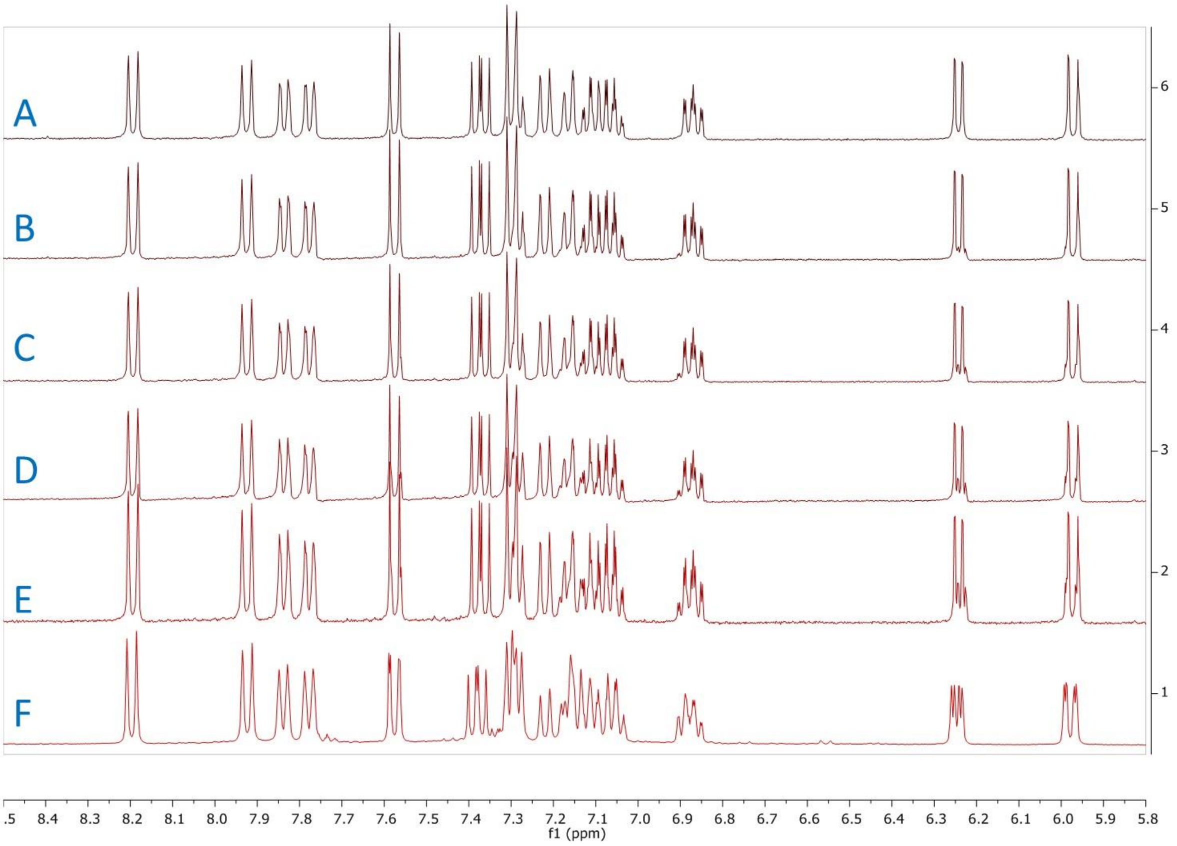

Figure S5. ^1H NMR (CD_3CN, 400 MHz) monitoring of the addition of (*S*)-methyl-*tert*-butyl amine on (+)-(*P*)-dioxa **1**. A: 5 min after addition, B: after 1 h at 60 °C, C: after 2 h at 60 °C, D: after 3 h at 60 °C, E: after 4 h at 60 °C, F: (*S*)-methyl-*tert*-butyl amine added on racemic dioxa **1**, after 5 min.

Configurational stability of intermediate **A** (R = *n*-propyl)

The configurational stability of Intermediate **A** was determined by Electronic Circular Dichroism (ECD). A solution of (+)-*P*-dioxa [6]helicene **1** in CH_3CN (2 10^{-5} M) was placed in cuvette in a CD measurement apparatus which was heated to 50 °C. ECD spectra were recorded immediately after the addition of an excess of propylamine and then every 10 minutes over a period of 90 minutes. Recorded spectra are gathered in Figure S6 and S7.

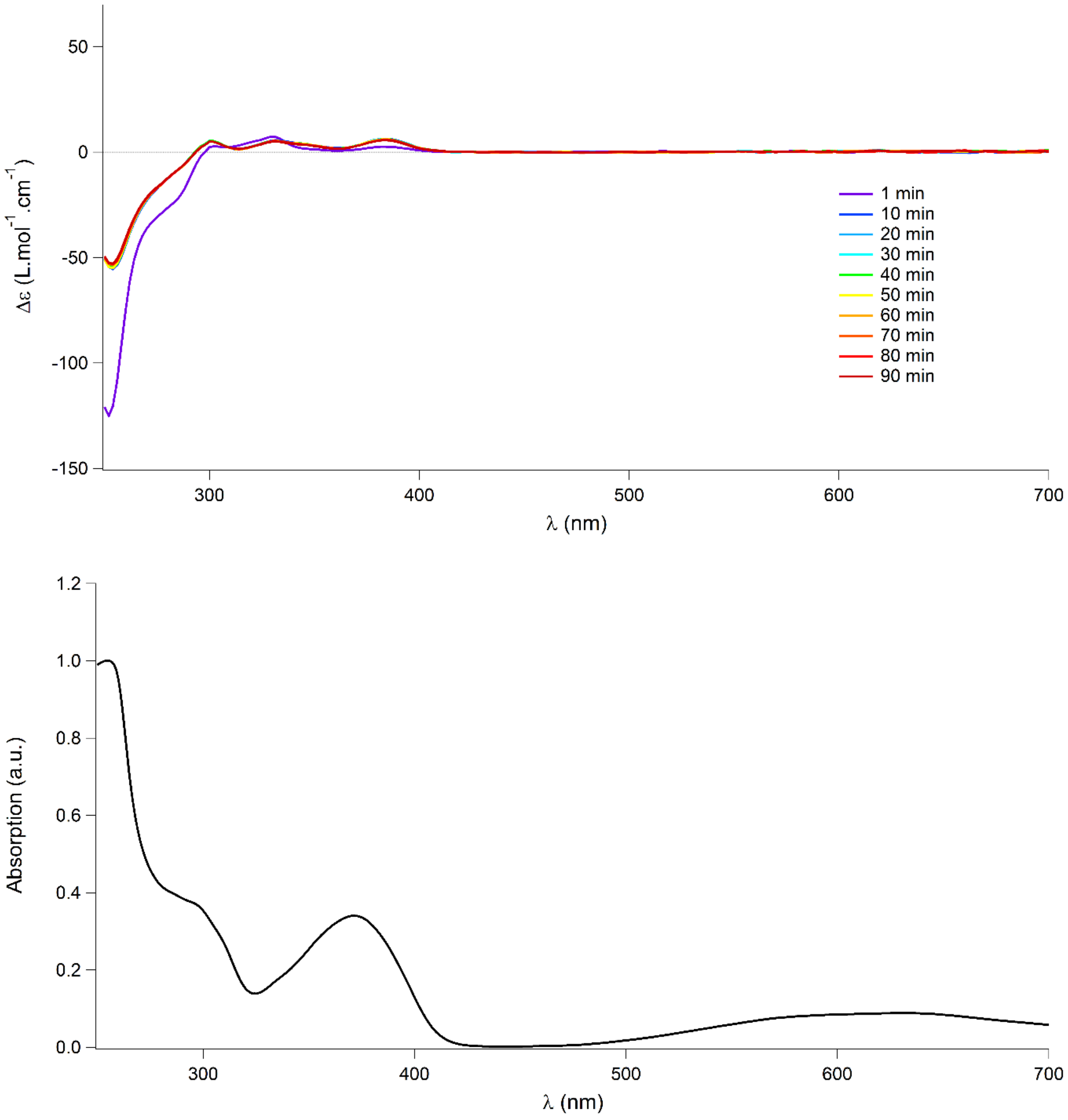

Figure S6. Top: ECD monitoring of the configuration stability of intermediate **A** prepared from (+)-*P*-dioxa [6]helicene **1** and *n*-propylamine. Bottom: normalized absorption spectrum of intermediate **A** prepared from (+)-*P*-dioxa [6]helicene **1** and *n*-propylamine.

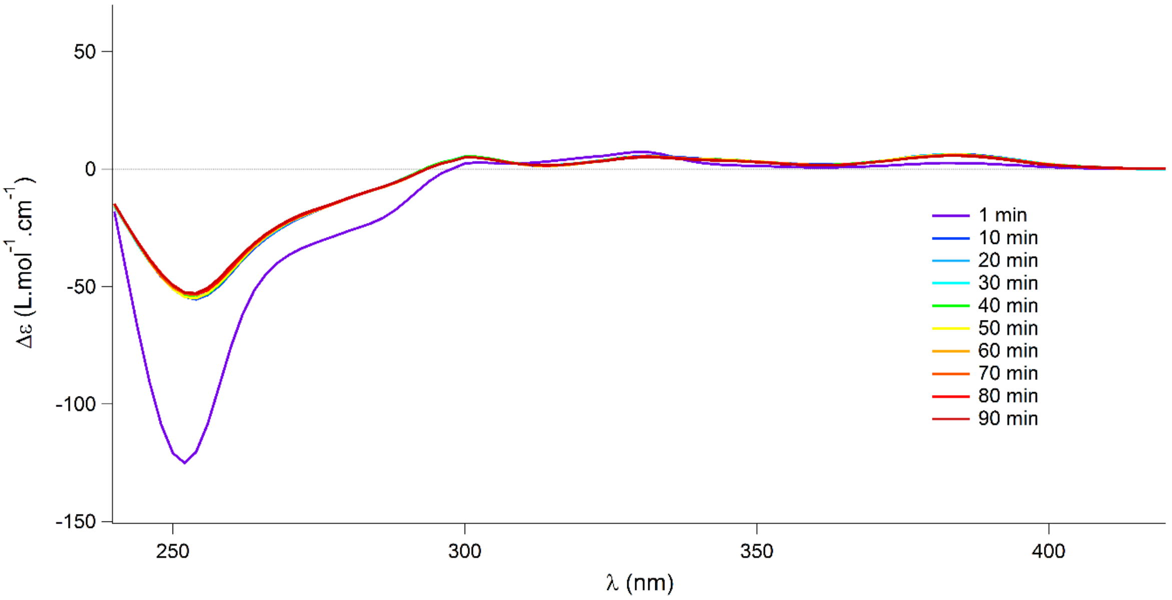

Figure S7. Zoom of the ECD monitoring of the configuration stability of intermediate **A** prepared from (+)-*P*-dioxa [6]helicene **1** and *n*-propylamine.

From azaoxa **2** to diaza **3**

In situ NMR analysis of intermediate **G** (R = *n*-propyl)

To a solution of *rac*-azaoxa **2a** (15.6 mg, 0.03 mmol) in 0.75 mL of CD_3CN was added 25 equiv of *n*-propylamine (70 µL, 0.85 mmol). A blue solution is formed after 30 min at 25 °C and a complete NMR analysis is recorded at 25 °C. The ^1H and ^{13}C NMR spectra are depicted below along with the attribution of the signals (Figure S6).**^1H NMR (500 MHz, CD_3CN) δ** 8.39 (d, *J* = 10.0 Hz,, 1H), 8.15 (d, *J* = 9.7 Hz, 1H), 8.01 (d, *J* = 9.5 Hz,, 1H), 7.93 (dd, *J* = 7.9, 1.5 Hz, 1H), 7.85 (dt, *J* = 8.1, 0.9 Hz, 1H), 7.71 (t, *J* = 8.4 Hz, 1H), 7.51 – 7.42 (m, 2H), 7.23 (d, *J* = 9.0 Hz, 1H), 7.19 – 7.10 (m, 2H), 6.99 (ddd, *J* = 8.2, 6.7, 1.4 Hz, 1H), 6.94 (ddd, *J* = 8.8, 7.0, 1.6 Hz, 1H), 6.79 (dd, *J* = 8.5, 1.0 Hz, 1H), 5.73 (d, *J* = 8.3 Hz, 1H), 4.93 (t, *J* = 8.7 Hz, 2H), 2.99 (ddd, *J* = 13.5, 7.6, 6.1 Hz, 1H), 2.87 (dt, *J* = 13.7, 7.0 Hz, 1H), 2.77 – 2.62 (m, 1H), 2.36 – 2.20 (m, 2H), 1.33 (t, *J* = 7.3 Hz, 3H), 1.12 (dt, *J* = 14.0, 7.1 Hz, 1H), 1.02 (qd, *J* = 7.4, 6.1, 4.1 Hz, 1H), 0.90 (t, *J* = 7.5 Hz, 3H), 0.39 (t, *J* = 7.4 Hz, 3H). **^{13}C NMR (126 MHz, CD_3CN) δ** 170.3 (C), 154.6 (C^+), 143.9 (C), 142.9 (C), 142.0 (C), 141.1 (C*H*), 140.1 (C*H*), 133.5 (C), 131.5 (C), 131.3 (C), 130.4 (C*H*), 130.4 (C*H*), 130.2 (C*H*), 129.3 (C*H*), 129.1 (C*H*), 128.5 (C), 128.4 (C*H*), 127.9 (C*H*), 127.9 (C*H*), 127.3 (C*H*), 124.2 (C), 122.8 (C*H*), 122.8 (C*H*), 122.1 (C), 119.6 (C), 118.5 (C), 118.3 (C), 118.0 (C), 117.1 (C*H*), 115.4 (C*H*), 113.8 (C*H*), 100.2 (C*H*), 54.0 (CH_2), 45.8 (CH_2), 42.7 (CH_2), 23.3 (CH_2), 21.8 (CH_2), 21.6 (CH_2), 11.2 (CH_3), 11.2 (CH_3), 11.0 (CH_3).

^1H NMR (500 MHz, CD_3CN):

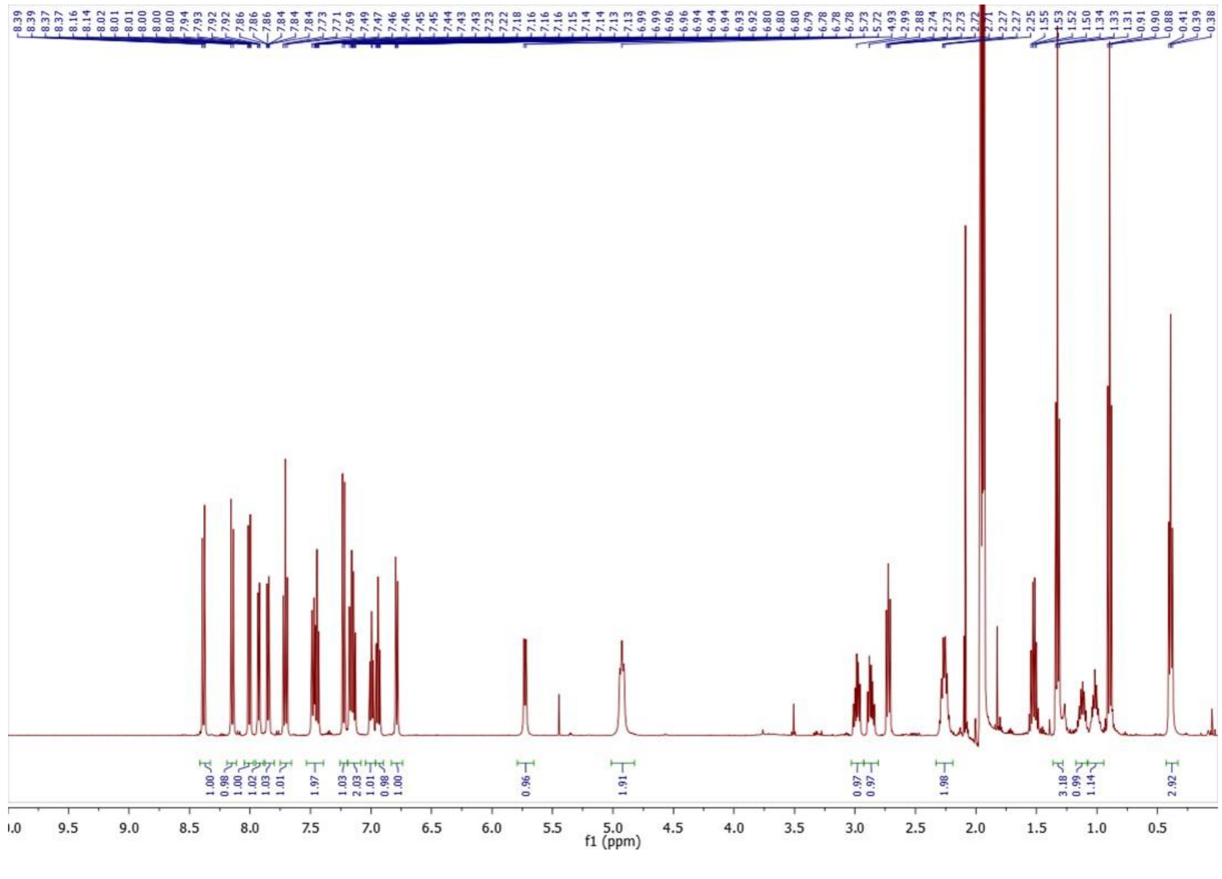

^{13}C NMR (126 MHz, CD_3CN):

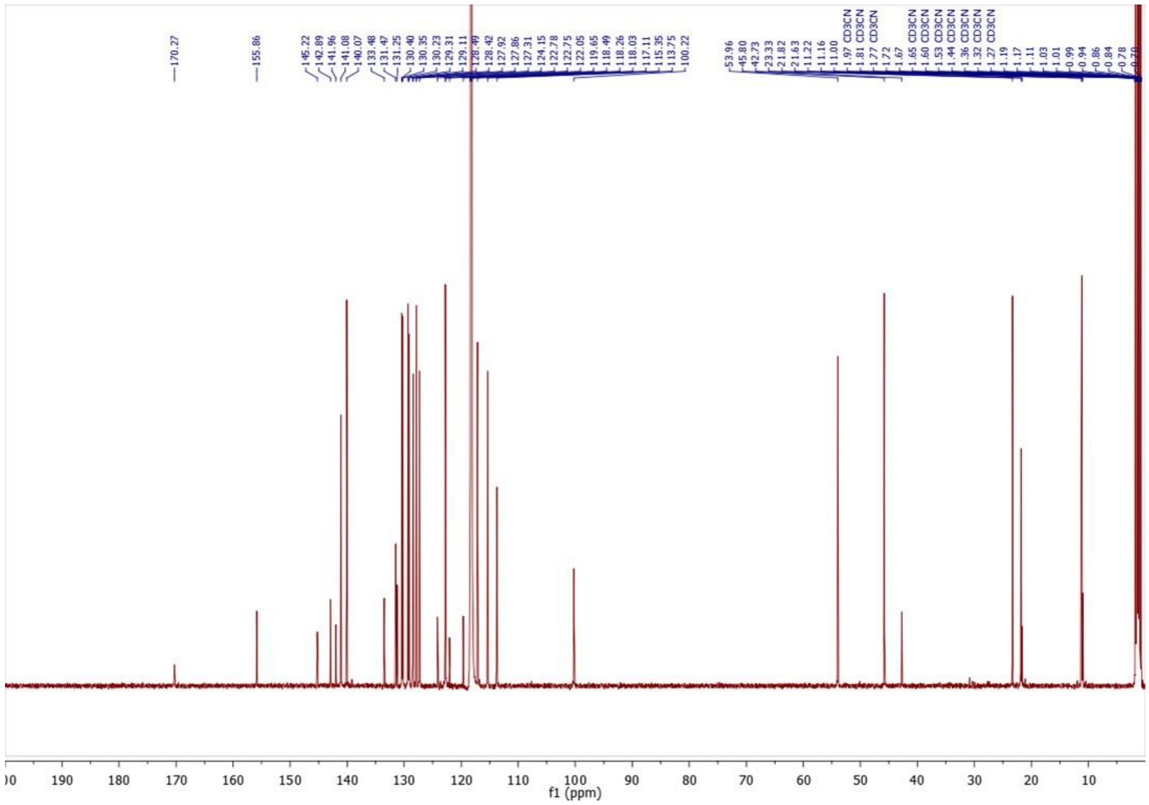

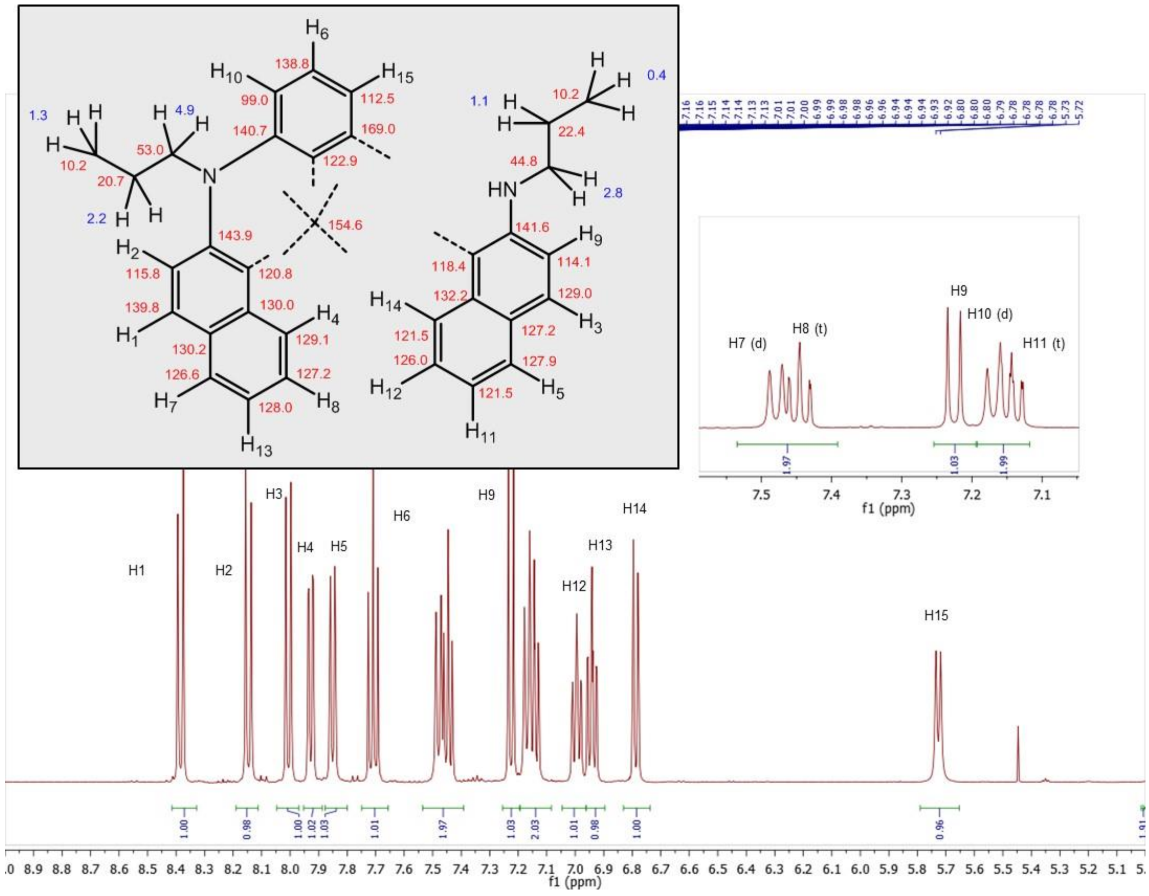

Figure S8: Attribution of the ^{1}H and ^{13}C NMR signals of intermediate **G**

Trapping of intermediate **G** with *para*-chlorobenzoyl chloride and generation of **6**

11-((4-chlorobenzoyl)oxy)-7-propyl-12-(2-(propylamino)naphthalen-1-yl)-7,12-dihydrobenzo[a]acridin-12-ylium tetrafluoroborate 6.

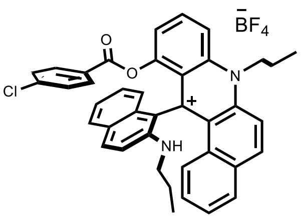

To a solution of azaoxa [6]helicene **2a** (25 mg, 0.05 mmol) in acetonitrile (1 mL) under N_2 atmosphere was added *n*-propylamine (100 µL, 1.25 mmol, 25 equiv.). The reaction mixture was stirred at 20 °C for 2 hours. After this time, the reaction mixture was evaporated to dryness using Schlenk technics. The residue was then dissolved in dry dichloromethane (1.5 mL) under N_2 atmosphere and 4-dimethylaminopyridine (DMAP, 30 mg, 0.25 mmol, 5 equiv.) and then *para*-chlorobenzoyl chloride (44 mg, 0.25 mmol, 5 equiv.) were added to this solution. The reaction mixture was stirred for 30 minutes at 20 °C. The reaction mixture was then diluted with dichloromethane and washed (3 x) with a 1 M aq. HBF_4 solution. The organic layer was then dried over Na_2SO_4, filtered and evaporated. The residue was dissolved in dichloromethane (5 mL) and precipitated with a 1:1 mixture of diethyl ether and pentane (40 mL). The precipitate was separated from the mother liquor by centrifugation. The residue was next purified by flash column chromatography (SiO_2, 10 x 1 cm, CH_2Cl_2/MeOH from 100:00 to 98.5:01.5). The title compound was isolated as a dark green solid (yellow-green in dichloromethane solutions). Diffusion of toluene in a dichloromethane solution of **6** led the formation of crystals. 22 mg, 0.0316 mmol, 63% yield. **MP** 151-153 °C. **Rf:** CH_2Cl_2/MeOH 95:05): 0.14. **^{1}H NMR (500 MHz, CD_2Cl_2) δ** 8.61 (t, *J* = 8.7 Hz, 2H), 8.44 (dd, *J* = 9.2, 7.6 Hz, 1H), 8.30 (d, *J* = 9.7 Hz, 1H), 8.02 (dd, *J* = 7.7, 1.5 Hz, 1H), 7.66 – 7.53 (m, 3H), 7.49 (dd, *J* = 9.1, 0.8 Hz, 1H), 7.36 – 7.27 (m, 3H), 7.21 (td, *J* = 6.5, 5.8, 2.3 Hz, 3H), 7.15 (t, *J* = 7.8 Hz, 1H), 7.08 (ddd, *J* = 8.8, 7.1, 1.6 Hz, 1H), 6.88 (d, *J* = 9.2 Hz, 1H), 6.78 (d, *J* = 8.5 Hz, 1H), 5.49 – 5.41 (m, 2H), 3.72 (brs, 1H), 3.13 – 2.79 (m, 2H), 2.65 – 2.47 (m, 2H), 1.53 (t, *J* = 7.4 Hz, 3H), 1.31 – 1.13 (m, 2H), 0.61 (t, *J* = 7.4 Hz, 3H). **^{13}C NMR (126 MHz, CD_2 Cl_2) δ** 164.1 (C), 153.3 (C), 150.2 (C), 146.4 (C), 144.7 (CH), 141.0 (C), 140.7 (C), 137.3 (CH), 132.8 (C), 132.3 (CH), 132.0 (CH), 131.9 (C), 130.7 (CH), 130.5 (CH), 130.4 (CH), 130.1 (C), 129.2 (CH), 128.9 (CH), 128.5 (C), 128.4 (CH), 128.2 (CH), 128.0 (C), 126.7 (C), 124.2 (CH), 124.0 (C), 123.3 (CH), 122.4 (CH), 117.6 (CH), 116.4 (CH), 115.3 (CH), 114.7 (C), 55.9 (CH_2), 46.0 (CH_2), 23.2 (CH_2), 23.2 (CH_2), 11.6 (CH_3), 11.5 (CH_3). **^{19}F NMR (282 MHz, CD_2Cl_2) δ** -153.13, -153.18. **HRMS** (**ESI**) (M^+) calculated for ($C_{40}H_{34}ClN_2O_2$): 609.2303. Found: 609.2307.

Evidence that **G** is a productive intermediate of the reaction from **2** to **3**

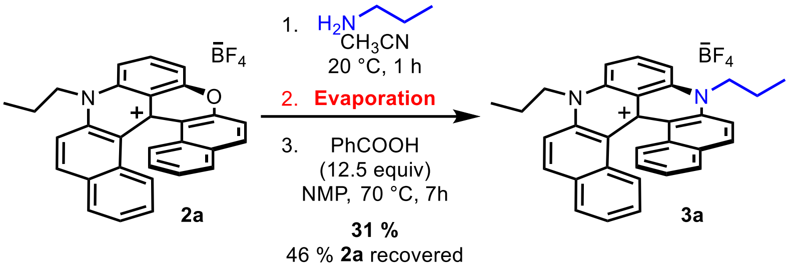

Scheme S4. Formation of **3a** from intermediate **G**.

To a solution of azaoxa [6]helicene **2a** (25 mg, 0.05 mmol, 1 equiv.) in CH_3CN (1 mL) was added $^nPrNH_2$ (103 µL, 1.25 mmol, 25 equiv.). The reaction mixture was stirred for 1 hour at 20 °C. After this time, the reaction mixture was evaporated using schlenk technics affording intermediate **G**. Intermediate **G** was next dissolved in dry NMP (1 mL) and benzoic acid (76 mg, 0.625 mmol, 12.5 equiv.) was added to the reaction mixture which was stirred at 70 °C for 7 hours. After this time, the reaction mixture was allowed to cool back to 20 °C and was diluted with CH_2Cl_2 (5 mL). Addition of Et_2O (40 mL) led to the precipitation of the products which were separated from the mother liquor by centrifugation. The precipitate was next dissolved in CH_2Cl_2 and washed with a 1 M aq. HBF_4 solution. The organic layer was dried over Na_2SO_4, filtered and evaporated affording 13 mg of crude material that were further purified by flash chromatography (CombiFlash, SiO_2 4 g, CH_2Cl_2 / CH_3OH, 100:00 to 95:05). 8 mg of **3a** were isolated (Yield: 31%) along with 9 mg of recovered azaoxa **2a** (46%).

Stereoselectivity of the O-ring opening during the formation of **G**

The formation of intermediate of type **G** was performed using enantiopure (+)-(*P*)-azaoxa [6]helicene **2a** and enantiopure (*S*)-methyl-*tert*butylamine acting as a stereogenic probe.

To a solution of azaoxa [6]helicene (+)-(*P*)-**2a** (5.0 mg, 0.01 mmol) in CD_3CN (0.5 mL) in a J-Young NMR tube was added (*S*)-methyl-*tert*butylamine (33 µL, 25 equiv.). After 30 min at 25 °C, ^1H NMR spectrum was recorded (CD_3CN, 400 MHz, Figures S7, spectrum a) and revealed the formation of a single diastereoisomer. The NMR tube was heated for 7 h at 70°C and a ^1H NMR spectrum was recorded (CD_3CN, 400 MHz, spectrum b).

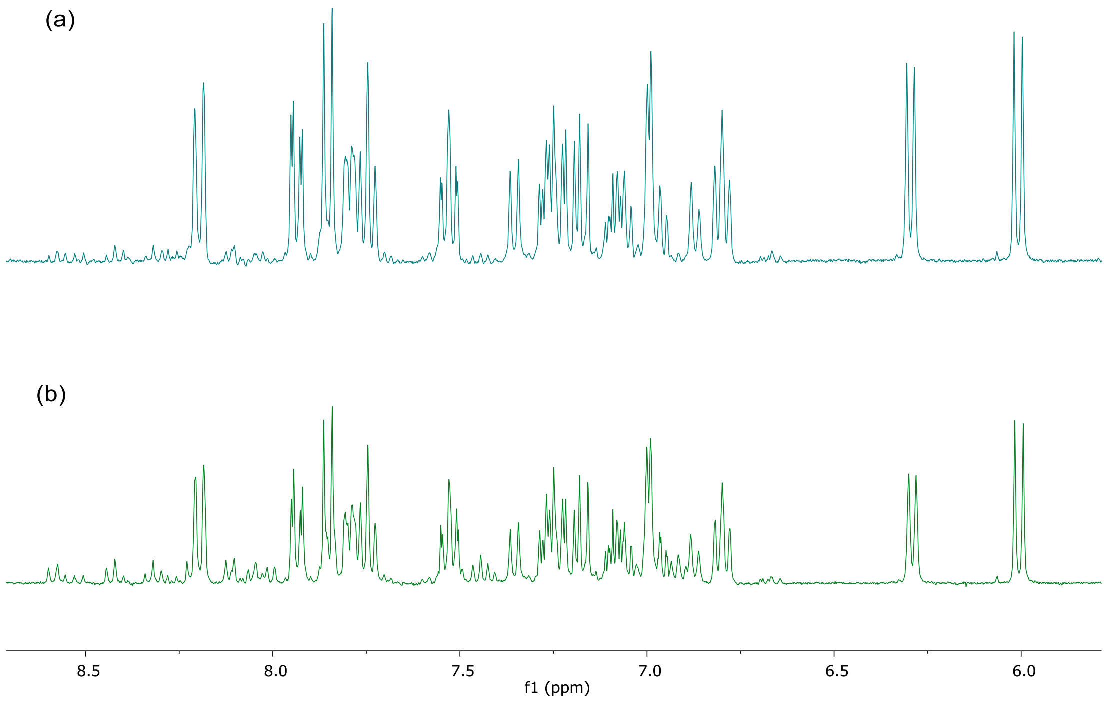

Figures S9. ^1H NMR (400 MHz, CD_3CN) monitoring with the addition of (*S*)-methyl-*tert*-butyl amine on (a) (+)-(*P*)-**2a**. (b) after heating 7 h at 70 °C.

5. Transformations from 1 to 2 to 3 in enantio-enriched series

The enantiomers of dioxa **1**, azaoxa **2a** and diaza **3a** [6]helicenes can be separated by CSP-HPLC, either directly as cationic compounds on analytical scale or under the form of neutral *leuco* adducts on semi preparative scale (Scheme S5).[2]

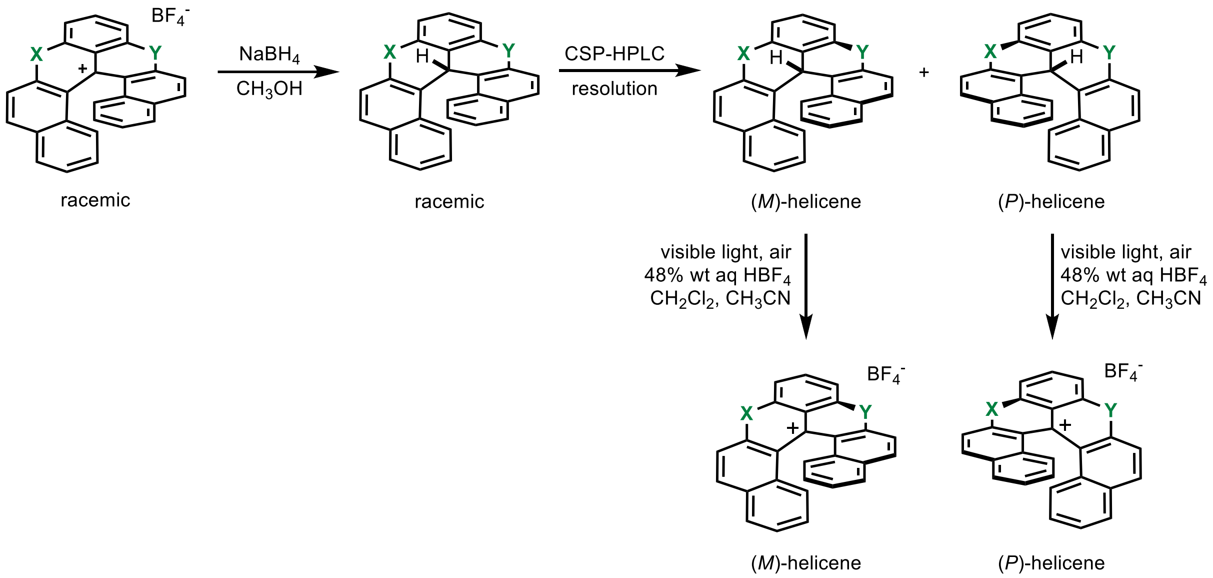

Scheme S5. Semi preparative resolution protocol of dioxa **1** (X = Y = O), azaoxa **2a** (X = O, Y = NPr) and diaza **3a** (X = Y = NPr) *via* the corresponding neutral *leuco* adducts.

From dioxa 1 to azaoxa 2a

Using 3 equiv of primary amine

Procedure:

1) To a solution of (+)-(*P*)- or (–)-(*M*)-dioxa [6]helicene **1** (23 mg, 0.05 mmol) in NMP (0.5 mL) were added benzoic acid (9 mg, 1.5 equiv) amine (12 µL, 3 equiv). The mixture was stirred at 60 °C for 3 hours. The reaction mixture was then cooled to 20 °C. Et_2O (*ca.* 10 mL) was added leading to the precipitation of the crude material. The resulting solid was dissolved in CH_2Cl_2 (*ca.* 5 mL) and washed with aqueous 1M HBF_4 solution (3 x 10 mL). The organic layer was dried over Na_2SO_4, filtrated and evaporated under reduced pressure. The solid obtained was dissolved in CH_2Cl_2 (*ca.* 1 mL) and precipitated by addition of Et_2O (*ca.* 10 mL). The precipitate was separated from the mother liquor by centrifugation.

2) A sample of the precipitated (ca. 5 mg) was dissolved in CH_3OH (1 mL) in a tinted flask in the absence of light and under N_2 atmosphere. Excess of $NaBH_4$ was added to the mixture, inducing an immediate color loss. The reaction mixture was stirred for 30 minutes at 20 °C and then evaporated to dryness.

The residue was dissolved in Et_2O (ca. 2 mL) and filtered through a short plug of Celite. The filtrate was evaporated and subjected to CSP-HPLC analysis.

(–)-(*M*) dioxa **1** to "(–)-(*M*)" azaoxa **2a**

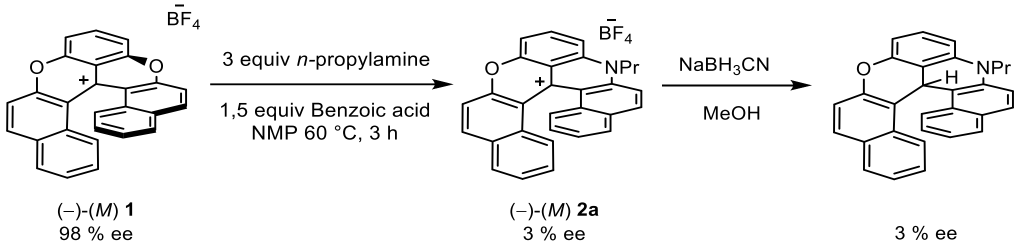

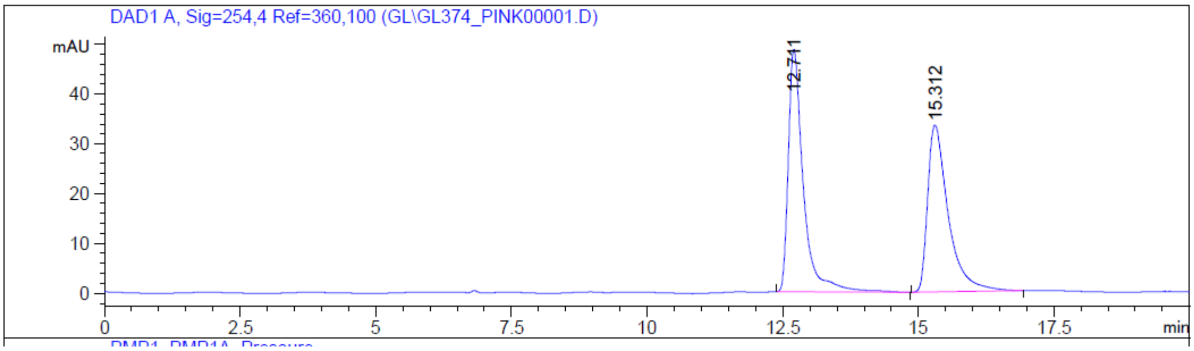

Peak #	RetTime [min]	Type	Width [min]	Area [mAU*s]	Height [mAU]	Area %
1	12.711	BB	0.2851	938.56561	48.63794	51.5766
2	15.312	BB	0.3930	881.18439	33.48461	48.4234

HPLC Conditions: Chiral IB; Hex/IPA 99:1, 0.5 ml/min; 23 °C

Figure S10. From (–)-(*M*) dioxa **1** to "(–)-(*M*)" azaoxa **2a** using optimized conditions

(+)-(*P*) dioxa **1** to "(+)-(*P*)" azaoxa **2a**

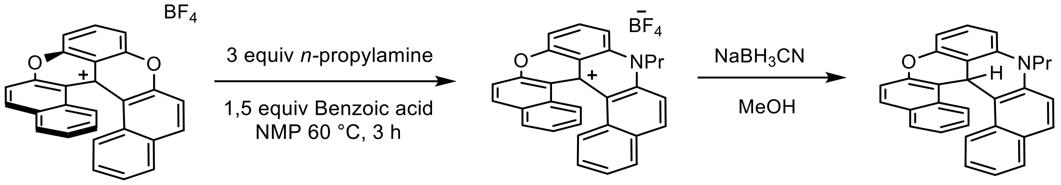

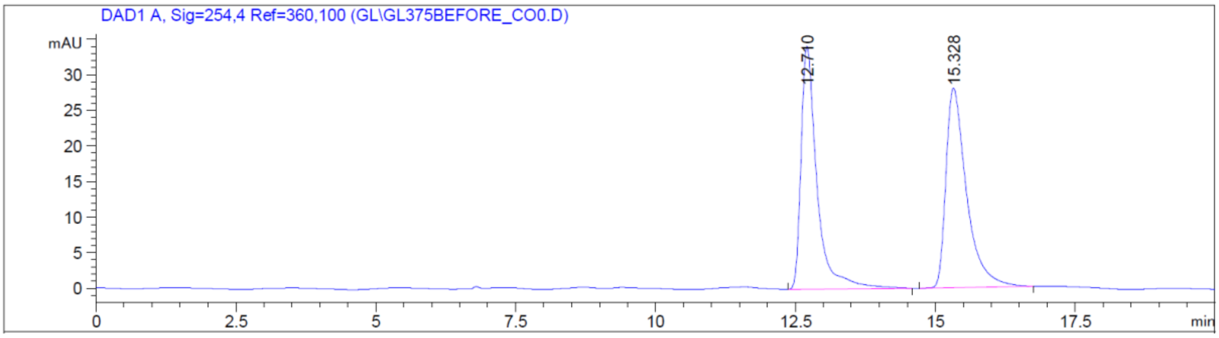

Signal 1: DAD1 A, Sig=254,4 Ref=360,100

Peak #	RetTime [min]	Type	Width [min]	Area [mAU*s]	Height [mAU]	Area %
1	12.710	BB	0.2935	676.31561	34.08321	48.5096
2	15.328	BB	0.3849	717.87396	28.02118	51.4904

HPLC Conditions: Chiral IB; Hex/IPA 99:1, 0.5 ml/min; 23 °C

Figure S11. From (+)-(*P*) dioxa **1** to "(+)-(*P*)" azaoxa **2a** using optimized conditions

Using 1 equiv of primary amine and 2 equiv of DIPEA

Procedure : To a solution of (+)-(*P*)- or (–)-(*M*)-dioxa [6]helicene **1** (23 mg, 0.05 mmol) and benzoic acid (9 mg, 1.5 equiv) in NMP (0.5 mL) at 60 °C was a solution of *n*-propylamine (4 µL, 1 equiv) and ethyl di-*iso*-propyl amine (17 µL, 2 equiv) in NMP (0.5 mL) over a period of 24 hours. After this time, the reaction mixture was cooled to 20 °C and Et_2O (*ca.* 10 mL) was added leading to the precipitation of the crude material. The resulting solid was dissolved in CH_2Cl_2 (*ca.* 5 mL) and washed with aqueous 1M HBF_4 solution (3 x 10 mL). The organic layer was dried over Na_2SO_4, filtrated and evaporated under reduced pressure. The solid obtained was dissolved in CH_2Cl_2 (*ca.* 1 mL) and precipitated by addition of Et_2O (*ca.* 10 mL). The precipitate was separated from the mother liquor by centrifugation. The enantiomeric purity of azaoxa **2a** was analyzed by CSP-HPLC under cationic form. The crude reaction mixture was next purified by flash chromatography (CombiFlash, SiO_2 4 g cartridge, CH_2Cl_2/MeOH, 100:0 to 95:5 over 30 min) yielding the corresponding **2a** as a pink powder (3 mg, 6%). The enantiomeric purity of the pure product was verified by CSP-HPLC, no erosion of the enantiomeric excess could be detected.

(–)-(*M*) dioxa **1** to (–)-(*M*) azaoxa **2a**

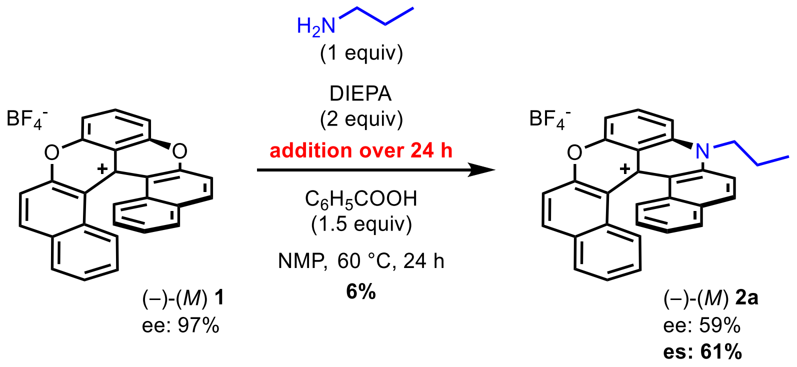

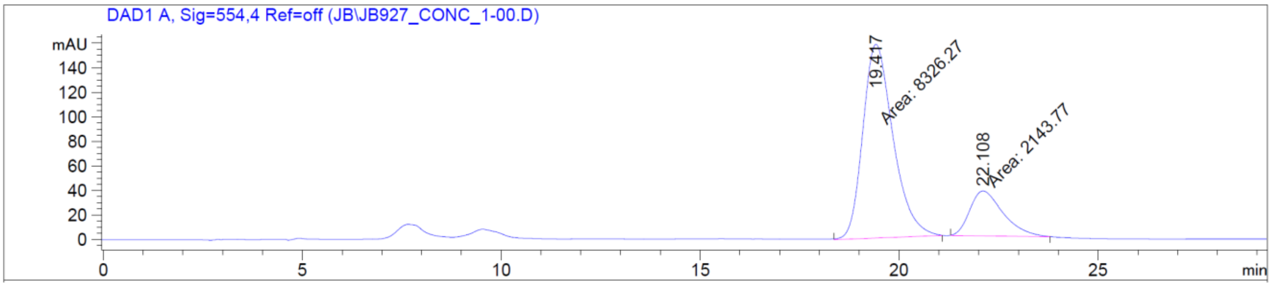

Peak #	RetTime [min]	Type	Width [min]	Area [mAU*s]	Height [mAU]	Area %
1	19.417	MM	0.8799	8326.27148	157.71902	79.5247
2	22.108	MM	0.9758	2143.76709	36.61384	20.4753

HPLC Conditions: LARIHC Hexane/EtOH 60:40 (TFA 0.6-TEA 0.4), 1 mL/min, 50 µL/inj

Figure S12. From (–)-(*M*) dioxa **1** to (–)-(*M*) azaoxa **2a** using modified conditions.

Enantiospecificity: 61%

(+)-(*P*) dioxa **1** to (+)-(*P*) azaoxa **2a**

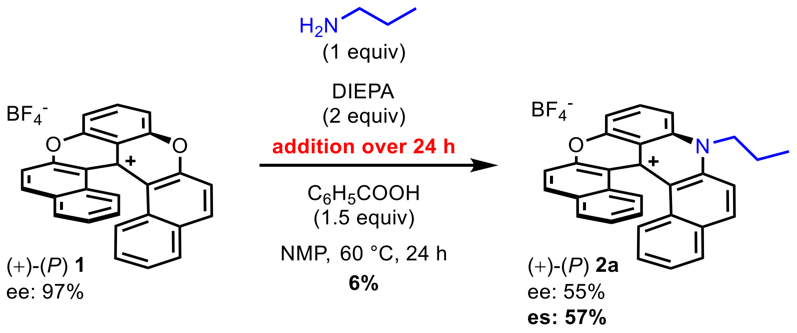

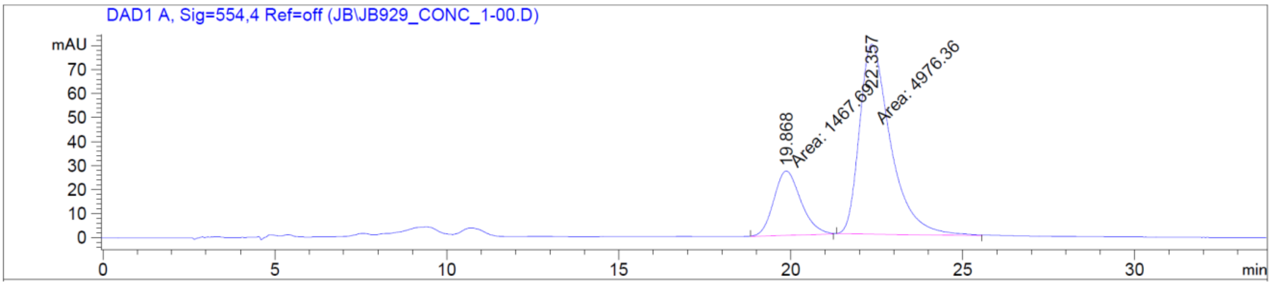

Peak #	RetTime [min]	Type	Width [min]	Area [mAU*s]	Height [mAU]	Area %
1	19.868	MM	0.9125	1467.69299	26.80614	22.7759
2	22.357	MM	1.0486	4976.35791	79.09190	77.2241

HPLC Conditions: LARIHC Hexane/EtOH 60:40 (TFA 0.6-TEA 0.4), 1 mL/min, 50 µL/inj

Figure S13. From (+)-(*P*) dioxa **1** to (+)-(*P*) azaoxa **2a** using modified conditions.

Enantiospecificity: 57%

From dioxa **1** to diaza **3a**

Procedure:

1) To a solution of (+)-(*P*)- or (–)-(*M*)-dioxa [6]helicene **1** (23 mg, 0.05 mmol) in NMP (0.5 mL) were added benzoic acid (76 mg, 12.5 equiv) *n*-propyl amine (103 µL, 25 equiv). The mixture was stirred at 70 °C for 7 hours. The reaction mixture was then cooled to 20 °C. Et_2O (*ca*. 10 mL) was added leading to the precipitation of the crude material. The resulting solid was dissolved in CH_2Cl_2 (*ca.* 5 mL) and washed with aqueous 1M HBF_4 solution (3 x 10 mL). The organic layer was dried over Na_2SO_4, filtrated and evaporated under reduced pressure. The solid obtained was dissolved in CH_2Cl_2 (*ca.* 1 mL) and precipitated by addition of Et_2O (*ca.* 10 mL). The precipitate was separated from the mother liquor by centrifugation.

2) A sample of the precipitated (ca. 5 mg) was dissolved in CH_3OH (1 mL) in a tinted flask in the absence of light and under N_2 atmosphere. Excess of $NaBH_4$ was added to the mixture, inducing a slow discoloration of the reaction mixture. The reaction mixture was stirred for 1 hour at 20 °C and then evaporated to dryness. The residue was dissolved in Et_2O (ca. 2 mL) and filtered through a short plug of Celite. The filtrate was evaporated and subjected to CSP-HPLC analysis.

(–)-(*M*) dioxa **1** to (–)-(*M*) diaza 3**a**

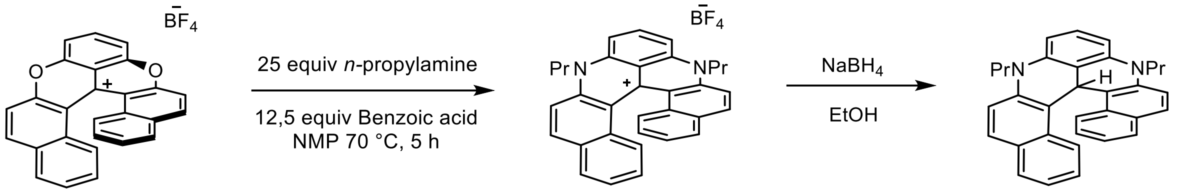

(–)-(*M*) **1**
98 % ee

3a
0 % ee

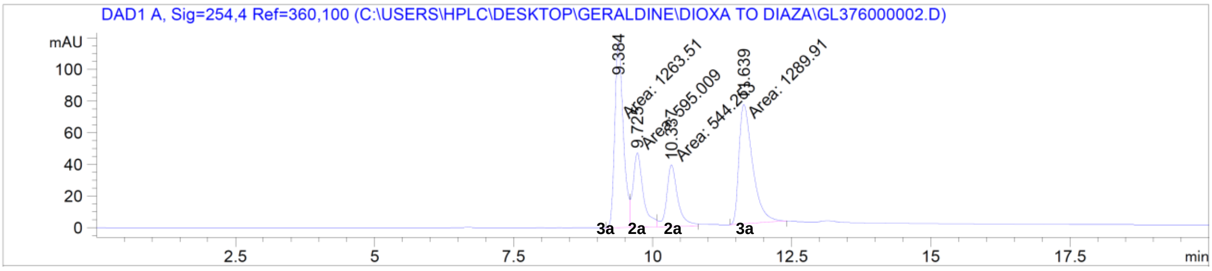

Peak #	RetTime [min]	Type	Width [min]	Area [mAU*s]	Height [mAU]	Area %	
1	9.384	MF	0.1795	1263.50562	117.33878	34.2165	diaza **3a**
2	9.725	MF	0.2110	595.00922	47.00842	16.1132	azaoxa **2a**
3	10.337	FM	0.2324	544.25275	39.03568	14.7387	azaoxa **2a**
4	11.639	MM	0.2849	1289.90759	75.44932	34.9315	diaza **3a**

HPLC Conditions: Chiral IB; Hex/IPA 99:1, 0.5 ml/min; 23 °C

Figure S14. From (–)-(*M*) dioxa **1** to "(–)-(*M*)" diaza **3a**

(+)-(*P*) dioxa **1** to (+)-(*P*) diaza 3**a**

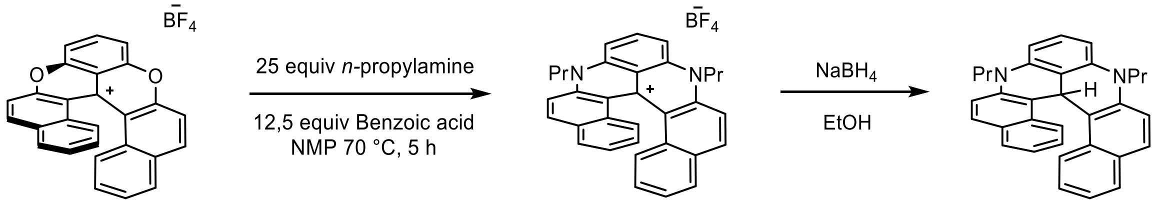

(+)-(*P*) **1**
99 % ee

(+)-(*P*) **3a**
4 % ee

4 % ee

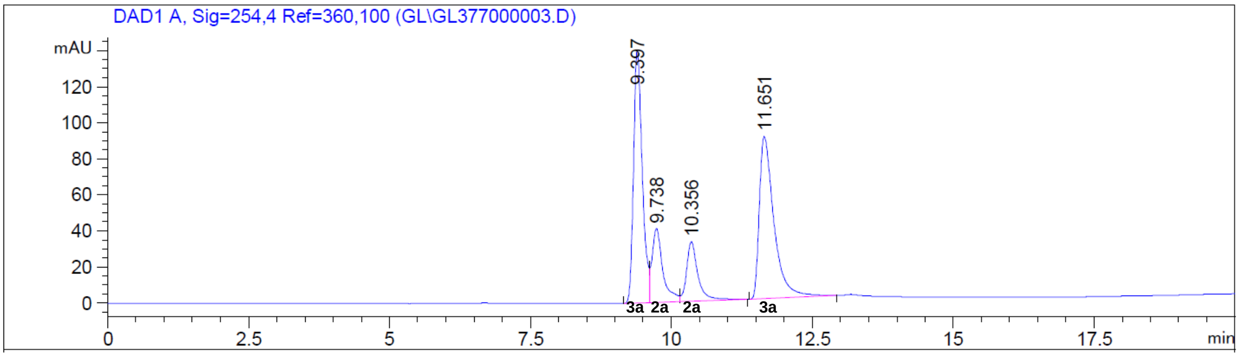

Peak #	RetTime [min]	Type	Width [min]	Area [mAU*s]	Height [mAU]	Area %	
1	9.397	BV	0.1633	1505.82104	140.19548	36.3238	diaza **3a**
2	9.738	VV	0.1935	546.98242	41.03147	13.1945	azaoxa **2a**
3	10.356	VB	0.2074	467.67468	32.98400	11.2814	azaoxa **2a**
4	11.651	BB	0.2688	1625.06653	89.93502	39.2003	diaza **3a**

HPLC Conditions: Chiral IB; Hex/IPA 95:5, 0.5 ml/min; 23 °C

Figure S15. From (+)-(*P*) dioxa **1** to “(+)-(*P*)” diaza **3a**

<u>From azaoxa **2** to diaza **3**</u>

Procedure:

1) To a solution of (+)-(*P*)- or (–)-(*M*)-azaoxa [6]helicene **2a** (25 mg, 0.05 mmol) in NMP (0.5 mL) were added benzoic acid (76 mg, 12.5 equiv) *n*-propyl amine (103 µL, 25 equiv). The mixture was stirred at 70 °C for 7 hours. The reaction mixture was then cooled to 20 °C. Et_2O (*ca*. 10 mL) was added leading to the precipitation of the crude material. The resulting solid was dissolved in CH_2Cl_2 (*ca.* 5 mL) and washed with aqueous 1M HBF_4 solution (3 x 10 mL). The organic layer was dried over Na_2SO_4, filtrated and evaporated under reduced pressure. The solid obtained was dissolved in CH_2Cl_2 (*ca.* 1 mL) and precipitated by addition of Et_2O (*ca.* 10 mL). The precipitate was separated from the mother liquor by centrifugation.

2) A sample of the precipitated (ca. 5 mg) was dissolved in CH_3OH (1 mL) in a tinted flask in the absence of light and under N_2 atmosphere. Excess of $NaBH_4$ was added to the mixture, inducing a slow discoloration of the reaction mixture. The reaction mixture was stirred for 1 hour at 20 °C and then evaporated to dryness. The residue was dissolved in Et_2O (ca. 2 mL) and filtered through a short plug of Celite. The filtrate was evaporated and subjected to CSP-HPLC analysis.

(–)-(*M*) azaoxa **2a** to (–)-(*M*) diaza 3**a**

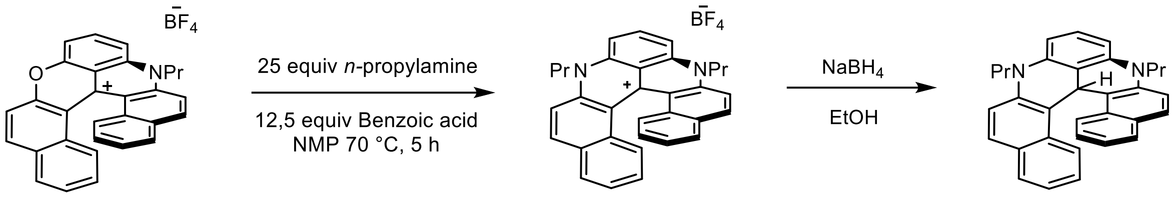

(–)-(*M*) **2a**
98 % ee

(–)-(*M*) **3a**
93 % ee

93 % ee

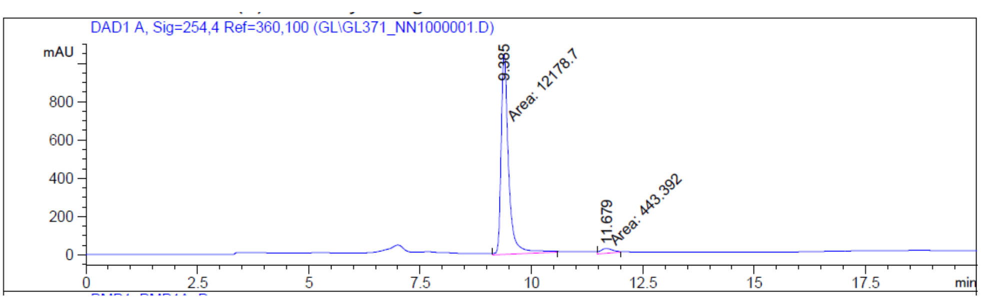

Peak #	RetTime [min]	Type	Width [min]	Area [mAU*s]	Height [mAU]	Area %
1	9.385	MM	0.1940	1.21787e4	1046.18945	96.4872
2	11.679	MM	0.2994	443.39166	24.68487	3.5128

HPLC Conditions: Chiral IB; Hex/IPA 95:5, 0.5 ml/min; 23 °C

Figure S16. From (–)-(*M*) azaoxa **2a** to (–)-(*M*) diaza **3a**

Enantiospecificity: 95%

(+)-(*P*) azaoxa **2a** to (+)-(*P*) diaza 3**a**

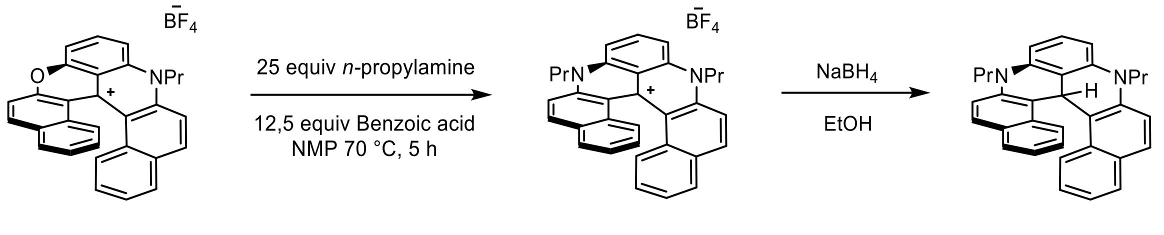

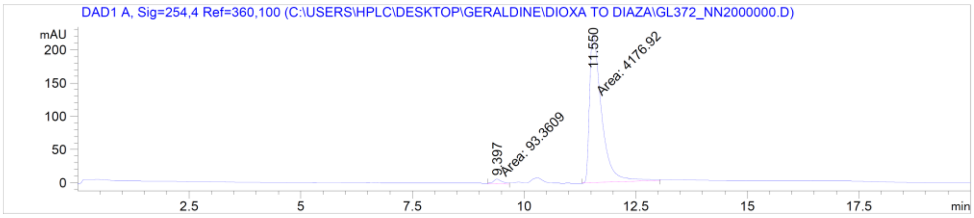

Peak #	RetTime [min]	Type	Width [min]	Area [mAU*s]	Height [mAU]	Area %
1	9.397	MM	0.2269	93.36093	6.85665	2.1863
2	11.550	MM	0.3129	4176.91699	222.49338	97.8137

HPLC Conditions: Chiral IB; Hex/IPA 95:5, 0.5 ml/min; 23 °C

Figure S17. From (+)-(*P*) azaoxa **2a** to (+)-(*P*) diaza **3a**

Enantiospecificity: 97%

6. Attribution of the absolute configuration of (+)- and (–)-1 and 2a

The attribution of the absolute configuration of the (+) and (–) enantiomers of dioxa [6]helicene **1** and the azaoxa [6]helicene **2a** was done using vibrational circular dichroism (VCD) technics. In short, the theoretical VCD spectra of both the (*M*) and the (*P*) enantiomers of each helicenes are calculated. Those spectra are then compared to the experimental VCD traces recorded for the (+) and (–) helicenes. In the present case and for both **1** and **2a**, a clear match is observed between the experimental spectra of the (+) enantiomers and the calculated VCD spectra of the of the (*P*) helicenes. The attribution of the absolute configuration of diaza [6]helicene **3a** has been reported previously: the (+) enantiomer is again of (*P*) helicity.[1]

Experiment

IR and vibrational circular dichroism (VCD) spectra were recorded on a Bruker PMA 50 accessory coupled to a Tensor 27 Fourier transform infrared spectrometer. A photoelastic modulator (Hinds PEM 90) set at l/4 retardation was used to modulate the handedness of the circular polarized light. Demodulation was performed by a lock-in amplifier (SR830 DSP). An optical low-pass filter (< 1800 cm^{-1}) in front of the photoelastic modulator was used to enhance the signal/noise ratio. Spectra were recorded with a transmission cell equipped with CaF_2 windows and a 0.2 mm spacer. Solutions were prepared in CD_2Cl_2 at concentrations of 4 mg in 180 µL. The pure solvent was used as the reference both for the IR and VCD measurements. Both sample and reference were measured at a resolution of 4 cm^{-1} by averaging about 6’000 scans in total for sample and reference, respectively. The reference VCD spectrum was subtracted from the sample spectrum. Spectra are presented without further data processing.

Calculations

The geometry optimizations, vibrational frequencies, IR absorption and VCD intensities were calculated with Density Functional Theory (DFT) using the B3PW91 functional and a 6-31G(d,p) basis set. Frequencies were scaled by a factor of 0.966. IR absorption and VCD spectra were constructed from calculated dipole and rotational strengths assuming Lorentzian band shape with a half-width at half maximum of 4 cm^{-1}. All calculations were performed using Gaussian09.

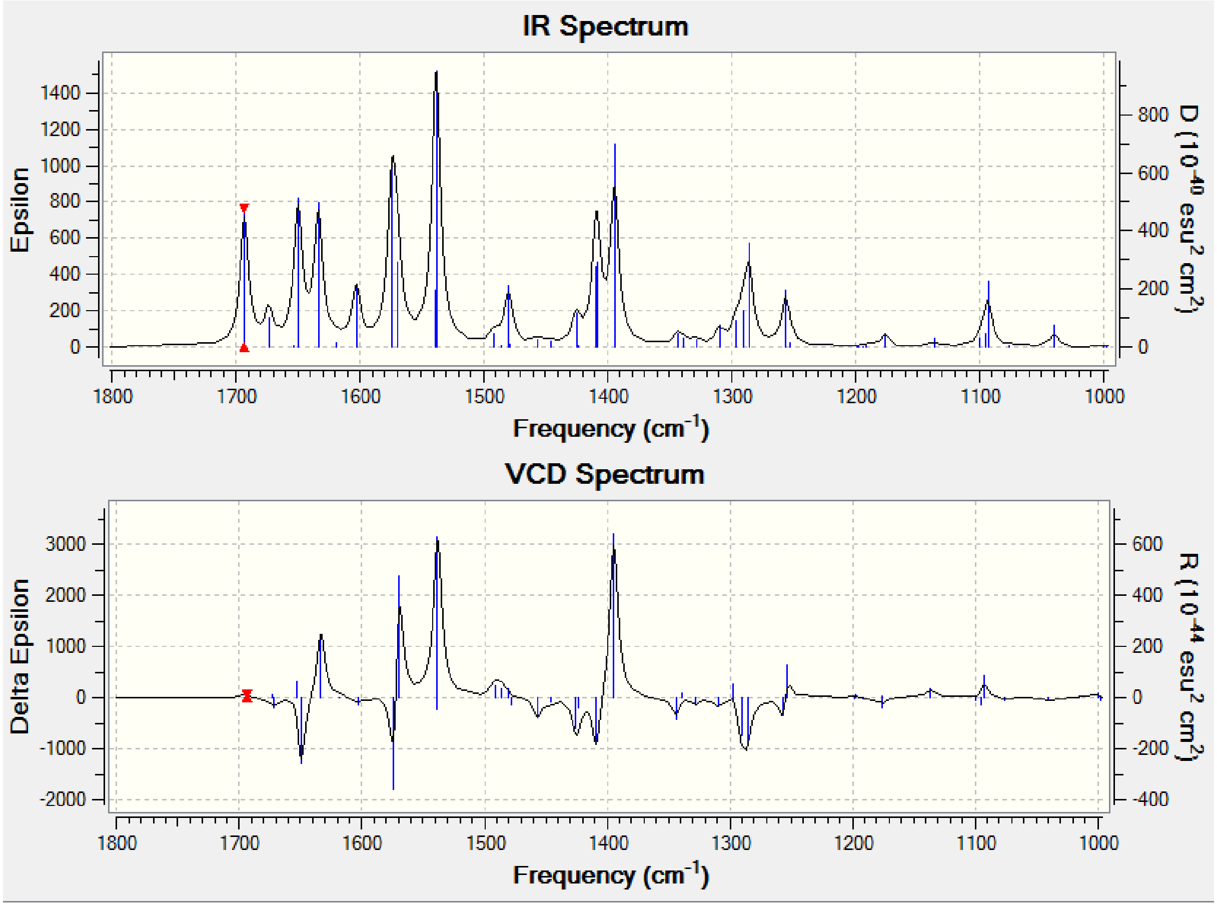

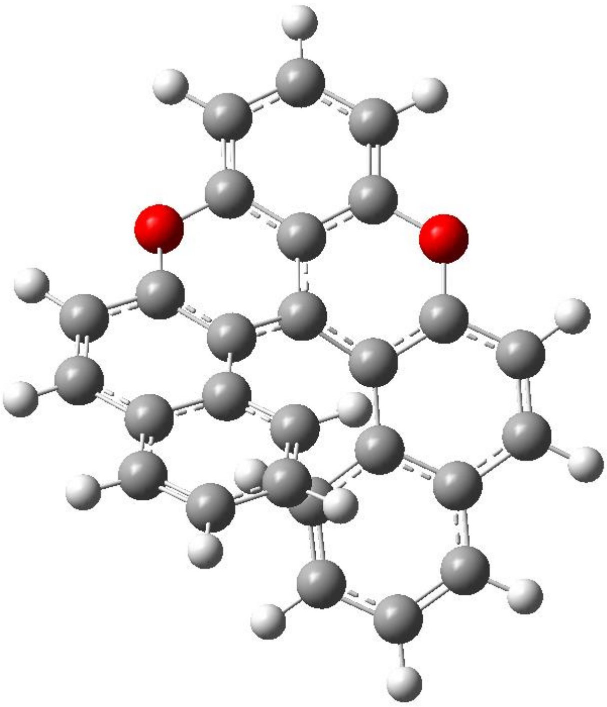

Figure S18. Calculated IR and VCD spectra of (*P*)-**1**

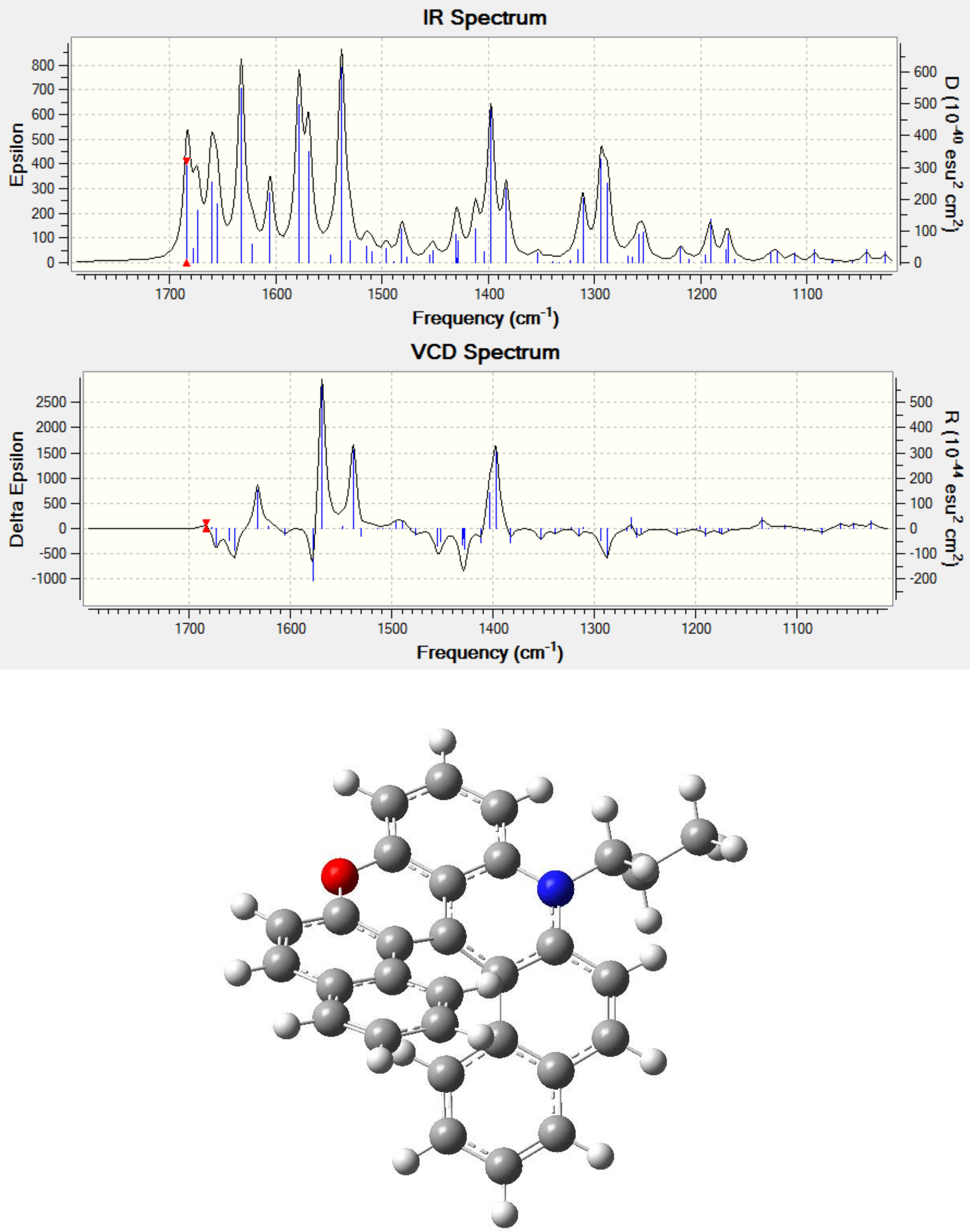

Figure S19. Calculated IR and VCD spectra of (*P*)-**2a**

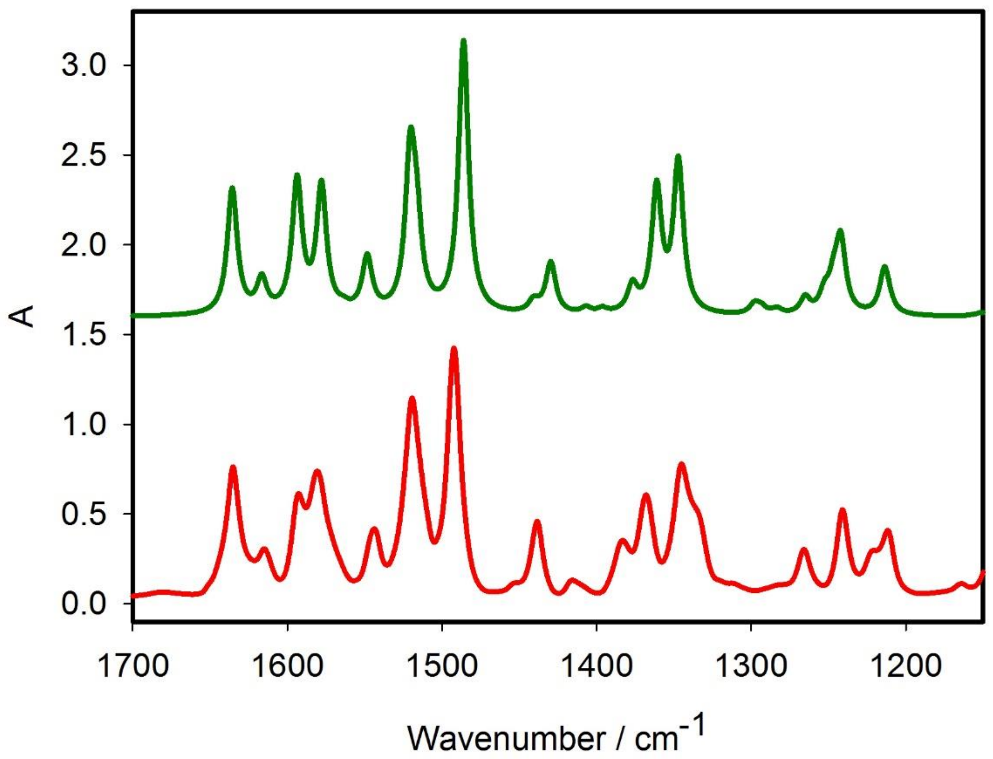

Figure S20. Comparison of the calculated (green) and experimental (red) IR spectra of **1**

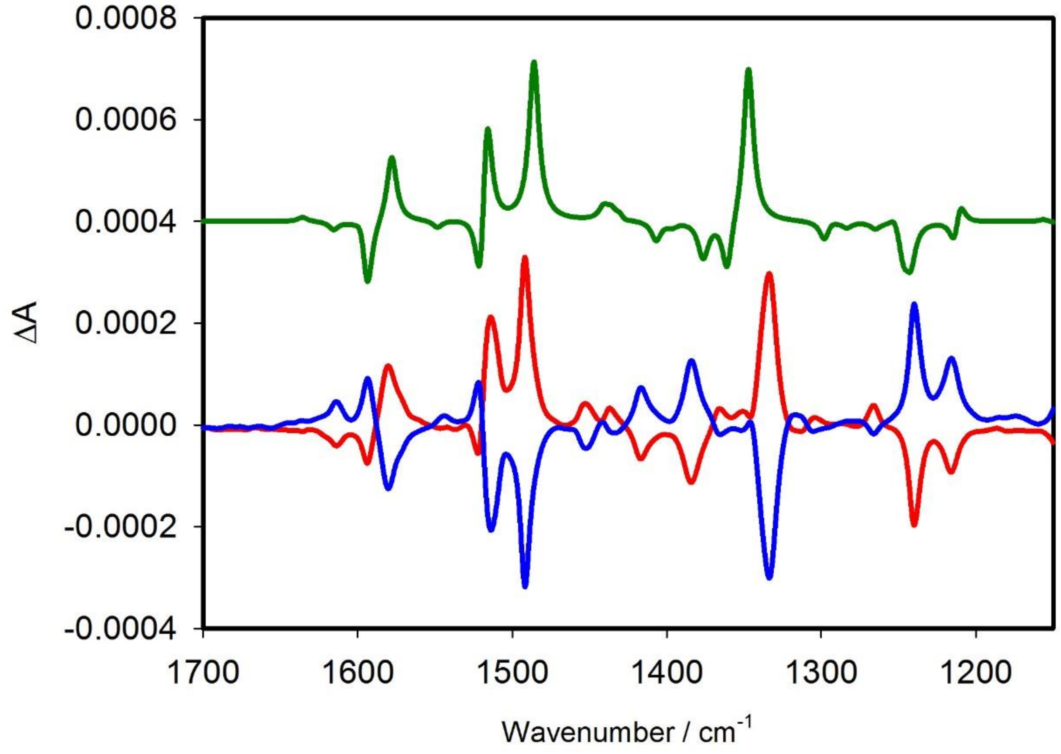

Figure S21. Comparison of the calculated VCD spectrum of (*P*)-**1** (green) and the experimental VCD spectra of (+)-**1** (red) and (–)-**1** (blue)

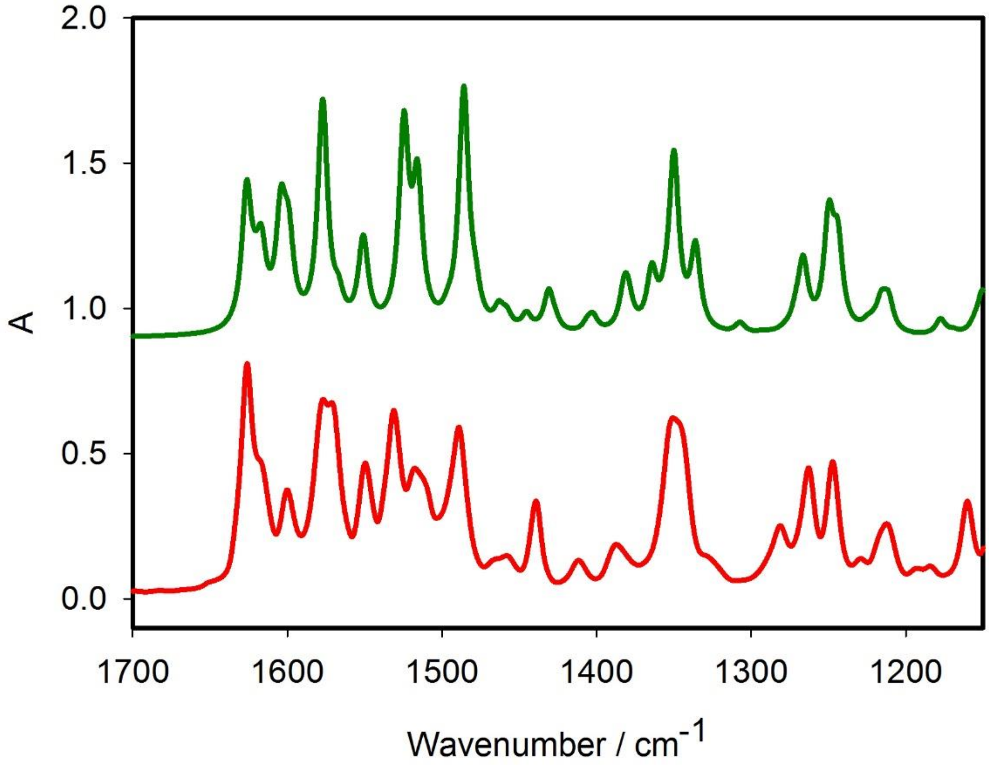

Figure S22. Comparison of the calculated (green) and experimental (red) IR spectra of **2a**

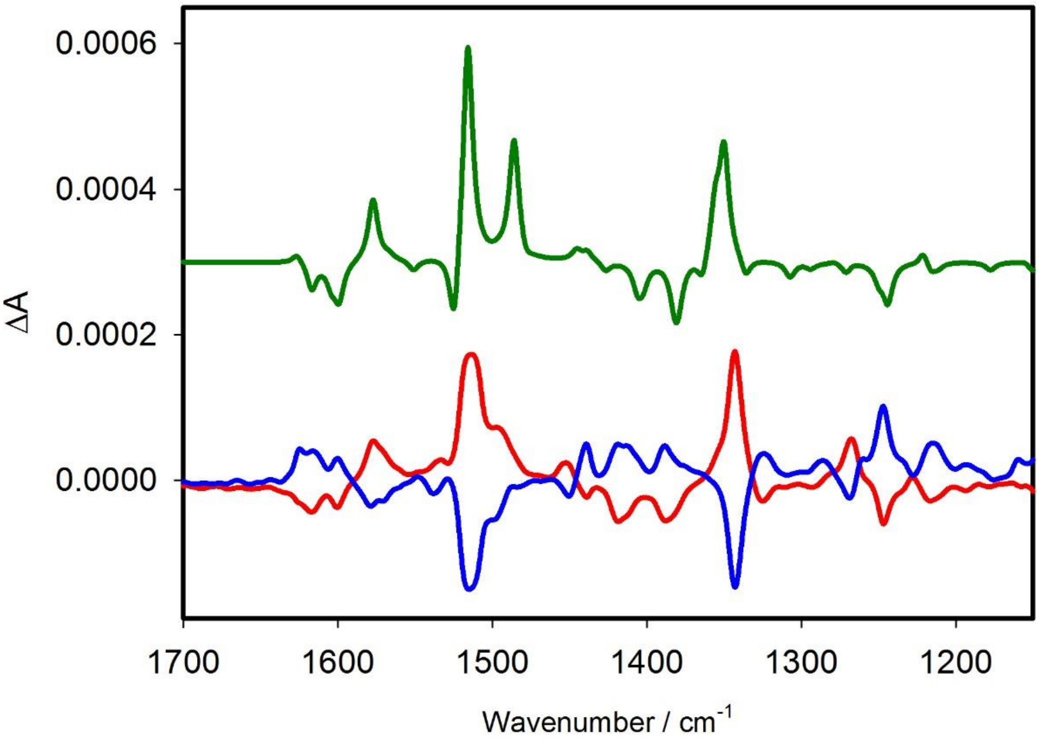

Figure S23. Comparison of the calculated VCD spectrum of (*P*)-**2a** (green) and the experimental VCD spectra of (+)-**2a** (red) and (–)-**2a** (blue)

7. Discussion about the theoretical rationalization of the mechanism

General remarks

All geometries were fully optimized in gas phase at the B3PLYP level[3, 4] of theory without any constraints at 298 K and 1 atm. The Berny algorithm was used for geometry optimizations.[5] 6-311G(d,p) Pople basis set was used for all atoms.[6, 7] Frequency calculations were performed at the same level for all intermediates and transition states to confirm minima (no imaginary frequencies) and first order saddle points (one imaginary frequency representing the desired reaction coordinate), respectively. Furthermore, transition states were further confirmed by either intrinsic reaction coordinates (IRC).[8-10] Single point energies were calculated on the optimized structures at 298 K and 1 atm at the M06L level of theory,[11] a method in which dispersion effects are included in the parameterization scheme. The Karlsruhe triple–ζ Gaussian basis set def2-TZVPP was used for all atoms.[12, 13] Solvents effects were assessed through the integral equation formalism variant of the polarizable continuum model (IEFPCM)[14] with default parameters for DMA and UFF radii, while NMP was defined by an eps = 32.17, density = 1.03 and epsinf = 2.1609. Relative energies are reported in kcal mol^{-1}. The following nomenclature will be used in the discussion: ΔE relative energy with respect to the correspondent reactant; ΔΔE difference between relative energies; $\Delta E^{\ddagger}$ activation energies; and $\Delta\Delta E^{\ddagger}$ difference between activation energies. The fully detailed potential energy surface profiles inclusive of all the intermediates not presented in the text are included in the Supplementary Information. All calculations were performed by using the *Gaussian09* package, revision D.01.[15]

From dioxa **1** to azaoxa **2**

Reaction occurring on the *Re* face of (*M*)-dioxa **1**

The reaction occurring on the *Re* face of (*M*)-dioxa **1** is discussed in the main text in Figure 7.

Reaction occurring on the *Si* face of (*M*)-dioxa **1**

The nucleophilic attack of the amine cluster on the *Si* face of (*M*)-dioxa **1** is less energy demanding (ΔE = +10.4 kcal mol^{-1}) and the resulting ^{Si}**B** amino adduct is at least 6 kcal mol^{-1} more stable than **B** (Figure S24). Unfortunately, any efforts to locate the following O-ring opening were unsuccessful. The optimization of the ^{Si}**A** intermediate is now less stable than the starting material by +5.7 kcal mol^{-1}. Interestingly, the analysis of the structure indicates that the positive charge is now carried by the iminium group and not by the ammonium as it was computed in the *Re* face attack. In addition, a strong deformation around the C-C bond linking the two aromatic units highly destabilizes this geometry. The overall reaction path model an easier formation of the amino adduct although the comparable ^{Si}**A** intermediate is disfavored by almost 12 kcal mol^{-1}, favoring the nucleophilic attack occurring by the *Re* face.

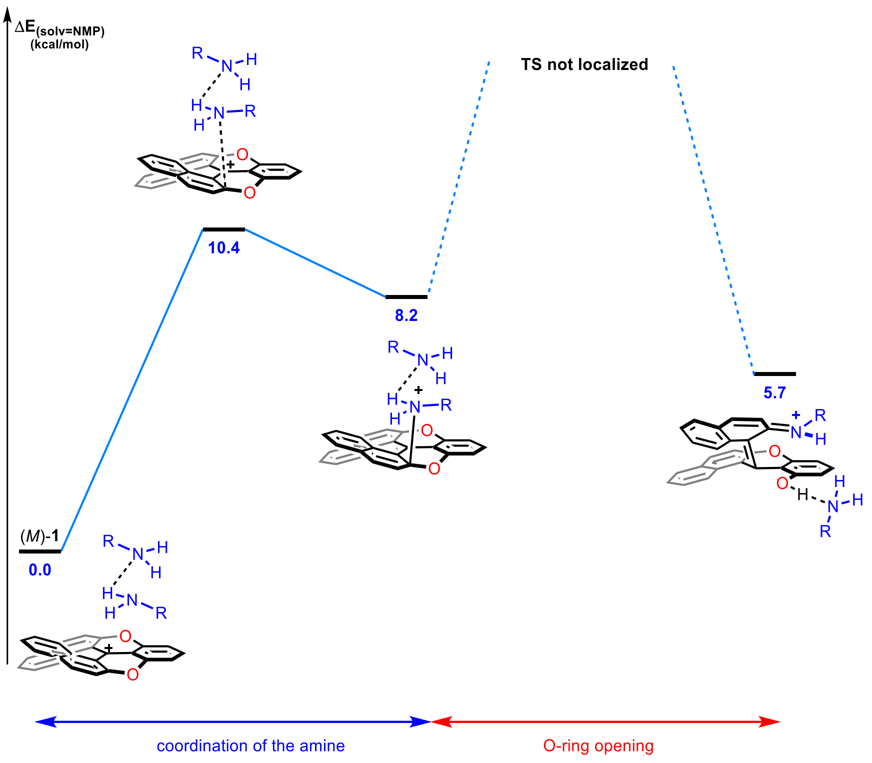

Figure S24. Reaction energy profile for the formation of the open ^{Si}**A** intermediate by the amine attack on the *Si* face of (*M*)-dioxa **1**. Solvation Energies (solv = NMP) in kcal mol^{-1}.

From (*P*)-dioxa **1** to (*P*)-azaoxa **2**

The reaction path for the **1→2** transformation was also calculated in the (*P*) series (Figure S25). According to the results in the (*M*) series, the calculation of the reaction pathway on the (*P*) enantiomer was carried out only from the *Si* face. The process follows a similar behavior, starting by the opening of the helicene to from intermediate (*aS*)-**A**, followed by an ammonium – acid exchange and finishing by the ring closure to form the (*P*)-azaoxa helicene. The formation of the new C-N bond ($\Delta\Delta E^{\ddagger}$ = +27.6 kcal mol^{-1}) is still the rate limiting step and the overall process is exothermic by 12.2 kcal mol^{-1}. As before, the final dehydration step is barrierless although in this case, no transition state has been located due to the flatness of the potential energy surface. The calculation of this transformation confirms the stereospecificity of the process and the retention of the configuration of the helicene.

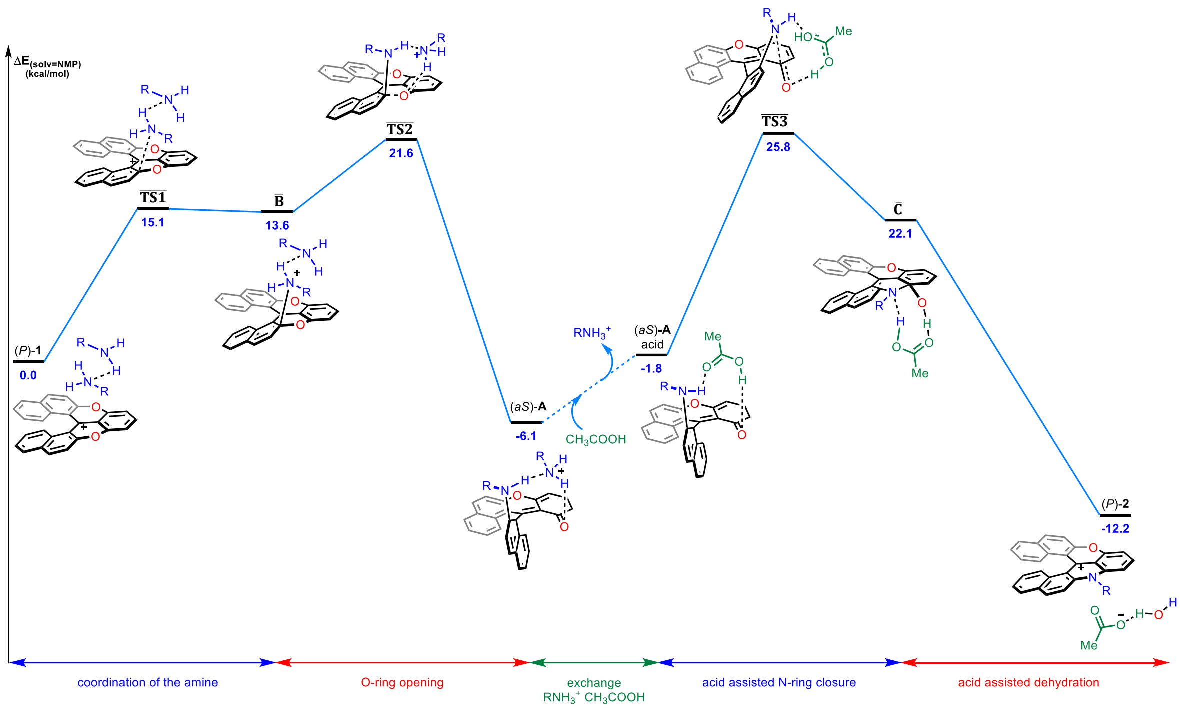

Figure S25. Reaction energy profile the formation of (*P*)-azaoxa **2** by the amine attack on the *Si* face of the (*P*)-dioxa **1** helicene via intermediate (*aS*)-**A**. Solvation Energies (solv = NMP) in kcal mol^{-1}.

Access to three-bladed propellers D and E.

The relative energy of the protonated form **A·H$^+$** is directly related to the stabilization brought by the amine cluster that as nucleophile.

Attack from the *Si* face leading to propeller (Δ)-E

The coordination of the amine cluster that leads to the formation of (*aR*)-**A·H$^+$** amine cluster is destabilized by 1.5 kcal mol^{-1} (Figure S26). The following nucleophilic attack occurs through a transition state located at +7.1 kcal mol^{-1} leading to the corresponding adduct localized at +3.4 kcal mol^{-1}. The analysis of the structure model the positive charge on the ammonium group directly attached to a sp^3 carbon. This carbon model an unusual trigonal planar geometry similar to a sp^2 hybridization although the bond lengths are similar to sp^3 hybridized atom. The following opening of the O-ring occurs through a high transition state (ΔΔE‡ = +30.8 kcal mol^{-1}) and leads to the formation of propeller (Δ)-**E** located at -8.1 kcal mol^{-1}.

The structural analysis of this system shows that the central carbon atom of (Δ)-**E** is sp^2 hybridized. Both OH groups are similar and consistent with phenol groups in terms of bond lengths and angles. The final (Δ)-**E** structure is thus cationic and stabilized through H-bonding by the extra amine. The two naphthyl groups exhibit different orientations relative to the central phenyl unit: one amino group is directed toward the center and the other outward.

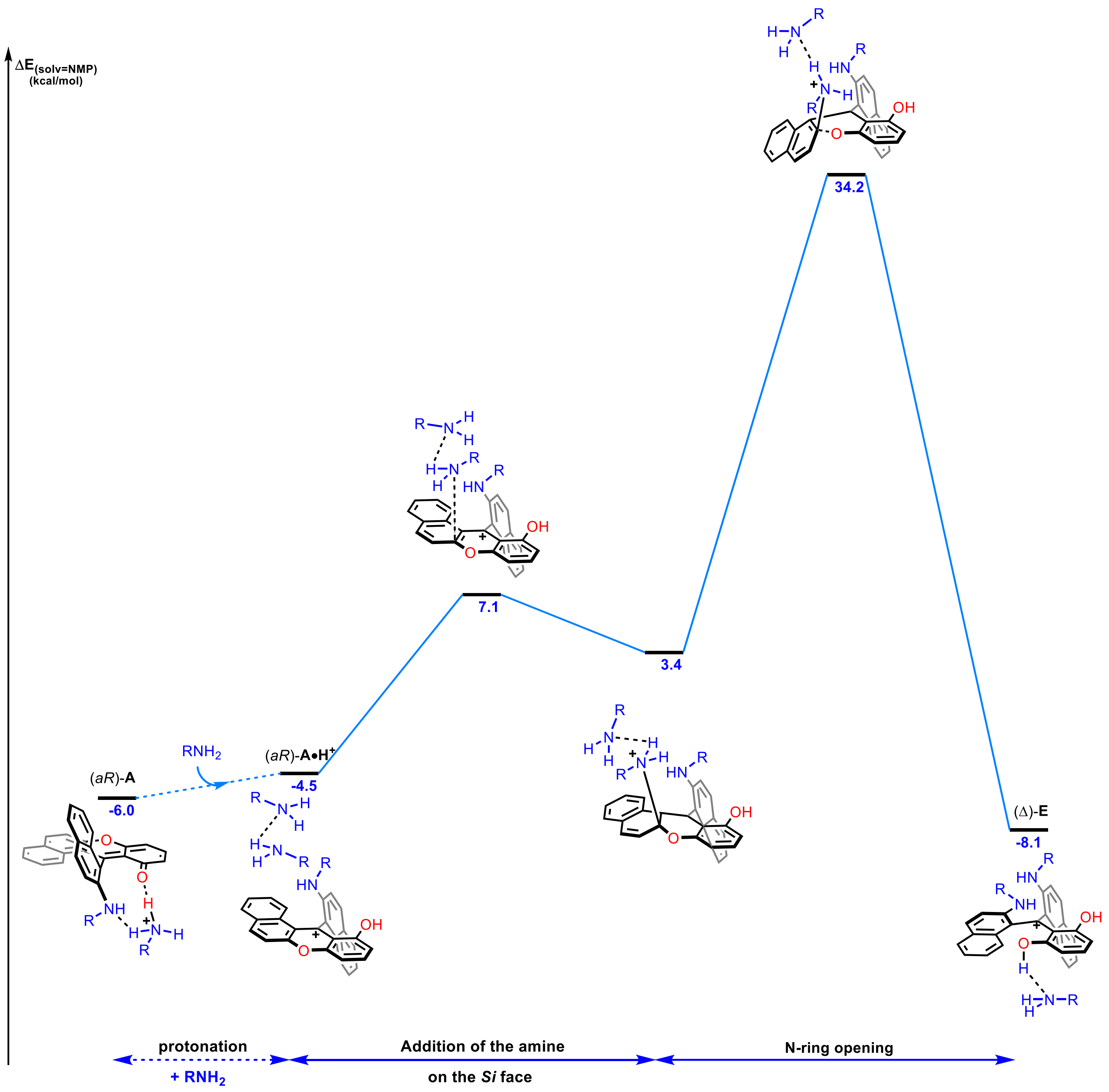

Figure S26. Reaction energy profile for the ring opening of the cationic (aR)-**A·H**$^+$ by the amine attack by the *Si* face to form the propeller (Δ)-**E**. Solvation Energies (solv = NMP) in kcal mol^{-1}.

<u>Attack from the *Re* face leading to propeller (Λ)-**D**</u>

The approach of the amine cluster by the *Re* face to form **A·H**$^+$ destabilized the system by 5.4 kcal mol^{-1} (Figure S27). This result shows that the H-bonding stabilization brought by the amines to intermediate **A·H**$^+$ on the *Si* face is as large as 3.8 kcal mol^{-1}. The formation of the new C-N bond occurs throughout an activation barrier of +7.0 kcal mol^{-1} providing the corresponding adduct at +3.0 kcal mol^{-1}. As detailed above, the structure models the positive charge back to the ammonium moiety. The transition state of the subsequent O-ring opening lies at +29.3 kcal mol^{-1} and provides the cationic (Λ)-**D** at -4.3 kcal mol^{-1}.

Structural analysis confirmed the cationic sp^2 character of the central carbon atom and the phenol character of the two Ar-OH groups. Moreover, the two naphthyl groups are oriented in the same direction with their amino groups pointing toward the phenyl ring. Interestingly, when coordinated to the extra amine by H-bond, (Δ)-**E** is more stable than diastereomeric (Λ)-**D** by 3.8 kcal mol^{-1}.

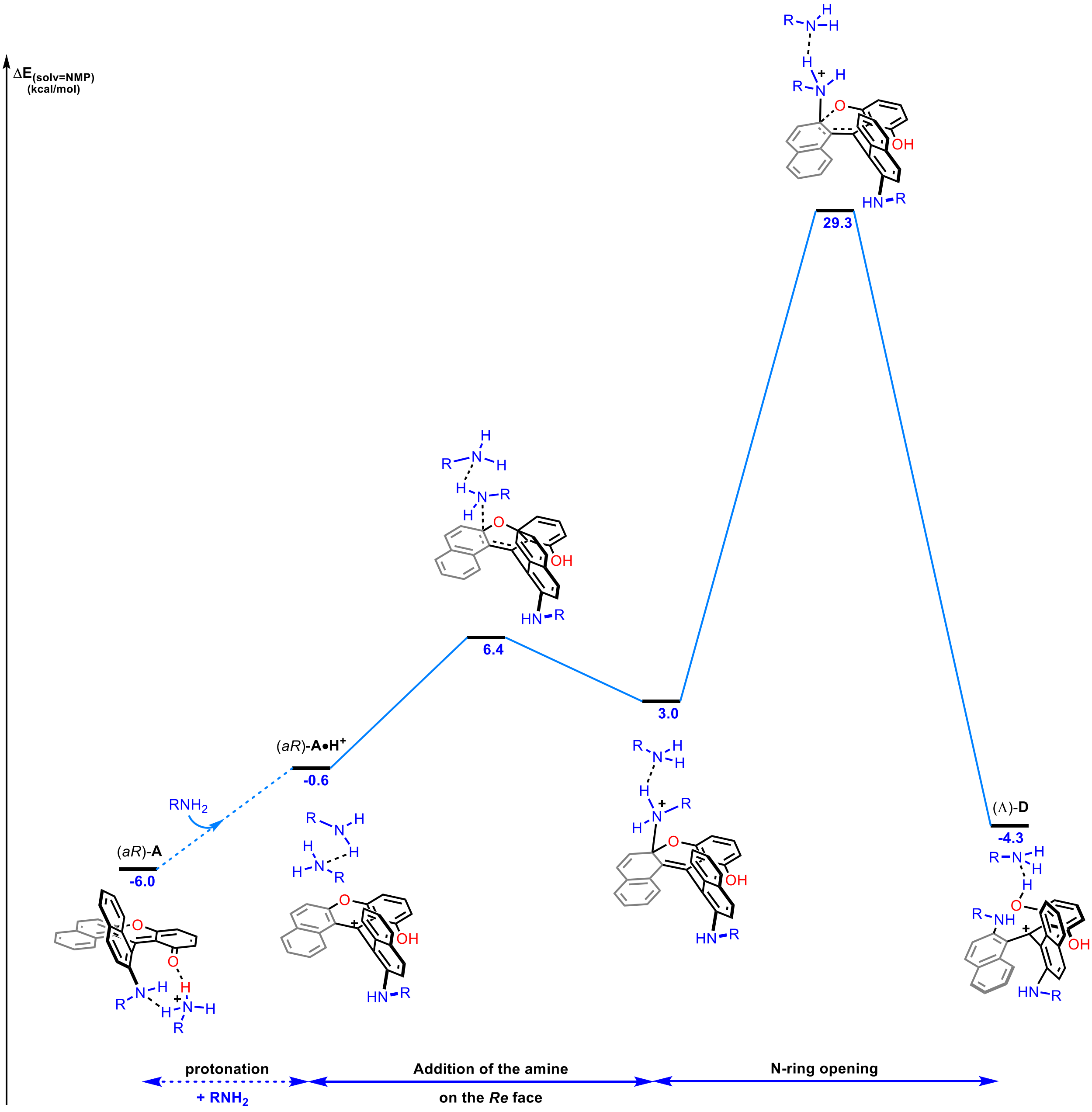

Figure S27. Reaction energy profile for the O-ring opening of cationic (*aR*)-**A·H**+ by the amine attack on the *Re* to form the propeller (Λ)-**D**. Solvation Energies (solv = NMP) in kcal mol^{-1}.

Influence of the extra amine on the relative energies of the isomerizations (Λ)-D → (Δ)-D, (Δ)-E → (Λ)-E and (Λ)-D → (Δ)-E

In order to highlight the detrimental role of the excess of primary amine in the lack of enantiospecificity of the transformation from **1** to **2**, the three isomerization mechanisms depicted above (*ie* the enantiomerization from (Δ)-**E** to (Λ)-**E** and from (Λ)-**D** to (Δ)-**D** and the isomerization of both propellers **E** and **D**), have been also investigated in the presence and in the absence of an extra amine (Figures S28 – S30). In all the cases, the extra amine models an H-bonding with one of the alcohols of the bis-phenol moiety. Even if this interaction stabilizes the species, it does not have a direct influence on the

geometry of the propellers in the ground or excited states. In a general manner, the extra amine tends to lower the isomerization barriers.

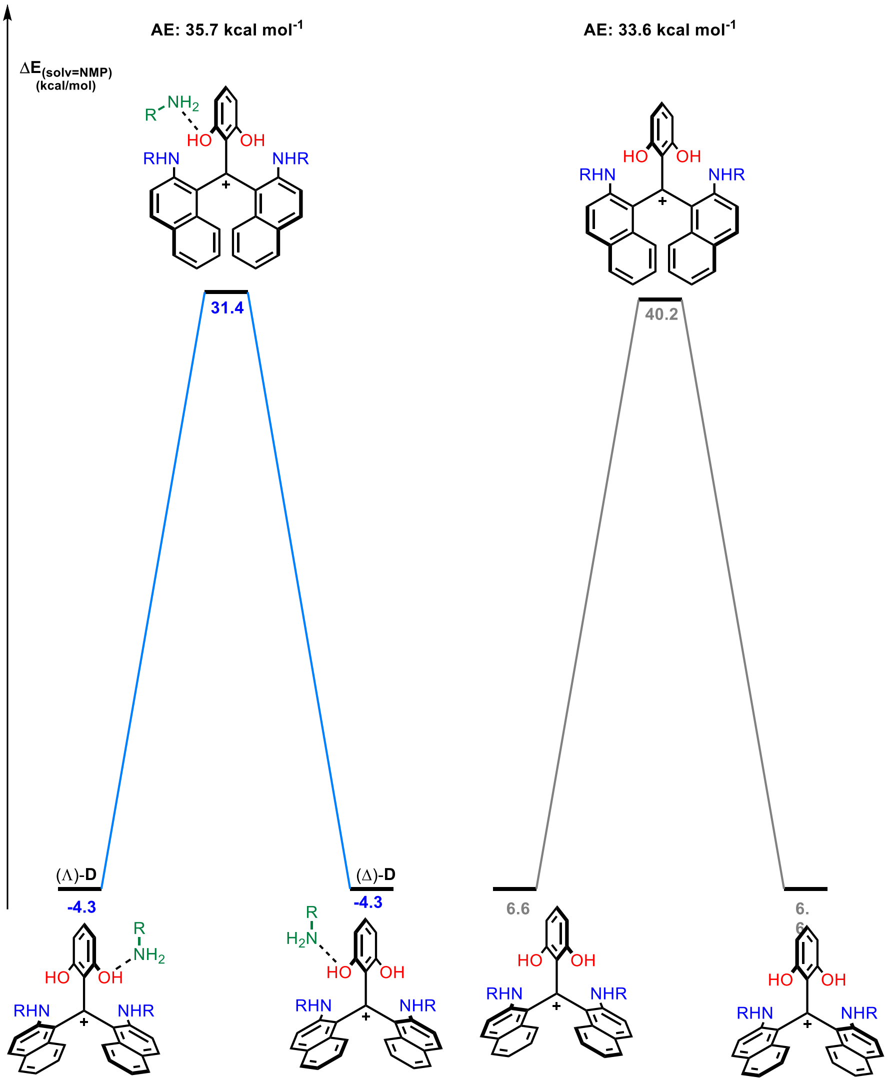

Figure S28. Comparison energy reaction profiles for the isomerization from (Λ)-**D** to (Δ)-**D** in the presence and in the absence of an extra amine. Solvation Energies (solv = NMP) in kcal mol^{-1}.

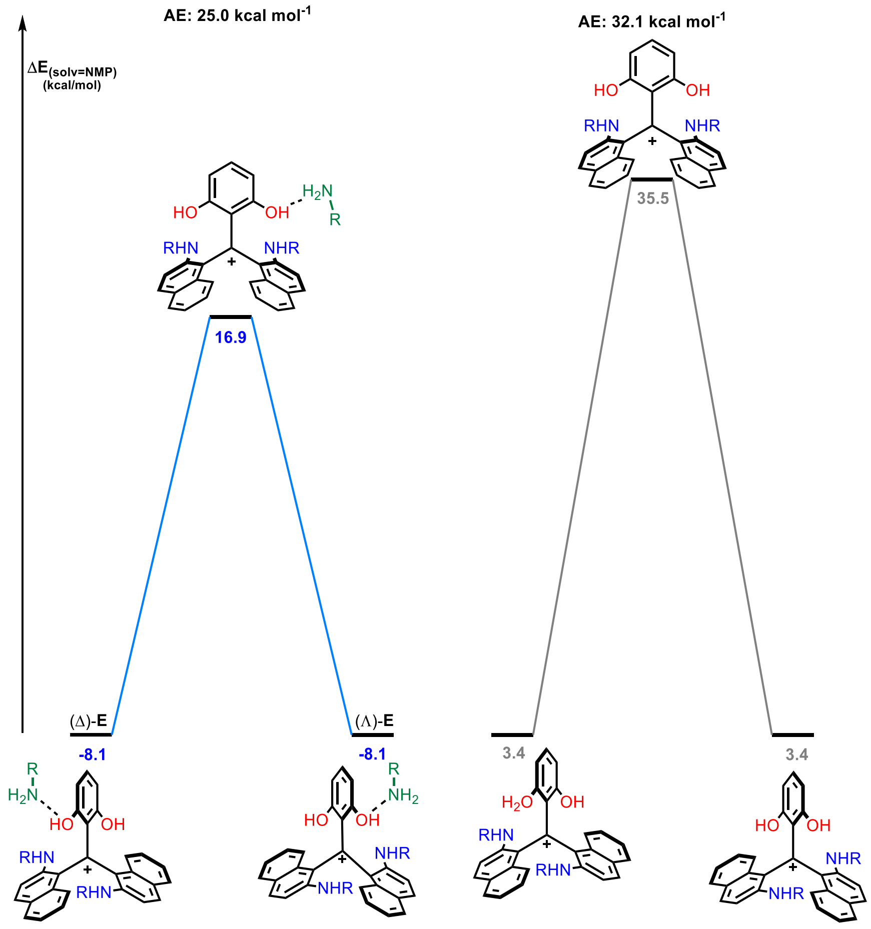

Figure S29. Comparison energy reaction profiles for the isomerization from (Δ)-**E** to (Λ)-**E** in the presence and in the absence of an extra amine. Solvation Energies (solv = NMP) in kcal mol^{-1}.

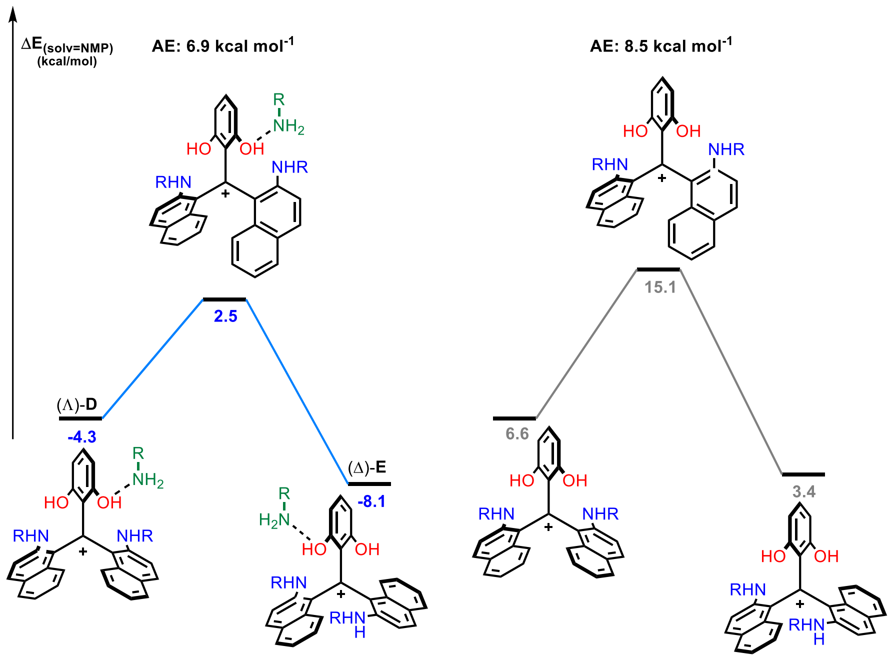

Figure S30. Comparison energy reaction profiles for the isomerization from (Λ)-**D** to (Δ)-**E** in the presence and in the absence of an extra amine. Solvation Energies (solv = NMP) in kcal mol^{-1}.

O-ring closure of propeller (Δ)-**D** or (Λ)-**E** leading to cationic (*aS*)-**A·H**$^{+}$

The reaction profile back to biaryl (*aS*)-**A·H**$^{+}$ intermediate was studied with the two enantiomerized propellers (Δ)-**D** or (Λ)-**E**. Not surprisingly, the reactions follow mirror pathways than those computed for propellers (Λ)-**D** and (Δ)-**E**. Therefore, both paths will not be systematically discussed. Nevertheless, the comparison of both mechanism highlight:

a) the transition state of the O-ring closure is slightly lower in energy than that of the TS located for the O-ring opening (32.5 *vs* 34.3 kcal mol^{-1}, respectively, Figure S28), this can be attributed to a conformational difference in the TS
b) (*aS*)-**A·H**$^{+}$ is less stable than (*aR*)-**A·H**$^{+}$ (-3.1 *vs* -4.5 kcal mol^{-1}, respectively, Figure S28) due to a conformational change in the stabilizing extra amine
c) (*aS*)-**A** and (*aR*)-**A** are localized at the same energy level (-6.0 kcal mol^{-1}).

O-ring closure of propeller (Δ)-**D** leading to (*aS*)-**A·H**$^{+}$

The O-ring closure of (Δ)-**D** to (*aS*)-**A·H**$^{+}$ is depicted in Figure S31.

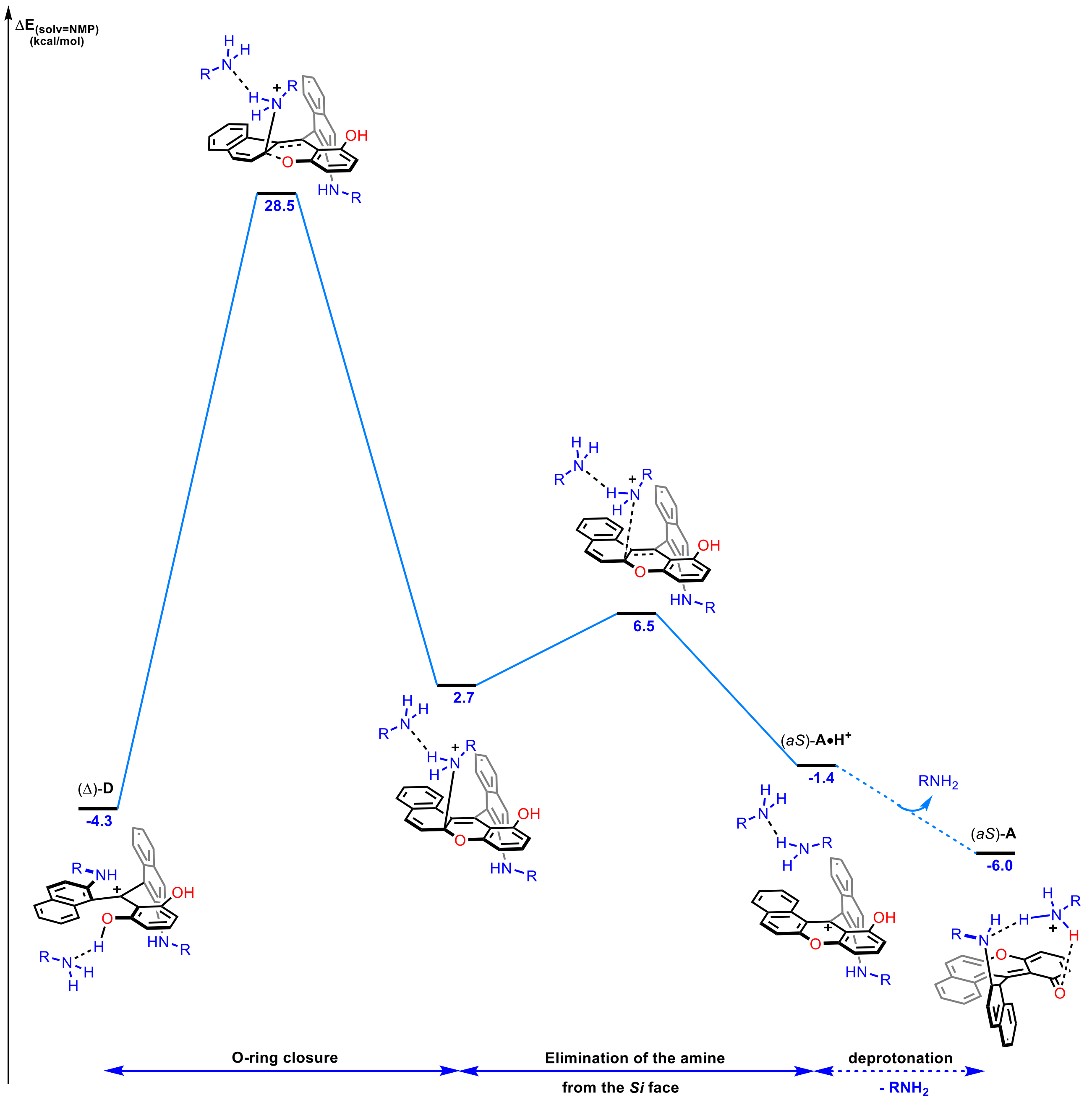

Figure S31. Reaction energy profile for the O-ring closure of (Δ)-**D** to (*aS*)-**A·H**$^+$. Solvation Energies (solv = NMP) in kcal mol^{-1}.

<u>O-ring closure of propeller (Λ)-**E** leading to (*aS*)-**A·H**$^+$</u>

The O-ring closure of (Λ)-**E** to (*aS*)-**A·H**$^+$ is depicted in Figure S32. The reaction profile is nearly mirror image of the O-ring opening leading to (Δ)-**E** (Figure S26).

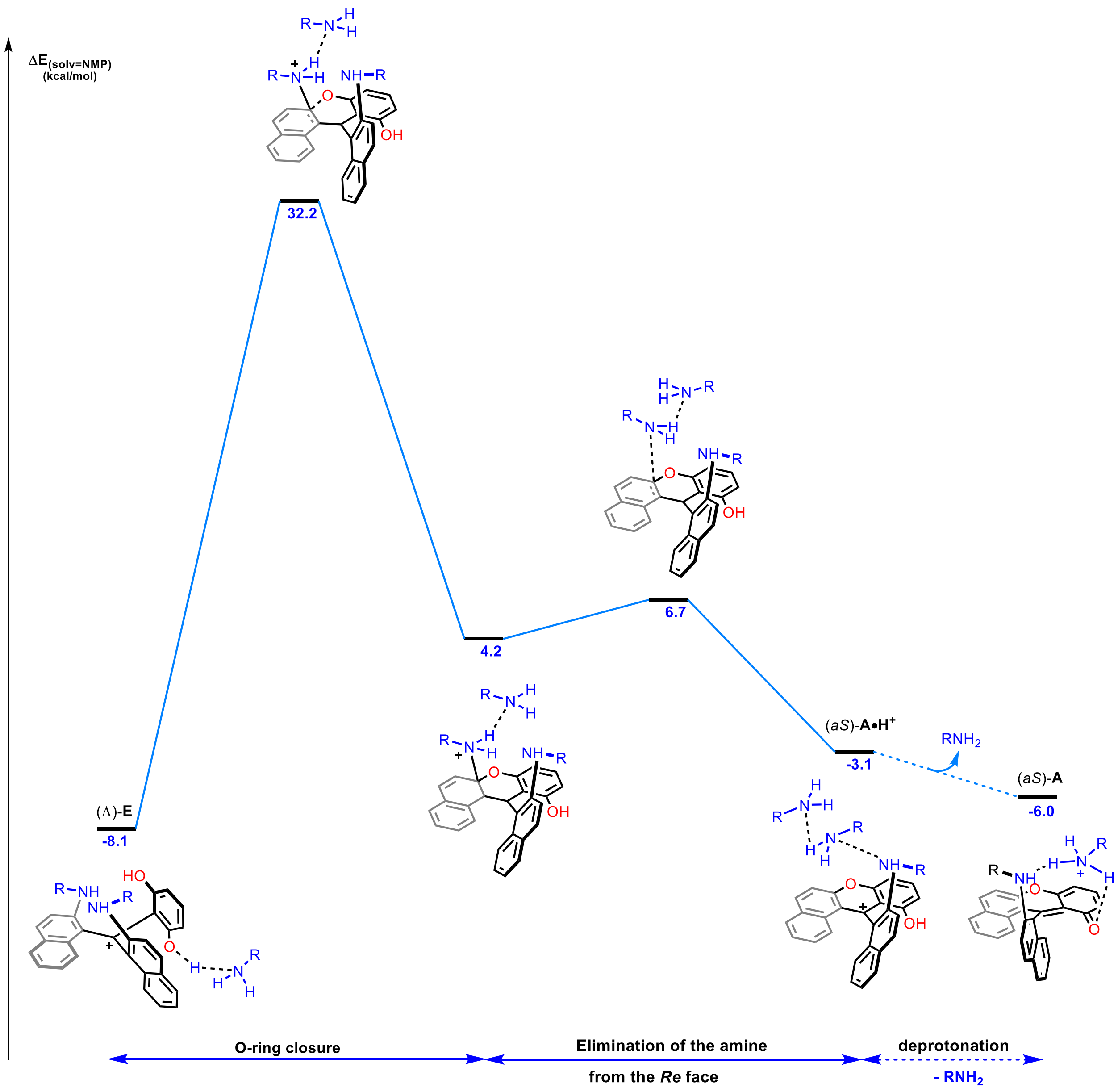

Figure S32. Reaction energy profile for the O-ring closure of (Λ)-**E** leading to (*aS*)-**A·H**$^+$. Solvation Energies (solv = NMP) in kcal mol^{-1}.

Formation of Dibenzoacridinium **5**.

The aza ring closure leading to the formation of the acridinium core of **5** occurs from a propeller intermediate **F** possessing its two amino group away from the central phenyl ring (Figure S33). Such propeller **F** is obtained by isomerization from **E**. This isomerization of (Δ)-**E** to (Λ)-**F** follows a two ring flip process. Although this process is not favored ((Λ)-**F** at -0.8 kcal mol^{-1}), the low energy of the associated transition state (TS at 3.0 kcal mol^{-1}) suggests a fast equilibrium. The extra amine is then replace by a carboxylate that will facilitate the aza ring closure through a TS located at +9.4 kcal mol^{-1}. The subsequent proton migration to the exo amino group is assisted by the carboxylate (TS at +4.1 kcal mol^{-1}). Such species undergoes then a facile deamination yielding dibenzoacridinium **5** that is located at -17.7 kcal mol^{-1}.

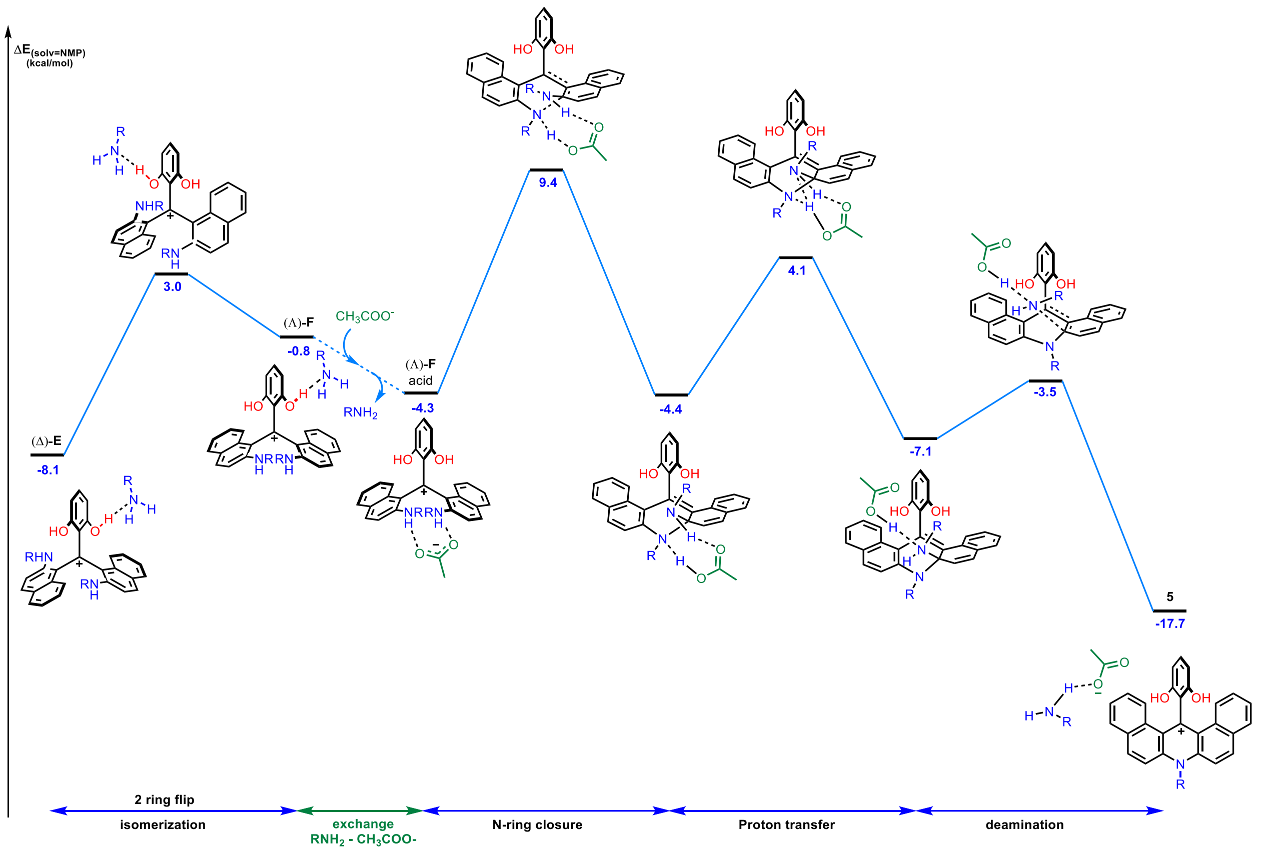

Figure S33. Reaction energy profile for the formation of the the Dibenzoacridinium X after (Δ)-**E** → (Λ)-**F** isomerization. Solvation Energies (solv = NMP) in kcal mol^{-1}.

From azaoxa **2** to diaza **3**

The transformation of azaoxa **2** to diaza **3** has been studied in the (*M*) series.

Reaction occurring on the *Re* face of (*M*)-**2**

The reaction from (*M*)-azaoxa **2** to (*M*)-diaza **3** depicted in Figure S34 proceeds in a similar fashion than the formation of the azaoxa discussed in the main text. Noteworthy, this reaction occurs with retention of the configuration. As discussed before, the nucleophilic attack is preferred on the *Re* face of the azaoxa **2**. Amine coordination and opening of the azaoxa helicene requires an activation barrier of 27.9 kcal mol^{-1} that leads to the formation of biaryl (*aR*)-**G** intermediate at -4.4 kcal mol^{-1}. In this case, the amine coordination intermediate was not located as the relaxation of the transition state of the O-ring opening leads directly to the reactant. Nevertheless, along the relaxation a plateau shows a similar intermediate that is depicted in Figure S25. The structural analysis of biaryl (*aR*)-**G** intermediate indicates again a neutral derivative with the positive charge located on the ammonium moiety, linked to the carbonyl through a H-bond. Again, this reaction occurs with retention of the configuration. From intermediate **G**, a carboxylate – amine exchange is required to model the N-ring closure to form the (*M*)-diaza helicene. The formation of the final C-N bond require an activation barrier of +22.6 kcal mol^{-1}, the lower computed so far, where the acid promote both the deprotonation of the amino group and the protonation of the oxygen atom. The final dehydration step is barrierless and the transition state was located at only +8.0 kcal mol^{-1}. In this case, the ring opening is the rate-limiting step of the reaction. The overall process is exothermic by almost 17 kcal mol^{-1}.

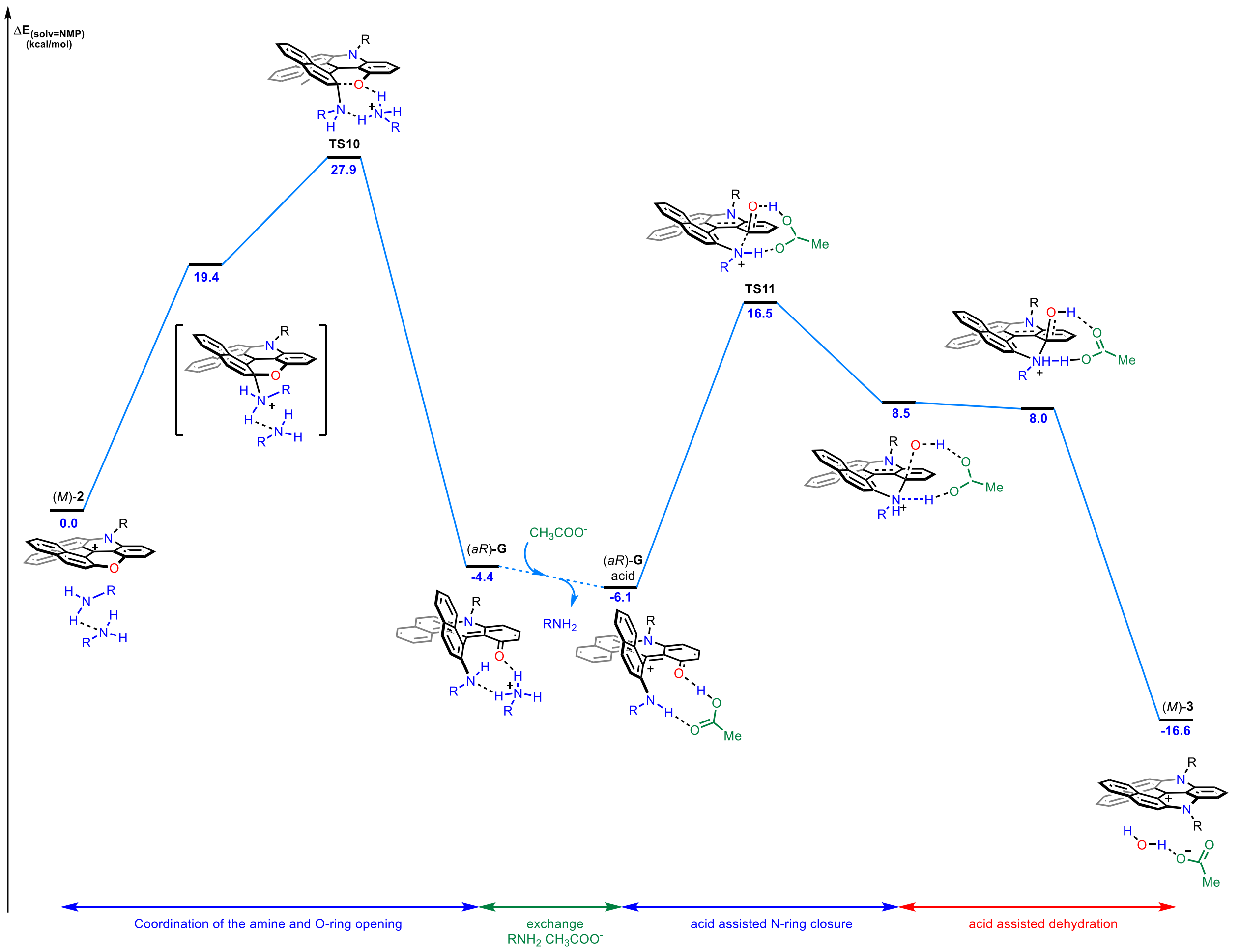

Figure S34. Reaction energy profile for the formation of (*M*)-diaza **3** by the amine attack on the *Re* face of the (*M*)-azaoxa **2** and *via* intermediate (*aR*)-**G**. Solvation Energies (solv = NMP) in kcal mol^{-1}.

Reaction occurring on the *Si* face of (*M*)-**2**

The approach of the two amines on the *Si* face of (*M*)-**2** was also evaluated and it is depicted in Figure S35, and it behaves similarly to the dioxa **1** helicene. The coordination of the two amines leads to an adduct located at +12.9 kcal mol^{-1}. The formation of such species occurs during a late transition state at +13.8 kcal mol^{-1}. As observed before, the search of the potential energy surface did not achieved the location of the transition state for the O-ring opening. On top of that, the final **G**-*Si* structure is not stable as compared to the starting material (ΔE = +9.5 kcal mol^{-1}). This again pinpoints towards a stereoselective opening of the azaoxa **2** that can occur only on the *Re* face.

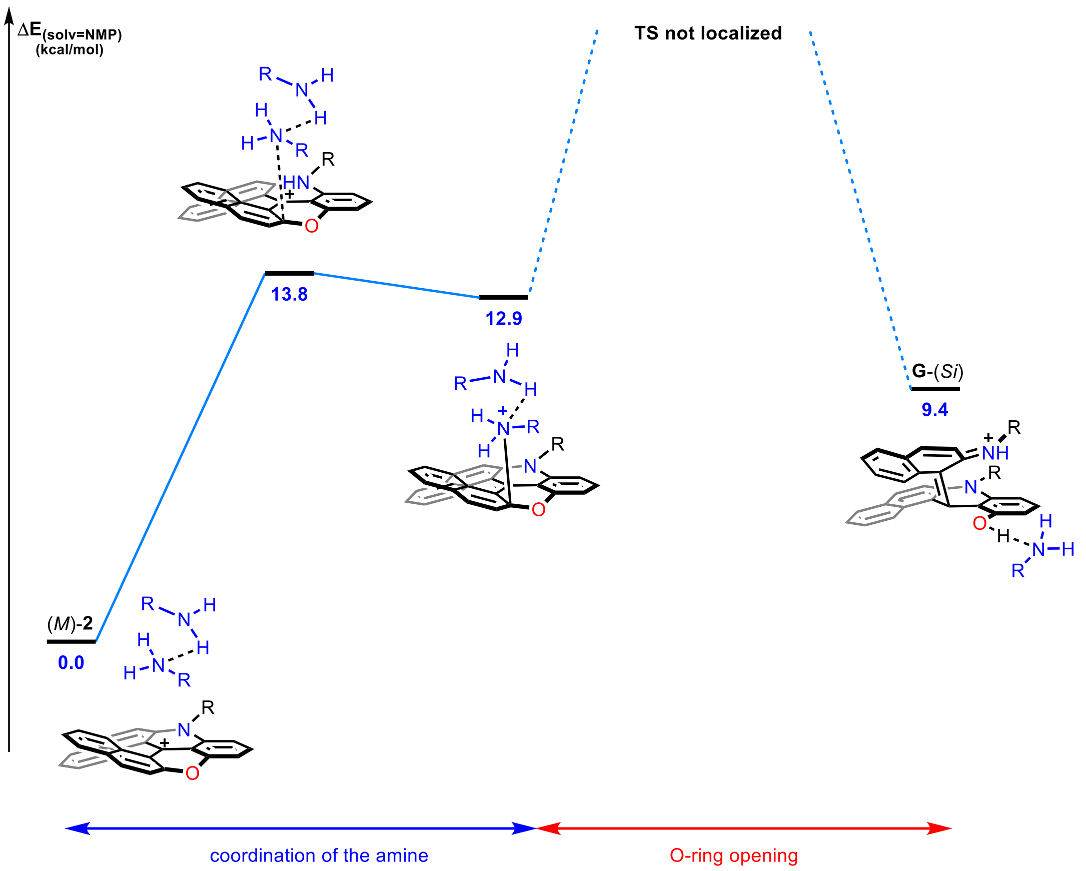

Figure S35. Reaction energy profile for the ring opening of the (*M*)-azaoxa **2** by the amine attack on the *Si* face. Solvation Energies (solv = NMP) in kcal mol^{-1}.

<u>N-ring opening of cationic (*aR*)-**G·H**$^{+}$</u>

Following the same analysis than for intermediate **A**, the reaction with an extra amine in protonated intermediate **G** was investigated, both from the *Si* or the *Re* faces. Contrary to the previous case, further reactivity of intermediate **G** with extra amines requires high-energy barriers and, therefore the isomerization pathway *via* propeller intermediates is disfavored here. Nevertheless, results for the first amine attacks are discussed below.

<u>N-ring opening of cationic (*aR*)-**G·H**$^{+}$ from the *Si* face</u>

The protonation of (*aR*)-**G** to form **G·H**$^{+}$ intermediate is depicted in Figure S36. This intermediate models the amine cluster interacting by H-bond to the hydrogen atoms of the side chain of **G·H**$^{+}$. The following N-ring opening not only requires a very high activation energy ($\Delta\Delta E^{\ddagger}$ = +50.6 kcal mol^{-1}) but also leads to a highly destabilize (Δ) three bladed propeller system, localized at +40.6 kcal mol^{-1}. Overall, this reaction is not thermodynamically favored.

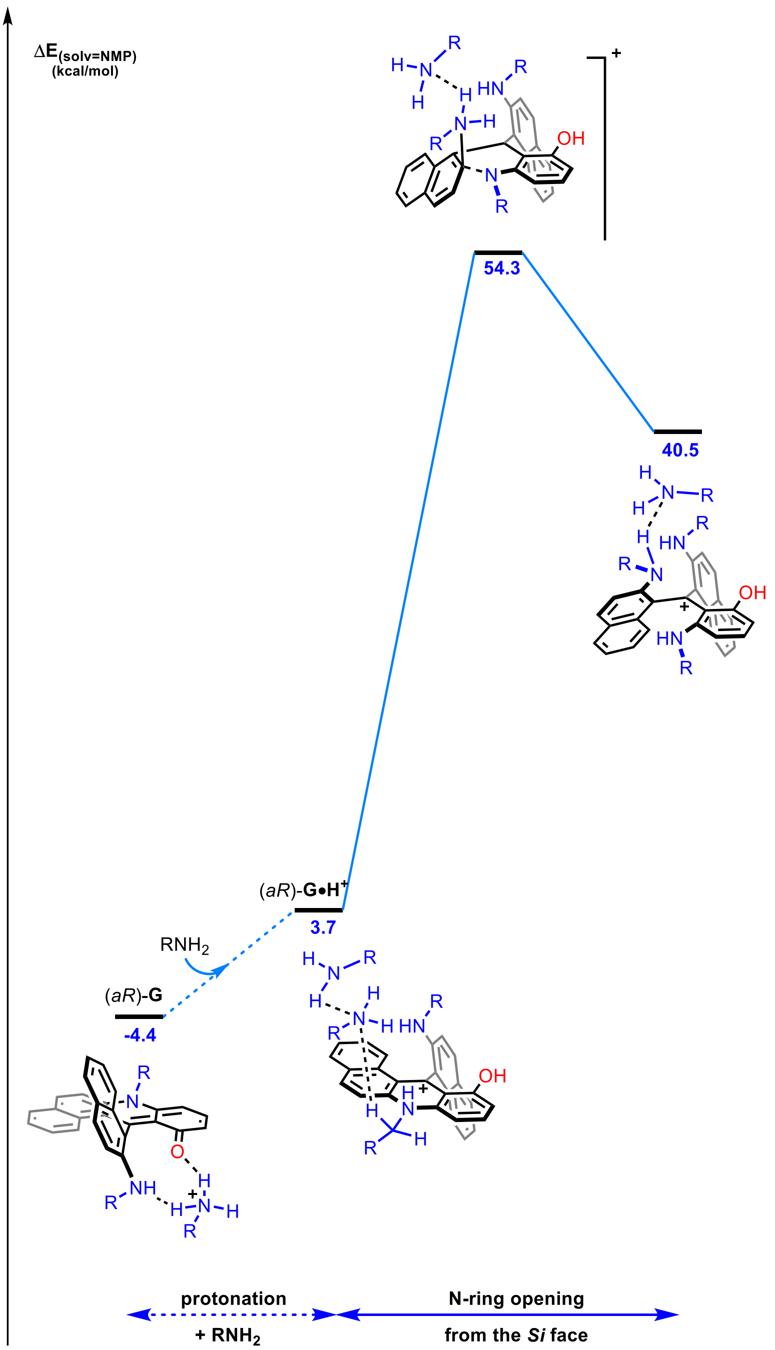

Figure S36. Reaction energy profile for the ring opening of the intermediate (*aR*)-**G** by the amine attack on the *Si* face. Solvation Energies (solv = NMP) in kcal mol^{-1}.

<u>N-ring opening of cationic (*aR*)-**G·H**$^+$ from the *Re* face</u>

The opening of the N-ring of intermediate **G** was also investigated from the *Re* face (Figure S37). A similar behavior was computed and, again, the ring opening requires a high activation energy ($\Delta\Delta E^{\ddagger}$ = +45.0 kcal mol^{-1}) and the overall reaction is endothermic by of 47.5 kcal mol^{-1}.

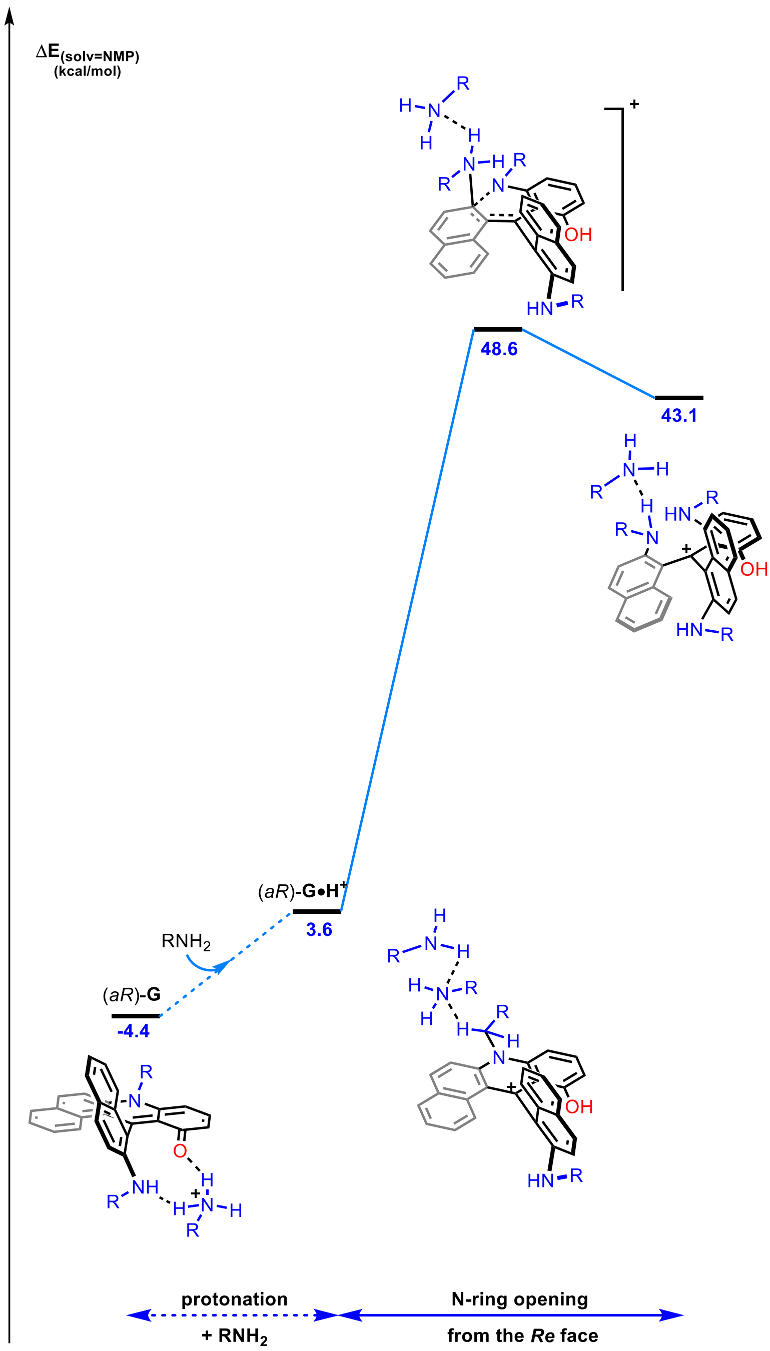

Figure S37. Reaction energy profile for the ring opening of the intermediate (*aR*)-**B** by the amine attack on the *Re* face. Solvation Energies (solv = NMP) in kcal mol^{-1}.

8. Synthesis and characterization of new derivatives

Synthesis azaoxa [6]Helicene of type **2** from dioxa [6]Helicene **1**

General procedure

To a solution of dioxa [6]helicene **1** in NMP (0.5 mL) were added benzoic acid (1.5 equiv) amine (3 equiv). The mixture was stirred at 60 °C while conversion of starting material was monitored by TLC and MS-ESI. After completion of reaction, the reaction mixture was cooled to 20 °C. Et_2O (*ca*. 10 mL) was added leading to the precipitation of the crude material. The resulting solid was dissolved in CH_2Cl_2 (*ca.* 5 mL) and washed with aqueous 1M HBF_4 solution (3 x 10 mL). The organic layer was dried over Na_2SO_4, filtrated and evaporated under reduced pressure. The solid obtained was dissolved in CH_2Cl_2 (*ca.* 1 mL) and precipitated by addition of Et_2O (*ca.* 10 mL). The precipitate was separated from the mother liquor by centrifugation. The product was then purified by flash chromatography (CombiFlash, SiO_2 4 g cartridge, CH_2Cl_2/MeOH, 100:0 to 95:5 over 30 min) yielding the corresponding azaoxa [6]helicene **2** as a pink powder.

11-propylbenzo[a]benzo[5,6]chromeno[2,3,4-kl]acridin-17c(11H)-ylium tetrafluoroborate **2a**

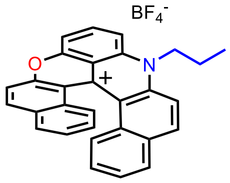

Dioxa **1** (25.0 mg, 0.05 mmol) benzoic acid (9.1 mg, 0.075 mmol) and *n*-propylamine (13 µL, 0.15 mmol) 9.5 mg of the desired compound were obtained (38%). **^{1}H NMR (500 MHz, CD_2Cl_2): δ** 8.57 (d, *J* = 9.6 Hz, 1 H), 8.38–8.31 (m, 2 H), 8.09 (d, *J* = 10 Hz, 1 H), 8.03–7.95 (m, 3 H), 7.86–7.81 (m, 2 H), 7.53 (t, *J* = 10 Hz, 1 H), 7.46–7.40 (m, 2 H), 6.93–7.01 (m, 3 H) 5.10–5.02 (m, 1 H), 4.90–4.82 (m, 1 H), 2.33–2.22 (m, 2 H), 1.35 (t, *J* = 8.6 Hz, 3 H). **^{13}C NMR (126 MHz, CD_2Cl_2): δ** 157.0 (C), 150.6 (C), 145.3 (C), 142.8 (C), 142.8 (C*H*), 140.1 (C*H*), 137.5 (C),136.4 (C*H*), 131.2 (C), 129.8 (C), 129.6 (C*H*), 129.5 (C*H*), 129.5 (C*H*), 129.0 (C*H*), 128.6 (C*H*), 127.9 (C), 127.8 (C), 127.5 (C*H*), 125.3 (C*H*), 124.1 (C*H*), 119.2 (C), 118.5 (C*H*), 117.8 (C*H*), 115,2 (C*H*), 110.9 (C*H*),110 (C), 110.3 (C*H*), 52.9 (C*H_2*), 21.9 (C*H_2*), 11.2 (C*H_3*). Data identical to the ones previously reported in the literature.[1]

11-octyl-11H-benzo[a]benzo[5,6]chromeno[2,3,4-kl]acridin-17c-ylium tetrafluoroborate **2b**

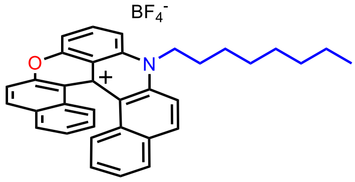

Dioxa **1** (25.0 mg, 0.05 mmol) benzoic acid (9.1 mg, 0.075 mmol) and *n*-octylamine (24.8 µL, 0.15 mmol) 7.4 mg of the desired compound were obtained (26%). **R*f*** (CH_2Cl_2/MeOH, 95:5): 0.12. **^{1}H NMR (500 MHz, CD_2Cl_2): δ** 8.58 (d, *J* = 9.5 Hz, 1H), 8.37 (d, *J* = 9.0 Hz, 1H), 8.33 (t, *J* = 8.4 Hz, 2H), 8.10 (d, *J* = 9.5 Hz, 1H), 8.03 (d, *J* = 8.0 Hz, 1H), 7.99 (d, *J* = 8.4 Hz, 1H), 7.96 (d, *J* = 8.0 Hz, 1H), 7.85 (d, *J* = 8.0 Hz, 1H), 7.82 (d, *J* = 9.0 Hz, 1H), 7.53 (t, *J* = 8.0 Hz, 1H), 7.44 (t, *J* = 8.0 Hz, 1H), 7.40 (d, *J* = 8.0 Hz, 1H), 7.02 – 6.89 (m, 3H), 5.16 – 5.04 (m, 1H), 4.99 – 4.83 (m,

1H), 2.29 – 2.15 (m, 2H), 1.80 – 1.69 (m, 2H), 1.45 – 1.31 (m, 5H), 0.90 (t, *J* = 7.2 Hz, 2H). **^{13}C NMR (126 MHz, CD_2Cl_2): δ** 157.1 (C+), 150.6 (C), 145.26 (C), 142.8 (CH), 142.7 (C), 140.1 (CH), 137.5 (C), 136.4 (CH), 131.2 (C), 129.8 (C), 129.7 (CH), 129.6 (C), 129.6 (CH), 129.5 (CH), 129.0 (CH), 128.6 (CH), 127.8 (C), 127.6 (CH), 125.3 (CH), 124.1 (CH), 119.2 (C), 118.6 (C), 117.9 (CH), 115.3 (CH), 114.8 (C), 111.0 (CH), 110.3 (CH), 32.1 (CH2), 30.1 (CH2), 29.6 (CH2), 29.6 (CH2), 28.4 (CH2), 27.2 (CH2), 23.0 (CH2), 14.2 (CH3). **^{19}F NMR (282 MHz, CD_2Cl_2): δ** -152.42, -152.37. **UV/VIS (CH_3CN, 2.10^{-5} M, λ_{max} (nm), (Log ε)):** 338 (4.03), 409 (3.84), 561 (3.97). **IR (neat, cm^{-1}): υ** 2928, 2856, 1624, 1597, 1571, 1548, 1529, 1510, 1487, 1459, 1438, 1381, 1348, 1263, 1244, 1208, 1162, 1141 1048, 871, 820, 786, 753, 729, 698, 594, 542. **HRMS** (**ESI**) calculated for (M^+): 482.2478. Found: 482.2490.

11-isopropyl-11H-benzo[a]benzo[5,6]chromeno[2,3,4-kl]acridin-17c-ylium tetrafluoroborate **2c**

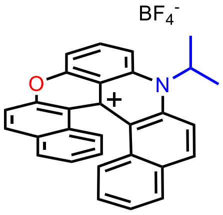

Dioxa **1** (25.0 mg, 0.05 mmol) benzoic acid (9.1 mg, 0.075 mmol) and isopropylamine (12.3 µL, 0.15 mmol) 5.0 mg of the desired compound were obtained (20 %). **R*f*** (CH_2Cl_2/MeOH, 95:5): 0.12. **^{1}H NMR (500 MHz, CD_2Cl_2): δ** 8.57 (d, *J* = 9.6 Hz, 1H), 8.39 (d, *J* = 9.0 Hz, 1H), 8.32–8.26 (m, 2H), 8.14 (d, *J* = 8.7 Hz, 1H), 8.03–7.97 (m, 2H), 7.86–7.83 (m, 2H), 7.54-7.44 (m, 2H), 7.40 (d, *J* = 8.7 Hz, 1H), 7.12 (d, *J* = 8.7 Hz, 1H), 7.01–6.93 (m, 2H) 5.89–5.82 (m, 1H), 2.15 (d, *J* = 7.0 Hz 3H), 2.04 (d, *J* = 7.0 Hz 3H). **^{13}C NMR (126 MHz, CD_2Cl_2): δ** 156.4 (C+), 149.7 (C), 145.7 (C), 142.7 (C), 142.0 (CH), 139.9 (CH), 136.8 (C), 135.0 (CH), 130.8 (C), 129.4 (C), 129.4 (CH), 129.3 (CH), 129.0 (CH), 128.7 (CH), 128.4 (CH), 128.3 (C), 127.3 (C), 127.2 (CH), 124.8 (CH), 123.9 (CH), 119.5 (C), 119.3 (C), 117.5 (CH), 115.5 (CH), 114.6 (C), 111.6 (CH), 110.3 (CH), 58.0 (CH), 21.2 (CH3), 20.6 (CH3). **^{19}F NMR (282 MHz, CD_2Cl_2): δ** -152.40, -152.45. **UV/VIS (CH_3CN, 2.10^{-5} M, λ_{max} (nm), (Log ε)):** 354 (3.97), 424 (3.95), 576 (4.03). **IR (neat, cm^{-1}): υ** 2918, 2857, 1625, 1597, 1572, 1548, 1527, 1488, 1434, 1378, 1340, 1264, 1245, 1217, 1161, 1131, 993, 873, 824, 791, 757, 729, 701, 596. **HRMS** (**ESI**) calculated for (M^+): 412.1696. Found: 412.1693.

11-benzylbenzo[a]benzo[5,6]chromeno[2,3,4-kl]acridin-17c(11H)-ylium tetrafluoroborate **2d**

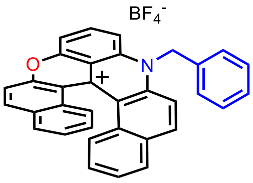

Reaction performed under N_2 atmosphere and in a tinted flask. Dioxa **1** (48.7 mg, 0.10 mmol) benzoic acid (19.4 mg, 0.15 mmol) and benzylamine (35 µL, 0.32 mmol). 11.0 mg of the desired compound were obtained (20 %). **R*f*** (CH_2Cl_2/MeOH, 95:5): 0.18. **^{1}H NMR (500 MHz, CD_2Cl_2): δ** 8.45 (d, *J* = 9.5 Hz, 1H), 8.41 (d, *J* = 9.0 Hz, 1H), 8.23 (t, *J* = 8.4 Hz, 1H), 7.97 (dd, *J* = 14.1, 8.4 Hz, 3H), 7.85 (dd, *J* = 8.7, 7.5 Hz, 3H), 7.53 (t, *J* = 8.1 Hz, 1H), 7.50 – 7.36 (m, 5H), 7.35 – 7.23 (m, 2H), 7.11 (dd, *J* = 8.7, 1.0 Hz, 1H), 7.04 – 6.91 (m, 2H), 6.43 (d, *J* = 18.1 Hz, 1H), 6.15 (d, *J* = 18.1 Hz, 1H). **^{13}C NMR (125 MHz, CD_2Cl_2): δ** 157.3(C+), 150.5(C), 146.1(C), 143.8(C), 143.1(CH), 140.5(CH), 138.2(C), 136.6(CH), 133.1(C), 131.2(C), 130.0(CH), 129.7(CH), 129.7(CH), 129.5(CH), 129.2(CH), 129.1(CH), 128.9(C), 128.8(CH), 127.9(C), 127.7(CH), 126.2(C), 126.1(CH), 125.3(CH), 124.4(CH), 119.3(C), 118.6(C), 117.9(CH), 115.8(CH), 115.0(C), 111.1(CH), 110.9(CH), 55.8(CH2). **^{19}F NMR (282 MHz, CD_2Cl_2): δ** -152.33, -152.28. **UV/VIS (CH_3CN, 2.10^{-5} M, λ_{max} (nm), (Log ε)):** 356 (4.38), 413 (4.34), 567 (4.45). **IR**

(neat, cm^{-1}): υ 2956, 2922, 2862, 1628, 1580, 1529, 1490, 1458, 1374, 1350, 1271, 1215, 1161, 1056, 819, 753, 727, 695, 617, 570, 542. **HRMS** (**ESI**) calculated for (M^+): 460.1696. Found: 460.1710.

11-allyl-11H-benzo[a]benzo[5,6]chromeno[2,3,4-kl]acridin-17c-ylium tetrafluoroborate **2e**

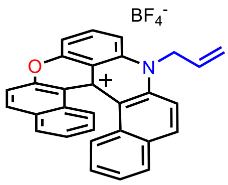

Reaction performed under N_2 atmosphere and in a tinted flask. Dioxa **1** (25.0 mg, 0.05 mmol) benzoic acid (9.1 mg, 0.075 mmol) and allylamine (11.2 µL, 0.15 mmol) 5.7 mg of the desired compound were obtained (23 %). **R*f*** (CH_2Cl_2/MeOH, 95:5): 0.40. **^{1}H NMR (500 MHz, CD_2Cl_2): δ** 8.55 (d, *J* = 9.5 Hz, 1H), 8.39 (d, *J* = 9.0 Hz, 1H), 8.30 (t, *J* = 8.4 Hz, 1H), 8.06 – 8.00 (m, 2H), 7.97 (d, *J* = 7.9 Hz, 1H), 7.94 (d, *J* = 8.7 Hz, 1H), 7.84 (dd, *J* = 8.7, 6.4 Hz, 2H), 7.53 (t, *J* = 7.5 Hz, 1H), 7.46 (t, *J* = 7.5 Hz, 1H), 7.42 (d, *J* = 8.4 Hz, 1H), 7.07 (dd, *J* = 8.5, 1.1 Hz, 1H), 7.01 – 6.88 (m, 2H), 6.49 – 6.37 (m, 1H), 5.87 – 5.75 (m, 1H), 5.60 – 5.46 (m, 2H), 5.15 (d, *J* = 17.4 Hz, 1H). **^{13}C NMR (126 MHz, CD_2Cl_2): δ** 157.2(C+), 150.4(C), 145.8(C), 143.4(C), 143.0(CH), 140.3(CH), 137.8(C), 136.5(CH), 131.2(C), 130.1(C), 129.7(CH), 129.4(CH), 129.2(CH), 129.0(CH), 128.8(C), 128.7(CH), 127.8(C), 127.7(CH), 125.3(CH), 124.3(CH), 119.2(C), 118.6(C), 117.9(CH), 115.8(CH), 114.9(C), 110.9(CH), 110.8(CH), 54.6(CH2), 30.1(CH), 30.0 (CH). **^{19}F NMR (282 MHz, CD_2Cl_2): δ** -152.51, -152.57. **UV/VIS (CH_3CN, 2.10^{-5} M, λ_{max} (nm), (Log ε)):** 354 (3.83), 426 (3.83), 578 (3.93). **IR (neat, cm^{-1}): υ** 3523, 2926, 1625, 1572, 1527, 1443, 1346, 1284, 1262, 1206, 1061, 875, 819, 791, 757. **HRMS** (**ESI**) calculated for (M^+): 410.1539. Found: 410.1535

11-(5-hydroxypentyl)-11H-benzo[a]benzo[5,6]chromeno[2,3,4-kl]acridin-17c-ylium tetrafluoroborate **2f**

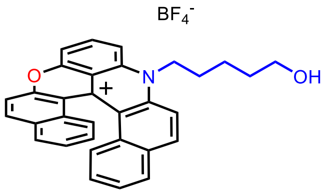

Dioxa **1** (27 mg, 0.05 mmol) benzoic acid (9 mg, 0.075 mmol) and 5-amino-1-pentanol (16 mg, 0.15 mmol) 7.9 mg of the desired compound were obtained (29%).

R*f* (CH_2Cl_2/MeOH, 95:5): 0.14. **^{1}H NMR (500 MHz, CD_2Cl_2): δ** 8.60 (d, *J* = 9.5 Hz, 1H), 8.37–8.33 (m, 2H), 8.20 (d, *J* = 9.5 Hz, 1H), 8.06 (d, *J* = 8.0 Hz, 1H) 8.02 (d, *J* = 8.0 Hz, 1H), 7.95 (d, *J* = 8.0 Hz, 1H), 7.85–7.80 (m, 2H), 7.53 (t, *J* = 10 Hz, 1H), 7.45–7.39 (m, 2H), 7.01-6.92 (m, 3H) 5.20–5.13 (m, 1H), 4.98–4.92 (m, 1H), 3.75 (t, *J* = 5.9 Hz, 2H) 2.33–2.25 (m, 2H), 1.90-1.78 (m, 4H). **^{13}C NMR (126 MHz, CD_2Cl_2): δ** 156.6 (C+), 150.1 (C), 145.0 (C), 142.5 (CH), 142.2 (C), 139.6 (CH), 137.1 (C), 136.0 (CH), 130.8 (C), 129.5 (C), 129.2 (CH), 129.2 (CH), 129.0 (CH), 128.6 (C), 128.5 (CH), 128.1 (CH), 127.4 (C), 127.1 (CH), 124.9 (CH), 123.7 (CH), 118.8 (C), 118.2 (C), 117.4 (CH), 115.1 (CH), 114.4 (C), 110.5 (CH), 110.1 (CH), 62.1 (CH2), 31.8 (CH2), 27.6 (CH2), 23.4 (CH2), 23.3 (CH2). **^{19}F NMR (282 MHz, CD_2Cl_2): δ** -152.01, -152.06. **UV/VIS (CH_3CN, 2.10^{-5} M, λ_{max} (nm), (Log ε)):** 355 (4.02), 421 (3.95), 573 (4.05) **IR (neat, cm^{-1}): υ** 3541, 3402, 2927, 2871, 1624, 1573, 1530, 1489, 1436, 1345, 1245, 1159, 1062, 820, 792, 756. **HRMS** (**ESI**) calculated for (M^+): 456.1958. Found: 456.1944.

11-(3-carboxypropyl)benzo[a]benzo[5,6]chromeno[2,3,4-kl]acridin-17c(11H)-ylium tetrafluoroborate **2g**

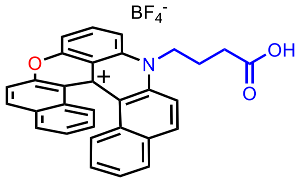

Dioxa **1** (49.1 mg, 0.1 mmol) benzoic acid (19.6 mg, 0.15 mmol) and butyric acid (33.1 mg, 0.3 mmol) 15.8 mg of the desired compound were obtained (29 %). ***Rf*** (CH_2Cl_2/MeOH, 95:5): 0.08. **^{1}H NMR (500 MHz, CD_2Cl_2): δ** 8.59 (d, *J* = 9.6 Hz, 1H), 8.44 (d, *J* = 9.6 Hz, 1H), 8.39 – 8.28 (m, 3H), 7.97 (d, *J* = 7.8 Hz, 1H), 7.93 (d, *J* = 7.8 Hz, 1H), 7.83 – 7.74 (m, 2H), 7.48 (t, *J* = 8.0 Hz, 1H), 7.41 (t, *J* = 8.0 Hz, 1H), 7.34 (d, *J* = 8.4 Hz, 1H), 7.00 – 6.86 (m, 3H), 5.24 (m, 1H), 5.05 (m, 1H), 2.93 (m, 2H), 2.58 – 2.31 (m, 2H). **^{13}C NMR (125 MHz, CD_2Cl_2): δ** 175.5(C+), 157.1(C), 150.5(C), 145.7(C), 143.2(CH), 142.8(C), 140.1(CH), 137.7(C), 136.8(CH), 131.3(C), 130.1(C), 129.7(CH), 129.7(CH), 129.6(C), 129.5(CH), 129.1(C), 129.0(CH), 128.7(CH), 128.0(C), 127.6(CH), 125.4(CH), 124.3(CH), 119.2(C), 118.6(C), 118.0(CH), 115.9(CH), 114.9(C), 111.1(CH), 111.0(CH), 30.9(CH2), 23.2(CH2). **^{19}F NMR (282 MHz, CD_2Cl_2): δ** -152.16, -152.21. **UV/VIS (CH_3CN, 2.10^{-5} M, λ_{max} (nm), (Log ε)):** 338 (4.08), 409 (3.92), 561 (4.01). **IR (neat, cm^{-1}): υ** 3073, 2918, 1707, 1619, 1595, 1570, 1533, 1514, 1484, 1438, 1406, 1387, 1344, 1266, 1243, 1211, 1159, 1045, 933, 873, 819, 746, 725, 692, 546. **HRMS** (**ESI**) calculated for (M^+): 456.1593. Found: 456.1594.

<u>11-(4-methoxy-4-oxobutyl)benzo[a]benzo[5,6]chromeno[2,3,4-kl]acridin-17c(11H)-ylium tetrafluoroborate **2h**</u>

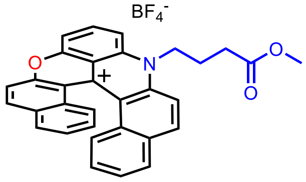

Dioxa **1** (47.3 mg, 0.1 mmol), *N,N*-Diisopropylethylamine (54 µL, 0.3 mmol) and 4-methoxy-4-oxobutan-1-aminium chloride (47.6 mg, 0.3 mmol). 13.9 mg of the desired compound were obtained (25 %). ***Rf*** (CH_2Cl_2/MeOH, 95:5): 0.07. **^{1}H NMR (500 MHz, CD_2Cl_2): δ** 8.62 (d, *J* = 9.5 Hz, 1H), 8.43 (d, *J* = 9.6 Hz, 1H), 8.39 – 8.33 (m, 3H), 8.03 (d, *J* = 8.1 Hz, 1H), 7.96 (d, *J* = 8.1 Hz, 1H), 7.85 (dd, *J* = 6.7, 2.1 Hz, 1H), 7.82 (d, *J* = 9.0 Hz, 1H), 7.52 (t, *J* = 7.5 Hz, 1H), 7.47 – 7.37 (m, 2H), 7.06 – 6.91 (m, 3H), 5.19 – 5.27 (m, 1H), 5.10 – 5.00 (m, 1H), 3.82 (s, 3H), 2.88 (t, *J* = 6.2 Hz, 2H), 2.59 – 2.39 (m, 2H). **^{13}C NMR (125 MHz, CD_2Cl_2): δ** 174.02(C+), 157.08(C), 150.49(C), 145.53(C), 143.03(CH), 142.89(C), 140.05(CH), 137.58(C), 136.59(CH), 131.19(C), 129.92(C), 129.63(CH), 129.57(CH), 129.43(CH), 129.43(C), 129.02(C), 128.93(CH), 128.56(CH), 127.84(C), 127.53(CH), 125.30(CH), 124.12(CH), 119.18(C), 118.56(C), 117.85(CH), 115.59(CH), 114.82(C), 110.93(CH), 110.74(CH), 52.50(CH3), 30.28(CH2), 22.85(CH2). **^{19}F NMR (282 MHz, CD_2Cl_2): δ** -152.17, -152.23. **UV/VIS (CH_3CN, 2.10^{-5} M, λ_{max} (nm), (Log ε)):** 338 (3.65), 409 (3.46), 563 (3.60). **IR (neat, cm^{-1}): υ** 3060, 2943, 1726, 1649, 1623, 1600, 1572, 1544, 1524, 1511, 1488, 1441, 1346, 1262, 1245, 1208, 1176, 1157, 1047, 929, 869, 817, 791, 755, 727, 701, 596. **HRMS** (**ESI**) calculated for (M^+): 470.1751. Found: 470.1782.

<u>11-(2-(2-hydroxyethoxy)ethyl)-11H-benzo[a]benzo[5,6]chromeno[2,3,4-kl]acridin-17c-ylium tetrafluoroborate **2i**</u>

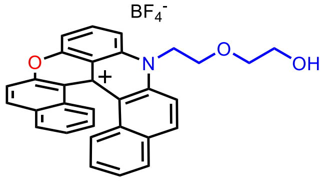

Dioxa **1** (25.0 mg, 0.05 mmol) benzoic acid (9.1 mg, 0.075 mmol) and 2-(2-Aminoethoxy) ethanol (15.0 µL, 0.15 mmol) 6.8 mg of the desired compound were obtained (25 %).

Rf (CH_2Cl_2/MeOH, 95:5): 0.11. **^{1}H NMR (500 MHz, CD_2Cl_2): δ** 8.56 (d, J = 9.5 Hz, 1H), 8.39 – 8.22 (m, 4H), 7.99 (d, J = 8.4 Hz, 1H), 7.94 (d, J = 7.4 Hz, 1H), 7.83 (d, J = 7.4 Hz, 1H), 7.80 (d, J = 8.0 Hz, 1H), 7.50 (t, J = 8.0 Hz, 1H), 7.42 (t, J = 8.0 Hz, 1H), 7.38 (d, J = 8.4 Hz, 1H), 7.00 – 6.86 (m, 3H), 5.50 – 5.40 (m, 1H), 5.33 – 5.22 (m, 1H), 4.63 – 3.97 (m, 2H),

3.78 – 3.36 (m, 4H), 2.12 (s, 1H). **^{13}C NMR (126 MHz, CD_2Cl_2): δ** 156.7(C+), 149.9(C), 145.9(C), 142.6(C), 142.0(CH), 139.6(CH), 137.8(C), 135.8(CH), 130.8(C), 129.5(C), 129.2(CH), 129.1(CH), 129.0(CH), 128.6(C), 128.4(CH), 128.1(CH), 127.5(C), 127.1(CH), 124.9(CH), 123.7(CH), 118.8(C), 118.2(C), 117.4(CH), 116.0(CH), 114.4(C), 111.3(CH), 110.5(CH), 73.2(CH2), 68.6(CH2), 61.6(CH2), 51.2(CH2). **^{19}F NMR (282 MHz, CD_2Cl_2): δ** -152.34, -152.39. **UV/VIS (CH_3CN, 2.10^{-5} M, λ_{max} (nm), (Log ε)):** 354 (4.04), 422 (3.99), 576 (4.09). **IR (neat, cm^{-1}):** 3524, 2929, 1624, 1577, 1527, 1443, 1344, 1288, 1262, 1241, 1206, 1060, 877, 820, 790,757. **HRMS** (**ESI**) calculated for (M^+): 458.1751. Found: 458.1754

Synthesis diaza [6]Helicene of type **3** from dioxa [6]Helicene **1**

General procedure

To a solution of dioxa [6]helicene **1** (0.1 mmol) in NMP (0.5 mL) were added benzoic acid (12.5 equiv) amine (25 equiv). The mixture was stirred at 70 °C while conversion of starting material was monitored by TLC and MS-ESI. After completion of reaction, the reaction mixture was then cooled to 20 °C. Et_2O (*ca*. 10 mL) was added leading to the precipitation of the crude material. The resulting solid was dissolved in CH_2Cl_2 (*ca.* 5 mL) and washed with aqueous 1 M HBF_4 solution (3 x 10 mL). The organic layer was dried over Na_2SO_4, filtrated and evaporated under reduced pressure. The solid obtained was dissolved in CH_2Cl_2 (*ca.* 1 mL) and precipitated by addition of Et_2O (*ca.* 10 mL). The precipitate was separated from the mother liquor by centrifugation. The product was then purified by flash chromatography (CombiFlash, SiO_2 4 g cartridge, CH_2Cl_2/MeOH, 100:0 to 95:5 over 30 min) yielding the corresponding diaza [6]helicene as a blue powder.

7,11-dipropyl-7,11-dihydro-17cH-benzo[a]benzo[5,6]quinolino[2,3,4-kl]acridin-17c-ylium tetrafluoroborate **3a**

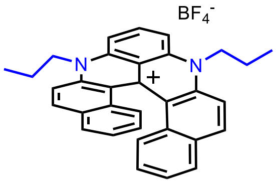

Dioxa **1** (25.0 mg, 0.05 mmol) benzoic acid (76.3 g, 0.625 mmol) and *n*-propylamine (102.0 µL, 1.25 mmol). 12.7 mg of the desired compound were obtained (47 %). **R*f*** (CH_2Cl_2/MeOH, 95:5): 0.14. **^{1}H NMR (500 MHz, CD_2Cl_2): δ** 8.39 (d, *J* = 9.4 Hz, 2H), 8.29 (t, *J* = 8.5 Hz, 1H), 7.97 (d, *J* = 9.5 Hz, 2H), 7.92 – 7.85 (m, 2H), 7.70 (d, *J* = 8.5 Hz, 2H), 7.36 (ddd, *J* = 8.0, 6.9, 1.1 Hz, 2H), 7.17 (dd, *J* = 8.5, 1.0 Hz, 2H), 6.83 (ddd, *J* = 8.5, 7.0, 1.4 Hz, 2H), 4.89-4.81 (m, 2H), 4.61-4.53 (m, 2H), 2.29-2.17 (m, 4H),, 1.32 (t, *J* = 7.4 Hz, 3H). **^{13}C NMR (125 MHz, CD_2Cl_2): δ** . 142.6 (C), 141.8 (CH), 139.1 (C), 138.4 (C), 135.5 (CH), 129.7 (C), 129.3 (CH), 129.0 (C), 128.2 (CH), 127.8 (CH), 123.1 (C), 121.9 (C), 116.6 (C), 115.4 (CH), 107.0 (CH), 52.1 (C*H$_2$*), 20.9 (C*H$_2$*), 11.3 (C*H$_3$*). Data identical to the ones previously reported in the literature.[1]

7,11-diisopropyl-7,11-dihydrobenzo[a]benzo[5,6]quinolino[2,3,4-kl]acridin-17c-ylium tetrafluoroborate **3b**

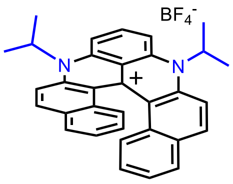

Dioxa **1** (25.0 mg, 0.05 mmol) benzoic acid (76.3 g, 0.625 mmol) and isopropylamine (102.0 µL, 1.25 mmol). 6.5 mg of the desired compound were obtained (24 %). **R*f*** (CH_2Cl_2/MeOH, 95:5): 0.14. **^{1}H NMR (500 MHz, CD_2Cl_2): δ** 8.37 (d, *J* = 9.5 Hz, 2H), 8.19 (t, *J* = 8.5 Hz, 1H), 8.15 (d, *J* = 9.5 Hz, 2H), 7.92 (d, *J* = 9.5 Hz, 2H), 7.83 (d, *J* = 8.5 Hz, 2H), 7.41 (t, *J* = 8.0 Hz, 2H), 7.30 (d, *J* = 8.5 Hz, 2H), 6.90 (t, *J* = 8.5 Hz, 2H), 5.53-5.47 (m, 2H), 2.08-2.17 (m, 4H), 2.08 (d, *J* = 7.0 Hz, 3H), 1.98 (d, *J* = 7.0 Hz, 3H). **^{13}C NMR (125 MHz, CD_2Cl_2): δ** 142.9 (C+), 138.9 (CH), 137.6 (C), 134.0 (CH), 129.6 (C), 129.3 (CH), 128.6 (C), 128.5 (CH), 128.0 (CH), 123.6 (C), 123.4 (C), 123.2 (CH), 118.6 (C), 116.4 (CH), 108.6 (CH), 57.4 (CH), 21.7 (CH3), 20.6 (CH3). **^{19}F NMR (282 MHz, CD_2Cl_2): δ** -151.39, -151.44. **UV/VIS (CH_3CN, 2.10^{-5} M, λ_{max} (nm), (Log ε)):** 372 (4.22), 419 (3.93), 620 (4.15). **IR (neat, cm^{-1}): υ** 3586, 2940, 1607, 1567, 1548, 1524, 1496, 1378, 1329, 1259, 1215, 1159, 1137, 1058, 813, 756. **HRMS** (**ESI**) calculated for (M^+): 453.2325. Found: 453.2308.

7,11-dioctyl-7,11-dihydrobenzo[a]benzo[5,6]quinolino[2,3,4-kl]acridin-17c-ylium tetrafluoroborate **3c**

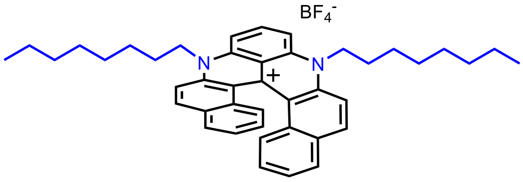

Dioxa **1** (25.0 mg, 0.05 mmol) benzoic acid (76.3 g, 0.63 mmol) and *n*-octylamine (206.0 µL, 1.25 mmol). 8.2 mg of the desired compound were obtained (24 %). **R*f*** (CH_2Cl_2/MeOH, 95:5): 0.16. **^{1}H NMR (500 MHz, CD_2Cl_2): δ** 8.37 (d, *J*=9.4 Hz, 2H), 8.25 (d, *J*=8.5 Hz, 1H), 7.93 (d, *J*=9.5 Hz, 2H), 7.88 (d, *J*=7.6 Hz, 2H), 7.66 (d, *J*=8.5 Hz, 2H), 7.47-7.31 (m, 2H), 7.14 (d, *J*=8.5 Hz, 2H), 6.81 (dd, *J*=8.4, 7.0 Hz, 2H), 4.95-4.76 (m, 2H), 4.60 (m, 2H), 2.30-1.99 (m, 4H), 1.76-1.68 (m, 4H), 1.47 (m, 12H), 0.96 (t, *J*=7.2 Hz, 6H). **^{13}C NMR (125 MHz, CD_2Cl_2): δ** 142.2 (C+), 141.4 (C), 139.2 (C), 138.1 (CH), 135.2 (C), 129.4 (C), 129.0 (CH), 128.7 (CH), 127.9 (CH), 127.5 (CH), 122.7 (C), 121.6 (C), 116.3 (CH), 115.0 (CH), 106.6 (CH), 50.5 (CH2), 31.8(CH2), 29.3 (CH2), 27.1 (CH2), 26.9 (CH2), 22.7 (CH2), 13.9 (CH3). **^{19}F NMR (282 MHz, CD_2Cl_2): δ** -152.54, -153.49. **UV/VIS (CH_3CN, 2.10^{-5} M, λ_{max} (nm), (Log ε)):** 374 (4.31), 419 (3.82), 579 (4.01), 622 (4.23). **IR (neat, cm^{-1}): υ** 3544, 2923, 2853, 1609, 1572, 1548, 1517, 1487, 1459, 1438, 1415, 1337, 1284, 1261, 1242, 1209, 1183, 1161, 1031, 892, 816, 789, 748, 674. **HRMS** (**ESI**) calculated for (M^+): 593.3890. Found: 593.3878. Data identical to the ones previously reported in the literature.[1]

7,11-dibenzyl-7,11-dihydro-17cH-benzo[a]benzo[5,6]quinolino[2,3,4-kl]acridin-17c-ylium tetrafluoroborate **3d**

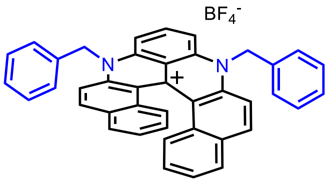

Reaction performed under N_2 atmosphere and in a tinted flask. Dioxa **1** (25.0 mg, 0.05 mmol) benzoic acid (76.0 mg, 0.63 mmol) and benzylamine (137.0 µL, 1.25 mmol) 6.1 mg of the desired compound were obtained (19 %). **R*f*** (CH_2Cl_2/MeOH, 95:5): 0.21. **^{1}H NMR (500 MHz, CD_2Cl_2): δ** 8.31 (d, *J* = 9.5 Hz, 2H), 8.06 (t, *J* = 8.5 Hz, 1H), 7.90 (d, *J* = 6.7 Hz, 2H), 7.83 (d, *J* = 9.5 Hz, 2H), 7.54 (d, *J* = 8.5 Hz, 2H), 7.50 – 7.37 (m, 10H), 7.36 – 7.27 (m, 4H), 6.91 (t, *J* = 7.8 Hz, 2H), 6.26 (d, *J* = 18.1 Hz, 2H), 5.88 (d, *J* = 18.1 Hz, 2H). **^{13}C NMR (125 MHz, CD_2Cl_2): δ** 143.4(C+), 142.5(C), 140.1(CH), 138.7(C), 135.9(CH), 133.5(C), 130.0(CH), 129.4(C), 129.2(CH), 129.1(C), 128.9(CH), 128.5(CH), 128.1(CH), 126.2(CH), 123.4(CH), 122.1(C), 117.2(C), 115.9(CH), 108.0(CH),

55.3(CH2). **^{19}F NMR (282 MHz, CD_2Cl_2): δ** -152.37, -152.38. **UV/VIS (CH_3CN, 2.10^{-5} M, λ_{max} (nm), (Log ε)):** 369 (4.12), 410 (3.63), 611(4.04). **IR (neat, cm^{-1}): υ**. 3434, 2926, 2869, 2158, 1658, 1504, 1466, 1427, 1404, 1300, 1262, 1171, 1113, 1064, 1023, 985, 927, 843, 782, 719, 654, 560. **HRMS** (**ESI**) calculated for (M^+): 549.2325. Found: 549.2320.

<u>7,11-diallyl-7,11-dihydrobenzo[a]benzo[5,6]quinolino[2,3,4-kl]acridin-17c-ylium tetrafluoroborate **3e**</u>

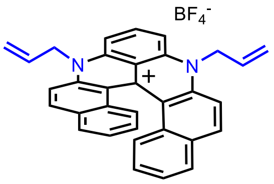

Reaction performed under N_2 atmosphere and in a tinted flask. Dioxa **1** (25.0 mg, 0.05 mmol) benzoic acid (76.3 mg, 0.625 mmol) and allylamine (94.0 µL, 1.25 mmol) 7.0 mg of the desired compound were obtained (26 %). **R*f*** (CH_2Cl_2/MeOH, 95:5): 0.10. **^{1}H NMR (500 MHz, CD_2Cl_2): δ** 8.38 (d, *J* = 9.4 Hz, 2H), 8.22 (t, *J* = 8.5 Hz, 1H), 7.89 (d, *J* = 9.2 Hz, 2H), 7.66 (d, *J* = 8.5 Hz, 2H), 7.39 (t, *J* = 7.4 Hz, 4H), 7.25 (d, *J* = 8.6 Hz, 2H), 6.87 (t, *J* = 8.4 Hz, 3H), 6.39 (m, 2H), 5.57 (m, 3H), 5.21 (m, 4H). **^{13}C NMR (101 MHz, CD2Cl2) δ** 143.2 (C), 142.4 (C+), 140.0 (CH), 138.5 (C), 135.9 (CH), 135.7 (C), 130.1 (C), 129.5 (CH), 129.3 (CH), 129.2 (C), 128.5 (CH), 128.1 (CH), 123.5 (CH), 119.1 (CH2), 117.1 (C), 116.1 (CH), 107.9 (CH), 53.9 (CH2). **^{19}F NMR (282 MHz, CD_2Cl_2): δ** -152.52, -152.58. **UV/VIS (CH_3CN, 2.10^{-5} M, λ_{max} (nm), (Log ε)):** 372 (4.44), 417 (4.06), 618 (4.33). **IR (neat, cm^{-1}): υ** 3601, 2924, 2857, 1612, 1573, 1548, 1489, 1339, 1262, 1211, 1165 1058, 869, 818, 784, 756. **HRMS** (**ESI**) calculated for (M^+): 449.2012. Found: 449.2014.

<u>7,11-bis(5-hydroxypentyl)-7,11-dihydrobenzo[a]benzo[5,6]quinolino[2,3,4-kl]acridin-17c-ylium tetrafluoroborate **3f**</u>

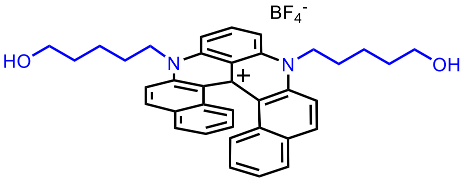

Dioxa. **1** (25 mg, 0.05 mmol) benzoic acid (9 mg, 0.63 mmol) and 5-amino-1-pentanol (129.0 mg, 1.25 mmol) 8.2 mg of the desired compound were obtained (26%). **R*f*** (CH_2Cl_2/MeOH, 95:5): 0.16. **^{1}H NMR (500 MHz, CD_2Cl_2): δ** 8.39 (d, *J* = 9.4 Hz, 2H), 8.29 (t, *J* = 8.4 Hz, 1H), 8.00 (d, *J* = 9.5 Hz, 2H), 7.88 (d, *J* = 8.4 Hz, 2H), 7.72 (d, *J* = 8.5 Hz, 2H), 7.35 (t, *J* = 8.4 Hz, 2H), 7.16 (d, *J* = 8.5 Hz, 2H), 6.83 (t, *J* = 8.4 Hz, 2H), 4.95-4.89 (m, 2H), 4.67-4.61 (m, 2H), 3.75 (t, *J* = 5.4 Hz, 4H), 2.29-2.19 (m, 4H), 1.85-1.78 (m, 10H). **^{13}C NMR (125 MHz, CD_2Cl_2): δ** 142.5 (C+), 141.7 (C), 139.5 (CH), 138.3 (C), 135.7 (CH), 129.7 (C), 129.3 (C), 129.0 (CH), 128.1 (CH), 127.7 (CH), 123.0 (CH), 121.9 (C), 116.6 (C), 115.4 (CH), 107.0 (CH), 62.6 (CH2), 50.8 (CH2), 32.4 (CH2), 27.1 (CH2), 23.7 (CH2). **^{19}F NMR (282 MHz, CD_2Cl_2): δ** -152.54, -152.59. **UV/VIS (CH_3CN, 2.10^{-5} M, λ_{max} (nm), (Log ε)):** 373 (4.27), 415 (3.79), 619 (4.19). **IR (neat, cm^{-1}): υ** 2926, 2851, 1610, 1574, 1550, 1339, 1256, 1161, 1058, 823, 783, 756. **HRMS** (**ESI**) calculated for (M^+): 541.2850. Found: 541.2830. Data identical to the ones previously reported in the literature.[1]

<u>7,11-bis(4-methoxy-4-oxobutyl)-7,11-dihydro-17cH-benzo[a]benzo[5,6]quinolino[2,3,4-kl]acridin-17c-ylium tetrafluoroborate **3h**</u>

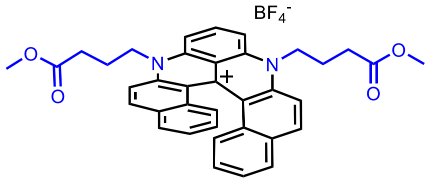

Dioxa **1** (48.6 mg, 0.1 mmol), *N,N*-Diisopropylethylamine (450 µL, 2.5 mmol) and 4-methoxy-4-oxobutan-1-aminium chloride (396 mg, 2.5 mmol). 11.8 mg of the desired compound were obtained (18 %). **R*f*** (CH_2Cl_2/MeOH, 95:5): 0.19. **^{1}H NMR (500 MHz, CD_2Cl_2): δ** 8.42 (d, J = 9.4 Hz, 2H), 8.34 (t, J = 8.5 Hz, 1H), 8.21 (d, J = 9.5 Hz, 2H), 7.96 (d, J = 8.5 Hz, 2H), 7.89 (d, J = 7.8 Hz, 2H), 7.36 (t, J = 8.0 Hz, 2H), 7.17 (d, J = 8.5 Hz, 2H), 6.83 (t, J = 7.8 Hz, 2H), 5.11 – 4.90 (m, 2H), 4.81 – 4.65 (m, 2H), 3.82 (s, 6H), 2.81 (t, J = 6.3 Hz, 4H), 2.59 – 2.47 (m, 2H), 2.48 – 2.34 (m, 2H). **^{13}C NMR (125 MHz, CD_2Cl_2): δ** 173.8(C+), 142.7(C), 142.0(C), 139.7(CH), 138.4(C), 135.9(CH), 129.7(C), 129.3(C), 129.0(CH), 128.2(CH), 127.8(CH), 123.0(CH), 121.9(C), 116.6(C), 115.4(CH), 107.1(CH), 52.5(CH3), 49.8(CH2), 30.6(CH2), 22.0(CH2). **^{19}F NMR (282 MHz, CD_2Cl_2): δ** -152.09, -152.13. **UV/VIS (CH_3CN, 2.10^{-5} M, λ_{max} (nm), (Log ε)):** 368 (4.29), 410 (3.68), 614 (4.14). **IR (neat, cm^{-1}): υ** 2956, 2922, 2857, 1729, 1610, 1574, 1550, 1527, 1492, 1441, 1417, 1367, 1337, 1262, 1208, 1180, 1159, 1056, 895, 873, 819, 785, 759, 667, 617, 581. **HRMS** (**ESI**) calculated for (M^+): 569.2435. Found: 569.2435

7,11-bis(2-(2-hydroxyethoxy)ethyl)-7,11-dihydrobenzo[a]benzo[5,6]quinolino[2,3,4-kl]acridin-17c-ylium tetrafluoroborate **3i**

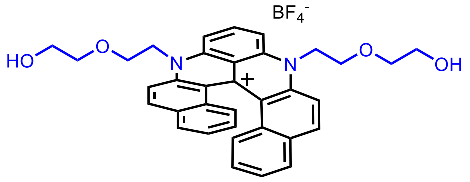

Dioxa **1** (25.0 mg, 0.05 mmol) benzoic acid (76.3 mg, 0.63 mmol) and 2-(2-Aminoethoxy)ethanol (125.0 µL, 1.25 mmol) 9.5 mg of the desired compound were obtained (30 %). **R*f*** (CH_2Cl_2/MeOH, 95:5): 0.17. **^{1}H NMR (500 MHz, CD_2Cl_2): δ** 8.37 (d, *J* = 9.4 Hz, 2H), 8.28 (t, *J* = 8.5 Hz, 1H), 8.11 (d, *J* = 9.5 Hz, 2H), 7.91 (d, *J* = 8.5 Hz, 2H), 7.88 (d, *J* = 8.5 Hz, 2H), 7.36 (t, *J* = 8.0 Hz, 2H), 7.14 (d, *J* = 8.5 Hz, 2H), 6.82 (t, *J* = 8.5 Hz, 2H), 5.23 – 5.13 (m, 2H), 5.01 – 4.88 (m, 2H), 4.36 – 4.22 (m, 4H), 3.77 – 3.54 (m, 8H).**^{13}C NMR (125 MHz, CD_2Cl_2): δ** 143.3(C+), 142.3(C), 139.3(CH), 138.8(C), 135.4(CH), 129.7(C), 129.3(C), 128.9(CH), 128.2(CH), 127.8(CH), 123.1(CH), 122.0(C), 116.8(C), 115.9(CH), 108.0(CH), 73.5(CH2), 68.5(CH2), 62.1(CH2), 50.6(CH2). **^{19}F NMR (282 MHz, CD_2Cl_2): δ** -152.16, -152.21. **UV/VIS (CH_3CN, 2.10^{-5} M, λ_{max} (nm), (Log ε)):** 372 (4.22), 415 (3.79), 616 (4.14). **IR (neat, cm^{-1}): υ** 3547, 3387, 2922, 1612, 1572, 1546, 1492, 1335, 1262, 1206, 1157, 1065, 888, 817, 787, 757. **HRMS** (**ESI**) calculated for (M^+): 545.2435. Found: 545.2444.

Synthesis diaza [6]Helicene of type **3** from azaoxa [6]Helicene of type **2**

General procedure

To a solution of azaoxa [6]helicene **2** (0.1 mmol) in NMP (0.5 mL)) were added benzoic acid (12.5 equiv) amine (25 equiv). The mixture was stirred at 70 °C while conversion of starting material was monitored by TLC and MS-ESI. After completion of reaction, the reaction mixture was cooled to 20 °C. Et_2O (*ca.* 10 mL) was added leading to the precipitation of the crude material. The resulting solid was dissolved in CH_2Cl_2 (*ca.* 5 mL) and washed with aqueous 1 M HBF_4 solution (3 x 10 mL). The organic layer was dried over Na_2SO_4, filtrated and evaporated under reduced pressure. The solid obtained was dissolved

in CH_2Cl_2 (*ca.* 1 mL) and precipitated by addition of Et_2O (*ca.* 10 mL). The precipitate was separated from the mother liquor by centrifugation. The product was then purified by flash chromatography (CombiFlash, SiO_2 4 g cartridge, CH_2Cl_2/MeOH, 100:0 to 95:5 over 30 min) yielding the corresponding diaza [6]helicene **3** as a blue powder.

7,11-dipropyl-7,11-dihydro-17cH-benzo[a]benzo[5,6]quinolino[2,3,4-kl]acridin-17c-ylium tetrafluoroborate **3a**

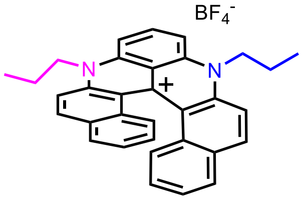

Azaoxa **2a** (24.0 mg, 0.05 mmol) benzoic acid (76.0 mg, 0.63 mmol) and *n*-propylamine (102.0 µL, 1.25 mmol). 9.7 mg of the desired compound were obtained (36 %).

7-octyl-11-propyl-7,11-dihydrobenzo[a]benzo[5,6]quinolino[2,3,4-kl]acridin-17c-ylium tetrafluoroborate **3j**

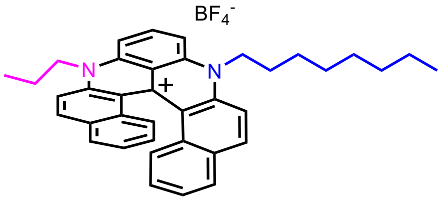

Azaoxa **2a** (24.0 mg, 0.05 mmol) benzoic acid (76.0 mg, 0.63 mmol) and octylamine (206.0 µL, 1.25 mmol). 4 mg of the desired compound were obtained (12 %). **R*f*** (CH_2Cl_2/MeOH, 95:5): 0.19. **^{1}H NMR (500 MHz, CD_2Cl_2): δ** 8.39 (d, *J* = 8.4 Hz, 2H), 8.28 (t, *J* = 8.5 Hz, 1H), 8.09 – 7.85 (m, 4H), 7.69 (dd, *J* = 8.5, 5.4 Hz, 2H), 7.37 (t, *J* = 7.4 Hz, 2H), 7.17 (d, *J* = 8.2 Hz, 2H), 6.84 (t, *J* = 8.5 Hz, 2H), 4.99 – 4.79 (m, 2H), 4.70 – 4.51 (m, 2H), 2.48 – 2.04 (m, 4H), 1.82 – 1.67 (m, 2H), 1.60 – 1.59 (m, 1H), 1.42 (d, *J* = 6.2 Hz, 3H), 1.38 – 1.25 (m, 7H), 0.96 – 0.88 (m, 1H). **^{13}C NMR (125 MHz, CD_2Cl_2): δ** 142.5 (C+), 141.7 (C), 139.6 (CH), 139.5 (CH), 138.4 (C), 135.5 (CH), 129.7 (C), 129.3 (C), 129.0 (CH), 128.9 (CH), 128.3 (CH), 128.2 (CH), 127.9 (CH), 123.0 (CH), 122.9 (CH), 123.1 (CH), 121.9 (C), 116.6 (C), 115.3 (CH), 115.2 (CH), 106.9 (CH), 106.8 (CH), 52,2 (CH2), 50.8 (CH2), 32.1 (CH2), 30.1 (CH2), 29.6 (CH2), 27.4 (CH2), 27.2 (CH2), 23.0 (CH2), 20.9 (CH2), 14.3 (CH3), 11.3 (CH3). **^{19}F NMR (282 MHz, CD_2Cl_2): δ** -152.52, -152.57. **UV/VIS (CH_3CN, 2.10^{-5} M, λ_{max} (nm), (Log ε)):** 373 (4.32), 415 (3.84), 619 (4.23). **IR (neat, cm^{-1}): υ** 2927, 2862, 1610, 1573, 1338, 1261, 1210, 1162, 1055, 818, 755. **HRMS** (**ESI**) calculated for (M^+): 523.3108. Found: 523.3100.

7-isopropyl-11-propyl-7,11-dihydrobenzo[a]benzo[5,6]quinolino[2,3,4-kl]acridin-17c-ylium tetrafluoroborate **3k**

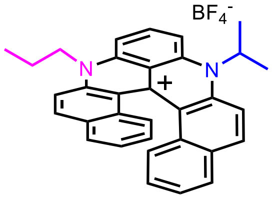

Azaoxa **2a** (24.0 mg, 0.05 mmol) benzoic acid (76.0 mg, 0.63 mmol) and isopropylamine (102.0 µL, 1.25 mmol) 10.3 mg of the desired compound were obtained (38 %). **R*f*** (CH_2Cl_2/MeOH, 95:5): 0.18. **^{1}H NMR (500 MHz, CD_2Cl_2): δ** 8.42 (d, *J* = 9.4 Hz, 1H), 8.35 (d, *J* = 9.4 Hz, 1H), 8.23 (t, *J* = 8.5 Hz, 1H), 8.13 (d, *J* = 9.5 Hz, 1H), 7.98 (d, *J* = 9.5 Hz, 1H), 7.92 (d, *J* = 6.7 Hz, 1H), 7.89 (d, *J* = 6.7 Hz, 1H), 7.82 (d, *J* = 8.4 Hz, 1H), 7.69 (d, *J* = 8.5 Hz, 1H), 7.41 (t, *J* = 6.9 Hz, 1H), 7.37 (t, *J* = 6.9 Hz, 1H),

7.32 (d, J = 8.5 Hz, 1H), 7.15 (d, J = 9.1 Hz, 1H), 6.89 (t, J = 8.5 Hz, 1H), 6.87 – 6.80 (m, 1H), 5.53 – 5.44 (m, 1H), 4.93 – 4.81 (m, 1H), 4.68 – 4.52 (m, 1H), 2.38 – 2.17 (m, 2H), 2.08 (d, J = 7.0 Hz, 3H), 1.98 (d, J = 7.0 Hz, 3H), 1.33 (t, J = 7.3 Hz, 3H). **^{13}C NMR (125 MHz, CD_2Cl_2) δ** 143.3 (C+), 142.5 (C), 142.0 (C), 139.9 (CH), 139.7 (CH), 138.9 (CH), 138.4 (C), 138.0 (C), 135.7 (C), 134.9 (CH), 129.9 (C), 129.8 (C), 129.3 (CH), 129.0 (C), 128.6 (CH), 128.4 (CH), 128.2 (CH), 127.9 (CH), 123.6 (CH), 123.0 (CH), 122.9 (C), 118.3 (C), 117.2 (C), 116.5 (CH), 115.5 (CH), 109.1 (CH), 107.1 (CH), 57.2 (CH), 21.8 (CH3), 21.2 (CH2), 21.1 (CH2), 20.7 (CH3), 11.5 (CH3). **^{19}F NMR (282 MHz, CD_2Cl_2): δ** -152.27, -152.32. **UV/VIS (CH_3CN, 2.10^{-5} M, λ_{max} (nm), (Log ε)):** 372 (4.20), 417 (3.81), 620 (4.12). **IR (neat, cm^{-1}): υ** 3607, 2928, 1609, 1573, 1522, 1484, 1334, 1257, 1212, 1159, 1055, 812, 784, 757. **HRMS** (**ESI**) calculated for (M^+): 453.2325. Found: 453.2323.

<u>7-allyl-11-propyl-7,11-dihydro-17cH-benzo[a]benzo[5,6]quinolino[2,3,4-kl]acridin-17c-ylium tetrafluoroborate **3l**</u>

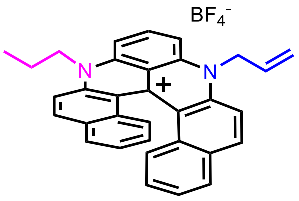

Azaoxa **2a** (31.9 mg, 0.06 mmol), benzoic acid (91 mg, 0.75 mmol) and allylamine (120 µL, 1.5 mmol) 5.5 mg of the desired compound were obtained (17 %). **R*f*** (CH_2Cl_2/MeOH, 95:5): 0.24. **^{1}H NMR (500 MHz, CD_2Cl_2): δ** 8.42 (d, J = 9.5 Hz, 1H), 8.35 (d, J = 9.5 Hz, 1H), 8.25 (t, J = 8.5 Hz, 1H), 7.98 (d, J = 9.5 Hz, 1H), 7.94 – 7.82 (m, 3H), 7.71 (d, J = 8.5 Hz, 1H), 7.63 (d, J = 8.5 Hz, 1H), 7.38 (m, 2H), 7.25 (d, J = 8.5 Hz, 1H), 7.17 (d, J = 8.6 Hz, 1H), 6.85 (t, J = 8.6 Hz, 2H), 6.46 – 6.30 (m, 1H), 5.71 – 5.45 (m, 2H), 5.27 – 5.08 (m, 2H), 5.04 – 4.78 (m, 1H), 4.71 – 4.52 (m, 1H), 2.35 – 2.11 (m, 2H), 1.33 (t, J = 7.4 Hz, 3H).

^{13}C NMR (125 MHz, CD_2Cl_2): δ 142.96(C+), 142.64(C), 141.94(C), 139.75(CH), 139.57(CH), 138.60(C), 138.07(C), 135.62(CH), 129.88(C), 129.67(C), 129.30(CH), 129.27(C), 129.09(CH), 129.02(CH), 129.00(C), 128.30(CH), 128.21(CH), 127.95(CH), 127.74(CH), 123.26(CH), 123.06(CH), 121.91(C), 118.82(CH2), 116.85(C), 116.64(C), 115.94(CH), 115.31(CH), 107.55(CH), 107.14(CH), 52.2 (CH2), 52.1 (CH2), 30.09(CH2), 21.02(CH2), 11.30(CH3). **^{19}F NMR (282 MHz, CD_2Cl_2): δ** -152.38, -152.43. **UV/VIS (CH_3CN, 2.10^{-5} M, λ_{max} (nm), (Log ε)):** 368 (4.33), 410 (3.71), 613 (4.18). **IR (neat, cm^{-1}): υ** 1613, 1570, 1550, 1520, 1488, 1331, 1264, 1213, 1170, 1056, 819, 757, 690, 621. **HRMS** (**ESI**) calculated for (M^+): 451.2186. Found: 451.2169.

<u>7-(5-hydroxypentyl)-11-propyl-7,11-dihydro-17cH-benzo[a]benzo[5,6]quinolino[2,3,4-kl]acridin-17c-ylium tetrafluoroborate **3m**</u>

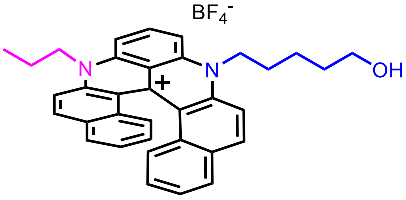

Azaoxa **2a** (31.1 mg, 0.06 mmol) benzoic acid (91 mg, 0.75 mmol) and 5-amino-1-pentanol (160 µL, 1.5 mmol). 9.1 mg of the desired compound were obtained (26 %). **R*f*** (CH_2Cl_2/MeOH, 95:5): 0.20 **^{1}H NMR (500 MHz, CD_2Cl_2): δ** 8.40 (d, J = 6.3 Hz, 1H), 8.38 (d, J = 6.3 Hz, 1H), 8.29 (t, J = 8.5 Hz, 1H), 8.02 (d, J = 9.5 Hz, 1H), 7.96 (d, J = 9.5 Hz, 1H), 7.88 (d, J = 7.8 Hz, 2H), 7.74 (d, J = 8.6 Hz, 1H), 7.68 (d, J = 8.5 Hz, 1H), 7.35 (dd, J = 8.1, 6.9 Hz, 2H), 7.16 (d, J = 8.5 Hz, 2H), 6.89 – 6.77 (m, 2H), 5.06 – 4.74 (m, 2H), 4.74 – 4.17 (m, 2H), 2.58 – 1.94 (m, 2H), 1.91 – 1.61 (m, 4H), 1.32 (t, J = 7.4 Hz, 3H). **^{13}C NMR (125 MHz, CD_2Cl_2): δ** 142.17(C+), 142.10(C), 141.28(C), 139.17(CH), 139.01(CH), 137.95(C), 135.19(CH), 129.28(C), 129.24(C), 128.90(C), 128.88(C), 128.57(CH), 128.52(CH), 127.75(CH), 127.69(CH), 127.33(CH), 122.64(CH), 122.61(CH),

121.47(C), 116.18(C), 116.14(C), 115.05(CH), 114.91(CH), 106.65(CH), 106.48(CH), 62.17(CH2), 51.71(CH2), 50.40(CH2), 32.00(CH2), 26.72(CH2), 23.28(CH2), 20.48(CH2), 10.87(CH3). **^{19}F NMR (282 MHz, CD_2Cl_2): δ** -152.11, -152.16. **UV/VIS (CH_3CN, 2.10^{-5} M, λ_{max} (nm), (Log ε)):** 263 (4.46), 369 (4.19), 614 (4.04). **IR (neat, cm^{-1}): υ** 3558, 3413, 2933, 2877, 1611, 1573, 1548, 1523, 1490, 1439, 1386, 1338, 1261, 1215, 1181, 1162, 1057, 873, 817, 784, 755, 539. **HRMS** (**ESI**) calculated for (M^+): 497.2586. Found: 497.2587.

7-(2-(2-hydroxyethoxy)ethyl)-11-propyl-7,11-dihydro-17cH-benzo[a]benzo[5,6]quinolino[2,3,4-kl]acridin-17c-ylium tetrafluoroborate **3n**

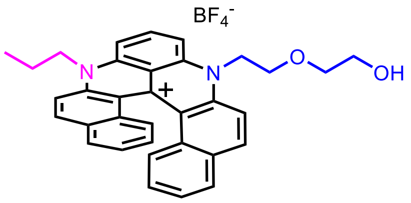

Azaoxa **2a** (31.2 mg, 0.06 mmol) benzoic acid (91 mg, 0.75 mmol) and 2-(2-Aminoethoxy) ethanol (150 µL, 1.5 mmol) 7.2 mg of the desired compound were obtained (19 %). **R*f*** (CH_2Cl_2/MeOH, 95:5): 0.20. **^{1}H NMR (500 MHz, CD_2Cl_2): δ** 8.38 (d, *J* = 9.5 Hz, 2H), 8.28 (t, *J* = 8.5 Hz, 1H), 8.15 (d, *J* = 9.5 Hz, 1H), 7.94 (t, *J* = 9.0, 2H), 7.88 (d, *J* = 8.0 Hz, 2H), 7.69 (d, *J* = 8.5 Hz, 1H), 7.41 – 7.31 (m, 2H), 7.15 (d, *J* = 8.5 Hz, 2H), 6.86 – 6.78 (m, 2H), 5.25 – 5.15 (m, 1H), 5.01 – 4.92 (m, 1H), 4.90 – 4.81 (m, 1H), 4.64 – 4.53 (m, 1H), 4.40 – 4.25 (m, 2H), 3.77 – 3.61 (m, 4H), 2.31 – 2.14 (m, 2H), 1.32 (t, *J* = 7.4 Hz, 3H). **^{13}C NMR (125 MHz, CD_2Cl_2): δ** 143.3(C+), 142.6(C), 142.0(C), 139.5(CH), 139.2(CH), 139.0(C), 138.2(C), 135.5(CH), 129.8(C), 129.6(C), 129.3(C), 129.2(C), 128.9(CH), 128.9(CH), 128.2(CH), 128.1(CH), 127.8(CH), 127.7(CH), 123.1(CH), 123.0(CH), 121.9(C), 116.6(CH), 116.1(C), 115.3(CH), 115.1 (C), 107.9(CH), 107.1(CH), 73.5(CH2), 68.5(CH2), 62.1(CH2), 52.2(CH2), 50.6(CH2), 20.9(CH2), 11.3(CH3). **^{19}F NMR (282 MHz, CD_2Cl_2): δ** -152.18, -152.23. **UV/VIS (CH_3CN, 2.10^{-5} M, λ_{max} (nm), (Log ε)):** 368 (4.28), 410 (3.65), 612 (4.13). **IR (neat, cm^{-1}): υ** 2968, 2926, 2870, 1610, 1570, 1548, 1522, 1492, 1460, 1333, 1262, 1211, 1178, 1159, 1049, 882, 819, 781, 731, 699, 544. **HRMS** (**ESI**) calculated for (M^+): 499.2375 Found: 499.2380.

7-(2-(2-hydroxyethoxy)ethyl)-11-isopropyl-7,11-dihydro-17cH-benzo[a]benzo[5,6]quinolino[2,3,4-kl]acridin-17c-ylium tetrafluoroborate **3o**

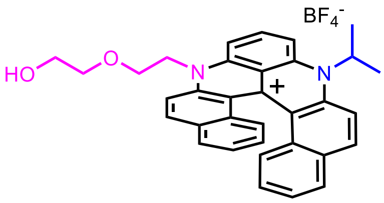

Azaoxa **2i** (25.0 mg, 0.05 mmol) benzoic acid (76 mg, 0.625 mmol) and isopropylamine (106 µL, 1.25 mmol) 6.7 mg of the desired compound were obtained (23 %). **R*f*** (CH_2Cl_2/MeOH, 95:5): 0.18. **^{1}H NMR (500 MHz, CD_2Cl_2): δ** .45 (d, *J* = 9.5 Hz, 1H), 8.38 (d, *J* = 9.5 Hz, 1H), 8.31 – 8.23 (m, 2H), 8.16 (d, *J* = 9.4 Hz, 1H), 7.98 (d, *J* = 8.5 Hz, 1H), 7.95 (d, *J* = 8.0 Hz, 1H), 7.91 (d, *J* = 8.0 Hz, 1H), 7.86 (d, *J* = 8.5 Hz, 1H), 7.41 (m, 2H), 7.34 (d, *J* = 8.5 Hz, 1H), 7.17 (d, *J* = 8.5 Hz, 1H), 6.97 – 6.81 (m, 2H), 5.51 (m, 1H), 5.29 (m, 1H), 5.05 (m, 1H), 4.44 – 4.29 (m, 2H), 3.78 – 3.64 (m, 4H), 2.11 (d, *J* = 7.0 Hz, 3H), 2.01 (d, *J* = 7.0 Hz, 3H). **^{13}C NMR (125 MHz, CD_2Cl_2): δ** 143.2(C+), 143.1(C), 142.1(C), 139.4(CH), 138.7(CH), 138.4(C), 138.1(C), 134.7(CH), 129.8(C), 129.6(C), 129.2(CH), 129.1(CH), 129.0(CH), 128.9(C), 128.8(C), 128.2(CH), 127.9(CH), 127.8 (CH), 127.7(CH), 123.4(CH), 123.0(CH), 122.8(C), 118.8(C), 117.1(C), 116.3(CH), 109.0(CH), 107.9(CH), 73.6(CH2), 68.6(CH2), 62.1(CH2), 57.1(CH), 50.9(CH2), 21.7(CH3), 20.5(CH3). **^{19}F NMR (282 MHz, CD_2Cl_2): δ** -152.35, -152.40. **UV/VIS (CH_3CN, 2.10^{-5} M, λ_{max} (nm), (Log ε)):** 369 (4.29), 411 (3.80), 614 (4.14). **IR (neat, cm^{-1}): υ** 2922, 2849, 1668, 1608, 1570, 1546, 1522,

1492, 1458, 1374, 1333, 1260, 1211, 1161, 1120, 1060, 897, 869, 817, 785, 759, 733, 699, 628, 568. **HRMS** (**ESI**) calculated for (M^+): 499.2380. Found: 499.2379.

7-benzyl-11-(5-hydroxypentyl)-7,11-dihydro-17cH-benzo[a]benzo[5,6]quinolino[2,3,4-kl]acridin-17c-ylium tetrafluoroborate **3p**

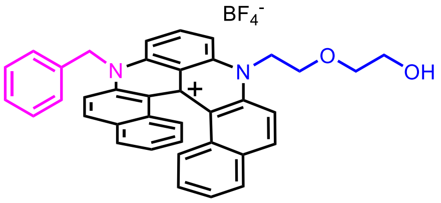

Azaoxa **2d** (48.7 mg, 0.1 mmol) benzoic acid (162 mg, 12.5 mmol) and 5-amino-1-pentanol (290 µL, 2.5 mmol) 15.8 mg of the desired compound were obtained (25 %). **R*f*** (CH_2Cl_2/MeOH, 95:5): 0.22. **^{1}H NMR (500 MHz, CD_2Cl_2): δ** 8.45 (d, *J* = 9.4 Hz, 1H), 8.26 (d, *J* = 9.2 Hz, 1H), 8.17 (t, *J* = 8.5 Hz, 1H), 8.06 (d, *J* = 9.5 Hz, 1H), 7.93 (d, *J* = 8.0 Hz, 1H), 7.86 (d, *J* = 7.2 Hz, 1H), 7.76 (dd, *J* = 9.0, 5.0 Hz, 2H), 7.50 (d, *J* = 8.4 Hz, 1H), 7.47 – 7.34 (m, 5H), 7.32 – 7.26 (m, 3H), 7.20 (d, *J* = 9.1 Hz, 1H), 6.86 (t, *J* = 8.5 Hz, 2H), 6.20 (d, *J* = 18.1 Hz, 1H), 5.81 (d, *J* = 18.2 Hz, 1H), 5.07 – 4.90 (m, 1H), 4.74 – 4.65 (m, 1H), 3.75 (t, *J* = 5.8 Hz, 2H), 2.37 – 2.15 (m, 2H), 1.93 – 1.74 (m, 4H). **^{13}C NMR (125 MHz, CD_2Cl_2): δ** 143.1(C+), 142.8(C), 142.0(C), 140.0(CH), 139.6(CH), 138.9(C), 138.1(C), 135.7(CH), 133.5(C), 130.0(CH), 129.9(C), 129.7(C), 129.3(C), 129.0(CH), 129.0(CH), 128.9(CH), 128.4(CH), 128.3(CH), 128.0(CH), 127.8(CH), 126.2(CH), 126.1(C), 123.3(CH), 123.1(CH), 122.0(C), 117.1(C), 116.7(C), 115.8(CH), 115.4(CH), 107.6(CH), 107.4(CH), 62.6(CH2), 55.2(CH2), 51.0(CH2), 32.4(CH2), 27.3(CH2), 23.7(CH2). **^{19}F NMR (282 MHz, CD_2Cl_2): δ** -152.27, -152.24. **UV/VIS (CH_3CN, 2.10^{-5} M, λ_{max} (nm), (Log ε)):** 368 (3.94), 409 (3.41), 613 (3.81). **IR (neat, cm^{-1}): υ** 3056, 2926, 2849, 1613, 1574, 1548, 1518, 1494, 1456, 1441, 1335, 1262, 1208, 1178, 1159, 1056, 1032, 897, 869, 815, 778, 731, 697, 587, 529. **HRMS** (**ESI**) calculated for (M^+): 545.2587. Found: 545.2589.

Synthesis of trapped intermediates 4 and **6**

13-propyl-12-(trifluoromethyl)-12,13-dihydro-12,19c-epoxybenzo[7,8]xantheno[9,1-fg]naphtho[2,1-d][1,3]oxazocine **4.**

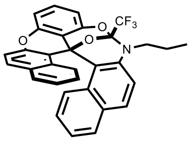

To a solution of dioxa[6]helicene **1** (92 mg, 0.20 mmol) in acetonitrile (2 mL) under nitrogen atmosphere was added *n*-propylamine (50 µL, 0.60 mmol, 3 equiv.). This reaction mixture was stirred 30 min at 20 °C while gradually evolving from a red color to a deep blue color. After this time, trifluoroacetyl chloride solution (4.3 M in dichloromethane, 560 µL, 2.40 mmol, 12 equiv.) was added to the reaction mixture, which immediately turned brown and was further stirred at 20 °C for 16 hours. After this time, the reaction mixture was diluted with dichloromethane, washed with a 1 M aqueous $NaBF_4$ solution (once) and a saturated aqueous NaCl solution (twice). The organic layer was dried over Na_2SO_4, filtered and evaporated. The crude material was purified by flash chromatography on silica gel (cyclohexane / EtOAc, from 100 : 00 to 80 : 20) affording 102 mg of the title compound as an off-white solid in *ca.* 90% purity. Second flash chromatography on silica gel (pentane / Et_2O, from 100 : 00 to 90 : 10) afforded 60 mg of the title compound as a white solid. Slow evaporation of a dichloromethane solution of **4** led to the formation of white crystals. (57 % yield). **MP** 173-175 °C. **Rf:** (pentane / Et_2O 90 : 10): 0.48. **^{1}H NMR (500 MHz, CD_2Cl_2)** δ 8.01 (dd, *J* = 8.0, 1.0 Hz, 1H), 7.88 (dd, *J* = 9.0, 0.7 Hz, 1H), 7.78 (dd, *J* = 10.0, 1.0 Hz, 1H), 7.75

– 7.66 (m, 2H), 7.63 – 7.55 (m, 1H), 7.50 (d, *J* = 9.0 Hz, 1H), 7.41 (d, *J* = 9.2 Hz, 1H), 7.39 – 7.30 (m, 2H), 7.29 – 7.18 (m, 2H), 7.12 – 7.04 (m, 3H), 6.86 (dd, *J* = 8.1, 0.9 Hz, 1H), 4.06 (ddd, *J* = 16.5, 12.0, 4.9 Hz, 1H), 3.68 – 3.55 (m, 1H), 2.12 – 1.99 (m, 1H), 1.93 (tdd, *J* = 12.4, 7.4, 4.9 Hz, 1H), 1.09 (t, *J* = 7.4 Hz, 3H). **^{13}C NMR (126 MHz, CD_2Cl_2)** δ 152.3 (C), 151.3 (C), 147.9 (C), 139.2 (C), 132.6 (CH), 131.6 (C), 131.1 (C), 131.0 (CH), 130.6 (C), 129.9 (CH), 129.3 (C), 129.0 (CH), 128.8 (CH), 127.2 (CH), 126.9 (CH), 125.4 (CH), 124.9 (CH), 123.5 (CH), 122.3 (C), 121.8 (CH), 120.0 (C), 118.9 (C), 117.8 (CH), 115.3 (CH), 114.5 (C), 113.0 (C), 110.5 (CH), 109.6 (CH), 102.3 (q, *J* = 34.0 Hz, CF_3), 71.4 (C), 49.1 (CH_2), 22.5 (CH_2), 11.4 (CH_3). **^{19}F NMR (282 MHz, CD_2Cl_2)** δ -78.62. **IR (neat, cm^{-1}) υ** 3327, 3059, 2966, 2931, 2876, 2255, 2032, 1944, 1694, 1620, 1559, 1480, 1450, 1390, 1363, 1304, 1210, 1139, 1093, 1018, 946, 928, 887, 866, 817, 748, 714, 624, 602. **HRMS** (**ESI**) (M^+) calculated for ($C_{32}H_{22}F_3NO_3$) 526.1630. Found: 526.1625.

11-((4-chlorobenzoyl)oxy)-7-propyl-12-(2-(propylamino)naphthalen-1-yl)-7,12-dihydrobenzo[a]acridin-12-ylium tetrafluoroborate **6**.

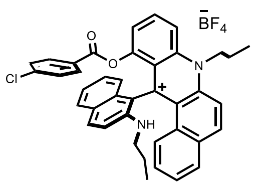

To a solution of azaoxa [6]helicene **2a** (25 mg, 0.05 mmol) in acetonitrile (1 mL) under N_2 atmosphere was added *n*-propylamine (100 µL, 1.25 mmol, 25 equiv.). The reaction mixture was stirred at 20 °C for 2 hours. After this time, the reaction mixture was evaporated to dryness using Schlenk technics. The residue was then dissolved in dry dichloromethane (1.5 mL) under N_2 atmosphere and 4-dimethylaminopyridine (DMAP, 30 mg, 0.25 mmol, 5 equiv.) and then *para*-chlorobenzoyl chloride (44 mg, 0.25 mmol, 5 equiv.) were added to this solution. The reaction mixture was stirred for 30 minutes at 20 °C. The reaction mixture was then diluted with dichloromethane and washed (3 x) with a 1 M aq. HBF_4 solution. The organic layer was then dried over Na_2SO_4, filtered and evaporated. The residue was dissolved in dichloromethane (5 mL) and precipitated with a 1:1 mixture of diethyl ether and pentane (40 mL). The precipitate was separated from the mother liquor by centrifugation. The residue was next purified by flash column chromatography (SiO_2, 10 x 1 cm, CH_2Cl_2/MeOH from 100:00 to 98.5:01.5). The title compound was isolated as a dark green solid (yellow-green in dichloromethane solutions). Diffusion of toluene in a dichloromethane solution of **6** led the formation of crystals. 22 mg, 0.0316 mmol, 63% yield. **MP** 151-153 °C. **Rf:** CH_2Cl_2/MeOH 95:05): 0.14. **^{1}H NMR (500 MHz, CD_2Cl_2) δ** 8.61 (t, *J* = 8.7 Hz, 2H), 8.44 (dd, *J* = 9.2, 7.6 Hz, 1H), 8.30 (d, *J* = 9.7 Hz, 1H), 8.02 (dd, *J* = 7.7, 1.5 Hz, 1H), 7.66 – 7.53 (m, 3H), 7.49 (dd, *J* = 9.1, 0.8 Hz, 1H), 7.36 – 7.27 (m, 3H), 7.21 (td, *J* = 6.5, 5.8, 2.3 Hz, 3H), 7.15 (t, *J* = 7.8 Hz, 1H), 7.08 (ddd, *J* = 8.8, 7.1, 1.6 Hz, 1H), 6.88 (d, *J* = 9.2 Hz, 1H), 6.78 (d, *J* = 8.5 Hz, 1H), 5.49 – 5.41 (m, 2H), 3.72 (brs, 1H), 3.13 – 2.79 (m, 2H), 2.65 – 2.47 (m, 2H), 1.53 (t, *J* = 7.4 Hz, 3H), 1.31 – 1.13 (m, 2H), 0.61 (t, *J* = 7.4 Hz, 3H). **^{13}C NMR (126 MHz, CD_2 Cl_2) δ** 164.1 (C), 153.3 (C), 150.2 (C), 146.4 (C), 144.7 (CH), 141.0 (C), 140.7 (C), 137.3 (CH), 132.8 (C), 132.3 (CH), 132.0 (CH), 131.9 (C), 130.7 (CH), 130.5 (CH), 130.4 (CH), 130.1 (C), 129.2 (CH), 128.9 (CH), 128.5 (C), 128.4 (CH), 128.2 (CH), 128.0 (C), 126.7 (C), 124.2 (CH), 124.0 (C), 123.3 (CH), 122.4 (CH), 117.6 (CH), 116.4 (CH), 115.3 (CH), 114.7 (C), 55.9 (CH_2), 46.0 (CH_2), 23.2 (CH_2), 23.2 (CH_2), 11.6 (CH_3), 11.5 (CH_3). **^{19}F NMR (282 MHz, CD_2Cl_2) δ** -153.13, -153.18. **HRMS** (**ESI**) (M^+) calculated for ($C_{40}H_{34}ClN_2O_2$): 609.2303. Found: 609.2307.

Isolation of side product **5**

14-(2,6-dihydroxyphenyl)-7-propyl-7,14-dihydrodibenzo[a,j]acridin-14-ylium tetrafluoroborate **5**.

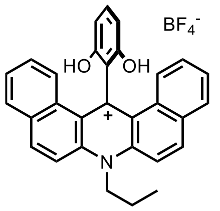

Dibenzoacridinium are formed during reactions of transformation of dioxa **1** to azaoxa derivatives of type **2**. For instance, during the formation of **2a**, **5** can be isolated in 10 to 30% yield as a yellow solid. **Rf:** CH_2Cl_2/MeOH 95:05): 0.09. **^{1}H NMR (500 MHz, CD_3OD) δ** 8.57 (d, *J* = 9.5 Hz, 2H), 8.39 (d, *J* = 9.6 Hz, 2H), 8.12 (dd, *J* = 7.8, 1.4 Hz, 2H), 7.97 (dd, *J* = 8.6, 0.9 Hz, 2H), 7.71 (ddd, *J* = 7.9, 7.0, 1.0 Hz, 2H), 7.46 (t, *J* = 8.3 Hz, 1H), 7.34 (ddd, *J* = 8.6, 7.0, 1.5 Hz, 2H), 6.60 (d, *J* = 8.3 Hz, 2H), 5.38 (t, *J* = 7.4 Hz, 2H), 2.30 (dt, *J* = 7.4, 7.4 Hz, 2H), 1.32 (t, *J* = 7.4 Hz, 3H). **^{13}C NMR (126 MHz, CD_3OD) δ** 156.0 (2 C), 151.1 (C), 142.6 (2 C), 141.5 (2 C*H*), 134.0 (C*H*), 133.2 (2 C), 131.7 (2 C), 130.4 (2 C*H*), 130.0 (2 C*H*), 129.2 (2 C*H*), 128.1 (2 C*H*), 127.4 (2 C), 117.6 (C), 116.6 (2 C*H*), 109.8 (2 C*H*), 54.7 (C*H₂*), 23.8 (C*H₂*), 11.0 (C*H₃*). **^{19}F NMR (282 MHz, CD_3OD) δ** -152.74, -152.79. **UV/VIS (CH_3CN, 4.3 10^{-4} M, λ_{max} (nm), (Log ε)):** 305 (3.71), 420 (3.25), 444 (3.34). **IR (neat, cm^{-1}) υ** 3382, 3140, 2931, 1660, 1615, 1600, 1570, 1555, 1526, 1466, 1367, 1313, 1271, 1226, 1201, 1160, 1054, 1026, 993, 927, 868, 825, 796, 753, 728, 620. **HRMS** (**ESI**) (M^+) calculated for ($C_{30}H_{24}NO_2$): 430.1802. Found: 430.1815.

9. Crystallographic data

Compound **4**

Table S4. Crystal data and structure refinement for **4**.

**CCDC 1908256**		
Empirical formula	C32 H22 F3 N O3	
Formula weight	525.50	
Temperature	179.95(10) K	
Wavelength	1.54184 Å	
Crystal system	Orthorhombic	
Space group	Pccn	
Unit cell dimensions	a = 8.96089(13) Å	α= 90°
	b = 35.7856(6) Å	β= 90°
	c = 14.92847(19) Å	γ = 90°
Volume	4787.13(12) $Å^3$	
Z	8	
Density (calculated)	1.458 Mg/m^3	
Absorption coefficient	0.914 mm^{-1}	
F(000)	2176	
Crystal size	0.631 x 0.25 x 0.05 mm^3	
Theta range for data collection	4.943 to 73.338°.	
Index ranges	-11<=h<=10, -44<=k<=43, -11<=l<=18	
Reflections collected	34874	
Independent reflections	4778 [R(int) = 0.0437]	
Completeness to theta = 67.684°	100.0 %	
Absorption correction	Gaussian	
Max. and min. transmission	0.955 and 0.691	
Refinement method	Full-matrix least-squares on F^2	
Data / restraints / parameters	4778 / 0 / 353	
Goodness-of-fit on F^2	1.048	
Final R indices [I>2sigma(I)]	R1 = 0.0369, wR2 = 0.0899	
R indices (all data)	R1 = 0.0415, wR2 = 0.0938	
Extinction coefficient	n/a	
Largest diff. peak and hole	0.170 and -0.234 e.$Å^{-3}$	

Figure S38. View of the asymmetric unit of **4**, with displacement ellipsoids depicted at 50 percent probability level.

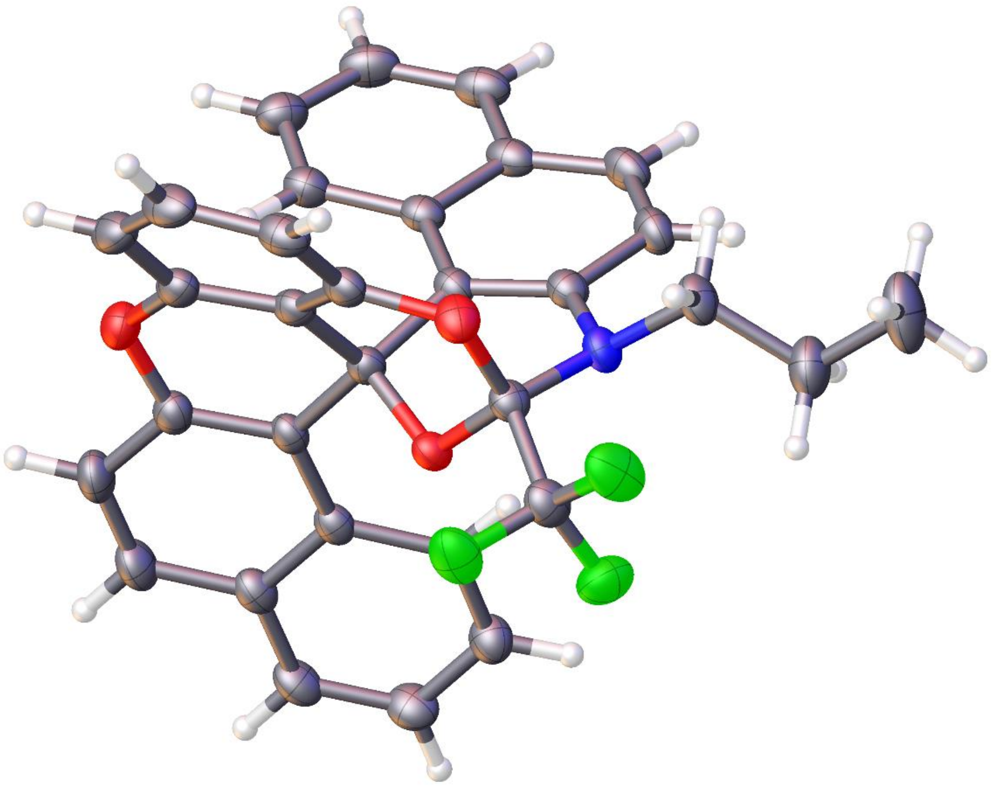

Compound **6**

Table S5. Crystal data and structure refinement for **6**.

**CCDC 1908257**		
Empirical formula	C40 H34 B Cl F4 N2 O2	
Formula weight	696.95	
Temperature	180.01(10) K	
Wavelength	1.54184 Å	
Crystal system	Triclinic	
Space group	P-1	
Unit cell dimensions	a = 7.9096(4) Å	α= 86.244(5)°
	b = 13.4176(8) Å	β= 78.883(5)°
	c = 16.0932(10) Å	γ = 89.014(5)°
Volume	1672.26(17) Å^3	
Z	2	
Density (calculated)	1.384 Mg/m^3	
Absorption coefficient	1.536 mm^{-1}	
F(000)	724	
Crystal size	0.431 x 0.063 x 0.014 mm^3	
Theta range for data collection	3.301 to 73.866°.	
Index ranges	-9<=h<=6, -14<=k<=16, -19<=l<=19	
Reflections collected	10264	
Independent reflections	6353 [R(int) = 0.0488]	
Completeness to theta = 67.684°	97.2 %	
Absorption correction	Gaussian	
Max. and min. transmission	0.979 and 0.734	
Refinement method	Full-matrix least-squares on F^2	
Data / restraints / parameters	6353 / 1 / 454	
Goodness-of-fit on F^2	1.097	
Final R indices [I>2sigma(I)]	R1 = 0.0721, wR2 = 0.1638	
R indices (all data)	R1 = 0.1321, wR2 = 0.2344	
Extinction coefficient	0.0013(3)	
Largest diff. peak and hole	0.437 and -0.420 e.Å^{-3}	

Figure S39. View of the asymmetric unit of **6**, with displacement ellipsoids depicted at 50 percent probability level.

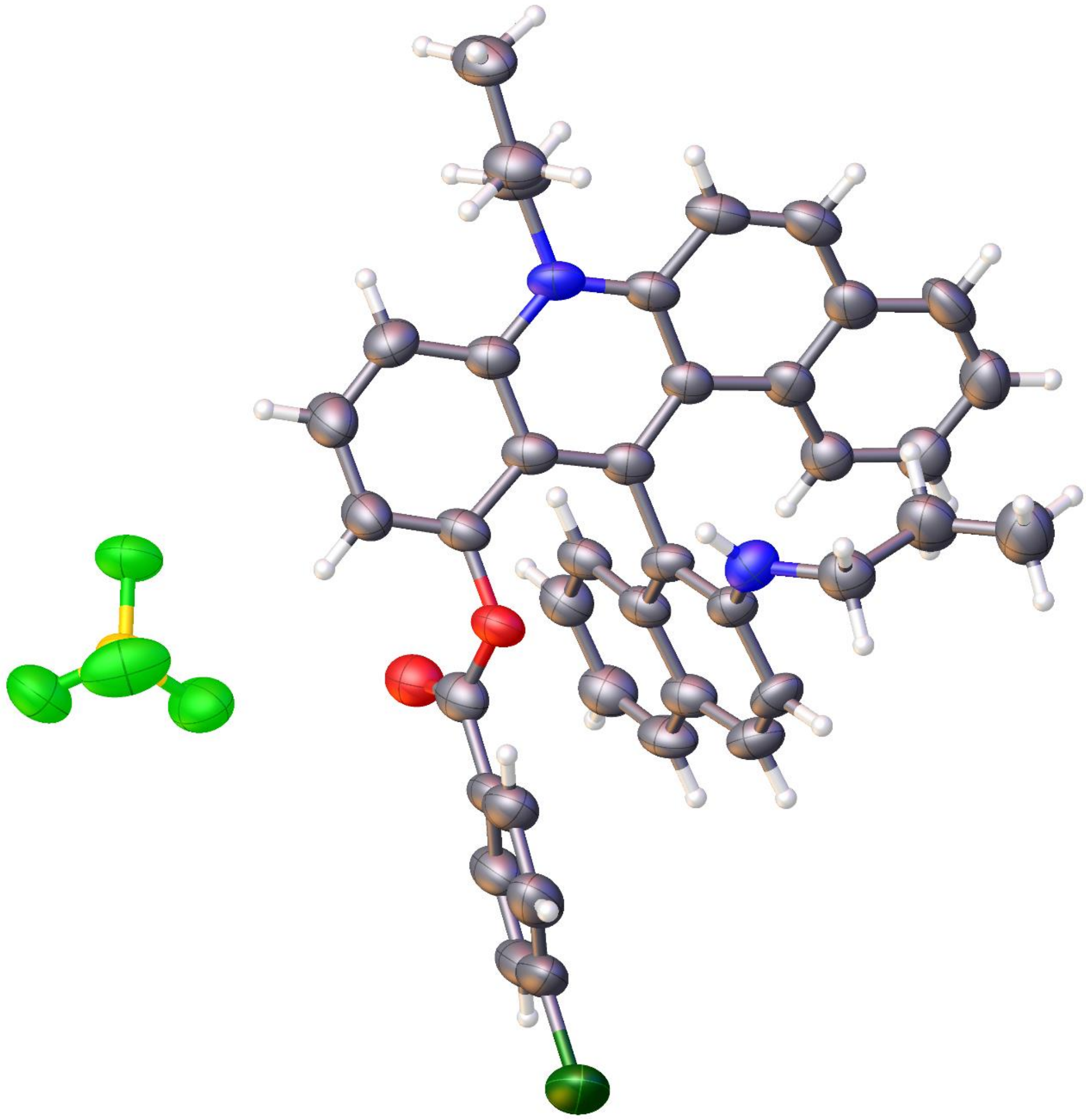

10. ^{1}H NMR, ^{13}C NMR, ^{19}F NMR and UV/Visible spectra

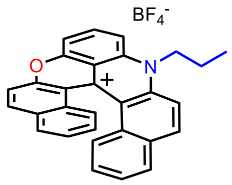

11-propylbenzo[a]benzo[5,6]chromeno[2,3,4-kl]acridin-17c(11H)-ylium tetrafluoroborate **2a**

^{1}H NMR (500 MHz, CD_2Cl_2) of **2a**

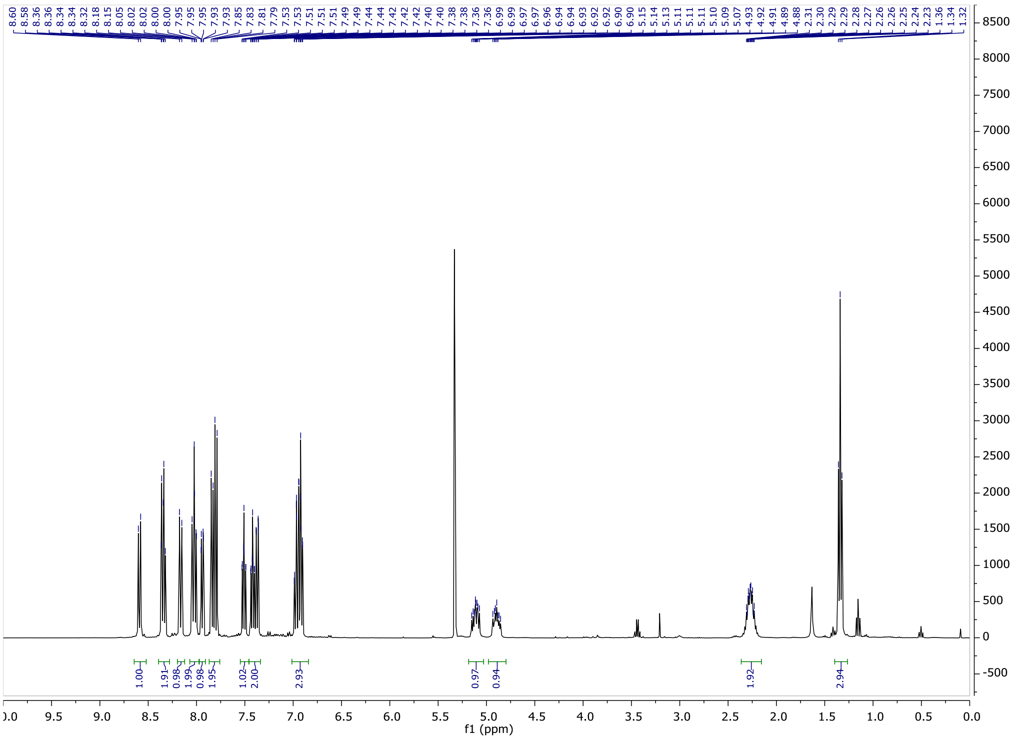

^{13}C NMR (125 MHz, CD_2Cl_2) of **2a**

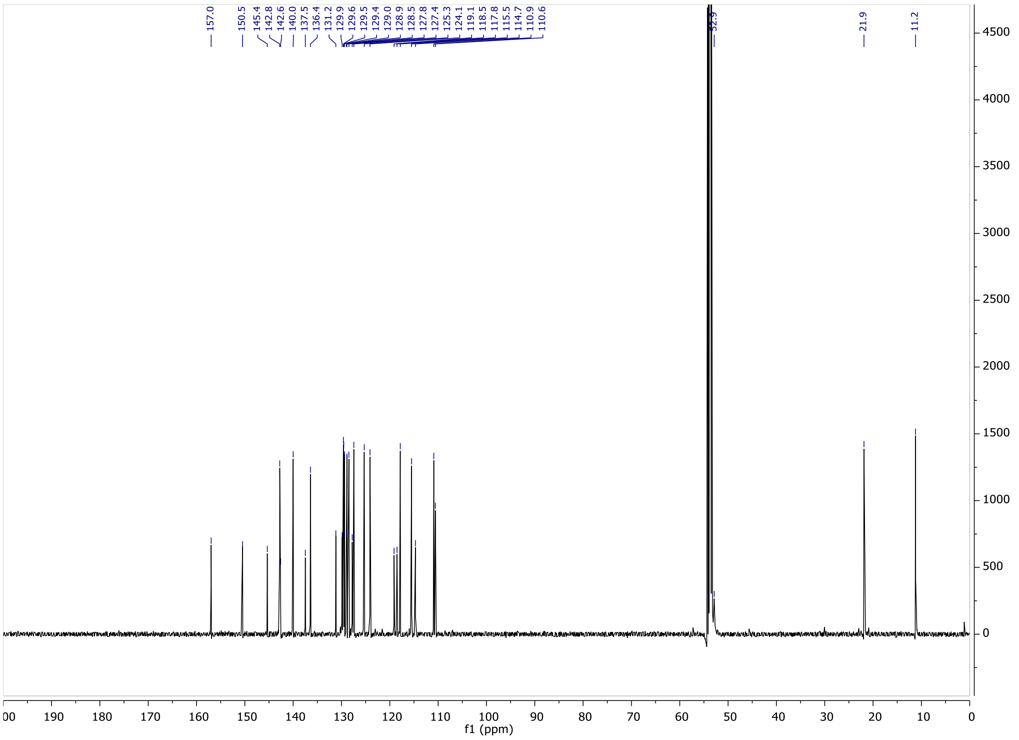

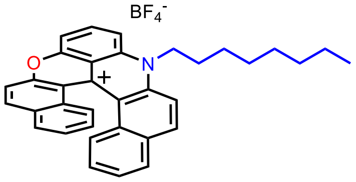

11-octyl-11H-benzo[a]benzo[5,6]chromeno[2,3,4-kl]acridin-17c-ylium tetrafluoroborate **2b**

^{1}H NMR (500 MHz, CD_2Cl_2) of **2b**

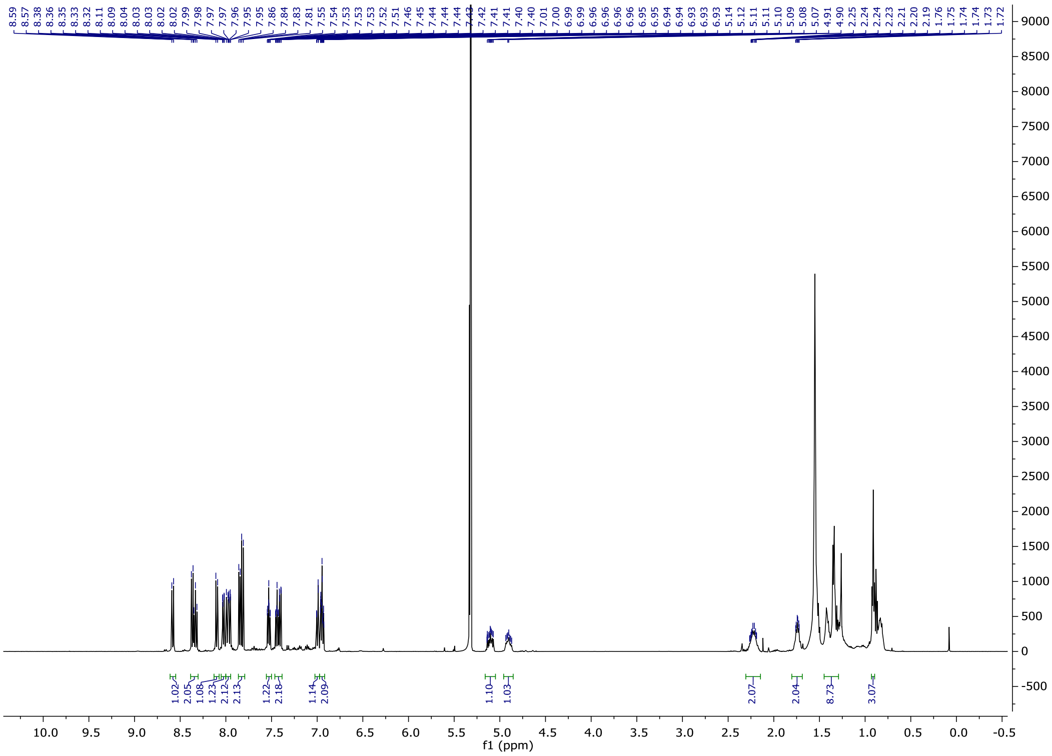

^{13}C NMR (125 MHz, CD_2Cl_2) of **2b**

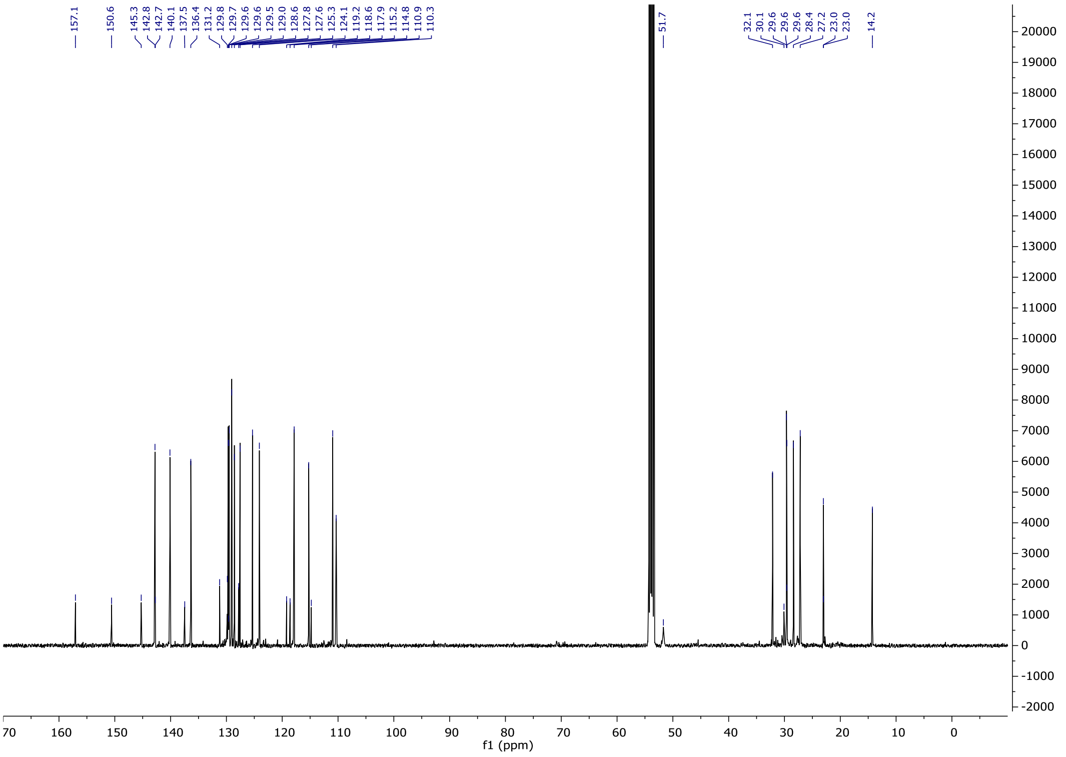

^{19}F NMR (282 MHz, CD_2Cl_2) of **2b**

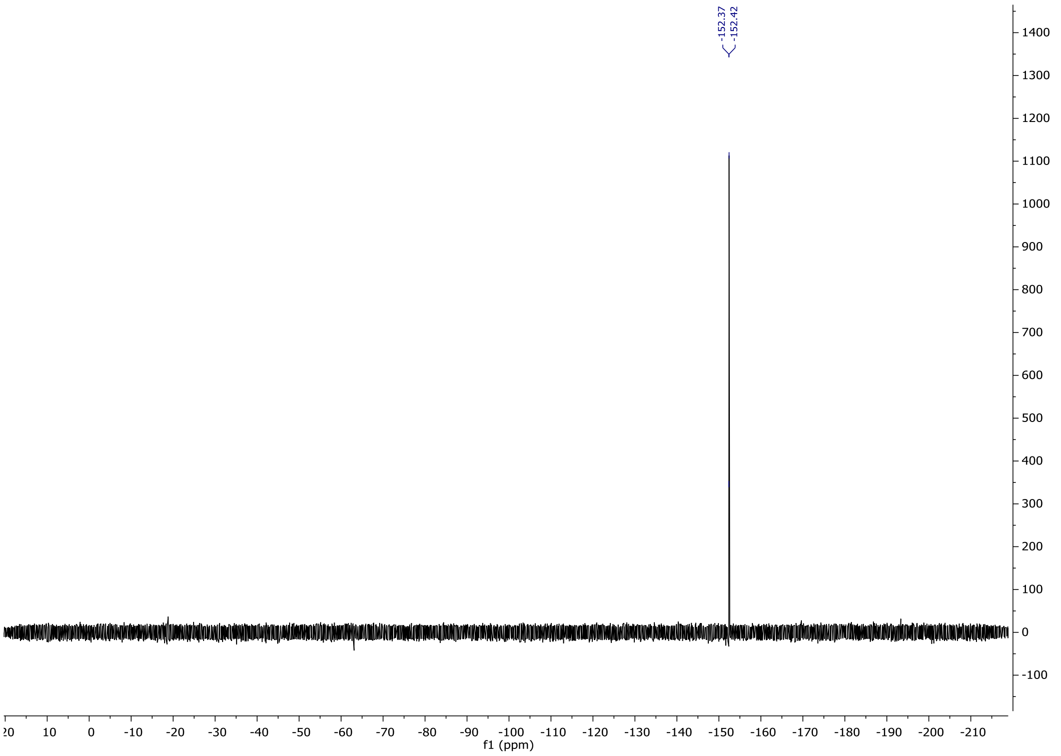

UV/VIS spectra recorded in CH_3CN (2.10^{-5} M) of **2b**

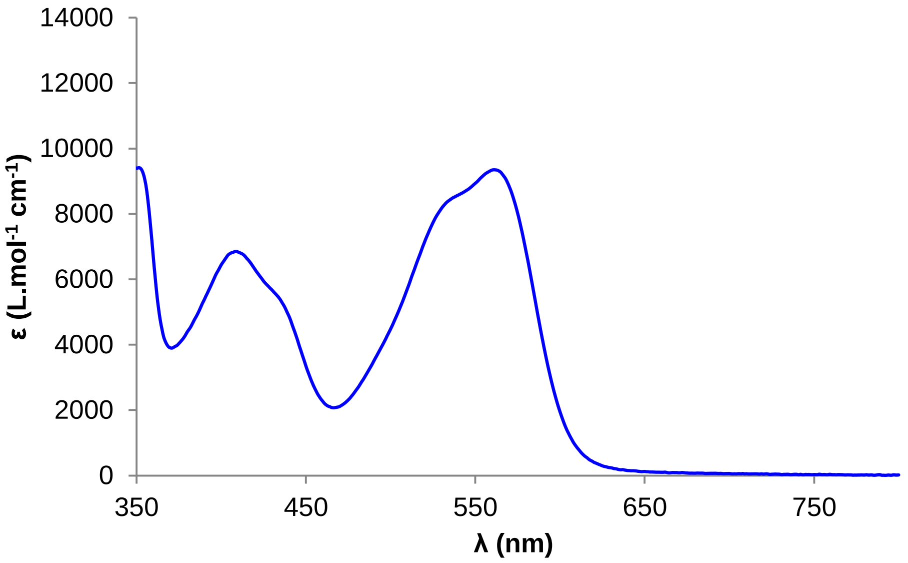

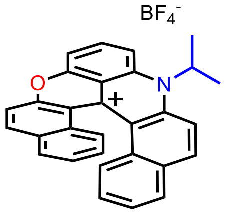

11-isopropyl-11H-benzo[a]benzo[5,6]chromeno[2,3,4-kl]acridin-17c-ylium tetrafluoroborate **2c**

^{1}H NMR (500 MHz, CD_2Cl_2) of **2c**

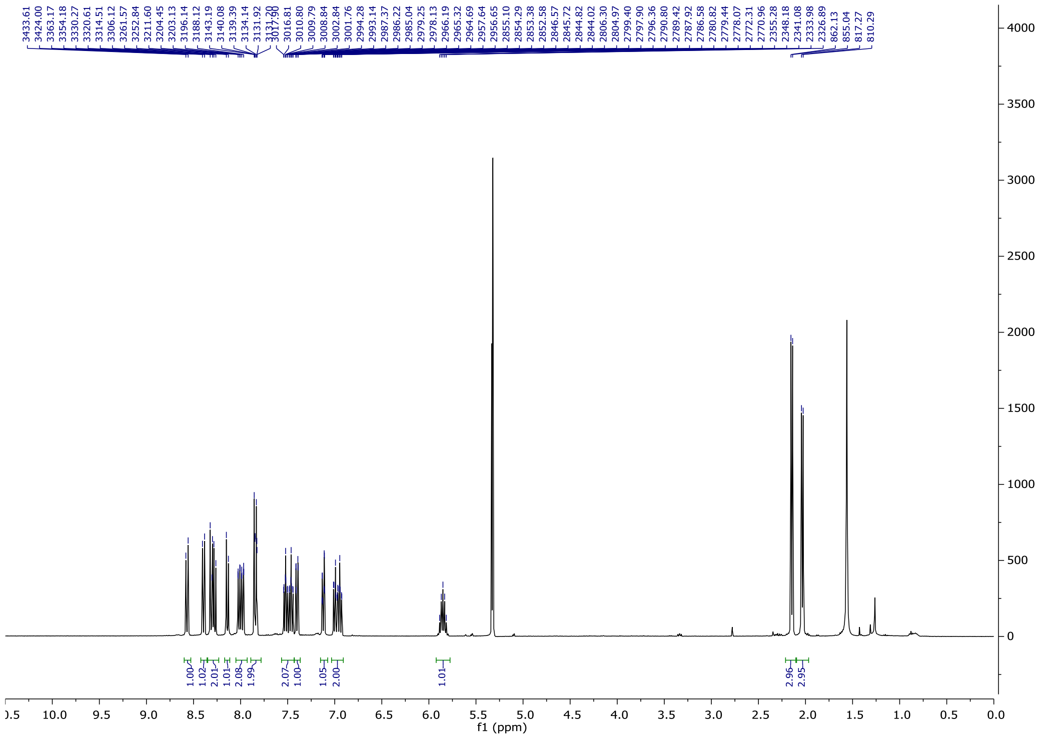

^{13}C NMR (125 MHz, CD_2Cl_2) of **2c**

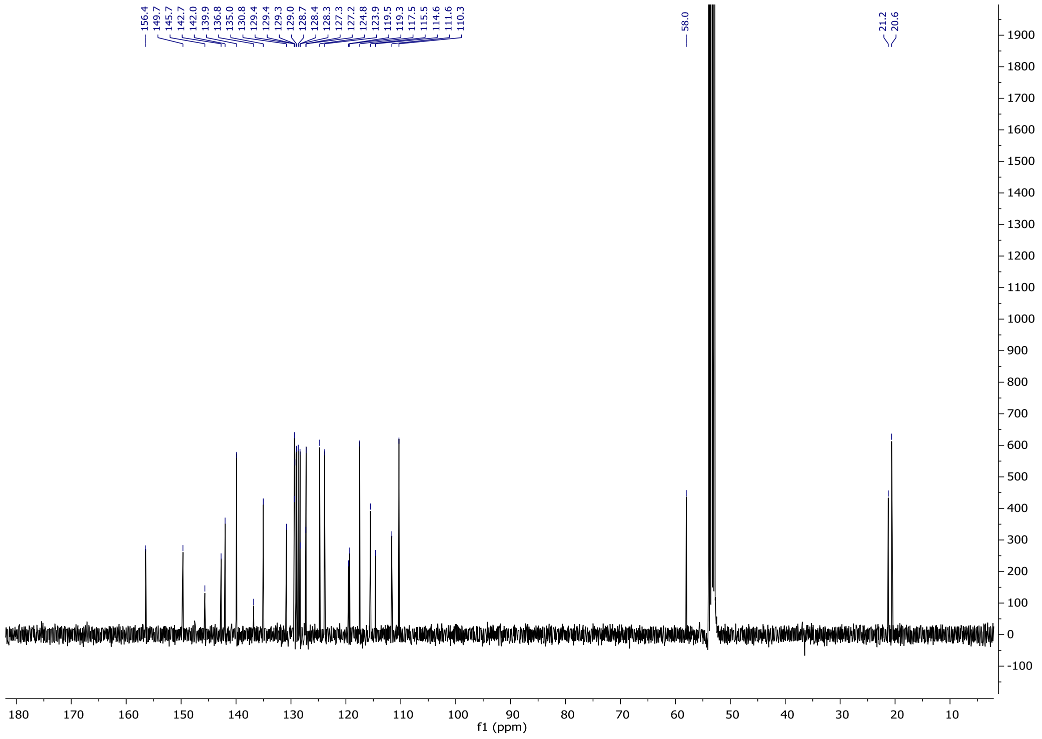

^{19}F NMR (282 MHz, CD_2Cl_2) of **2c**

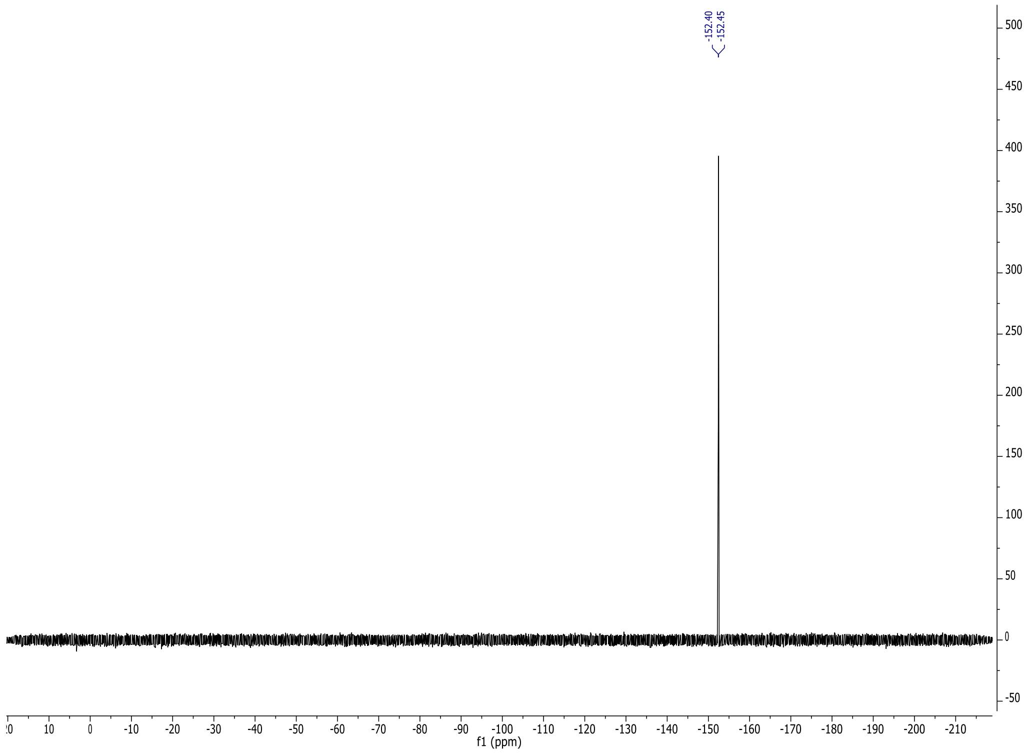

UV/VIS spectra recorded in CH_3CN (2.10^{-5} M) of **2c**

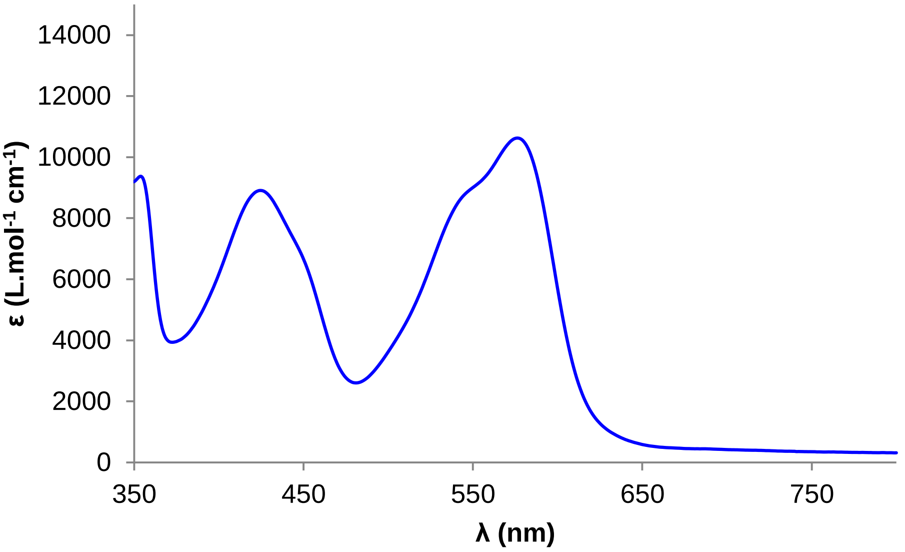

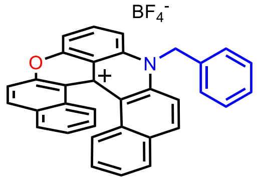

11-benzylbenzo[a]benzo[5,6]chromeno[2,3,4-kl]acridin-17c(11H)-ylium tetrafluoroborate **2d**

^1H NMR (500 MHz, CD_2Cl_2) of **2d**

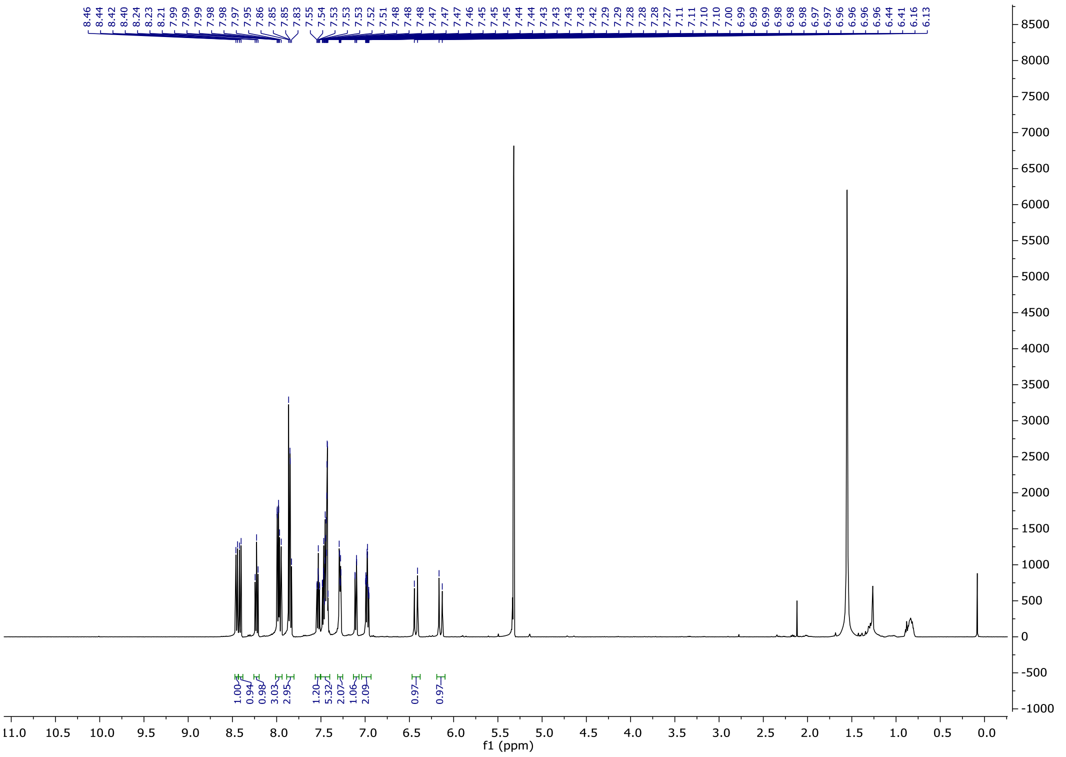

^{13}C NMR (125 MHz, CD_2Cl_2) of **2d**

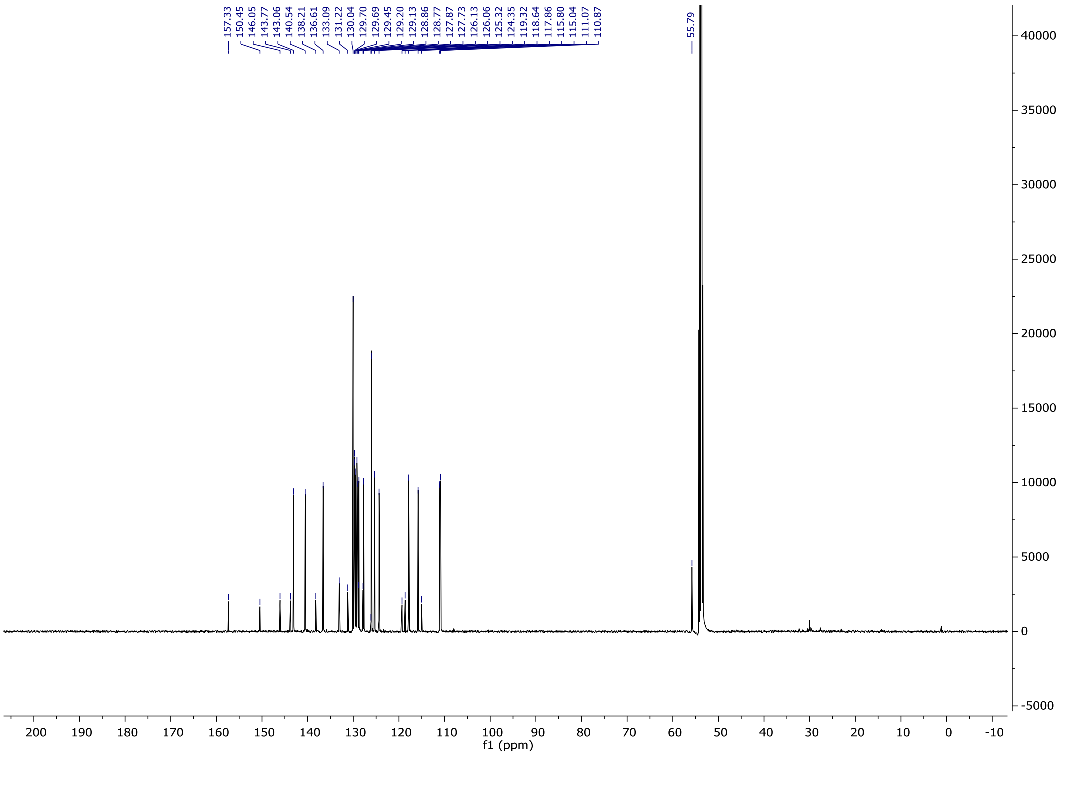

^{19}F NMR (282 MHz, CD_2Cl_2) of **2d**

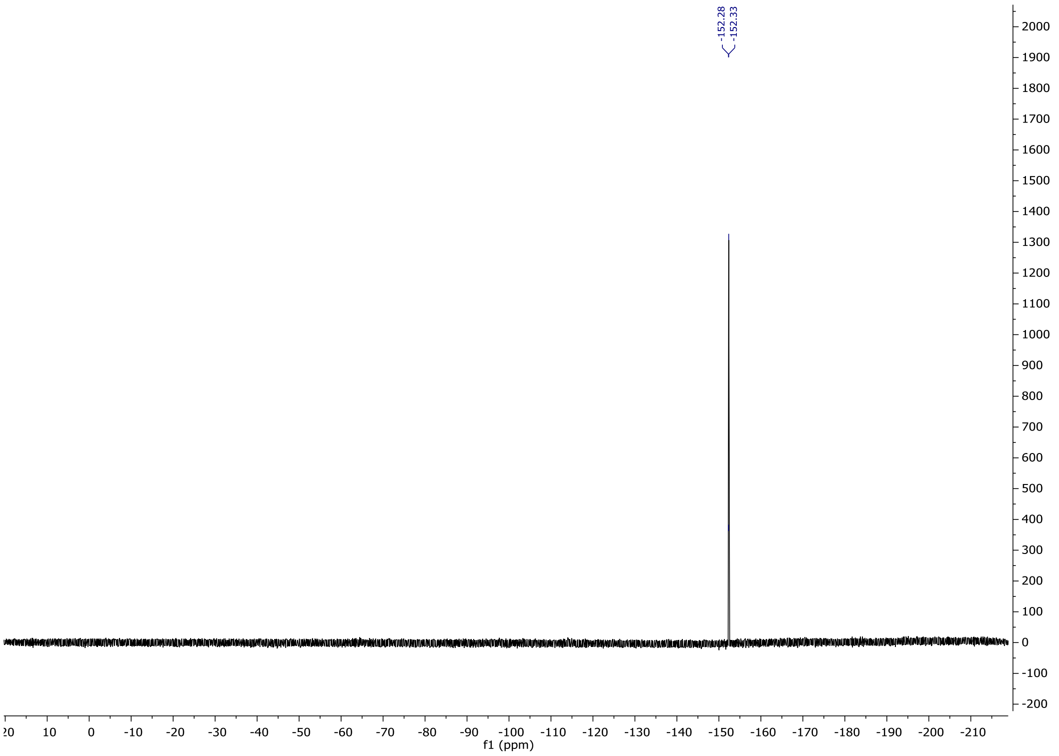

UV/VIS spectra recorded in CH_3CN (2.10^{-5} M) of **2d**

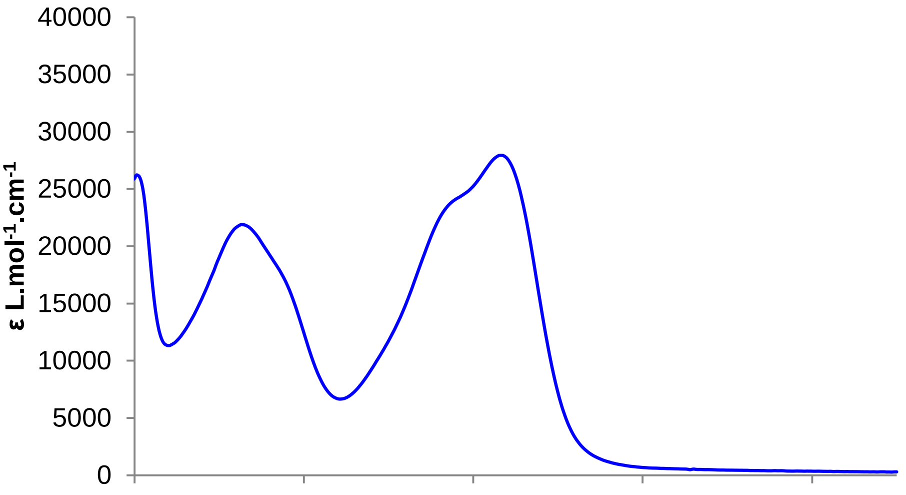

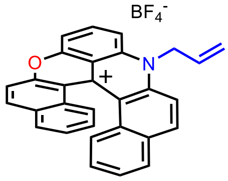

11-allyl-11H-benzo[a]benzo[5,6]chromeno[2,3,4-kl]acridin-17c-ylium tetrafluoroborate **2e**

^{1}H NMR (500 MHz, CD_2Cl_2) of **2e**

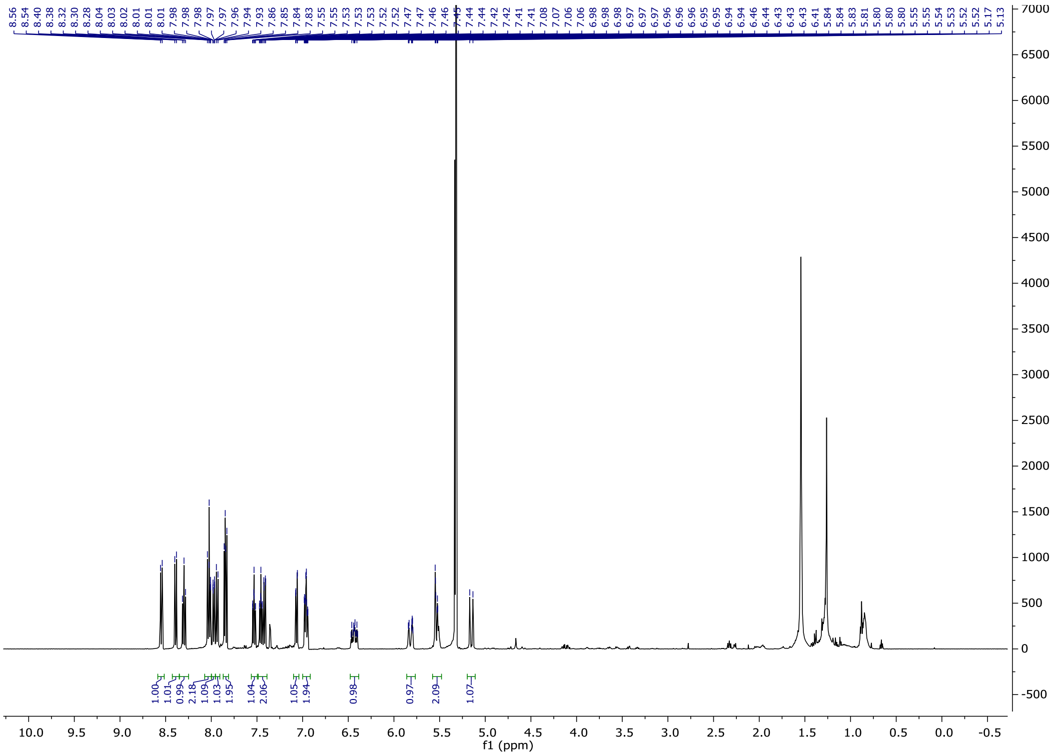

^{13}C NMR (125 MHz, CD_2Cl_2) of **2e**

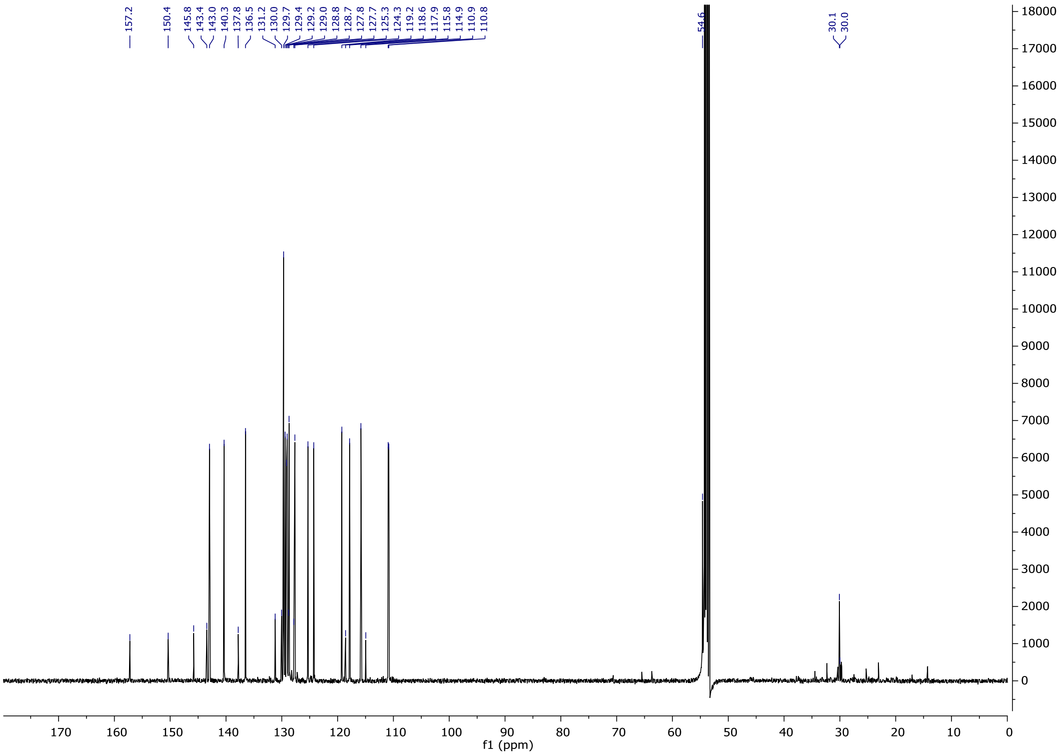

^{19}F NMR (282 MHz, CD_2Cl_2) of **2e**

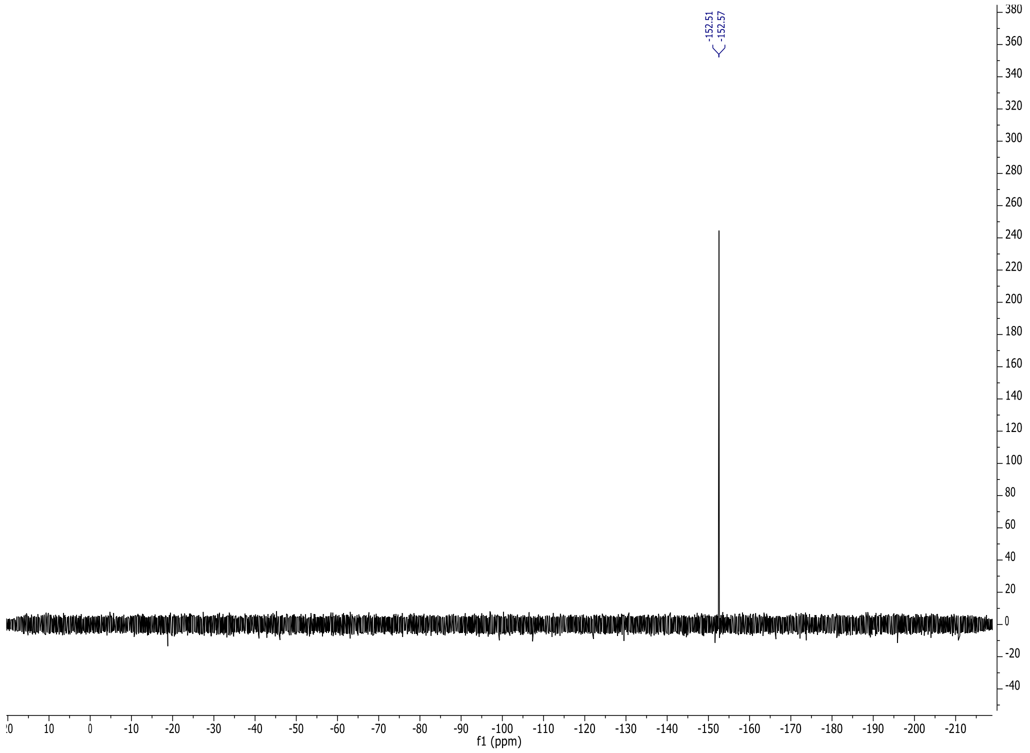

UV/VIS spectra recorded in CH_3CN (2.10^{-5} M) of **2e**

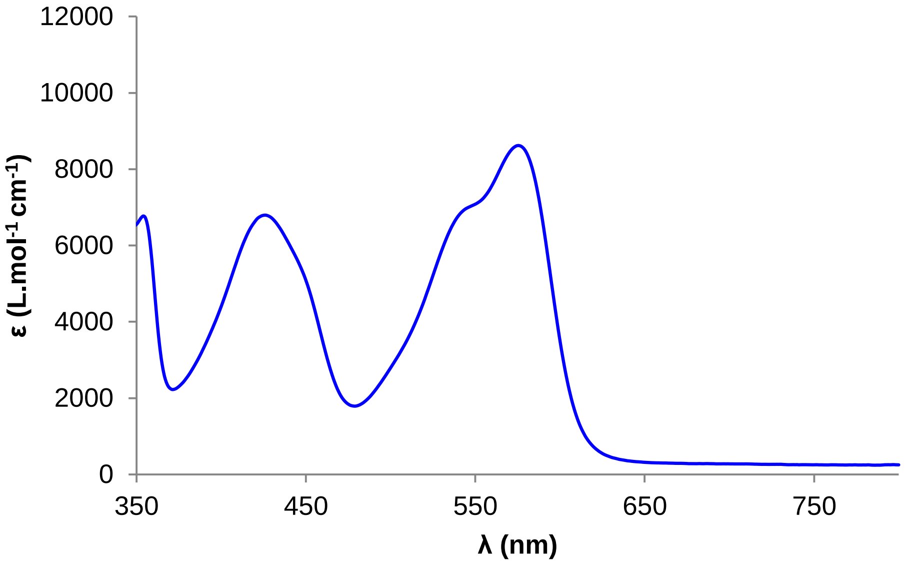

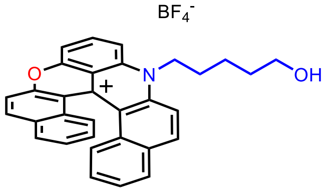

11-(5-hydroxypentyl)-11H-benzo[a]benzo[5,6]chromeno[2,3,4-kl]acridin-17c-ylium tetrafluoroborate 2f

^{1}H NMR (500 MHz, CD_2Cl_2) of **2f**

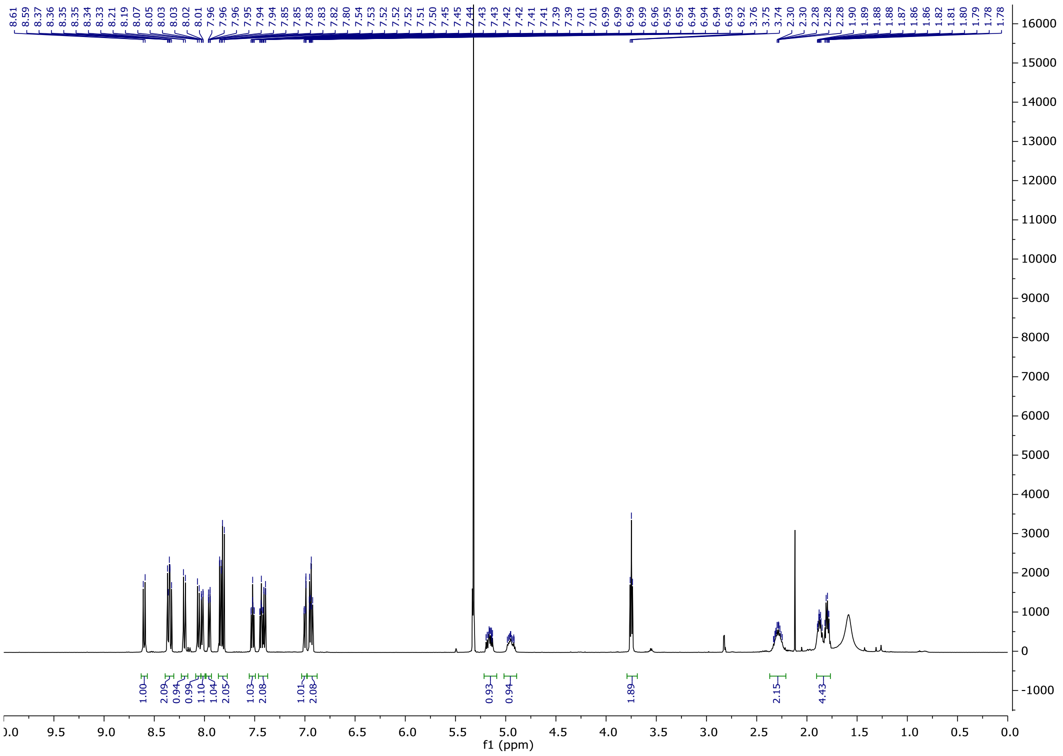

^{13}C NMR (125 MHz, CD_2Cl_2) of **2f**

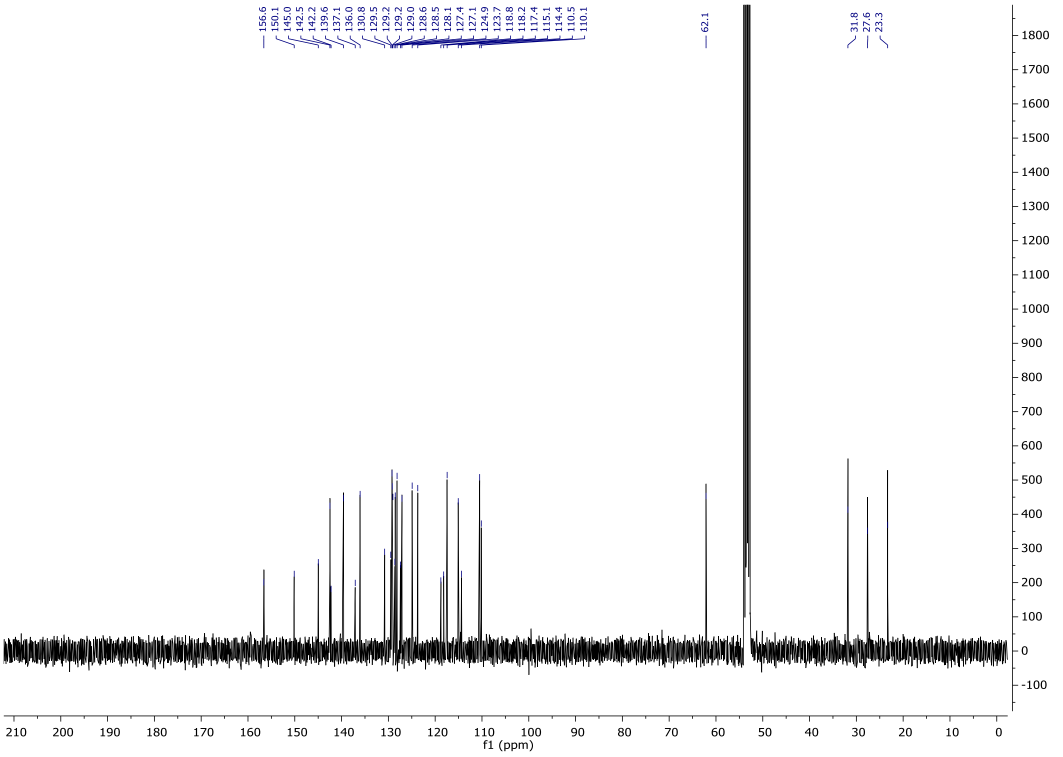

^{19}F NMR (282 MHz, CD_2Cl_2) of **2f**

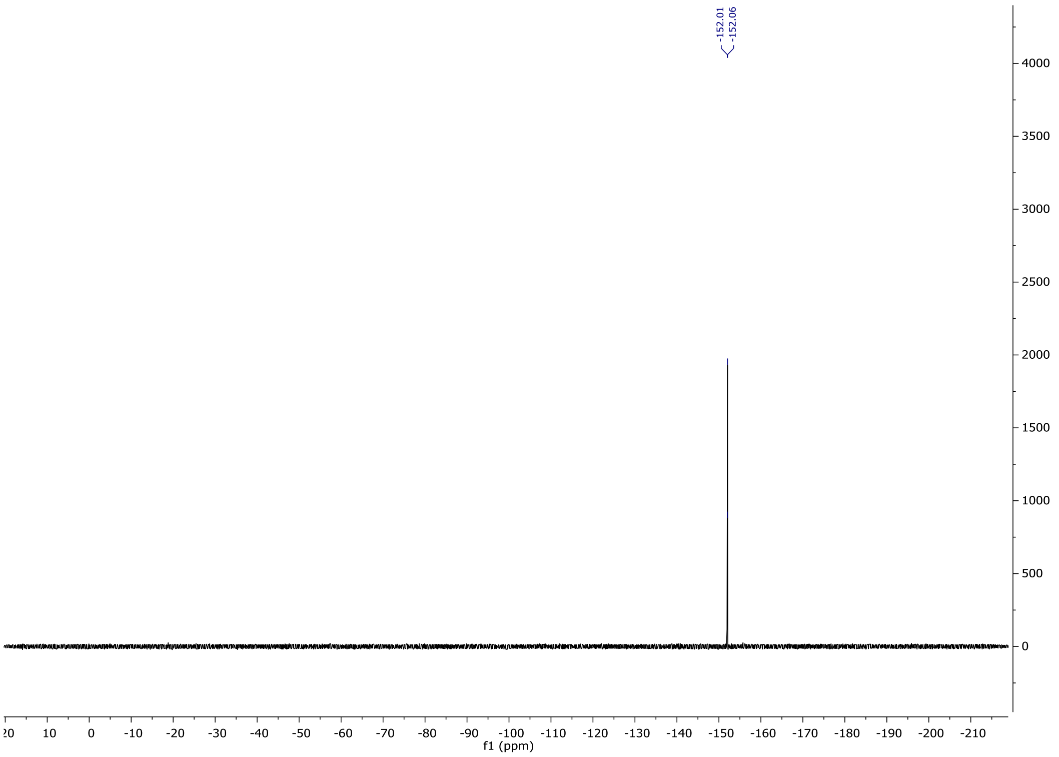

UV/VIS spectra recorded in CH_3CN (2.10^{-5} M) of **2f**

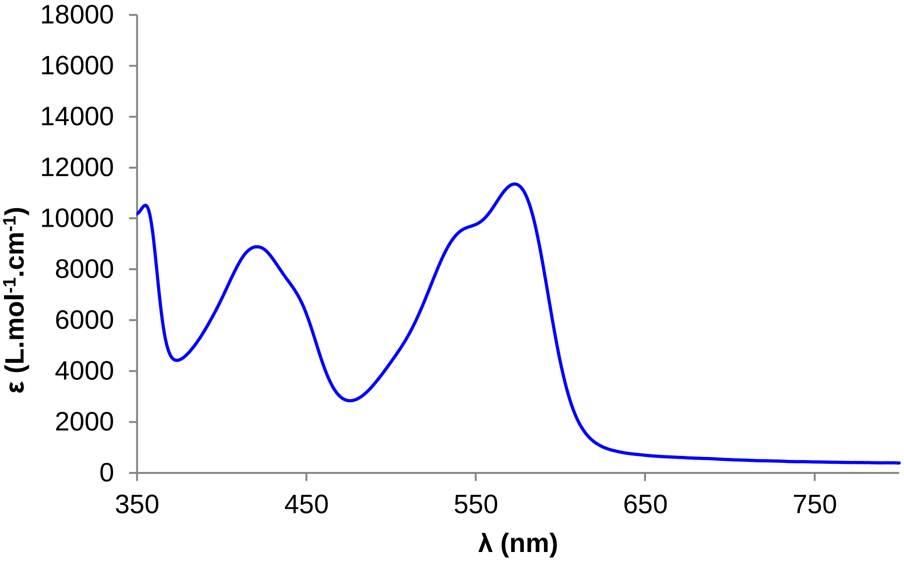

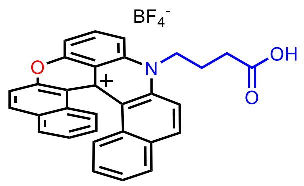

11-(3-carboxypropyl)benzo[a]benzo[5,6]chromeno[2,3,4-kl]acridin-17c(11H)-ylium tetrafluoroborate **2g**

^{1}H NMR (500 MHz, CD_2Cl_2) of **2g**

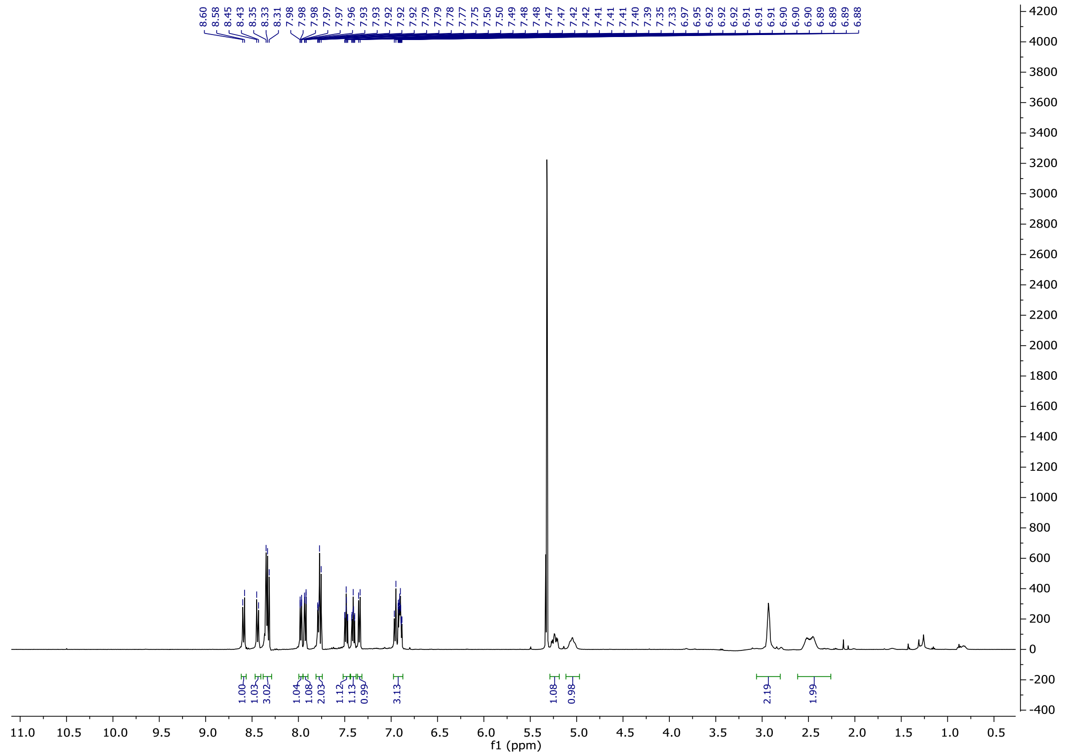

^{13}C NMR (125 MHz, CD_2Cl_2) of **2g**

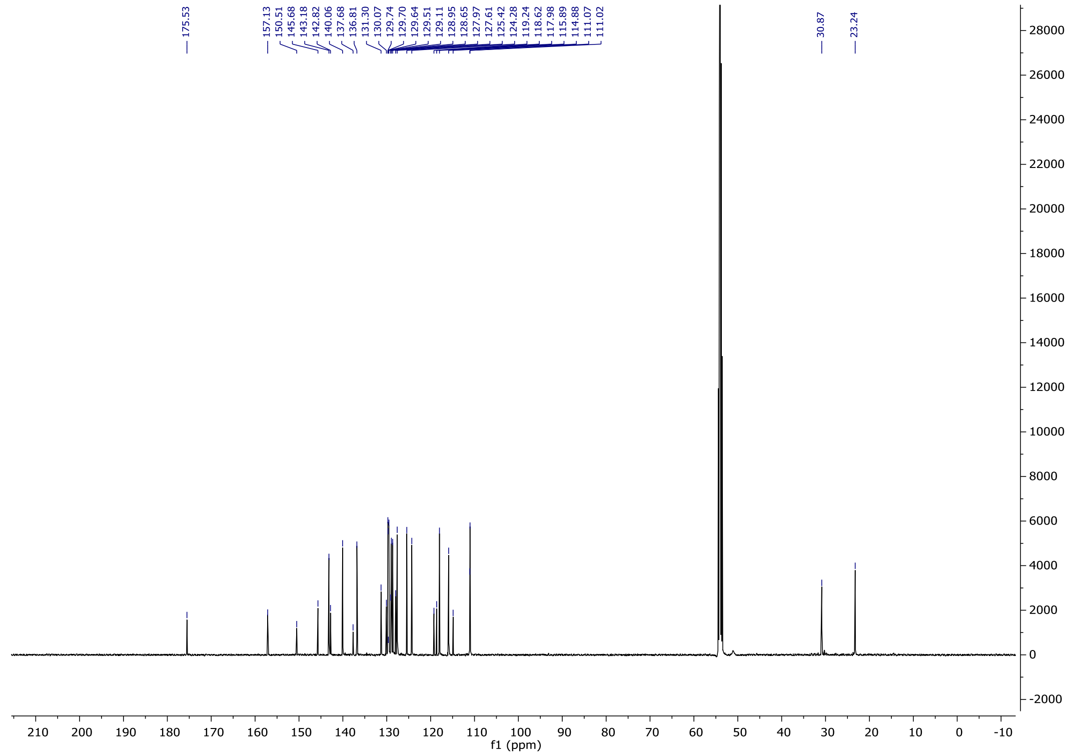

^{19}F NMR (282 MHz, CD_2Cl_2) of **2g**

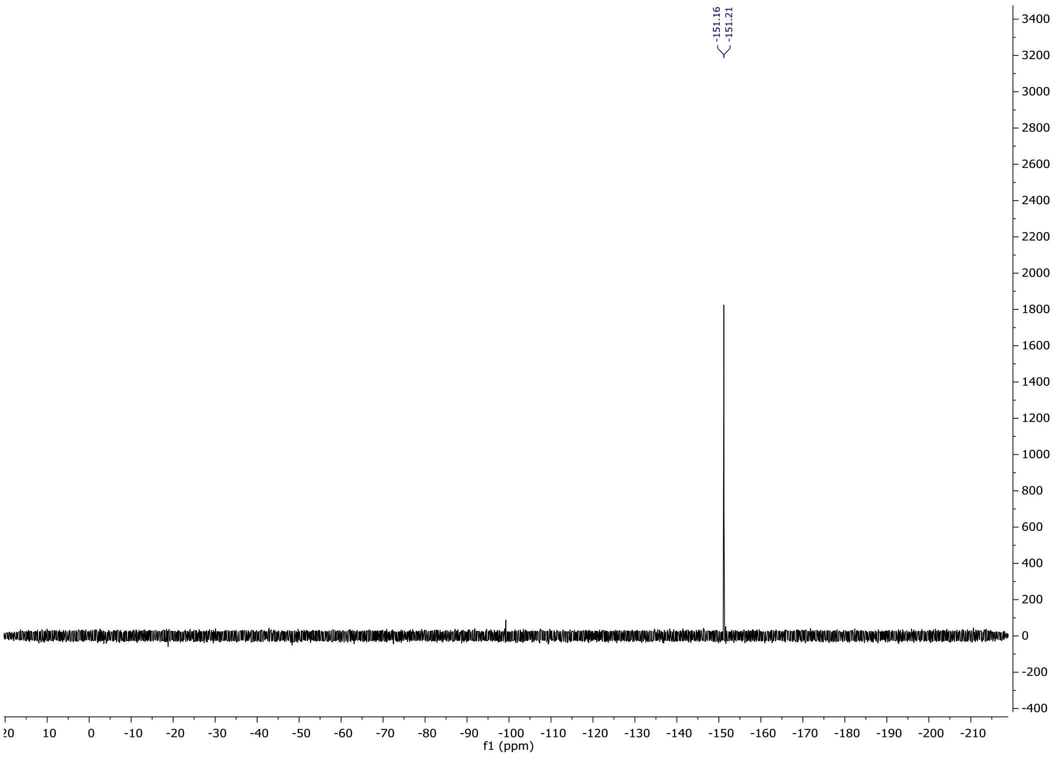

UV/VIS spectra recorded in CH_3CN (2.10^{-5} M) of **2g**

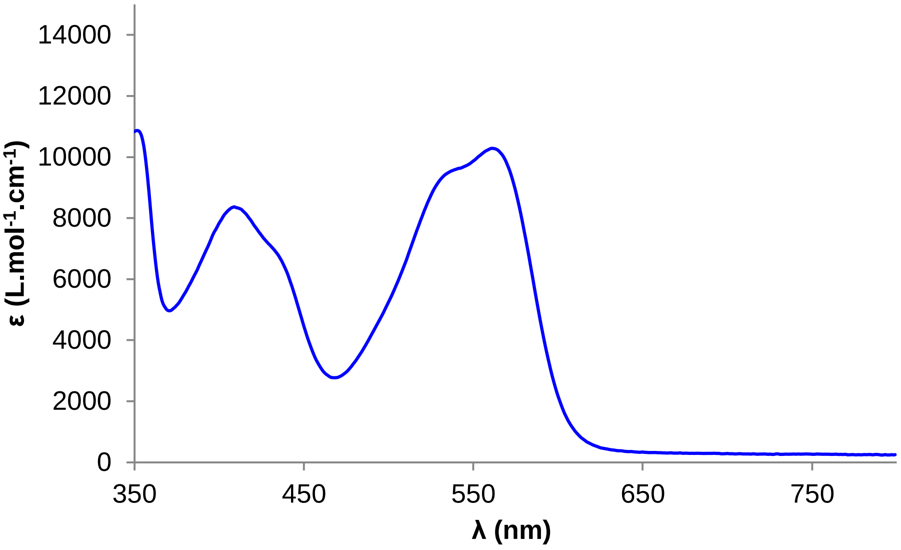

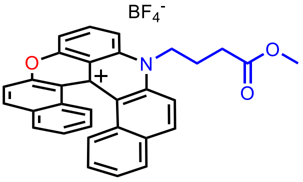

11-(4-methoxy-4-oxobutyl)benzo[a]benzo[5,6]chromeno[2,3,4-kl]acridin-17c(11H)-ylium tetrafluoroborate **2h**

^{1}H NMR (500 MHz, CD_2Cl_2) of **2h**

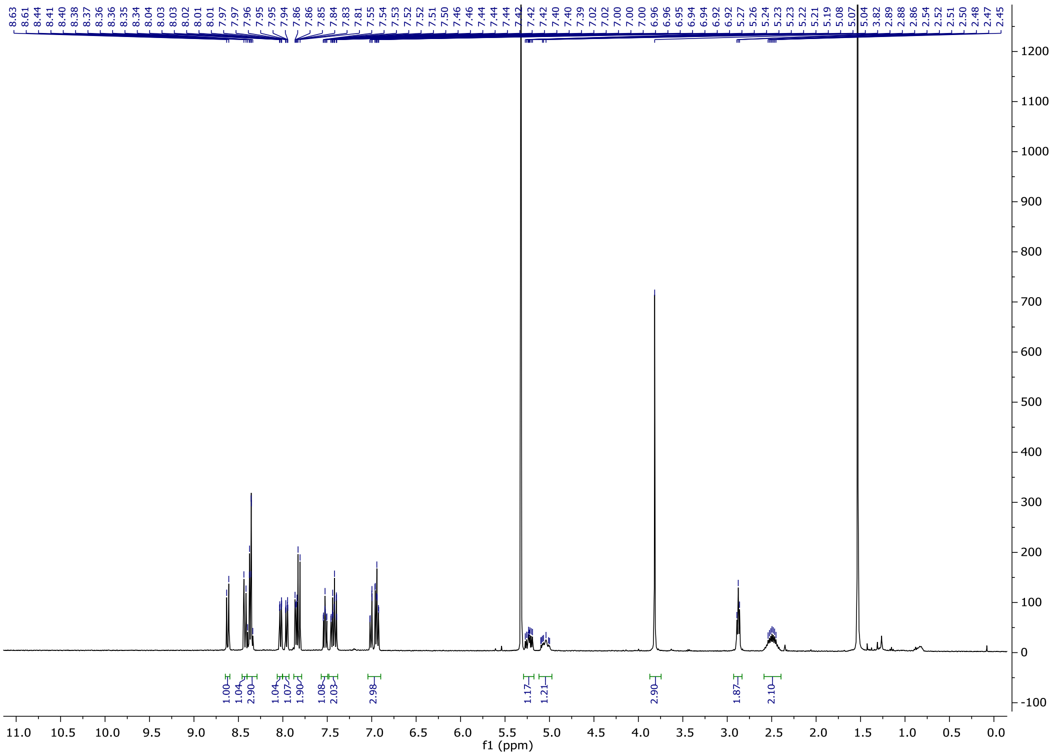

^{13}C NMR (125 MHz, CD_2Cl_2) of **2h**

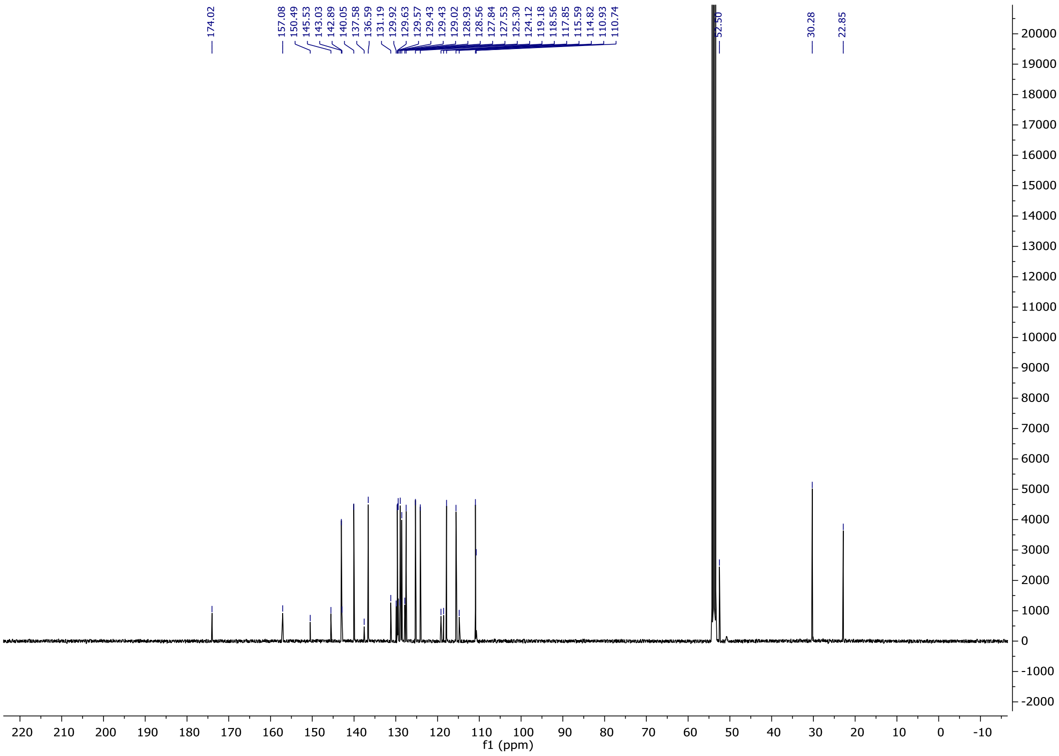

^{19}F NMR (282 MHz, CD_2Cl_2) of **2h**

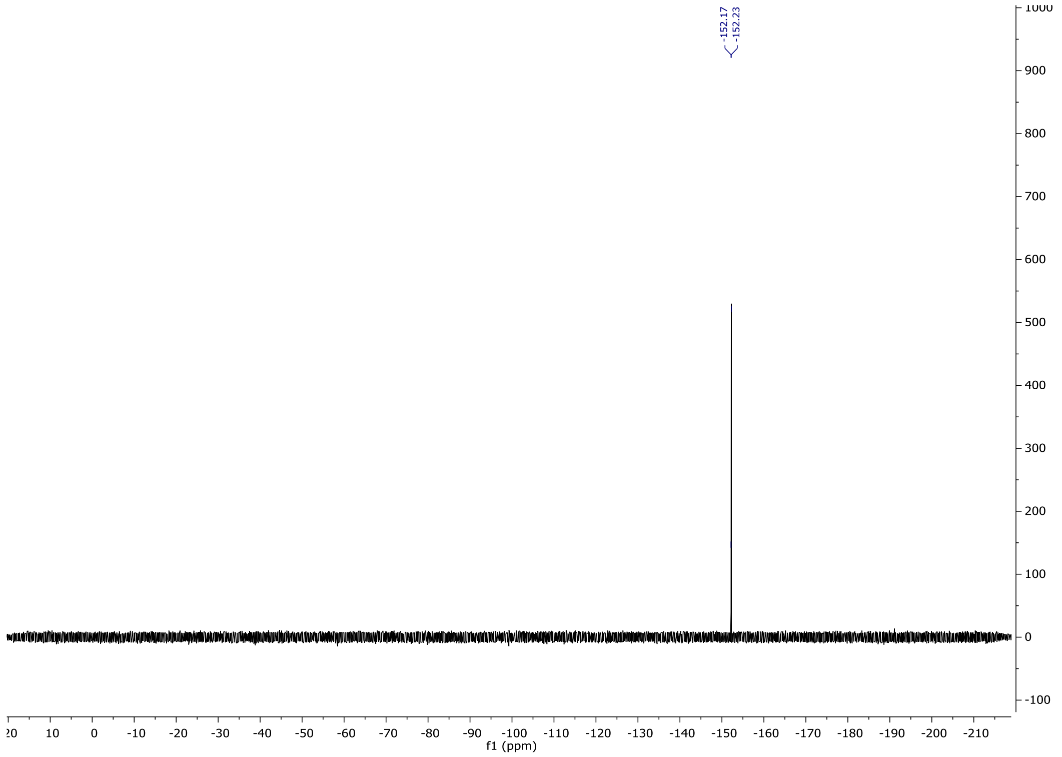

UV/VIS spectra recorded in CH_3CN (2.10^{-5} M) of **2h**

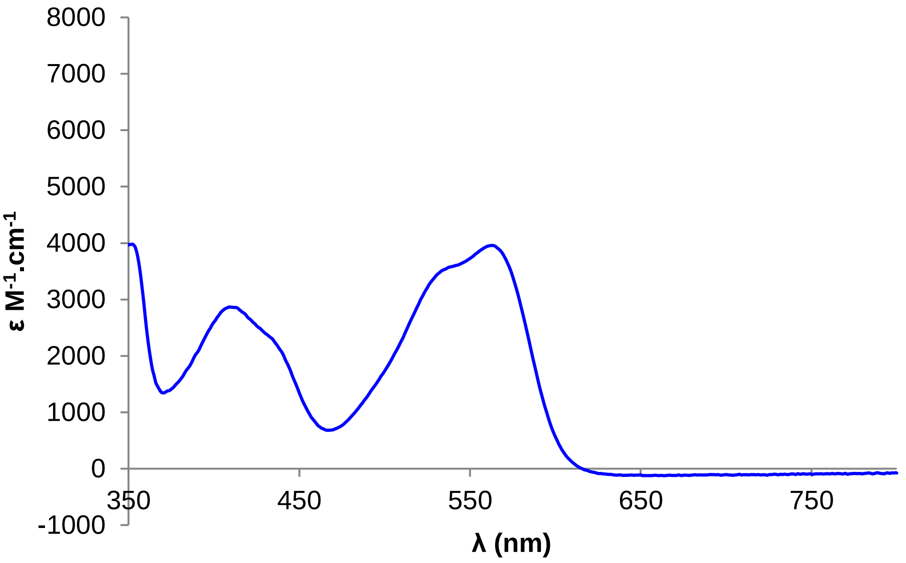

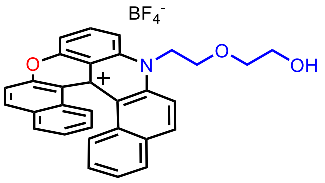

11-(2-(2-hydroxyethoxy)ethyl)-11H-benzo[a]benzo[5,6]chromeno[2,3,4-kl]acridin-17c-ylium tetrafluoroborate **2i**

^{1}H NMR (500 MHz, CD_2Cl_2) of **2i**

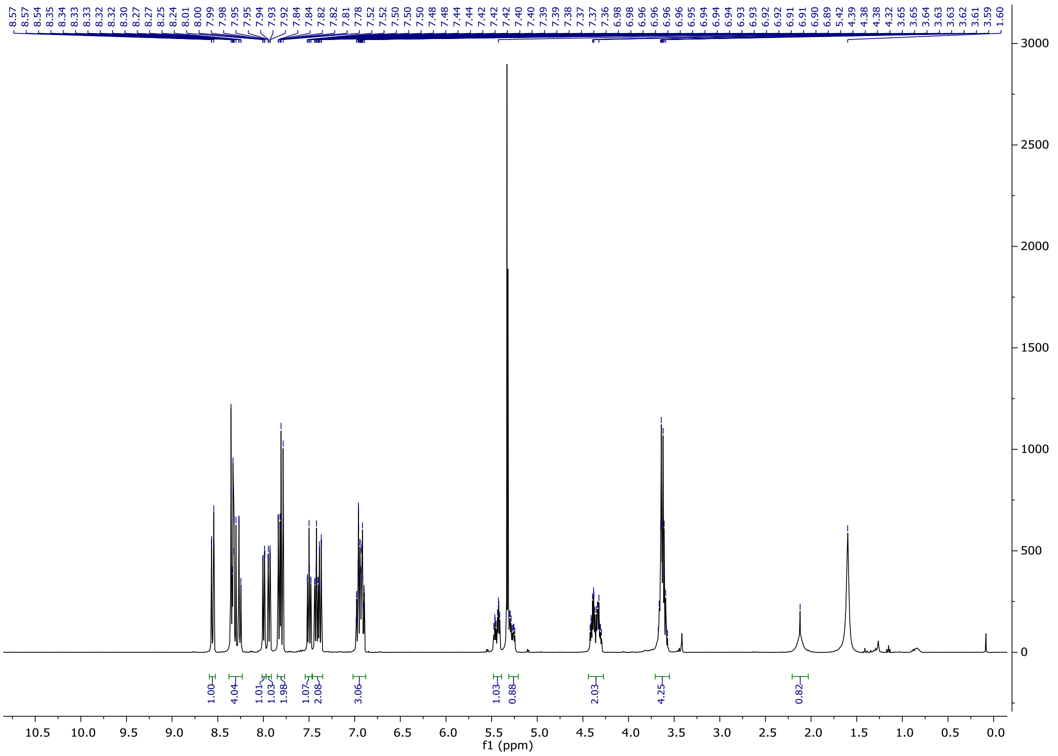

^{13}C NMR (125 MHz, CD_2Cl_2) of **2i**

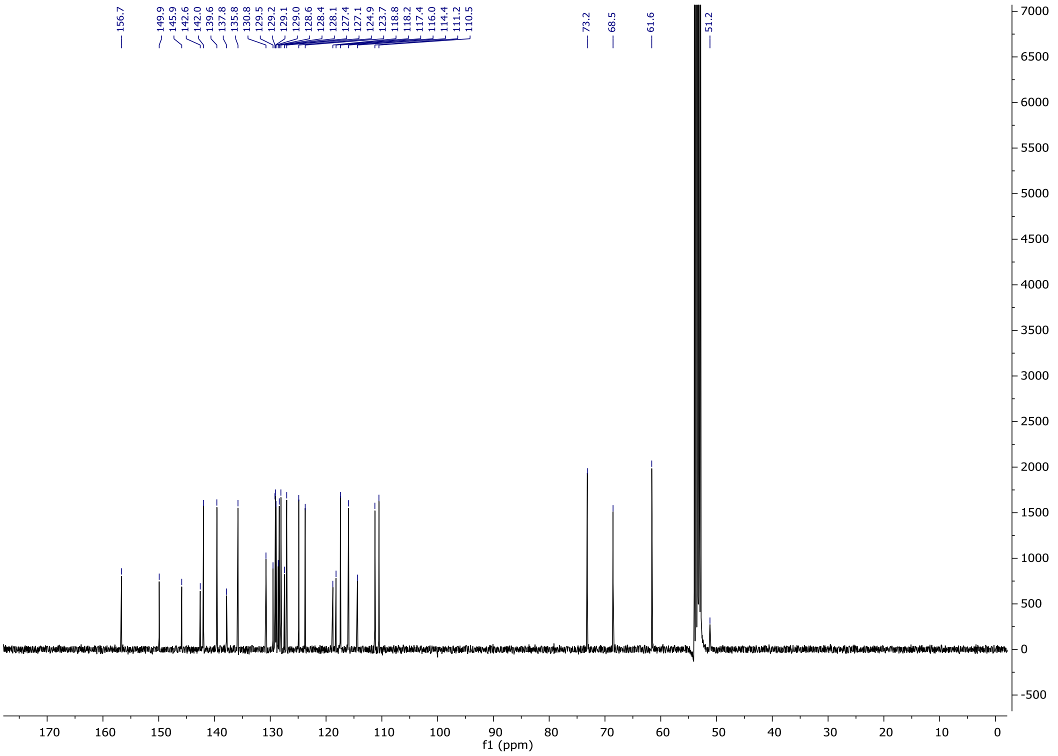

^{19}F NMR (282 MHz, CD_2Cl_2) of **2i**

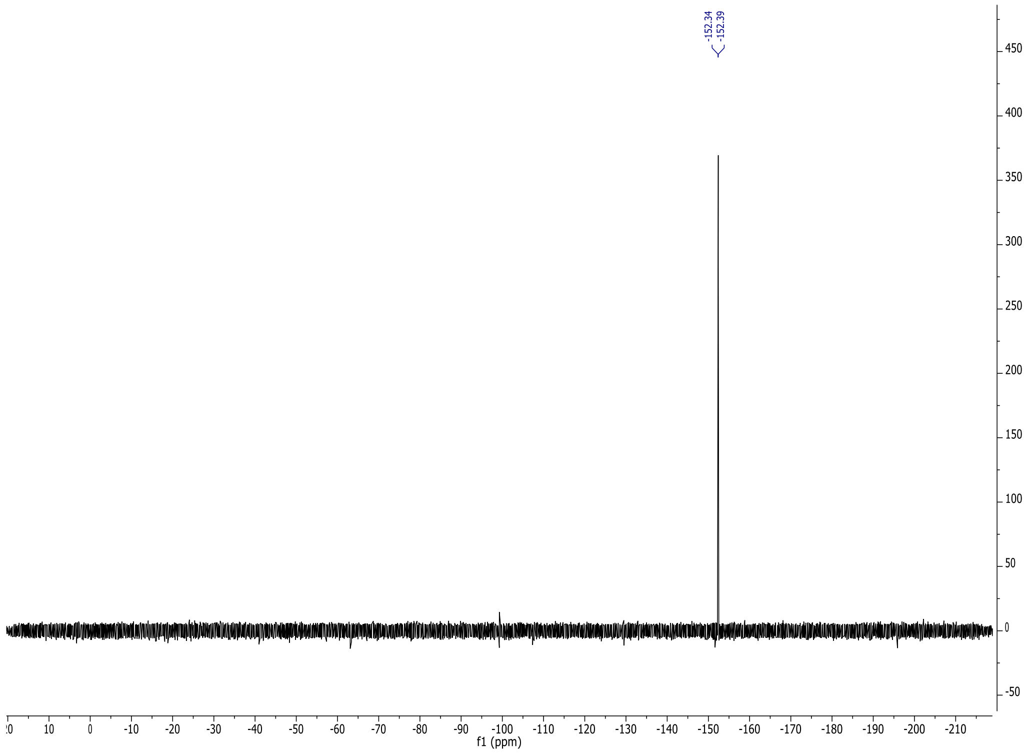

UV/VIS spectra recorded in CH_3CN (2.10^{-5} M) of **2i**

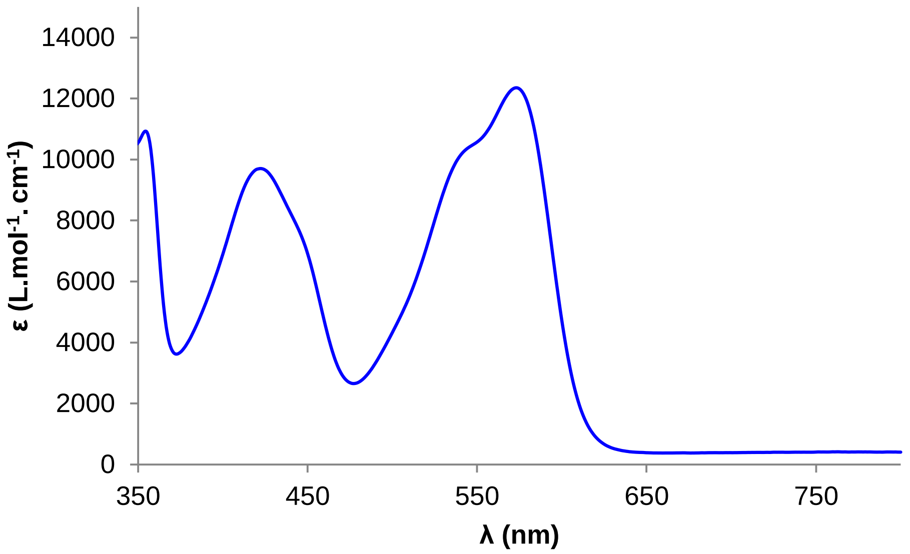

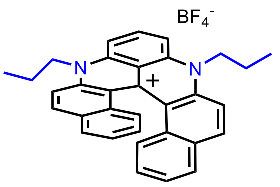

7,11-dipropyl-7,11-dihydro-17cH-benzo[a]benzo[5,6]quinolino[2,3,4-kl]acridin-17c-ylium tetrafluoroborate **3a**

^{1}H NMR (500 MHz, CD_2Cl_2) of **3a**

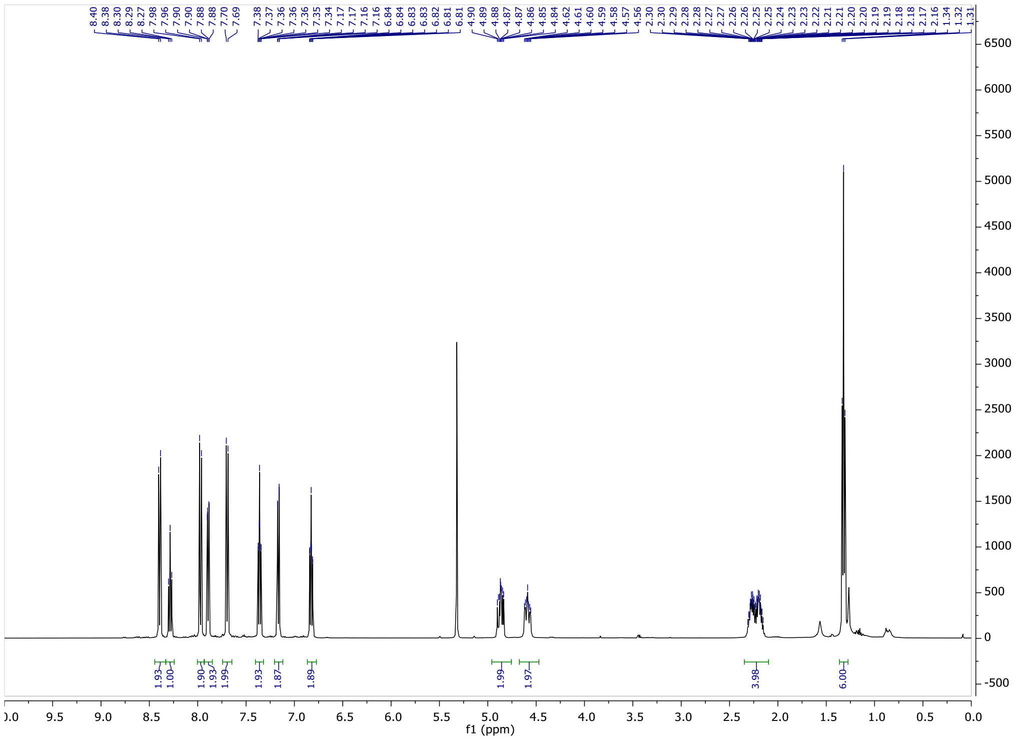

^{13}C NMR (125 MHz, CD_2Cl_2) of **3a**

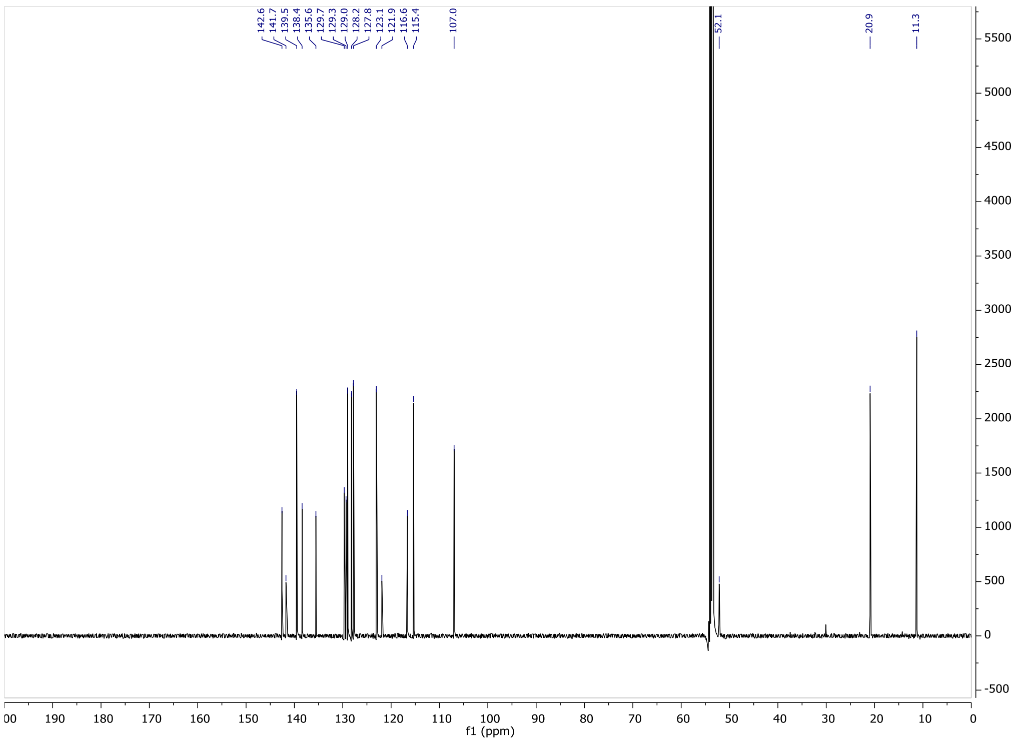

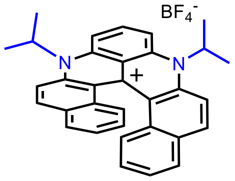

7,11-diisopropyl-7,11-dihydrobenzo[a]benzo[5,6]quinolino[2,3,4-kl]acridin-17c-ylium tetrafluoroborate **3b**

^{1}H NMR (500 MHz, CD_2Cl_2) of **3b**

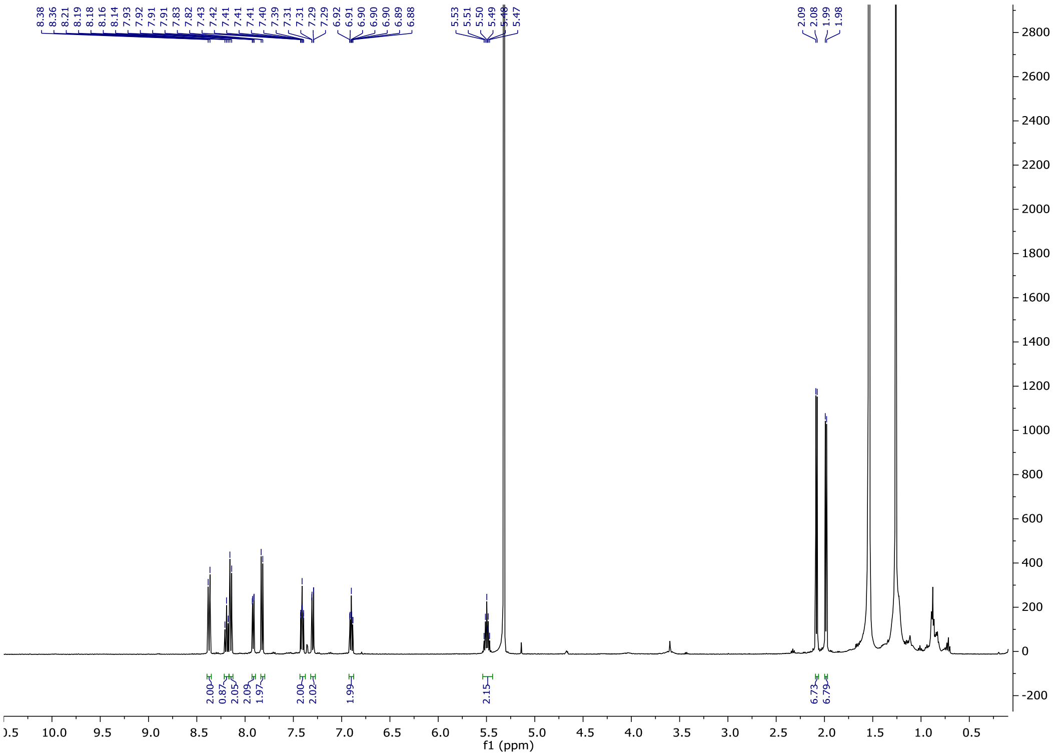

^{13}C NMR (125 MHz, CD_2Cl_2) of **3b**

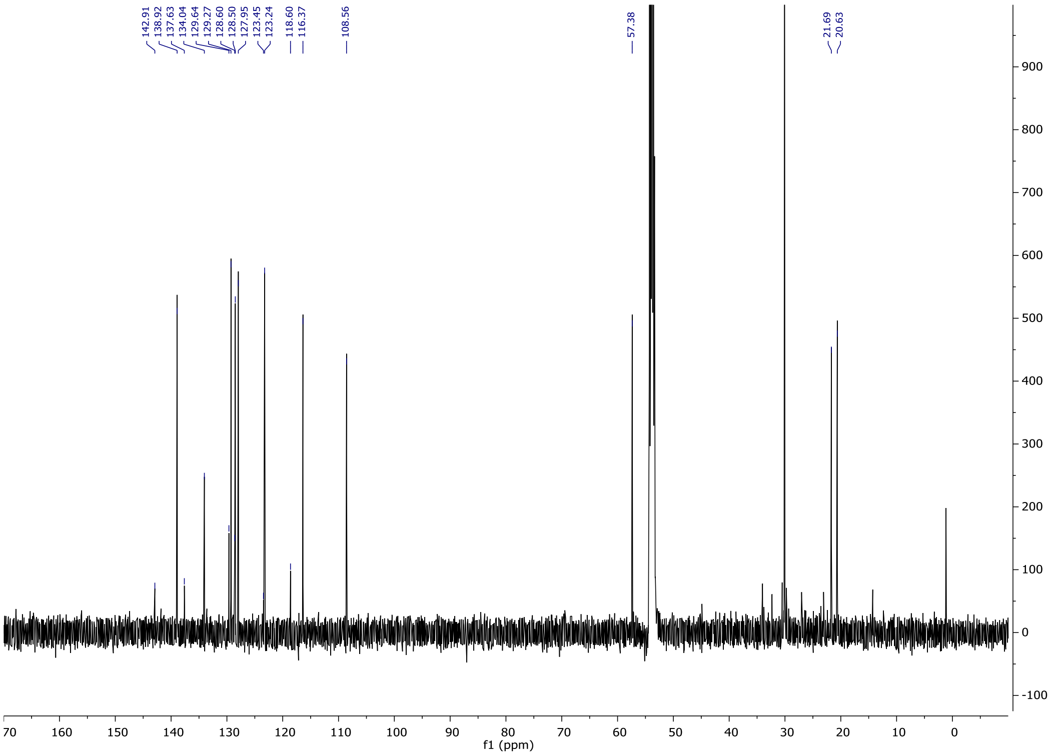

^{19}F NMR (282 MHz, CD_2Cl_2) of **3b**

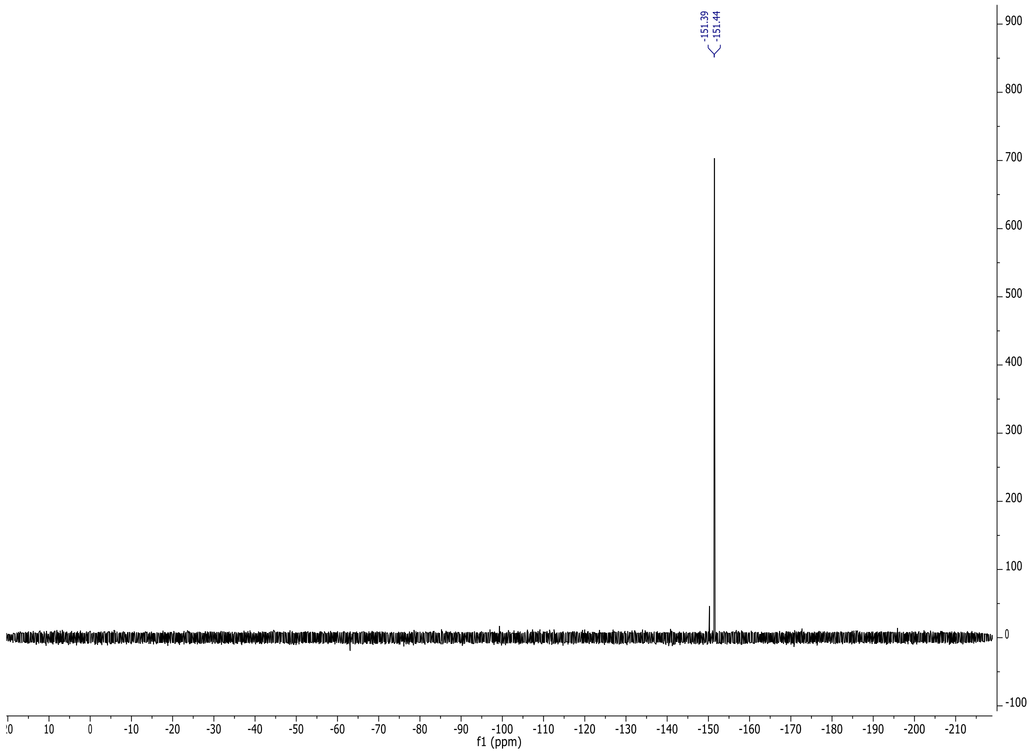

UV/VIS spectra recorded in CH_3CN (2.10^{-5} M) of **3b**

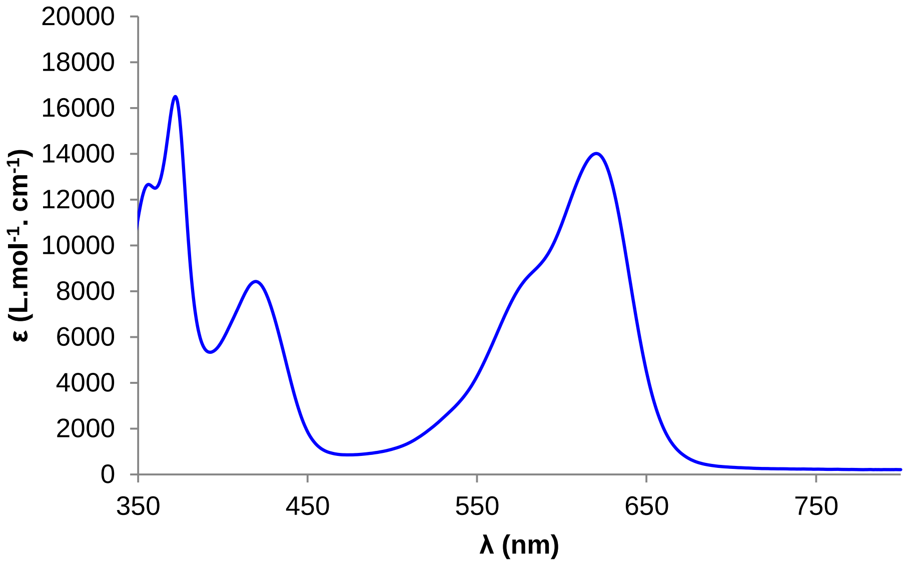

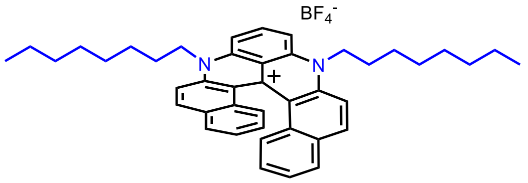

7,11-dioctyl-7,11-dihydrobenzo[a]benzo[5,6]quinolino[2,3,4-kl]acridin-17c-ylium tetrafluoroborate **3c**

^{1}H NMR (500 MHz, CD_2Cl_2) of **3c**

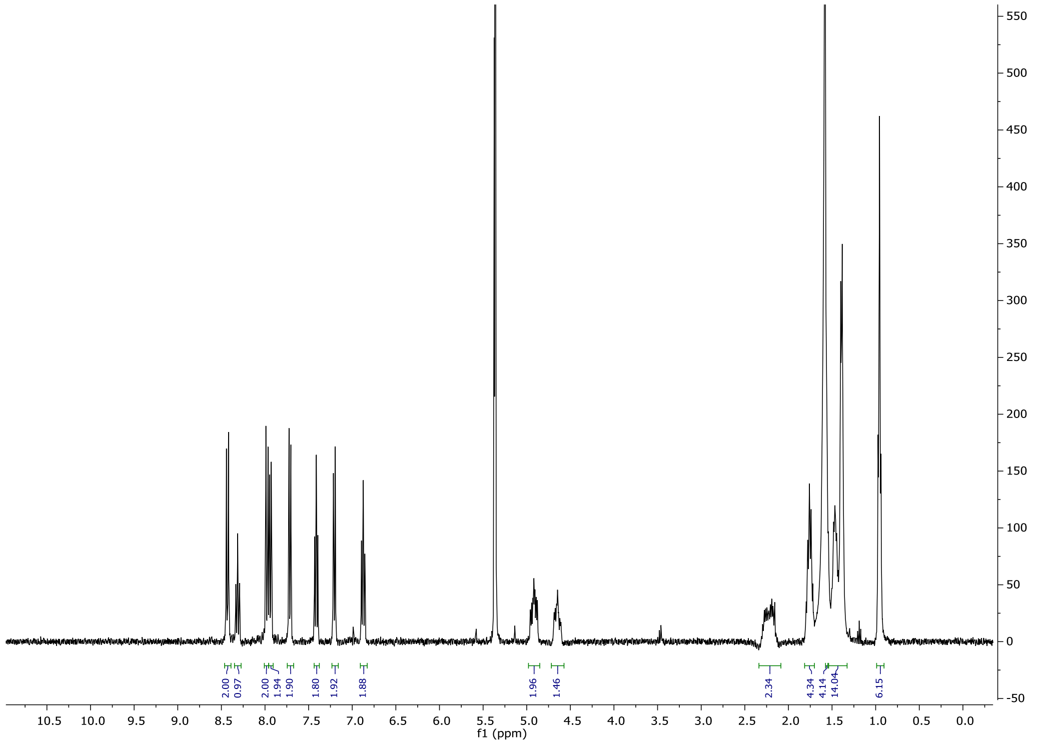

^{13}C NMR (125 MHz, CD_2Cl_2) of **3c**

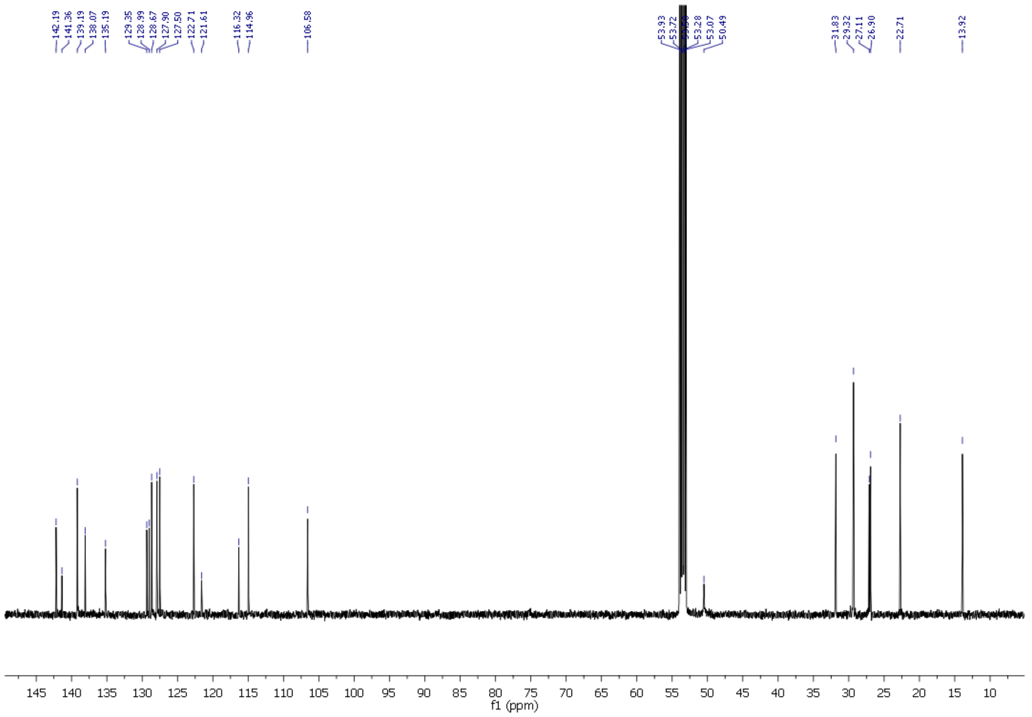

^{19}F NMR (282 MHz, CD_2Cl_2) of **3c**

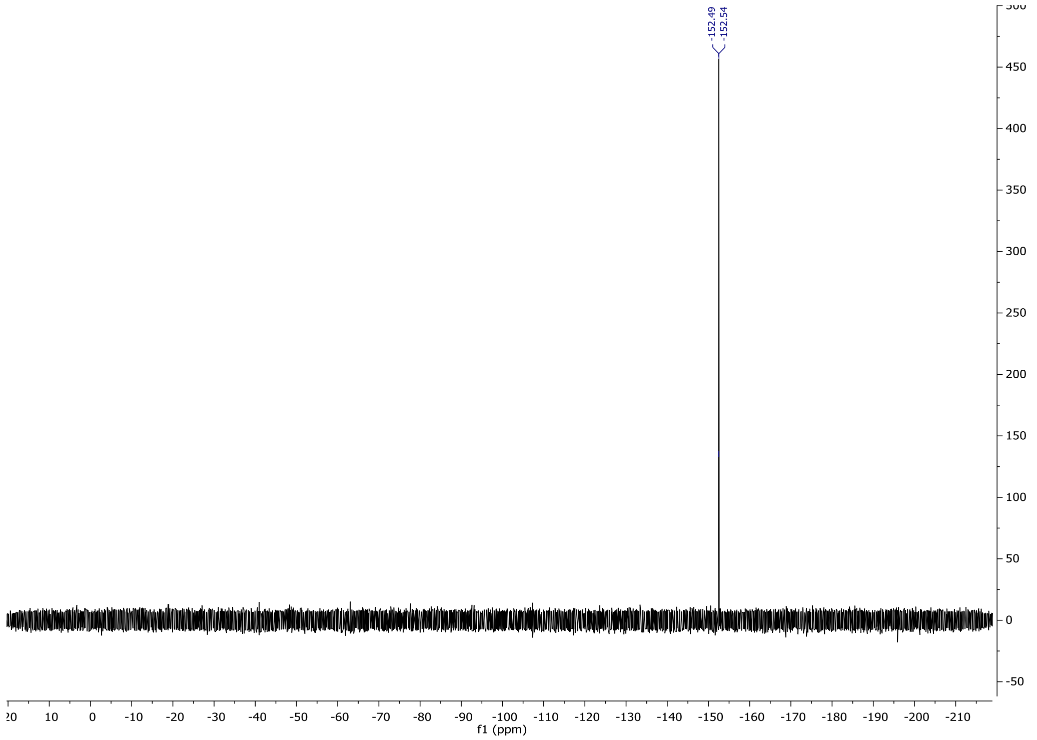

UV/VIS spectra recorded in CH_3CN (2.10^{-5} M) of **3c**

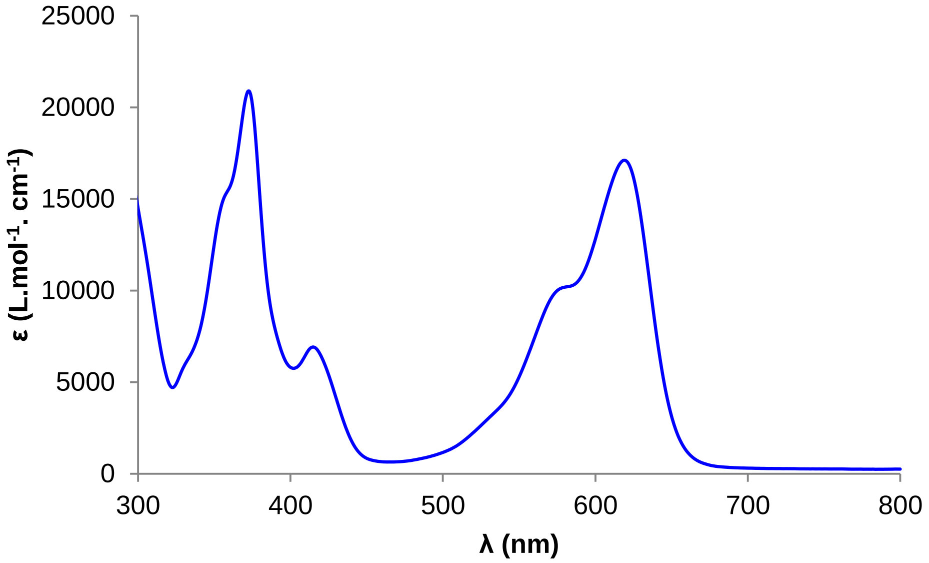

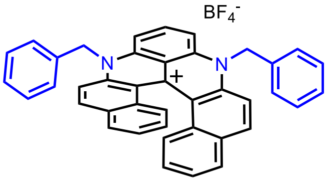

7,11-dibenzyl-7,11-dihydro-17cH-benzo[a]benzo[5,6]quinolino[2,3,4-kl]acridin-17c-ylium tetrafluoroborate **3d**

^{1}H NMR (500 MHz, CD_2Cl_2) of **3d**

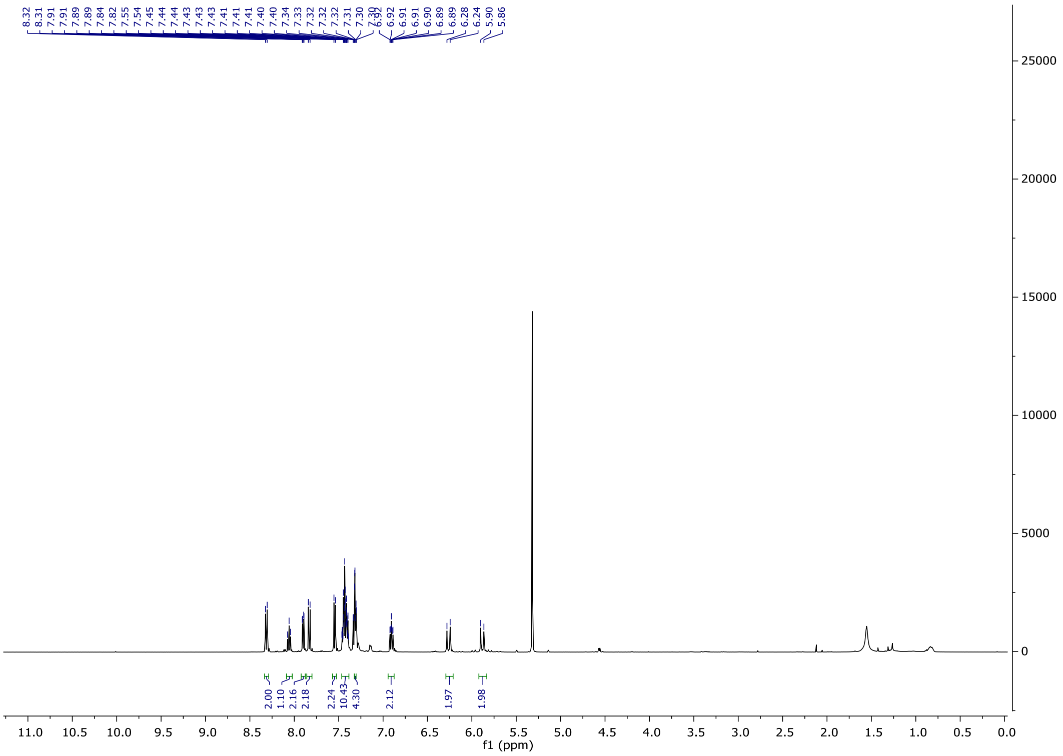

^{13}C NMR (125 MHz, CD_2Cl_2) of **3d**

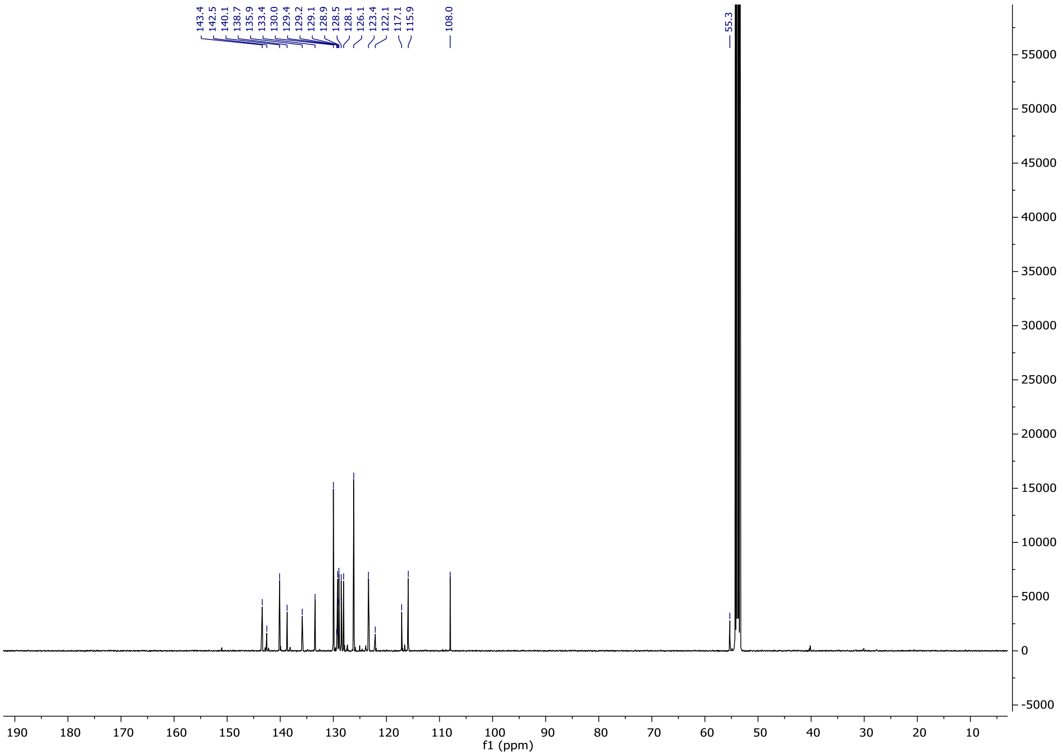

^{19}F NMR (282 MHz, CD_2Cl_2) of **3d**

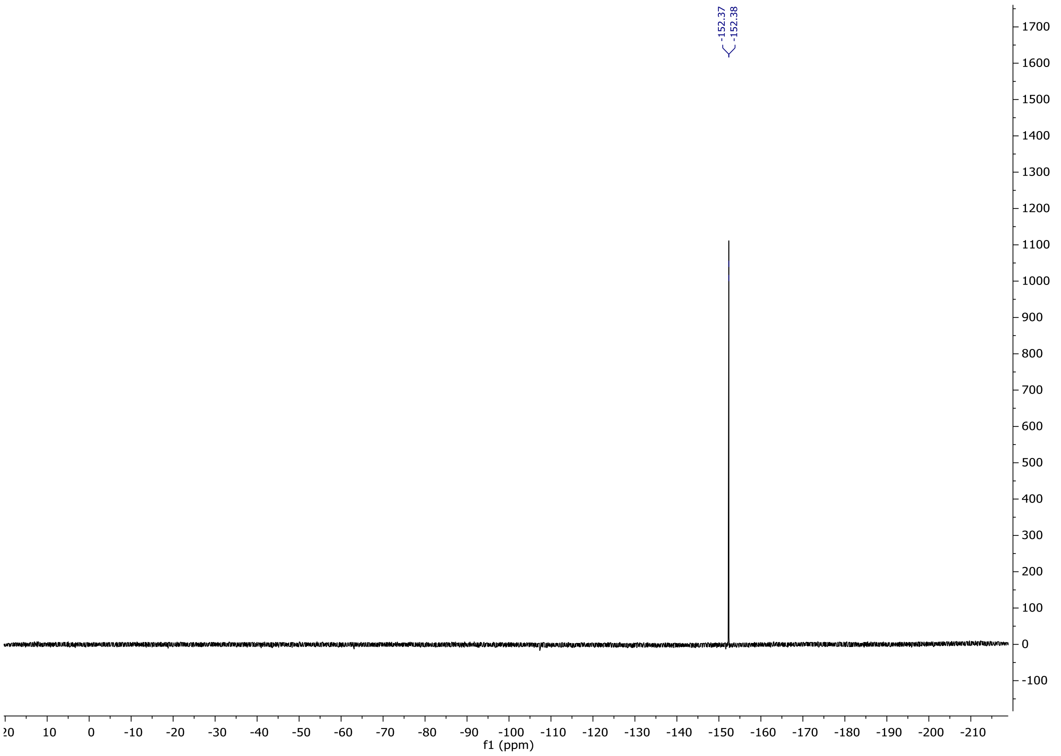

UV/VIS spectra recorded in CH_3CN (2.10^{-5} M) of **3d**

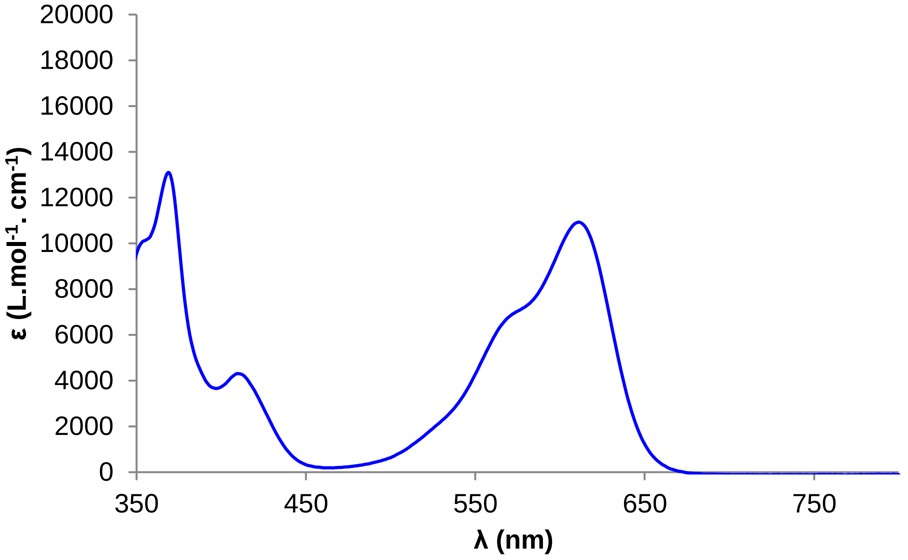

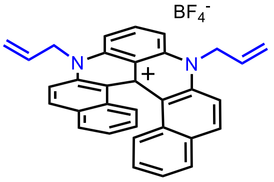

7,11-diallyl-7,11-dihydrobenzo[a]benzo[5,6]quinolino[2,3,4-kl]acridin-17c-ylium tetrafluoroborate **3e**

^{1}H NMR (500 MHz, CD_2Cl_2) of **3e**

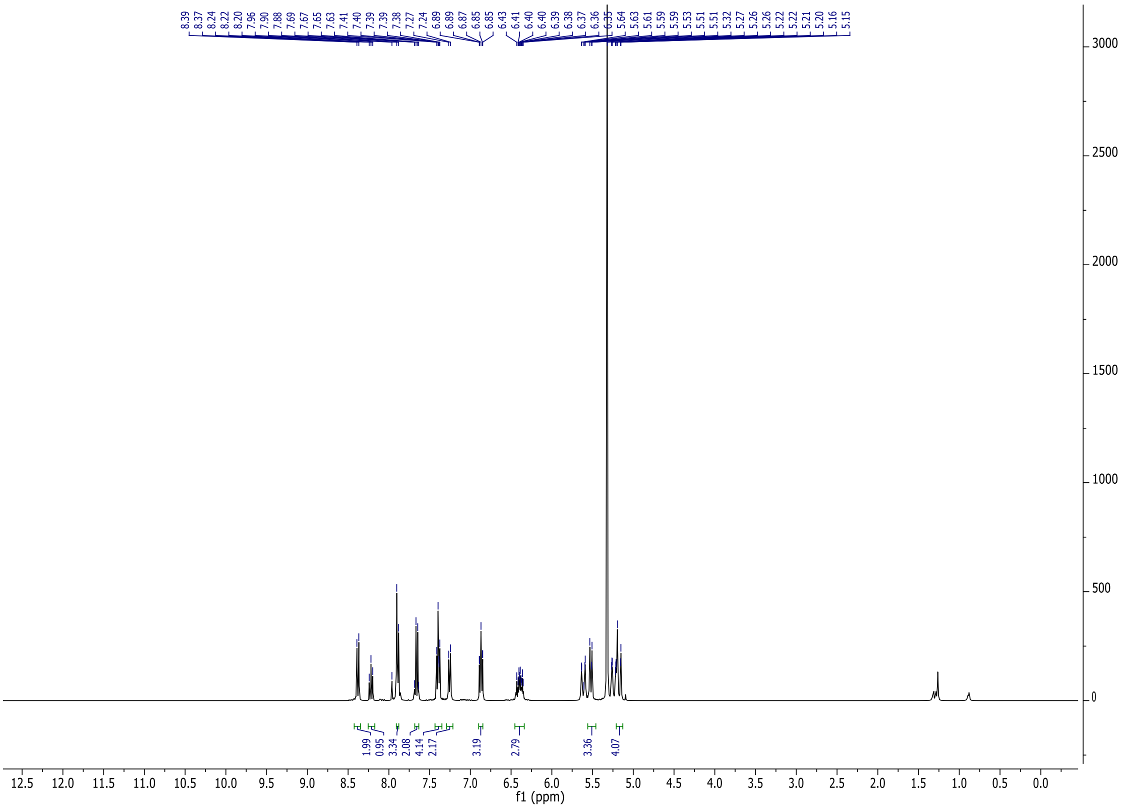

^{13}C NMR (125 MHz, CD_2Cl_2) of **3e**

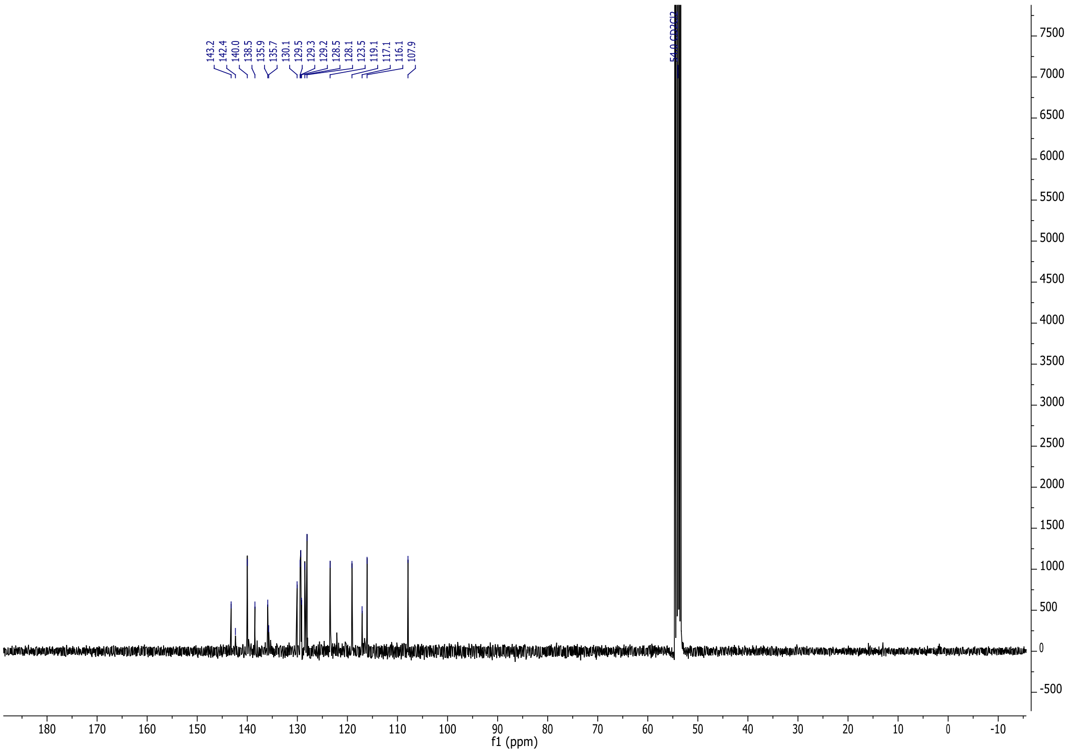

^{19}F NMR (282 MHz, CD_2Cl_2) of **3e**

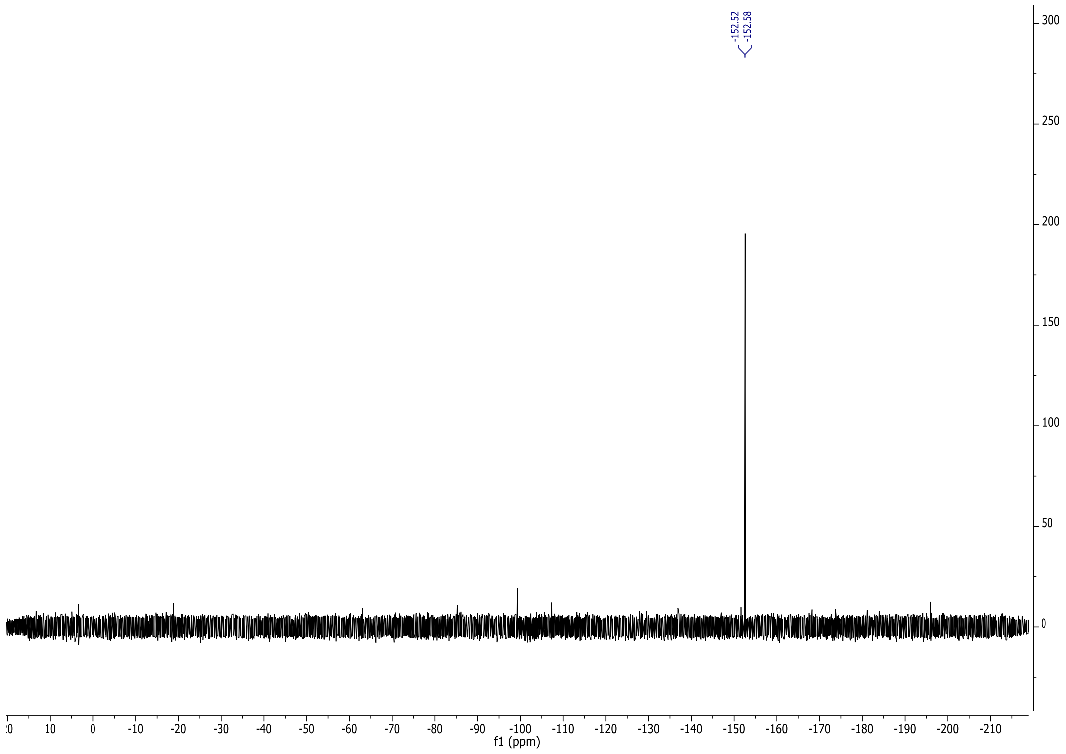

UV/VIS spectra recorded in CH_3CN (2.10^{-5} M) of **3e**

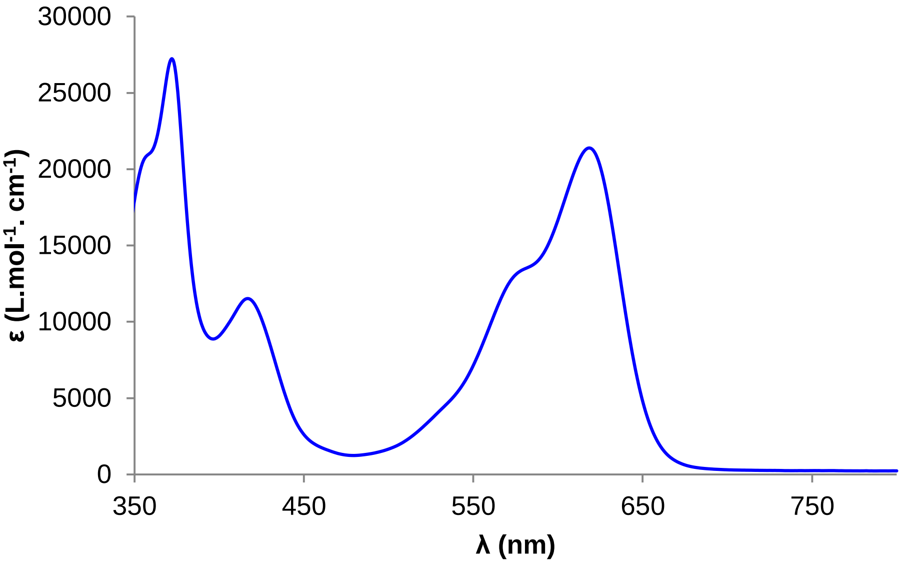

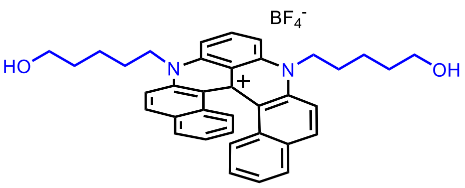

7,11-bis(5-hydroxypentyl)-7,11-dihydrobenzo[a]benzo[5,6]quinolino[2,3,4-kl]acridin-17c-ylium tetrafluoroborate **3f**

^{1}H NMR (500 MHz, CD_2Cl_2) of **3f**

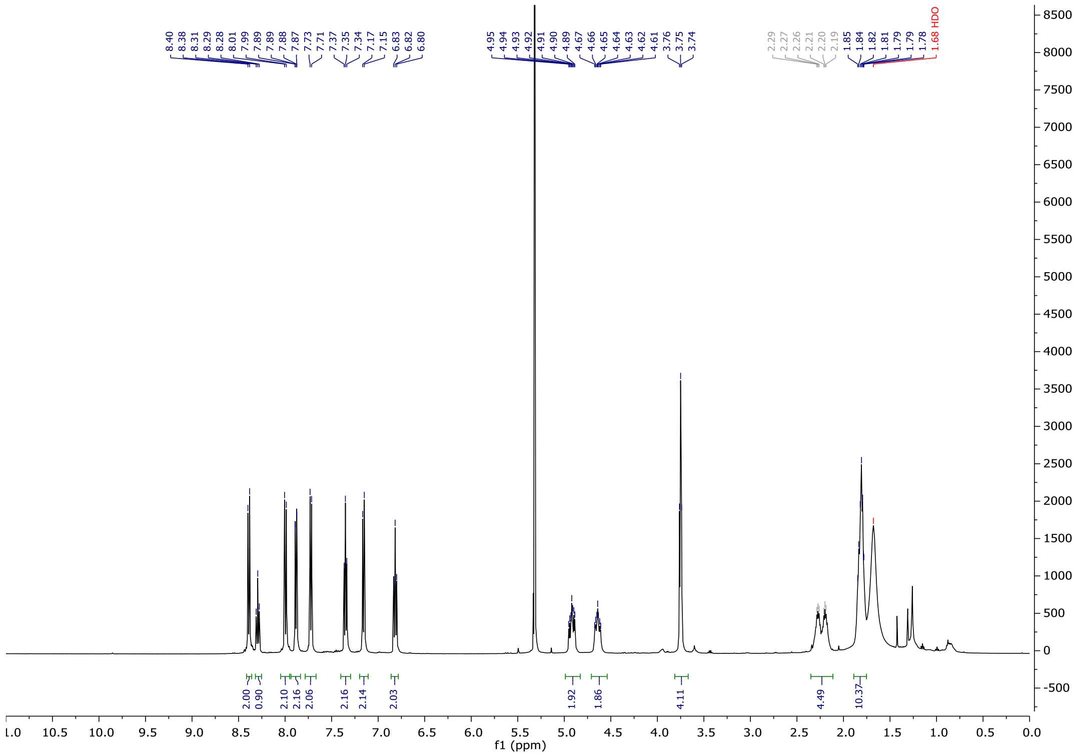

^{13}C NMR (125 MHz, CD_2Cl_2) of **3f**

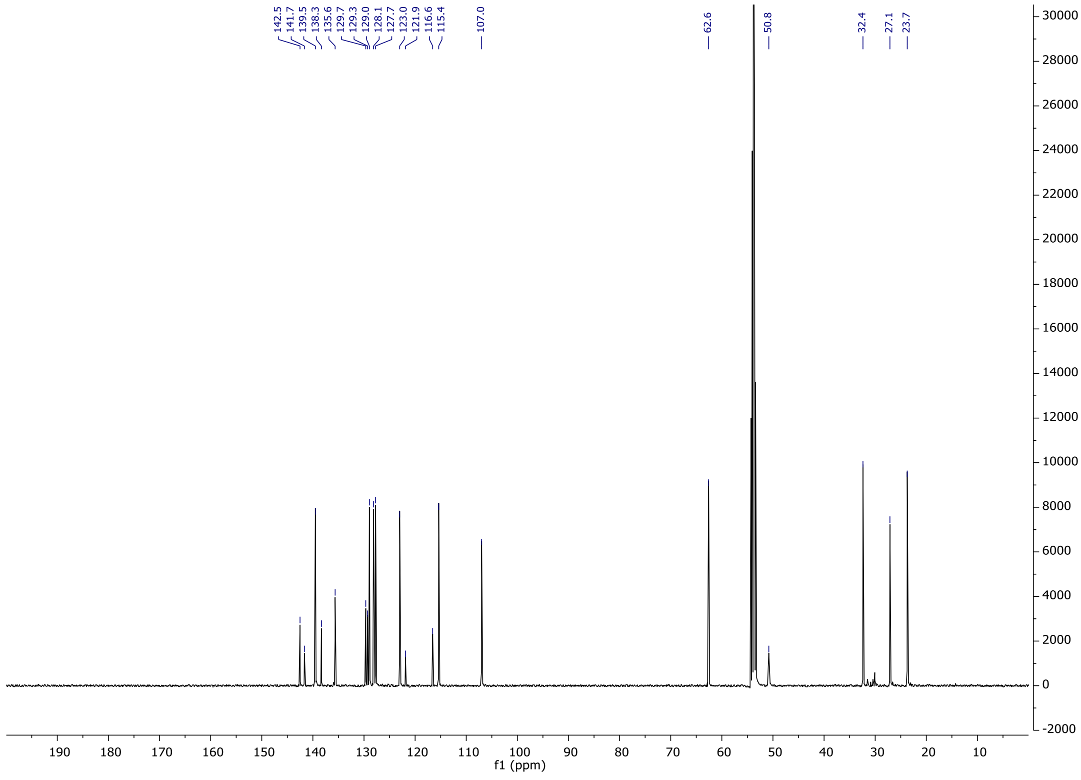

^{19}F NMR (282 MHz, CD_2Cl_2) of **3f**

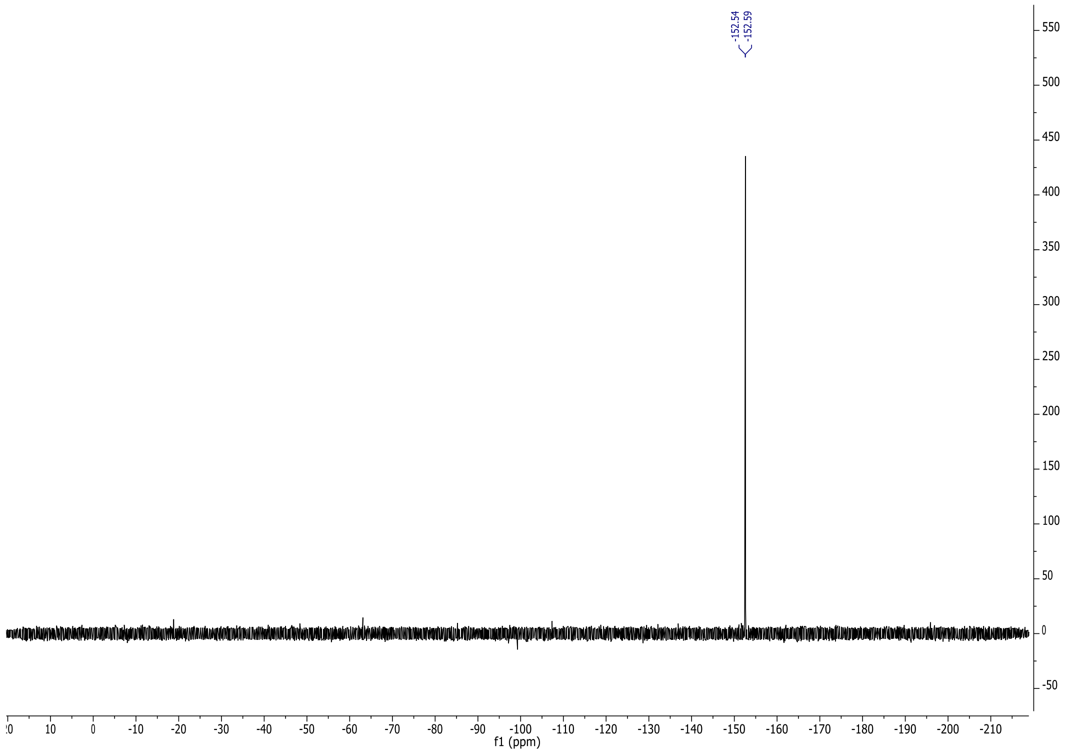

UV/VIS spectra recorded in CH_3CN (2.10^{-5} M) of **3f**

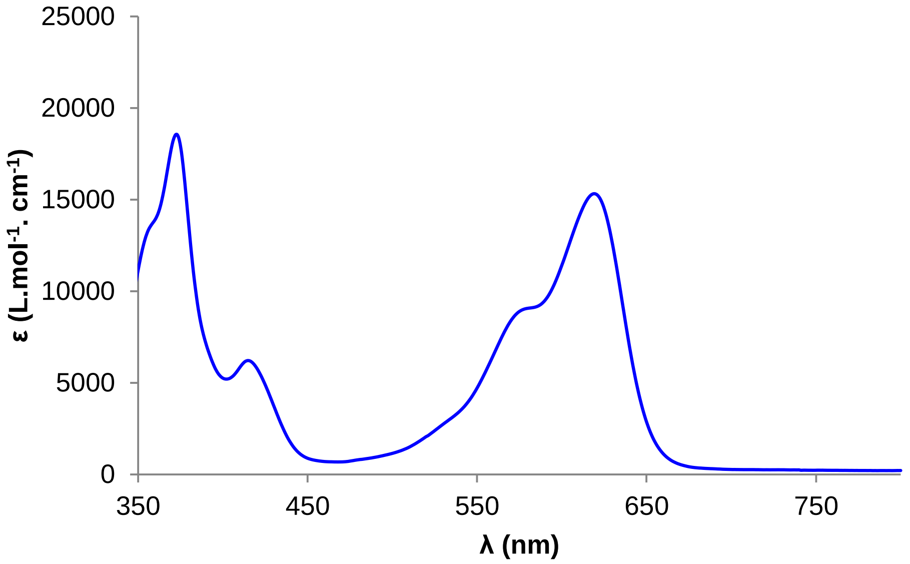

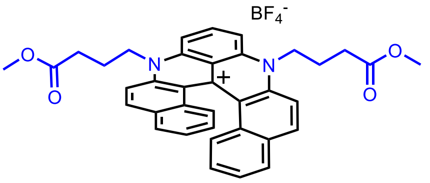

7,11-bis(4-methoxy-4-oxobutyl)-7,11-dihydro-17cH-benzo[a]benzo[5,6]quinolino[2,3,4-kl]acridin-17c-ylium tetrafluoroborate **3h**

^{1}H NMR (500 MHz, CD_2Cl_2) of **3h**

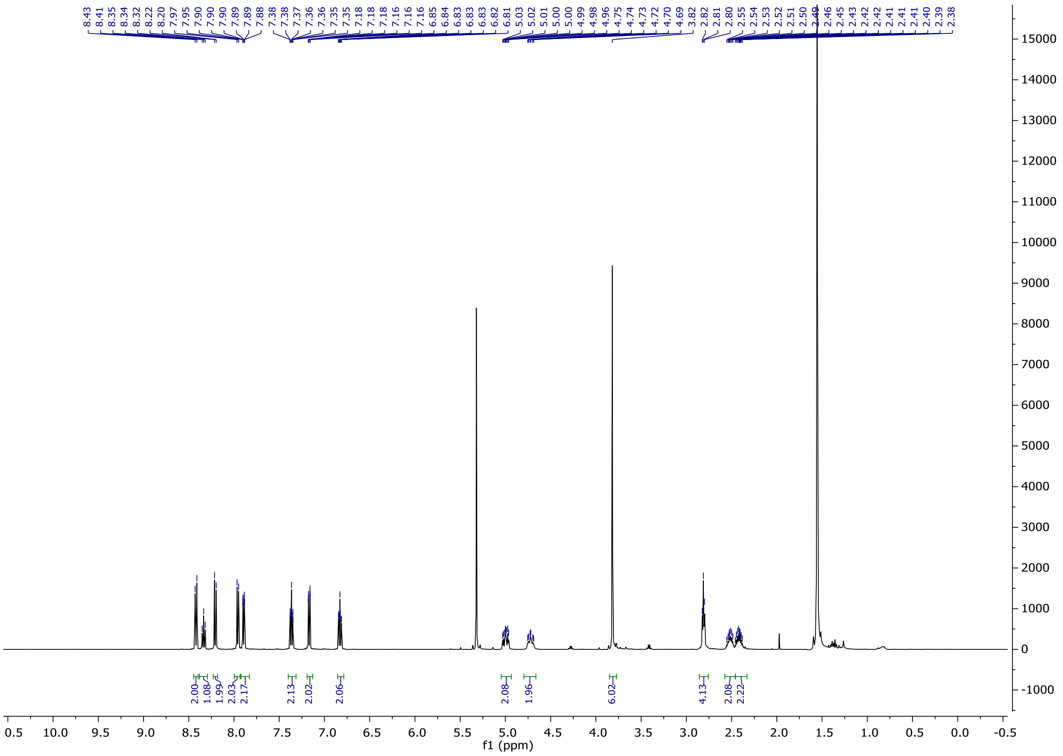

^{13}C NMR (125 MHz, CD_2Cl_2) of **3h**

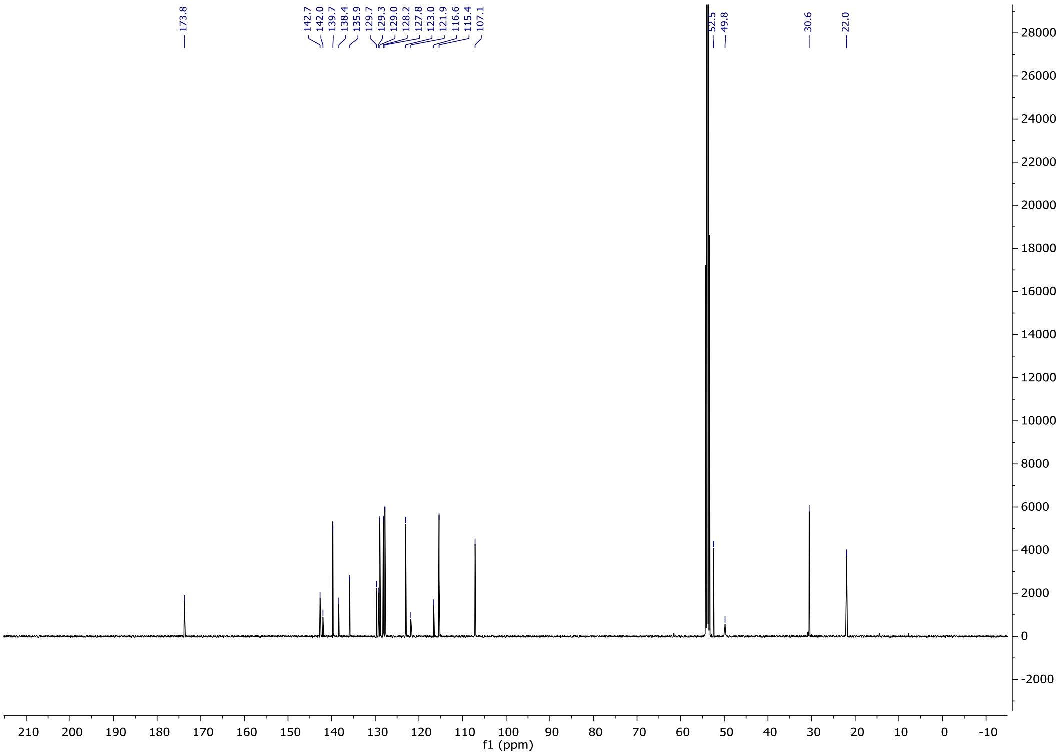

^{19}F NMR (282 MHz, CD_2Cl_2) of **3h**

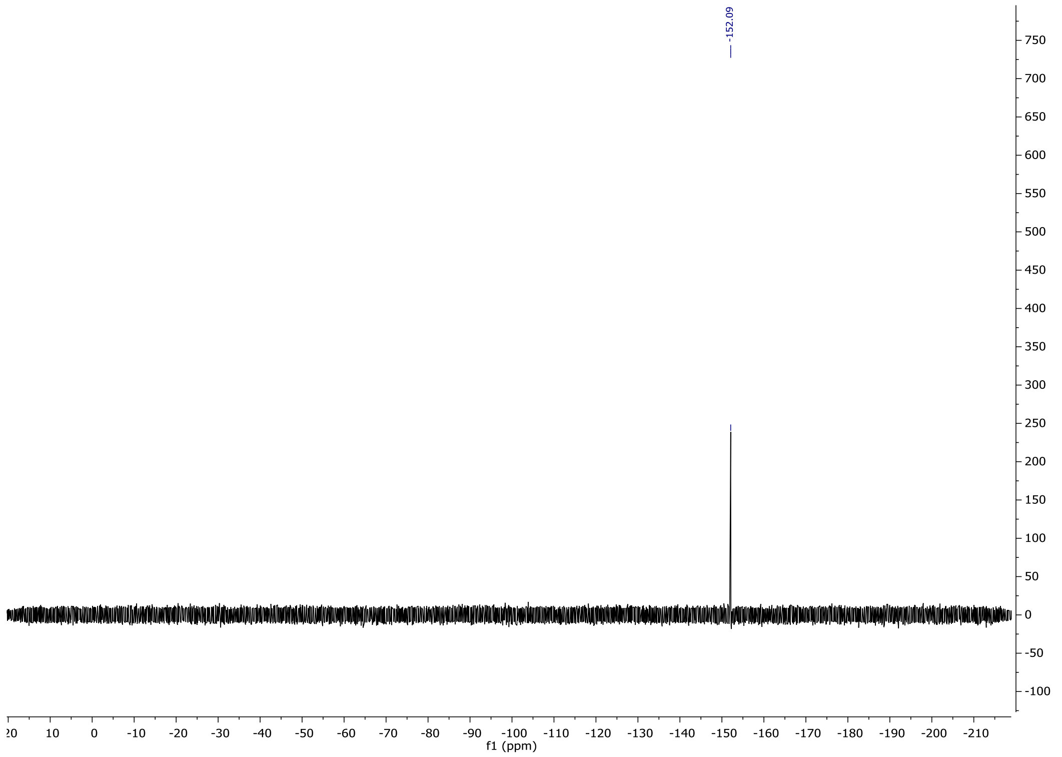

UV/VIS spectra recorded in CH_3CN (2.10^{-5} M) of **3h**

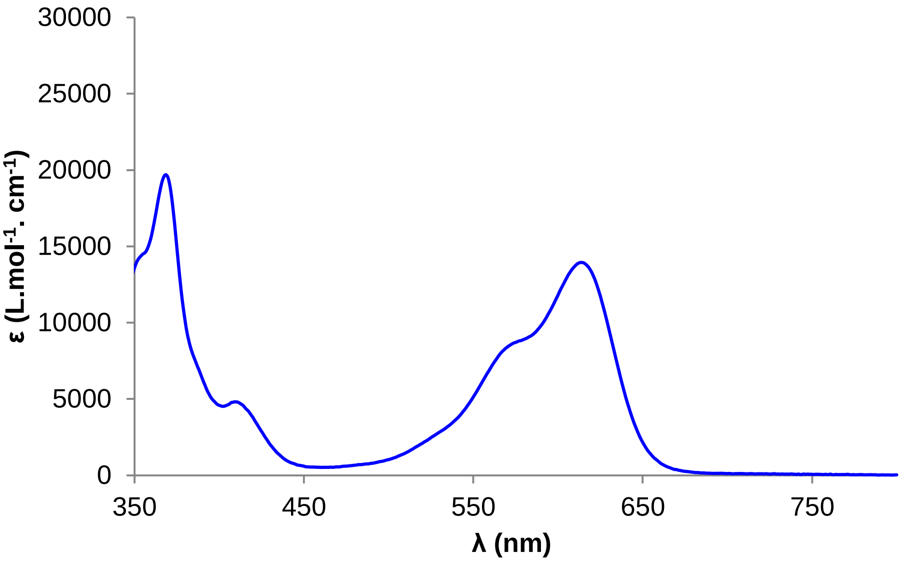

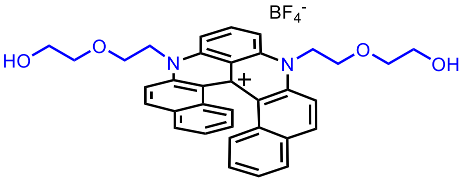

7,11-bis(2-(2-hydroxyethoxy)ethyl)-7,11-dihydrobenzo[a]benzo[5,6]quinolino[2,3,4-kl]acridin-17c-ylium tetrafluoroborate **3i**

^{1}H NMR (500 MHz, CD_2Cl_2) of **3i**

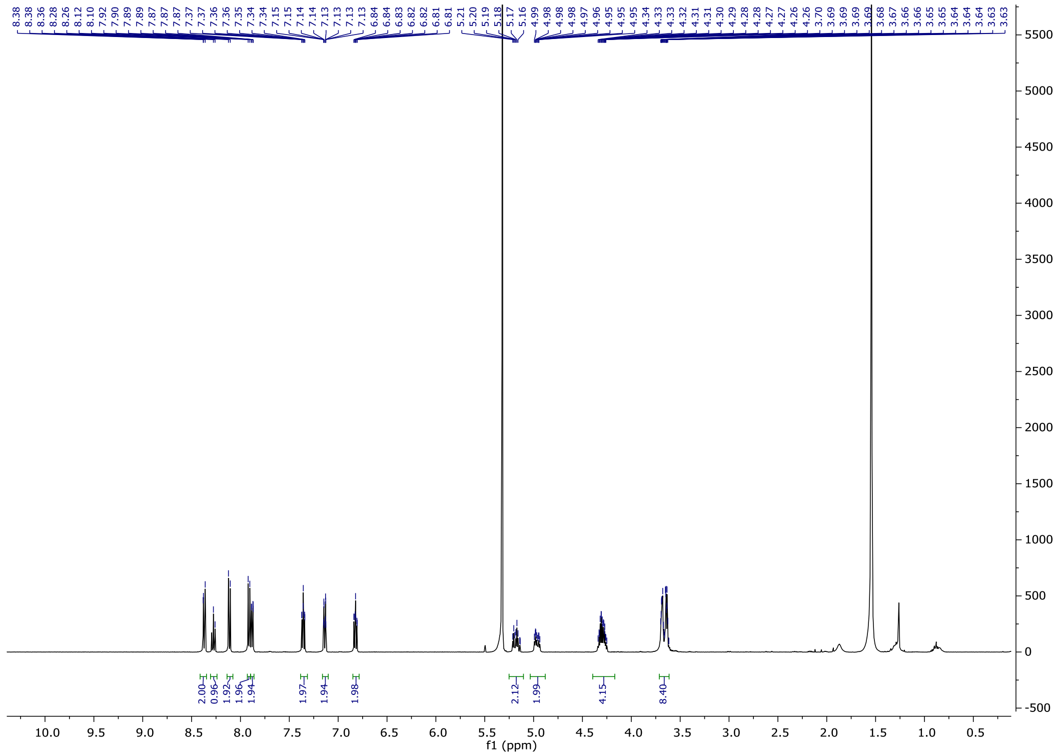

^{13}C NMR (125 MHz, CD_2Cl_2) of **3i**

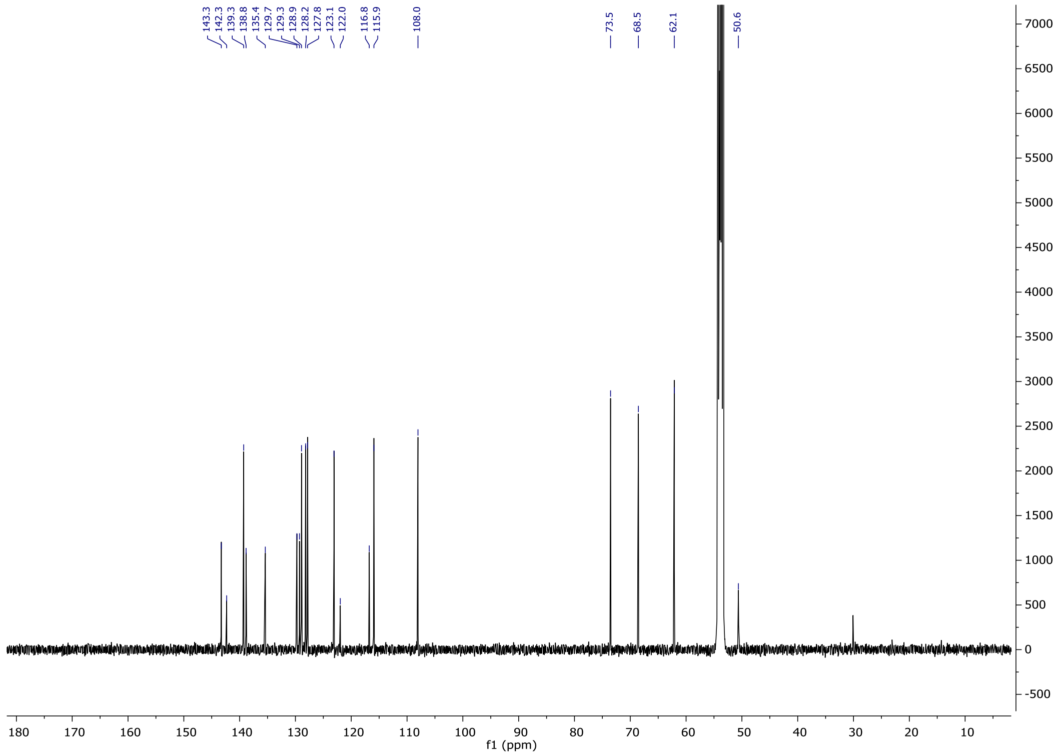

^{19}F NMR (282 MHz, CD_2Cl_2) of **3i**

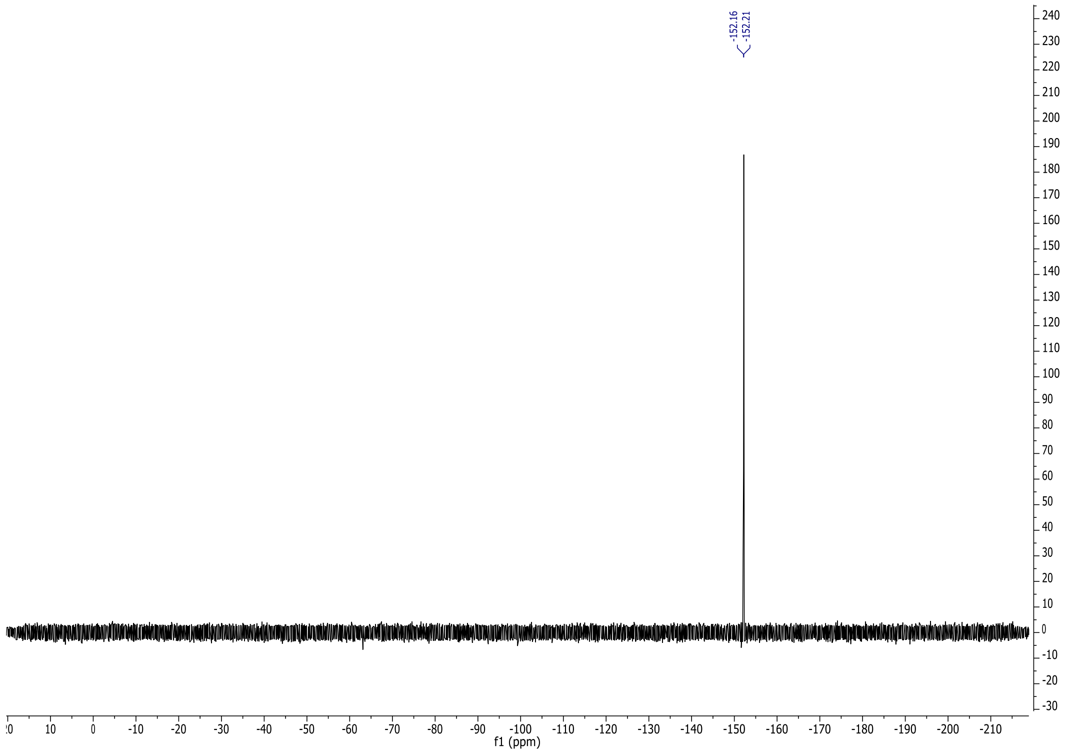

UV/VIS spectra recorded in CH_3CN (2.10^{-5} M) of **3i**

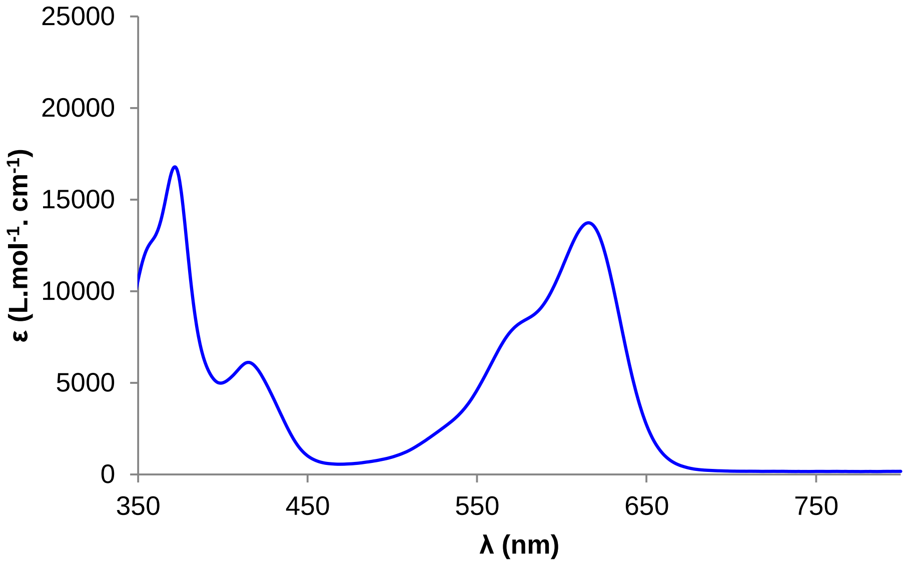

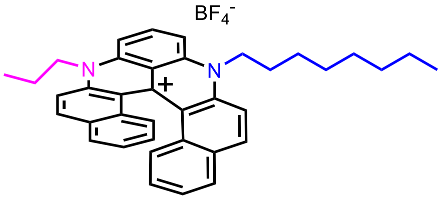

7-octyl-11-propyl-7,11-dihydrobenzo[a]benzo[5,6]quinolino[2,3,4-kl]acridin-17c-ylium tetrafluoroborate **3j**

^{1}H NMR (500 MHz, CD_2Cl_2) of **3j**

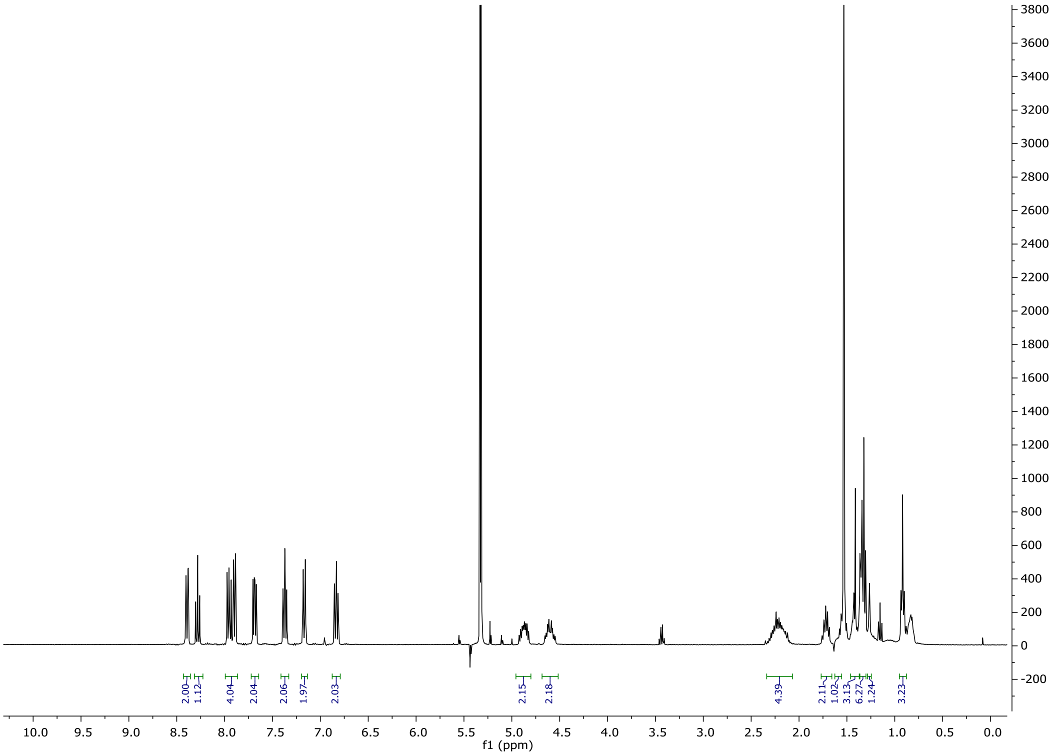

^{13}C NMR (125 MHz, CD_2Cl_2) of **3j**

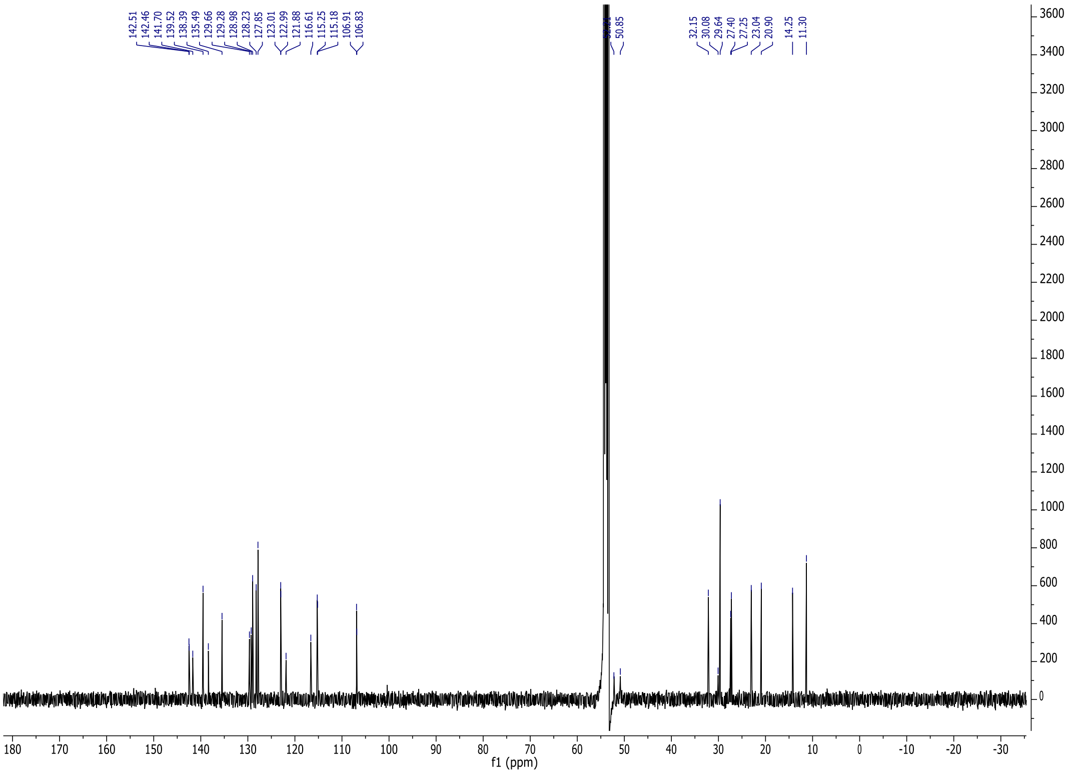

^{19}F NMR (282 MHz, CD_2Cl_2) of **3j**

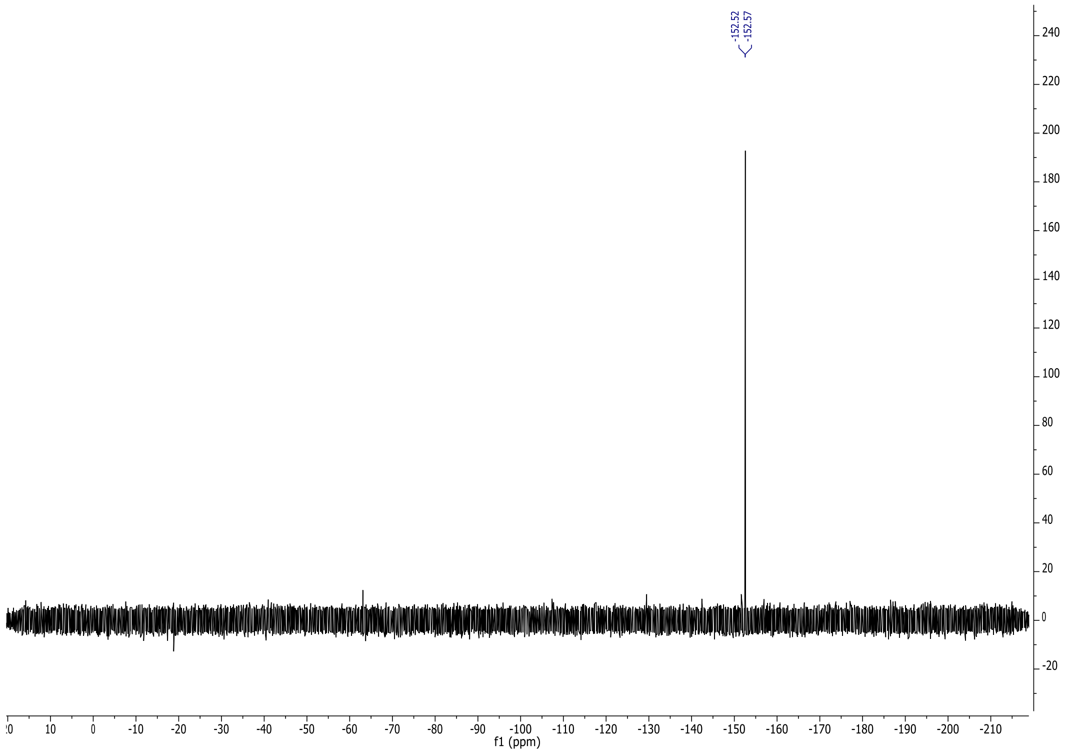

UV/VIS spectra recorded in CH_3CN (2.10^{-5} M) of **3j**

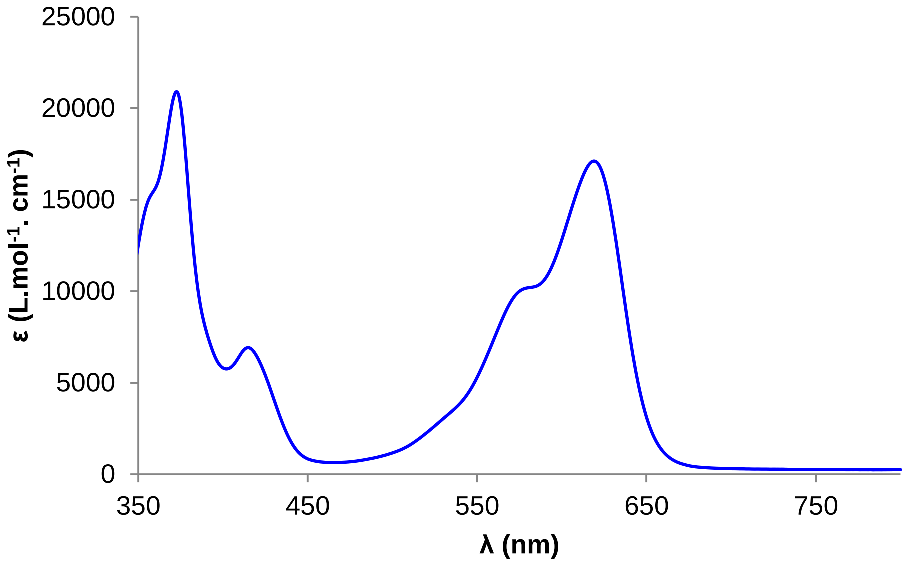

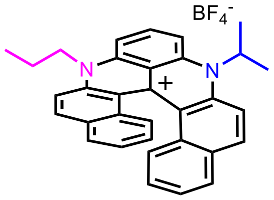

<u>7-isopropyl-11-propyl-7,11-dihydrobenzo[a]benzo[5,6]quinolino[2,3,4-kl]acridin-17c-ylium tetrafluoroborate **3k**</u>

^{1}H NMR (500 MHz, CD_2Cl_2) of **3k**

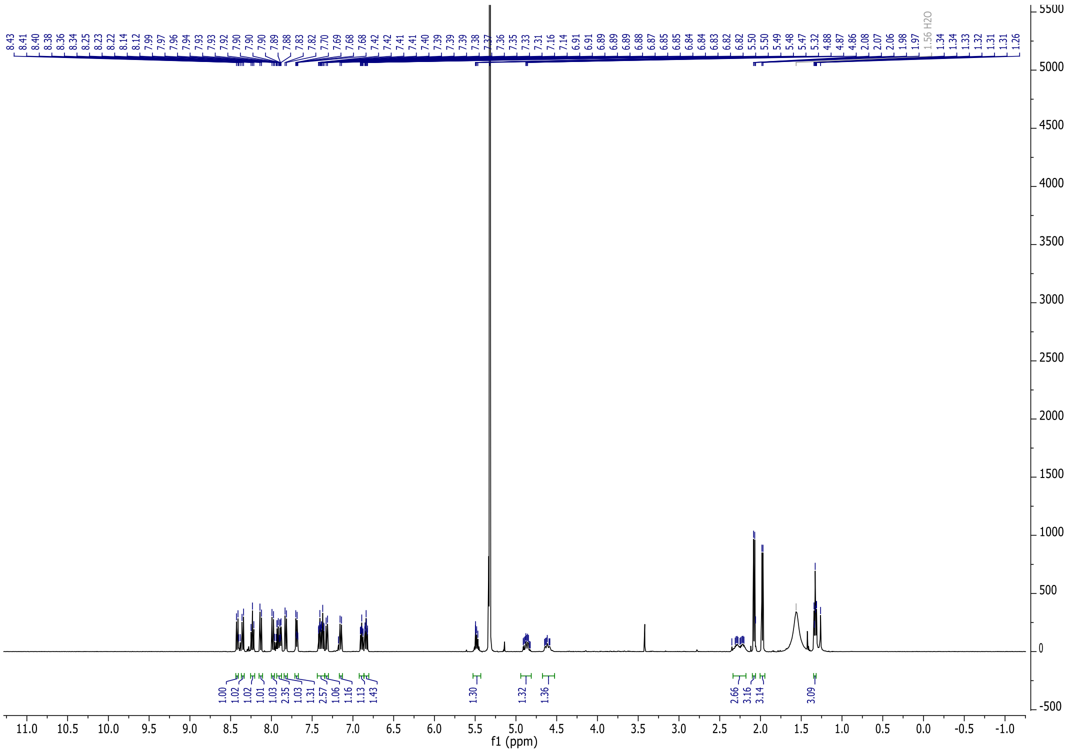

^{13}C NMR (125 MHz, CD_2Cl_2) of **3k**

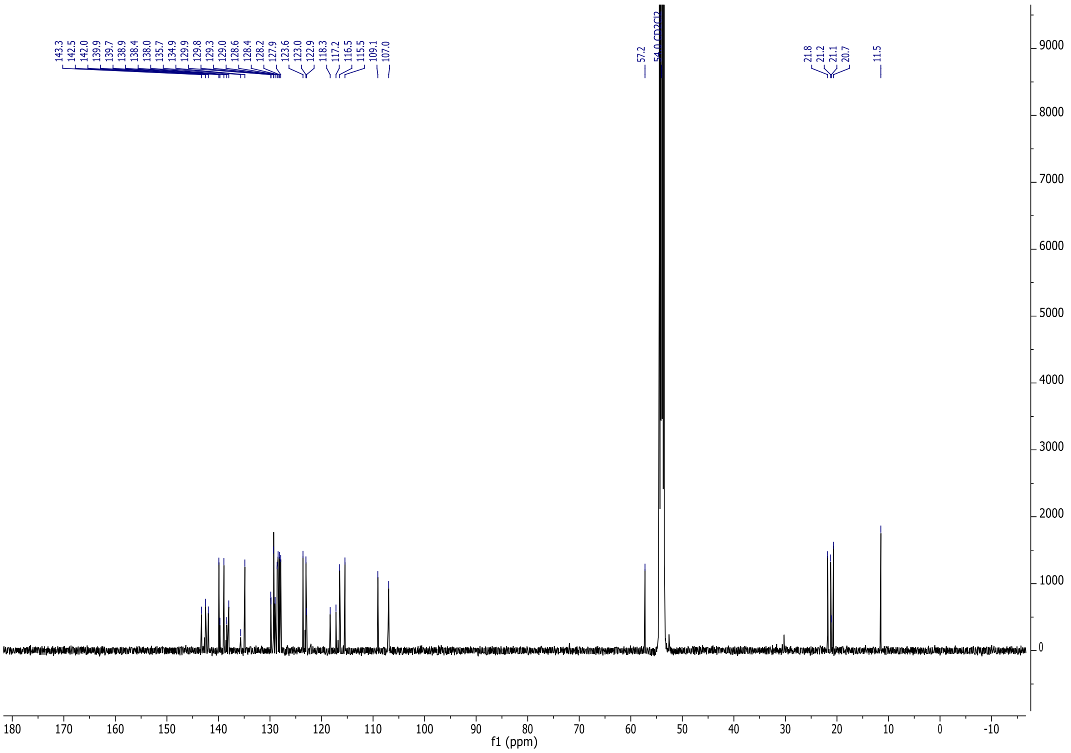

^{19}F NMR (282 MHz, CD_2Cl_2) of **3k**

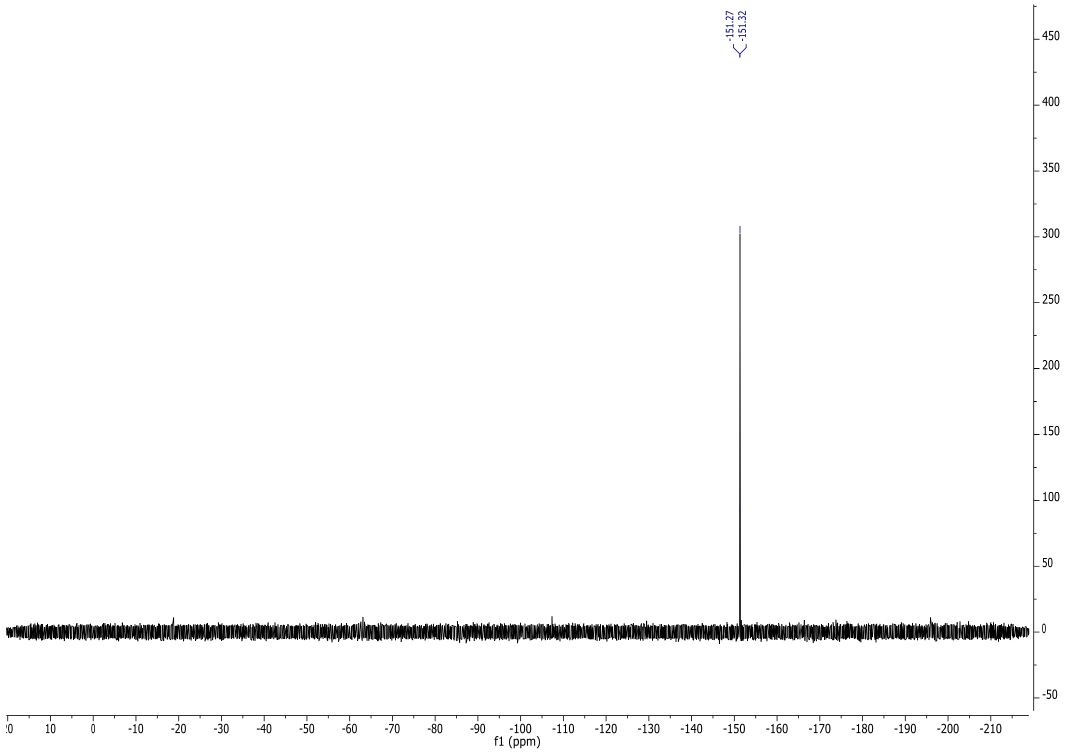

UV/VIS spectra recorded in CH_3CN (2.10^{-5} M) of **3k**

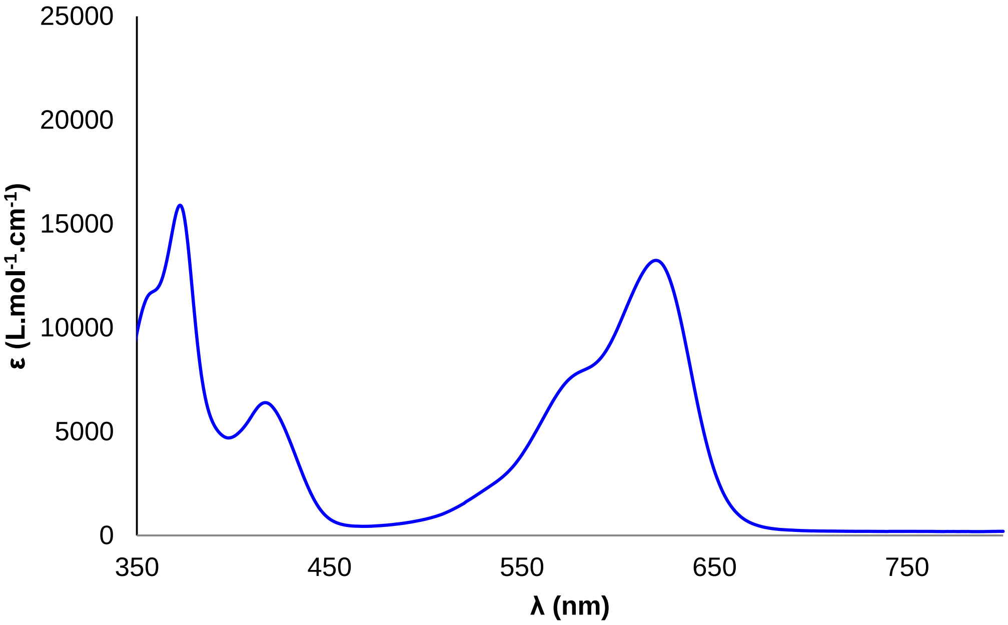

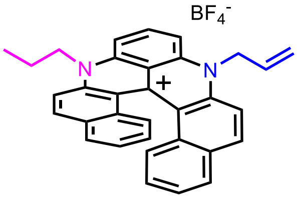

7-allyl-11-propyl-7,11-dihydro-17cH-benzo[a]benzo[5,6]quinolino[2,3,4-kl]acridin-17c-ylium tetrafluoroborate **3l**

^{1}H NMR (500 MHz, CD_2Cl_2) of **3l**

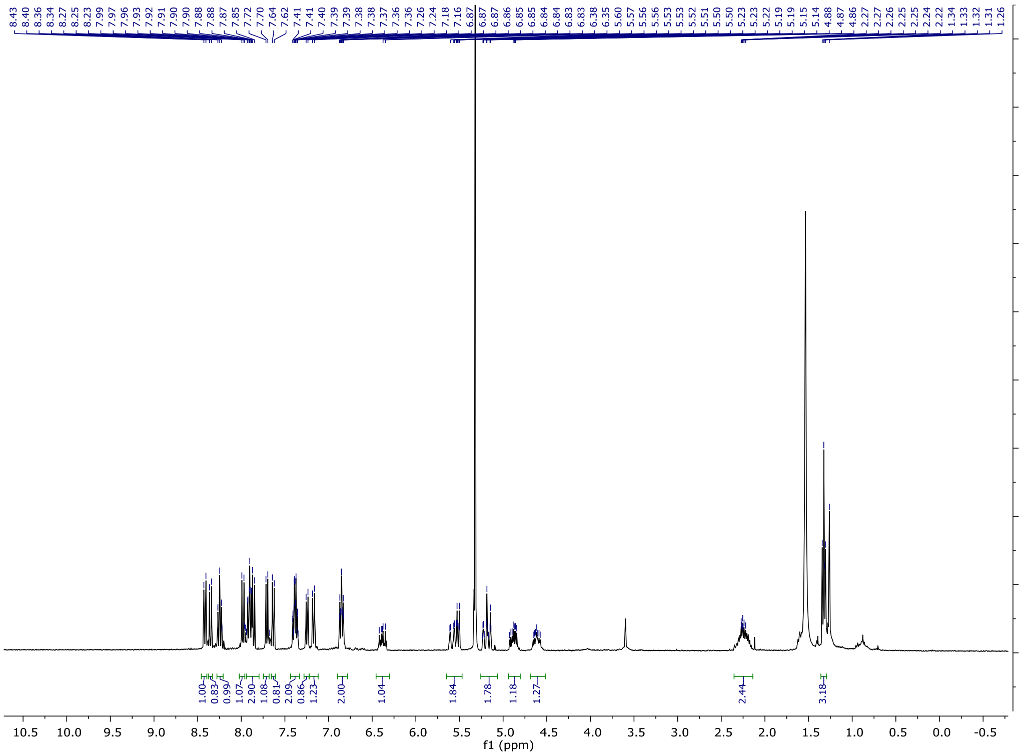

^{13}C NMR (125 MHz, CD_2Cl_2) of **3l**

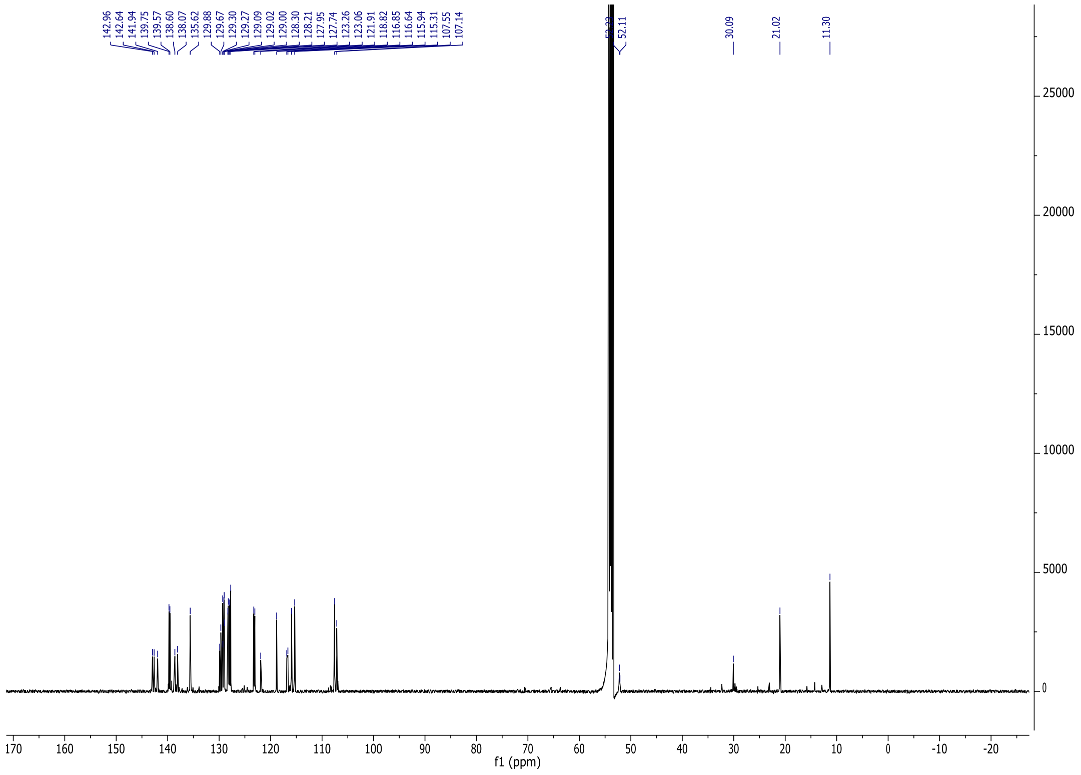

^{19}F NMR (282 MHz, CD_2Cl_2) of **3l**

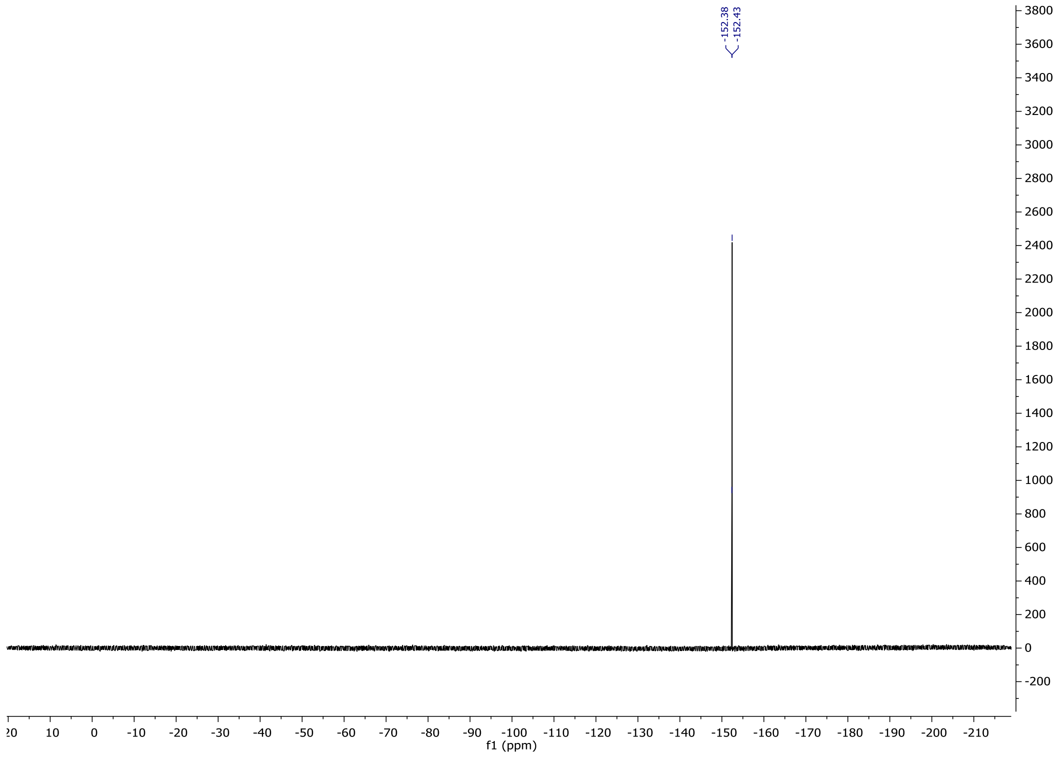

UV/VIS spectra recorded in CH_3CN (2.10^{-5} M) of **3l**

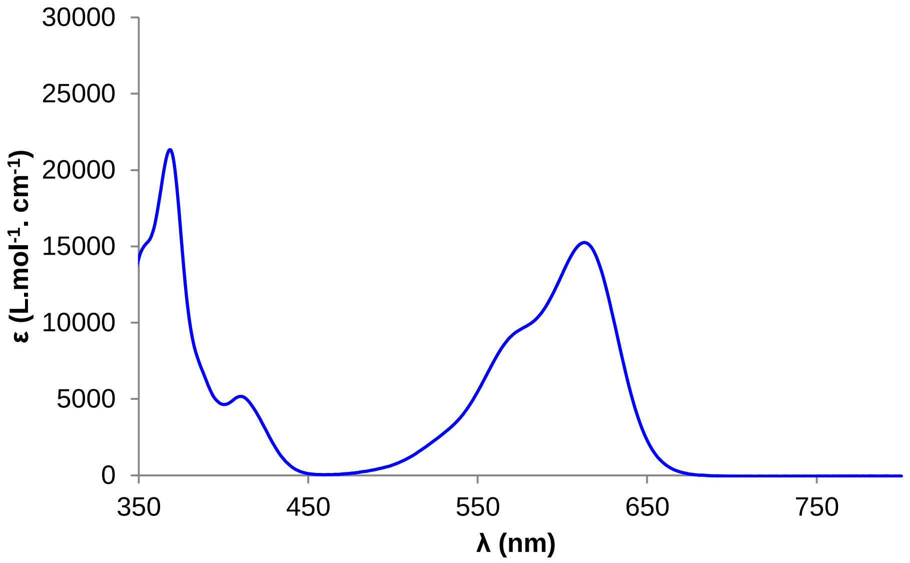

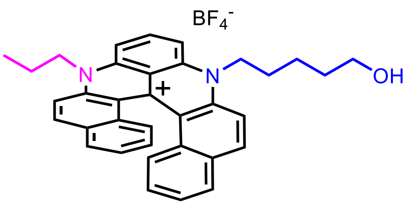

7-(5-hydroxypentyl)-11-propyl-7,11-dihydro-17cH-benzo[a]benzo[5,6]quinolino[2,3,4-kl]acridin-17c-ylium tetrafluoroborate **3m**

^{1}H NMR (500 MHz, CD_2Cl_2) of **3m**

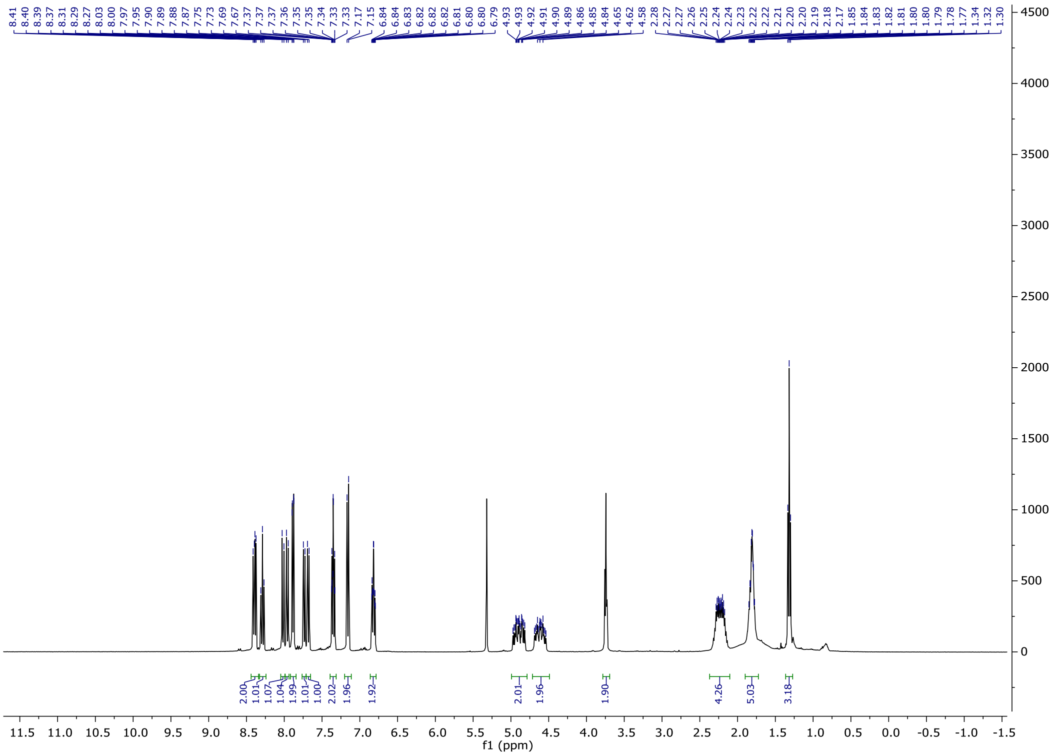

^{13}C NMR (125 MHz, CD_2Cl_2) of **3m**

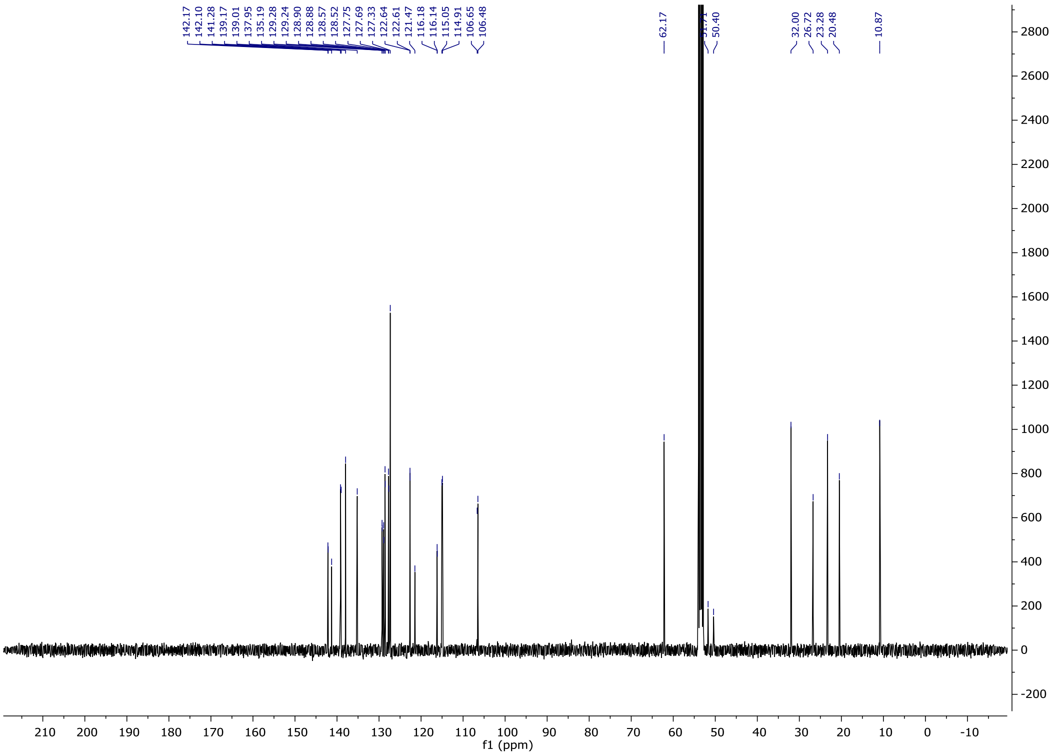

^{19}F NMR (282 MHz, CD_2Cl_2) of **3m**

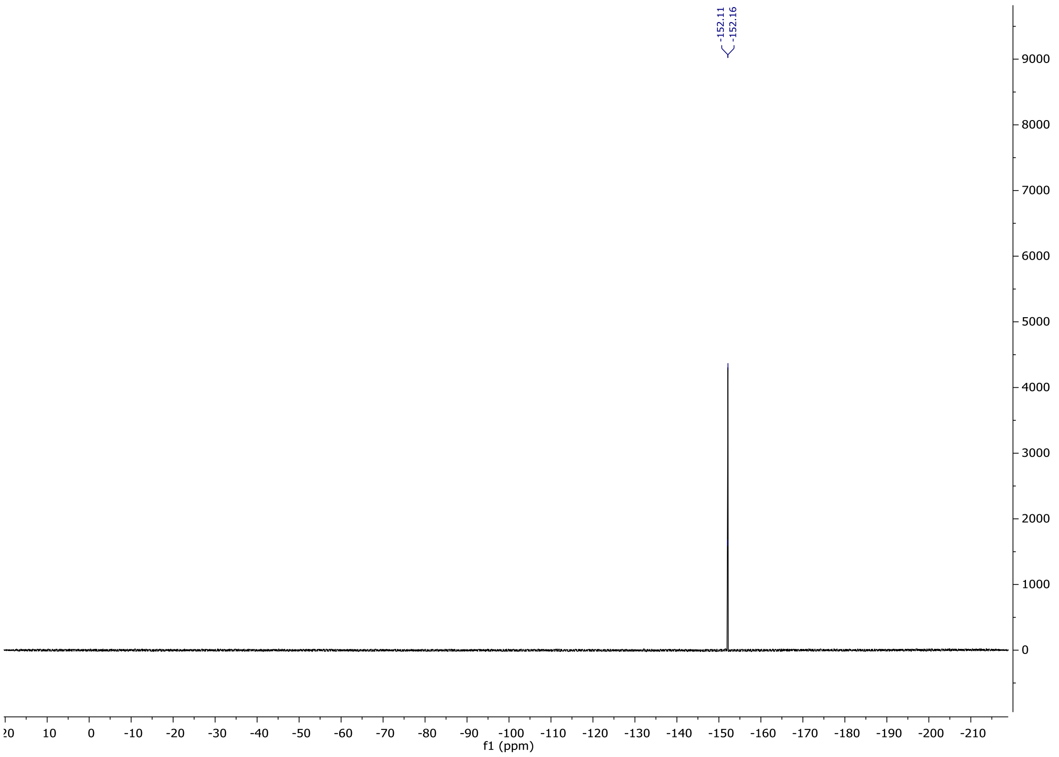

UV/VIS spectra recorded in CH_3CN (2.10^{-5} M) of **3m**

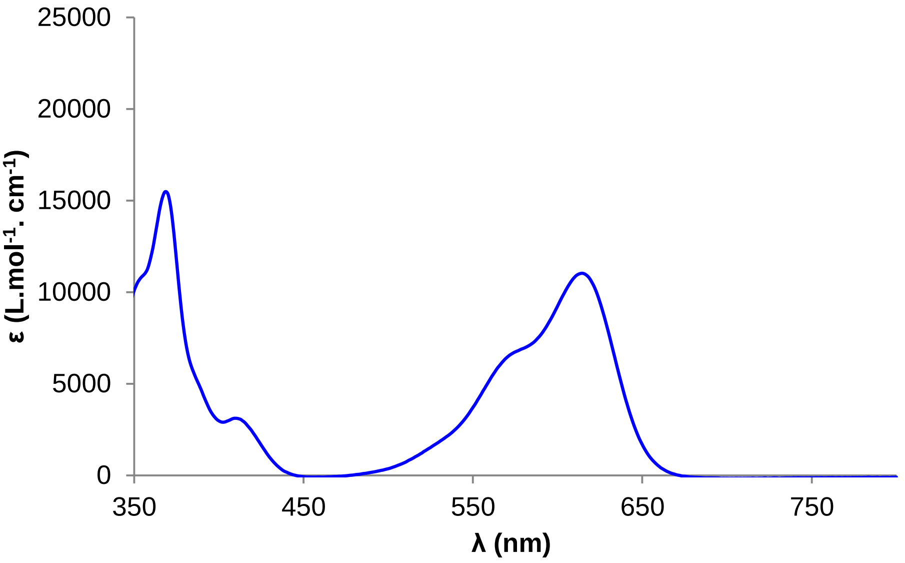

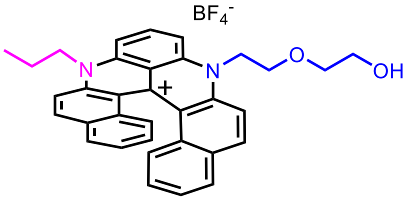

7-(2-(2-hydroxyethoxy)ethyl)-11-propyl-7,11-dihydro-17cH-benzo[a]benzo[5,6]quinolino[2,3,4-kl]acridin-17c-ylium tetrafluoroborate **3n**

^{1}H NMR (500 MHz, CD_2Cl_2) of **3n**

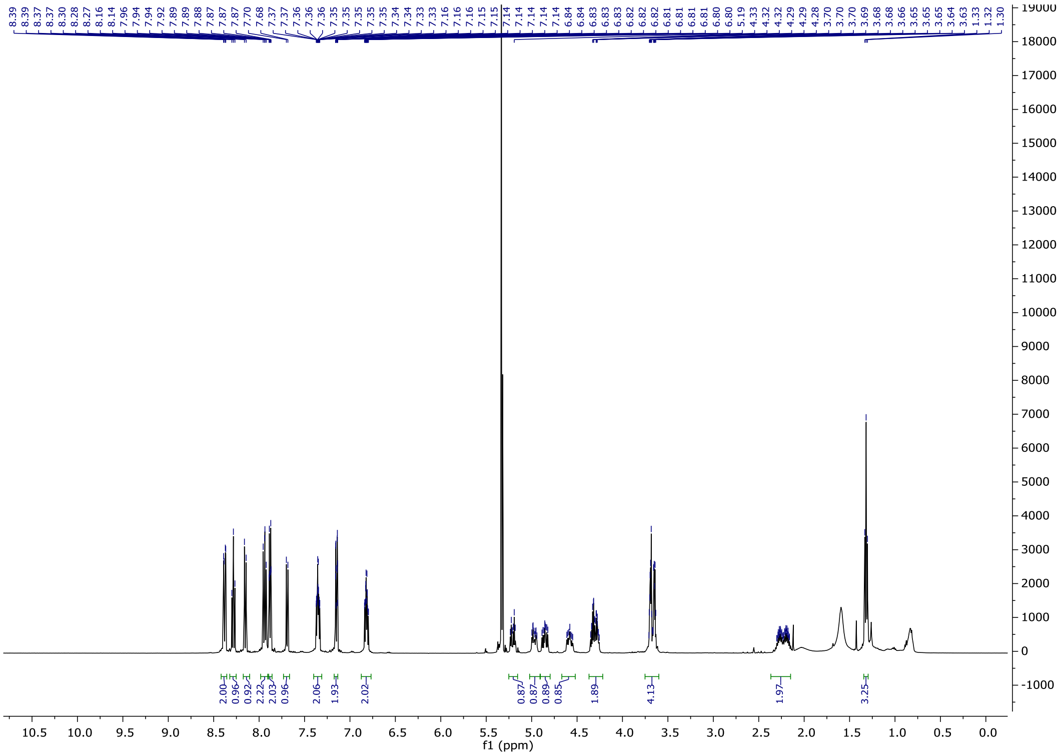

^{13}C NMR (125 MHz, CD_2Cl_2) of **3n**

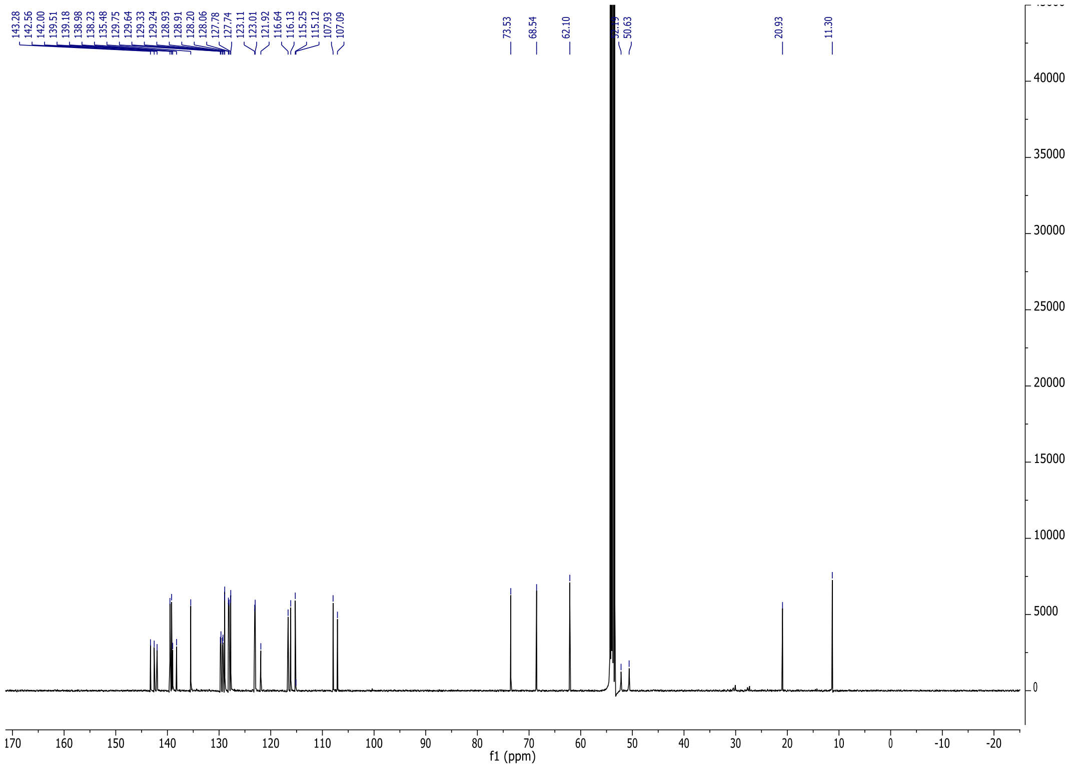

^{19}F NMR (282 MHz, CD_2Cl_2) of **3n**

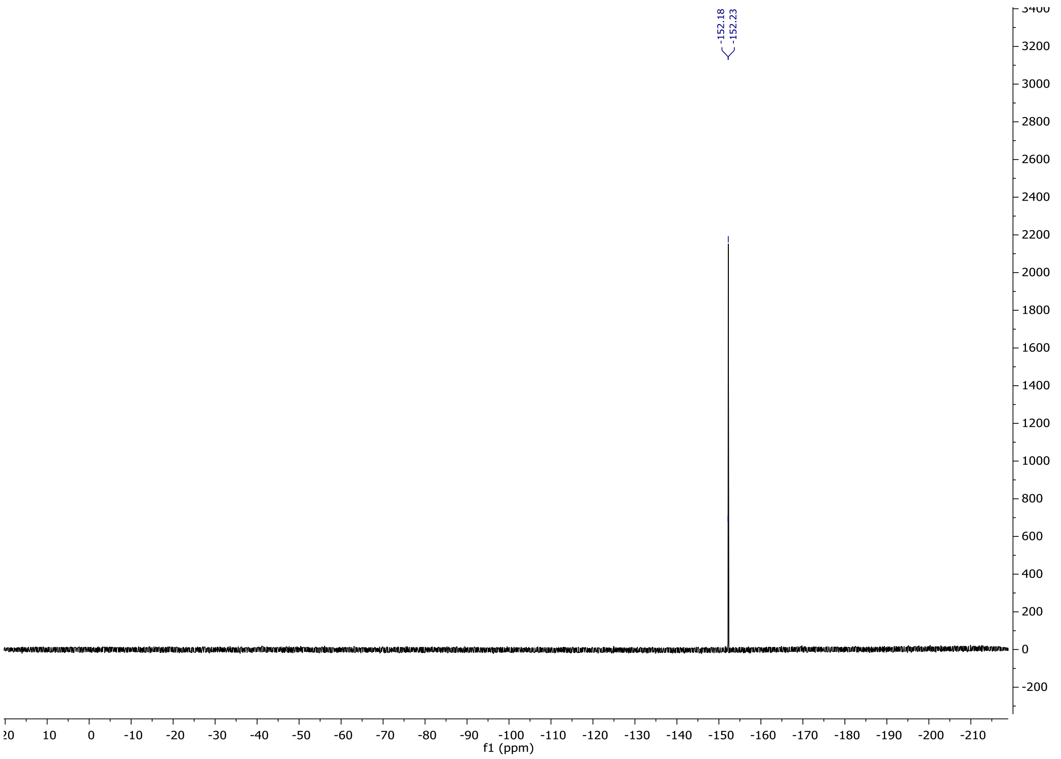

UV/VIS spectra recorded in CH_3CN (2.10^{-5} M) of **3n**

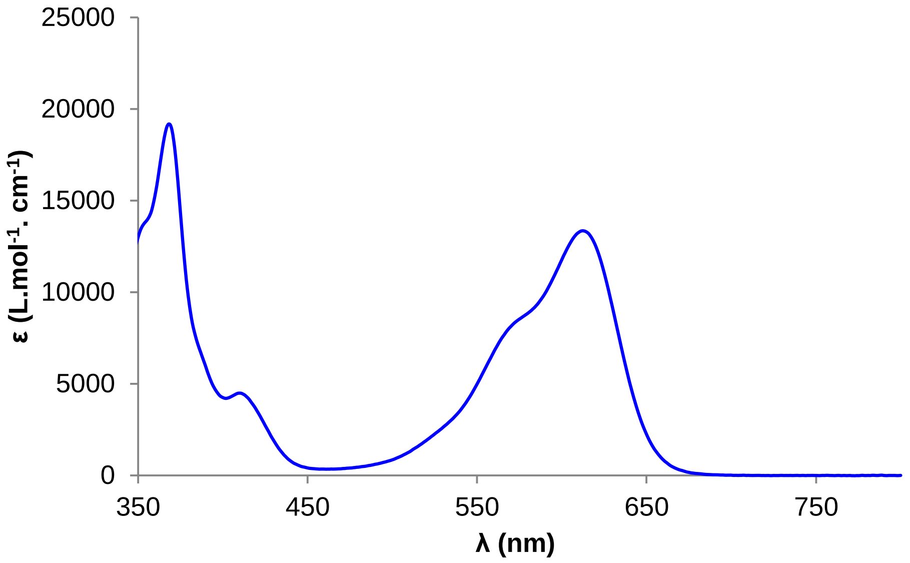

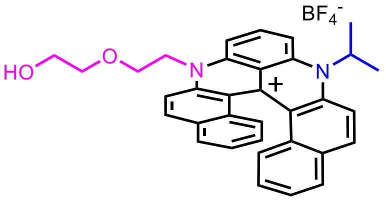

7-(2-(2-hydroxyethoxy)ethyl)-11-isopropyl-7,11-dihydro-17cH-benzo[a]benzo[5,6]quinolino[2,3,4-kl]acridin-17c-ylium tetrafluoroborate **3o**

^{1}H NMR (500 MHz, CD_2Cl_2) of **3o**

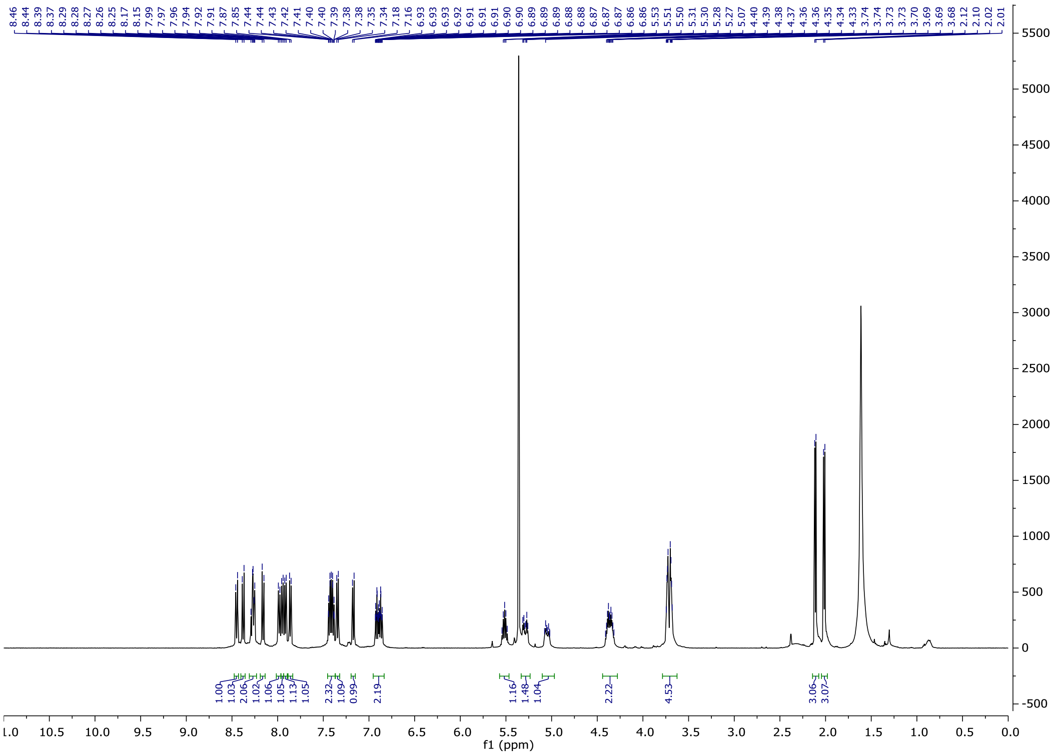

^{13}C NMR (125 MHz, CD_2Cl_2) of **3o**

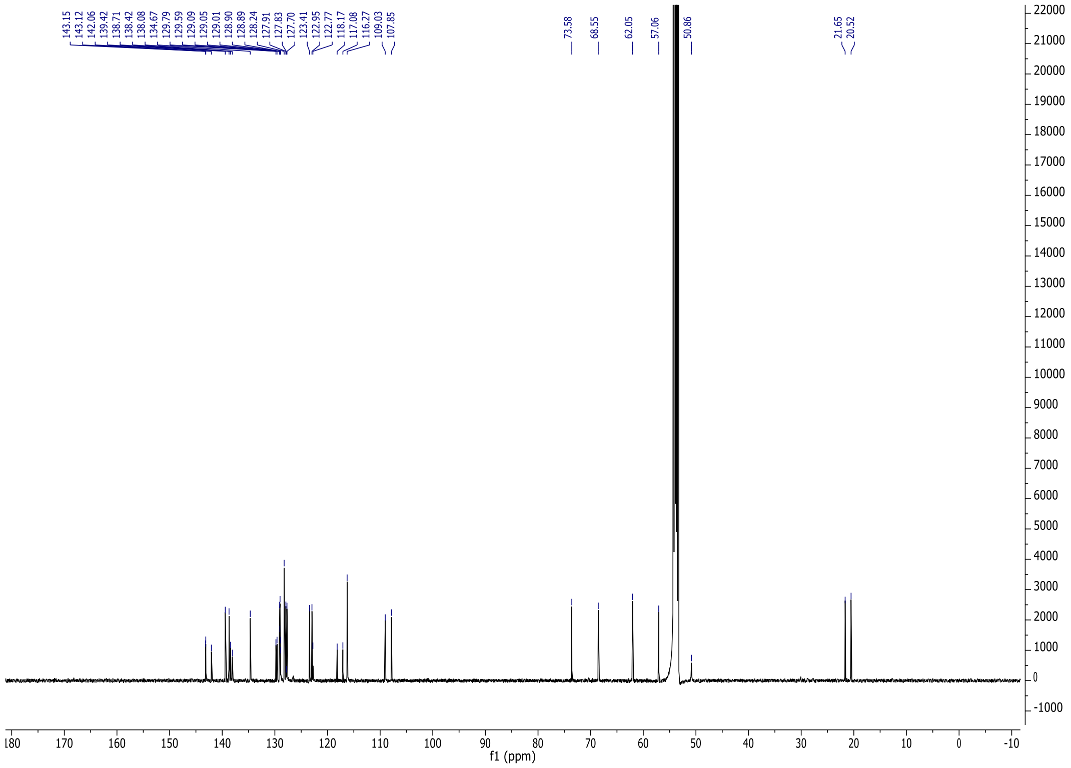

^{19}F NMR (282 MHz, CD_2Cl_2) of **3o**

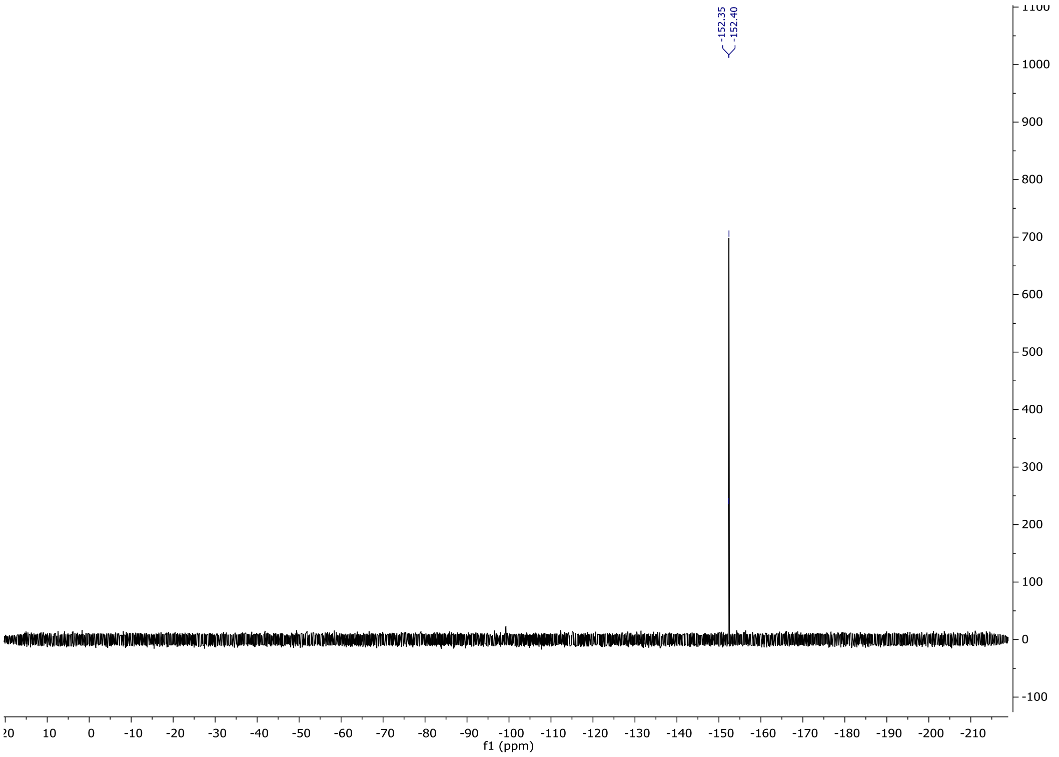

UV/VIS spectra recorded in CH_3CN (2.10^{-5} M) of **3o**

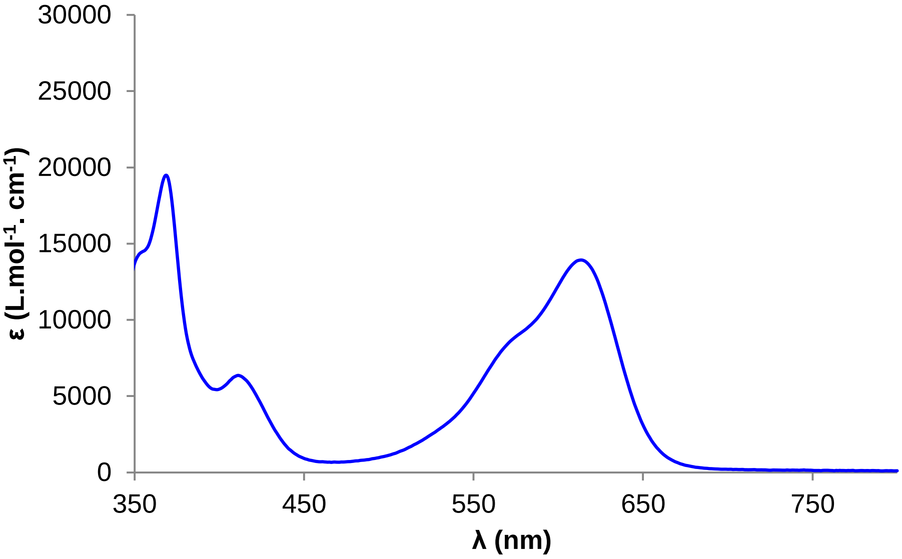

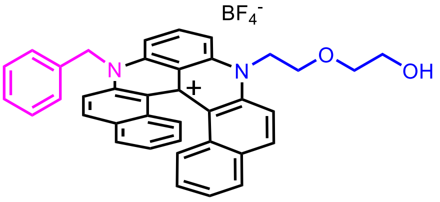

7-benzyl-11-(5-hydroxypentyl)-7,11-dihydro-17cH-benzo[a]benzo[5,6]quinolino[2,3,4-kl]acridin-17c-ylium tetrafluoroborate **3p**

^{1}H NMR (500 MHz, CD_2Cl_2) of **3p**

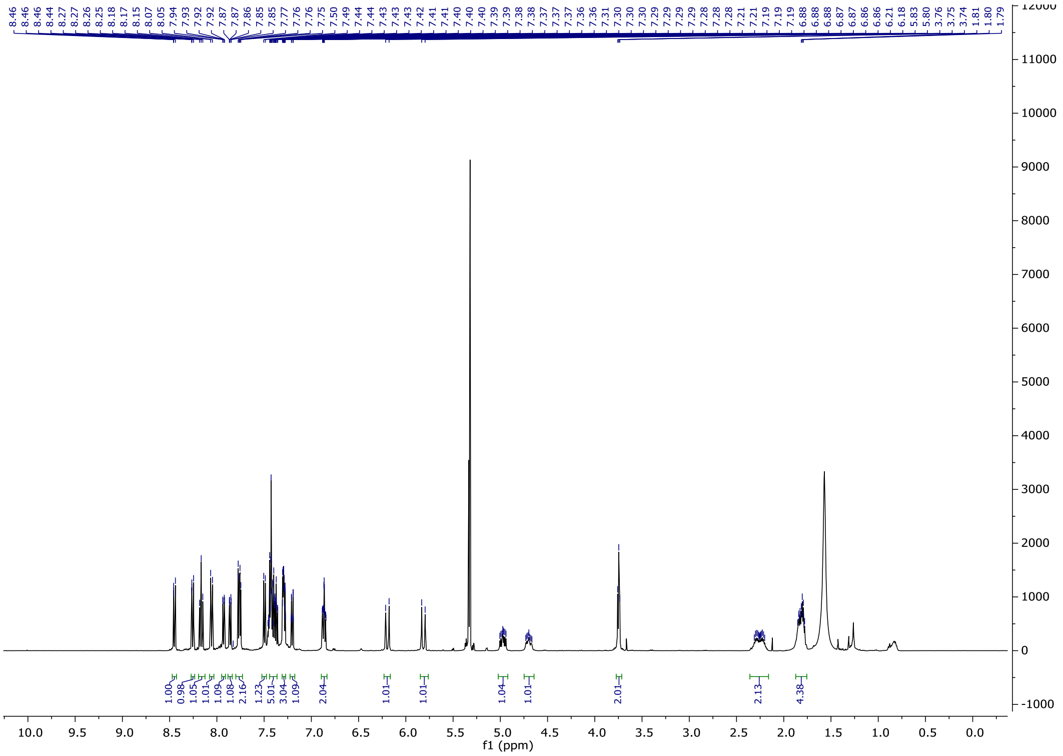

^{13}C NMR (125 MHz, CD_2Cl_2) of **3p**

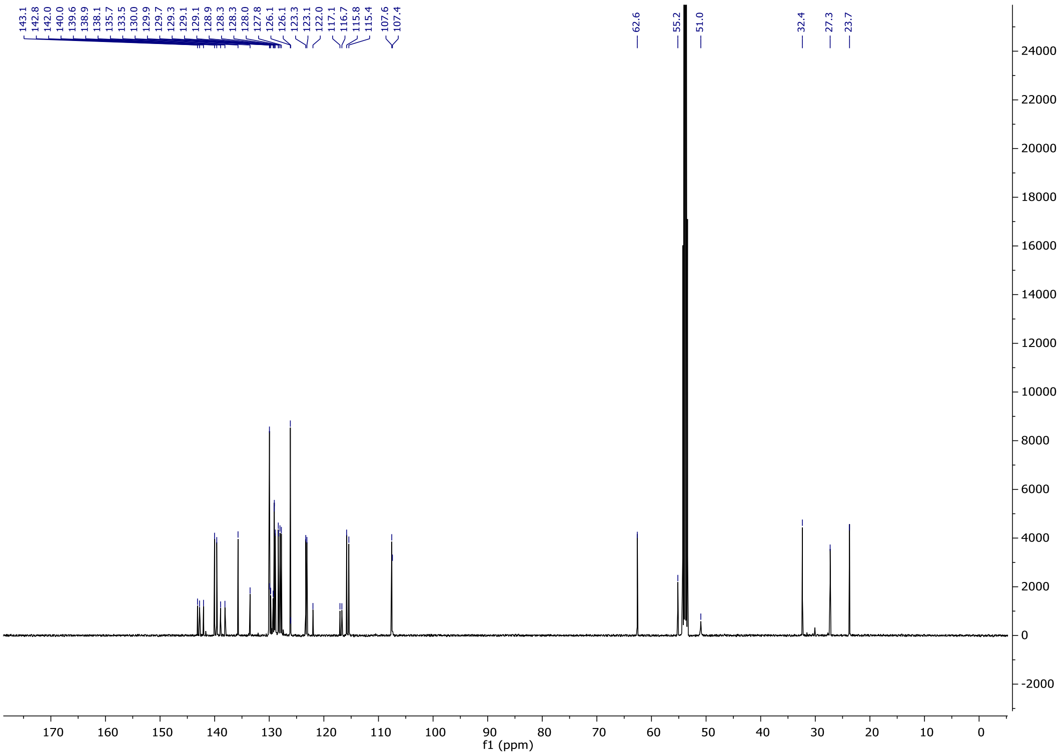

^{19}F NMR (282 MHz, CD_2Cl_2) of **3p**

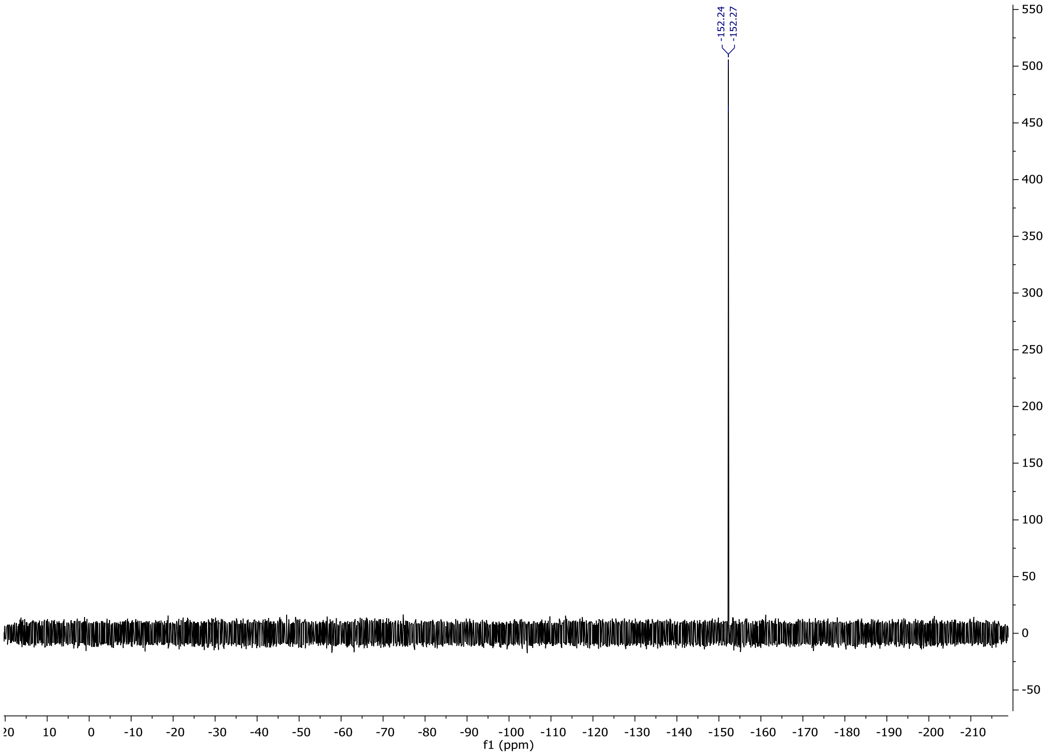

UV/VIS spectra recorded in CH_3CN (2.10^{-5} M) of **3p**

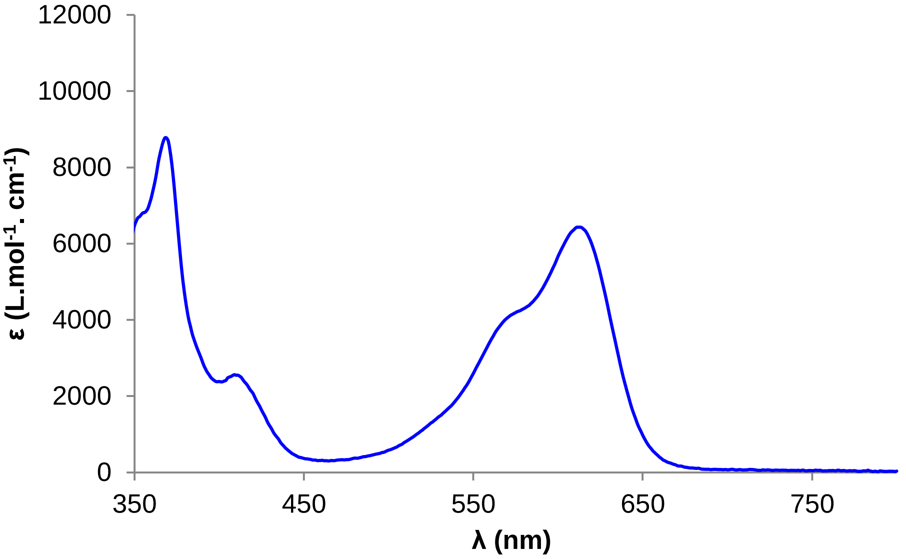

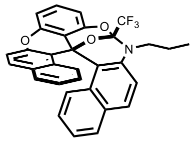

13-propyl-12-(trifluoromethyl)-12,13-dihydro-12,19c-epoxybenzo[7,8]xantheno[9,1-fg]naphtho[2,1-d][1,3]oxazocine **5**.

^{1}H NMR (500 MHz, CD_2Cl_2) of **5**

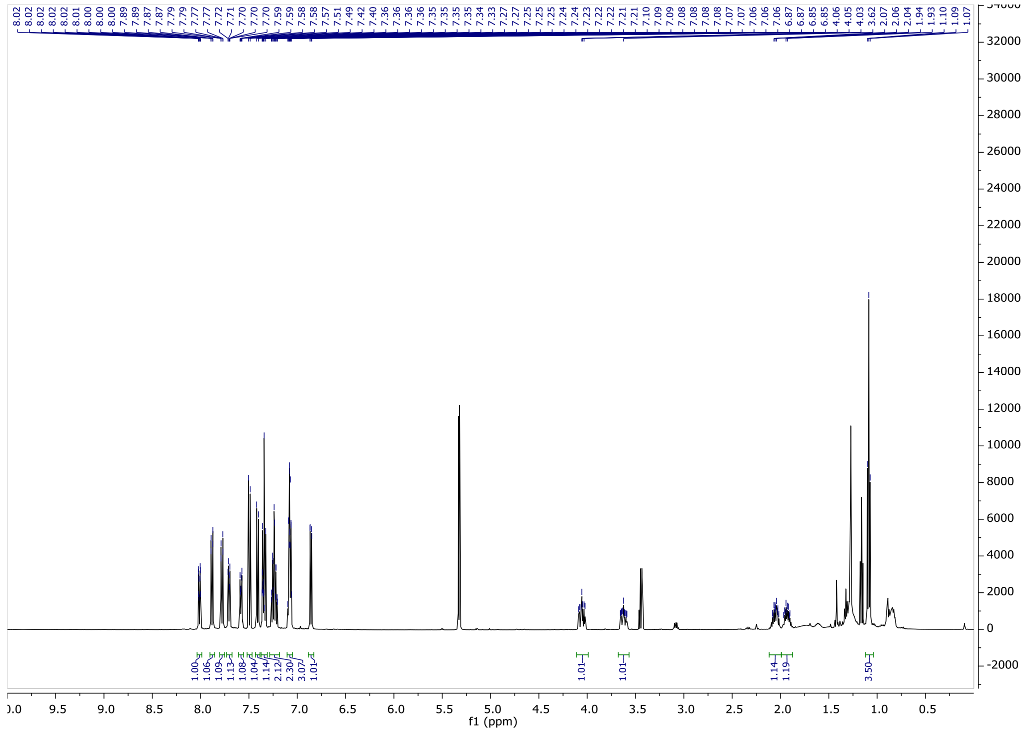

^{13}C NMR (125 MHz, CD_2Cl_2) of **5**

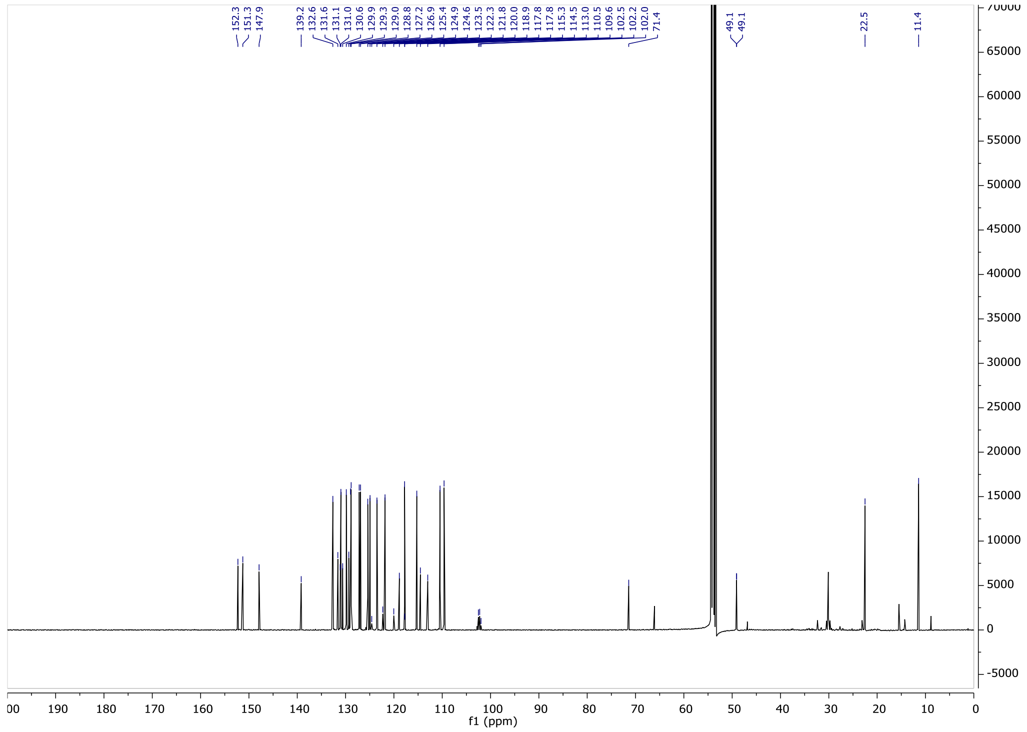

^{19}F NMR (125 MHz, CD_2Cl_2) of **5**

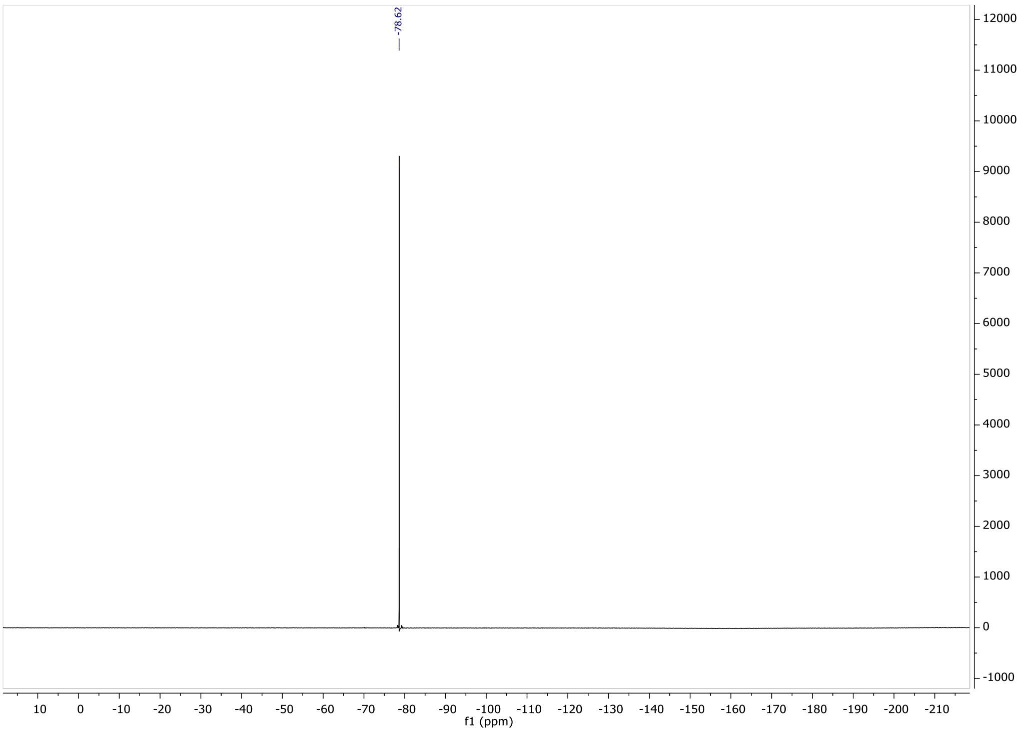

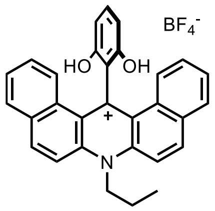

14-(2,6-dihydroxyphenyl)-7-propyl-7,14-dihydrodibenzo[a,j]acridin-14-ylium tetrafluoroborate **5**.

^{1}H NMR (500 MHz, CD_3OD) of **5**

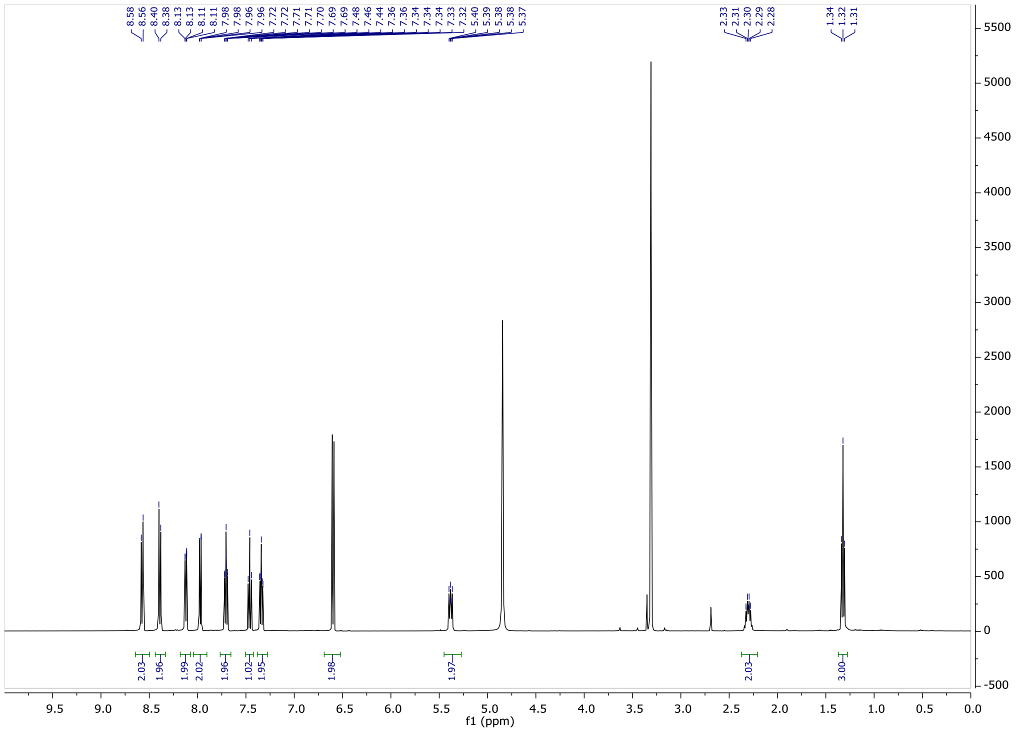

^{13}C NMR (125 MHz, CD_3OD) of **5**

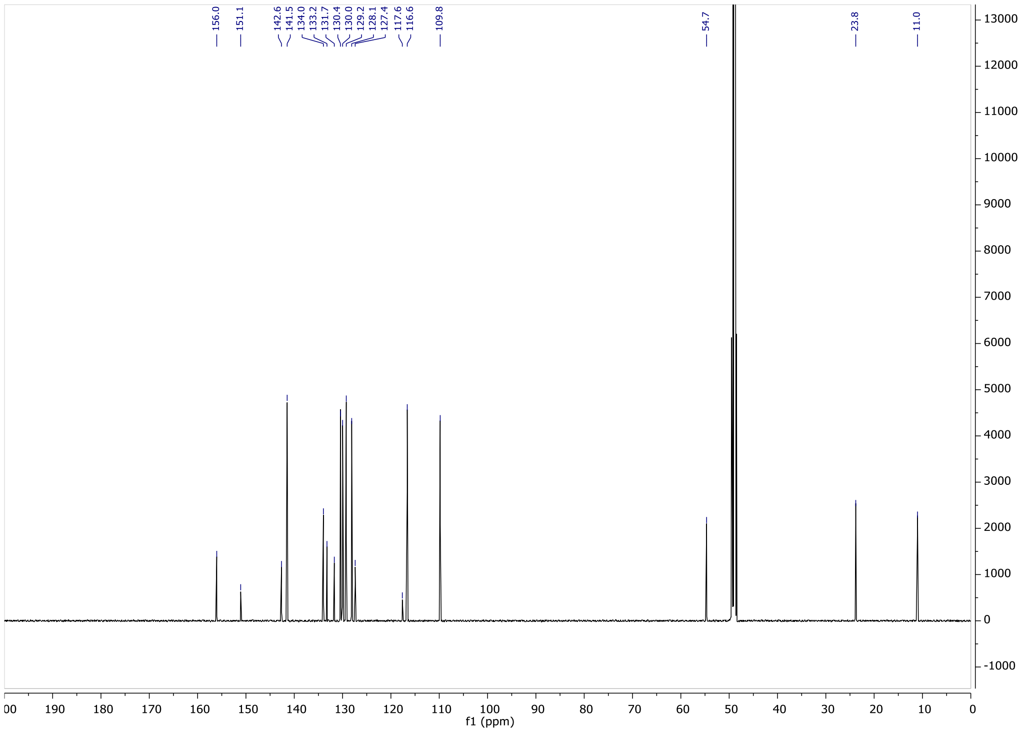

^{19}F NMR (282 MHz, CD_3OD) of **5**

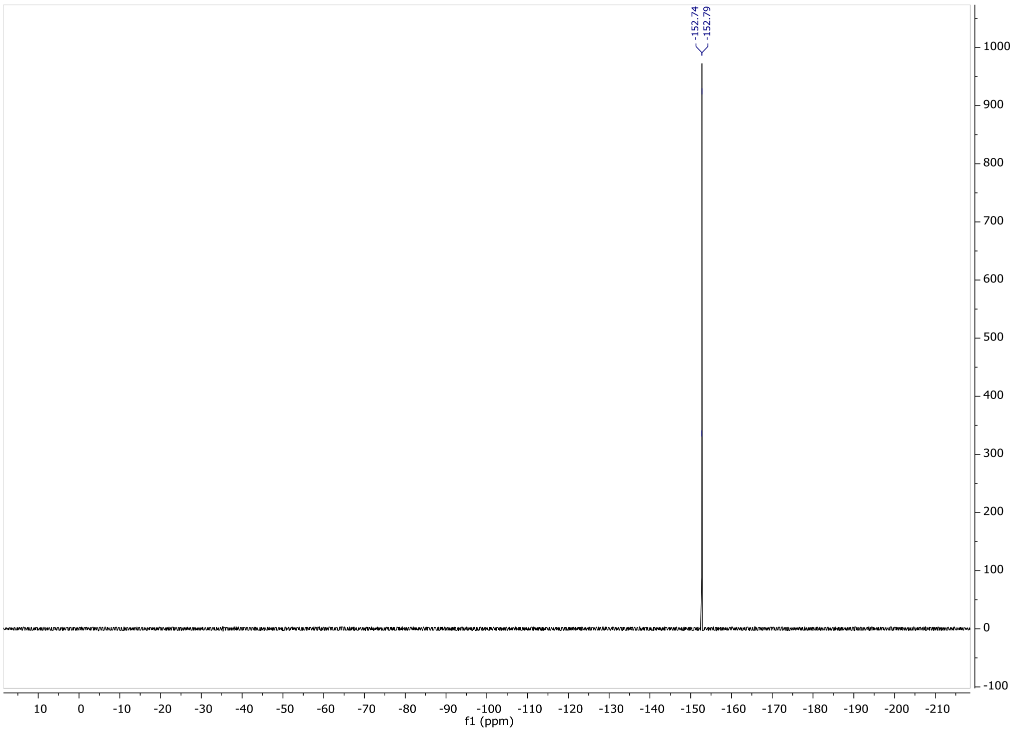

UV/VIS spectra recorded in CH_3CN (2.10^{-5} M) of **5**

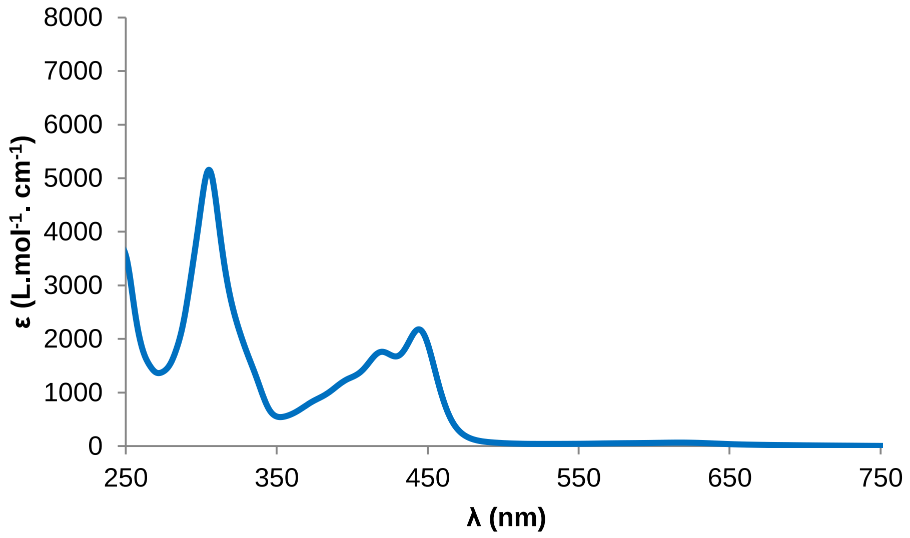

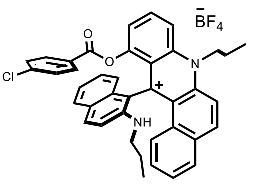

11-((4-chlorobenzoyl)oxy)-7-propyl-12-(2-(propylamino)naphthalen-1-yl)-7,12-dihydrobenzo[a]acridin-12-ylium tetrafluoroborate **6**.

^{1}H NMR (500 MHz, CD_2Cl_2) of **6**

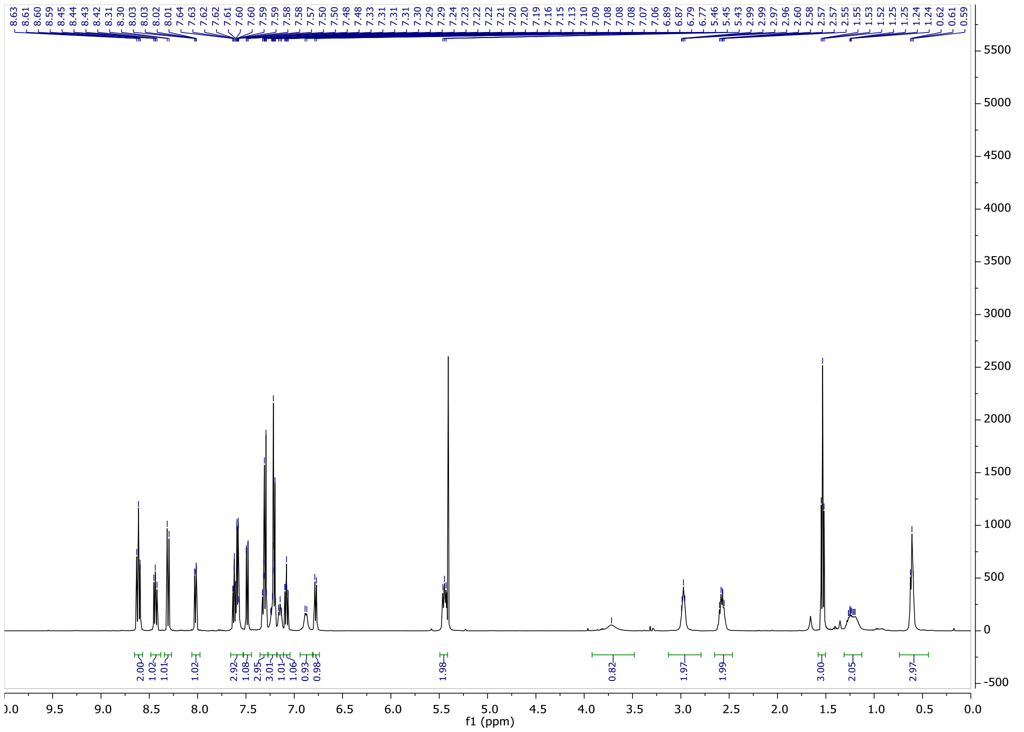

^{13}C NMR (125 MHz, CD_2Cl_2) of **6**

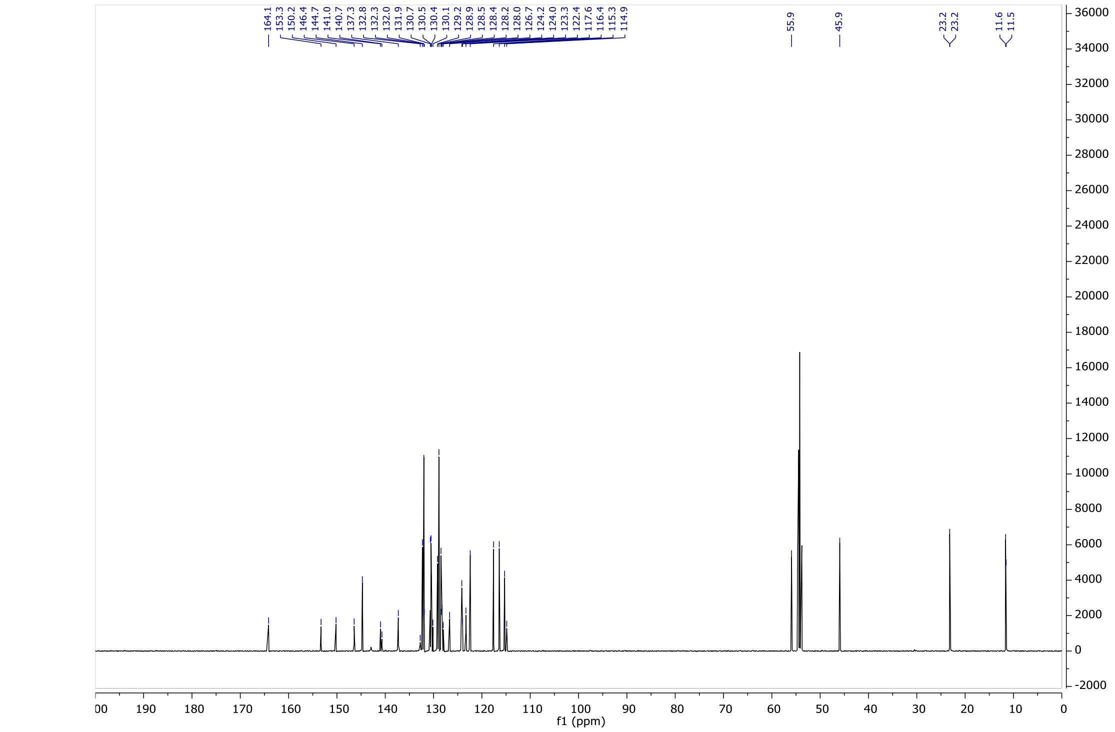

^{19}F NMR (282 MHz, CD_2Cl_2) of **6**

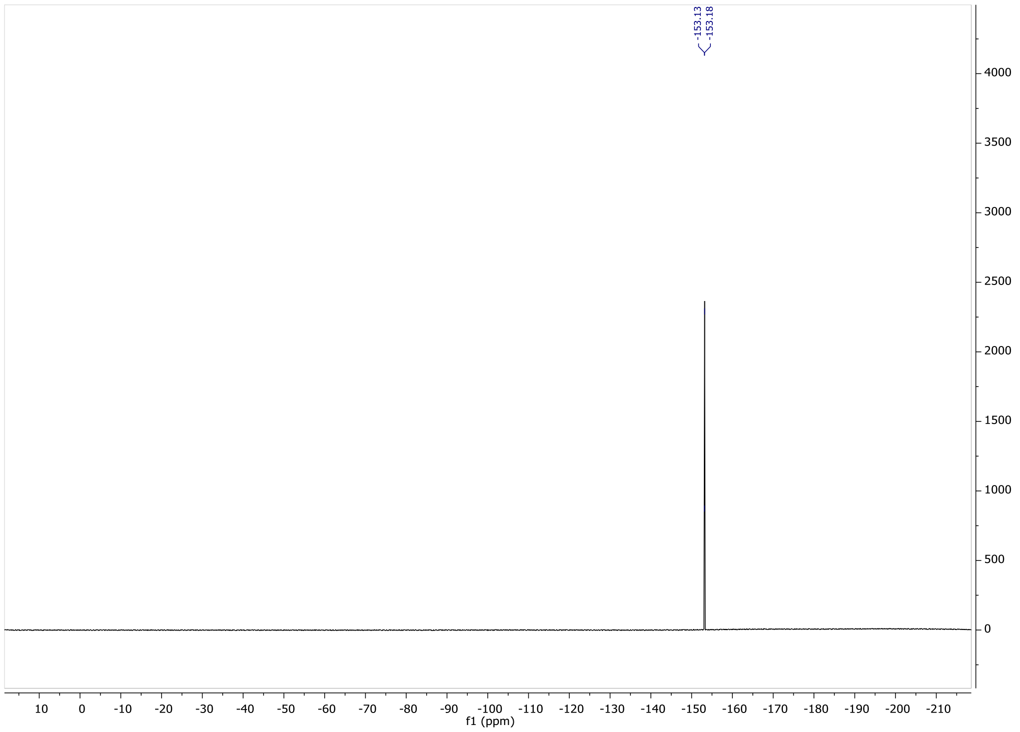

11. **Energies and Cartesian coordinates for all the intermediates and transition states**

From Dioxa (*M*)-**1** to Azaoxa (*M*)-**2** *via* intermediate (*aR*)-**A**

Attack on the *Re* face.

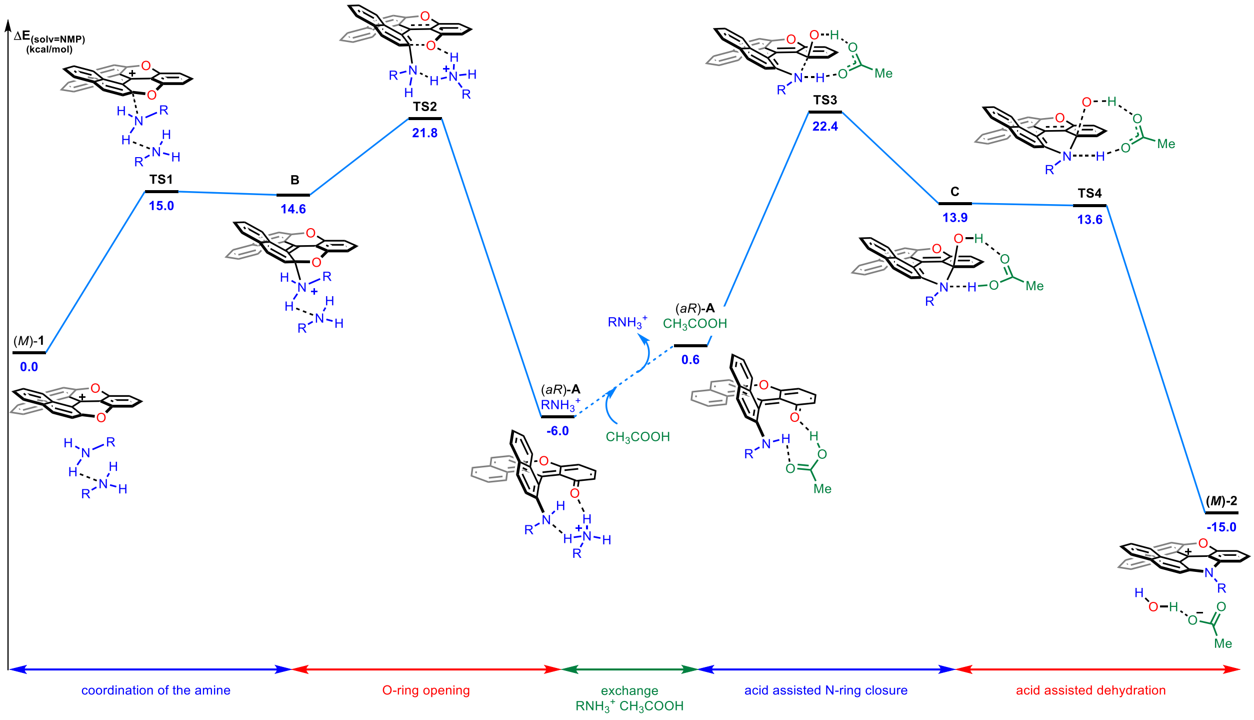

(*M*)-**1**

Geometry optimization in gas phase, B3LYP/6-311G**

```
 SCF Done:  E(RB3LYP) =  -1380.23090013
 Zero-point correction=                           0.469567 (Hartree/Particle)
 Thermal correction to Energy=                    0.498904
 Thermal correction to Enthalpy=                  0.499848
 Thermal correction to Gibbs Free Energy=         0.405431
 Sum of electronic and zero-point Energies=          -1379.761333
 Sum of electronic and thermal Energies=             -1379.731996
 Sum of electronic and thermal Enthalpies=           -1379.731052
 Sum of electronic and thermal Free Energies=        -1379.825469
```

Energy in DMA:

```
 SCF Done:  E(RM06L) =  -1380.26606140
```

Energy in NMP:

```
 SCF Done:  E(RM06L) =  -1380.26580467
```

Cartesian coordinates:

```
 C    -0.607724    -2.404239     1.724115
 C    -1.448646    -1.317557     1.402517
 C    -2.560002    -1.062081     2.263362
 C    -2.733876    -1.829198     3.438143
 C    -1.872200    -2.858730     3.741198
 C    -0.818244    -3.158076     2.861664
 C    -3.525485    -0.081489     1.897008
 C    -3.467862     0.556774     0.691219
 C    -2.389768     0.290560    -0.174404
 C    -1.296217    -0.516549     0.199050
 C    -0.155526    -0.484037    -0.678822
 C    -0.438560    -0.140048    -2.030726
```

C	-1.620171	0.543226	-2.371245
O	-2.482428	0.890782	-1.383653
C	0.452241	-0.461515	-3.070893
O	1.589266	-1.144142	-2.773303
C	2.012106	-1.212253	-1.487713
C	1.222454	-0.794034	-0.396533
C	3.298179	-1.765139	-1.328032
C	3.858606	-1.794897	-0.083134
C	3.202367	-1.213378	1.039350
C	1.888961	-0.672874	0.888681
C	1.337783	0.032605	1.979004
C	2.021827	0.143234	3.173739
C	3.284349	-0.450776	3.340598
C	3.867717	-1.110649	2.282627
C	-1.903475	0.902624	-3.678303
C	-1.000169	0.542136	-4.678512
C	0.177853	-0.146993	-4.391514
N	0.216832	2.641353	-0.280502
C	1.155882	2.972544	-1.359938
N	-1.840479	5.047219	-0.264543
C	-2.056340	5.636482	1.068078
H	0.380815	0.519867	1.870038
H	1.578865	0.700412	3.990845
H	3.803501	-0.367223	4.287527
H	4.859088	-1.538561	2.379780
H	4.846735	-2.219780	0.052968
H	3.811316	-2.135820	-2.205540
H	0.877007	-0.429575	-5.167037
H	-1.220460	0.802872	-5.706316
H	-2.816321	1.438341	-3.901656
H	0.200014	-2.676334	1.060865
H	-0.164122	-3.995758	3.072679
H	-2.018278	-3.451130	4.636071
H	-3.578010	-1.610312	4.082319
H	-4.351622	0.112897	2.571769
H	-4.233128	1.247336	0.362616
H	0.695692	2.735273	0.610940
H	0.647247	2.889897	-2.324436
H	1.987763	2.261861	-1.366015
H	1.587128	3.981823	-1.300671
H	-0.537131	3.337043	-0.266040
H	-1.368688	5.719278	-0.863009
H	-2.734973	4.860228	-0.708234
H	-2.640556	6.565328	1.065861
H	-2.572205	4.912942	1.703293
H	-1.089061	5.846734	1.529359

TS1

Geometry optimization in gas phase, B3LYP/6-311G**

SCF Done: E(RB3LYP) = -1380.20675625
Zero-point correction= 0.471227 (Hartree/Particle)
Thermal correction to Energy= 0.498305
Thermal correction to Enthalpy= 0.499249
Thermal correction to Gibbs Free Energy= 0.413481
Sum of electronic and zero-point Energies= -1379.735529
Sum of electronic and thermal Energies= -1379.708451
Sum of electronic and thermal Enthalpies= -1379.707507
Sum of electronic and thermal Free Energies= -1379.793275

Energy in DMA:

SCF Done: E(RM06L) = -1380.24221725

Energy in NMP:

SCF Done: E(RM06L) = -1380.24190320

Cartesian coordinates:

C	8.459477	13.996444	0.958796
C	7.772822	15.181653	0.621456
C	6.629211	15.503682	1.408839
C	6.292260	14.727748	2.529276
C	7.033074	13.611452	2.875289

C	8.104060	13.235399	2.061286
C	5.719341	16.533889	0.965035
C	5.936488	17.253582	-0.145472
C	7.200459	17.131660	-0.888725
C	8.095941	15.980412	-0.563482
C	9.131721	15.745881	-1.480999
C	8.858655	16.112588	-2.858309
C	7.754235	16.902774	-3.206438
O	6.969720	17.444569	-2.225117
C	9.580469	15.535822	-3.915430
O	10.596210	14.669589	-3.651492
C	11.087161	14.576188	-2.386463
C	10.449273	15.138378	-1.272201
C	12.298634	13.858894	-2.306734
C	12.955139	13.786346	-1.114340
C	12.466903	14.476281	0.029245
C	11.221301	15.178701	-0.039797
C	10.844746	15.928073	1.100026
C	11.626850	15.963680	2.235853
C	12.825276	15.233693	2.309613
C	13.233882	14.504900	1.217868
C	7.406745	17.152722	-4.524071
C	8.174126	16.584018	-5.540798
C	9.258665	15.765618	-5.247158
N	8.035027	18.599147	-0.348883
C	9.329318	18.957713	-0.972158
N	6.262183	20.866720	-0.788813
C	6.910326	22.187986	-0.626424
H	9.921460	16.485048	1.091811
H	11.307093	16.557196	3.084595
H	13.423212	15.255795	3.212443
H	14.166931	13.953145	1.243481
H	13.886198	13.236572	-1.037225
H	12.679678	13.399658	-3.209491
H	9.848447	15.294927	-6.022310
H	7.913548	16.774415	-6.574474
H	6.542803	17.767511	-4.739709
H	9.272110	13.647630	0.341500
H	8.663141	12.334042	2.283162
H	6.764582	13.019385	3.741478
H	5.416277	15.001701	3.107105
H	4.794435	16.663377	1.517308
H	5.213501	17.956992	-0.538017
H	8.142595	18.468757	0.656337
H	9.167610	19.160622	-2.029055
H	10.044357	18.145619	-0.857567
H	9.731687	19.853161	-0.494350
H	7.347517	19.387804	-0.493873
H	5.880945	20.788872	-1.728812
H	5.460630	20.807085	-0.165258
H	6.234333	23.029833	-0.805544
H	7.303631	22.274964	0.387916
H	7.747500	22.267842	-1.321885

B

Geometry optimization in gas phase, B3LYP/6-311G**

SCF Done: E(RB3LYP) = -1380.20683841

Zero-point correction=   0.471949 (Hartree/Particle)
Thermal correction to Energy=   0.499450
Thermal correction to Enthalpy=   0.500394
Thermal correction to Gibbs Free Energy=   0.413854
Sum of electronic and zero-point Energies=   -1379.734889
Sum of electronic and thermal Energies=   -1379.707389
Sum of electronic and thermal Enthalpies=   -1379.706445
Sum of electronic and thermal Free Energies=   -1379.792985

Energy in DMA:

SCF Done: E(RM06L) = -1380.24289766

Energy in NMP:

SCF Done: E(RM06L) = -1380.24257242

Cartesian coordinates:

C	-0.540177	-2.099060	1.762969
C	-1.229119	-0.917000	1.418092
C	-2.387872	-0.607544	2.188580
C	-2.737678	-1.390453	3.299497
C	-1.993640	-2.501960	3.655205
C	-0.906905	-2.866369	2.857597
C	-3.302091	0.415131	1.732329
C	-3.068783	1.152178	0.638640
C	-1.777193	1.065337	-0.079945
C	-0.892377	-0.112560	0.240551
C	0.136139	-0.356161	-0.677904
C	-0.137720	0.008251	-2.058400
C	-1.237893	0.802988	-2.408592
O	-2.010833	1.368157	-1.430157
C	0.569808	-0.588821	-3.113424
O	1.579704	-1.462241	-2.848690
C	2.079248	-1.547361	-1.586130
C	1.450326	-0.975965	-0.472435
C	3.290699	-2.265357	-1.510264
C	3.956484	-2.329960	-0.322702
C	3.476117	-1.633026	0.819777
C	2.229635	-0.931259	0.755201
C	1.858510	-0.180767	1.896521
C	2.647350	-0.140351	3.027357
C	3.848074	-0.867270	3.095523
C	4.250470	-1.599012	2.003604
C	-1.593559	1.037728	-3.726919
C	-0.838654	0.450419	-4.742634
C	0.240795	-0.372796	-4.446150
N	-0.988973	2.454570	0.447395
C	0.310640	2.821732	-0.171812
N	-2.720683	4.716017	0.024135
C	-2.074686	6.039156	0.186748
H	0.932204	0.371194	1.893988
H	2.330865	0.453574	3.877088
H	4.451583	-0.841605	3.994546
H	5.184009	-2.150134	2.025363
H	4.887901	-2.879534	-0.248669
H	3.664362	-2.731241	-2.412720
H	0.821503	-0.858525	-5.218871
H	-1.105470	0.630195	-5.776626
H	-2.454497	1.656334	-3.943877
H	0.284617	-2.440493	1.158229
H	-0.343650	-3.763626	3.085518
H	-2.271755	-3.098753	4.515076
H	-3.626129	-1.126053	3.862626
H	-4.242701	0.523346	2.262318
H	-3.794139	1.847196	0.235214
H	-0.879570	2.327075	1.453764
H	0.153374	3.008878	-1.231674
H	1.035571	2.022773	-0.037458
H	0.684668	3.729748	0.303125
H	-1.671387	3.258200	0.306104
H	-3.100817	4.636914	-0.916410
H	-3.523054	4.655101	0.646746
H	-2.753967	6.877865	0.007292
H	-1.682293	6.127270	1.201254
H	-1.238346	6.121130	-0.509297

TS2

Geometry optimization in gas phase, B3LYP/6-311G**

SCF Done: E(RB3LYP) = -1380.18839166

Zero-point correction= 0.470578 (Hartree/Particle)
Thermal correction to Energy= 0.497240
Thermal correction to Enthalpy= 0.498184
Thermal correction to Gibbs Free Energy= 0.414473

Sum of electronic and zero-point Energies= -1379.717814
Sum of electronic and thermal Energies= -1379.691152
Sum of electronic and thermal Enthalpies= -1379.690207
Sum of electronic and thermal Free Energies= -1379.773918

Energy in DMA:

SCF Done: E(RM06L) = -1380.23145348

Energy in NMP:

SCF Done: E(RM06L) = -1380.23105803

Cartesian coordinates:

C	7.771589	18.005893	-0.592643
C	8.387969	16.708017	-0.357373
C	7.839717	15.822719	0.657999
C	6.765900	16.277833	1.478502
C	6.221372	17.583375	1.254814
C	6.681441	18.399690	0.281866
C	6.202429	15.432230	2.453405
C	6.661292	14.142385	2.627110
C	7.687109	13.669925	1.796682
C	8.255746	14.482997	0.833728
C	9.327394	16.271052	-1.348979
C	10.575568	15.561050	-1.113942
C	11.198464	14.962893	-2.222480
O	10.683469	15.012969	-3.471960
C	9.565701	15.736385	-3.747726
C	8.924067	16.469554	-2.720510
C	12.403708	14.236271	-2.151224
C	13.048949	14.133797	-0.956118
C	12.548080	14.785086	0.204213
C	11.318686	15.518740	0.145096
C	13.290094	14.726065	1.406878
C	12.869900	15.390521	2.534607
C	11.686105	16.142561	2.478398
C	10.930425	16.197018	1.323956
C	7.696889	17.136732	-3.078509
C	7.167653	16.966422	-4.365338
C	7.864136	16.253814	-5.332964
C	9.076915	15.637981	-5.039327
O	7.067113	17.931955	-2.230946
N	8.653100	19.150911	-0.921940
C	9.346591	19.736985	0.251603
H	10.019106	16.769287	1.336777
H	11.349985	16.682947	3.355817
H	13.445368	15.341420	3.450935
H	14.210059	14.152265	1.417007
H	13.971268	13.569004	-0.881461
H	12.777861	13.778962	-3.057675
H	9.621955	15.054904	-5.768971
H	7.454191	16.172609	-6.332782
H	6.213680	17.426700	-4.591681
H	9.026720	14.068599	0.200497
H	8.037366	12.649504	1.901326
H	6.223728	13.497863	3.379437
H	5.385538	15.809836	3.059101
H	5.405718	17.913307	1.890069
H	6.248345	19.383795	0.141775
H	9.368946	18.809858	-1.561941
H	9.972079	19.008675	0.773923
H	8.613152	20.131658	0.953602
H	9.977815	20.556429	-0.096757
H	7.744253	20.195791	-1.845994
N	7.024964	20.618914	-2.564367
H	7.522305	20.834690	-3.428446
H	6.445173	19.788099	-2.745377
C	6.253874	21.793705	-2.068874
H	5.528546	22.106559	-2.818167
H	5.733784	21.511573	-1.155576
H	6.942303	22.610410	-1.858728

(***aR***)-**A**-RNH_3^+

Geometry optimization in gas phase, B3LYP/6-311G**

SCF Done: E(RB3LYP) = -1380.23563024
Zero-point correction= 0.471726 (Hartree/Particle)
Thermal correction to Energy= 0.499445
Thermal correction to Enthalpy= 0.500389
Thermal correction to Gibbs Free Energy= 0.412567
Sum of electronic and zero-point Energies= -1379.763904
Sum of electronic and thermal Energies= -1379.736186
Sum of electronic and thermal Enthalpies= -1379.735241
Sum of electronic and thermal Free Energies= -1379.823063

Energy in DMA:

SCF Done: E(RM06L) = -1380.27578828

Energy in NMP:

SCF Done: E(RM06L) = -1380.27541090

Cartesian coordinates:

C	-0.462856	1.723027	0.801172
C	-0.653604	0.402153	0.426710
C	-1.621948	-0.409691	1.102072
C	-2.359686	0.149595	2.192435
C	-2.108094	1.492174	2.569402
C	-1.195778	2.257975	1.892131
C	-3.309274	-0.649587	2.877984
C	-3.524108	-1.955446	2.513551
C	-2.788830	-2.515693	1.446595
C	-1.861749	-1.767015	0.760777
C	0.183718	-0.224123	-0.650126
C	1.503496	-0.735036	-0.345512
C	2.240923	-1.245680	-1.437567
O	1.703013	-1.410128	-2.656512
C	0.426173	-1.041871	-2.945358
C	-0.331977	-0.333296	-1.949270
C	3.597205	-1.617358	-1.373061
C	4.254110	-1.504916	-0.186045
C	3.581570	-1.083412	0.994483
C	2.189982	-0.734426	0.955168
C	4.297791	-1.062124	2.212982
C	3.675524	-0.744692	3.397330
C	2.302861	-0.461025	3.382243
C	1.582348	-0.454870	2.202078
C	-1.655856	0.198546	-2.374547
C	-2.130818	-0.215046	-3.652702
C	-1.358212	-0.957049	-4.514901
C	-0.049613	-1.363575	-4.187399
O	-2.324426	0.986931	-1.647159
N	0.378601	2.598557	0.050181
C	1.295633	3.490166	0.791480
H	0.528910	-0.258443	2.263250
H	1.785212	-0.254142	4.311645
H	4.228614	-0.738756	4.328536
H	5.350139	-1.322325	2.196035
H	5.303841	-1.767671	-0.119991
H	4.074422	-1.973926	-2.276149
H	0.574221	-1.910965	-4.880127
H	-1.755480	-1.223869	-5.488015
H	-3.124481	0.113148	-3.930349
H	-1.313167	-2.222072	-0.053850
H	-2.958179	-3.548372	1.164843
H	-4.252053	-2.559012	3.042135
H	-3.863731	-0.208781	3.699334
H	-2.651653	1.912651	3.408480
H	-1.025153	3.282665	2.199042
H	0.905384	2.072387	-0.638074
H	1.906200	2.949417	1.521258
H	0.738877	4.268929	1.312630
H	1.952282	3.980286	0.071879
H	-1.093631	3.483145	-0.914505
N	-2.003307	3.539446	-1.424218

H	-1.845689	3.998077	-2.320523
H	-2.243289	2.503223	-1.599194
C	-3.070664	4.224929	-0.640486
H	-3.993674	4.219420	-1.217480
H	-3.217270	3.673051	0.285309
H	-2.775159	5.250407	-0.423217

(***aR***)-**A**-CH3COO$^-$

Geometry optimization in gas phase, B3LYP/6-311G**

SCF Done: E(RB3LYP) = -1513.08559322

Zero-point correction=	0.453757	(Hartree/Particle)
Thermal correction to Energy=	0.483465	
Thermal correction to Enthalpy=	0.484409	
Thermal correction to Gibbs Free Energy=	0.390108	
Sum of electronic and zero-point Energies=	-1512.631836	
Sum of electronic and thermal Energies=	-1512.602128	
Sum of electronic and thermal Enthalpies=	-1512.601184	
Sum of electronic and thermal Free Energies=	-1512.695485	

Energy in DMA:

SCF Done: E(RM06L) = -1513.08526288

Energy in NMP:

SCF Done: E(RM06L) = -1513.08539490

Cartesian coordinates:

O	-0.228945	2.407531	2.666759
C	0.997812	0.552353	1.731380
C	2.131544	-0.351054	1.948175
C	0.811531	1.555403	2.694557
C	-1.022864	1.420969	0.606819
C	0.057496	0.539130	0.615104
C	-1.191316	2.342423	1.700246
C	-0.544459	-1.471997	-0.723966
C	4.137309	-0.933035	3.279437
H	4.789698	-0.696648	4.113202
C	0.272443	-0.365001	-0.555933
C	-3.169703	2.370617	-0.260367
C	3.021893	-0.094786	3.044479
C	3.502290	-2.310607	1.432476
H	3.669545	-3.180423	0.807571
C	0.561317	-1.996482	-2.822360
H	0.666836	-2.624902	-3.700780
C	4.385796	-2.026012	2.485710
H	5.240883	-2.664367	2.674291
C	-2.246420	3.198668	1.833224
C	-3.247639	3.185768	0.832325
H	-4.095142	3.854697	0.940473
C	-2.062042	1.475074	-0.475195
C	1.690249	1.791390	3.773143
H	1.464190	2.611025	4.442584
C	2.779975	0.992308	3.927854
H	3.472144	1.164002	4.744767
C	1.408661	-0.869845	-2.682044
C	3.024824	1.407456	-2.399483
H	3.646277	2.290476	-2.300311
C	2.414717	-1.502705	1.171769
H	1.769032	-1.774302	0.357024
C	3.175542	0.569982	-3.527098
H	3.912416	0.811653	-4.284595
C	2.380817	-0.541321	-3.661436
H	2.478959	-1.186058	-4.528812
C	2.095953	1.115458	-1.429932
H	1.993131	1.776484	-0.578415
C	1.263130	-0.032335	-1.529907
C	-0.382950	-2.287200	-1.872603
H	-1.047506	-3.134669	-1.994531
N	-1.581463	-1.738774	0.205503
C	-1.251857	-2.560894	1.369347
H	-0.861941	-3.559899	1.115831
H	-0.507606	-2.062314	1.995174

H	-2.154072	-2.690082	1.971263
H	-3.935007	2.377935	-1.026322
H	-2.292701	3.865593	2.683055
O	-1.984059	0.778562	-1.504766
H	-2.392480	-2.094180	-0.294416
O	-3.808858	-2.135510	-1.809158
C	-4.398324	-1.404032	-2.581381
C	-5.610661	-1.840775	-3.372300
O	-4.068077	-0.145157	-2.842878
H	-3.259503	0.153359	-2.332094
H	-5.390887	-1.780794	-4.440986
H	-5.879315	-2.860480	-3.104849
H	-6.446589	-1.165982	-3.175234

TS3

Geometry optimization in gas phase, B3LYP/6-311G**

SCF Done: E(RB3LYP) = -1513.051206

Zero-point correction= 0.451080 (Hartree/Particle)

Thermal correction to Energy= 0.478891

Thermal correction to Enthalpy= 0.479835

Thermal correction to Gibbs Free Energy= 0.393499

Sum of electronic and zero-point Energies= -1512.600125

Sum of electronic and thermal Energies= -1512.572315

Sum of electronic and thermal Enthalpies= -1512.571371

Sum of electronic and thermal Free Energies= -1512.657707

Energy in DMA:

SCF Done: E(RM06L) = -1513.05057768

Energy in NMP:

SCF Done: E(RM06L) = -1513.05043738

Cartesian coordinates:

O	1.653903	4.229821	7.653093
C	3.062912	2.449392	6.821216
C	4.258284	1.678971	7.076837
C	2.846510	3.567968	7.626122
C	0.780827	2.689215	6.032674
C	2.018937	2.154605	5.824088
C	0.548496	3.612520	7.108706
C	1.136698	0.451990	4.282724
C	6.446640	1.479903	8.197145
H	7.197159	1.915906	8.848150
C	2.189724	1.285577	4.628394
C	-1.725030	2.449508	5.879720
C	5.260687	2.215513	7.950255
C	5.625622	-0.327943	6.846118
H	5.751732	-1.326932	6.444301
C	2.345759	-0.501341	2.439221
H	2.414736	-1.197278	1.610512
C	6.635584	0.233683	7.652703
H	7.542264	-0.324039	7.856414
C	-0.690722	3.908367	7.562988
C	-1.833307	3.263451	6.945938
H	-2.813899	3.476273	7.358936
C	-0.407746	2.309711	5.161512
C	3.838443	4.105664	8.471145
H	3.611073	5.017235	9.009155
C	5.034871	3.458153	8.599461
H	5.808485	3.862569	9.242774
C	3.410961	0.401903	2.669146
C	5.423076	2.323631	3.014039
H	6.188614	3.083094	3.124244
C	4.475625	0.373210	6.564416
H	3.717342	-0.094554	5.955004
C	5.529652	1.368980	1.978078
H	6.382334	1.391028	1.309159
C	4.540867	0.434032	1.809544
H	4.594507	-0.285656	0.999583
C	4.357068	2.303899	3.879399
H	4.290879	3.052628	4.655963

C	3.327554	1.332548	3.756652
C	1.211341	-0.453712	3.209862
H	0.352432	-1.073776	2.983074
N	-0.130311	0.590720	4.985773
C	-0.265375	-0.243541	6.203408
H	-0.198296	-1.299870	5.935836
H	0.523912	0.004628	6.912262
H	-1.234535	-0.040994	6.657615
H	-2.598317	2.039741	5.387345
H	-0.820867	4.612989	8.373392
O	-0.380350	2.788187	3.946796
H	-0.903018	0.356740	4.308856
O	-2.109681	0.100696	3.183897
C	-2.557019	0.991027	2.436896
C	-3.580800	0.643429	1.376107
O	-2.220270	2.232499	2.473396
H	-1.366555	2.491799	3.210579
H	-3.117489	0.739234	0.390346
H	-3.940678	-0.374949	1.511594
H	-4.410455	1.351665	1.413227

C

Geometry optimization in gas phase, B3LYP/6-311G**

SCF Done: E(RB3LYP) = -1513.07054980
Zero-point correction= 0.455203 (Hartree/Particle)
Thermal correction to Energy= 0.484005
Thermal correction to Enthalpy= 0.484949
Thermal correction to Gibbs Free Energy= 0.395007
Sum of electronic and zero-point Energies= -1512.615347
Sum of electronic and thermal Energies= -1512.586545
Sum of electronic and thermal Enthalpies= -1512.585601
Sum of electronic and thermal Free Energies= -1512.675543

Energy in DMA:

SCF Done: E(RM06L) = -1513.06417139

Energy in NMP:

SCF Done: E(RM06L) = -1513.06405559

Cartesian coordinates:

O	-0.175253	2.618120	2.300137
C	1.319415	0.875090	1.544248
C	2.572763	0.209194	1.822451
C	1.062250	2.045636	2.255089
C	-1.026442	0.848120	0.923062
C	0.247537	0.404949	0.646060
C	-1.262036	1.888287	1.878439
C	-0.706924	-1.402429	-0.675429
C	4.818347	0.267415	2.840713
H	5.569396	0.818862	3.396703
C	0.392729	-0.545274	-0.449572
C	-3.513494	0.533636	0.847735
C	3.577101	0.898656	2.577494
C	4.045562	-1.733098	1.760380
H	4.211223	-2.766292	1.476916
C	0.607333	-2.719885	-2.228748
H	0.705653	-3.578567	-2.884726
C	5.058200	-1.022132	2.434659
H	6.006921	-1.499558	2.651017
C	-2.503259	2.229629	2.308644
C	-3.627121	1.491038	1.791669
H	-4.612788	1.734637	2.174279
C	-2.201197	0.262237	0.174421
C	2.052576	2.733692	2.987018
H	1.784508	3.675840	3.448168
C	3.298357	2.187169	3.106689
H	4.073083	2.707076	3.659419
C	1.655412	-1.760929	-2.196436
C	3.618712	0.228932	-2.236830
H	4.365609	1.014081	-2.273381
C	2.843216	-1.135413	1.459434

H	2.082405	-1.712614	0.956943
C	3.752647	-0.901297	-3.068211
H	4.605934	-0.990561	-3.730592
C	2.780808	-1.873235	-3.048139
H	2.851273	-2.736034	-3.702648
C	2.545173	0.353725	-1.384132
H	2.458315	1.239540	-0.769900
C	1.539463	-0.643347	-1.314614
C	-0.544430	-2.540194	-1.516848
H	-1.362838	-3.236132	-1.638130
N	-1.921136	-1.131207	-0.078178
C	-2.949951	-2.162996	0.062623
H	-3.617812	-2.214348	-0.801205
H	-2.464536	-3.125153	0.222111
H	-3.548116	-1.948710	0.945905
H	-4.385259	0.060736	0.416036
H	-2.636064	3.037517	3.014906
O	-2.272979	0.897215	-1.190332
H	-3.388369	0.454904	-2.465620
O	-3.975746	0.399780	-3.261902
C	-5.066138	-0.316272	-2.981789
C	-6.008275	-0.389438	-4.159378
O	-5.272902	-0.847869	-1.910774
H	-2.361905	1.847435	-1.042445
H	-6.310974	0.617994	-4.454025
H	-5.496006	-0.836761	-5.014349
H	-6.882461	-0.980555	-3.895674

TS4

Geometry optimization in gas phase, B3LYP/6-311G**

SCF Done: E(RB3LYP) = -1513.060209
Zero-point correction= 0.451555 (Hartree/Particle)
Thermal correction to Energy= 0.479973
Thermal correction to Enthalpy= 0.480918
Thermal correction to Gibbs Free Energy= 0.392280
Sum of electronic and zero-point Energies= -1512.608654
Sum of electronic and thermal Energies= -1512.580236
Sum of electronic and thermal Enthalpies= -1512.579292
Sum of electronic and thermal Free Energies= -1512.667929

Energy in DMA:

SCF Done: E(RM06L) = -1513.06469042

Energy in NMP:

SCF Done: E(RM06L) = -1513.06453450

Cartesian coordinates:

O	-0.165787	2.409515	2.218936
C	1.352349	0.653146	1.545731
C	2.620929	0.022939	1.842321
C	1.076155	1.852706	2.201929
C	-1.007306	0.524919	0.991287
C	0.286910	0.111897	0.691780
C	-1.242128	1.651878	1.831762
C	-0.681402	-1.662178	-0.662022
C	4.863803	0.161551	2.854007
H	5.604472	0.746086	3.389443
C	0.425021	-0.813938	-0.404228
C	-3.460422	0.200615	0.905420
C	3.611747	0.758999	2.567909
C	4.131274	-1.889278	1.841241
H	4.318475	-2.927090	1.590254
C	0.622186	-2.930690	-2.261869
H	0.709666	-3.760073	-2.955540
C	5.128024	-1.137154	2.492748
H	6.085412	-1.588585	2.725406
C	-2.497570	2.042063	2.197179
C	-3.603724	1.274578	1.733230
H	-4.600878	1.574579	2.034979
C	-2.153519	-0.150676	0.410235
C	2.057810	2.588005	2.900072

H	1.777199	3.546711	3.317167
C	3.310324	2.064586	3.043191
H	4.076827	2.620039	3.572171
C	1.686958	-1.989828	-2.185078
C	3.651053	-0.006640	-2.190261
H	4.393740	0.782578	-2.216468
C	2.917174	-1.325120	1.520353
H	2.169981	-1.932587	1.032547
C	3.783695	-1.119087	-3.041500
H	4.634497	-1.195575	-3.708452
C	2.808052	-2.088807	-3.041324
H	2.870544	-2.933737	-3.718993
C	2.579525	0.099847	-1.329980
H	2.492525	0.978301	-0.706859
C	1.579238	-0.899502	-1.273724
C	-0.529729	-2.763941	-1.554057
H	-1.357250	-3.436570	-1.721618
N	-1.889379	-1.435783	-0.041038
C	-2.960480	-2.442052	-0.055667
H	-3.625225	-2.307037	-0.911252
H	-2.515906	-3.434296	-0.045369
H	-3.544283	-2.330923	0.855768
H	-4.320348	-0.296109	0.480851
H	-2.634478	2.916459	2.818918
O	-2.196781	0.774431	-1.379084
H	-3.133196	0.451294	-2.337793
O	-3.836944	0.320291	-3.160938
C	-4.899818	-0.371492	-2.853118
C	-5.910176	-0.436526	-3.985936
O	-5.101790	-0.933948	-1.780625
H	-2.405617	1.682163	-1.130098
H	-6.223568	0.574074	-4.258865
H	-5.443474	-0.877918	-4.870032
H	-6.774492	-1.026820	-3.686336

(M)-2

Geometry optimization in gas phase, B3LYP/6-311G**

SCF Done: E(RB3LYP) = -1513.086285

Zero-point correction= 0.453763 (Hartree/Particle)

Thermal correction to Energy= 0.483500

Thermal correction to Enthalpy= 0.484444

Thermal correction to Gibbs Free Energy= 0.392885

Sum of electronic and zero-point Energies= -1512.632521

Sum of electronic and thermal Energies= -1512.602785

Sum of electronic and thermal Enthalpies= -1512.601841

Sum of electronic and thermal Free Energies= -1512.693400

Energy in DMA:

SCF Done: E(RM06L) = -1207.95107102

Energy in NMP:

SCF Done: E(RM06L) = -1207.95079130

Cartesian coordinates:

O	-0.764232	2.358885	1.994022
C	0.909483	0.732979	1.369096
C	2.276032	0.308065	1.591466
C	0.528256	1.959774	1.914118
C	-1.458978	0.240690	1.107715
C	-0.133074	-0.031830	0.692089
C	-1.758560	1.461848	1.751786
C	-1.032229	-1.952990	-0.495078
C	4.553826	0.843769	2.359741
H	5.248605	1.573124	2.762465
C	0.010975	-0.988806	-0.342359
C	-3.836820	-0.298328	1.159527
C	3.212429	1.239406	2.140366
C	4.032691	-1.379374	1.616053
H	4.341537	-2.403621	1.441133
C	0.315600	-3.175967	-2.097962
H	0.453049	-4.035564	-2.745449

C	4.965064	-0.441057	2.097281
H	5.992091	-0.736972	2.276373
C	-3.048101	1.808429	2.092028
C	-4.077317	0.914109	1.783265
H	-5.099687	1.189565	2.013384
C	-2.523932	-0.648152	0.808047
C	1.458197	2.888155	2.430809
H	1.088066	3.849858	2.761742
C	2.775308	2.543260	2.507217
H	3.501632	3.248068	2.896682
C	1.281950	-2.126927	-2.144482
C	2.939322	0.088574	-2.464989
H	3.548837	0.970699	-2.625048
C	2.728528	-1.014340	1.364593
H	2.036357	-1.760674	1.004540
C	3.176617	-1.069440	-3.222826
H	3.983900	-1.092020	-3.945639
C	2.342891	-2.157205	-3.074042
H	2.468928	-3.038129	-3.694446
C	1.922929	0.127813	-1.529964
H	1.709622	1.047679	-1.007114
C	1.099886	-0.995335	-1.306893
C	-0.812738	-3.079700	-1.348941
H	-1.571568	-3.838047	-1.447017
N	-2.198090	-1.848152	0.207726
C	-3.251818	-2.866295	0.074499
H	-3.824230	-2.632696	-0.831010
H	-2.805709	-3.856273	0.046592
H	-3.887448	-2.818905	0.955020
H	-4.666943	-0.913237	0.853340
H	-3.240001	2.766706	2.555556
O	-0.724271	2.173070	-1.249317
H	-1.284343	1.441732	-1.623829
O	-2.172056	0.083508	-2.058443
C	-3.407778	0.005724	-2.353908
C	-3.969714	1.175128	-3.180231
O	-4.184550	-0.917137	-2.034178
H	-0.441528	2.671735	-2.020458
H	-3.777953	2.116349	-2.656140
H	-3.447363	1.229191	-4.140089
H	-5.039564	1.057685	-3.353336

Opening of (*M*)-**1**, attack on the *Si* face.

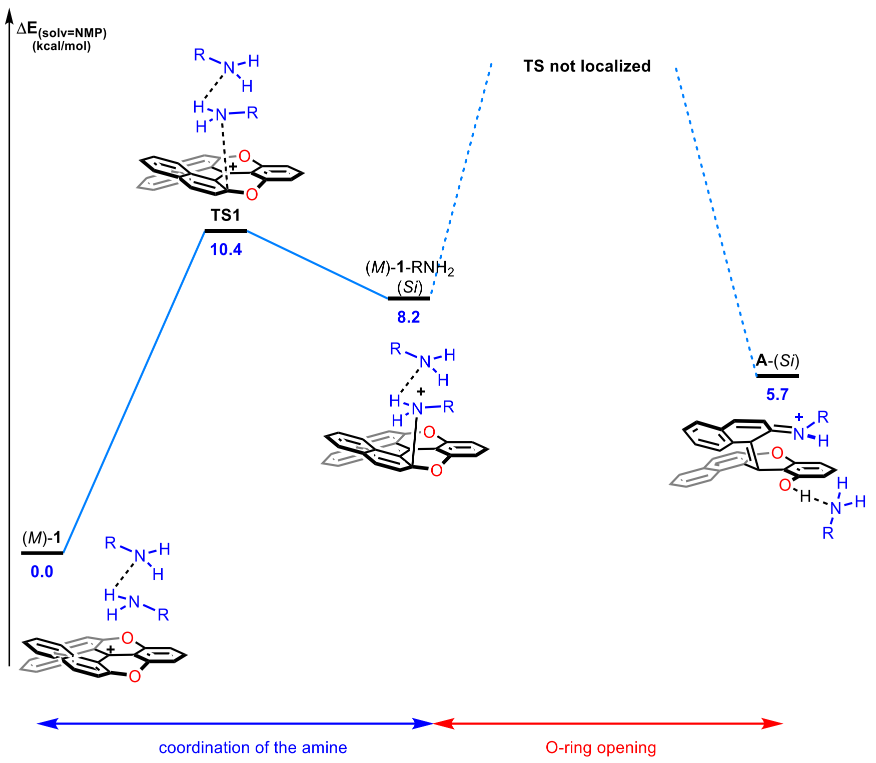

(*M*) -**1**

Geometry optimization in gas phase, B3LYP/6-311G**

SCF Done: E(RB3LYP) = -1380.23040588

Zero-point correction=	0.469601 (Hartree/Particle)
Thermal correction to Energy=	0.498884
Thermal correction to Enthalpy=	0.499828
Thermal correction to Gibbs Free Energy=	0.405550
Sum of electronic and zero-point Energies=	-1379.760805
Sum of electronic and thermal Energies=	-1379.731522
Sum of electronic and thermal Enthalpies=	-1379.730578
Sum of electronic and thermal Free Energies=	-1379.824856

Energy in DMA:

SCF Done: E(RM06L) = -1380.26515863

Energy in NMP:

SCF Done: E(RM06L) = -1380.26489503

Cartesian coordinates:

O	-1.461860	2.252731	-0.731981
C	-1.356525	1.677196	0.482215
C	-0.442830	0.636942	0.766358
C	0.513126	0.330451	-0.259678
C	0.133102	0.707749	-1.578489
C	-0.882387	1.657435	-1.801114
C	-0.606226	-0.049456	2.038570
C	-1.474015	0.516469	3.021579
C	-2.209372	1.699433	2.717245
C	-2.183144	2.250566	1.469728
C	-1.651359	-0.131777	4.264434
C	-1.037349	-1.336826	4.524693
C	-0.239463	-1.932088	3.534490
C	-0.023779	-1.302238	2.323739
C	0.759834	0.146705	-2.708642
O	1.712844	-0.805029	-2.524319
C	2.293291	-0.945079	-1.307073
C	1.803896	-0.309476	-0.147480
C	3.412823	-1.799324	-1.292959
C	4.132656	-1.931469	-0.140107

C	3.819746	-1.167078	1.019680
C	2.670387	-0.317756	1.019776
C	-1.235111	2.062865	-3.078139
C	-0.595855	1.468824	-4.163537
C	0.396269	0.500171	-3.995448
C	2.489110	0.538789	2.126677
C	3.360043	0.515747	3.197720
C	4.448643	-0.372160	3.223781
C	4.676958	-1.194078	2.143784
N	-3.025620	-0.507567	-0.954691
C	-2.595121	-1.782408	-1.542727
N	-5.621682	0.334434	-2.586799
C	-5.689322	1.521085	-3.455272
H	1.674706	1.248066	2.133079
H	3.202874	1.197225	4.025350
H	5.116167	-0.389006	4.076561
H	5.535960	-1.855272	2.126904
H	4.996068	-2.586645	-0.115504
H	3.680260	-2.312223	-2.207379
H	0.888960	0.036140	-4.839267
H	-0.873699	1.766685	-5.167123
H	-2.003098	2.813019	-3.207917
H	0.581743	-1.798611	1.579926
H	0.210598	-2.900641	3.717460
H	-1.186657	-1.834549	5.475022
H	-2.303607	0.323819	5.000938
H	-2.842000	2.130709	3.485035
H	-2.789746	3.104163	1.197950
H	-3.395482	-0.680220	-0.023726
H	-1.753644	-2.192941	-0.975740
H	-2.247675	-1.610366	-2.564620
H	-3.373528	-2.557650	-1.583247
H	-3.807716	-0.130334	-1.500350
H	-5.818476	-0.501292	-3.130170
H	-6.351306	0.382895	-1.881312
H	-6.651963	1.660199	-3.963634
H	-5.489702	2.414932	-2.859902
H	-4.911571	1.452408	-4.219223

TS1

Geometry optimization in gas phase, B3LYP/6-311G**

SCF Done: E(RB3LYP) = -1380.21628479

Zero-point correction= 0.471205 (Hartree/Particle)
Thermal correction to Energy= 0.498388
Thermal correction to Enthalpy= 0.499332
Thermal correction to Gibbs Free Energy= 0.413417
Sum of electronic and zero-point Energies= -1379.745080
Sum of electronic and thermal Energies= -1379.717897
Sum of electronic and thermal Enthalpies= -1379.716953
Sum of electronic and thermal Free Energies= -1379.802868

Energy in DMA:

SCF Done: E(RM06L) = -1380.24863947

Energy in NMP:

SCF Done: E(RM06L) = -1380.24834521

Cartesian coordinates:

O	6.675224	17.281207	-2.484076
C	6.613714	16.570490	-1.315400
C	7.790656	15.804646	-0.862322
C	8.867773	15.703104	-1.733519
C	8.619740	16.058477	-3.116583
C	7.514879	16.836466	-3.477961
C	7.634696	15.194251	0.464899
C	6.739719	15.786113	1.397529
C	5.997689	16.970639	1.007841
C	5.930371	17.359322	-0.276961
C	6.576444	15.218759	2.670335
C	7.248651	14.057555	3.019986
C	8.097600	13.448648	2.092743

C	8.292467	14.011442	0.838517
C	9.448414	15.591948	-4.143950
O	10.490668	14.769396	-3.845208
C	10.948457	14.727488	-2.556970
C	10.243129	15.270519	-1.476783
C	12.194004	14.083789	-2.411968
C	12.800421	14.072309	-1.188824
C	12.236605	14.770865	-0.085720
C	10.965692	15.412232	-0.228578
C	7.266658	17.200775	-4.790076
C	8.127261	16.728497	-5.784435
C	9.209291	15.910210	-5.476177
C	10.523940	16.231263	0.836622
C	11.262841	16.360962	1.993035
C	12.475873	15.667053	2.157222
C	12.954922	14.892014	1.127944
N	5.299522	15.270611	-1.697301
C	5.673059	14.157236	-2.591168
N	2.874347	16.624946	-2.691280
C	3.060994	17.595899	-3.790879
H	9.608286	16.794528	0.735154
H	10.906542	17.012602	2.782266
H	13.038534	15.766340	3.077533
H	13.908662	14.384201	1.218575
H	13.755406	13.575299	-1.061498
H	12.641733	13.629381	-3.286163
H	9.871569	15.527349	-6.241251
H	7.944298	16.999757	-6.816791
H	6.421059	17.833556	-5.024605
H	8.954614	13.521544	0.137143
H	8.611589	12.530382	2.350807
H	7.109858	13.621758	4.001882
H	5.904363	15.693693	3.376785
H	5.475711	17.531078	1.776003
H	5.377107	18.229129	-0.606822
H	5.067554	14.916952	-0.771421
H	6.627542	13.736349	-2.273609
H	5.764115	14.526191	-3.611307
H	4.916008	13.369487	-2.564085
H	4.465355	15.790825	-2.059782
H	2.207891	15.912308	-2.978586
H	2.443277	17.092290	-1.897256
H	2.136625	18.097627	-4.095366
H	3.781070	18.355174	-3.481565
H	3.471157	17.078522	-4.660010

(*M*)-**1**-RNH_3^+

Geometry optimization in gas phase, B3LYP/6-311G**

SCF Done: E(RB3LYP) = -1380.21803928
Zero-point correction= 0.472626 (Hartree/Particle)
Thermal correction to Energy= 0.499832
Thermal correction to Enthalpy= 0.500776
Thermal correction to Gibbs Free Energy= 0.415199
Sum of electronic and zero-point Energies= -1379.745414
Sum of electronic and thermal Energies= -1379.718207
Sum of electronic and thermal Enthalpies= -1379.717263
Sum of electronic and thermal Free Energies= -1379.802841

Energy in DMA:

SCF Done: E(RM06L) = -1380.25212494

Energy in NMP:

SCF Done: E(RM06L) = -1380.25179624

Cartesian coordinates:

O	-1.763261	1.535554	-1.381970
C	-1.857528	0.796532	-0.199052
C	-0.620734	0.077118	0.276628
C	0.469678	0.023971	-0.561983
C	0.249001	0.381720	-1.956397
C	-0.868564	1.123586	-2.347366

C	-0.779061	-0.512943	1.616089
C	-1.640765	0.121139	2.551011
C	-2.315895	1.351593	2.154044
C	-2.409604	1.704438	0.865143
C	-1.811573	-0.426561	3.828976
C	-1.172368	-1.606915	4.185048
C	-0.347533	-2.249476	3.260591
C	-0.149586	-1.707386	1.996129
C	1.115183	-0.064075	-2.959137
O	2.170277	-0.862300	-2.636830
C	2.598766	-0.893431	-1.335750
C	1.854511	-0.373107	-0.274114
C	3.860636	-1.498287	-1.163140
C	4.437594	-1.498105	0.074146
C	3.825774	-0.821716	1.164935
C	2.541510	-0.214821	0.990800
C	-1.089813	1.479982	-3.666423
C	-0.185573	1.038833	-4.637802
C	0.907997	0.251397	-4.299308
C	2.051226	0.589823	2.046741
C	2.754392	0.734060	3.222789
C	3.980178	0.069930	3.417673
C	4.507208	-0.686796	2.398807
N	-3.030601	-0.288094	-0.459205
C	-2.757368	-1.365435	-1.449880
N	-5.445807	1.043633	-1.190548
C	-5.346399	1.885670	-2.405806
H	1.126118	1.131778	1.919628
H	2.360990	1.374131	4.003913
H	4.514851	0.179323	4.353474
H	5.471472	-1.169409	2.513426
H	5.403553	-1.967053	0.223199
H	4.342852	-1.934796	-2.028130
H	1.604212	-0.111234	-5.043806
H	-0.346163	1.309505	-5.674074
H	-1.947738	2.086343	-3.925950
H	0.496736	-2.217915	1.293770
H	0.145953	-3.176498	3.527213
H	-1.315719	-2.026689	5.173276
H	-2.455893	0.079452	4.539871
H	-2.745112	1.980184	2.927086
H	-2.879169	2.618767	0.525163
H	-3.201243	-0.708195	0.456223
H	-1.820545	-1.860389	-1.201092
H	-2.695414	-0.931195	-2.444682
H	-3.573102	-2.088047	-1.417481
H	-3.918033	0.241971	-0.731031
H	-6.195296	0.365482	-1.308423
H	-5.727411	1.621314	-0.401569
H	-6.270117	2.425939	-2.632414
H	-4.540646	2.608835	-2.274917
H	-5.098257	1.254110	-3.260241

A^{Si}

Geometry optimization in gas phase, B3LYP/6-311G**

SCF Done: E(RB3LYP) = -1380.21947566

Zero-point correction= 0.470940 (Hartree/Particle)
Thermal correction to Energy= 0.498765
Thermal correction to Enthalpy= 0.499709
Thermal correction to Gibbs Free Energy= 0.412492
Sum of electronic and zero-point Energies= -1379.748536
Sum of electronic and thermal Energies= -1379.720711
Sum of electronic and thermal Enthalpies= -1379.719766
Sum of electronic and thermal Free Energies= -1379.806983

Energy in DMA:

SCF Done: E(RM06L) = -1380.25609148

Energy in NMP:

SCF Done: E(RM06L) = -1380.25577793

Cartesian coordinates:

O	-2.472330	1.062564	-1.285899
C	-2.540020	-1.138995	0.365152
C	-1.093676	-1.051804	0.595409
C	-0.229460	-0.616848	-0.395069
C	-0.612246	-0.328290	-1.790642
C	-1.704454	0.471204	-2.218086
C	-0.720103	-1.307649	2.009069
C	-1.638235	-0.933264	3.024078
C	-2.944089	-0.454913	2.641321
C	-3.400770	-0.573942	1.371192
C	-1.307640	-1.112284	4.376487
C	-0.108992	-1.709937	4.733776
C	0.763793	-2.144483	3.735250
C	0.467349	-1.938647	2.391354
C	0.250670	-0.839536	-2.777389
O	1.408470	-1.472509	-2.399522
C	1.992377	-1.007771	-1.249154
C	1.227484	-0.442695	-0.234348
C	3.392285	-1.142201	-1.158625
C	4.041628	-0.587042	-0.090300
C	3.336461	0.167485	0.889293
C	1.911933	0.265002	0.814822
C	-1.948668	0.613255	-3.588019
C	-1.121065	0.003443	-4.530078
C	-0.006644	-0.726408	-4.138115
C	1.249521	1.112910	1.734165
C	1.952683	1.794936	2.701286
C	3.351696	1.657554	2.806352
C	4.027761	0.862249	1.912199
N	-3.099222	-1.770304	-0.652841
C	-2.488640	-2.688144	-1.611224
N	-3.749398	3.315405	-2.064397
C	-2.711727	4.361842	-2.208887
H	0.178119	1.244643	1.657692
H	1.425928	2.449421	3.386007
H	3.891231	2.193881	3.577683
H	5.107354	0.773452	1.962708
H	5.119526	-0.670202	-0.009373
H	3.917559	-1.656016	-1.953362
H	0.681347	-1.162615	-4.849593
H	-1.335509	0.126030	-5.585093
H	-2.795086	1.206292	-3.911401
H	1.159743	-2.289620	1.638885
H	1.682680	-2.651975	4.003638
H	0.136687	-1.860068	5.777817
H	-2.015187	-0.805051	5.138722
H	-3.608815	-0.089654	3.417032
H	-4.430656	-0.350629	1.117233
H	-4.106657	-1.687409	-0.715645
H	-1.507531	-2.990063	-1.252219
H	-2.387647	-2.225708	-2.593685
H	-3.131163	-3.566911	-1.690428
H	-4.330328	3.286189	-2.898395
H	-4.375971	3.557692	-1.300703
H	-2.997064	1.842937	-1.660184
H	-2.107949	4.390245	-1.300995
H	-2.054842	4.100874	-3.039658
H	-3.123724	5.359324	-2.388023

From Dioxa (*P*)-**1** to Azaoxa (*P*)-**2** via intermediate (*aS*)-**A**, Attack on the *Si* face.

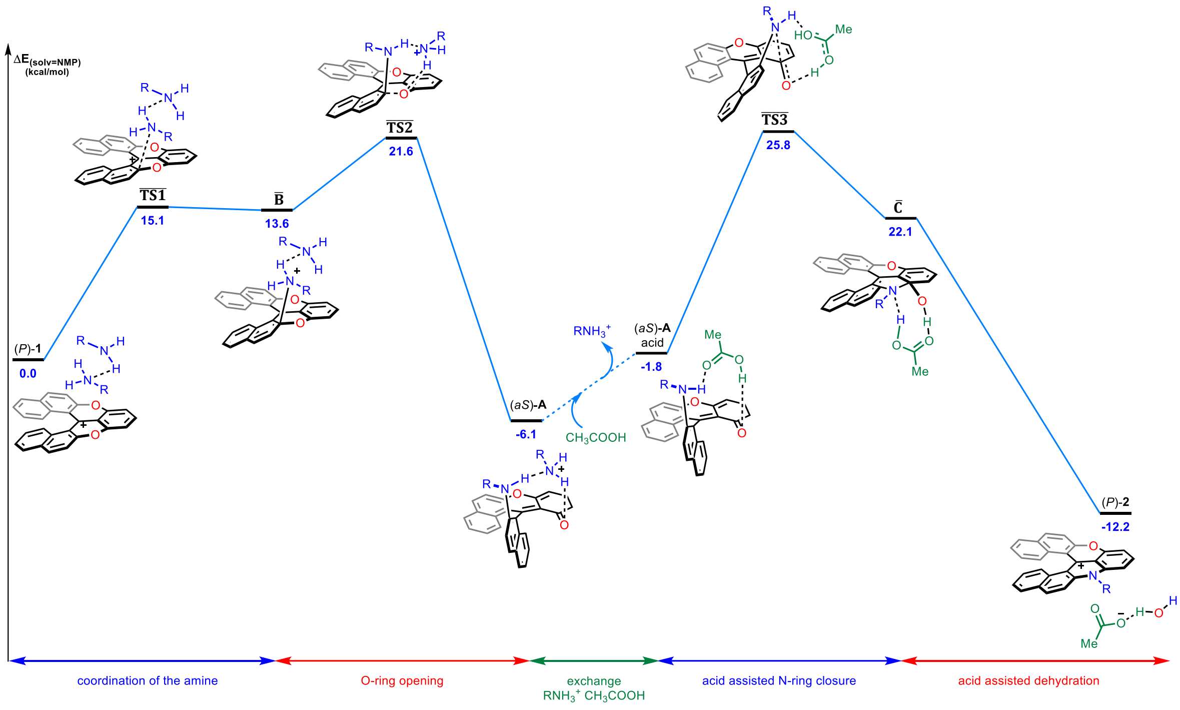

(*P*)-1

Geometry optimization in gas phase, B3LYP/6-311G**

```
 SCF Done:  E(RB3LYP) =  -1380.23031458
 Zero-point correction=                           0.469667 (Hartree/Particle)
 Thermal correction to Energy=                    0.498988
 Thermal correction to Enthalpy=                  0.499932
 Thermal correction to Gibbs Free Energy=         0.405759
 Sum of electronic and zero-point Energies=          -1379.760648
 Sum of electronic and thermal Energies=             -1379.731326
 Sum of electronic and thermal Enthalpies=           -1379.730382
 Sum of electronic and thermal Free Energies=        -1379.824556
```

Energy in DMA:

```
 SCF Done:  E(RM06L) =  -1380.26560458
```

Energy in NMP:

```
 SCF Done:  E(RM06L) =  -1380.26534425
```

Cartesian coordinates:

```
 O      0.167374     0.017450    -0.806551
 C     -0.087709     0.154003     0.517382
 C      0.980513     0.058022     1.427367
 C      2.306214    -0.200496     0.977020
 C      2.409328    -0.785883    -0.332302
 C      1.333157    -0.537259    -1.209950
 C     -1.381341     0.418491     0.936432
 C     -1.622096     0.558825     2.302866
 C     -0.597603     0.457849     3.244670
 C      0.691365     0.227028     2.794061
 C      3.348958     0.130024     1.914753
 C      2.986180     0.165982     3.276770
 O      1.699272     0.106942     3.698204
 C      3.936260     0.236446     4.314518
 C      5.255711     0.393080     3.997988
 C      5.679039     0.564026     2.648809
 C      4.723176     0.471627     1.591723
 C      5.145090     0.807622     0.288344
 C      6.457945     1.152975     0.033655
 C      7.412460     1.172294     1.064914
 C      7.022867     0.890828     2.354997
 C      1.393085    -0.826520    -2.586389
```

C	2.484821	-1.480886	-3.080698
C	3.526298	-1.938999	-2.224091
C	3.485673	-1.626562	-0.830581
C	4.448098	-2.232872	0.004548
C	5.430621	-3.052165	-0.515669
C	5.507452	-3.299915	-1.896267
C	4.560699	-2.756131	-2.734538
N	2.178748	2.723969	-0.417763
C	2.238168	3.066499	-1.846876
N	-0.060550	4.693799	0.709012
C	-1.262723	4.898888	-0.116994
H	4.424756	0.827756	-0.515503
H	6.752366	1.418274	-0.974915
H	8.441259	1.432468	0.847927
H	7.736430	0.943393	3.169489
H	5.996766	0.444643	4.787747
H	3.586986	0.179264	5.337032
H	-0.785428	0.558236	4.305268
H	-2.633112	0.746016	2.643248
H	-2.175873	0.495416	0.206417
H	4.407826	-2.083693	1.073499
H	6.146237	-3.514857	0.153607
H	6.290467	-3.935172	-2.291791
H	4.577784	-2.971072	-3.797008
H	2.542420	-1.710305	-4.138804
H	0.557854	-0.537161	-3.210308
H	3.022564	3.063734	0.036574
H	1.323810	2.725435	-2.339005
H	3.074773	2.548149	-2.324149
H	2.347885	4.139496	-2.058133
H	0.494887	5.544636	0.720061
H	1.406266	3.243564	0.011774
H	-0.330584	4.527023	1.674100
H	-1.858508	3.983399	-0.119888
H	-1.907207	5.723493	0.213448
H	-0.962182	5.096791	-1.147819

TS1

Geometry optimization in gas phase, B3LYP/6-311G**

```
SCF Done:  E(RB3LYP) =  -1380.20675625
Zero-point correction=                           0.471227 (Hartree/Particle)
Thermal correction to Energy=                    0.498305
Thermal correction to Enthalpy=                  0.499249
Thermal correction to Gibbs Free Energy=         0.413482
Sum of electronic and zero-point Energies=          -1379.735529
Sum of electronic and thermal Energies=             -1379.708451
Sum of electronic and thermal Enthalpies=           -1379.707507
Sum of electronic and thermal Free Energies=        -1379.793275
```

Energy in DMA:

SCF Done: E(RM06L) = -1380.24192136

Energy in NMP:

SCF Done: E(RM06L) = -1380.24162220

Cartesian coordinates:

C	-8.459477	13.991681	0.960384
C	-7.769647	15.173715	0.619868
C	-6.629211	15.500507	1.410427
C	-6.293848	14.726160	2.534039
C	-7.034662	13.609864	2.878464
C	-8.105648	13.232224	2.064461
C	-5.724104	16.535477	0.972973
C	-5.936488	17.242469	-0.148647
C	-7.173471	17.080859	-0.906188
C	-8.081653	15.964537	-0.571420
C	-9.128546	15.737943	-1.485762
C	-8.855480	16.103063	-2.861484
C	-7.752647	16.894836	-3.208026
O	-6.966545	17.427106	-2.225117
C	-9.582057	15.532647	-3.918605

O	-10.599385	14.669589	-3.653080
C	-11.088749	14.577776	-2.388051
C	-10.447685	15.139966	-1.273789
C	-12.298634	13.858894	-2.306734
C	-12.953551	13.786346	-1.114340
C	-12.463728	14.476281	0.029245
C	-11.218126	15.178701	-0.039797
C	-10.841571	15.929661	1.098438
C	-11.623675	15.965268	2.234265
C	-12.822101	15.235281	2.309613
C	-13.230707	14.504900	1.217868
C	-7.408333	17.154310	-4.525659
C	-8.177301	16.587193	-5.542386
C	-9.261840	15.767206	-5.250333
N	-8.068365	18.642010	-0.329833
C	-9.353131	18.984701	-0.962633
N	-6.247895	20.887358	-0.791988
C	-6.907151	22.200686	-0.626424
H	-9.919872	16.489811	1.087048
H	-11.303918	16.560371	3.083007
H	-13.418449	15.257383	3.212443
H	-14.163756	13.954733	1.245069
H	-13.884610	13.236572	-1.035637
H	-12.681266	13.399658	-3.209491
H	-9.851622	15.299690	-6.027073
H	-7.918311	16.780765	-6.576062
H	-6.545978	17.770686	-4.739709
H	-9.270522	13.641280	0.341500
H	-8.664729	12.332454	2.287925
H	-6.767757	13.019385	3.746241
H	-5.421040	15.003289	3.115043
H	-4.810310	16.682427	1.539533
H	-5.219851	17.955404	-0.534842
H	-8.174346	18.500508	0.673800
H	-9.183485	19.200310	-2.016355
H	-10.055470	18.156732	-0.870267
H	-9.795188	19.865861	-0.488000
H	-7.372917	19.414792	-0.476410
H	-5.868245	20.811097	-1.731987
H	-5.446342	20.832485	-0.168433
H	-6.240683	23.050471	-0.805544
H	-7.302043	22.282902	0.387916
H	-7.745912	22.272605	-1.320297

$\overline{\mathbf{B}}$

Geometry optimization in gas phase, B3LYP/6-311G**

SCF Done: E(RB3LYP) = -1380.20875961

Zero-point correction=	0.472437	(Hartree/Particle)
Thermal correction to Energy=	0.499725	
Thermal correction to Enthalpy=	0.500669	
Thermal correction to Gibbs Free Energy=	0.414220	
Sum of electronic and zero-point Energies=	-1379.736323	
Sum of electronic and thermal Energies=	-1379.709034	
Sum of electronic and thermal Enthalpies=	-1379.708090	
Sum of electronic and thermal Free Energies=	-1379.794540	

Energy in DMA:

SCF Done: E(RM06L) = -1380.24442446

Energy in NMP:

SCF Done: E(RM06L) = -1380.24408829

Cartesian coordinates:

C	-0.219776	-1.415959	-4.246398
C	0.343505	-1.136045	-3.007143
C	-0.388953	-0.571140	-1.947789
C	-1.725804	-0.229811	-2.235163
C	-2.309530	-0.480978	-3.467441
C	-1.551400	-1.089590	-4.466497
C	0.280940	-0.124575	-0.729065
C	1.647691	-0.635079	-0.550954

C	2.283662	-1.194048	-1.668391
O	1.676244	-1.357614	-2.874914
C	3.618339	-1.648452	-1.666498
C	4.327249	-1.639285	-0.503713
C	3.716189	-1.233973	0.713235
C	2.368694	-0.745684	0.711811
C	1.803727	-0.451299	1.975282
C	2.513095	-0.607360	3.148531
C	3.846265	-1.048361	3.132947
C	4.431377	-1.356413	1.928023
C	-0.458632	0.754115	0.075565
C	-1.955138	0.540768	0.009809
C	-2.802186	1.511058	0.742459
C	-2.267480	2.473426	1.505489
C	-0.842508	2.707917	1.585957
C	0.049717	1.894825	0.831448
C	-0.364659	3.819393	2.294801
C	0.973911	4.173970	2.250332
C	1.844470	3.429297	1.451555
C	1.391724	2.320431	0.753179
N	-2.301671	-0.921437	0.628636
C	-2.094731	-1.103792	2.088019
O	-2.494001	0.400717	-1.293089
N	-5.019734	-1.495962	-0.039716
C	-6.014047	-1.608322	1.052573
H	0.794857	-0.082639	2.034918
H	2.034561	-0.375329	4.093066
H	4.399989	-1.151488	4.058079
H	5.452840	-1.718057	1.889295
H	5.355962	-1.980423	-0.487566
H	4.034402	-2.005031	-2.599632
H	0.399303	-1.855564	-5.016974
H	-2.002732	-1.298365	-5.428478
H	-3.337144	-0.185678	-3.634508
H	2.088685	1.788939	0.122404
H	2.884570	3.720844	1.365546
H	1.332511	5.034874	2.800944
H	-1.068550	4.419213	2.861513
H	-2.921586	3.161560	2.031261
H	-3.872543	1.412179	0.606879
H	-1.718907	-1.590099	0.120640
H	-2.673699	-0.354215	2.622581
H	-1.039998	-0.998883	2.329081
H	-2.433265	-2.101629	2.367482
H	-5.040527	-2.342970	-0.603524
H	-3.317937	-1.136270	0.378786
H	-5.289061	-0.746623	-0.673398
H	-6.003336	-0.693299	1.646978
H	-7.033938	-1.773871	0.693175
H	-5.740715	-2.438744	1.705234

TS2

Geometry optimization in gas phase, B3LYP/6-311G**

SCF Done: E(RB3LYP) = -1380.18838297

Zero-point correction= 0.470724 (Hartree/Particle)
Thermal correction to Energy= 0.497320
Thermal correction to Enthalpy= 0.498264
Thermal correction to Gibbs Free Energy= 0.414813
Sum of electronic and zero-point Energies= -1379.717659
Sum of electronic and thermal Energies= -1379.691063
Sum of electronic and thermal Enthalpies= -1379.690119
Sum of electronic and thermal Free Energies= -1379.773570

Energy in DMA:

SCF Done: E(RM06L) = -1380.23143125

Energy in NMP:

SCF Done: E(RM06L) = -1380.23103620

Cartesian coordinates:

C	8.818833	15.459914	-4.897941

C	9.333262	15.544459	-3.615249
C	8.597082	16.025995	-2.506095
C	7.240495	16.439236	-2.766054
C	6.754068	16.426052	-4.080458
C	7.523323	15.916824	-5.119049
C	9.291784	16.368562	-1.288137
C	10.667908	15.916256	-1.151914
C	11.274663	15.340676	-2.281084
O	10.643095	15.209572	-3.469864
C	12.598767	14.859191	-2.303496
C	13.340328	14.898134	-1.161676
C	12.784149	15.375812	0.056417
C	11.442358	15.877799	0.088116
C	10.943055	16.266319	1.353216
C	11.712528	16.183675	2.496890
C	13.037677	15.723675	2.447149
C	13.557882	15.323331	1.239271
C	8.572366	17.216190	-0.383032
C	7.172257	16.876441	-0.171310
C	6.362718	17.810705	0.590553
C	6.867267	18.976384	1.050053
C	8.222424	19.371561	0.807592
C	9.078816	18.501773	0.070575
C	8.680419	20.632416	1.235558
C	9.957669	21.063057	0.939493
C	10.794040	20.233750	0.179404
C	10.365844	18.991166	-0.250086
N	6.834615	15.469067	0.148444
C	7.106247	15.085500	1.555838
O	6.428064	16.806149	-1.789105
N	4.362128	15.238268	-1.003447
C	3.104989	15.371251	-0.214638
H	9.939067	16.643916	1.439563
H	11.284578	16.489320	3.444695
H	13.636855	15.679186	3.348392
H	14.572878	14.947460	1.174606
H	14.364024	14.541402	-1.164564
H	12.984838	14.471655	-3.237082
H	9.444844	15.085052	-5.696083
H	7.108553	15.877840	-6.119401
H	5.753201	16.799125	-4.260023
H	11.036083	18.396590	-0.853869
H	11.789448	20.570948	-0.085840
H	10.303282	22.034142	1.271784
H	8.002038	21.270576	1.791714
H	6.231797	19.649567	1.616401
H	5.332547	17.540452	0.793802
H	7.410223	14.875664	-0.447321
H	6.495537	15.688754	2.226424
H	8.156534	15.214833	1.829351
H	6.837009	14.035460	1.683151
H	4.343722	14.411949	-1.601245
H	5.287761	15.215257	-0.406721
H	4.560365	16.047946	-1.607115
H	3.167002	16.271197	0.394013
H	2.250061	15.444229	-0.884855
H	2.994172	14.501684	0.430880

(*aS*)-**A**-RNH_3^+

Geometry optimization in gas phase, B3LYP/6-311G**

```
SCF Done:  E(RB3LYP) =  -1380.23564048
Zero-point correction=                           0.471654 (Hartree/Particle)
Thermal correction to Energy=                    0.499401
Thermal correction to Enthalpy=                  0.500345
Thermal correction to Gibbs Free Energy=         0.412762
Sum of electronic and zero-point Energies=          -1379.763987
Sum of electronic and thermal Energies=             -1379.736240
Sum of electronic and thermal Enthalpies=           -1379.735295
```

Sum of electronic and thermal Free Energies= -1379.822878

Energy in DMA:

SCF Done: E(RM06L) = -1380.27576977

Energy in NMP:

SCF Done: E(RM06L) = -1380.27539064

Cartesian coordinates:

C	0.089767	-0.843924	-4.316585
C	0.419195	-0.895874	-2.989329
C	-0.380958	-0.330175	-1.936225
C	-1.713616	0.205295	-2.328693
C	-1.981372	0.294344	-3.725288
C	-1.118992	-0.211969	-4.669057
C	0.145124	-0.269745	-0.638063
C	1.434828	-0.859605	-0.347892
C	2.054503	-1.556801	-1.409455
O	1.572330	-1.538335	-2.662219
C	3.214597	-2.342155	-1.270728
C	3.805766	-2.431865	-0.047670
C	3.312235	-1.699674	1.067688
C	2.151249	-0.866445	0.936552
C	1.817650	-0.079854	2.064304
C	2.536190	-0.153663	3.243433
C	3.632076	-1.017629	3.376441
C	4.015200	-1.771177	2.291975
C	-0.626789	0.436518	0.437632
C	-1.396567	-0.271315	1.347062
C	-2.041023	0.415866	2.408398
C	-1.928019	1.775238	2.537131
C	-1.178386	2.538445	1.607797
C	-0.514934	1.861732	0.536554
C	-1.061159	3.946549	1.724979
C	-0.313373	4.667607	0.827640
C	0.354098	4.002399	-0.223501
C	0.259549	2.638142	-0.365474
N	-1.647288	-1.668033	1.187867
C	-1.543877	-2.532872	2.382697
O	-2.574581	0.554095	-1.470997
N	-4.178971	-1.006935	-0.183855
C	-5.219385	-0.286037	0.604614
H	1.006264	0.621667	2.018299
H	2.247498	0.482163	4.072255
H	4.181141	-1.070740	4.308536
H	4.880312	-2.421898	2.351650
H	4.688794	-3.046823	0.084485
H	3.593383	-2.855677	-2.144378
H	0.748870	-1.279809	-5.054355
H	-1.381587	-0.144491	-5.719037
H	-2.920250	0.751709	-4.010718
H	0.780770	2.154511	-1.181566
H	0.947417	4.574727	-0.926994
H	-0.229886	5.743430	0.923867
H	-1.573980	4.445072	2.540344
H	-2.417284	2.283883	3.360636
H	-2.622889	-0.143156	3.130985
H	-1.063369	-2.045340	0.449746
H	-2.359316	-2.330198	3.076964
H	-0.594553	-2.402812	2.911538
H	-1.633877	-3.571463	2.062607
H	-4.592073	-1.724463	-0.777909
H	-3.447495	-1.449995	0.414955
H	-3.607987	-0.328734	-0.798627
H	-4.724829	0.485331	1.190672
H	-5.927440	0.175925	-0.081054
H	-5.739196	-0.981416	1.262138

(*aS*)-**A**-CH_3COO^-

Geometry optimization in gas phase, B3LYP/6-311G**

SCF Done: E(RB3LYP) = -1513.087803

```
 Zero-point correction=                           0.454193 (Hartree/Particle)
 Thermal correction to Energy=                    0.483626
 Thermal correction to Enthalpy=                  0.484570
 Thermal correction to Gibbs Free Energy=         0.391628
 Sum of electronic and zero-point Energies=          -1512.633610
 Sum of electronic and thermal Energies=             -1512.604177
 Sum of electronic and thermal Enthalpies=           -1512.603233
 Sum of electronic and thermal Free Energies=        -1512.696175
```

Energy in DMA:

```
 SCF Done:  E(RM06L) =  -1513.08941488
```

Energy in NMP:

```
 SCF Done:  E(RM06L) =  -1513.08928958
Cartesian coordinates:
 O    -1.145804     0.812438     3.298055
 C     0.449620    -0.469947     2.004945
 C     1.733665    -1.172470     2.072126
 C     0.013069     0.133253     3.195474
 C    -1.606688     0.413133     0.967629
 C    -0.433561    -0.343027     0.849812
 C    -1.970733     0.986731     2.228245
 C    -0.891218    -2.487198    -0.180250
 C     3.665737    -1.915473     3.433733
 H     4.151737    -1.944678     4.403093
 C    -0.283574    -1.257599    -0.339477
 C    -3.739400     1.366811     0.110842
 C     2.426876    -1.240405     3.327426
 C     3.590957    -2.435992     1.103282
 H     4.037953    -2.888712     0.225597
 C    -0.056159    -3.264237    -2.298791
 H     0.048319    -4.036130    -3.053705
 C     4.246818    -2.512603     2.341880
 H     5.196526    -3.026789     2.431482
 C    -3.134390     1.674000     2.449802
 C    -4.020623     1.844261     1.364075
 H    -4.948566     2.380595     1.533411
 C    -2.515221     0.678116    -0.177696
 C     0.710334     0.066639     4.421012
 H     0.272409     0.558989     5.279391
 C     1.885819    -0.613019     4.482017
 H     2.431654    -0.679509     5.416665
 C     0.615125    -2.028548    -2.492910
 C     1.997933     0.388429    -2.828752
 H     2.514824     1.324004    -2.995888
 C     2.379469    -1.788529     0.972964
 H     1.933085    -1.758669    -0.002209
 C     2.178686    -0.668756    -3.743245
 H     2.856369    -0.533974    -4.579216
 C     1.479734    -1.839828    -3.602960
 H     1.592952    -2.645764    -4.319562
 C     1.124070     0.269618    -1.762530
 N     0.861341     1.313691    -0.860158
 C     0.460347    -0.981385    -1.530150
 C    -0.803426    -3.484683    -1.172987
 H    -1.311919    -4.430298    -1.023456
 H    -4.416144     1.528537    -0.718659
 H    -3.353726     2.054950     3.437633
 O    -2.185608     0.356928    -1.344601
 H    -0.107553     1.609723    -0.960816
 C     1.731914     2.483551    -0.858494
 H     1.680499     3.062120    -1.789469
 H     1.421503     3.140237    -0.044078
 H     2.765372     2.177618    -0.679941
 H    -1.445632    -2.693219     0.728189
 O    -1.087597     3.185683    -2.278184
 C    -1.934219     3.082447    -3.144753
 O    -2.753423     2.044078    -3.275661
 H    -2.560548     1.361183    -2.565162
 C    -2.173787     4.130727    -4.207006
```

H	-1.528726	4.989378	-4.032956
H	-1.968665	3.704478	-5.192135
H	-3.221977	4.437433	-4.198456

TS3

Geometry optimization in gas phase, B3LYP/6-311G**

```
SCF Done:  E(RB3LYP) =  -1513.04328424
Zero-point correction=                           0.452349 (Hartree/Particle)
Thermal correction to Energy=                    0.479927
Thermal correction to Enthalpy=                  0.480871
Thermal correction to Gibbs Free Energy=         0.395340
Sum of electronic and zero-point Energies=          -1512.590935
Sum of electronic and thermal Energies=             -1512.563357
Sum of electronic and thermal Enthalpies=           -1512.562413
Sum of electronic and thermal Free Energies=        -1512.647945
```

Energy in DMA:

SCF Done: E(RM06L) = -1513.04545184

Energy in NMP:

SCF Done: E(RM06L) = -1513.04531466

Cartesian coordinates:

O	-1.436854	0.678469	2.620792
C	0.248258	-0.581963	1.457129
C	1.491542	-1.313011	1.604947
C	-0.395545	-0.192513	2.628681
C	-1.296642	0.838567	0.214830
C	-0.391244	-0.187591	0.187290
C	-1.709371	1.398613	1.480389
C	-0.217019	-2.362454	-0.860954
C	3.066930	-2.522614	3.072162
H	3.311927	-2.889363	4.063370
C	-0.109984	-0.991791	-1.036919
C	-2.535847	2.777653	-0.830845
C	1.863821	-1.792584	2.904255
C	3.597739	-2.207963	0.753742
H	4.285891	-2.334872	-0.074280
C	0.577115	-2.823853	-3.068850
H	0.870504	-3.513661	-3.852499
C	3.919892	-2.738303	2.018448
H	4.842130	-3.289608	2.159886
C	-2.425828	2.540759	1.600227
C	-2.806452	3.244453	0.403588
H	-3.366721	4.167377	0.509874
C	-1.869186	1.462318	-1.036519
C	-0.036980	-0.672788	3.904729
H	-0.638009	-0.370233	4.752750
C	1.051056	-1.487924	4.028886
H	1.336781	-1.873121	5.001437
C	0.678254	-1.433035	-3.323082
C	0.686619	1.267553	-3.989563
H	0.591818	2.306119	-4.280710
C	2.421633	-1.521760	0.551791
H	2.220171	-1.121330	-0.429482
C	1.173184	0.327336	-4.913853
H	1.519077	0.658158	-5.885786
C	1.150342	-1.001606	-4.587957
H	1.470024	-1.753194	-5.301380
C	0.258143	0.894239	-2.731375
N	-0.298046	1.966087	-1.920393
C	0.271819	-0.477559	-2.323828
C	0.093588	-3.283289	-1.873676
H	-0.019932	-4.344744	-1.688721
H	-2.882700	3.285376	-1.718570
H	-2.731546	2.886761	2.578202
O	-2.431624	0.643390	-1.895311
H	-0.757575	2.619391	-2.608899
C	0.672359	2.702249	-1.087472
H	1.420208	3.189186	-1.717870
H	0.132067	3.461055	-0.519374

H	1.174539	2.022544	-0.399573
H	-0.533963	-2.737621	0.103978
O	-1.618710	3.326357	-3.883521
C	-2.636040	2.782496	-4.381737
O	-3.271184	1.797980	-3.886616
H	-2.820714	1.181875	-2.777496
C	-3.173907	3.320855	-5.699245
H	-2.737930	4.292804	-5.926886
H	-2.918498	2.617733	-6.497634
H	-4.262510	3.386390	-5.661174

$\bar{C}$

Geometry optimization in gas phase, B3LYP/6-311G**

SCF Done: E(RB3LYP) = -1513.05496176

Zero-point correction= 0.455649 (Hartree/Particle)

Thermal correction to Energy= 0.483665

Thermal correction to Enthalpy= 0.484610

Thermal correction to Gibbs Free Energy= 0.398121

Sum of electronic and zero-point Energies= -1512.599313

Sum of electronic and thermal Energies= -1512.571296

Sum of electronic and thermal Enthalpies= -1512.570352

Sum of electronic and thermal Free Energies= -1512.656840

Energy in DMA:

SCF Done: E(RM06L) = -1513.05143314

Energy in NMP:

SCF Done: E(RM06L) = -1513.05132824

Cartesian coordinates:

O	-1.134628	0.474708	3.404745
C	0.527195	-0.826493	2.256071
C	1.760336	-1.570022	2.404425
C	-0.096594	-0.402025	3.423324
C	-0.964469	0.635615	0.996709
C	-0.106385	-0.427277	0.982084
C	-1.417248	1.176754	2.253983
C	0.016137	-2.632884	-0.038878
C	3.342142	-2.765822	3.875369
H	3.599178	-3.111043	4.871389
C	0.107376	-1.264256	-0.230060
C	-2.278423	2.527581	-0.057478
C	2.147179	-2.021470	3.709817
C	3.836687	-2.519364	1.539820
H	4.508628	-2.678478	0.703978
C	0.768525	-3.100846	-2.264103
H	1.065108	-3.794152	-3.043713
C	4.172537	-3.022123	2.812882
H	5.088517	-3.584441	2.952658
C	-2.203352	2.273930	2.365306
C	-2.626121	2.952684	1.167071
H	-3.270561	3.819461	1.270238
C	-1.461712	1.274868	-0.303795
C	0.277151	-0.850353	4.706934
H	-0.308138	-0.518388	5.555106
C	1.357149	-1.675611	4.838254
H	1.652493	-2.038537	5.816546
C	0.864026	-1.709641	-2.525077
C	0.903356	0.999435	-3.149190
H	0.849950	2.040882	-3.439838
C	2.668640	-1.819650	1.340447
H	2.455700	-1.438229	0.353855
C	1.451178	0.071329	-4.051389
H	1.863596	0.418867	-4.991631
C	1.388817	-1.266239	-3.763631
H	1.734369	-2.006840	-4.476433
C	0.370539	0.617951	-1.930327
N	-0.274878	1.667999	-1.170753
C	0.432451	-0.758152	-1.534118
C	0.303156	-3.555173	-1.059369
H	0.200623	-4.616794	-0.867505

H	-2.663382	3.015738	-0.942663
H	-2.547335	2.590321	3.340754
O	-2.231549	0.332031	-1.002831
H	-1.048970	2.660765	-2.485590
C	0.671494	2.582907	-0.500492
H	1.349686	3.009577	-1.239744
H	0.109799	3.393496	-0.034382
H	1.265905	2.076026	0.268815
H	-0.256204	-3.005594	0.940462
O	-1.360844	3.215820	-3.263717
C	-2.470805	2.701291	-3.793283
O	-3.061150	1.744348	-3.329217
H	-2.592478	0.763348	-1.802754
C	-2.916411	3.440886	-5.027738
H	-3.012785	4.506250	-4.808232
H	-2.158647	3.336527	-5.808185
H	-3.864651	3.038452	-5.376497

(*P*)-**2**

Geometry optimization in gas phase, B3LYP/6-311G**

```
 SCF Done:  E(RB3LYP) =   -1207.8989355
  Zero-point correction=                           0.380071 (Hartree/Particle)
 Thermal correction to Energy=                     0.400775
 Thermal correction to Enthalpy=                   0.401719
 Thermal correction to Gibbs Free Energy=          0.332212
 Sum of electronic and zero-point Energies=           -1207.518864
 Sum of electronic and thermal Energies=              -1207.498161
 Sum of electronic and thermal Enthalpies=            -1207.497216
 Sum of electronic and thermal Free Energies=         -1207.566723
```

Energy in DMA:

SCF Done: E(RM06L) = -1207.90448807

Energy in NMP:

SCF Done: E(RM06L) = -1207.90422854

Cartesian coordinates:

O	-0.916509	0.792064	3.699631
C	0.428740	-0.776476	2.437317
C	1.691097	-1.494694	2.409628
C	0.110060	-0.093957	3.622389
C	-1.313330	0.531145	1.356307
C	-0.509912	-0.641325	1.339777
C	-1.502506	1.244363	2.561074
C	3.644960	-2.359223	3.632811
H	4.169340	-2.486089	4.573186
C	-0.741634	-1.543050	0.254685
C	-2.778768	2.138694	0.255747
C	2.412476	-1.665040	3.632256
C	3.511860	-2.614655	1.248440
H	3.954879	-2.951483	0.318685
C	4.186452	-2.836036	2.461639
H	5.135787	-3.357398	2.468489
C	-2.318601	2.356022	2.636392
C	-2.949290	2.790422	1.470923
H	-3.597882	3.656771	1.514068
C	-1.965776	1.000633	0.184416
C	0.808148	-0.285786	4.829960
H	0.454511	0.222751	5.717143
C	1.916054	-1.084218	4.833388
H	2.464970	-1.242338	5.754776
C	2.293718	-1.966965	1.222931
H	1.813000	-1.800605	0.270321
N	-1.725285	0.310655	-0.993127
H	-3.310689	2.497271	-0.612806
H	-2.454945	2.859073	3.584137
C	-2.043898	0.957025	-2.276566
H	-1.308356	0.666118	-3.022178
H	-3.045299	0.690324	-2.623374
H	-1.978188	2.034069	-2.151630
C	-1.258883	-0.984056	-0.954523

C	-0.529079	-2.982918	0.313560
C	-0.580353	-3.741694	-0.890498
C	-1.275964	-1.771133	-2.142202
C	-0.914031	-3.084191	-2.107509
C	-0.364889	-3.691111	1.522884
C	-0.199682	-5.062674	1.531262
C	-0.181364	-5.794931	0.333282
C	-0.379525	-5.139389	-0.861501
H	-1.616000	-1.345471	-3.073369
H	-0.942092	-3.666821	-3.021731
H	-0.413205	-5.691034	-1.794203
H	-0.038958	-6.868399	0.353620
H	-0.089909	-5.578371	2.477842
H	-0.392477	-3.166322	2.466351

Isomerization from (*aR*)-**A** to (*aS*)-**A**

Reaction on the *Re* face of (*aR*)-**A·H**$^+$ *via* intermediate **D**.

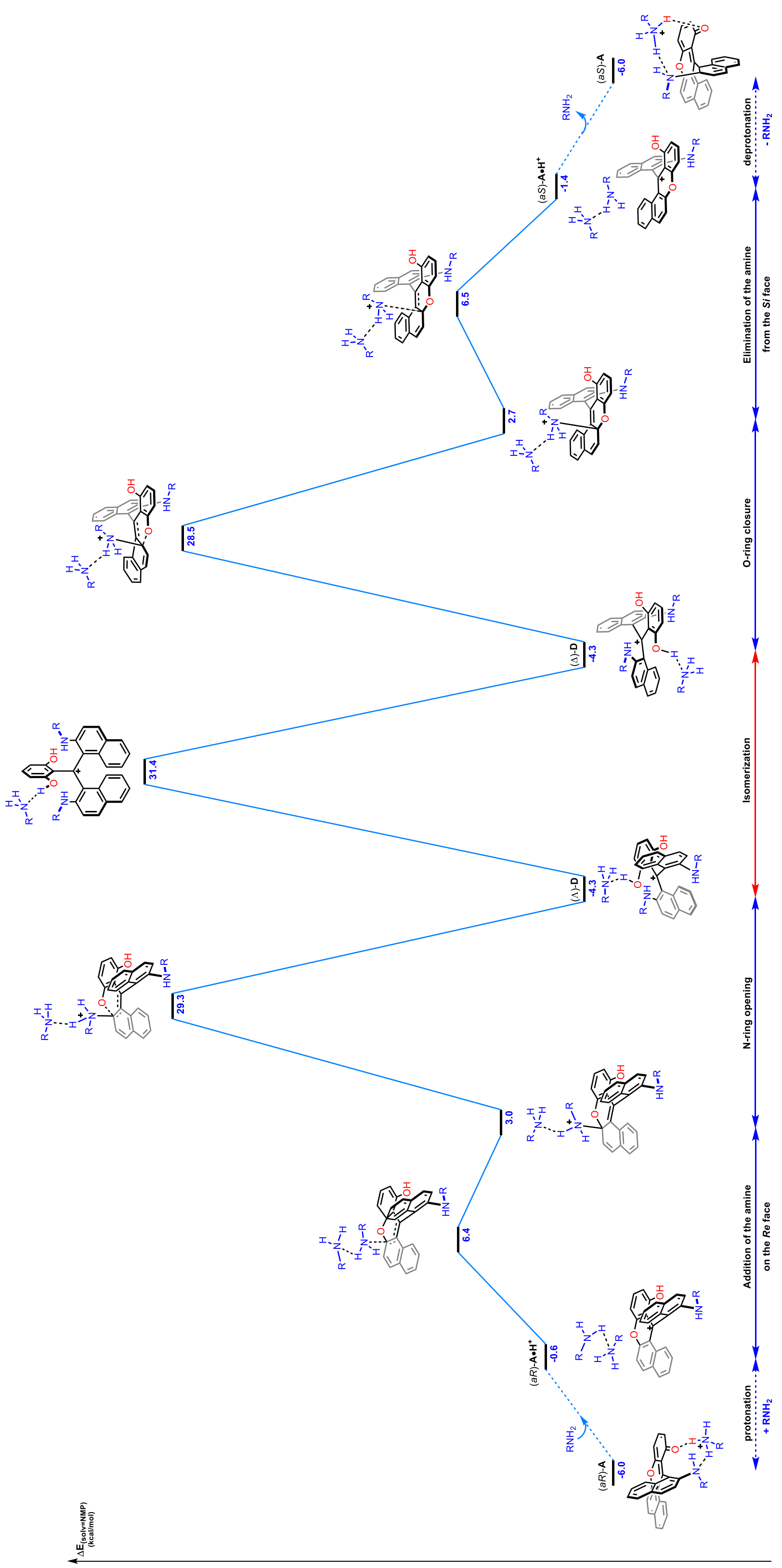

(*aR*)-**A·H**$^+$

Geometry optimization in gas phase, B3LYP/6-311G**

```
 SCF Done:  E(RB3LYP) =  -1476.12452293
 Zero-point correction=                           0.535228 (Hartree/Particle)
 Thermal correction to Energy=                    0.569439
 Thermal correction to Enthalpy=                  0.570383
 Thermal correction to Gibbs Free Energy=         0.463791
 Sum of electronic and zero-point Energies=          -1475.589295
 Sum of electronic and thermal Energies=             -1475.555084
 Sum of electronic and thermal Enthalpies=           -1475.554139
 Sum of electronic and thermal Free Energies=        -1475.660732
```

Energy in DMA:

```
 SCF Done:  E(RM06L) =  -1476.14809076
```

Energy in NMP:

```
 SCF Done:  E(RM06L) =  -1476.14779874
```

Cartesian coordinates:

C	2.886654	1.696059	-2.215554
C	2.130078	0.833245	-1.443180
C	1.341586	1.230107	-0.321307
C	1.342720	2.654070	-0.038454
C	2.100884	3.515049	-0.823850
C	2.862806	3.044714	-1.889262
C	0.633134	0.230635	0.406343
C	0.700208	-1.134078	0.000318
C	1.478073	-1.410944	-1.158659
O	2.176671	-0.467570	-1.790343
C	1.620440	-2.686426	-1.733067
C	0.983726	-3.740122	-1.154315
C	0.198790	-3.583166	0.023390
C	0.045164	-2.295377	0.634582
C	-0.725128	-2.255673	1.816278
C	-1.309280	-3.393765	2.347014
C	-1.163245	-4.641549	1.731539
C	-0.411969	-4.726987	0.581006
C	-0.188367	0.658803	1.582715
C	0.411079	0.768576	2.850363
C	-0.408861	1.155056	3.953901
C	-1.745576	1.395640	3.789792
C	-2.381401	1.282358	2.525824
C	-1.590377	0.903475	1.397482
C	-3.763982	1.536937	2.354933
C	-4.353234	1.421420	1.118953
C	-3.572876	1.047278	0.004346
C	-2.225309	0.794855	0.131795
N	1.748348	0.528917	3.032373
C	2.426502	0.574440	4.319533
N	-0.691061	0.037931	-3.160476
C	-1.013682	1.370531	-3.696518
O	0.652890	3.228586	0.946811
N	0.703611	-1.334674	-5.700061
C	-0.073483	-2.484196	-6.195737
H	-0.879167	-1.330476	2.337788
H	-1.891461	-3.305692	3.256669
H	-1.629282	-5.522901	2.154503
H	-0.272997	-5.680386	0.084112
H	1.074533	-4.730764	-1.585619
H	2.227725	-2.782332	-2.622475
H	3.461144	1.305173	-3.043687
H	3.442042	3.747464	-2.476193
H	2.074315	4.567936	-0.575810
H	-1.643836	0.513704	-0.739809
H	-4.043472	0.959623	-0.968381
H	-5.411026	1.619058	0.995713
H	-4.350204	1.827977	3.219734
H	-2.342612	1.684064	4.648193
H	0.034940	1.249950	4.935173
H	2.241815	0.066935	2.287341
H	0.139685	2.559100	1.434688

H	2.040880	-0.169162	5.026414
H	2.346072	1.565875	4.772319
H	3.484136	0.371997	4.154712
H	-0.141239	-0.473409	-3.857712
H	-1.551768	-0.492661	-3.052987
H	-1.550402	1.361459	-4.655437
H	-1.622609	1.925743	-2.978537
H	-0.089155	1.935780	-3.838324
H	0.558962	-0.537666	-6.313839
H	1.696593	-1.543303	-5.751177
H	0.164094	-2.785498	-7.223676
H	0.095941	-3.343539	-5.543050
H	-1.137974	-2.244858	-6.150536

Structure 2 (TS)

Geometry optimization in gas phase, B3LYP/6-311G**

```
 SCF Done:  E(RB3LYP) =  -1476.11405760
 Zero-point correction=                           0.536174 (Hartree/Particle)
 Thermal correction to Energy=                    0.568560
 Thermal correction to Enthalpy=                  0.569504
 Thermal correction to Gibbs Free Energy=         0.470299
 Sum of electronic and zero-point Energies=          -1475.577884
 Sum of electronic and thermal Energies=             -1475.545498
 Sum of electronic and thermal Enthalpies=           -1475.544554
 Sum of electronic and thermal Free Energies=        -1475.643759
```

Energy in DMA:

SCF Done: E(RM06L) = -1476.13703141

Energy in NMP:

SCF Done: E(RM06L) = -1476.13670161

Cartesian coordinates:

C	2.718879	2.165589	-2.366449
C	1.980378	1.293678	-1.592146
C	1.251127	1.667579	-0.441642
C	1.365704	3.042554	-0.058905
C	2.097108	3.935506	-0.851070
C	2.760088	3.506081	-1.987400
C	0.484943	0.640481	0.263104
C	0.444866	-0.649340	-0.227372
C	1.219908	-0.977692	-1.449385
O	2.054532	-0.033694	-1.961690
C	1.900692	-2.289236	-1.462862
C	1.449229	-3.284958	-0.687511
C	0.282710	-3.113576	0.159307
C	-0.257603	-1.812157	0.369028
C	-1.440010	-1.713254	1.116836
C	-2.037044	-2.838679	1.676100
C	-1.470749	-4.102198	1.513742
C	-0.319483	-4.232437	0.752785
C	-0.205952	0.989629	1.554540
C	0.410153	0.645441	2.771628
C	-0.271494	0.937467	3.992644
C	-1.508079	1.520359	3.989379
C	-2.164345	1.874830	2.782180
C	-1.499467	1.610954	1.542694
C	-3.443143	2.480353	2.778199
C	-4.063644	2.823319	1.599634
C	-3.408085	2.576989	0.375034
C	-2.164450	1.988620	0.344363
N	1.641824	0.052912	2.806646
C	2.339438	-0.349090	4.016018
N	-0.034203	-1.204920	-2.774378
C	-0.985666	-0.096677	-3.010187
O	0.809676	3.581535	1.035351
N	1.366593	-1.781891	-5.316137
C	0.503381	-1.757085	-6.517616
H	-1.918269	-0.756023	1.251542
H	-2.954560	-2.724198	2.241242
H	-1.931920	-4.972650	1.961044

```
H     0.122875    -5.211207     0.597244
H     1.939222    -4.252955    -0.696861
H     2.761562    -2.384509    -2.114525
H     3.263194     1.789666    -3.223691
H     3.327587     4.215130    -2.577738
H     2.136850     4.967013    -0.528303
H    -1.673198     1.834750    -0.608678
H    -3.889511     2.861930    -0.554071
H    -5.041068     3.286383     1.607461
H    -3.928885     2.670894     3.729145
H    -2.003401     1.724093     4.931699
H     0.197279     0.684331     4.933592
H     2.056628    -0.209018     1.930015
H     0.347502     2.900406     1.550548
H     1.787411    -1.106220     4.583758
H     2.537693     0.503775     4.672159
H     3.300948    -0.777116     3.729357
H     0.507433    -1.419791    -3.648163
H    -0.531477    -2.048375    -2.497097
H    -1.721366    -0.384165    -3.764426
H    -1.502506     0.147309    -2.084389
H    -0.435800     0.775744    -3.363971
H     2.122649    -1.109795    -5.429114
H     1.820886    -2.690128    -5.247797
H     1.039136    -1.976375    -7.447264
H    -0.295717    -2.492454    -6.402465
H     0.043110    -0.773074    -6.611975
```

Structure 3

Geometry optimization in gas phase, B3LYP/6-311G**

```
SCF Done:  E(RB3LYP) =  -1476.11793627
Zero-point correction=                           0.538139 (Hartree/Particle)
Thermal correction to Energy=                    0.570234
Thermal correction to Enthalpy=                  0.571178
Thermal correction to Gibbs Free Energy=         0.473339
Sum of electronic and zero-point Energies=          -1475.579797
Sum of electronic and thermal Energies=             -1475.547703
Sum of electronic and thermal Enthalpies=           -1475.546758
Sum of electronic and thermal Free Energies=        -1475.644597
```

Energy in DMA:

```
SCF Done:  E(RM06L) =  -1476.14237922
```

Energy in NMP:

```
SCF Done:  E(RM06L) =  -1476.14201854
```

Cartesian coordinates:

```
C     2.541190     2.098450    -2.470107
C     1.789849     1.207264    -1.728831
C     1.124299     1.540456    -0.530083
C     1.311869     2.879403    -0.068391
C     2.064925     3.790255    -0.819211
C     2.667202     3.406858    -2.002825
C     0.341454     0.502127     0.152889
C     0.255714    -0.754963    -0.379827
C     0.982518    -1.086894    -1.655870
O     1.765748    -0.084313    -2.221051
C     1.818989    -2.331547    -1.527318
C     1.426662    -3.313741    -0.707022
C     0.196858    -3.205482     0.069604
C    -0.422826    -1.933663     0.217648
C    -1.637434    -1.870622     0.915190
C    -2.200748    -3.011796     1.479596
C    -1.563974    -4.246683     1.370251
C    -0.371276    -4.338245     0.664946
C    -0.318133     0.800411     1.471392
C     0.304795     0.381194     2.661173
C    -0.357978     0.623293     3.904150
C    -1.583033     1.226143     3.945501
C    -2.246551     1.653801     2.765900
C    -1.599263     1.442730     1.507505
```

C	-3.515883	2.279327	2.806213
C	-4.139892	2.693505	1.653326
C	-3.502376	2.499182	0.410499
C	-2.268607	1.892462	0.336787
N	1.517656	-0.248899	2.648141
C	2.275966	-0.623537	3.829394
N	-0.102576	-1.410773	-2.782401
C	-1.042275	-0.304773	-3.111453
O	0.808225	3.367916	1.076731
N	1.270424	-2.130645	-5.157265
C	0.461472	-2.153281	-6.399024
H	-2.157888	-0.929624	1.008485
H	-3.143888	-2.931648	2.006937
H	-1.999068	-5.129846	1.822170
H	0.128818	-5.295177	0.561772
H	2.013431	-4.222038	-0.618226
H	2.727520	-2.367142	-2.115631
H	3.030856	1.761293	-3.373954
H	3.251843	4.124042	-2.566193
H	2.164517	4.795038	-0.430160
H	-1.786884	1.779957	-0.627016
H	-3.986854	2.840961	-0.497225
H	-5.109805	3.173647	1.695435
H	-3.988922	2.429772	3.770734
H	-2.064783	1.391868	4.903083
H	0.117920	0.316756	4.825574
H	1.968969	-0.364798	1.757238
H	0.315481	2.678386	1.551482
H	1.739640	-1.356061	4.440688
H	2.522955	0.240963	4.455345
H	3.209770	-1.081411	3.504861
H	0.430109	-1.703807	-3.665815
H	-0.623769	-2.224490	-2.450244
H	-1.724879	-0.644317	-3.890173
H	-1.606212	-0.024544	-2.224298
H	-0.467790	0.544880	-3.474168
H	2.034667	-1.466599	-5.260089
H	1.714058	-3.037015	-5.026546
H	1.042694	-2.418636	-7.286766
H	-0.348024	-2.876692	-6.289459
H	0.019366	-1.168891	-6.559963

Structure 4 (TS)

Geometry optimization in gas phase, B3LYP/6-311G**

SCF Done: E(RB3LYP) = -1476.06706173

Zero-point correction= 0.536996 (Hartree/Particle)
Thermal correction to Energy= 0.568170
Thermal correction to Enthalpy= 0.569114
Thermal correction to Gibbs Free Energy= 0.475414
Sum of electronic and zero-point Energies= -1475.530065
Sum of electronic and thermal Energies= -1475.498892
Sum of electronic and thermal Enthalpies= -1475.497948
Sum of electronic and thermal Free Energies= -1475.591647

Energy in DMA:

SCF Done: E(RM06L) = -1476.10057688

Energy in NMP:

SCF Done: E(RM06L) = -1476.10020755

Cartesian coordinates:

C	11.081756	18.291411	-1.549842
C	9.733817	17.849664	-1.487129
C	8.779334	18.602431	-0.658772
C	9.136529	19.981141	-0.380901
C	10.411425	20.440888	-0.628661
C	11.384760	19.577653	-1.165056
C	7.726307	17.904068	0.005824
C	7.368945	16.569670	-0.403895
C	7.559650	16.205282	-1.775166
O	9.374327	16.829882	-2.174367

C	7.556535	14.834411	-2.184799
C	7.359057	13.842786	-1.284116
C	7.084287	14.123241	0.083661
C	7.065660	15.477112	0.545148
C	6.787967	15.655223	1.920227
C	6.543211	14.586445	2.763381
C	6.566665	13.266779	2.294858
C	6.838615	13.047665	0.964202
C	7.136203	18.528879	1.226039
C	7.967235	18.869504	2.325792
C	7.393469	19.543693	3.446303
C	6.063647	19.841367	3.487375
C	5.181745	19.470215	2.439194
C	5.713405	18.776247	1.305530
C	3.800130	19.767228	2.512601
C	2.935789	19.383700	1.515493
C	3.440748	18.674122	0.409709
C	4.783602	18.382882	0.305730
N	9.287492	18.537539	2.368576
C	10.209387	18.892486	3.435883
N	7.026963	17.026590	-2.939708
C	6.808093	18.505420	-2.935588
O	8.247306	20.886102	0.082586
N	8.727694	16.547512	-5.072626
C	8.928128	17.636522	-6.057173
H	6.744417	16.642029	2.341892
H	6.324569	14.783973	3.806435
H	6.373003	12.438849	2.965555
H	6.868319	12.039235	0.566558
H	7.377201	12.808307	-1.609260
H	7.721721	14.607074	-3.230774
H	11.800242	17.655364	-2.050122
H	12.388659	19.956428	-1.322396
H	10.641705	21.471730	-0.394660
H	5.124096	17.801129	-0.538345
H	2.761518	18.343448	-0.368120
H	1.879315	19.612222	1.581384
H	3.433705	20.303392	3.381314
H	5.658709	20.365420	4.346342
H	8.028570	19.822358	4.275254
H	9.677630	18.053254	1.578581
H	7.454975	20.437658	0.412397
H	9.919284	18.445422	4.391817
H	10.289485	19.976696	3.563395
H	11.194623	18.510515	3.172378
H	7.667333	16.805300	-3.803670
H	6.124069	16.591948	-3.142259
H	6.256172	18.738471	-3.847120
H	6.229762	18.814860	-2.070358
H	7.764855	19.015088	-2.945152
H	9.567029	16.426188	-4.507963
H	8.584176	15.669300	-5.565643
H	9.756014	17.441144	-6.744312
H	8.017292	17.769972	-6.642722
H	9.135529	18.567521	-5.527909

(Λ)-**D**

Geometry optimization in gas phase, B3LYP/6-311G**

```
SCF Done:  E(RB3LYP) =  -1476.13205541
Zero-point correction=                           0.537190 (Hartree/Particle)
Thermal correction to Energy=                    0.569754
Thermal correction to Enthalpy=                  0.570698
Thermal correction to Gibbs Free Energy=         0.470999
Sum of electronic and zero-point Energies=          -1475.594866
Sum of electronic and thermal Energies=             -1475.562302
Sum of electronic and thermal Enthalpies=           -1475.561357
Sum of electronic and thermal Free Energies=        -1475.661056
```

Energy in DMA:

SCF Done: E(RM06L) = -1476.1539203

Energy in NMP:

SCF Done: E(RM06L) = -1476.1536269

Cartesian coordinates:

C	-1.418648	-2.036902	0.683750
C	-0.078474	-1.602727	0.691531
C	0.894448	-2.486144	1.179203
C	0.567816	-3.767528	1.616742
C	-0.761129	-4.178382	1.574774
C	-1.756240	-3.324530	1.115302
C	0.285949	-0.228442	0.180845
C	0.136717	-0.001795	-1.233086
C	0.544078	-1.007929	-2.174065
C	0.055876	-0.979589	-3.513235
C	-0.685944	0.074802	-3.949602
C	-0.996981	1.181725	-3.109325
C	-0.573882	1.171855	-1.744759
C	-0.946983	2.284123	-0.957184
C	-1.664066	3.340305	-1.485637
C	-2.063110	3.345755	-2.831254
C	-1.735752	2.270139	-3.625016
N	1.461770	-1.933242	-1.824709
C	1.902243	-3.040333	-2.658898
O	2.199047	-2.056977	1.108015
O	-2.347652	-1.108502	0.357663
C	0.744469	0.737949	1.119972
C	1.888715	1.622647	0.849581
C	2.558729	2.226639	1.957102
C	2.072945	2.017192	3.281607
C	0.934693	1.316924	3.537071
C	0.195740	0.750271	2.459028
C	3.710091	3.017579	1.756825
C	4.212248	3.233780	0.492149
C	3.566185	2.641121	-0.600446
C	2.439666	1.855246	-0.427296
N	-1.053017	0.304820	2.668351
C	-1.693722	0.171417	3.968540
H	1.930363	-1.828154	-0.935380
H	-0.659323	2.330547	0.081628
H	-1.916079	4.179407	-0.847173
H	-2.620154	4.182195	-3.235192
H	-2.040280	2.241150	-4.665686
H	-1.030263	0.097032	-4.978221
H	0.317115	-1.776549	-4.195306
H	1.344786	-4.439942	1.964819
H	-1.022385	-5.174007	1.913421
H	-2.794136	-3.633045	1.122682
H	1.974382	1.432474	-1.302715
H	3.946138	2.802699	-1.602708
H	5.090474	3.849899	0.343823
H	4.194833	3.454673	2.622966
H	2.617097	2.469885	4.103805
H	0.560779	1.235498	4.548095
H	-1.612619	0.048816	1.861631
H	-3.261867	-1.499099	0.172522
H	-1.111894	-0.466939	4.638629
H	-1.842037	1.144695	4.446233
H	-2.668739	-0.288995	3.818467
H	2.777201	-2.698381	1.536226
H	2.588631	-3.651920	-2.075164
H	2.430481	-2.691803	-3.552242
H	1.063587	-3.670025	-2.967977
N	-4.851883	-1.928753	-0.267535
C	-5.857158	-1.707418	0.793902
H	-4.911244	-2.884399	-0.609845
H	-5.058158	-1.335648	-1.067795
H	-6.883274	-1.907728	0.470680
H	-5.796965	-0.671722	1.131540

H -5.630817 -2.353085 1.644096

Structure 6 (TS)

Geometry optimization in gas phase, B3LYP/6-311G**

SCF Done: E(RB3LYP) = -1476.06535863
Zero-point correction= 0.536110 (Hartree/Particle)
Thermal correction to Energy= 0.567825
Thermal correction to Enthalpy= 0.568770
Thermal correction to Gibbs Free Energy= 0.473037
Sum of electronic and zero-point Energies= -1475.529248
Sum of electronic and thermal Energies= -1475.497533
Sum of electronic and thermal Enthalpies= -1475.496589
Sum of electronic and thermal Free Energies= -1475.592321

Energy in DMA:

SCF Done: E(RM06L) = -1476.09709718

Energy in NMP:

SCF Done: E(RM06L) = -1476.09675482

Cartesian coordinates:

C	2.626599	1.966403	-2.230879
C	1.756689	1.091140	-1.569683
C	0.494872	1.549132	-1.135183
C	0.142452	2.886826	-1.375107
C	1.022583	3.769952	-2.008630
C	2.257153	3.295521	-2.433012
C	-0.469279	0.695473	-0.346647
C	-1.298856	-0.193264	-1.106126
C	-0.727846	-0.771807	-2.316494
O	2.071619	-0.190992	-1.306944
C	-0.583031	-2.212200	-2.314957
C	-1.360045	-2.986832	-1.521111
C	-2.448784	-2.393171	-0.787165
C	-2.467095	-0.981596	-0.650539
C	-3.684024	-0.376075	-0.309573
C	-4.799896	-1.135336	0.013993
C	-4.727014	-2.531362	0.023139
C	-3.560450	-3.154547	-0.389385
C	-0.265557	0.693418	1.081547
C	1.052699	1.033523	1.612575
C	1.642647	0.117185	2.554789
C	0.893740	-0.810852	3.200666
C	-0.532960	-0.785051	3.094042
C	-1.120116	0.042178	2.095745
C	-1.335516	-1.426201	4.054526
C	-2.695550	-1.181537	4.112403
C	-3.257959	-0.264095	3.220853
C	-2.483056	0.329142	2.233851
N	1.761205	2.113641	1.267141
C	3.125809	2.415201	1.683166
N	-0.319019	-0.123707	-3.405785
C	-0.731069	1.194113	-3.876583
O	-1.069894	3.386975	-0.998903
N	4.573072	-1.121160	-1.944712
C	4.640101	-2.445592	-1.287744
H	-3.767376	0.703343	-0.341276
H	-5.734667	-0.640374	0.250125
H	-5.592724	-3.121672	0.297838
H	-3.517137	-4.235145	-0.471199
H	-1.262527	-4.066476	-1.548050
H	0.121919	-2.653678	-3.011466
H	3.591186	1.607409	-2.568405
H	2.943719	3.969257	-2.932615
H	0.715353	4.795863	-2.164445
H	-2.931967	1.070526	1.590985
H	-4.305695	-0.000675	3.305327
H	-3.307462	-1.656470	4.869478
H	-0.862070	-2.072561	4.785479
H	1.361653	-1.501537	3.893738
H	2.703875	0.186846	2.747462

H	1.349740	2.750850	0.603589
H	-1.561051	2.712012	-0.514493
H	3.837644	1.684221	1.287942
H	3.209233	2.445069	2.771587
H	3.385577	3.395901	1.289630
H	5.329273	-0.532214	-1.605270
H	0.171894	-0.689115	-4.086056
H	-1.141707	1.083742	-4.883507
H	-1.503054	1.589116	-3.220102
H	0.107047	1.889789	-3.905501
H	3.001989	-0.426518	-1.597417
H	4.732122	-1.224946	-2.943654
H	3.840705	-3.077775	-1.676866
H	5.595194	-2.958899	-1.436342
H	4.472716	-2.321181	-0.216987

(Δ)-**D**

Geometry optimization in gas phase, B3LYP/6-311G**

SCF Done: E(RB3LYP) = -1476.13205785
Zero-point correction= 0.537192 (Hartree/Particle)
Thermal correction to Energy= 0.569756
Thermal correction to Enthalpy= 0.570700
Thermal correction to Gibbs Free Energy= 0.470998
Sum of electronic and zero-point Energies= -1475.594865
Sum of electronic and thermal Energies= -1475.562302
Sum of electronic and thermal Enthalpies= -1475.561358
Sum of electronic and thermal Free Energies= -1475.661059

Energy in DMA:

SCF Done: E(RM06L) = -1476.15392378

Energy in NMP:

SCF Done: E(RM06L) = -1476.15363039

Cartesian coordinates:

C	1.417783	-2.039385	0.678884
C	0.077748	-1.604770	0.686927
C	-0.895759	-2.489024	1.171880
C	-0.569872	-3.771481	1.606811
C	0.758983	-4.182622	1.564834
C	1.754637	-3.328090	1.107801
C	-0.286109	-0.229070	0.179659
C	-0.135978	0.001640	-1.233342
C	-0.541286	-1.002566	-2.177416
C	-0.051041	-0.970783	-3.515744
C	0.690314	0.085397	-3.948557
C	0.998784	1.190840	-3.105356
C	0.573734	1.177484	-1.741429
C	0.944246	2.288520	-0.950963
C	1.660839	3.346723	-1.476095
C	2.061873	3.355560	-2.821075
C	1.736968	2.281249	-3.617627
N	-1.458924	-1.929153	-1.831582
C	-1.897603	-3.034544	-2.668982
O	-2.200180	-2.059332	1.100412
O	2.347333	-1.110690	0.355239
C	-0.745080	0.734819	1.121384
C	-1.889966	1.619265	0.853099
C	-2.559475	2.221434	1.961912
C	-2.072746	2.010212	3.285764
C	-0.934287	1.309625	3.539437
C	-0.195931	0.744555	2.460132
C	-3.711292	3.012219	1.763497
C	-4.214406	3.229932	0.499478
C	-3.568882	2.638973	-0.594389
C	-2.441951	1.853334	-0.423088
N	1.052808	0.298302	2.668368
C	1.693890	0.162806	3.968129
H	-1.928708	-1.826444	-0.942591
H	0.654883	2.332472	0.087470
H	1.910826	4.184763	-0.835446

H	2.618482	4.193542	-3.222403
H	2.043022	2.254870	-4.657919
H	1.036189	0.110311	-4.976592
H	-0.310410	-1.766472	-4.200014
H	-1.347295	-4.444416	1.952848
H	1.019757	-5.179057	1.901470
H	2.792407	-3.637034	1.115082
H	-1.977167	1.431846	-1.299413
H	-3.949644	2.801711	-1.596158
H	-5.092994	3.845879	0.352588
H	-4.195609	3.447935	2.630571
H	-2.616400	2.461649	4.108980
H	-0.559783	1.226672	4.550114
H	1.612223	0.043692	1.861095
H	3.261682	-1.501173	0.170464
H	1.112124	-0.476336	4.637531
H	1.842713	1.135323	4.447240
H	2.668724	-0.297678	3.817060
H	-2.778900	-2.701397	1.526864
H	-2.584888	-3.647579	-2.087836
H	-2.424305	-2.684145	-3.562493
H	-1.058216	-3.663324	-2.977923
N	4.852430	-1.929827	-0.267779
C	5.855529	-1.707919	0.795600
H	4.913176	-2.885346	-0.610190
H	5.059745	-1.336366	-1.067500
H	6.882428	-1.907205	0.474239
H	5.793769	-0.672383	1.133455
H	5.628168	-2.354073	1.645151

Structure 8 (TS)

Geometry optimization in gas phase, B3LYP/6-311G**

SCF Done: E(RB3LYP) = -1476.06715377

Zero-point correction=	0.536784	(Hartree/Particle)
Thermal correction to Energy=	0.568019	
Thermal correction to Enthalpy=	0.568963	
Thermal correction to Gibbs Free Energy=	0.475009	
Sum of electronic and zero-point Energies=	-1475.530369	
Sum of electronic and thermal Energies=	-1475.499135	
Sum of electronic and thermal Enthalpies=	-1475.498191	
Sum of electronic and thermal Free Energies=	-1475.592145	

Energy in DMA:

SCF Done: E(RM06L) = -1476.10139404

Energy in NMP:

SCF Done: E(RM06L) = -1476.10102315

Cartesian coordinates:

C	-3.494575	1.115746	-1.530658
C	-2.192232	0.535770	-1.483439
C	-1.157957	1.193765	-0.647689
C	-1.381172	2.607713	-0.375234
C	-2.600980	3.188004	-0.625418
C	-3.662580	2.422330	-1.157357
C	-0.180550	0.409352	0.017171
C	0.057407	-0.966822	-0.387322
C	-0.119502	-1.331186	-1.748715
O	-1.952640	-0.495798	-2.173682
C	-0.280698	-2.683070	-2.154532
C	-0.203483	-3.686868	-1.246096
C	0.096285	-3.423065	0.115863
C	0.237816	-2.072620	0.571327
C	0.525951	-1.913780	1.947919
C	0.671179	-2.994574	2.795667
C	0.529077	-4.310657	2.333492
C	0.240573	-4.512774	1.005021
C	0.464444	0.979590	1.235901
C	-0.334729	1.400588	2.332952
C	0.295456	2.024666	3.452677
C	1.647055	2.198725	3.496556

C	2.493934	1.741463	2.453501
C	1.903522	1.093323	1.321620
C	3.896835	1.910750	2.529531
C	4.724825	1.444142	1.537013
C	4.159432	0.779083	0.432821
C	2.795792	0.611909	0.326182
N	-1.679932	1.190884	2.374092
C	-2.569229	1.635982	3.434696
N	0.433264	-0.551631	-2.922619
C	0.794404	0.897844	-2.922346
O	-0.404864	3.423885	0.079836
N	-1.311203	-0.805097	-5.049393
C	-1.146173	-1.797698	-6.136570
H	0.657531	-0.932323	2.364857
H	0.905471	-2.813316	3.838992
H	0.646033	-5.148832	3.009492
H	0.118301	-5.515848	0.612905
H	-0.336231	-4.715391	-1.562673
H	-0.459172	-2.894794	-3.201273
H	-4.273395	0.549692	-2.025886
H	-4.621352	2.903697	-1.314588
H	-2.727980	4.237968	-0.397893
H	2.405964	0.064052	-0.518938
H	4.807185	0.384191	-0.341904
H	5.797690	1.574580	1.605086
H	4.308618	2.415418	3.396785
H	2.096120	2.688103	4.354046
H	-0.313659	2.364403	4.278046
H	-2.110009	0.742512	1.583888
H	0.334083	2.901559	0.422879
H	-2.323130	1.172020	4.395190
H	-2.550579	2.723979	3.554692
H	-3.584287	1.343229	3.170171
H	-0.245207	-0.701959	-3.775299
H	1.286973	-1.064483	-3.156821
H	1.348949	1.077034	-3.844498
H	1.416825	1.149673	-2.069401
H	-0.108339	1.499105	-2.913362
H	-1.454648	0.118298	-5.451983
H	-2.149979	-0.998530	-4.504695
H	-1.997132	-1.822584	-6.822764
H	-1.026340	-2.792844	-5.705181
H	-0.246761	-1.564266	-6.707542

Structure 9

Geometry optimization in gas phase, B3LYP/6-311G**

```
SCF Done:  E(RB3LYP) =  -1476.11800668
Zero-point correction=                           0.538292 (Hartree/Particle)
Thermal correction to Energy=                    0.570331
Thermal correction to Enthalpy=                  0.571275
Thermal correction to Gibbs Free Energy=         0.473643
Sum of electronic and zero-point Energies=          -1475.579715
Sum of electronic and thermal Energies=             -1475.547676
Sum of electronic and thermal Enthalpies=           -1475.546732
Sum of electronic and thermal Free Energies=        -1475.644363
```

Energy in DMA:

SCF Done: E(RM06L) = -1476.14262274

Energy in NMP:

SCF Done: E(RM06L) = -1476.14226064

Cartesian coordinates:

C	-2.231654	2.483099	-2.629418
C	-1.604981	1.540562	-1.837491
C	-0.957153	1.835957	-0.618950
C	-1.026014	3.198776	-0.195446
C	-1.650995	4.160933	-0.998063
C	-2.241470	3.809533	-2.197458
C	-0.311556	0.742084	0.118682
C	-0.327125	-0.529803	-0.385423

C	-1.025279	-0.817173	-1.688405
O	-1.695384	0.240029	-2.297198
C	-1.972702	-1.981461	-1.584865
C	-1.709296	-2.980240	-0.733424
C	-0.512843	-2.968239	0.100083
C	0.211811	-1.754649	0.259675
C	1.395308	-1.790360	1.010598
C	1.828257	-2.967967	1.613746
C	1.087633	-4.142148	1.492103
C	-0.076855	-4.137206	0.735509
C	0.315114	1.007016	1.460171
C	-0.406192	0.693086	2.626498
C	0.215971	0.905465	3.895485
C	1.493681	1.379909	3.985115
C	2.255601	1.698675	2.830816
C	1.652741	1.515133	1.546305
C	3.580065	2.190247	2.921554
C	4.301274	2.498864	1.792582
C	3.709458	2.331141	0.523559
C	2.423730	1.853973	0.400915
N	-1.675651	0.188959	2.566315
C	-2.531976	-0.050451	3.715597
N	0.075512	-1.249659	-2.763496
C	1.132510	-0.241955	-3.048498
O	-0.521844	3.665236	0.958756
N	-1.219445	-1.822379	-5.217460
C	-1.420373	-3.260339	-5.513741
H	1.995529	-0.899645	1.114809
H	2.751260	-2.963899	2.181277
H	1.420733	-5.053343	1.974029
H	-0.656755	-5.046704	0.621720
H	-2.378794	-3.831028	-0.661666
H	-2.851966	-1.947403	-2.216103
H	-2.717298	2.173117	-3.545092
H	-2.726125	4.567227	-2.801226
H	-1.662031	5.180663	-0.636252
H	1.982545	1.759876	-0.584106
H	4.272350	2.590098	-0.366197
H	5.313302	2.876226	1.872737
H	4.017358	2.322001	3.905469
H	1.942129	1.525864	4.961893
H	-0.334384	0.679515	4.798544
H	-2.096515	0.107577	1.656776
H	-0.124310	2.942855	1.472080
H	-2.106508	-0.802713	4.386832
H	-2.722982	0.862849	4.289993
H	-3.488216	-0.429100	3.356167
H	-0.438907	-1.489108	-3.673997
H	0.499044	-2.108919	-2.408383
H	1.809823	-0.646480	-3.800580
H	1.684038	-0.019715	-2.137746
H	0.660023	0.660648	-3.430165
H	-0.711542	-1.386541	-5.983922
H	-2.120062	-1.349586	-5.187525
H	-1.962623	-3.436420	-6.447073
H	-1.977546	-3.721332	-4.696865
H	-0.449255	-3.753167	-5.581669

Structure 10 (TS)

Geometry optimization in gas phase, B3LYP/6-311G**

```
SCF Done:  E(RB3LYP) =  -1476.11405760
Zero-point correction=                           0.536174 (Hartree/Particle)
Thermal correction to Energy=                    0.568560
Thermal correction to Enthalpy=                  0.569504
Thermal correction to Gibbs Free Energy=         0.470299
Sum of electronic and zero-point Energies=         -1475.577884
Sum of electronic and thermal Energies=            -1475.545498
Sum of electronic and thermal Enthalpies=          -1475.544554
```

Sum of electronic and thermal Free Energies= -1475.643759

Energy in DMA:

SCF Done: E(RM06L) = -1476.13635465

Energy in NMP:

SCF Done: E(RM06L) = -1476.13603855

Cartesian coordinates:

C	-2.718879	2.164001	-2.366449
C	-1.983553	1.292090	-1.587383
C	-1.251127	1.667579	-0.436879
C	-1.365704	3.045729	-0.057317
C	-2.095520	3.935506	-0.851070
C	-2.756913	3.504493	-1.988988
C	-0.486531	0.645244	0.266279
C	-0.460741	-0.652515	-0.217847
C	-1.267534	-0.977692	-1.401759
O	-2.065645	-0.033694	-1.944227
C	-1.910217	-2.290824	-1.456512
C	-1.444466	-3.291308	-0.690686
C	-0.285885	-3.115164	0.160895
C	0.249665	-1.812157	0.372203
C	1.435247	-1.711666	1.118424
C	2.035456	-2.835504	1.674512
C	1.470749	-4.100610	1.512154
C	0.319483	-4.234025	0.752785
C	0.205952	0.992804	1.554540
C	-0.410153	0.645441	2.771628
C	0.273082	0.937467	3.992644
C	1.508079	1.520359	3.989379
C	2.164345	1.876418	2.782180
C	1.499467	1.614129	1.542694
C	3.444731	2.480353	2.778199
C	4.063644	2.823319	1.599634
C	3.409673	2.576989	0.375034
C	2.164450	1.990208	0.344363
N	-1.641824	0.054500	2.808234
C	-2.339438	-0.349090	4.017606
N	0.083416	-1.212858	-2.812479
C	1.012654	-0.093502	-3.029237
O	-0.809676	3.583123	1.036939
N	-1.374531	-1.785066	-5.330425
C	-0.503381	-1.758673	-6.525554
H	1.911919	-0.754435	1.251542
H	2.954560	-2.719435	2.236479
H	1.935095	-4.971062	1.959456
H	-0.119700	-5.212795	0.594069
H	-1.916997	-4.267243	-0.720674
H	-2.758387	-2.394034	-2.120875
H	-3.263194	1.788078	-3.222103
H	-3.322824	4.213542	-2.580913
H	-2.135262	4.968601	-0.531478
H	1.674786	1.833162	-0.608678
H	3.891099	2.860342	-0.554071
H	5.042656	3.286383	1.607461
H	3.930473	2.670894	3.729145
H	2.004989	1.724093	4.931699
H	-0.195691	0.684331	4.933592
H	-2.058216	-0.207430	1.931603
H	-0.345914	2.901994	1.552136
H	-1.785823	-1.106220	4.583758
H	-2.537693	0.503775	4.673747
H	-3.299360	-0.777116	3.730945
H	-0.466157	-1.424554	-3.675151
H	0.583866	-2.054725	-2.536785
H	1.772167	-0.347652	-3.773951
H	1.513619	0.156834	-2.093914
H	0.454850	0.774156	-3.381434
H	-2.128999	-1.112970	-5.444989
H	-1.827236	-2.693303	-5.263672

H	-1.031198	-1.976375	-7.459964
H	0.295717	-2.492454	-6.405640
H	-0.043110	-0.773074	-6.615150

(*aS*)-**A·H**+

Geometry optimization in gas phase, B3LYP/6-311G**

SCF Done: E(RB3LYP) = -1476.12287968
Zero-point correction= 0.537284 (Hartree/Particle)
Thermal correction to Energy= 0.569725
Thermal correction to Enthalpy= 0.570669
Thermal correction to Gibbs Free Energy= 0.472327
Sum of electronic and zero-point Energies= -1475.585595
Sum of electronic and thermal Energies= -1475.553155
Sum of electronic and thermal Enthalpies= -1475.552211
Sum of electronic and thermal Free Energies= -1475.650553

Energy in DMA:

SCF Done: E(RM06L) = -1476.14894206

Energy in NMP:

SCF Done: E(RM06L) = -1476.14865396

Cartesian coordinates:

C	-3.612024	-0.094466	-2.268357
C	-2.606840	-0.538926	-1.427704
C	-1.820042	0.304486	-0.587915
C	-2.103739	1.723015	-0.699506
C	-3.112217	2.162814	-1.549837
C	-3.857584	1.270395	-2.315268
C	-0.844634	-0.292533	0.263637
C	-0.647132	-1.702850	0.246512
C	-1.438917	-2.441967	-0.677420
O	-2.388642	-1.868892	-1.418760
C	-1.334222	-3.828753	-0.880865
C	-0.420113	-4.529461	-0.155662
C	0.401685	-3.898044	0.820568
C	0.300127	-2.488586	1.061745
C	1.119454	-1.971981	2.087529
C	1.988103	-2.777715	2.804966
C	2.094465	-4.147672	2.542624
C	1.301751	-4.695868	1.559321
C	-0.029652	0.601948	1.145371
C	-0.522455	0.978795	2.407949
C	0.299187	1.805244	3.233920
C	1.540460	2.205013	2.820612
C	2.070349	1.830976	1.558002
C	1.271827	1.012517	0.700748
C	3.355196	2.245424	1.129967
C	3.843936	1.865938	-0.097031
C	3.053982	1.060550	-0.945201
C	1.797065	0.643875	-0.566159
N	-1.758582	0.579265	2.842324
C	-2.315090	0.889094	4.151260
N	0.161964	-1.174736	-3.270039
C	1.226091	-2.149932	-3.555846
O	-1.453591	2.677441	-0.032040
N	1.143156	1.654362	-4.329087
C	0.131828	2.400161	-5.097253
H	1.087143	-0.929487	2.340663
H	2.594703	-2.328586	3.582440
H	2.782497	-4.765303	3.106503
H	1.351502	-5.756316	1.339915
H	-0.314656	-5.597960	-0.306622
H	-1.981301	-4.291512	-1.613743
H	-4.166254	-0.808581	-2.861538
H	-4.637657	1.651638	-2.963296
H	-3.296027	3.228270	-1.594583
H	1.206277	0.034757	-1.242219
H	3.444434	0.761104	-1.911345
H	4.829466	2.182897	-0.415832
H	3.948013	2.868612	1.790617

H	2.142326	2.827775	3.473596
H	-0.064710	2.109462	4.205544
H	-2.224859	-0.142375	2.319242
H	-0.763737	2.282992	0.532291
H	-1.720966	0.468912	4.970628
H	-2.403683	1.968626	4.297730
H	-3.316979	0.464865	4.204760
H	0.459221	-0.247596	-3.594348
H	-0.655566	-1.407114	-3.828177
H	1.542462	-2.187353	-4.607376
H	0.900437	-3.153823	-3.270542
H	2.105822	-1.914939	-2.951015
H	1.959977	1.488834	-4.910172
H	1.464678	2.214490	-3.544730
H	0.477893	3.366142	-5.486338
H	-0.738791	2.582730	-4.463390
H	-0.198008	1.793257	-5.943164

<u>Reaction on the *Si* face of (*aR*)-**A·H⁺** *via* intermediates **E**.</u>

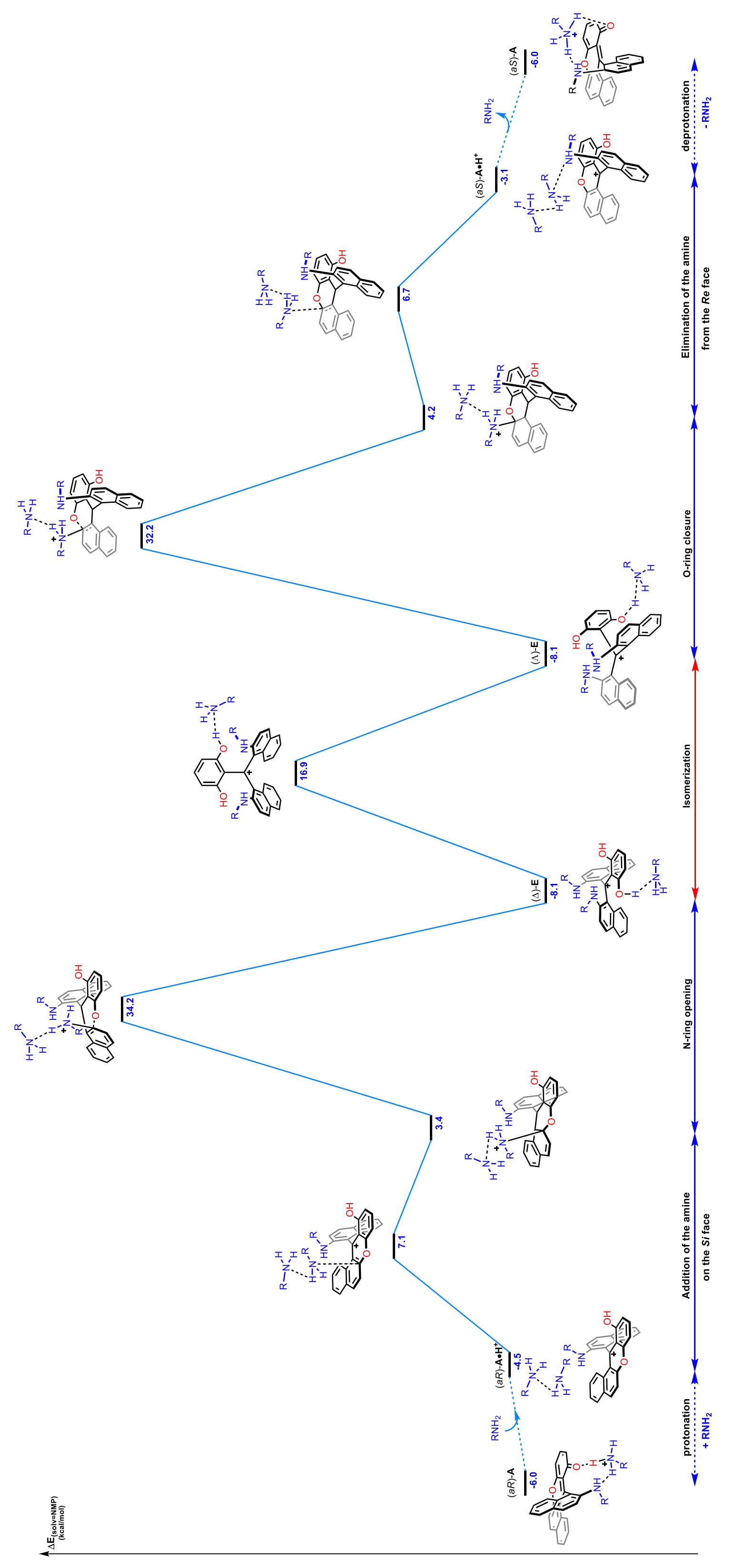

(*aR*)-**A·H**$^+$

Geometry optimization in gas phase, B3LYP/6-311G**

```
 SCF Done:  E(RB3LYP) =  -1476.13352559
 Zero-point correction=                           0.536124 (Hartree/Particle)
 Thermal correction to Energy=                    0.569553
 Thermal correction to Enthalpy=                  0.570498
 Thermal correction to Gibbs Free Energy=         0.466470
 Sum of electronic and zero-point Energies=          -1475.597401
 Sum of electronic and thermal Energies=             -1475.563972
 Sum of electronic and thermal Enthalpies=           -1475.563028
 Sum of electronic and thermal Free Energies=        -1475.667055
```

Energy in DMA:

```
 SCF Done:  E(RM06L) =  -1476.15420484
```

Energy in NMP:

```
 SCF Done:  E(RM06L) =  -1476.15393053
```

Cartesian coordinates:

```
 C      0.971573     1.458268    -3.919474
 C      0.489529     0.629819    -2.923515
 C      0.011077     1.076760    -1.655537
 C      0.006566     2.516480    -1.472197
 C      0.492298     3.345033    -2.479673
 C      0.972232     2.826467    -3.676838
 C     -0.438498     0.109706    -0.708160
 C     -0.423450    -1.279324    -1.043705
 C      0.005598    -1.613360    -2.355452
 O      0.458017    -0.690606    -3.214279
 C     -0.007539    -2.913123    -2.889405
 C     -0.444086    -3.937868    -2.104586
 C     -0.834519    -3.726907    -0.752927
 C     -0.807410    -2.411796    -0.181284
 C     -1.130841    -2.319530     1.188938
 C     -1.487069    -3.433158     1.930122
 C     -1.547855    -4.705905     1.350085
 C     -1.217026    -4.844369     0.021413
 C     -0.954279     0.588938     0.608160
 C     -0.042414     0.907774     1.640555
 C     -0.586289     1.341880     2.893965
 C     -1.934263     1.417383     3.098320
 C     -2.872141     1.086485     2.083633
 C     -2.376006     0.659643     0.813568
 C     -4.267423     1.185274     2.295881
 C     -5.158167     0.874459     1.295537
 C     -4.673731     0.459478     0.038832
 C     -3.321241     0.357667    -0.200849
 N      1.302996     0.814063     1.473328
 C      2.269083     1.222906     2.479603
 N      3.167327    -0.525380    -0.403094
 C      3.334693    -1.941883    -0.033322
 O     -0.443496     3.134609    -0.382858
 N      5.826560     0.881016     0.547055
 C      6.602881     1.207301    -0.664208
 H     -1.099019    -1.372239     1.693160
 H     -1.723222    -3.307697     2.980189
 H     -1.839277    -5.566299     1.939727
 H     -1.234348    -5.819328    -0.452140
 H     -0.481857    -4.945340    -2.503514
 H      0.316904    -3.054333    -3.911677
 H      1.311143     1.030617    -4.852708
 H      1.338894     3.503738    -4.438847
 H      0.473290     4.411572    -2.298156
 H     -2.981893     0.048823    -1.182080
 H     -5.375167     0.222205    -0.752761
 H     -6.224841     0.953393     1.465553
 H     -4.622690     1.516761     3.265593
 H     -2.308023     1.744007     4.062929
 H      0.089544     1.600837     3.696753
 H      1.720247     0.346183     0.658190
 H     -0.788727     2.480218     0.254714
```

H	2.215301	0.613646	3.389529
H	2.145343	2.274181	2.757741
H	3.262021	1.101875	2.047781
H	3.146612	-0.438942	-1.415673
H	3.996044	-0.000758	-0.096570
H	4.234568	-2.411760	-0.449962
H	3.385501	-2.025029	1.054494
H	2.468023	-2.520454	-0.362138
H	5.759474	1.704632	1.138501
H	6.327746	0.188500	1.096829
H	7.615835	1.576508	-0.464280
H	6.684214	0.316825	-1.291204
H	6.071206	1.969174	-1.238189

Structure 2 (TS)

Geometry optimization in gas phase, B3LYP/6-311G**

SCF Done: E(RB3LYP) = -1476.11380116
Zero-point correction=   0.536894 (Hartree/Particle)
Thermal correction to Energy=   0.568820
Thermal correction to Enthalpy=   0.569764
Thermal correction to Gibbs Free Energy=   0.471705
Sum of electronic and zero-point Energies=   -1475.576907
Sum of electronic and thermal Energies=   -1475.544981
Sum of electronic and thermal Enthalpies=   -1475.544037
Sum of electronic and thermal Free Energies=   -1475.642097

Energy in DMA:

SCF Done: E(RM06L) = -1476.13588826

Energy in NMP:

SCF Done: E(RM06L) = -1476.13556851

Cartesian coordinates:

C	1.719458	2.933079	-2.371786
C	1.141069	1.854451	-1.730163
C	0.378685	1.945336	-0.542793
C	0.164115	3.273860	-0.048932
C	0.759407	4.366334	-0.684556
C	1.529567	4.199315	-1.824352
C	-0.132872	0.714455	0.051213
C	0.164735	-0.504703	-0.525530
C	0.927010	-0.522467	-1.781069
O	1.276140	0.643731	-2.367020
C	0.564091	-1.521017	-2.787358
C	-0.113025	-2.620585	-2.416892
C	-0.447006	-2.870414	-1.029201
C	-0.266490	-1.843055	-0.060287
C	-0.492367	-2.170717	1.285411
C	-0.932270	-3.436348	1.655216
C	-1.168042	-4.418074	0.692947
C	-0.918019	-4.133062	-0.640154
C	-1.020330	0.788230	1.261272
C	-0.454085	0.936553	2.536832
C	-1.309206	0.924648	3.675359
C	-2.662636	0.771106	3.536157
C	-3.271881	0.635320	2.264472
C	-2.438373	0.644930	1.101658
C	-4.675150	0.498456	2.121142
C	-5.248146	0.379391	0.879084
C	-4.430505	0.396790	-0.271555
C	-3.065277	0.525993	-0.167626
N	0.920688	1.032676	2.695079
C	1.531613	1.457282	3.951582
N	2.665912	-1.224956	-1.213658
C	3.344826	-0.495980	-0.131257
O	-0.580400	3.575312	1.028466
N	4.404170	-1.328247	-3.609048
C	5.827012	-1.582443	-3.295090
H	-0.303637	-1.442042	2.056459
H	-1.090147	-3.656347	2.704349
H	-1.526914	-5.397611	0.984549

```
H    -1.071147    -4.892573    -1.398892
H    -0.395753    -3.362512    -3.155865
H     0.851902    -1.313949    -3.809883
H     2.273368     2.776004    -3.287401
H     1.970453     5.064268    -2.304753
H     0.577744     5.346877    -0.264268
H    -2.462145     0.540556    -1.066554
H    -4.886326     0.310369    -1.251255
H    -6.321772     0.277941     0.777332
H    -5.290107     0.493240     3.014513
H    -3.292405     0.765916     4.419209
H    -0.883806     1.037986     4.662879
H     1.411826     1.358361     1.876604
H    -1.038105     2.784030     1.359147
H     1.363814     0.714643     4.734104
H     1.161115     2.428454     4.300883
H     2.607940     1.533161     3.798329
H     2.459344    -2.181640    -0.935473
H     3.247400    -1.252450    -2.081506
H     4.249409    -1.018792     0.191494
H     3.623958     0.495621    -0.487843
H     2.676143    -0.398755     0.725591
H     4.061851    -2.054421    -4.233407
H     4.320941    -0.459526    -4.131072
H     6.469561    -1.640240    -4.179879
H     6.202809    -0.784907    -2.651675
H     5.913282    -2.523269    -2.748444
```

Structure 3

Geometry optimization in gas phase, B3LYP/6-311G**

```
SCF Done:  E(RB3LYP) =  -1476.11738597
Zero-point correction=                           0.538849 (Hartree/Particle)
Thermal correction to Energy=                    0.570532
Thermal correction to Enthalpy=                  0.571477
Thermal correction to Gibbs Free Energy=         0.474299
Sum of electronic and zero-point Energies=          -1475.578537
Sum of electronic and thermal Energies=             -1475.546854
Sum of electronic and thermal Enthalpies=           -1475.545909
Sum of electronic and thermal Free Energies=        -1475.643087
```

Energy in DMA:

```
SCF Done:  E(RM06L) =  -1476.14181527
```

Energy in NMP:

```
SCF Done:  E(RM06L) =  -1476.14144381
```

Cartesian coordinates:

```
C     1.909487     2.878310    -2.274562
C     1.353349     1.782248    -1.643146
C     0.520015     1.865791    -0.507311
C     0.218180     3.186617    -0.055086
C     0.786669     4.300361    -0.683377
C     1.622570     4.148133    -1.774553
C     0.015219     0.619614     0.087128
C     0.376448    -0.586481    -0.440147
C     1.250455    -0.646520    -1.662814
O     1.628630     0.565175    -2.234948
C     0.665404    -1.522836    -2.737015
C    -0.085862    -2.577378    -2.397557
C    -0.342973    -2.903823    -0.999057
C    -0.080508    -1.928101     0.000983
C    -0.257940    -2.291861     1.342355
C    -0.725079    -3.557482     1.685465
C    -1.032788    -4.491174     0.697785
C    -0.836552    -4.162763    -0.637544
C    -0.922448     0.670304     1.261390
C    -0.405160     0.834439     2.554292
C    -1.294633     0.796842     3.664494
C    -2.638323     0.605251     3.481164
C    -3.200457     0.453448     2.190034
C    -2.328860     0.487535     1.055772
```

C	-4.593796	0.277360	1.998713
C	-5.120348	0.144374	0.737719
C	-4.264857	0.185655	-0.384907
C	-2.908168	0.351834	-0.234484
N	0.964676	0.971174	2.753505
C	1.514680	1.423042	4.029322
N	2.635387	-1.330127	-1.247594
C	3.414822	-0.616055	-0.200669
O	-0.594229	3.470701	0.980299
N	4.228331	-1.555883	-3.582318
C	5.669210	-1.838756	-3.381635
H	-0.013425	-1.590100	2.125190
H	-0.848576	-3.812920	2.731173
H	-1.411627	-5.469459	0.967759
H	-1.058548	-4.885997	-1.414665
H	-0.504376	-3.222144	-3.163096
H	0.874894	-1.238524	-3.760657
H	2.527095	2.732352	-3.150744
H	2.045249	5.022320	-2.254760
H	0.531863	5.277525	-0.294596
H	-2.272979	0.384107	-1.110568
H	-4.685130	0.088565	-1.379459
H	-6.186779	0.013745	0.599433
H	-5.238777	0.253700	2.870408
H	-3.296625	0.581831	4.342868
H	-0.905482	0.922437	4.665399
H	1.454790	1.346223	1.954886
H	-1.026101	2.663818	1.305901
H	1.345975	0.677336	4.808687
H	1.097060	2.381005	4.361998
H	2.592558	1.537610	3.914581
H	2.399742	-2.270984	-0.926068
H	3.225409	-1.427798	-2.137901
H	4.315589	-1.188264	0.020295
H	3.690310	0.365363	-0.581136
H	2.811942	-0.515306	0.700298
H	3.824025	-2.270339	-4.183618
H	4.119724	-0.680671	-4.090092
H	6.229949	-1.903153	-4.318506
H	6.110550	-1.049606	-2.770989
H	5.780643	-2.784410	-2.848794

Structure 4 (TS)

Geometry optimization in gas phase, B3LYP/6-311G**

SCF Done: E(RB3LYP) = -1476.06057157

Zero-point correction= 0.536949 (Hartree/Particle)
Thermal correction to Energy= 0.567967
Thermal correction to Enthalpy= 0.568912
Thermal correction to Gibbs Free Energy= 0.475538
Sum of electronic and zero-point Energies= -1475.523622
Sum of electronic and thermal Energies= -1475.492604
Sum of electronic and thermal Enthalpies= -1475.491660
Sum of electronic and thermal Free Energies= -1475.585033

Energy in DMA:

SCF Done: E(RM06L) = -1476.09269826

Energy in NMP:

SCF Done: E(RM06L) = -1476.09231907

Cartesian coordinates:

C	9.107168	20.006183	-2.896661
C	9.441753	19.024665	-1.917175
C	9.062635	19.259416	-0.507227
C	8.775766	20.649328	-0.161026
C	8.645523	21.607734	-1.134261
C	8.771254	21.272731	-2.499636
C	8.713556	18.163828	0.326127
C	8.948394	16.779398	-0.032607
C	10.014016	16.412621	-0.915941
O	10.068039	17.981319	-2.273147

C	9.936097	15.260640	-1.744156
C	8.855132	14.439956	-1.687452
C	7.877816	14.598364	-0.668470
C	7.963972	15.702382	0.239858
C	7.091881	15.653565	1.350840
C	6.148918	14.651840	1.497779
C	6.008741	13.635355	0.544347
C	6.880851	13.608211	-0.518472
C	7.806955	18.463547	1.492820
C	8.316203	18.455588	2.798242
C	7.422170	18.580451	3.895800
C	6.079564	18.760135	3.688232
C	5.535398	18.852756	2.384535
C	6.408876	18.704213	1.260135
C	4.150399	19.075372	2.176063
C	3.632168	19.140803	0.907330
C	4.486168	18.975970	-0.204954
C	5.833521	18.760357	-0.037696
N	9.699619	18.330435	3.002690
C	10.258360	18.349711	4.353838
N	11.457081	16.745547	-0.659794
C	11.864463	17.855743	0.243182
O	8.702219	21.084400	1.121949
N	12.735520	17.127884	-3.082071
C	13.845700	18.107345	-3.118178
H	7.171673	16.378297	2.138826
H	5.516789	14.656198	2.378024
H	5.252879	12.868673	0.661565
H	6.838333	12.806628	-1.247475
H	8.773427	13.599029	-2.366830
H	10.744457	15.067267	-2.438996
H	9.274223	19.749213	-3.934504
H	8.625134	22.047998	-3.243569
H	8.422855	22.620045	-0.824709
H	6.459965	18.634378	-0.910364
H	4.073184	19.020257	-1.206082
H	2.573802	19.314304	0.754870
H	3.507777	19.193418	3.041661
H	5.415266	18.860358	4.539700
H	7.806314	18.553948	4.905875
H	10.218043	18.929897	2.375425
H	8.332510	20.387727	1.685560
H	9.891383	17.494881	4.924138
H	10.033732	19.267937	4.910999
H	11.341908	18.252988	4.277347
H	11.838480	15.884210	-0.260422
H	11.967807	16.876695	-1.624921
H	12.948321	17.803786	0.348042
H	11.580564	18.804800	-0.199133
H	11.388656	17.722904	1.211433
H	13.009530	16.285123	-3.581432
H	11.921538	17.503138	-3.566173
H	14.163811	18.353244	-4.135150
H	13.528025	19.028568	-2.627713
H	14.703555	17.706822	-2.575742

(Δ)-E

Geometry optimization in gas phase, B3LYP/6-311G**

SCF Done: E(RB3LYP) = -1476.13724323

Zero-point correction=	0.536897	(Hartree/Particle)
Thermal correction to Energy=	0.569587	
Thermal correction to Enthalpy=	0.570531	
Thermal correction to Gibbs Free Energy=	0.471618	
Sum of electronic and zero-point Energies=	-1475.600346	
Sum of electronic and thermal Energies=	-1475.567656	
Sum of electronic and thermal Enthalpies=	-1475.566712	
Sum of electronic and thermal Free Energies=	-1475.665626	

Energy in DMA:

SCF Done: E(RM06L) = -1476.15998666

Energy in NMP:

SCF Done: E(RM06L) = -1476.15968748

Cartesian coordinates:

C	-1.880902	1.555181	1.032867
C	-1.375610	0.559449	0.175685
C	-2.229477	0.047230	-0.824647
C	-3.533331	0.532034	-0.963665
C	-3.994076	1.526426	-0.109387
C	-3.178513	2.043086	0.891183
C	0.016690	0.040697	0.355595
C	0.949432	0.159094	-0.679250
C	2.013585	-0.834384	-0.853088
C	2.432949	-1.148964	-2.186411
C	2.039131	-0.395376	-3.246603
C	1.253735	0.788928	-3.084862
C	0.756764	1.104217	-1.794252
C	0.156774	2.361449	-1.615515
C	-0.014173	3.231891	-2.682534
C	0.409308	2.880625	-3.969831
C	1.050482	1.671554	-4.163379
N	2.542589	-1.513263	0.163293
C	3.470028	-2.636032	0.056648
O	-1.753843	-0.974913	-1.574889
O	-1.009921	2.079637	1.940466
C	0.259413	-0.635522	1.637631
C	1.230018	-0.126157	2.584103
C	1.328308	-0.723331	3.880223
C	0.458495	-1.799710	4.208133
C	-0.440212	-2.298107	3.313016
C	-0.543862	-1.753588	1.995952
C	2.258343	-0.226015	4.821662
C	3.083917	0.831094	4.513536
C	2.983221	1.435581	3.246970
C	2.076620	0.980455	2.312411
N	-1.345608	-2.360088	1.084526
C	-2.289326	-3.424035	1.383774
H	2.280741	-1.232897	1.097822
H	-0.147346	2.674759	-0.627291
H	-0.469290	4.200163	-2.510188
H	0.266459	3.564461	-4.797675
H	1.433199	1.402582	-5.141953
H	2.387272	-0.654285	-4.241165
H	3.098842	-1.985881	-2.341465
H	-4.183469	0.111332	-1.719980
H	-5.004808	1.901290	-0.220191
H	-3.537186	2.828278	1.548159
H	1.999821	1.492074	1.362668
H	3.616204	2.281730	3.004931
H	3.795269	1.204066	5.240202
H	2.305700	-0.693296	5.799441
H	0.525969	-2.236529	5.198822
H	-1.069230	-3.131826	3.592958
H	-1.327813	-2.036285	0.125843
H	-1.463518	2.727006	2.491398
H	-1.786502	-4.346820	1.692515
H	-2.997132	-3.133263	2.165983
H	-2.855829	-3.636094	0.477835
H	-2.373005	-1.223984	-2.330846
H	4.407236	-2.331300	-0.415267
H	3.033987	-3.461652	-0.510938
H	3.689186	-2.985029	1.063808
N	-3.215343	-1.716124	-3.748408
C	-3.073449	-0.705601	-4.819454
H	-4.199004	-1.918013	-3.588588
H	-2.798503	-2.594622	-4.046473
H	-3.527644	-1.009062	-5.767733
H	-2.013133	-0.510511	-4.984936

H	-3.535760	0.227690	-4.494739

Structure 5 (TS)

Geometry optimization in gas phase, B3LYP/6-311G**

```
SCF Done:  E(RB3LYP) =  -1476.09017020
Zero-point correction=                           0.535529 (Hartree/Particle)
Thermal correction to Energy=                    0.566817
Thermal correction to Enthalpy=                  0.567761
Thermal correction to Gibbs Free Energy=         0.473154
Sum of electronic and zero-point Energies=          -1475.554642
Sum of electronic and thermal Energies=             -1475.523353
Sum of electronic and thermal Enthalpies=           -1475.522409
Sum of electronic and thermal Free Energies=        -1475.617017
```

Energy in DMA:

SCF Done: E(RM06L) = -1476.12029412

Energy in NMP:

SCF Done: E(RM06L) = -1476.11993587

Cartesian coordinates:

C	13.068147	17.859089	-2.409217
C	12.198074	17.355571	-3.470373
C	12.779041	17.365495	-4.821899
C	13.942846	18.091552	-5.077872
C	14.638321	18.683246	-4.033323
C	14.226154	18.558901	-2.705509
C	10.827283	17.180410	-3.279505
C	9.859734	16.752378	-4.361826
C	9.743860	15.374644	-4.609927
C	8.559849	14.869849	-5.202365
C	7.570895	15.709890	-5.642269
C	7.760643	17.111536	-5.622734
C	8.936512	17.644495	-5.003396
C	9.189666	19.030252	-5.170188
C	8.298155	19.852061	-5.825299
C	7.096154	19.333703	-6.346864
C	6.840075	17.988254	-6.254550
N	10.800442	14.508194	-4.238144
C	10.650624	13.063135	-4.420084
O	12.160444	16.683673	-5.751535
O	12.690636	17.626861	-1.152082
C	10.169922	17.329259	-1.928142
C	9.544192	18.552118	-1.524714
C	8.746186	18.581739	-0.335089
C	8.671600	17.414851	0.464485
C	9.376888	16.295174	0.141889
C	10.143415	16.214192	-1.063206
C	8.121574	19.785354	0.066721
C	8.315653	20.957418	-0.633110
C	9.184543	20.957939	-1.738061
C	9.788838	19.794814	-2.162202
N	10.797003	15.049953	-1.359972
C	10.822614	13.891757	-0.484277
H	11.660821	14.837763	-4.665679
H	10.122799	19.441528	-4.820832
H	8.527664	20.904017	-5.945793
H	6.391302	19.994052	-6.840019
H	5.942689	17.567392	-6.689101
H	6.662426	15.303255	-6.071361
H	8.432744	13.801605	-5.306712
H	14.333056	18.131831	-6.087307
H	15.556968	19.221819	-4.244389
H	14.829684	18.965947	-1.902841
H	10.503414	19.843720	-2.968516
H	9.399538	21.890928	-2.251517
H	7.835632	21.874490	-0.314452
H	7.505630	19.775778	0.957830
H	8.064927	17.431555	1.362519
H	9.328987	15.429192	0.789721
H	10.997933	14.860987	-2.340402

H	13.259258	18.105749	-0.533466
H	9.830635	13.437102	-0.342793
H	11.227415	14.141115	0.498726
H	11.477281	13.141647	-0.932300
H	12.440527	16.889907	-6.748128
H	11.573734	12.581062	-4.100752
H	9.839256	12.687589	-3.792612
H	10.452585	12.767065	-5.459535
N	12.578034	17.128477	-8.273433
C	11.995124	18.429882	-8.683299
H	13.531595	17.048026	-8.615017
H	12.063053	16.368480	-8.711736
H	11.986921	18.563180	-9.767450
H	10.970831	18.492924	-8.311745
H	12.571630	19.237980	-8.233632

(Λ)-E

Geometry optimization in gas phase, B3LYP/6-311G**

SCF Done: E(RB3LYP) = -1476.13724293

Zero-point correction= 0.536895 (Hartree/Particle)

Thermal correction to Energy= 0.569586

Thermal correction to Enthalpy= 0.570530

Thermal correction to Gibbs Free Energy= 0.471611

Sum of electronic and zero-point Energies= -1475.600348

Sum of electronic and thermal Energies= -1475.567657

Sum of electronic and thermal Enthalpies= -1475.566712

Sum of electronic and thermal Free Energies= -1475.665632

Energy in DMA:

SCF Done: E(RM06L) = -1476.15998476

Energy in NMP:

SCF Done: E(RM06L) = -1476.15968558

Cartesian coordinates:

C	1.880059	1.556673	1.033404
C	1.375645	0.560960	0.175691
C	2.229681	0.050669	-0.825483
C	3.532836	0.537300	-0.964744
C	3.992686	1.531630	-0.109926
C	3.176933	2.046426	0.891460
C	-0.015874	0.040238	0.355842
C	-0.949343	0.158419	-0.678387
C	-2.012102	-0.836414	-0.852866
C	-2.432008	-1.149695	-2.186327
C	-2.040066	-0.394060	-3.245755
C	-1.256255	0.791145	-3.082935
C	-0.758768	1.105309	-1.792255
C	-0.160518	2.363182	-1.612155
C	0.008323	3.235403	-2.678054
C	-0.415606	2.885351	-3.965538
C	-1.055139	1.675616	-4.160341
N	-2.539257	-1.517675	0.162898
C	-3.465042	-2.641702	0.055253
O	1.755092	-0.971643	-1.576167
O	1.008778	2.079273	1.941800
C	-0.257135	-0.637342	1.637426
C	-1.228246	-0.130115	2.584572
C	-1.325120	-0.728270	3.880336
C	-0.453509	-1.803514	4.207230
C	0.445633	-2.299959	3.311475
C	0.548004	-1.754401	1.994726
C	-2.255557	-0.232987	4.822449
C	-3.082868	0.823050	4.515317
C	-2.983614	1.428482	3.249087
C	-2.076690	0.975330	2.313890
N	1.350349	-2.359063	1.082625
C	2.295500	-3.422051	1.380742
H	-2.276755	-1.238590	1.097626
H	0.143824	2.675541	-0.623697
H	0.462105	4.204116	-2.504664

```
 H    -0.274394     3.570589    -4.792502
 H    -1.438211     1.407486    -5.139007
 H    -2.388547    -0.652099    -4.240423
 H    -3.096767    -1.987384    -2.342069
 H     4.183155     0.117983    -1.721675
 H     5.002869     1.907919    -0.220912
 H     3.534868     2.831609     1.548848
 H    -2.001066     1.487629     1.364421
 H    -3.618009     2.273792     3.007822
 H    -3.794493     1.194470     5.242511
 H    -2.301834    -0.700965     5.799946
 H    -0.519962    -2.241075     5.197661
 H     1.076058    -3.132869     3.590646
 H     1.331626    -2.034770     0.124128
 H     1.461849     2.726517     2.493313
 H     1.793937    -4.345599     1.689246
 H     3.003418    -3.130890     2.162707
 H     2.861730    -3.632951     0.474362
 H     2.374009    -1.219051    -2.332854
 H    -4.402975    -2.337745    -0.415717
 H    -3.028105    -3.465916    -0.513698
 H    -3.683124    -2.992389     1.062059
 N     3.215747    -1.708670    -3.751722
 C     3.071703    -0.697039    -4.821440
 H     4.199793    -1.909473    -3.592909
 H     2.799828    -2.587346    -4.050541
 H     3.525645    -0.998716    -5.770411
 H     2.011002    -0.503225    -4.985941
 H     3.532954     0.236497    -4.495923
```

Structure 6 (TS)

Geometry optimization in gas phase, B3LYP/6-311G**

```
 SCF Done:  E(RB3LYP) =  -1476.06307949
 Zero-point correction=                           0.537177 (Hartree/Particle)
 Thermal correction to Energy=                    0.568184
 Thermal correction to Enthalpy=                  0.569129
 Thermal correction to Gibbs Free Energy=         0.475996
 Sum of electronic and zero-point Energies=          -1475.525902
 Sum of electronic and thermal Energies=             -1475.494895
 Sum of electronic and thermal Enthalpies=           -1475.493951
 Sum of electronic and thermal Free Energies=        -1475.587083
```

Energy in DMA:

```
 SCF Done:  E(RM06L) =  -1476.09538956
```

Energy in NMP:

```
 SCF Done:  E(RM06L) =  -1476.09501612
```

Cartesian coordinates:

```
 C     3.129380    -4.364858    -2.281425
 C     3.478710    -3.364674    -3.265770
 C     3.861447    -3.879910    -4.586885
 C     3.417671    -5.186027    -4.955919
 C     2.960739    -6.049779    -3.995477
 C     2.863008    -5.662682    -2.639616
 C     3.136051    -1.991698    -3.109014
 C     3.491592    -0.995595    -4.087011
 C     4.629078    -1.195574    -4.936870
 O     4.556948    -3.193333    -5.398035
 C     4.716957    -0.590933    -6.222116
 C     3.730777     0.228440    -6.667431
 C     2.685666     0.652338    -5.801115
 C     2.602624     0.128468    -4.471544
 C     1.669691     0.757141    -3.617611
 C     0.827086     1.758886    -4.067700
 C     0.856488     2.195054    -5.397951
 C     1.791490     1.650240    -6.247505
 C     2.115862    -1.663991    -2.054117
 C     0.734304    -1.951625    -2.291027
 C    -0.236582    -1.500780    -1.338267
 C     0.204966    -0.797463    -0.192373
```

C	1.540133	-0.592962	0.047745
C	2.517530	-1.057649	-0.867360
C	-1.614547	-1.750943	-1.568426
C	-2.033014	-2.412639	-2.694793
C	-1.081646	-2.844158	-3.646217
C	0.259828	-2.617130	-3.455490
N	3.902690	-0.957380	-0.600071
C	4.359536	-0.191575	0.558976
N	6.014240	-1.437622	-4.400796
C	6.257890	-1.954291	-3.023231
O	3.141718	-3.962641	-0.983376
N	7.377927	-3.064039	-6.185385
C	8.347042	-4.035155	-5.628545
H	1.620549	0.492817	-2.578088
H	0.139345	2.215409	-3.365506
H	0.178561	2.966840	-5.741050
H	1.876267	1.997564	-7.271248
H	3.776763	0.637393	-7.670618
H	5.572529	-0.821351	-6.845561
H	3.577143	-5.496641	-5.980190
H	2.717409	-7.071306	-4.264961
H	2.595585	-6.392039	-1.883142
H	0.962496	-2.954104	-4.206055
H	-1.415507	-3.359662	-4.539439
H	-3.086943	-2.601192	-2.860853
H	-2.333405	-1.405728	-0.833057
H	-0.532223	-0.438947	0.517989
H	1.848155	-0.090842	0.954837
H	4.300071	-1.890394	-0.600279
H	3.998504	-0.577602	1.521442
H	4.045401	0.849376	0.464226
H	5.450567	-0.207771	0.576627
H	2.753055	-4.644809	-0.423031
H	6.452354	-0.514665	-4.449570
H	6.562977	-2.064509	-5.113292
H	7.324077	-1.840969	-2.825203
H	5.673750	-1.378880	-2.307211
H	5.983049	-3.002945	-2.978784
H	8.690218	-4.766196	-6.366096
H	9.216417	-3.501691	-5.241101
H	7.880181	-4.575302	-4.803657
H	6.551634	-3.551534	-6.527647
H	7.791234	-2.592480	-6.986277

Structure 7

Geometry optimization in gas phase, B3LYP/6-311G**

```
 SCF Done:  E(RB3LYP) =  -1476.11203919
 Zero-point correction=                           0.538671 (Hartree/Particle)
 Thermal correction to Energy=                    0.570456
 Thermal correction to Enthalpy=                  0.571400
 Thermal correction to Gibbs Free Energy=         0.474388
 Sum of electronic and zero-point Energies=          -1475.573369
 Sum of electronic and thermal Energies=             -1475.541583
 Sum of electronic and thermal Enthalpies=           -1475.540639
 Sum of electronic and thermal Free Energies=        -1475.637652
```

Energy in DMA:

SCF Done: E(RM06L) = -1476.14003211

Energy in NMP:

SCF Done: E(RM06L) = -1476.13964723

Cartesian coordinates:

C	-0.148953	-2.836678	1.327707
C	0.350633	-1.869796	0.409557
C	1.237132	-2.362154	-0.566647
C	1.629000	-3.689058	-0.660454
C	1.118621	-4.598514	0.257241
C	0.239414	-4.175284	1.243356
C	-0.025495	-0.444552	0.374368
C	0.550179	0.380252	-0.547430

C	1.522298	-0.163214	-1.558001
O	1.746991	-1.540320	-1.546843
C	1.174505	0.244314	-2.964281
C	0.551719	1.407815	-3.187694
C	0.218647	2.309216	-2.091045
C	0.256481	1.820650	-0.757378
C	0.026930	2.726352	0.286385
C	-0.285341	4.057111	0.021991
C	-0.380286	4.516332	-1.290282
C	-0.122582	3.643618	-2.339837
C	-1.089686	0.084831	1.288724
C	-2.431277	0.167697	0.815691
C	-3.441215	0.708462	1.674773
C	-3.068804	1.137117	2.970028
C	-1.777238	1.025377	3.416720
C	-0.765144	0.481435	2.584326
C	-4.779160	0.799038	1.212582
C	-5.123116	0.370550	-0.045099
C	-4.131436	-0.168796	-0.895111
C	-2.825006	-0.266847	-0.480525
N	0.562865	0.400279	3.028266
C	0.895730	0.608379	4.434323
N	2.954446	0.474964	-1.254503
C	3.525212	0.168481	0.085794
O	-0.996372	-2.434830	2.302797
N	4.808906	-0.315459	-3.258059
C	5.041178	-1.777416	-3.331464
H	0.111864	2.394452	1.309629
H	-0.456906	4.736506	0.848446
H	-0.641649	5.548078	-1.492097
H	-0.175948	3.992948	-3.365221
H	0.301558	1.710529	-4.199040
H	1.434190	-0.457128	-3.747272
H	2.300563	-3.989814	-1.453311
H	1.405063	-5.641552	0.202101
H	-0.159086	-4.884616	1.961185
H	-2.084551	-0.685471	-1.150714
H	-4.406872	-0.511463	-1.886237
H	-6.148459	0.441343	-0.387955
H	-5.530002	1.213537	1.876996
H	-3.828749	1.552348	3.623306
H	-1.532293	1.349692	4.419043
H	1.049435	-0.393753	2.637105
H	0.341375	-0.049061	5.116870
H	0.706823	1.644187	4.723418
H	1.962322	0.422710	4.566008
H	-1.307425	-3.202174	2.796822
H	2.830122	1.484793	-1.352869
H	3.633077	0.161955	-2.022002
H	4.475908	0.690855	0.190899
H	2.834828	0.497465	0.860880
H	3.689131	-0.904520	0.157945
H	5.702783	-2.067232	-4.152804
H	5.484979	-2.117540	-2.394621
H	4.084309	-2.285483	-3.455602
H	4.447093	0.015179	-4.149886
H	5.697879	0.163804	-3.133658

Structure 8 (TS)

Geometry optimization in gas phase, B3LYP/6-311G**

```
SCF Done:  E(RB3LYP) =  -1476.11380116
Zero-point correction=                           0.536894 (Hartree/Particle)
Thermal correction to Energy=                    0.568819
Thermal correction to Enthalpy=                  0.569764
Thermal correction to Gibbs Free Energy=         0.471703
Sum of electronic and zero-point Energies=          -1475.576907
Sum of electronic and thermal Energies=             -1475.544982
Sum of electronic and thermal Enthalpies=           -1475.544038
```

Sum of electronic and thermal Free Energies= -1475.642098

Energy in DMA:

SCF Done: E(RM06L) = -1476.13597932

Energy in NMP:

SCF Done: E(RM06L) = -1476.13566435

Cartesian coordinates:

C	-1.719458	2.932550	-2.371786
C	-1.138952	1.854451	-1.730692
C	-0.377097	1.945336	-0.542793
C	-0.163586	3.274918	-0.048403
C	-0.759936	4.366334	-0.684027
C	-1.530096	4.198786	-1.823823
C	0.133930	0.716043	0.051213
C	-0.159972	-0.503645	-0.529234
C	-0.907430	-0.517175	-1.788477
O	-1.269261	0.644789	-2.368078
C	-0.560916	-1.520488	-2.789475
C	0.111967	-2.623231	-2.416892
C	0.447535	-2.870414	-1.030259
C	0.268607	-1.841997	-0.061875
C	0.492896	-2.169659	1.284353
C	0.931741	-3.435290	1.654687
C	1.167513	-4.417545	0.692947
C	0.918019	-4.133591	-0.640154
C	1.020330	0.789288	1.261801
C	0.453556	0.937082	2.537361
C	1.309206	0.924648	3.675888
C	2.662636	0.771106	3.536157
C	3.271881	0.635320	2.264472
C	2.438373	0.644930	1.101658
C	4.675150	0.498456	2.121142
C	5.248146	0.379391	0.879084
C	4.430505	0.396790	-0.271555
C	3.065277	0.525993	-0.167626
N	-0.920159	1.032676	2.696137
C	-1.532142	1.457282	3.952111
N	-2.682846	-1.233423	-1.205191
C	-3.353822	-0.498097	-0.125436
O	0.580400	3.575841	1.028995
N	-4.407345	-1.328776	-3.613281
C	-5.829129	-1.582972	-3.296148
H	0.304166	-1.440454	2.054871
H	1.088559	-3.654759	2.703820
H	1.525856	-5.397082	0.985078
H	1.070089	-4.893631	-1.398363
H	0.386757	-3.369391	-3.154277
H	-0.853490	-1.317653	-3.811471
H	-2.272839	2.775475	-3.287930
H	-1.971511	5.063739	-2.304224
H	-0.578802	5.346877	-0.263739
H	2.462674	0.541085	-1.066554
H	4.886326	0.310369	-1.251255
H	6.321772	0.277941	0.777332
H	5.290107	0.493240	3.014513
H	3.292405	0.765916	4.419209
H	0.883806	1.037986	4.663408
H	-1.414472	1.350953	1.876604
H	1.037576	2.784030	1.360205
H	-1.364343	0.715172	4.735162
H	-1.162703	2.428983	4.300883
H	-2.608469	1.532632	3.798329
H	-2.476807	-2.189048	-0.925948
H	-3.260629	-1.258800	-2.073039
H	-4.258934	-1.012971	0.209486
H	-3.631896	0.492975	-0.485197
H	-2.680376	-0.396109	0.728237
H	-4.065555	-2.054950	-4.237111
H	-4.324645	-0.460055	-4.135305

H	-6.474324	-1.641298	-4.179350
H	-6.203867	-0.784907	-2.652204
H	-5.914340	-2.523269	-2.748444

(*aS*)-**A·H**$^+$

Geometry optimization in gas phase, B3LYP/6-311G**

```
SCF Done:  E(RB3LYP) =  -1476.12690156
Zero-point correction=                           0.535805 (Hartree/Particle)
Thermal correction to Energy=                    0.569369
Thermal correction to Enthalpy=                  0.570314
Thermal correction to Gibbs Free Energy=         0.465410
Sum of electronic and zero-point Energies=          -1475.591097
Sum of electronic and thermal Energies=             -1475.557532
Sum of electronic and thermal Enthalpies=           -1475.556588
Sum of electronic and thermal Free Energies=        -1475.661492
```

Energy in DMA:

SCF Done: E(RM06L) = -1476.15156772

Energy in NMP:

SCF Done: E(RM06L) = -1476.15127498

Cartesian coordinates:

C	-0.178905	-2.910779	0.320720
C	-0.118584	-1.846194	-0.650583
C	0.246715	-2.262148	-1.958193
C	0.609504	-3.556943	-2.307989
C	0.579736	-4.528110	-1.324831
C	0.177211	-4.206768	-0.028867
C	-0.497431	-0.470618	-0.436199
C	-0.477699	0.435505	-1.549325
C	-0.164148	-0.101127	-2.820972
O	0.207775	-1.381943	-2.984492
C	-0.215545	0.621910	-4.025216
C	-0.559040	1.940140	-3.984233
C	-0.784925	2.607344	-2.748445
C	-0.707951	1.890455	-1.509009
C	-0.814078	2.662469	-0.331832
C	-1.027269	4.028648	-0.373383
C	-1.165588	4.706876	-1.591533
C	-1.034921	3.998090	-2.763330
C	-0.962413	-0.041629	0.891509
C	-2.350682	0.281230	1.082180
C	-2.802329	0.666407	2.381439
C	-1.858901	0.716283	3.443487
C	-0.544202	0.405661	3.256185
C	-0.048966	0.026788	1.969746
C	-4.168596	0.958230	2.598143
C	-5.082642	0.874727	1.573178
C	-4.649602	0.474249	0.294737
C	-3.325308	0.176983	0.056449
N	1.277680	-0.206265	1.794270
C	2.248272	-0.189272	2.873729
N	3.130491	-0.076575	-0.506893
C	3.365339	1.312734	-0.938202
O	-0.588352	-2.637162	1.571666
N	5.822401	-0.925227	0.922916
C	6.372809	-2.174515	0.365169
H	-0.719624	2.195709	0.631708
H	-1.090443	4.577097	0.559027
H	-1.354030	5.773278	-1.608993
H	-1.103629	4.500025	-3.721782
H	-0.633952	2.509332	-4.903913
H	0.003946	0.101875	-4.948145
H	0.876162	-3.771815	-3.333651
H	0.851633	-5.549162	-1.562507
H	0.133124	-4.982039	0.728825
H	-3.035721	-0.151829	-0.933447
H	-5.368653	0.388049	-0.511896
H	-6.127495	1.100744	1.747390
H	-4.485829	1.243561	3.595489

H	-2.203716	1.011038	4.429084
H	0.140981	0.462931	4.090322
H	1.702375	-0.221664	0.856957
H	2.001546	-0.916529	3.653430
H	2.343422	0.798500	3.341396
H	3.215898	-0.457086	2.450055
H	-0.662982	-3.457091	2.075091
H	3.028081	-0.672695	-1.323821
H	3.963653	-0.418202	-0.012908
H	4.245027	1.439390	-1.582058
H	2.493676	1.690092	-1.477730
H	3.500319	1.943971	-0.057174
H	7.397291	-2.399125	0.685644
H	5.734400	-3.011246	0.656434
H	6.364009	-2.116711	-0.725382
H	6.423626	-0.145224	0.671286
H	5.842357	-0.968137	1.937958

Isomerization from (Λ)-**D** to (Δ)-**E**

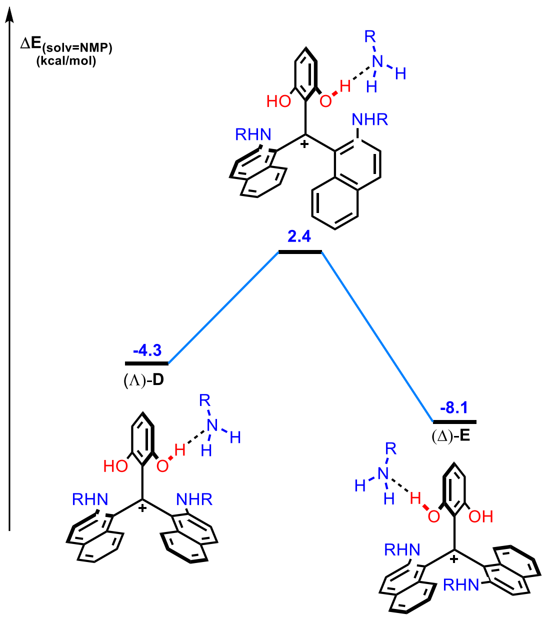

TS

Geometry optimization in gas phase, B3LYP/6-311G**

SCF Done: E(RB3LYP) = -1476.12055369

Zero-point correction=	0.537392	(Hartree/Particle)
Thermal correction to Energy=	0.568988	
Thermal correction to Enthalpy=	0.569932	
Thermal correction to Gibbs Free Energy=	0.474654	
Sum of electronic and zero-point Energies=	-1475.583161	
Sum of electronic and thermal Energies=	-1475.551565	
Sum of electronic and thermal Enthalpies=	-1475.550621	
Sum of electronic and thermal Free Energies=	-1475.645900	

Energy in DMA:

SCF Done: E(RM06L) = -1476.14331884

Energy in NMP:

SCF Done: E(RM06L) = -1476.14297939

Cartesian coordinates:

C	-1.198107	-2.494018	0.611254
C	0.015277	-1.858709	0.265855
C	1.187790	-2.608022	0.428555
C	1.210073	-3.850842	1.050028
C	0.009992	-4.401841	1.483011
C	-1.188901	-3.748637	1.239813
C	0.042629	-0.404225	-0.184008

C	-0.144072	-0.028793	-1.489977
C	-0.302966	-1.056195	-2.538300
C	-1.341804	-0.893152	-3.504126
C	-1.899014	0.337960	-3.668148
C	-1.441395	1.499318	-2.957976
C	-0.480188	1.355969	-1.917526
C	0.052803	2.514348	-1.351299
C	-0.382435	3.772655	-1.763513
C	-1.368373	3.910264	-2.739391
C	-1.889727	2.774881	-3.338321
N	0.515905	-2.087316	-2.569824
C	0.424423	-3.226180	-3.485119
O	2.315583	-2.100705	-0.188436
O	-2.361197	-1.886977	0.283804
C	0.184448	0.594163	0.940249
C	1.490277	1.050150	1.324064
C	1.637177	1.891364	2.475118
C	0.474536	2.294489	3.175294
C	-0.768679	1.927280	2.747188
C	-0.951309	1.081485	1.612449
C	2.920215	2.337274	2.868909
C	4.040748	2.019578	2.143404
C	3.902878	1.245700	0.973349
C	2.670296	0.774667	0.576980
N	-2.226567	0.815909	1.170599
C	-3.415729	1.187671	1.911878
H	1.296307	-2.086138	-1.917547
H	0.832027	2.449293	-0.609716
H	0.062277	4.654685	-1.318105
H	-1.709659	4.895170	-3.041956
H	-2.635916	2.860402	-4.120298
H	-2.664117	0.475481	-4.424987
H	-1.624590	-1.717677	-4.144729
H	2.147102	-4.387747	1.165024
H	0.007199	-5.366416	1.978537
H	-2.127068	-4.206500	1.519376
H	2.600677	0.186953	-0.325908
H	4.776896	1.025938	0.369797
H	5.019081	2.375083	2.449948
H	2.998268	2.958310	3.756333
H	0.580753	2.921780	4.052533
H	-1.639643	2.277083	3.287310
H	-2.330562	-0.007354	0.593305
H	-3.171320	-2.385516	0.621423
H	-3.456573	0.750272	2.920306
H	-3.505332	2.273607	2.008317
H	-4.285301	0.839666	1.353329
H	3.107425	-2.531445	0.153676
H	1.245211	-3.905677	-3.260784
H	0.508550	-2.897394	-4.522972
H	-0.519332	-3.758723	-3.346488
N	-4.666749	-3.044851	1.163742
C	-4.878903	-2.898411	2.620687
H	-4.755726	-4.022529	0.897085
H	-5.404354	-2.557926	0.659461
H	-5.841111	-3.288199	2.960936
H	-4.817930	-1.842378	2.885694
H	-4.083566	-3.421155	3.155036

Isomerization without extra amine

From (Λ)-**D** to (Δ)-**D**

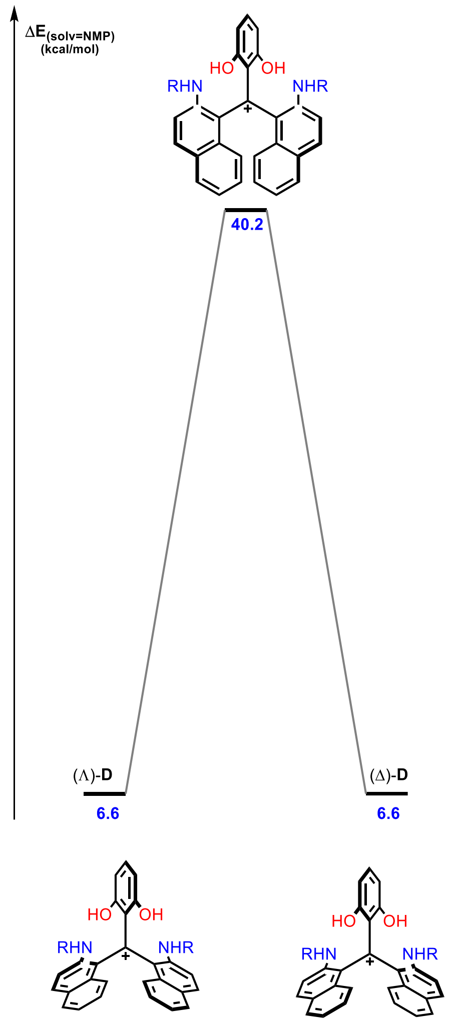

(Λ)-**D**

Geometry optimization in gas phase, B3LYP/6-311G**

```
 SCF Done:  E(RB3LYP) =  -1380.21590005
 Zero-point correction=                           0.470882 (Hartree/Particle)
 Thermal correction to Energy=                    0.498957
 Thermal correction to Enthalpy=                  0.499902
 Thermal correction to Gibbs Free Energy=         0.413083
 Sum of electronic and zero-point Energies=          -1379.745018
 Sum of electronic and thermal Energies=             -1379.716943
 Sum of electronic and thermal Enthalpies=           -1379.715999
 Sum of electronic and thermal Free Energies=        -1379.802817
```

Energy in DMA:

```
 SCF Done:  E(RM06L) =  -1380.25560336
```

Energy in NMP:

```
 SCF Done:  E(RM06L) =  -1380.25529654
```

Cartesian coordinates:

```
 C     1.074261     2.311717    -0.751563
 C     0.471727     2.109193     0.508434
 C     0.572710     3.179992     1.448485
 C     1.239220     4.376732     1.105402
 C     1.802588     4.543664    -0.139983
 C     1.716864     3.495192    -1.067096
 C    -0.162258     0.856060     0.934841
```

C	-0.849934	0.847809	2.200851
C	-0.677579	1.919724	3.122436
C	0.013170	3.032296	2.750456
C	0.033013	-0.369757	0.218960
C	-0.063698	-0.521352	-1.198583
C	0.831041	-1.420120	-1.888501
C	0.480537	-1.951823	-3.161863
C	-0.619717	-1.483851	-3.812528
C	-1.444990	-0.455759	-3.270976
C	-1.166436	0.067930	-1.971324
C	-2.039269	1.065890	-1.489601
C	-3.105269	1.524231	-2.243730
C	-3.366253	1.007769	-3.520157
C	-2.542499	0.022875	-4.018788
N	2.040525	-1.691039	-1.364273
H	2.335146	-1.160586	-0.555831
N	-1.727562	-0.135398	2.481112
C	-2.422805	-0.310840	3.747920
C	0.367251	-1.598336	1.033680
C	-0.522507	-2.679422	1.130698
C	-0.221251	-3.813608	1.882612
C	0.991373	-3.879831	2.559594
C	1.899108	-2.828901	2.488320
C	1.582107	-1.704459	1.728671
O	-1.744269	-2.512449	0.530512
O	2.480742	-0.688166	1.528047
H	-1.876733	1.505159	-0.518444
H	-3.742172	2.301608	-1.837530
H	-4.201283	1.377464	-4.102281
H	-2.725855	-0.403132	-4.999100
H	-0.865014	-1.874237	-4.794539
H	1.118491	-2.686821	-3.632291
H	-0.938472	-4.624327	1.954586
H	1.231087	-4.757597	3.147226
H	2.854761	-2.889193	2.998128
H	1.027552	1.540262	-1.503923
H	2.154575	3.611386	-2.051947
H	2.304735	5.467175	-0.400403
H	1.299875	5.165861	1.846872
H	0.117591	3.854624	3.450270
H	-1.139612	1.869025	4.098348
H	-1.974537	-0.776613	1.739878
H	3.245131	-0.803839	2.103974
H	-3.119801	0.509679	3.945037
H	-1.720233	-0.387056	4.581675
H	-2.992692	-1.237229	3.696686
C	3.004556	-2.638050	-1.906303
H	-2.252355	-3.330042	0.583031
H	3.848735	-2.692558	-1.220919
H	2.573095	-3.637937	-1.998732
H	3.376835	-2.319881	-2.884914

TS

Geometry optimization in gas phase, B3LYP/6-311G**

SCF Done: E(RB3LYP) = -1380.15818510
Zero-point correction= 0.469784 (Hartree/Particle)
Thermal correction to Energy= 0.497063
Thermal correction to Enthalpy= 0.498008
Thermal correction to Gibbs Free Energy= 0.414087
Sum of electronic and zero-point Energies= -1379.688401
Sum of electronic and thermal Energies= -1379.661122
Sum of electronic and thermal Enthalpies= -1379.660178
Sum of electronic and thermal Free Energies= -1379.744098

Energy in DMA:

SCF Done: E(RM06L) = -1380.20200619

Energy in NMP:

SCF Done: E(RM06L) = -1380.20167014

Cartesian coordinates:

C	2.809994	1.972882	-2.068868
C	1.815202	1.137759	-1.543248
C	0.581727	1.743184	-1.180346
C	0.312738	3.077819	-1.534285
C	1.289252	3.854927	-2.131335
C	2.557441	3.307628	-2.350909
C	2.092961	-0.257928	-1.154818
C	1.493849	-0.590688	0.130592
C	0.192944	-0.036406	0.407297
C	-0.281118	1.029761	-0.287265
C	2.578349	-1.317573	-1.996316
C	2.398794	-1.446321	-3.416167
C	2.042724	-2.745492	-3.970398
C	0.891388	-2.790757	-4.835850
C	0.403696	-1.665427	-5.418786
C	1.141797	-0.440257	-5.338286
C	2.204444	-0.362508	-4.398046
C	3.163654	0.639916	-4.593746
C	3.010268	1.602635	-5.580936
C	1.877921	1.607009	-6.401792
C	0.957681	0.579992	-6.289010
N	2.680091	-3.892401	-3.702108
H	3.512542	-3.837630	-3.139527
N	2.044773	-1.399166	1.045629
C	1.406616	-1.868957	2.269633
C	3.119530	-2.536296	-1.282985
C	2.330719	-3.607967	-0.827371
C	2.900725	-4.699367	-0.174448
C	4.278988	-4.732825	0.032814
C	5.087319	-3.692237	-0.405063
C	4.506224	-2.598480	-1.061075
O	0.991469	-3.517763	-1.059505
O	5.245037	-1.547777	-1.513603
H	4.066901	0.620251	-4.001321
H	3.782602	2.349658	-5.722810
H	1.751080	2.375218	-7.154856
H	0.120644	0.514800	-6.975432
H	-0.472283	-1.719550	-6.055867
H	0.422296	-3.746160	-5.025630
H	2.273315	-5.515241	0.168849
H	4.724158	-5.579943	0.540448
H	6.159547	-3.720068	-0.243352
H	3.807377	1.578800	-2.198293
H	3.355727	3.932487	-2.734211
H	1.090658	4.891286	-2.375974
H	-0.648476	3.507167	-1.273926
H	-1.268368	1.423592	-0.071741
H	-0.402493	-0.479268	1.193483
H	2.975643	-1.735680	0.865121
H	6.176719	-1.690640	-1.311790
H	0.527598	-2.483353	2.053324
H	1.107736	-1.033800	2.907233
H	2.127010	-2.476684	2.814008
C	2.255093	-5.225536	-4.112440
H	0.546356	-4.295942	-0.705697
H	3.005110	-5.938207	-3.774451
H	1.293650	-5.494114	-3.664986
H	2.174445	-5.298972	-5.199274

(Δ)-D

Geometry optimization in gas phase, B3LYP/6-311G**

SCF Done: E(RB3LYP) = -1380.21585901
Zero-point correction= 0.470853 (Hartree/Particle)
Thermal correction to Energy= 0.498989
Thermal correction to Enthalpy= 0.499933
Thermal correction to Gibbs Free Energy= 0.412637
Sum of electronic and zero-point Energies= -1379.745006
Sum of electronic and thermal Energies= -1379.716870

Sum of electronic and thermal Enthalpies= -1379.715926
Sum of electronic and thermal Free Energies= -1379.803222

Energy in DMA:

SCF Done: E(RM06L) = -1380.25557636

Energy in NMP:

SCF Done: E(RM06L) = -1380.25526956

Cartesian coordinates:

C	-2.212011	1.552451	-0.475774
C	-1.564798	1.487060	0.776664
C	-2.165034	2.216021	1.848704
C	-3.342973	2.965329	1.637167
C	-3.938047	3.017709	0.396424
C	-3.361590	2.299265	-0.660640
C	-0.385119	0.661529	1.062134
C	0.243620	0.812246	2.350321
C	-0.420830	1.506776	3.400887
C	-1.581038	2.172478	3.147712
C	0.034146	-0.386881	0.180960
C	0.153796	-0.275699	-1.238976
C	-0.199854	-1.395021	-2.078717
C	0.339052	-1.511613	-3.391359
C	1.049090	-0.480586	-3.926286
C	1.269265	0.738607	-3.221720
C	0.801660	0.876982	-1.879028
C	1.078073	2.100698	-1.234127
C	1.754624	3.123070	-1.875956
C	2.202094	2.980114	-3.196395
C	1.963421	1.793223	-3.853296
N	-1.110257	-2.288459	-1.649798
H	-1.606945	-2.083440	-0.793388
N	1.500866	0.366613	2.538215
C	2.220207	0.375945	3.803744
C	0.376753	-1.719805	0.806500
C	-0.604720	-2.510374	1.424272
C	-0.299110	-3.737785	2.009370
C	1.012299	-4.198730	1.977671
C	2.012093	-3.446206	1.371593
C	1.688492	-2.218930	0.795713
O	-1.895107	-2.057050	1.323124
O	2.647349	-1.384894	0.280326
H	0.745228	2.266879	-0.222082
H	1.932840	4.051337	-1.345357
H	2.726522	3.789238	-3.689457
H	2.306872	1.649662	-4.871908
H	1.432537	-0.564867	-4.937606
H	0.137454	-2.395653	-3.980045
H	-1.083287	-4.335026	2.462286
H	1.257255	-5.152603	2.428641
H	3.038917	-3.796064	1.365427
H	-1.804216	1.026043	-1.324323
H	-3.817317	2.330932	-1.643639
H	-4.836065	3.601931	0.238128
H	-3.772335	3.501791	2.476244
H	-2.066981	2.721257	3.947466
H	0.024634	1.546656	4.385017
H	2.015490	0.046043	1.729536
H	3.496502	-1.839922	0.244830
H	1.676837	-0.174266	4.576284
H	2.404327	1.395272	4.156850
H	3.182100	-0.110808	3.650947
C	-1.495346	-3.506261	-2.348741
H	-2.484019	-2.616580	1.842250
H	-2.198605	-4.050123	-1.720271
H	-1.986051	-3.286431	-3.301722
H	-0.632037	-4.150453	-2.534121

From (Δ)-**E** to (Λ)-**E**

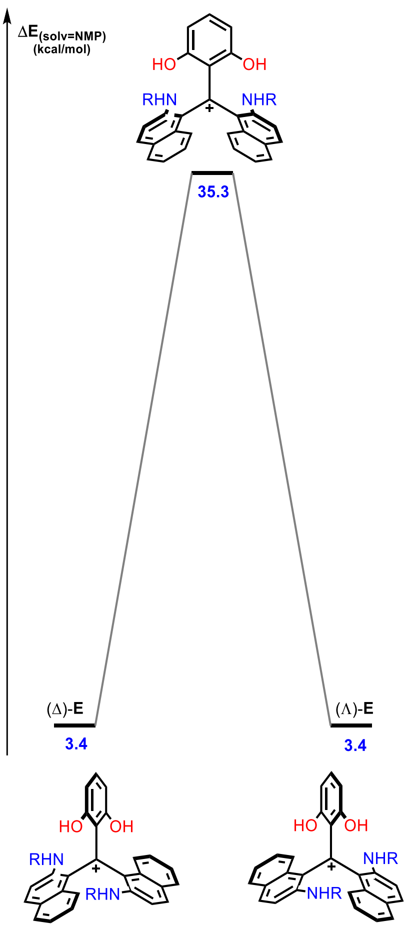

(Δ)-**E**

Geometry optimization in gas phase, B3LYP/6-311G**

```
 SCF Done:  E(RB3LYP) =  -1380.22180138
 Zero-point correction=                           0.470547 (Hartree/Particle)
 Thermal correction to Energy=                    0.498771
 Thermal correction to Enthalpy=                  0.499715
 Thermal correction to Gibbs Free Energy=         0.412871
 Sum of electronic and zero-point Energies=          -1379.751255
 Sum of electronic and thermal Energies=             -1379.723031
 Sum of electronic and thermal Enthalpies=           -1379.722087
 Sum of electronic and thermal Free Energies=        -1379.808930
```

Energy in DMA:

```
 SCF Done:  E(RM06L) =  -1380.26069769
```

Energy in NMP:

```
 SCF Done:  E(RM06L) =  -1380.26039161
```

Cartesian coordinates:

```
 C      0.201730      2.840683      1.033395
 C     -0.178349      1.697636      1.753586
 C     -0.302314      1.814327      3.161587
 C      0.051550      3.011585      3.811381
 C      0.474719      4.107368      3.081774
 C      0.523576      4.023142      1.685979
 C     -0.511148      0.423741      1.089146
 C     -1.381016     -0.487561      1.843166
 C     -1.423573     -0.351309      3.268575
 C     -0.874841      0.725086      3.891604
 C      0.166874     -0.010203     -0.051876
 C     -0.276032     -1.094288     -0.940569
 C      0.555290     -2.233969     -1.117370
 C      0.224583     -3.196469     -2.119420
 C     -0.908374     -3.067672     -2.865893
```

C	-1.806936	-1.980037	-2.687080
C	-1.491350	-0.974219	-1.719785
C	-2.383918	0.123993	-1.609463
C	-3.528492	0.197228	-2.376006
C	-3.839219	-0.805823	-3.312388
C	-2.980827	-1.870346	-3.467068
N	1.615836	-2.461361	-0.299588
H	1.756355	-1.849060	0.490141
N	-2.055022	-1.481416	1.270107
C	-2.794832	-2.531091	1.966998
C	1.489643	0.594533	-0.414075
C	1.675496	1.228554	-1.658138
C	2.908112	1.776126	-2.014690
C	3.977695	1.692175	-1.132699
C	3.832894	1.063522	0.099928
C	2.598464	0.523848	0.446970
O	0.576682	1.352723	-2.449533
O	2.419972	-0.168283	1.619119
H	-2.157737	0.930882	-0.925561
H	-4.189352	1.049121	-2.263414
H	-4.738049	-0.731493	-3.912070
H	-3.191224	-2.643999	-4.197582
H	-1.145070	-3.817281	-3.613419
H	0.871139	-4.049960	-2.268964
H	3.020075	2.276716	-2.970497
H	4.935269	2.116839	-1.408517
H	4.674829	0.977967	0.778461
H	0.214402	2.815748	-0.046927
H	0.803332	4.892628	1.102886
H	0.735847	5.031479	3.582746
H	-0.038568	3.071294	4.890393
H	-0.933650	0.800214	4.972363
H	-1.927288	-1.108905	3.851804
H	-2.066177	-1.518615	0.260421
H	3.217957	-0.103062	2.155852
H	-3.620206	-2.112885	2.548002
H	-2.142548	-3.104166	2.629996
H	-3.206399	-3.203742	1.217225
C	2.578556	-3.539276	-0.460040
H	0.816750	1.771049	-3.283839
H	3.360075	-3.409743	0.287599
H	2.124132	-4.524091	-0.307720
H	3.048273	-3.519109	-1.447680

TS

Geometry optimization in gas phase, B3LYP/6-311G**

SCF Done: E(RB3LYP) = -1380.16320830
Zero-point correction= 0.469739 (Hartree/Particle)
Thermal correction to Energy= 0.496735
Thermal correction to Enthalpy= 0.497679
Thermal correction to Gibbs Free Energy= 0.413997
Sum of electronic and zero-point Energies= -1379.693470
Sum of electronic and thermal Energies= -1379.666473
Sum of electronic and thermal Enthalpies= -1379.665529
Sum of electronic and thermal Free Energies= -1379.749211

Energy in DMA:

SCF Done: E(RM06L) = -1380.20990931

Energy in NMP:

SCF Done: E(RM06L) = -1380.20954459

Cartesian coordinates:

C	9.204272	19.381716	-2.353657
C	9.437827	18.293273	-1.474178
C	8.924926	18.429574	-0.145177
C	8.220702	19.593760	0.235307
C	8.030558	20.634077	-0.645370
C	8.535928	20.517949	-1.951973
C	10.133790	17.091927	-1.866342
C	10.302990	16.057142	-0.909466

C	9.778764	16.239376	0.409286
C	9.119711	17.370208	0.774899
C	10.740274	17.054709	-3.248547
C	9.893631	16.416973	-4.310692
C	9.677772	15.036105	-4.379604
C	8.529341	14.513001	-5.012316
C	7.672243	15.350009	-5.677272
C	7.991664	16.716193	-5.859683
C	9.158210	17.264043	-5.228597
C	9.511146	18.595112	-5.574092
C	8.744389	19.345733	-6.435486
C	7.559080	18.824167	-6.992349
C	7.198179	17.532368	-6.706809
N	10.707412	14.198485	-3.895367
H	11.590485	14.467805	-4.312709
N	11.038422	14.933127	-1.149209
C	11.254752	13.874273	-0.178217
C	11.945988	17.734026	-3.549076
C	12.636825	17.528223	-4.819508
C	13.870253	18.093151	-5.089445
C	14.510704	18.871240	-4.128145
C	13.940172	19.072643	-2.882421
C	12.699475	18.521354	-2.576803
O	12.057063	16.719849	-5.723641
O	12.163960	18.719961	-1.375309
H	10.420689	19.033532	-5.187560
H	9.061345	20.349875	-6.692898
H	6.954709	19.432712	-7.653720
H	6.305925	17.100126	-7.146114
H	6.778328	14.953298	-6.144786
H	8.337706	13.449059	-4.993424
H	14.342056	17.912838	-6.048978
H	15.473945	19.312843	-4.354018
H	14.453057	19.666984	-2.134483
H	9.529518	19.324785	-3.378284
H	8.381111	21.321946	-2.662615
H	7.489082	21.522231	-0.343457
H	7.827831	19.649725	1.244866
H	8.725968	17.467131	1.780793
H	9.905556	15.443715	1.129081
H	11.125378	14.663680	-2.124609
H	12.762015	19.239374	-0.821241
H	10.325590	13.391158	0.149959
H	11.780533	14.244251	0.706042
H	11.886485	13.115579	-0.640696
C	10.532375	12.743857	-3.900923
H	12.602261	16.668377	-6.519270
H	11.439187	12.285529	-3.504680
H	9.700346	12.466936	-3.251423
H	10.349807	12.334622	-4.901102

(Λ)-E

Geometry optimization in gas phase, B3LYP/6-311G**

SCF Done: E(RB3LYP) = -1380.22185135
Zero-point correction= 0.470687 (Hartree/Particle)
Thermal correction to Energy= 0.498863
Thermal correction to Enthalpy= 0.499808
Thermal correction to Gibbs Free Energy= 0.413087
Sum of electronic and zero-point Energies= -1379.751164
Sum of electronic and thermal Energies= -1379.722988
Sum of electronic and thermal Enthalpies= -1379.722044
Sum of electronic and thermal Free Energies= -1379.808765

Energy in DMA:

SCF Done: E(RM06L) = -1380.26064614

Energy in NMP:

SCF Done: E(RM06L) = -1380.26033915

Cartesian coordinates:

C	-2.046402	1.096340	1.620760

C	-1.076126	0.222808	2.177855
C	-1.142566	-0.016251	3.586697
C	-2.159886	0.586463	4.361472
C	-3.101654	1.406119	3.782478
C	-3.034747	1.660549	2.400432
C	-0.015501	-0.388475	1.402520
C	0.908836	-1.254945	2.048309
C	0.829522	-1.439396	3.462375
C	-0.155645	-0.842813	4.191076
C	0.186718	-0.041490	-0.011318
C	-0.715511	-0.308656	-1.043255
C	-1.644494	-1.442598	-0.959586
C	-1.959023	-2.145300	-2.167692
C	-1.608432	-1.650250	-3.384254
C	-0.986541	-0.370131	-3.532155
C	-0.598581	0.335291	-2.364607
C	-0.174607	1.664908	-2.513863
C	-0.061616	2.242638	-3.771284
C	-0.370423	1.513693	-4.924964
C	-0.842318	0.219727	-4.801873
N	-2.129107	-1.894980	0.194182
H	-1.949676	-1.346721	1.023755
N	1.817832	-1.967105	1.333535
C	2.864325	-2.799084	1.905393
C	1.510431	0.591407	-0.319031
C	2.448810	-0.025796	-1.163969
C	3.687266	0.549547	-1.430195
C	4.009506	1.770003	-0.845556
C	3.112040	2.408566	0.000663
C	1.873820	1.821544	0.262446
O	2.109314	-1.261816	-1.656343
O	0.927297	2.441911	1.016854
H	0.035998	2.263078	-1.638759
H	0.258235	3.274604	-3.855054
H	-0.271134	1.969951	-5.902263
H	-1.133206	-0.345084	-5.680720
H	-1.871040	-2.204694	-4.279114
H	-2.510268	-3.072622	-2.104867
H	4.397331	0.040374	-2.072969
H	4.971122	2.224891	-1.049841
H	3.360649	3.365890	0.446105
H	-2.004316	1.342090	0.568332
H	-3.761239	2.321108	1.941065
H	-3.879331	1.861531	4.383195
H	-2.181262	0.393049	5.428535
H	-0.202307	-1.006102	5.262472
H	1.547687	-2.079909	3.955298
H	1.772396	-1.934368	0.325818
H	1.282882	3.257722	1.386631
H	2.458168	-3.670697	2.429744
H	3.495523	-2.235807	2.598657
H	3.493907	-3.158155	1.092250
C	-2.907468	-3.117651	0.378048
H	2.792052	-1.563965	-2.266004
H	-3.123581	-3.219767	1.439594
H	-3.854149	-3.068258	-0.165134
H	-2.349490	-3.997343	0.048952

From (Λ)-**D** to (Δ)-**E**

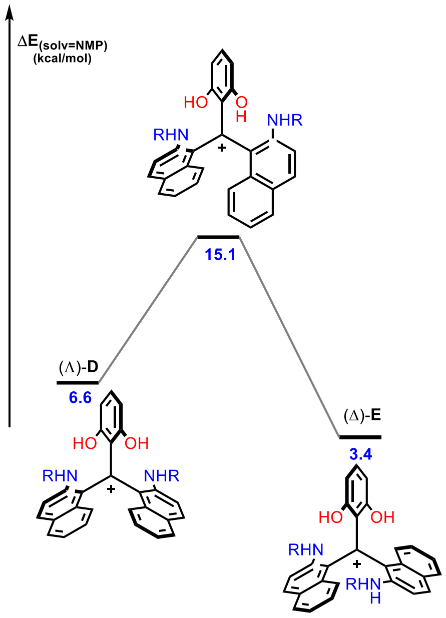

TS

Geometry optimization in gas phase, B3LYP/6-311G**

SCF Done: E(RB3LYP) = -1380.20455285	
Zero-point correction=	0.471044 (Hartree/Particle)
Thermal correction to Energy=	0.498260
Thermal correction to Enthalpy=	0.499204
Thermal correction to Gibbs Free Energy=	0.415570
Sum of electronic and zero-point Energies=	-1379.733508
Sum of electronic and thermal Energies=	-1379.706293
Sum of electronic and thermal Enthalpies=	-1379.705349
Sum of electronic and thermal Free Energies=	-1379.788983

Energy in DMA:

SCF Done: E(RM06L) = -1380.24206677

Energy in NMP:

SCF Done: E(RM06L) = -1380.24171383

Cartesian coordinates:

C	-2.724718	0.902579	0.488831
C	-1.573671	1.193586	1.274766
C	-1.730505	2.185129	2.301907
C	-3.000194	2.757518	2.550811
C	-4.091279	2.422822	1.786048
C	-3.940317	1.502878	0.733407
C	-0.274029	0.629221	1.022389
C	0.852042	1.149345	1.684375
C	0.660365	2.126349	2.705014
C	-0.579750	2.603177	3.019309
C	-0.164311	-0.523072	0.046747
C	0.208340	-0.344723	-1.257823
C	0.389398	-1.515120	-2.149724
C	1.545292	-1.556182	-2.988874
C	2.219316	-0.396018	-3.233859
C	1.776809	0.881661	-2.740912
C	0.701910	0.938533	-1.818705
C	0.197412	2.199085	-1.471193
C	0.769552	3.354357	-1.987856
C	1.871747	3.298835	-2.842947
C	2.368111	2.064168	-3.225910
N	-0.508905	-2.479842	-2.160045
H	-1.366389	-2.327287	-1.632398
N	2.136975	0.778357	1.345418
C	3.298924	1.121199	2.160912

C	-0.382722	-1.887327	0.681687
C	-1.638428	-2.520127	0.724456
C	-1.888206	-3.633561	1.519754
C	-0.846384	-4.192675	2.258206
C	0.434536	-3.672512	2.154239
C	0.662674	-2.545090	1.358340
O	-2.587422	-2.060017	-0.162237
O	1.935828	-2.075363	1.177033
H	-0.668676	2.278033	-0.830974
H	0.347967	4.319293	-1.724118
H	2.319405	4.211117	-3.226206
H	3.201114	1.993400	-3.909681
H	3.073741	-0.415067	-3.897017
H	1.829991	-2.469720	-3.486824
H	-2.881675	-4.077865	1.536012
H	-1.032765	-5.051363	2.888912
H	1.263363	-4.139620	2.675934
H	-2.635322	0.215190	-0.337020
H	-4.788901	1.269278	0.097214
H	-5.056035	2.877605	1.978520
H	-3.086132	3.494320	3.346830
H	-0.692828	3.329817	3.814327
H	1.522531	2.492118	3.252148
H	2.210494	-0.110476	0.884616
H	2.551145	-2.549157	1.748010
H	3.222503	0.763333	3.197126
H	3.466265	2.198636	2.179279
H	4.172826	0.666740	1.700223
C	-0.414047	-3.721775	-2.925294
H	-3.463891	-2.378655	0.080670
H	-1.304296	-4.313139	-2.713470
H	-0.367011	-3.522300	-3.997977
H	0.464894	-4.300243	-2.626567

From Azaoxa (*M*)-**2** to Diaza (*M*)-**3** *via* intermediate (*aR*)-**G**.

Attack on the *Re* face.

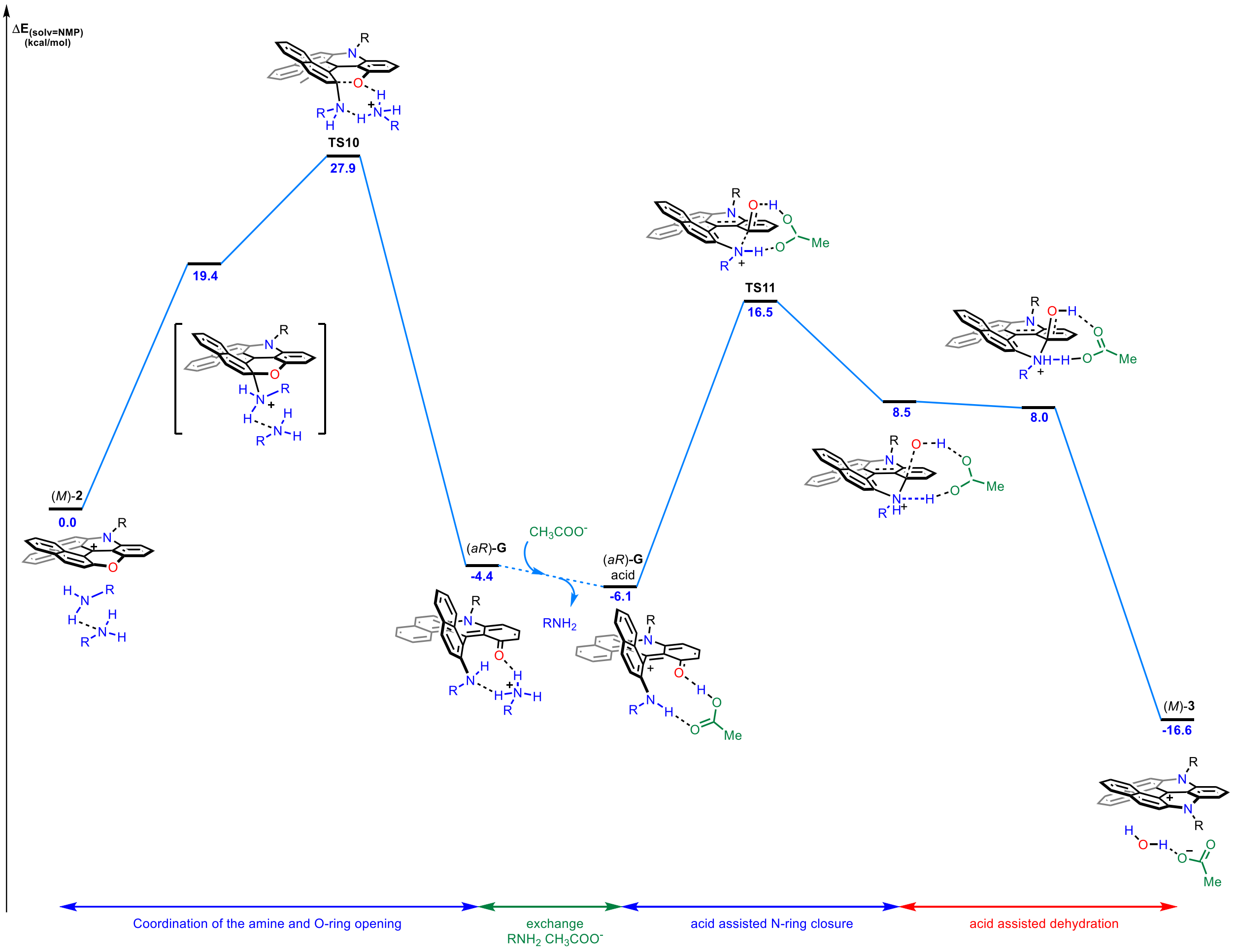

(*M*)-**2**

Geometry optimization in gas phase, B3LYP/6-311G**

```
 SCF Done:  E(RB3LYP) =  -1399.69462330
 Zero-point correction=                         0.510750 (Hartree/Particle)
 Thermal correction to Energy=                  0.541823
 Thermal correction to Enthalpy=                0.542768
 Thermal correction to Gibbs Free Energy=       0.444503
 Sum of electronic and zero-point Energies=        -1399.183874
 Sum of electronic and thermal Energies=           -1399.152800
 Sum of electronic and thermal Enthalpies=         -1399.151856
 Sum of electronic and thermal Free Energies=      -1399.250120
```

Energy in DMA:

```
 SCF Done:  E(RM06L) =  -1399.72584844
```

Energy in NMP:

```
 SCF Done:  E(RM06L) =  -1399.72557328
```

Cartesian coordinates:

```
 C    -0.449637     0.041819    -4.081434
 C    -0.004116    -0.367984    -2.818286
 C    -0.829539    -0.155385    -1.682521
 C    -2.086366     0.458377    -1.868143
 C    -2.520788     0.877746    -3.112041
 C    -1.688992     0.658803    -4.208982
 C    -0.406669    -0.522808    -0.377714
 C     1.002830    -0.690934    -0.202728
 C     1.763907    -1.051941    -1.353357
 N     1.204044    -1.016178    -2.612136
 C     3.116619    -1.473362    -1.197389
```

C	3.718495	-1.432071	0.024883
C	3.061975	-0.898216	1.168767
C	1.706208	-0.479392	1.052316
C	1.135207	0.208138	2.144649
C	1.847163	0.402414	3.312291
C	3.159340	-0.081388	3.447933
C	3.761330	-0.712238	2.382155
C	-1.459272	-0.684487	0.602642
C	-2.641299	0.033307	0.368759
C	-3.670114	0.146716	1.323651
C	-3.572688	-0.557349	2.489100
C	-2.489671	-1.451186	2.723824
C	-1.430841	-1.549428	1.768405
C	-2.488031	-2.284228	3.866980
C	-1.503534	-3.226650	4.052204
C	-0.495329	-3.370998	3.083073
C	-0.455853	-2.550982	1.974409
N	-0.515941	2.754394	0.309868
C	-1.401291	3.521735	1.197892
O	-2.880164	0.687191	-0.795075
N	-0.582929	4.477533	-2.364579
C	0.690646	5.162526	-2.640648
H	0.141946	0.621277	2.053198
H	1.387184	0.942123	4.131747
H	3.700509	0.066604	4.374507
H	4.789028	-1.051001	2.450440
H	4.749374	-1.754931	0.120633
H	3.689394	-1.794852	-2.053387
H	0.169499	-0.083412	-4.957228
H	-2.015053	0.979218	-5.191229
H	-3.486336	1.355418	-3.209689
H	0.324733	-2.702857	1.243608
H	0.259052	-4.139588	3.203582
H	-1.515392	-3.868771	4.924431
H	-3.295399	-2.183950	4.583675
H	-4.356612	-0.481340	3.234154
H	-4.519462	0.776806	1.094892
H	0.439233	2.829353	0.649418
H	-1.393272	3.089662	2.202588
H	-2.427944	3.456326	0.828059
H	-1.149351	4.587737	1.292372
H	-0.517220	3.194397	-0.615716
H	-0.912489	4.005104	-3.200993
H	-1.296456	5.161068	-2.128466
H	0.643788	5.906367	-3.446403
H	1.031629	5.666915	-1.734168
H	1.448186	4.421867	-2.906987
C	1.905715	-1.602689	-3.763879
H	1.174787	-1.859925	-4.525337
H	2.640829	-0.911299	-4.183847
H	2.401145	-2.521226	-3.459381

Structure 2

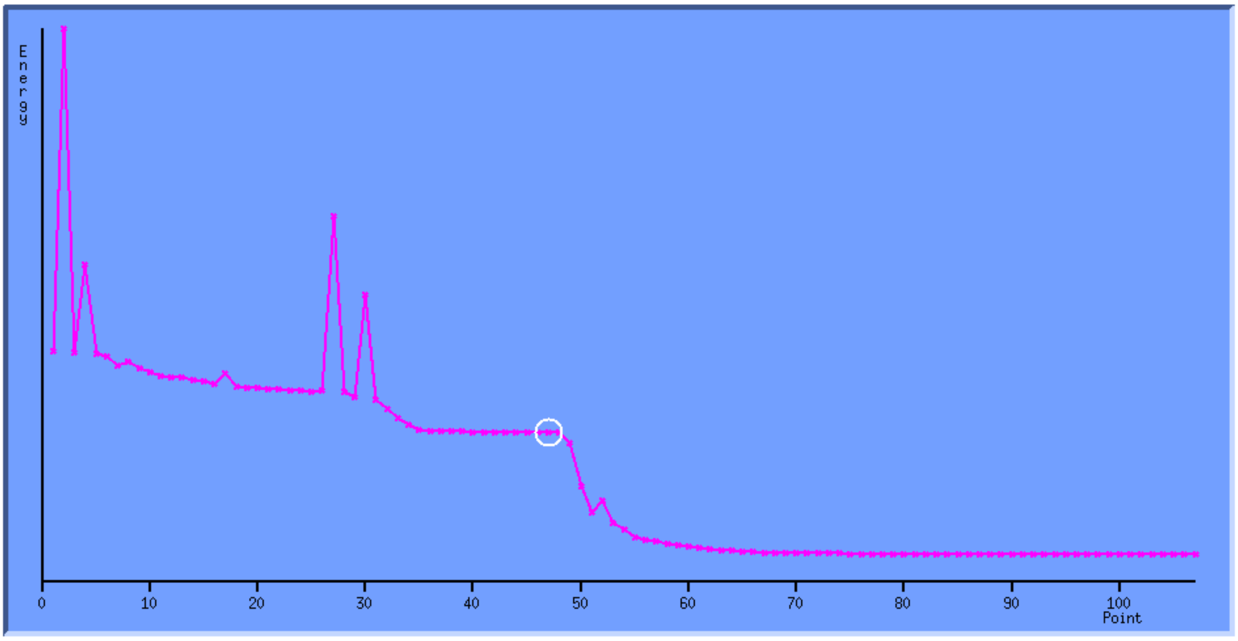

Geometry in gas phase, B3LYP/6-311G**

SCF Done: -1399.664427

Energy in DMA:

SCF Done: E(RM06L) = -1399.69502972

Energy in NMP:

SCF Done: E(RM06L) = -1399.69470489

Cartesian coordinates:

C	0.098923	-0.541592	-4.327605
C	0.450037	-0.772694	-2.988063
C	-0.262187	-0.123665	-1.944275
C	-1.391857	0.631128	-2.311484
C	-1.768871	0.827530	-3.630100
C	-0.994917	0.254158	-4.634426
C	0.033767	-0.402757	-0.552579
C	1.354750	-0.979806	-0.307731
C	1.994572	-1.658567	-1.374099
N	1.440171	-1.679315	-2.643857
C	3.238494	-2.321780	-1.171338
C	3.881446	-2.246887	0.025692
C	3.368028	-1.460308	1.088106
C	2.111197	-0.799666	0.924577
C	1.707140	0.048212	1.983058
C	2.470527	0.216030	3.119274
C	3.684138	-0.472655	3.283349
C	4.121315	-1.296694	2.274080
C	-0.982049	-0.093517	0.379273
C	-1.853701	1.060552	-0.000520
C	-3.067875	1.294704	0.798033
C	-3.288724	0.618927	1.935816
C	-2.420538	-0.440355	2.392363
C	-1.302811	-0.829969	1.597168
C	-2.760267	-1.161276	3.548341
C	-2.043691	-2.281684	3.929743
C	-0.989948	-2.715233	3.120168
C	-0.632632	-2.011685	1.981686
N	-0.904508	2.485025	0.177159
C	-0.775677	3.023544	1.551442
O	-2.192091	1.188923	-1.355989
N	-1.958633	4.553360	-1.568022
C	-1.814802	5.969201	-1.160840
H	0.772829	0.576903	1.909535
H	2.123092	0.882843	3.900314
H	4.267414	-0.347430	4.187334

H	5.064507	-1.824273	2.362909
H	4.830339	-2.754069	0.159704
H	3.703806	-2.858329	-1.983708
H	0.679334	-0.972183	-5.129549
H	-1.252493	0.424567	-5.672642
H	-2.654476	1.409226	-3.849152
H	0.170631	-2.397457	1.373053
H	-0.445280	-3.616141	3.377705
H	-2.313164	-2.829578	4.824246
H	-3.616236	-0.836921	4.130341
H	-4.187528	0.818101	2.510543
H	-3.769768	2.022823	0.409675
H	0.016489	2.234943	-0.185051
H	-0.486572	2.232141	2.240130
H	-1.734180	3.429819	1.865462
H	-0.022372	3.812168	1.566699
H	-1.310557	3.223960	-0.461555
H	-1.531816	4.417822	-2.481430
H	-2.942935	4.331397	-1.697548
H	-2.256491	6.674125	-1.872107
H	-2.292257	6.116601	-0.190808
H	-0.755215	6.207405	-1.054855
C	1.919225	-2.629465	-3.654852
H	1.103132	-2.859860	-4.335537
H	2.764398	-2.234852	-4.226681
H	2.213161	-3.557580	-3.171215

TS10

Geometry optimization in gas phase, B3LYP/6-311G**

```
 SCF Done:  E(RB3LYP) =  -1399.64407156
 Zero-point correction=                           0.511266 (Hartree/Particle)
 Thermal correction to Energy=                    0.539505
 Thermal correction to Enthalpy=                  0.540449
 Thermal correction to Gibbs Free Energy=         0.453379
 Sum of electronic and zero-point Energies=          -1399.132806
 Sum of electronic and thermal Energies=             -1399.104567
 Sum of electronic and thermal Enthalpies=           -1399.103623
 Sum of electronic and thermal Free Energies=        -1399.190692
```

Energy in DMA:

```
 SCF Done:  E(RM06L) =  -1399.68147684
```

Energy in NMP:

```
 SCF Done:  E(RM06L) =  -1399.68108077
```

Cartesian coordinates:

C	9.086228	15.656565	-5.095923
C	9.585097	15.638404	-3.786269
C	8.958023	16.403109	-2.753014
C	7.766174	17.138197	-3.102250
C	7.231615	17.039835	-4.390157
C	7.912848	16.342251	-5.376176
C	9.352904	16.214898	-1.383479
C	10.616806	15.556813	-1.125465
C	11.259381	14.902246	-2.211506
N	10.651928	14.820197	-3.448877
C	12.546452	14.312867	-2.058130
C	13.194848	14.360861	-0.864705
C	12.626173	15.008913	0.261329
C	11.338585	15.621945	0.149658
C	10.868695	16.287026	1.307742
C	11.606432	16.339865	2.474381
C	12.860342	15.718268	2.571155
C	13.357914	15.063841	1.469620
C	8.393185	16.644313	-0.392171
C	7.786114	17.944731	-0.613054
C	6.663203	18.310594	0.236191
C	6.170773	17.464061	1.167271
C	6.719022	16.159490	1.379681
C	7.826196	15.735744	0.584614
C	6.139296	15.287385	2.322128

C	6.616455	14.004753	2.493995
C	7.684196	13.566484	1.696130
C	8.268788	14.402909	0.764865
N	8.691886	19.106163	-0.858061
C	9.314708	19.668216	0.364943
O	7.164503	17.947593	-2.244072
N	7.185214	20.664556	-2.464082
C	6.478261	21.856330	-1.919778
H	9.901782	16.755354	1.299956
H	11.196686	16.860637	3.332352
H	13.423021	15.754344	3.495989
H	14.327910	14.580495	1.506300
H	14.180346	13.918548	-0.770147
H	13.038631	13.860269	-2.904811
H	9.599960	15.145674	-5.894495
H	7.528159	16.336973	-6.389459
H	6.304773	17.557103	-4.604763
H	9.076296	14.016424	0.159357
H	8.055151	12.553517	1.804141
H	6.166396	13.340779	3.221653
H	5.296792	15.641773	2.906756
H	5.327005	17.770878	1.777039
H	6.222369	19.292249	0.100503
H	9.441964	18.777535	-1.465043
H	9.927197	18.936312	0.895325
H	8.538328	20.026387	1.039767
H	9.946478	20.509773	0.073983
H	7.872244	20.142548	-1.740352
H	7.716039	20.896471	-3.303260
H	6.556204	19.891160	-2.708982
H	5.792821	22.262044	-2.662515
H	5.918534	21.558749	-1.035003
H	7.212131	22.612632	-1.646012
C	11.107313	13.821646	-4.427676
H	10.244087	13.447768	-4.973007
H	11.827592	14.240113	-5.136242
H	11.555638	12.980521	-3.908197

(*aR*)-**G**-RNH_3^+

Geometry optimization in gas phase, B3LYP/6-311G**

SCF Done: E(RB3LYP) = -1399.69656459
Zero-point correction= 0.512732 (Hartree/Particle)
Thermal correction to Energy= 0.541874
Thermal correction to Enthalpy= 0.542818
Thermal correction to Gibbs Free Energy= 0.453241
Sum of electronic and zero-point Energies= -1399.183832
Sum of electronic and thermal Energies= -1399.154691
Sum of electronic and thermal Enthalpies= -1399.153746
Sum of electronic and thermal Free Energies= -1399.243323

Energy in DMA:

SCF Done: E(RM06L) = -1399.73303263

Energy in NMP:

SCF Done: E(RM06L) = -1399.73264613

Cartesian coordinates:

C	-0.293413	-1.470452	-4.034994
C	0.291486	-1.114238	-2.824702
C	-0.440144	-0.344750	-1.839985
C	-1.759320	0.209917	-2.234876
C	-2.322502	-0.263802	-3.443702
C	-1.605151	-1.064716	-4.304947
C	0.084029	-0.206302	-0.542440
C	1.416351	-0.645759	-0.252527
C	2.203251	-1.107867	-1.350576
N	1.601556	-1.463502	-2.529148
C	3.625818	-1.163780	-1.247527
C	4.241683	-0.886063	-0.068879
C	3.500159	-0.654304	1.123334
C	2.074130	-0.592569	1.063549

C	1.395545	-0.551853	2.303286
C	2.081375	-0.490389	3.503562
C	3.482246	-0.455716	3.540152
C	4.179343	-0.550626	2.356725
C	-0.753145	0.412127	0.541373
C	-0.486719	1.695019	0.998116
C	-1.232653	2.229186	2.080398
C	-2.223982	1.498027	2.679621
C	-2.539902	0.191103	2.233079
C	-1.793798	-0.365739	1.147020
C	-3.561451	-0.575389	2.848892
C	-3.837361	-1.851385	2.424911
C	-3.091305	-2.413248	1.366616
C	-2.096751	-1.694338	0.746233
N	0.451013	2.540047	0.333365
C	1.342688	3.380269	1.157545
O	-2.363786	1.078142	-1.529676
N	-1.793925	3.555209	-1.285770
C	-2.881554	4.289122	-0.580572
H	0.322774	-0.613171	2.339650
H	1.517585	-0.484242	4.429073
H	4.005939	-0.392111	4.486193
H	5.263251	-0.579991	2.355555
H	5.324885	-0.880371	-0.018320
H	4.224729	-1.362103	-2.123024
H	0.244053	-2.023871	-4.786804
H	-2.053061	-1.366524	-5.245619
H	-3.320429	0.077658	-3.687552
H	-1.541950	-2.150791	-0.062978
H	-3.305831	-3.423862	1.038970
H	-4.619670	-2.430298	2.900898
H	-4.120526	-0.133939	3.666839
H	-2.778490	1.917018	3.512284
H	-1.010390	3.225133	2.443081
H	1.005834	1.998649	-0.319640
H	1.846895	2.809375	1.943596
H	0.787683	4.196548	1.620514
H	2.093960	3.824515	0.503665
H	-0.918309	3.487877	-0.725727
H	-1.575209	3.986625	-2.182222
H	-2.069601	2.507120	-1.450598
H	-3.773729	4.284545	-1.204366
H	-3.091391	3.768921	0.351396
H	-2.576718	5.314896	-0.376508
C	2.336613	-2.273252	-3.515880
H	1.682371	-3.069168	-3.866094
H	2.655778	-1.667581	-4.367084
H	3.195767	-2.738321	-3.047551

(*aR*)-**G**-CH_3COO^-

Geometry optimization in gas phase, B3LYP/6-311G**

```
 SCF Done:  E(RB3LYP) =  -1532.538895
 Zero-point correction=                           0.495202 (Hartree/Particle)
 Thermal correction to Energy=                    0.526283
 Thermal correction to Enthalpy=                  0.527227
 Thermal correction to Gibbs Free Energy=         0.431716
 Sum of electronic and zero-point Energies=          -1532.043693
 Sum of electronic and thermal Energies=             -1532.012612
 Sum of electronic and thermal Enthalpies=           -1532.011667
 Sum of electronic and thermal Free Energies=        -1532.107178
```

Energy in DMA:

SCF Done: E(RM06L) = -1532.54060804

Energy in NMP:

SCF Done: E(RM06L) = -1532.54043321

Cartesian coordinates:

N	-0.165879	2.458335	2.467703
C	1.025177	0.520443	1.611582
C	2.139251	-0.414550	1.807338

C	0.910456	1.604935	2.524028
C	-1.064729	1.285950	0.549607
C	0.062730	0.464741	0.527958
C	-1.268490	2.194185	1.658751
C	-0.548549	-1.549460	-0.829577
C	4.236020	-0.986239	2.998980
H	4.967330	-0.714683	3.752928
C	0.276115	-0.445495	-0.642201
C	-3.389642	1.850232	-0.165169
C	3.132823	-0.124937	2.795823
C	3.381203	-2.472540	1.337534
H	3.453145	-3.396759	0.775469
C	0.574846	-2.058659	-2.926215
H	0.684008	-2.681367	-3.808321
C	4.372732	-2.145980	2.275925
H	5.218574	-2.804118	2.436284
C	-2.483677	2.814042	1.877721
C	-3.542182	2.593983	0.970141
H	-4.501306	3.057374	1.179462
C	-2.132528	1.244896	-0.504874
C	1.908270	1.837342	3.516724
H	1.801459	2.651637	4.215430
C	2.985948	1.017687	3.622701
H	3.735656	1.210585	4.382605
C	1.429050	-0.940998	-2.766932
C	3.049441	1.326653	-2.456912
H	3.670861	2.208709	-2.347189
C	2.304144	-1.639927	1.112256
H	1.575672	-1.949936	0.386584
C	3.210548	0.495243	-3.587519
H	3.955811	0.738329	-4.336398
C	2.412207	-0.611761	-3.734588
H	2.515036	-1.252578	-4.604494
C	2.111791	1.031028	-1.497068
H	2.003475	1.688451	-0.644010
C	1.275628	-0.114621	-1.607352
C	-0.378847	-2.351541	-1.987920
H	-1.046375	-3.194098	-2.124598
N	-1.605429	-1.834263	0.059518
C	-1.321958	-2.446466	1.352672
H	-0.827123	-3.428532	1.283462
H	-0.689981	-1.796955	1.962684
H	-2.265002	-2.578340	1.887672
H	-4.195298	1.732766	-0.878904
H	-2.651657	3.437318	2.742570
O	-1.949958	0.707471	-1.615110
H	-2.365528	-2.286785	-0.437420
O	-3.725847	-2.283669	-2.035453
C	-4.294293	-1.526590	-2.798894
C	-5.472676	-1.945199	-3.650282
O	-3.971453	-0.255630	-2.999953
H	-3.177457	0.043041	-2.458061
H	-5.210219	-1.852204	-4.706988
H	-5.745403	-2.974072	-3.425041
H	-6.320058	-1.280730	-3.467697
C	-0.191204	3.675266	3.285983
H	-0.844720	4.397187	2.804108
H	-0.556476	3.481989	4.299227
H	0.805133	4.108052	3.336762

TS11

Geometry optimization in gas phase, B3LYP/6-311G**

```
SCF Done:  E(RB3LYP) =  -1532.538895
Zero-point correction=                           0.491862 (Hartree/Particle)
Thermal correction to Energy=                    0.521371
Thermal correction to Enthalpy=                  0.522315
Thermal correction to Gibbs Free Energy=         0.432614
Sum of electronic and zero-point Energies=          -1532.014613
```

Sum of electronic and thermal Energies= -1531.985104
Sum of electronic and thermal Enthalpies= -1531.984160
Sum of electronic and thermal Free Energies= -1532.073861

Energy in DMA:

SCF Done: E(RM06L) = -1532.50459210

Energy in NMP:

SCF Done: E(RM06L) = -1532.50443401

Cartesian coordinates:

O	1.653903	4.229821	7.653093
C	3.062912	2.449392	6.821216
C	4.258284	1.678971	7.076837
C	2.846510	3.567968	7.626122
C	0.780827	2.689215	6.032674
C	2.018937	2.154605	5.824088
C	0.548496	3.612520	7.108706
C	1.136698	0.451990	4.282724
C	6.446640	1.479903	8.197145
H	7.197159	1.915906	8.848150
C	2.189724	1.285577	4.628394
C	-1.725030	2.449508	5.879720
C	5.260687	2.215513	7.950255
C	5.625622	-0.327943	6.846118
H	5.751732	-1.326932	6.444301
C	2.345759	-0.501341	2.439221
H	2.414736	-1.197278	1.610512
C	6.635584	0.233683	7.652703
H	7.542264	-0.324039	7.856414
C	-0.690722	3.908367	7.562988
C	-1.833307	3.263451	6.945938
H	-2.813899	3.476273	7.358936
C	-0.407746	2.309711	5.161512
C	3.838443	4.105664	8.471145
H	3.611073	5.017235	9.009155
C	5.034871	3.458153	8.599461
H	5.808485	3.862569	9.242774
C	3.410961	0.401903	2.669146
C	5.423076	2.323631	3.014039
H	6.188614	3.083094	3.124244
C	4.475625	0.373210	6.564416
H	3.717342	-0.094554	5.955004
C	5.529652	1.368980	1.978078
H	6.382334	1.391028	1.309159
C	4.540867	0.434032	1.809544
H	4.594507	-0.285656	0.999583
C	4.357068	2.303899	3.879399
H	4.290879	3.052628	4.655963
C	3.327554	1.332548	3.756652
C	1.211341	-0.453712	3.209862
H	0.352432	-1.073776	2.983074
N	-0.130311	0.590720	4.985773
C	-0.265375	-0.243541	6.203408
H	-0.198296	-1.299870	5.935836
H	0.523912	0.004628	6.912262
H	-1.234535	-0.040994	6.657615
H	-2.598317	2.039741	5.387345
H	-0.820867	4.612989	8.373392
O	-0.380350	2.788187	3.946796
H	-0.903018	0.356740	4.308856
O	-2.109681	0.100696	3.183897
C	-2.557019	0.991027	2.436896
C	-3.580800	0.643429	1.376107
O	-2.220270	2.232499	2.473396
H	-1.366555	2.491799	3.210579
H	-3.117489	0.739234	0.390346
H	-3.940678	-0.374949	1.511594
H	-4.410455	1.351665	1.413227

Structure 7

Geometry optimization in gas phase, B3LYP/6-311G**

```
 SCF Done:  E(RB3LYP) =  -1532.523151
 Zero-point correction=                           0.496152 (Hartree/Particle)
 Thermal correction to Energy=                    0.526563
 Thermal correction to Enthalpy=                  0.527507
 Thermal correction to Gibbs Free Energy=         0.434350
 Sum of electronic and zero-point Energies=          -1532.028204
 Sum of electronic and thermal Energies=             -1531.997792
 Sum of electronic and thermal Enthalpies=           -1531.996848
 Sum of electronic and thermal Free Energies=        -1532.090005
```

Energy in DMA:

```
 SCF Done:  E(RM06L) =  -1532.51637551
```

Energy in NMP:

```
 SCF Done:  E(RM06L) =  -1532.51625197
```

Cartesian coordinates:

```
 N    -0.161959     2.477482     2.378744
 C     1.243794     0.691983     1.536064
 C     2.407438    -0.133017     1.757269
 C     1.062113     1.823997     2.355874
 C    -1.070984     0.885840     0.829869
 C     0.181993     0.406075     0.565862
 C    -1.307024     1.834843     1.891555
 C    -0.738438    -1.188165    -1.052191
 C     4.621013    -0.439677     2.805517
 H     5.410976    -0.038870     3.432399
 C     0.343175    -0.402935    -0.665065
 C    -3.559681     0.609886     0.644885
 C     3.462117     0.348082     2.596045
 C     3.669287    -2.205009     1.486647
 H     3.731888    -3.210030     1.084618
 C     0.544347    -2.229313    -2.810086
 H     0.636524    -2.955538    -3.610587
 C     4.733978    -1.692678     2.255759
 H     5.619770    -2.292968     2.428406
 C    -2.577323     2.115724     2.312096
 C    -3.692270     1.442411     1.692330
 H    -4.681873     1.635597     2.094514
 C    -2.229388     0.431582    -0.041973
 C     2.128414     2.286981     3.178095
 H     2.004978     3.181328     3.771119
 C     3.300506     1.590979     3.257444
 H     4.104361     1.959174     3.886001
 C     1.611558    -1.325338    -2.576439
 C     3.634124     0.579054    -2.212844
 H     4.404941     1.332027    -2.093212
 C     2.544941    -1.450514     1.243325
 H     1.743179    -1.883876     0.664993
 C     3.757164    -0.391540    -3.230237
 H     4.626718    -0.389920    -3.877673
 C     2.761282    -1.319008    -3.407741
 H     2.827738    -2.053329    -4.203889
 C     2.544186     0.583750    -1.375414
 H     2.466198     1.344225    -0.611258
 C     1.507320    -0.376562    -1.509115
 C    -0.617175    -2.138474    -2.089025
 H    -1.467653    -2.764703    -2.330580
 N    -2.019004    -1.015447    -0.417461
 C    -2.250351    -1.997354     0.667389
 H    -2.172514    -3.002142     0.248940
 H    -1.521317    -1.895702     1.479368
 H    -3.250243    -1.861026     1.077106
 H    -4.423211     0.166520     0.163429
 H    -2.766847     2.810937     3.117179
 O    -2.176991     1.191340    -1.233164
 H    -3.272295    -1.450574    -1.594148
 O    -3.951819    -1.859495    -2.216467
 C    -4.578370    -0.919265    -2.921931
```

C	-5.570065	-1.496996	-3.899073
O	-4.376959	0.273212	-2.792114
H	-2.968288	0.981456	-1.766223
H	-5.044835	-2.120726	-4.626471
H	-6.279502	-2.138831	-3.372359
H	-6.094967	-0.693186	-4.410132
C	-0.324713	3.747139	3.074940
H	-1.186722	4.260538	2.651323
H	-0.486295	3.618576	4.152084
H	0.552884	4.373115	2.918955

Structure 8 (TS)

Geometry optimization in gas phase, B3LYP/6-311G**

SCF Done: E(RB3LYP) = -1532.51559782
Zero-point correction= 0.492838 (Hartree/Particle)
Thermal correction to Energy= 0.522863
Thermal correction to Enthalpy= 0.523808
Thermal correction to Gibbs Free Energy= 0.431878
Sum of electronic and zero-point Energies= -1532.022760
Sum of electronic and thermal Energies= -1531.992734
Sum of electronic and thermal Enthalpies= -1531.991790
Sum of electronic and thermal Free Energies= -1532.083720

Energy in DMA:

SCF Done: E(RM06L) = -1532.51814383

Energy in NMP:

SCF Done: E(RM06L) = -1532.51796384

Cartesian coordinates:

N	-0.132101	2.531835	2.139374
C	1.352200	0.713745	1.538579
C	2.578088	0.006111	1.839483
C	1.127682	1.956639	2.166038
C	-1.012838	0.606383	0.994958
C	0.279818	0.192192	0.696468
C	-1.249192	1.756708	1.822407
C	-0.667834	-1.602738	-0.649723
C	4.839184	0.012769	2.824837
H	5.622026	0.559405	3.340246
C	0.424378	-0.742319	-0.400814
C	-3.460256	0.212023	0.939260
C	3.625870	0.683363	2.536008
C	3.959791	-2.003397	1.864595
H	4.073346	-3.056656	1.634250
C	0.627376	-2.841960	-2.277289
H	0.716395	-3.663374	-2.980278
C	5.013568	-1.307622	2.489069
H	5.941455	-1.817419	2.720744
C	-2.532216	2.092206	2.199627
C	-3.616833	1.285542	1.763656
H	-4.616085	1.553233	2.089679
C	-2.152313	-0.101536	0.424378
C	2.192709	2.615914	2.845433
H	2.033998	3.589167	3.285816
C	3.407416	2.011471	2.985179
H	4.208236	2.526769	3.504662
C	1.683883	-1.893256	-2.198375
C	3.645810	0.092608	-2.185151
H	4.389122	0.881708	-2.202061
C	2.781690	-1.366745	1.545765
H	1.990885	-1.935197	1.080379
C	3.776877	-1.008767	-3.052168
H	4.626174	-1.076329	-3.722127
C	2.802886	-1.979062	-3.059977
H	2.865080	-2.817038	-3.746428
C	2.577140	0.187502	-1.321197
H	2.494164	1.055561	-0.683331
C	1.574516	-0.811847	-1.274845
C	-0.518492	-2.693121	-1.555021

H	-1.342485	-3.371549	-1.718445
N	-1.868767	-1.392117	-0.005969
C	-2.916248	-2.420889	0.012733
H	-3.606614	-2.310451	-0.826245
H	-2.447871	-3.402583	0.015193
H	-3.478085	-2.319622	0.939503
H	-4.312711	-0.312007	0.533759
H	-2.727136	2.937745	2.842945
O	-2.233202	0.793596	-1.348696
H	-3.139590	0.402288	-2.335752
O	-3.806017	0.226519	-3.170363
C	-4.866262	-0.475598	-2.868619
C	-5.832935	-0.608103	-4.033178
O	-5.093673	-0.996153	-1.781837
H	-2.514712	1.680995	-1.098476
H	-6.153662	0.383591	-4.361359
H	-5.326625	-1.078618	-4.879687
H	-6.697118	-1.201235	-3.738893
C	-0.340297	3.907685	2.580165
H	-1.230039	4.296661	2.087986
H	-0.475400	3.983083	3.665082
H	0.505671	4.523933	2.281317

(*M*)-3

Geometry optimization in gas phase, B3LYP/6-311G**

SCF Done: E(RB3LYP) = -1532.54392901

Zero-point correction= 0.495076 (Hartree/Particle)

Thermal correction to Energy= 0.526390

Thermal correction to Enthalpy= 0.527335

Thermal correction to Gibbs Free Energy= 0.430892

Sum of electronic and zero-point Energies= -1532.048853

Sum of electronic and thermal Energies= -1532.017539

Sum of electronic and thermal Enthalpies= -1532.016594

Sum of electronic and thermal Free Energies= -1532.113037

Energy in DMA:

SCF Done: E(RM06L) = -1532.55746332

Energy in NMP:

SCF Done: E(RM06L) = -1532.55719796

Cartesian coordinates:

N	-0.142023	2.627327	2.245157
C	1.320185	0.807149	1.584141
C	2.498657	0.022524	1.895923
C	1.082304	1.988298	2.322797
C	-1.012740	0.827441	0.901679
C	0.310085	0.413347	0.616441
C	-1.253191	1.942661	1.757146
C	-0.626732	-1.100356	-1.052906
C	4.659898	-0.170344	3.064312
H	5.407341	0.280576	3.708327
C	0.492002	-0.341767	-0.582296
C	-3.431844	0.578073	0.570227
C	3.497605	0.574288	2.752975
C	3.818598	-2.022543	1.790381
H	3.925638	-3.042868	1.440672
C	0.731444	-2.115310	-2.771079
H	0.825966	-2.794925	-3.611277
C	4.827349	-1.447874	2.586057
H	5.715713	-2.016651	2.833797
C	-2.559754	2.344489	2.011359
C	-3.621708	1.662525	1.408130
H	-4.633143	2.006723	1.590174
C	-2.120109	0.151302	0.315140
C	2.105807	2.523478	3.158000
H	1.941886	3.448260	3.690795
C	3.279484	1.852551	3.332516
H	4.045994	2.271446	3.975566
C	1.840258	-1.276673	-2.444873
C	3.896989	0.550194	-1.980504

H	4.678859	1.283049	-1.817141
C	2.690697	-1.308064	1.450356
H	1.933870	-1.786494	0.847216
C	4.037367	-0.406945	-2.997999
H	4.931550	-0.425670	-3.609959
C	3.013589	-1.299303	-3.229414
H	3.081800	-2.018899	-4.038025
C	2.766996	0.575930	-1.187587
H	2.684461	1.338428	-0.427036
C	1.717686	-0.352337	-1.370703
C	-0.464404	-2.023230	-2.133325
H	-1.314405	-2.575495	-2.522415
N	-1.855876	-0.956049	-0.467297
C	-2.939022	-1.956664	-0.604062
H	-3.819032	-1.509580	-1.094775
H	-2.607815	-2.797020	-1.197535
H	-3.203047	-2.281171	0.405885
H	-4.267762	0.133454	0.039874
H	-2.768307	3.174669	2.670721
O	-2.985834	-3.137029	-3.534827
H	-3.570155	-2.326473	-3.594116
O	-4.600693	-1.040144	-3.762480
C	-5.313788	-0.400661	-2.948413
C	-6.317268	0.601470	-3.542431
O	-5.293017	-0.483286	-1.689723
H	-3.602684	-3.870003	-3.608498
H	-6.036750	1.615879	-3.242123
H	-6.340270	0.539206	-4.630660
H	-7.312953	0.409603	-3.133974
C	-0.320646	3.981238	2.769447
H	-1.124575	4.465196	2.218364
H	-0.568370	3.983895	3.836101
H	0.586948	4.559443	2.609929

Opening of (*M*)-**2**, attack on the *Si* face.

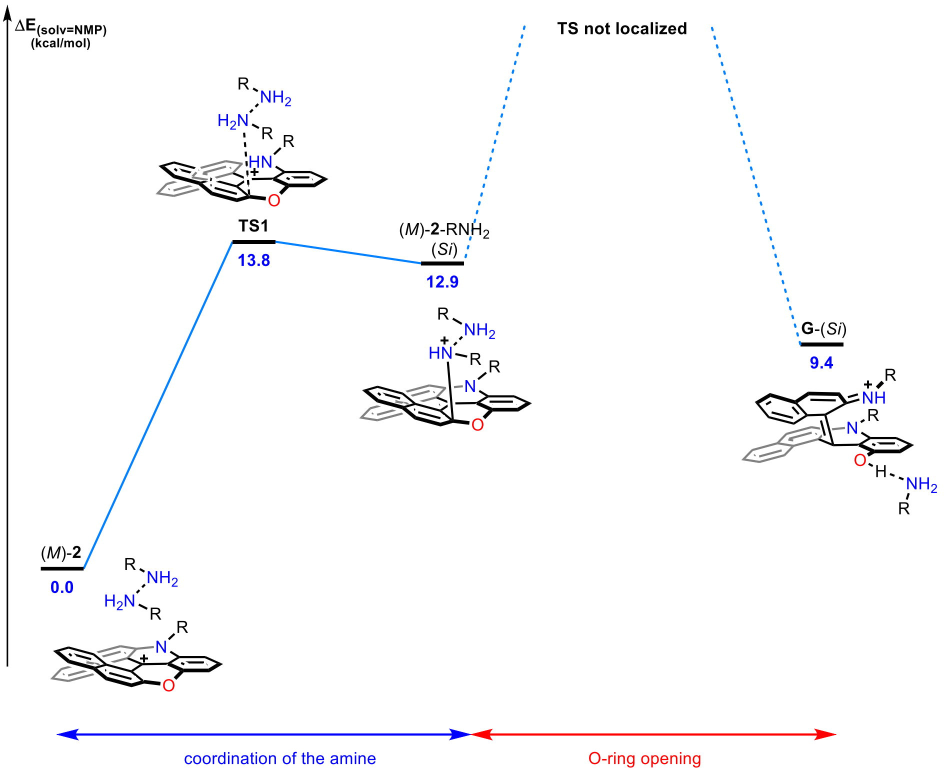

(*M*)-**2**

Geometry optimization in gas phase, B3LYP/6-311G**

```
 SCF Done:  E(RB3LYP) =  -1399.69440372
 Zero-point correction=                           0.510800 (Hartree/Particle)
 Thermal correction to Energy=                    0.541769
 Thermal correction to Enthalpy=                  0.542713
 Thermal correction to Gibbs Free Energy=         0.445005
 Sum of electronic and zero-point Energies=          -1399.183603
 Sum of electronic and thermal Energies=             -1399.152635
 Sum of electronic and thermal Enthalpies=           -1399.151691
 Sum of electronic and thermal Free Energies=        -1399.249399
```

Energy in DMA:

```
 SCF Done:  E(RM06L) =  -1399.72477048
```

Energy in NMP:

```
 SCF Done:  E(RM06L) =  -1399.72449456
```

Cartesian coordinates:

```
 C      4.637601    -0.950720     2.343538
 C      3.799692    -1.004651     1.207337
 C      2.637126    -0.184811     1.131930
 C      2.418019     0.730995     2.183939
 C      3.266580     0.787512     3.271720
 C      4.372234    -0.073294     3.370515
 C      1.790792    -0.259925    -0.050172
 C      2.293304    -0.941563    -1.197690
 C      3.449181    -1.766555    -1.077948
 C      4.144352    -1.818368     0.092634
 C      0.498631     0.350701    -0.168553
 C      0.109144     0.732742    -1.478699
 C      0.728673     0.174217    -2.629314
 N      1.669377    -0.822737    -2.420661
 C     -0.894312     1.709141    -1.663343
 O     -1.458094     2.291123    -0.578486
 C     -1.361476     1.684921     0.626633
 C     -0.453798     0.644172     0.879676
 C     -0.610400    -0.076230     2.131084
 C     -1.492405     0.451443     3.124357
```

C	-2.239356	1.633463	2.850255
C	-2.204844	2.222410	1.620228
C	0.348548	0.616193	-3.901325
C	-0.620046	1.606992	-4.026163
C	-1.250216	2.166998	-2.917920
C	-1.663460	-0.231002	4.351024
C	-1.028348	-1.428519	4.587007
C	-0.211086	-1.984544	3.587783
C	-0.001857	-1.323738	2.394517
N	-3.067114	-0.484005	-0.920737
C	-2.712871	-1.731623	-1.607335
N	-5.745829	0.514503	-2.332065
C	-5.777434	1.682634	-3.226620
H	1.588940	1.421119	2.135790
H	3.077430	1.511237	4.055688
H	5.022492	-0.027008	4.235493
H	5.509912	-1.593659	2.379401
H	5.025386	-2.446982	0.160780
H	3.808849	-2.326867	-1.926920
H	0.827466	0.232573	-4.789819
H	-0.883570	1.957549	-5.016732
H	-2.002834	2.937437	-3.013146
H	0.621343	-1.788514	1.644913
H	0.259258	-2.946780	3.752850
H	-1.172274	-1.950764	5.525002
H	-2.326784	0.196064	5.094838
H	-2.882757	2.034745	3.625243
H	-2.815503	3.079108	1.367622
H	-3.368972	-0.699627	0.025506
H	-1.847477	-2.193845	-1.121827
H	-2.427824	-1.507342	-2.638851
H	-3.512837	-2.485671	-1.641439
H	-3.877401	-0.061301	-1.384880
H	-6.051041	-0.313713	-2.835151
H	-6.412924	0.640817	-1.576244
H	-6.759207	1.891868	-3.670690
H	-5.462015	2.569216	-2.671862
H	-5.064089	1.531801	-4.040038
C	2.049016	-1.689615	-3.546111
H	2.245915	-2.694840	-3.181850
H	1.214259	-1.747358	-4.238995
H	2.930178	-1.309345	-4.069369

TS1

Geometry optimization in gas phase, B3LYP/6-311G**

SCF Done: E(RB3LYP) = -1399.67420835
Zero-point correction= 0.512524 (Hartree/Particle)
Thermal correction to Energy= 0.541223
Thermal correction to Enthalpy= 0.542167
Thermal correction to Gibbs Free Energy= 0.453447
Sum of electronic and zero-point Energies= -1399.161684
Sum of electronic and thermal Energies= -1399.132985
Sum of electronic and thermal Enthalpies= -1399.132041
Sum of electronic and thermal Free Energies= -1399.220761

Energy in DMA:

SCF Done: E(RM06L) = -1399.70283372

Energy in NMP:

SCF Done: E(RM06L) = -1399.70253089

Cartesian coordinates:

C	12.884531	15.080848	1.151675
C	12.193925	14.878750	-0.067055
C	10.919959	15.486512	-0.281113
C	10.441512	16.358890	0.726555
C	11.150574	16.568232	1.889333
C	12.370487	15.905858	2.123075
C	10.227851	15.258147	-1.533108
C	10.944858	14.669399	-2.599440
C	12.209458	14.063079	-2.354110

C	12.785089	14.132424	-1.119231
C	8.850913	15.664412	-1.794152
C	8.597714	16.039494	-3.168638
C	9.416407	15.567440	-4.218999
N	10.440288	14.686801	-3.896508
C	7.501150	16.849232	-3.487365
O	6.674202	17.275304	-2.473628
C	6.596337	16.518699	-1.324893
C	7.784652	15.747856	-0.903134
C	7.656919	15.119425	0.416941
C	6.776046	15.693037	1.375152
C	6.044886	16.897035	1.023761
C	5.962118	17.314707	-0.250215
C	9.148198	15.977236	-5.535690
C	8.083524	16.829577	-5.795695
C	7.237030	17.270873	-4.777838
C	6.633191	15.103773	2.640812
C	7.317033	13.942316	2.963568
C	8.160986	13.356655	2.015830
C	8.332967	13.938513	0.767954
N	5.302715	15.316855	-1.704223
C	5.632430	14.230810	-2.651508
N	2.895428	16.739429	-2.582910
C	3.089397	17.727938	-3.666076
H	9.519188	16.898921	0.573290
H	10.765098	17.258857	2.630372
H	12.908920	16.067591	3.049047
H	13.843467	14.594204	1.291943
H	13.748263	13.663514	-0.950834
H	12.741264	13.570916	-3.154154
H	9.786535	15.667613	-6.350198
H	7.907049	17.156829	-6.813117
H	6.400052	17.927508	-4.973839
H	8.993376	13.467871	0.051659
H	8.688942	12.440884	2.254476
H	7.195394	13.490539	3.940518
H	5.970748	15.565752	3.364952
H	5.554872	17.454859	1.814723
H	5.431596	18.209085	-0.551001
H	5.086656	14.926535	-0.788387
H	6.587362	13.785378	-2.372979
H	5.701086	14.637806	-3.658447
H	4.859310	13.459771	-2.627402
H	4.462642	15.861472	-2.025022
H	2.192011	16.061005	-2.864939
H	2.508708	17.205012	-1.765308
H	2.177021	18.272080	-3.930811
H	3.848451	18.449830	-3.361239
H	3.453561	17.215080	-4.557977
C	11.075580	13.902272	-4.958243
H	11.379619	12.934354	-4.564955
H	10.348927	13.721314	-5.746799
H	11.947741	14.408659	-5.383360

(*M*)-**2**-RNH_2

Geometry optimization in gas phase, B3LYP/6-311G**

SCF Done: E(RB3LYP) = -1399.67466242
Zero-point correction= 0.513485 (Hartree/Particle)
Thermal correction to Energy= 0.542459
Thermal correction to Enthalpy= 0.543404
Thermal correction to Gibbs Free Energy= 0.453911
Sum of electronic and zero-point Energies= -1399.161177
Sum of electronic and thermal Energies= -1399.132203
Sum of electronic and thermal Enthalpies= -1399.131259
Sum of electronic and thermal Free Energies= -1399.220752

Energy in DMA:

SCF Done: E(RM06L) = -1399.70426834

Energy in NMP:

SCF Done: E(RM06L) = -1399.70394326

Cartesian coordinates:

C	4.373212	-0.493624	2.541727
C	3.708621	-0.705910	1.309821
C	2.430715	-0.114929	1.071214
C	1.923985	0.754739	2.068275
C	2.608160	0.974485	3.243435
C	3.831114	0.326018	3.501847
C	1.763641	-0.356072	-0.190807
C	2.505104	-0.937476	-1.241873
C	3.775084	-1.522759	-0.975028
C	4.328813	-1.444491	0.269641
C	0.382663	0.032299	-0.475737
C	0.150654	0.406487	-1.858954
C	0.993158	-0.060851	-2.891071
N	2.020553	-0.930781	-2.548638
C	-0.951061	1.197134	-2.200474
O	-1.813252	1.609941	-1.206928
C	-1.913918	0.843510	-0.045638
C	-0.692876	0.092789	0.392628
C	-0.825202	-0.516052	1.723222
C	-1.681153	0.093273	2.681228
C	-2.362572	1.328807	2.320750
C	-2.459525	1.719880	1.042011
C	0.747057	0.343687	-4.214674
C	-0.323052	1.180517	-4.498047
C	-1.196820	1.609867	-3.497660
C	-1.831850	-0.477870	3.952826
C	-1.175662	-1.654435	4.283788
C	-0.350863	-2.270316	3.339378
C	-0.174083	-1.707367	2.082530
N	-3.124722	-0.228754	-0.338946
C	-2.869396	-1.289594	-1.348865
N	-5.522832	1.186487	-1.035188
C	-5.398179	2.071300	-2.216307
H	0.999636	1.284951	1.894700
H	2.201325	1.662666	3.975354
H	4.349807	0.495338	4.437725
H	5.335272	-0.968229	2.700954
H	5.295623	-1.899240	0.455265
H	4.327540	-2.007858	-1.765415
H	1.406329	0.041140	-5.015026
H	-0.483513	1.504257	-5.519270
H	-2.040254	2.252383	-3.712850
H	0.472600	-2.198192	1.366706
H	0.158450	-3.194321	3.586639
H	-1.302540	-2.091822	5.266614
H	-2.473708	0.010491	4.678300
H	-2.790282	1.934671	3.112648
H	-2.930954	2.643596	0.731011
H	-3.299252	-0.661483	0.569329
H	-1.932438	-1.792612	-1.116186
H	-2.812663	-0.838658	-2.336718
H	-3.687406	-2.010352	-1.323263
H	-3.997158	0.323757	-0.596422
H	-6.290038	0.533888	-1.178922
H	-5.788104	1.740440	-0.224000
H	-6.304284	2.649066	-2.421863
H	-4.569875	2.763090	-2.058702
H	-5.169141	1.466136	-3.094956
C	2.687699	-1.705334	-3.597110
H	2.996219	-2.669781	-3.198402
H	1.980418	-1.895396	-4.401044
H	3.562288	-1.188120	-4.004360

G^{Si}

Geometry optimization in gas phase, B3LYP/6-311G**

SCF Done: E(RB3LYP) = -1399.67717047

```
 Zero-point correction=                           0.511792 (Hartree/Particle)
 Thermal correction to Energy=                    0.541336
 Thermal correction to Enthalpy=                  0.542281
 Thermal correction to Gibbs Free Energy=         0.451804
 Sum of electronic and zero-point Energies=          -1399.165378
 Sum of electronic and thermal Energies=             -1399.135834
 Sum of electronic and thermal Enthalpies=           -1399.134890
 Sum of electronic and thermal Free Energies=        -1399.225367
```

Energy in DMA:

```
 SCF Done:  E(RM06L) =  -1399.70972623
```

Energy in NMP:

```
 SCF Done:  E(RM06L) =  -1399.70941456
```

Cartesian coordinates:

```
 C     3.931393     0.976955     1.927623
 C     3.291695     0.245604     0.897657
 C     1.872769     0.308774     0.761927
 C     1.158663     1.165576     1.634650
 C     1.809496     1.883371     2.612474
 C     3.205697     1.778341     2.776560
 C     1.240515    -0.438107    -0.292351
 C     2.035438    -1.011144    -1.300741
 C     3.444178    -1.101392    -1.122584
 C     4.039610    -0.515461    -0.041223
 C    -0.206766    -0.628720    -0.453860
 C    -0.594542    -0.323361    -1.838915
 C     0.248241    -0.838445    -2.858364
 N     1.431378    -1.466620    -2.469970
 C    -0.096109    -0.682431    -4.210370
 C    -1.211429     0.071206    -4.540491
 C    -1.989272     0.682970    -3.558971
 C    -1.683874     0.508599    -2.207990
 C    -1.073361    -1.062422     0.545164
 C    -2.516034    -1.154785     0.322770
 C    -3.379606    -0.629935     1.346564
 C    -2.918837    -0.515357     2.615738
 C    -1.602199    -0.970488     2.981654
 C    -0.686334    -1.317970     1.954272
 C    -1.256446    -1.152571     4.331096
 C    -0.044383    -1.726865     4.675250
 C     0.828286    -2.137939     3.665114
 C     0.516964    -1.929049     2.326649
 N    -3.084174    -1.756686    -0.714558
 C    -2.480082    -2.658594    -1.688854
 O    -2.393976     1.102865    -1.232160
 N    -3.715154     3.371135    -1.916621
 C    -2.680991     4.417872    -2.077463
 H     0.089308     1.276244     1.511938
 H     1.242722     2.542814     3.259570
 H     3.703329     2.343778     3.555244
 H     5.009974     0.911376     2.020247
 H     5.116038    -0.576267     0.075148
 H     4.055274    -1.594142    -1.865196
 H     0.535132    -1.078760    -4.992246
 H    -1.461750     0.217017    -5.584864
 H    -2.833391     1.302039    -3.835889
 H     1.210933    -2.261020     1.567222
 H     1.759187    -2.629257     3.922403
 H     0.213543    -1.878538     5.716148
 H    -1.965013    -0.865216     5.100197
 H    -3.585481    -0.172567     3.399964
 H    -4.415863    -0.424184     1.102814
 H    -4.091732    -1.676363    -0.764647
 H    -1.515502    -2.998690    -1.317017
 H    -2.341350    -2.171909    -2.654962
 H    -3.143473    -3.517141    -1.808944
 H    -4.331661     3.365333    -2.725041
 H    -4.306719     3.592960    -1.119631
 H    -2.937248     1.881246    -1.575129
```

H	-2.038251	4.419610	-1.196243
H	-2.061240	4.177152	-2.942282
H	-3.095295	5.421605	-2.211952
C	2.174629	-2.287796	-3.424893
H	2.778079	-3.013598	-2.882719
H	1.468325	-2.838452	-4.043465
H	2.826758	-1.692481	-4.073046

N-ring opening of cationic (*aR*)-**G·H**+

From the *Si* face

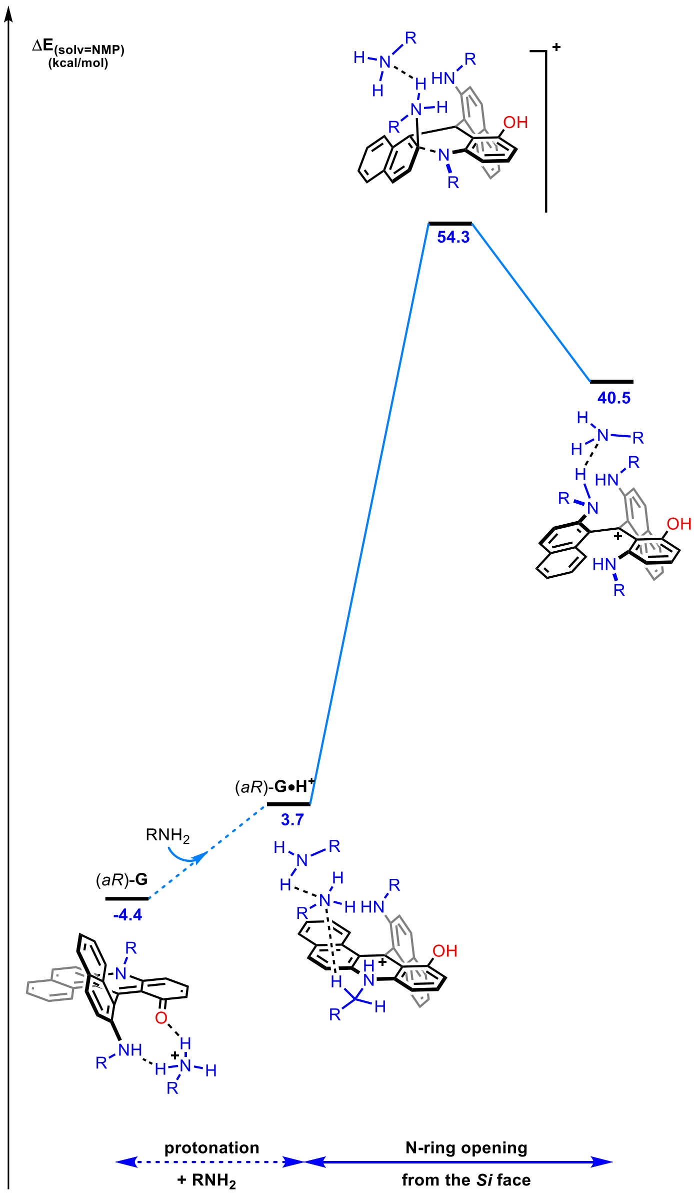

(*aR*)-**G·H**+

Geometry optimization in gas phase, B3LYP/6-311G**

```
 SCF Done:  E(RB3LYP) =  -1495.59054250
 Zero-point correction=                           0.577107 (Hartree/Particle)
 Thermal correction to Energy=                    0.612603
 Thermal correction to Enthalpy=                  0.613547
 Thermal correction to Gibbs Free Energy=         0.501838
 Sum of electronic and zero-point Energies=          -1495.013436
 Sum of electronic and thermal Energies=             -1494.977939
 Sum of electronic and thermal Enthalpies=           -1494.976995
 Sum of electronic and thermal Free Energies=        -1495.088705
```

Energy in DMA:

```
 SCF Done:  E(RM06L) =  -1495.60097034
```

Energy in NMP:

```
 SCF Done:  E(RM06L) =  -1495.60067836
```

Cartesian coordinates:

C	-3.506733	0.703287	-0.149452
C	-3.087926	0.819009	1.202714
C	-4.094741	1.009175	2.200947
C	-5.458163	1.065932	1.821843
C	-5.829866	0.943108	0.505144
C	-4.840114	0.762290	-0.483916
C	-1.709297	0.778855	1.600322
C	-1.343816	0.913164	2.953125
C	-2.372696	1.110910	3.921715
C	-3.689454	1.148838	3.552906
C	-0.653619	0.533151	0.561421
C	-0.409794	-0.769748	0.055165
C	0.456608	-0.881218	-1.090754
C	0.616924	-2.124867	-1.769964
C	-0.062140	-3.224760	-1.359651
C	-0.890778	-3.214059	-0.203783
C	-1.029823	-2.015135	0.558888
C	-1.728908	-2.139468	1.778764
C	-2.304571	-3.334728	2.175576
C	-2.216217	-4.482925	1.381698
C	-1.500221	-4.417651	0.207080
N	4.698741	-1.556850	-2.426540
C	5.449384	-1.223885	-1.206389
N	-0.036535	0.813619	3.353359
C	0.442607	1.142585	4.688012
C	0.106667	1.641706	0.083473
C	1.109884	1.425595	-0.926068
C	2.028324	2.428450	-1.273709
C	1.912344	3.681089	-0.704517
C	0.873656	3.981860	0.171521
C	-0.034845	3.009900	0.557021
N	1.126257	0.205599	-1.572368
O	-1.001440	3.432079	1.378865
N	6.686063	-1.061047	-4.833793
C	6.274147	-0.243039	-5.986927
H	-1.820636	-1.303997	2.444847
H	-2.828844	-3.372278	3.123162
H	-2.681662	-5.408921	1.696179
H	-1.376538	-5.297935	-0.413573
H	0.061923	-4.158702	-1.896733
H	1.302642	-2.206260	-2.596117
H	2.839972	2.226145	-1.953271
H	2.632704	4.449265	-0.959338
H	0.745010	4.977078	0.576215
H	-2.769817	0.569501	-0.931549
H	-5.134756	0.671274	-1.523122
H	-6.874461	0.989064	0.222642
H	-6.205731	1.212585	2.593816
H	-4.450497	1.297444	4.311345
H	-2.105049	1.224210	4.963169
H	0.662592	0.752732	2.632663
H	-1.604591	2.699457	1.597976
H	0.206643	2.174064	4.972443
H	1.525376	1.023590	4.700258
H	0.027464	0.466366	5.440221
H	4.520874	-2.557637	-2.445265
H	5.282213	-1.360412	-3.248036
H	6.413401	-1.741959	-1.109117
H	5.648468	-0.149198	-1.185306
H	4.852903	-1.463283	-0.321801
H	7.520835	-0.664789	-4.411149
H	6.950630	-1.990337	-5.148318
H	7.039025	-0.138391	-6.766876
H	5.384860	-0.684111	-6.442441
H	6.005167	0.757519	-5.641178
C	1.868764	0.086536	-2.852962
H	1.250139	-0.464584	-3.557735
H	2.013857	1.078718	-3.262461

H	2.833574	-0.424583	-2.708528

TS

Geometry optimization in gas phase, B3LYP/6-311G**

SCF Done: E(RB3LYP) = -1495.49163026
Zero-point correction= 0.577859 (Hartree/Particle)
Thermal correction to Energy= 0.610701
Thermal correction to Enthalpy= 0.611645
Thermal correction to Gibbs Free Energy= 0.514266
Sum of electronic and zero-point Energies= -1494.913771
Sum of electronic and thermal Energies= -1494.880929
Sum of electronic and thermal Enthalpies= -1494.879985
Sum of electronic and thermal Free Energies= -1494.977364

Energy in DMA:

SCF Done: E(RM06L) = -1495.52043121

Energy in NMP:

SCF Done: E(RM06L) = -1495.52006545

Cartesian coordinates:

C	5.743866	19.188540	0.222392
C	6.477961	19.084520	1.434221
C	5.837916	19.521100	2.637530
C	4.531195	20.069346	2.581798
C	3.859988	20.169138	1.389196
C	4.473110	19.713978	0.202921
C	7.813482	18.532175	1.498977
C	8.425917	18.384556	2.743511
C	7.767585	18.828115	3.920960
C	6.522482	19.391810	3.871246
C	8.563098	18.189390	0.236321
C	8.916260	16.811970	-0.070121
C	10.136765	16.560013	-0.742630
C	10.448440	15.258385	-1.249597
C	9.563434	14.235271	-1.136606
C	8.322995	14.409109	-0.465855
C	7.983190	15.691705	0.073064
C	6.711695	15.791354	0.691055
C	5.864739	14.706916	0.796293
C	6.220130	13.451100	0.277828
C	7.434816	13.315091	-0.350258
N	11.449192	17.229617	-0.376786
C	11.607013	18.605333	0.193840
N	9.753755	17.842010	2.869917
C	9.787472	16.408249	3.237327
C	8.818401	19.208637	-0.693145
C	9.117115	18.880106	-2.110767
C	8.513845	19.722262	-3.110067
C	8.097718	20.978883	-2.770382
C	8.174606	21.460102	-1.439354
C	8.543431	20.618807	-0.425977
N	9.929029	17.889336	-2.400601
O	8.672059	21.153324	0.811869
N	13.381346	17.192817	-2.495899
C	13.938802	18.540559	-2.763211
H	6.375068	16.728007	1.096955
H	4.905051	14.835869	1.283337
H	5.544275	12.609282	0.365082
H	7.733045	12.362048	-0.773308
H	9.808224	13.259005	-1.540562
H	11.411283	15.098672	-1.721904
H	8.554474	19.425717	-4.148356
H	7.754483	21.652972	-3.547261
H	7.939025	22.488975	-1.201605
H	6.183715	18.838947	-0.701536
H	3.935401	19.776526	-0.735942
H	2.860548	20.586023	1.355760
H	4.069098	20.402415	3.504469
H	6.040863	19.737095	4.779359
H	8.278611	18.717897	4.871790

H	10.223544	18.354209	3.611326
H	8.717327	20.455664	1.478564
H	9.202625	16.181352	4.137710
H	10.824881	16.117997	3.419485
H	9.395240	15.807663	2.418196
H	11.854118	16.591400	0.312610
H	12.091426	17.178387	-1.227207
H	12.670693	18.718387	0.411448
H	11.302375	19.339061	-0.543122
H	11.026316	18.679116	1.109293
H	14.142597	16.548202	-2.293997
H	12.946113	16.840475	-3.345059
H	14.646218	18.556492	-3.597486
H	13.121837	19.227525	-2.987276
H	14.452847	18.902744	-1.871609
C	9.953646	17.333230	-3.745112
H	10.412994	16.342535	-3.703215
H	8.959034	17.217698	-4.188022
H	10.555165	17.959870	-4.414198

Structure 3

Geometry optimization in gas phase, B3LYP/6-311G**

SCF Done: E(RB3LYP) = -1495.51881437

Zero-point correction= 0.577709 (Hartree/Particle)

Thermal correction to Energy= 0.611808

Thermal correction to Enthalpy= 0.612752

Thermal correction to Gibbs Free Energy= 0.511847

Sum of electronic and zero-point Energies= -1494.941105

Sum of electronic and thermal Energies= -1494.907006

Sum of electronic and thermal Enthalpies= -1494.906062

Sum of electronic and thermal Free Energies= -1495.006967

Energy in DMA:

SCF Done: E(RM06L) = -1495.54240859

Energy in NMP:

SCF Done: E(RM06L) = -1495.54204493

Cartesian coordinates:

C	-2.864103	1.281019	0.646336
C	-1.862699	1.095731	1.638475
C	-2.255287	1.302326	3.002216
C	-3.593268	1.657222	3.312919
C	-4.529228	1.817181	2.324146
C	-4.152611	1.628418	0.978721
C	-0.488586	0.725734	1.338777
C	0.405738	0.616347	2.401307
C	-0.001573	0.848232	3.737393
C	-1.294932	1.174667	4.033159
C	-0.069448	0.483438	-0.094585
C	0.409178	-0.904094	-0.454608
C	1.753322	-1.162669	-0.665133
C	2.241183	-2.421563	-1.060926
C	1.374170	-3.465207	-1.245060
C	-0.009661	-3.289955	-1.015763
C	-0.503606	-2.004486	-0.611557
C	-1.901973	-1.875767	-0.406382
C	-2.752293	-2.941478	-0.576971
C	-2.259099	-4.203548	-0.972830
C	-0.914965	-4.369594	-1.187255
N	2.772213	-0.126753	-0.417309
C	3.105056	0.831184	-1.521042
N	1.810024	0.316044	2.178774
C	2.201007	-1.018422	2.701946
C	-0.214188	1.461875	-1.047794
C	-0.285300	1.147495	-2.533273
C	-1.121605	2.037928	-3.335048
C	-1.473121	3.258262	-2.871382
C	-1.083306	3.713324	-1.566518
C	-0.468048	2.877846	-0.694719
N	0.376076	0.147902	-2.997507

O	-0.032433	3.400940	0.481376
N	5.399536	-1.224258	0.238462
C	6.312624	-1.318473	-0.925844
H	-2.311672	-0.926316	-0.103491
H	-3.814419	-2.810659	-0.405736
H	-2.942260	-5.033766	-1.106602
H	-0.520643	-5.331746	-1.494957
H	1.741561	-4.436007	-1.557206
H	3.304345	-2.555876	-1.221161
H	-1.353367	1.751389	-4.350890
H	-2.023439	3.941249	-3.509058
H	-1.265568	4.736276	-1.263362
H	-2.619021	1.157756	-0.397751
H	-4.886393	1.762040	0.192481
H	-5.548388	2.090232	2.569732
H	-3.859282	1.803746	4.353748
H	-1.595541	1.353938	5.059220
H	0.735119	0.772242	4.529833
H	2.348546	1.012348	2.690504
H	0.122231	2.698590	1.124998
H	1.988455	-1.134734	3.769227
H	3.272730	-1.161108	2.545622
H	1.660274	-1.790623	2.156184
H	2.456025	0.358857	0.462451
H	3.678867	-0.599840	-0.156677
H	3.995059	1.382976	-1.216698
H	3.299261	0.270444	-2.431954
H	2.277921	1.510296	-1.686875
H	5.827930	-0.634569	0.948538
H	5.312145	-2.141731	0.669851
H	7.297739	-1.721052	-0.672099
H	5.865888	-1.960715	-1.686839
H	6.447951	-0.326806	-1.359914
C	0.218247	-0.252266	-4.388028
H	0.756522	-1.187054	-4.546722
H	-0.829780	-0.411810	-4.668081
H	0.635961	0.497469	-5.071862

From the *Re* face

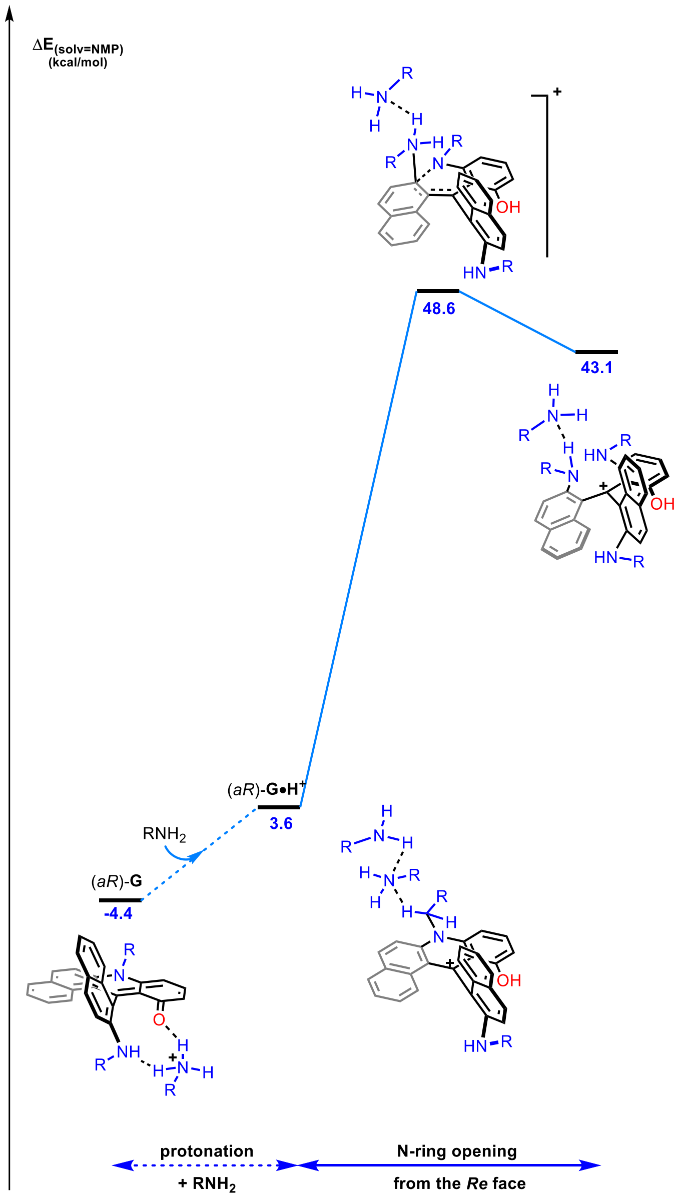

(*a*R)-G·H+

Geometry optimization in gas phase, B3LYP/6-311G**

```
 SCF Done:  E(RB3LYP) =  -1495.59059290
 Zero-point correction=                           0.577097 (Hartree/Particle)
 Thermal correction to Energy=                    0.612581
 Thermal correction to Enthalpy=                  0.613525
 Thermal correction to Gibbs Free Energy=         0.501753
 Sum of electronic and zero-point Energies=          -1495.013495
 Sum of electronic and thermal Energies=             -1494.978012
 Sum of electronic and thermal Enthalpies=           -1494.977067
 Sum of electronic and thermal Free Energies=        -1495.088840
```

Energy in DMA:

```
 SCF Done:  E(RM06L) =  -1495.60108781
```

Energy in NMP:

```
 SCF Done:  E(RM06L) =  -1495.60079281
```

Cartesian coordinates:

```
 C     -2.335426     2.061666     1.409137
 C     -1.493443     1.715102     2.498622
 C     -1.930276     2.053883     3.817062
 C     -3.159437     2.733281     3.995997
 C     -3.946576     3.064220     2.919175
```

C	-3.527263	2.718356	1.617931
C	-0.240990	1.029877	2.330513
C	0.537125	0.664842	3.444123
C	0.072724	1.024592	4.746736
C	-1.108846	1.689451	4.916366
C	0.226219	0.657809	0.953661
C	0.065176	-0.660973	0.454740
C	0.717400	-0.982821	-0.788321
C	0.774813	-2.330221	-1.254858
C	0.235005	-3.335334	-0.521301
C	-0.489147	-3.091709	0.679300
C	-0.655396	-1.752912	1.146038
C	-1.551288	-1.579484	2.223380
C	-2.162887	-2.655743	2.844866
C	-1.922989	-3.969170	2.426662
C	-1.103935	-4.176204	1.338693
N	0.054684	-1.335269	-5.153101
C	-0.917434	-0.295810	-5.525137
N	1.735670	0.016082	3.299974
C	2.546467	-0.471125	4.405536
C	0.843626	1.657284	0.148311
C	1.269051	1.323312	-1.186365
C	1.652757	2.317055	-2.099427
C	1.724428	3.631614	-1.680707
C	1.469399	3.984482	-0.359389
C	1.058709	3.034731	0.561783
N	1.319381	-0.013300	-1.535181
O	0.887605	3.487095	1.806785
N	1.613456	-2.042861	-7.811116
C	3.072047	-2.244649	-7.791350
H	-1.805112	-0.596884	2.572121
H	-2.847419	-2.465803	3.663115
H	-2.397542	-4.803259	2.928881
H	-0.937203	-5.176783	0.955976
H	0.307743	-4.355782	-0.881277
H	1.225931	-2.555940	-2.205917
H	1.862144	2.077348	-3.128933
H	2.002770	4.400184	-2.391879
H	1.577864	5.004246	-0.014511
H	-2.041180	1.805024	0.398762
H	-4.152902	2.971945	0.769862
H	-4.885325	3.584449	3.064709
H	-3.469956	2.987631	5.003594
H	-1.435118	1.949081	5.917668
H	0.667252	0.762989	5.611009
H	1.938387	-0.373463	2.395098
H	0.555586	2.776751	2.383576
H	2.018775	-1.206302	5.024077
H	2.879918	0.350646	5.044631
H	3.434891	-0.947477	3.992339
H	0.626720	-1.573306	-5.971511
H	-0.445181	-2.184889	-4.904613
H	-1.571777	-0.564875	-6.365632
H	-1.555769	-0.055484	-4.670605
H	-0.383657	0.617280	-5.801403
H	1.155262	-2.877221	-8.166659
H	1.377794	-1.299757	-8.462827
H	3.510168	-2.491823	-8.766666
H	3.556216	-1.337388	-7.423437
H	3.314951	-3.052125	-7.097141
C	2.091007	-0.413207	-2.739330
H	2.723211	-1.259242	-2.476647
H	1.418135	-0.682354	-3.568808
H	2.747294	0.400302	-3.020546

TS

Geometry optimization in gas phase, B3LYP/6-311G**

SCF Done: E(RB3LYP) = -1495.49732356

Zero-point correction= 0.577208 (Hartree/Particle)
Thermal correction to Energy= 0.610324
Thermal correction to Enthalpy= 0.611268
Thermal correction to Gibbs Free Energy= 0.513036
Sum of electronic and zero-point Energies= -1494.920116
Sum of electronic and thermal Energies= -1494.887000
Sum of electronic and thermal Enthalpies= -1494.886055
Sum of electronic and thermal Free Energies= -1494.984288

Energy in DMA:

SCF Done: E(RM06L) = -1495.52959094

Energy in NMP:

SCF Done: E(RM06L) = -1495.52921868

Cartesian coordinates:

C	4.823746	18.237802	0.186887
C	5.694256	18.718379	1.203188
C	5.076761	19.423316	2.286859
C	3.677877	19.638030	2.300058
C	2.874469	19.166664	1.290346
C	3.463190	18.450584	0.231299
C	7.131750	18.553726	1.183200
C	7.889306	18.982855	2.305595
C	7.235900	19.674770	3.369147
C	5.890934	19.895406	3.347564
C	7.812194	17.911253	0.013750
C	7.453000	16.564249	-0.404593
C	7.501608	16.238784	-1.782202
C	7.325637	14.891383	-2.230989
C	7.150231	13.879004	-1.342553
C	7.102243	14.126779	0.055420
C	7.262130	15.463101	0.541520
C	7.277991	15.620218	1.948958
C	7.100576	14.550274	2.801781
C	6.910896	13.248567	2.309137
C	6.922264	13.046658	0.949988
N	6.980450	17.150039	-2.884925
C	6.926543	18.646233	-2.856232
N	9.220095	18.707456	2.429948
C	10.079717	19.189517	3.498955
C	8.886708	18.571222	-0.621645
C	9.884166	17.808828	-1.414450
C	11.261163	18.212771	-1.288915
C	11.551865	19.480294	-0.866522
C	10.538978	20.384511	-0.459368
C	9.241755	19.959065	-0.346734
N	9.492675	16.853223	-2.227306
O	8.310870	20.880119	-0.001590
N	8.175156	16.647793	-5.422520
C	8.907637	17.832698	-5.931434
H	7.426569	16.593874	2.380865
H	7.112328	14.722810	3.871797
H	6.769864	12.418832	2.990840
H	6.799839	12.050445	0.539211
H	7.026450	12.862052	-1.698611
H	7.321315	14.690072	-3.296539
H	12.038052	17.586019	-1.703229
H	12.578623	19.827914	-0.895435
H	10.770717	21.414130	-0.220601
H	5.226561	17.645403	-0.620684
H	2.834924	18.050925	-0.557171
H	1.804397	19.331818	1.310892
H	3.249125	20.182375	3.134532
H	5.422168	20.433147	4.164575
H	7.821152	20.026614	4.207025
H	9.674205	18.245104	1.661419
H	7.514256	20.436862	0.326472
H	9.765119	18.809794	4.475661
H	10.112046	20.283397	3.540515
H	11.089527	18.827976	3.309872

H	7.494455	16.894160	-3.789920
H	6.018457	16.829631	-3.022364
H	6.395310	18.945721	-3.761081
H	6.385882	18.998400	-1.984522
H	7.933644	19.044933	-2.868855
H	8.821046	15.865226	-5.350194
H	7.479327	16.361552	-6.107966
H	9.421168	17.646165	-6.878956
H	8.206621	18.655623	-6.078294
H	9.644540	18.142211	-5.189389
C	10.442472	15.865921	-2.717341
H	9.885271	14.988786	-3.056551
H	11.010150	16.258418	-3.569520
H	11.155893	15.531243	-1.957308

Structure 3

Geometry optimization in gas phase, B3LYP/6-311G**

SCF Done: E(RB3LYP) = -1495.50792624

Zero-point correction=	0.577257	(Hartree/Particle)
Thermal correction to Energy=	0.611301	
Thermal correction to Enthalpy=	0.612245	
Thermal correction to Gibbs Free Energy=	0.510585	
Sum of electronic and zero-point Energies=	-1494.930669	
Sum of electronic and thermal Energies=	-1494.896625	
Sum of electronic and thermal Enthalpies=	-1494.895681	
Sum of electronic and thermal Free Energies=	-1494.997342	

Energy in DMA:

SCF Done: E(RM06L) = -1495.53827984

Energy in NMP:

SCF Done: E(RM06L) = -1495.53790284

Cartesian coordinates:

C	-2.738839	0.949980	-0.005211
C	-1.968950	1.174665	1.166582
C	-2.699151	1.631894	2.310812
C	-4.100893	1.814602	2.251562
C	-4.805556	1.567758	1.098625
C	-4.104432	1.132644	-0.039541
C	-0.526757	1.015787	1.234962
C	0.126265	1.316811	2.467715
C	-0.642725	1.770137	3.581575
C	-1.992977	1.917384	3.504950
C	0.272109	0.438559	0.099684
C	-0.189619	-0.895197	-0.423765
C	-0.680408	-1.102454	-1.701583
C	-1.253422	-2.330740	-2.102089
C	-1.318430	-3.388915	-1.239774
C	-0.777903	-3.276563	0.060287
C	-0.202431	-2.031366	0.471393
C	0.393211	-1.984233	1.758995
C	0.381537	-3.078546	2.591833
C	-0.223036	-4.289031	2.193181
C	-0.786689	-4.383696	0.946671
N	-0.730475	-0.127734	-2.836368
C	-0.371018	1.325902	-2.789429
N	1.466048	1.124005	2.671517
C	2.184794	1.477878	3.887461
C	1.432891	1.036492	-0.349101
C	2.513792	0.278367	-1.083340
C	3.881846	0.696792	-0.787940
C	4.121762	1.943080	-0.312764
C	3.059472	2.856867	-0.004374
C	1.766656	2.437822	-0.022631
N	2.197104	-0.646293	-1.926547
O	0.794808	3.352100	0.218428
N	0.718524	-1.139241	-4.956718
C	0.245761	-0.827385	-6.325521
H	0.884955	-1.084672	2.095749
H	0.848671	-3.010748	3.567468

H	-0.228787	-5.139208	2.864682
H	-1.238465	-5.311295	0.613319
H	-1.773253	-4.322061	-1.551161
H	-1.658891	-2.428821	-3.104328
H	4.702745	0.063919	-1.093292
H	5.143814	2.288469	-0.201138
H	3.271835	3.886663	0.251759
H	-2.253558	0.606003	-0.901107
H	-4.645639	0.938965	-0.959220
H	-5.878455	1.709873	1.061914
H	-4.610936	2.161238	3.143776
H	-2.546653	2.266757	4.369562
H	-0.136598	1.997694	4.508471
H	2.040807	0.956447	1.865300
H	-0.016035	2.903421	0.502777
H	1.816765	0.916706	4.750382
H	2.124577	2.548344	4.111847
H	3.233053	1.219028	3.743642
H	-0.129743	-0.556972	-3.648330
H	-1.691365	-0.176210	-3.178986
H	-0.692028	1.747866	-3.742934
H	-0.878449	1.836403	-1.978183
H	0.702699	1.420029	-2.687641
H	1.659099	-0.778224	-4.817989
H	0.796055	-2.146550	-4.837806
H	0.892201	-1.239789	-7.104981
H	-0.758706	-1.230831	-6.463232
H	0.199488	0.254950	-6.454782
C	3.248781	-1.491663	-2.479818
H	2.788829	-2.325517	-3.013782
H	3.887072	-0.943785	-3.186265
H	3.902788	-1.919401	-1.711622

Formation of dibenzoacridinium **5**

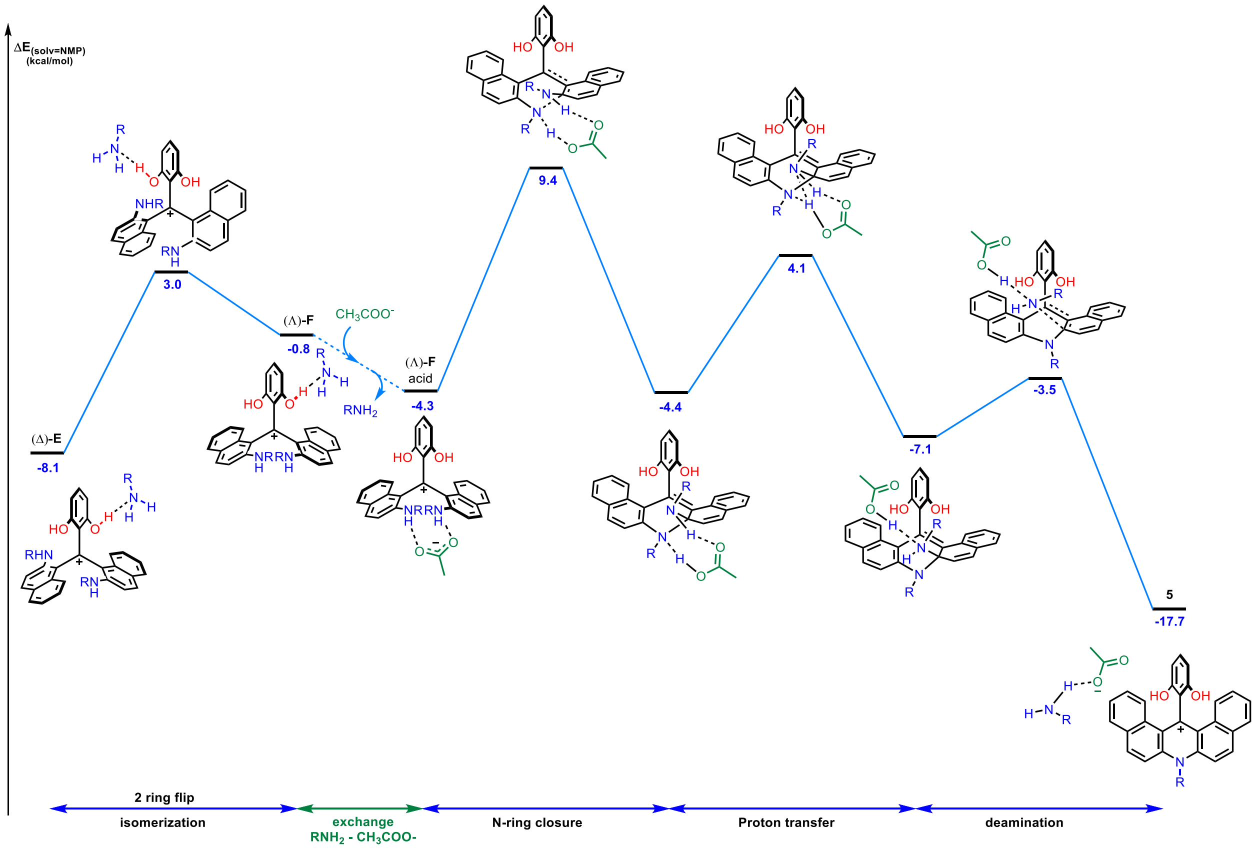

(Δ)-E

Geometry optimization in gas phase, B3LYP/6-311G**

```
 SCF Done:  E(RB3LYP) =  -1476.13724323
 Zero-point correction=                           0.536897 (Hartree/Particle)
 Thermal correction to Energy=                    0.569587
 Thermal correction to Enthalpy=                  0.570531
 Thermal correction to Gibbs Free Energy=         0.471618
 Sum of electronic and zero-point Energies=          -1475.600346
 Sum of electronic and thermal Energies=             -1475.567656
 Sum of electronic and thermal Enthalpies=           -1475.566712
 Sum of electronic and thermal Free Energies=        -1475.665626
```

Energy in DMA:

```
 SCF Done:  E(RM06L) =  -1476.15998666
```

Energy in NMP:

```
 SCF Done:  E(RM06L) =  -1476.15968748
```

Cartesian coordinates:

```
 C    -1.880902     1.555181     1.032867
 C    -1.375610     0.559449     0.175685
 C    -2.229477     0.047230    -0.824647
 C    -3.533331     0.532034    -0.963665
 C    -3.994076     1.526426    -0.109387
 C    -3.178513     2.043086     0.891183
 C     0.016690     0.040697     0.355595
 C     0.949432     0.159094    -0.679250
 C     2.013585    -0.834384    -0.853088
 C     2.432949    -1.148964    -2.186411
 C     2.039131    -0.395376    -3.246603
 C     1.253735     0.788928    -3.084862
 C     0.756764     1.104217    -1.794252
 C     0.156774     2.361449    -1.615515
 C    -0.014173     3.231891    -2.682534
 C     0.409308     2.880625    -3.969831
 C     1.050482     1.671554    -4.163379
 N     2.542589    -1.513263     0.163293
```

```
 C     3.470028    -2.636032     0.056648
 O    -1.753843    -0.974913    -1.574889
 O    -1.009921     2.079637     1.940466
 C     0.259413    -0.635522     1.637631
 C     1.230018    -0.126157     2.584103
 C     1.328308    -0.723331     3.880223
 C     0.458495    -1.799710     4.208133
 C    -0.440212    -2.298107     3.313016
 C    -0.543862    -1.753588     1.995952
 C     2.258343    -0.226015     4.821662
 C     3.083917     0.831094     4.513536
 C     2.983221     1.435581     3.246970
 C     2.076620     0.980455     2.312411
 N    -1.345608    -2.360088     1.084526
 C    -2.289326    -3.424035     1.383774
 H     2.280741    -1.232897     1.097822
 H    -0.147346     2.674759    -0.627291
 H    -0.469290     4.200163    -2.510188
 H     0.266459     3.564461    -4.797675
 H     1.433199     1.402582    -5.141953
 H     2.387272    -0.654285    -4.241165
 H     3.098842    -1.985881    -2.341465
 H    -4.183469     0.111332    -1.719980
 H    -5.004808     1.901290    -0.220191
 H    -3.537186     2.828278     1.548159
 H     1.999821     1.492074     1.362668
 H     3.616204     2.281730     3.004931
 H     3.795269     1.204066     5.240202
 H     2.305700    -0.693296     5.799441
 H     0.525969    -2.236529     5.198822
 H    -1.069230    -3.131826     3.592958
 H    -1.327813    -2.036285     0.125843
 H    -1.463518     2.727006     2.491398
 H    -1.786502    -4.346820     1.692515
 H    -2.997132    -3.133263     2.165983
 H    -2.855829    -3.636094     0.477835
 H    -2.373005    -1.223984    -2.330846
 H     4.407236    -2.331300    -0.415267
 H     3.033987    -3.461652    -0.510938
 H     3.689186    -2.985029     1.063808
 N    -3.215343    -1.716124    -3.748408
 C    -3.073449    -0.705601    -4.819454
 H    -4.199004    -1.918013    -3.588588
 H    -2.798503    -2.594622    -4.046473
 H    -3.527644    -1.009062    -5.767733
 H    -2.013133    -0.510511    -4.984936
 H    -3.535760     0.227690    -4.494739
```

Structure 2 (TS)

Geometry optimization in gas phase, B3LYP/6-311G**

```
 SCF Done:  E(RB3LYP) =  -1476.12294442
 Zero-point correction=                           0.537632 (Hartree/Particle)
 Thermal correction to Energy=                    0.569210
 Thermal correction to Enthalpy=                  0.570154
 Thermal correction to Gibbs Free Energy=         0.474762
 Sum of electronic and zero-point Energies=          -1475.585312
 Sum of electronic and thermal Energies=             -1475.553734
 Sum of electronic and thermal Enthalpies=           -1475.552790
 Sum of electronic and thermal Free Energies=        -1475.648183
```

Energy in DMA:

```
 SCF Done:  E(RM06L) =  -1476.14229436
```

Energy in NMP:

```
 SCF Done:  E(RM06L) =  -1476.14197712
```

Cartesian coordinates:

```
 C    -1.011070     2.557272     0.517390
 C    -0.966426     1.223268     0.061313
 C    -2.167059     0.654145    -0.402918
 C    -3.376793     1.358601    -0.323902
```

C	-3.393298	2.643259	0.202387
C	-2.215796	3.258092	0.615711
C	0.294090	0.386098	0.126708
C	1.257258	0.410767	-0.849139
C	2.376269	-0.564382	-0.801892
C	2.758621	-1.228062	-2.011187
C	2.348664	-0.734061	-3.197703
C	1.598586	0.483477	-3.315665
C	1.137702	1.135722	-2.152968
C	0.573790	2.406580	-2.281056
C	0.423517	2.994508	-3.550135
C	0.816444	2.318047	-4.697044
C	1.414118	1.076546	-4.588152
N	2.979895	-0.816728	0.343419
C	3.999914	-1.832961	0.568714
O	-2.119206	-0.585792	-0.959984
O	0.172631	3.180167	0.816128
C	0.335763	-0.617845	1.272748
C	0.703323	-0.181100	2.591848
C	0.669707	-1.124992	3.678629
C	0.285360	-2.457652	3.409817
C	0.002839	-2.876674	2.132387
C	0.055541	-1.968338	1.029360
C	1.051393	-0.715117	4.980243
C	1.505161	0.563571	5.221080
C	1.583869	1.478438	4.147968
C	1.194778	1.124041	2.874126
N	-0.077918	-2.470486	-0.255315
C	-0.560846	-3.827064	-0.501525
H	2.655016	-0.315327	1.162973
H	0.319139	2.983067	-1.416155
H	0.003333	3.984839	-3.610090
H	0.685456	2.772778	-5.671690
H	1.770935	0.545433	-5.470907
H	2.667460	-1.228537	-4.120629
H	3.415714	-2.082522	-1.957408
H	-4.294174	0.888887	-0.669776
H	-4.331599	3.185383	0.275711
H	-2.220549	4.278033	0.983418
H	1.250873	1.856498	2.086956
H	1.960558	2.485193	4.324885
H	1.799987	0.873099	6.212651
H	0.994695	-1.439167	5.781881
H	0.232829	-3.163522	4.239206
H	-0.255858	-3.911918	1.967667
H	-0.536684	-1.797737	-0.868909
H	-0.004040	4.061204	1.192567
H	0.158471	-4.556940	-0.138288
H	-1.539676	-4.034163	-0.056946
H	-0.637369	-3.960442	-1.588512
H	-3.006839	-0.884538	-1.329432
H	4.871793	-1.666200	-0.075560
H	3.596760	-2.831254	0.374305
H	4.321463	-1.767702	1.611140
N	-4.345920	-1.545352	-2.186788
C	-4.421598	-0.986815	-3.551360
H	-5.224527	-1.405054	-1.691524
H	-4.225702	-2.561983	-2.231918
H	-5.218057	-1.405788	-4.156110
H	-3.465502	-1.161564	-4.062476
H	-4.560564	0.088053	-3.487068

(Λ)-F

Geometry optimization in gas phase, B3LYP/6-311G**

SCF Done: E(RB3LYP) = -1476.12610017
Zero-point correction= 0.536777 (Hartree/Particle)
Thermal correction to Energy= 0.569615
Thermal correction to Enthalpy= 0.570559

Thermal correction to Gibbs Free Energy= 0.470103
Sum of electronic and zero-point Energies= -1475.589323
Sum of electronic and thermal Energies= -1475.556485
Sum of electronic and thermal Enthalpies= -1475.555541
Sum of electronic and thermal Free Energies= -1475.655997

Energy in DMA:

SCF Done: E(RM06L) = -1476.14844670

Energy in NMP:

SCF Done: E(RM06L) = -1476.14814914

Cartesian coordinates:

C	-0.644432	2.603649	0.230505
C	-0.852760	1.231810	-0.042466
C	-2.188949	0.780060	-0.196798
C	-3.258316	1.672966	-0.045139
C	-3.011975	3.003507	0.256391
C	-1.709984	3.480444	0.395420
C	0.278585	0.283466	-0.056043
C	1.352978	0.359092	-0.942543
C	2.599937	-0.354530	-0.616686
C	3.415753	-0.889257	-1.656219
C	3.190020	-0.514075	-2.940734
C	2.181423	0.435066	-3.299698
C	1.283149	0.938560	-2.310365
C	0.339018	1.882367	-2.747785
C	0.283150	2.301795	-4.071967
C	1.160197	1.794577	-5.031535
C	2.100293	0.862314	-4.638362
N	3.011747	-0.438298	0.642613
C	4.192643	-1.153590	1.116140
O	-2.368109	-0.496868	-0.557596
O	0.650152	3.017242	0.362276
C	0.182252	-0.843529	0.932916
C	0.237147	-0.581235	2.342363
C	0.129332	-1.666451	3.271326
C	-0.020822	-2.980164	2.763569
C	-0.063187	-3.223508	1.419340
C	0.024948	-2.164050	0.470187
C	0.208084	-1.418797	4.663382
C	0.419760	-0.150764	5.148716
C	0.577912	0.916893	4.241788
C	0.495185	0.712296	2.881736
N	-0.056247	-2.445728	-0.881310
C	-0.381897	-3.771709	-1.383381
H	2.416611	-0.039042	1.356779
H	-0.360636	2.317083	-2.057558
H	-0.454300	3.044583	-4.353591
H	1.108663	2.128819	-6.060268
H	2.797217	0.448008	-5.358466
H	3.826397	-0.902733	-3.728875
H	4.236778	-1.547935	-1.411868
H	-4.271903	1.319163	-0.181209
H	-3.843701	3.687410	0.378478
H	-1.524725	4.521569	0.636820
H	0.637229	1.555631	2.219327
H	0.768576	1.915216	4.619533
H	0.479845	0.028251	6.215340
H	0.109514	-2.258022	5.343830
H	-0.103674	-3.805331	3.462939
H	-0.190716	-4.238250	1.068400
H	-0.471771	-1.700165	-1.419118
H	0.666888	3.963076	0.547245
H	0.392416	-4.493114	-1.112154
H	-1.348567	-4.152165	-1.024992
H	-0.415070	-3.723295	-2.471994
H	-3.336882	-0.782858	-0.532390
H	5.098203	-0.763596	0.646531
H	4.114598	-2.225540	0.916547
H	4.266090	-1.005034	2.191351

```
 N    -4.899814    -1.477023    -0.548122
 C    -5.564455    -1.378054    -1.865475
 H    -5.495760    -1.081634     0.174810
 H    -4.774585    -2.454127    -0.295345
 H    -6.539343    -1.873911    -1.900201
 H    -4.919975    -1.823802    -2.624459
 H    -5.701517    -0.326054    -2.120793
```

(Λ)-**F**-acid

```
Geometry optimization in gas phase, B3LYP/6-311G**
 SCF Done:  E(RB3LYP) =  -1608.95145089
 Zero-point correction=                           0.519401 (Hartree/Particle)
 Thermal correction to Energy=                    0.553330
 Thermal correction to Enthalpy=                  0.554274
 Thermal correction to Gibbs Free Energy=         0.452845
 Sum of electronic and zero-point Energies=          -1608.432049
 Sum of electronic and thermal Energies=             -1608.398121
 Sum of electronic and thermal Enthalpies=           -1608.397177
 Sum of electronic and thermal Free Energies=        -1608.498606
Energy in DMA:
 SCF Done:  E(RM06L) =  -1608.95183263
Energy in NMP:
 SCF Done:  E(RM06L) =  -1608.95167872
Cartesian coordinates:
 C    -2.603204    -0.777001     4.151566
 C    -2.220831    -0.194745     2.935314
 C    -1.214492    -0.801631     2.144680
 C    -0.672927    -2.017732     2.581718
 C    -1.074988    -2.595156     3.781272
 C    -2.032917    -1.967902     4.579901
 C    -0.802487    -0.152444     0.882622
 C    -1.849676     0.687837     0.224684
 C    -2.681487     1.437043     1.176614
 C    -2.855705     1.010742     2.443131
 C     0.501809    -0.102326     0.455047
 C     0.886577     0.417378    -0.888003
 C     0.612775    -0.291297    -2.069475
 N    -1.966079     0.774871    -1.057523
 C     1.048247     0.261618    -3.322361
 C     1.745171     1.424707    -3.399290
 C     2.045498     2.173831    -2.229483
 C     1.593578     1.671600    -0.969491
 C     2.733977     3.405966    -2.301146
 C     2.961767     4.161250    -1.173357
 C     2.472501     3.703662     0.065165
 C     1.801475     2.503659     0.164096
 C     1.641981    -0.453314     1.369352
 C     2.664820    -1.336676     0.967892
 C     3.762127    -1.623561     1.782415
 C     3.869896    -1.023444     3.029022
 C     2.889446    -0.141335     3.461422
 C     1.795908     0.139244     2.640941
 O     2.524439    -1.952317    -0.241717
 O     0.851594     1.041328     3.037705
 N     0.001880    -1.512682    -2.197315
 C    -0.485193    -2.447896    -1.205044
 C    -2.836981     1.804685    -1.624946
 H    -0.287323    -1.727489    -3.142067
 H     4.518089    -2.321685     1.436835
 H     1.074125     1.360108     3.918307
 H     1.394704     2.212245     1.121406
 H     2.610993     4.310452     0.954031
 H     3.489337     5.106128    -1.238583
 H     3.070691     3.753410    -3.273208
 H     2.072161     1.800238    -4.363846
 H     0.817696    -0.295220    -4.224345
 H    -3.233845     2.294489     0.816330
 H    -3.538186     1.535973     3.104936
```

H	4.720764	-1.241243	3.663817
H	2.970876	0.343871	4.429002
H	3.326383	-2.445743	-0.441802
H	0.065650	-2.520535	1.972003
H	-0.642967	-3.540002	4.091659
H	-2.341343	-2.415066	5.518094
H	-3.366415	-0.290547	4.750776
H	-3.897505	1.587466	-1.449650
H	-2.617227	2.802933	-1.229928
H	-2.680552	1.832831	-2.703618
H	-0.737140	-3.371004	-1.732155
H	0.285191	-2.676824	-0.469815
H	-1.379344	-2.101436	-0.674361
O	-3.189530	-0.937114	-2.979244
C	-2.893880	-1.566316	-4.120499
O	-1.769999	-1.809277	-4.507460
C	-4.142882	-1.943891	-4.878019
H	-3.874743	-2.492699	-5.777888
H	-4.792182	-2.550936	-4.243443
H	-4.698826	-1.041284	-5.142272
H	-2.399022	-0.625100	-2.475894

Structure 5 (TS)

Geometry optimization in gas phase, B3LYP/6-311G**

SCF Done: E(RB3LYP) = -1608.93169630

Zero-point correction=	0.519353	(Hartree/Particle)
Thermal correction to Energy=	0.552184	
Thermal correction to Enthalpy=	0.553128	
Thermal correction to Gibbs Free Energy=	0.455094	
Sum of electronic and zero-point Energies=	-1608.412343	
Sum of electronic and thermal Energies=	-1608.379513	
Sum of electronic and thermal Enthalpies=	-1608.378568	
Sum of electronic and thermal Free Energies=	-1608.476602	

Energy in DMA:

SCF Done: E(RM06L) = -1608.93001534

Energy in NMP:

SCF Done: E(RM06L) = -1608.92985944

Cartesian coordinates:

C	-1.342548	-1.275407	8.867323
C	-1.005144	-0.514655	7.725527
C	0.231656	-0.743591	7.053089
C	1.041273	-1.802919	7.530513
C	0.682225	-2.545356	8.637417
C	-0.510980	-2.271445	9.329614
C	0.582175	0.052682	5.889833
C	-0.467317	0.784278	5.243885
C	-1.642653	1.085146	6.016376
C	-1.894537	0.469575	7.203033
C	1.952142	0.404932	5.570647
C	2.313554	0.938880	4.331393
C	1.441085	0.599213	3.186428
N	-0.337889	1.209236	3.968161
C	1.459638	1.459669	1.996876
C	2.383825	2.414119	1.849968
C	3.408849	2.642419	2.843581
C	3.410577	1.902600	4.064904
C	4.400362	3.598438	2.574797
C	5.424606	3.851254	3.469857
C	5.451989	3.129259	4.661989
C	4.473394	2.187706	4.949541
C	2.892455	0.403163	6.740276
C	3.963378	-0.496660	6.834565
C	4.831808	-0.496256	7.926391
C	4.637169	0.422515	8.951961
C	3.584409	1.325724	8.895618
C	2.717529	1.310408	7.799004
O	4.111793	-1.396477	5.812424
O	1.689021	2.195270	7.694685

N	1.210616	-0.703555	2.845976
C	1.358512	-1.902423	3.649263
C	-0.984913	2.468598	3.591620
H	0.627826	-0.812844	2.021265
H	5.646696	-1.211833	7.971558
H	1.660836	2.739544	8.488063
H	4.555544	1.664907	5.883982
H	6.246042	3.295543	5.381969
H	6.187695	4.587731	3.246004
H	4.351289	4.138726	1.634682
H	2.409059	3.015615	0.946611
H	0.736858	1.237441	1.220134
H	-2.370077	1.772778	5.605286
H	-2.814188	0.680621	7.739958
H	5.309238	0.430416	9.802192
H	3.430538	2.044301	9.694454
H	4.901294	-1.923942	5.971668
H	1.941861	-2.065200	6.993451
H	1.321843	-3.357208	8.966579
H	-0.783279	-2.855442	10.201521
H	-2.284807	-1.071197	9.366345
H	-2.063216	2.341213	3.433443
H	-0.835080	3.259379	4.335395
H	-0.557844	2.810213	2.647506
H	1.408831	-2.750926	2.963283
H	2.279731	-1.865387	4.228607
H	0.522288	-2.058732	4.340821
O	-2.286748	-0.320297	2.644510
C	-2.079175	-0.826556	1.431631
O	-0.998105	-0.845764	0.873244
C	-3.343880	-1.380317	0.824884
H	-3.131296	-1.792326	-0.158955
H	-3.755380	-2.154210	1.476801
H	-4.093279	-0.589314	0.747711
H	-1.455582	0.076721	3.033413

Structure 6

Geometry optimization in gas phase, B3LYP/6-311G**

```
SCF Done:  E(RB3LYP) =  -1608.95793931
Zero-point correction=                           0.521822 (Hartree/Particle)
Thermal correction to Energy=                    0.554650
Thermal correction to Enthalpy=                  0.555594
Thermal correction to Gibbs Free Energy=         0.457628
Sum of electronic and zero-point Energies=          -1608.436117
Sum of electronic and thermal Energies=             -1608.403289
Sum of electronic and thermal Enthalpies=           -1608.402345
Sum of electronic and thermal Free Energies=        -1608.500311
```

Energy in DMA:

```
SCF Done:  E(RM06L) =  -1608.95202910
```

Energy in NMP:

```
SCF Done:  E(RM06L) =  -1608.95186515
```

Cartesian coordinates:

C	-2.736277	-2.273305	3.555389
C	-2.432064	-1.462459	2.431527
C	-1.075549	-1.349713	1.981010
C	-0.109222	-2.163362	2.630382
C	-0.439307	-2.961435	3.701343
C	-1.761315	-2.999978	4.191794
C	-0.775798	-0.491889	0.861057
C	-1.845984	-0.012601	0.109554
C	-3.178799	-0.133882	0.554302
C	-3.461955	-0.804677	1.714170
C	0.568389	0.049003	0.541655
C	0.829437	0.534543	-0.713601
C	-0.280067	0.205633	-1.726327
N	-1.597320	0.627903	-1.143406
C	-0.120233	0.828349	-3.083808
C	1.016070	1.405025	-3.474851

C	2.093902	1.699282	-2.542042
C	1.991837	1.337093	-1.167294
C	3.202669	2.415837	-3.010185
C	4.214325	2.828574	-2.156230
C	4.099103	2.538962	-0.799742
C	3.011834	1.816445	-0.322231
C	1.505800	0.194003	1.699647
C	2.676519	-0.570676	1.813076
C	3.536196	-0.436920	2.903378
C	3.229484	0.475307	3.907723
C	2.076786	1.245444	3.830659
C	1.222603	1.099531	2.733551
O	2.926335	-1.467190	0.812498
O	0.092601	1.851710	2.603275
N	-0.373335	-1.252711	-1.921105
C	0.859081	-1.942559	-2.294935
C	-1.796687	2.098019	-1.079900
H	-1.101844	-1.436485	-2.609149
H	4.432595	-1.045806	2.964474
H	-0.021803	2.388047	3.394406
H	2.962917	1.635855	0.736381
H	4.856026	2.877403	-0.100802
H	5.065221	3.382185	-2.536740
H	3.249275	2.661316	-4.066272
H	1.147530	1.719647	-4.505863
H	-0.926599	0.633151	-3.782789
H	-3.976694	0.286111	-0.046367
H	-4.485631	-0.892083	2.062200
H	3.893746	0.583917	4.757133
H	1.836210	1.959836	4.611492
H	3.766281	-1.905614	0.983522
H	0.897476	-2.197923	2.245814
H	0.323436	-3.579328	4.162410
H	-2.007782	-3.623855	5.043668
H	-3.768040	-2.326474	3.887974
H	-1.622055	2.531915	-2.063962
H	-2.828081	2.297876	-0.789237
H	-1.127762	2.569298	-0.352838
H	0.610928	-2.990404	-2.476766
H	1.345014	-1.544829	-3.199844
H	1.576366	-1.904775	-1.473917
O	-3.867758	0.099974	-2.701761
C	-3.778311	-0.791353	-3.692446
O	-2.753855	-1.372348	-3.984980
C	-5.094812	-0.987719	-4.402122
H	-5.831515	-1.386884	-3.700733
H	-5.472384	-0.027161	-4.759259
H	-4.965040	-1.675500	-5.234685
H	-2.990092	0.182931	-2.226484

Structure 7 (TS)

Geometry optimization in gas phase, B3LYP/6-311G**

SCF Done: E(RB3LYP) = -1608.95256432

Zero-point correction=	0.521199 (Hartree/Particle)
Thermal correction to Energy=	0.553833
Thermal correction to Enthalpy=	0.554778
Thermal correction to Gibbs Free Energy=	0.456284
Sum of electronic and zero-point Energies=	-1608.431365
Sum of electronic and thermal Energies=	-1608.398731
Sum of electronic and thermal Enthalpies=	-1608.397787
Sum of electronic and thermal Free Energies=	-1608.496281

Energy in DMA:

SCF Done: E(RM06L) = -1608.93856501

Energy in NMP:

SCF Done: E(RM06L) = -1608.93838798

Cartesian coordinates:

C	-2.876805	-2.058276	3.569200
C	-2.525110	-1.236165	2.469650

C	-1.181765	-1.244460	1.974064
C	-0.282884	-2.162180	2.557631
C	-0.645734	-2.978723	3.610626
C	-1.948687	-2.906293	4.143741
C	-0.841567	-0.385788	0.866033
C	-1.878820	0.200629	0.140611
C	-3.203052	0.216416	0.658462
C	-3.501241	-0.441422	1.813685
C	0.526041	0.098677	0.560880
C	0.809419	0.627027	-0.669756
C	-0.301627	0.358840	-1.705772
N	-1.632612	0.751266	-1.135653
C	-0.121724	1.048372	-3.038848
C	1.035201	1.630841	-3.382085
C	2.112648	1.846586	-2.438123
C	1.996810	1.410034	-1.090581
C	3.251839	2.552547	-2.866495
C	4.276374	2.865404	-1.996652
C	4.169586	2.481709	-0.664058
C	3.049791	1.784123	-0.226373
C	1.474824	0.124851	1.719117
C	2.584907	-0.715395	1.786203
C	3.457347	-0.684764	2.887033
C	3.224139	0.211953	3.924338
C	2.130685	1.053864	3.882514
C	1.262828	1.010781	2.787180
O	2.776633	-1.594614	0.766641
O	0.192549	1.849129	2.679546
N	-0.395376	-1.086231	-1.963895
C	0.853963	-1.770149	-2.333550
C	-1.982059	2.184903	-1.263091
H	-1.094102	-1.227055	-2.711728
H	4.318604	-1.360716	2.920309
H	0.115954	2.369155	3.487039
H	3.004046	1.527120	0.816334
H	4.949506	2.739640	0.048500
H	5.151460	3.401852	-2.358241
H	3.303829	2.854683	-3.903837
H	1.177154	1.989669	-4.405236
H	-0.924279	0.892790	-3.745614
H	-3.979330	0.732831	0.107460
H	-4.517116	-0.435941	2.206980
H	3.898625	0.241145	4.772181
H	1.946717	1.755466	4.689993
H	3.586468	-2.093730	0.914707
H	0.703558	-2.279544	2.124237
H	0.065295	-3.686709	4.009694
H	-2.231473	-3.537331	4.978882
H	-3.885230	-2.021112	3.935439
H	-1.930561	2.492217	-2.305926
H	-3.004796	2.338903	-0.904214
H	-1.312424	2.820953	-0.671146
H	0.597242	-2.801485	-2.577491
H	1.389717	-1.325913	-3.194488
H	1.546031	-1.790698	-1.481248
O	-4.064151	-0.804921	-2.243985
C	-3.861247	-1.177382	-3.570803
O	-2.803962	-1.011955	-4.157068
C	-5.096299	-1.821628	-4.228742
H	-5.314100	-2.770750	-3.685353
H	-5.958369	-1.115525	-4.119336
H	-4.911669	-2.034960	-5.312705
H	-3.282852	-0.244583	-1.870028

Structure 8

Geometry optimization in gas phase, B3LYP/6-311G**

SCF Done: E(RB3LYP) = -1608.95973007

Zero-point correction= 0.521775 (Hartree/Particle)

Thermal correction to Energy= 0.554810
Thermal correction to Enthalpy= 0.555754
Thermal correction to Gibbs Free Energy= 0.456158
Sum of electronic and zero-point Energies= -1608.437955
Sum of electronic and thermal Energies= -1608.404920
Sum of electronic and thermal Enthalpies= -1608.403976
Sum of electronic and thermal Free Energies= -1608.503572

Energy in DMA:

SCF Done: E(RM06L) = -1608.95636486

Energy in NMP:

SCF Done: E(RM06L) = -1608.95619087

Cartesian coordinates:

C	-3.325590	-1.638188	3.025540
C	-2.825766	-0.776231	2.018757
C	-1.475342	-0.914524	1.566123
C	-0.730280	-2.004802	2.083600
C	-1.249205	-2.843222	3.045268
C	-2.551273	-2.647074	3.545974
C	-0.969547	-0.000090	0.567414
C	-1.893147	0.803462	-0.114580
C	-3.229546	0.925062	0.343018
C	-3.668825	0.188681	1.405637
C	0.456713	0.292030	0.335354
C	0.853510	0.903045	-0.831682
C	-0.262139	0.953035	-1.885630
N	-1.491793	1.490507	-1.262625
C	0.056690	1.743264	-3.122605
C	1.280066	2.194044	-3.392984
C	2.363294	2.123511	-2.424888
C	2.158235	1.535666	-1.142529
C	3.594132	2.700231	-2.762408
C	4.646691	2.743594	-1.860941
C	4.450610	2.215611	-0.588115
C	3.237019	1.633790	-0.241050
C	1.368538	0.066392	1.501674
C	2.339843	-0.945182	1.505496
C	3.176615	-1.154318	2.601742
C	3.048034	-0.341847	3.723492
C	2.093541	0.665847	3.756656
C	1.259098	0.862775	2.652113
O	2.416328	-1.731522	0.389057
O	0.325326	1.854738	2.631051
N	-0.601862	-0.431826	-2.343150
C	0.529512	-1.229797	-2.838049
C	-1.673914	2.949522	-1.261345
H	-1.305473	-0.354902	-3.078797
H	3.916226	-1.948235	2.575741
H	0.311814	2.292433	3.488273
H	3.136490	1.257320	0.760316
H	5.244521	2.256787	0.149564
H	5.593716	3.192002	-2.138546
H	3.708503	3.129187	-3.752757
H	1.490760	2.669192	-4.346469
H	-0.749457	1.824201	-3.845328
H	-3.910740	1.581484	-0.184531
H	-4.692722	0.285183	1.751453
H	3.695822	-0.498380	4.578134
H	1.992786	1.302512	4.629836
H	3.128613	-2.369517	0.501449
H	0.246751	-2.217348	1.679360
H	-0.653148	-3.673955	3.407159
H	-2.947129	-3.306947	4.309808
H	-4.348173	-1.499972	3.362438
H	-1.606612	3.342902	-2.274068
H	-2.663843	3.182416	-0.873417
H	-0.931654	3.459617	-0.634853
H	0.126494	-2.152230	-3.260082
H	1.114834	-0.722289	-3.615509

H	1.190880	-1.486343	-2.010538
O	-2.431030	-2.318675	-1.451736
C	-3.191282	-2.415417	-2.542642
O	-3.026233	-1.754913	-3.549391
C	-4.283207	-3.445915	-2.381030
H	-3.843198	-4.416213	-2.139851
H	-4.928669	-3.166714	-1.545014
H	-4.865519	-3.514419	-3.297447
H	-1.739875	-1.607558	-1.592456

Structure 9 (TS)

Geometry optimization in gas phase, B3LYP/6-311G**

```
 SCF Done:  E(RB3LYP) =  -1608.94452101
 Zero-point correction=                           0.520233 (Hartree/Particle)
 Thermal correction to Energy=                    0.553215
 Thermal correction to Enthalpy=                  0.554159
 Thermal correction to Gibbs Free Energy=         0.454694
 Sum of electronic and zero-point Energies=          -1608.424288
 Sum of electronic and thermal Energies=             -1608.391306
 Sum of electronic and thermal Enthalpies=           -1608.390362
 Sum of electronic and thermal Free Energies=        -1608.489827
```

Energy in DMA:

SCF Done: E(RM06L) = -1608.95070888

Energy in NMP:

SCF Done: E(RM06L) = -1608.95049077

Cartesian coordinates:

C	-1.497623	-1.457970	9.120776
C	-1.179097	-0.650412	8.012297
C	0.144642	-0.621837	7.477831
C	1.061295	-1.532368	8.031084
C	0.730874	-2.340494	9.107316
C	-0.539878	-2.282098	9.682402
C	0.445834	0.268632	6.349706
C	-0.654701	0.838710	5.655702
C	-1.981208	0.765918	6.202395
C	-2.211717	0.093857	7.349721
C	1.753755	0.715435	6.001547
C	1.938899	1.388796	4.768614
C	0.807465	1.601099	3.947851
N	-0.434082	1.500844	4.484140
C	0.903778	1.766474	2.530545
C	2.119977	2.079207	1.997414
C	3.261232	2.291863	2.845655
C	3.193858	1.946178	4.230513
C	4.418452	2.875622	2.320581
C	5.506935	3.177562	3.114490
C	5.435852	2.895317	4.488030
C	4.316112	2.289517	5.030809
C	2.857406	0.558554	6.994679
C	3.936000	-0.323533	6.821299
C	4.952037	-0.416020	7.772085
C	4.897923	0.383121	8.909364
C	3.850910	1.261351	9.105567
C	2.835940	1.342841	8.158558
O	3.918382	-1.097373	5.703065
O	1.778353	2.202471	8.293830
N	0.668412	-1.148402	3.298000
C	1.933233	-1.495443	2.679305
C	-1.534068	2.170919	3.735612
H	0.477705	-1.721374	4.121215
H	5.773238	-1.109317	7.622212
H	1.863592	2.674960	9.124657
H	4.293989	2.130269	6.092509
H	6.254592	3.166790	5.138709
H	6.393598	3.639825	2.696295
H	4.447628	3.103166	1.254172
H	2.240752	2.213464	0.922421
H	0.022518	1.585884	1.908752

H	-2.802217	1.209549	5.668444
H	-3.217742	0.041278	7.753867
H	5.696795	0.317044	9.650038
H	3.807675	1.884897	10.003179
H	4.724688	-1.623359	5.657435
H	2.037623	-1.643545	7.589791
H	1.467555	-3.031813	9.501021
H	-0.786264	-2.905191	10.534288
H	-2.509335	-1.434580	9.511951
H	-1.107618	3.053878	3.262671
H	-1.903415	1.490417	2.923470
H	-2.319588	2.494064	4.419077
H	2.018484	-2.541156	2.272880
H	2.138007	-0.807884	1.832188
H	2.767207	-1.368964	3.392759
O	-1.337732	-1.398078	1.453487
C	-1.863244	-0.440356	0.834513
O	-1.737797	0.797748	1.060089
C	-2.797851	-0.860084	-0.307994
H	-2.264052	-1.548543	-0.980577
H	-3.637857	-1.423196	0.109031
H	-3.180599	-0.014980	-0.878150
H	-0.103241	-1.202176	2.664343

5

Geometry optimization in gas phase, B3LYP/6-311G**

SCF Done: E(RB3LYP) = -1608.95791580

Zero-point correction=	0.520115	(Hartree/Particle)
Thermal correction to Energy=	0.554700	
Thermal correction to Enthalpy=	0.555644	
Thermal correction to Gibbs Free Energy=	0.450495	
Sum of electronic and zero-point Energies=	-1608.437801	
Sum of electronic and thermal Energies=	-1608.403216	
Sum of electronic and thermal Enthalpies=	-1608.402272	
Sum of electronic and thermal Free Energies=	-1608.507421	

Energy in DMA:

SCF Done: E(RM06L) = -1608.97364223

Energy in NMP:

SCF Done: E(RM06L) = -1608.97303612

Cartesian coordinates:

C	-2.743759	-1.756163	4.034801
C	-2.451238	-0.996005	2.882710
C	-1.126445	-0.953668	2.359827
C	-0.175615	-1.801495	2.970109
C	-0.490304	-2.565296	4.079138
C	-1.773963	-2.524174	4.641283
C	-0.851991	-0.114871	1.187950
C	-1.972040	0.333632	0.434244
C	-3.289661	0.267793	0.986270
C	-3.500833	-0.322636	2.188163
C	0.426373	0.361747	0.798154
C	0.576759	1.038158	-0.431418
C	-0.540690	1.049955	-1.325188
N	-1.793297	0.850010	-0.813819
C	-0.357635	1.268460	-2.726639
C	0.871165	1.603373	-3.189393
C	1.965053	1.886628	-2.312526
C	1.816549	1.664188	-0.914966
C	3.155951	2.440396	-2.823513
C	4.171592	2.840917	-1.980931
C	3.996143	2.714611	-0.597108
C	2.849301	2.139200	-0.078148
C	1.602781	0.156761	1.695266
C	2.650334	-0.705332	1.334704
C	3.750601	-0.890419	2.174171
C	3.813392	-0.204809	3.381911
C	2.792141	0.658350	3.762636
C	1.692014	0.832277	2.921018

O	2.520930	-1.363171	0.152773
O	0.674490	1.685590	3.227145
N	-1.027956	-2.096819	-1.676002
C	0.143348	-2.711861	-2.303298
C	-2.998487	1.165472	-1.623127
H	-1.535209	-2.806774	-1.154812
H	4.546283	-1.567068	1.880777
H	0.831635	2.071294	4.095142
H	2.744066	2.095124	0.992454
H	4.758051	3.080638	0.081739
H	5.079013	3.277607	-2.381448
H	3.245530	2.572210	-3.896365
H	1.018606	1.713566	-4.258604
H	-1.172502	1.098031	-3.436814
H	-4.117710	0.701060	0.449976
H	-4.499588	-0.342466	2.610440
H	4.668137	-0.344414	4.033280
H	2.844940	1.196708	4.703176
H	3.313161	-1.882096	-0.021318
H	0.811699	-1.893990	2.551814
H	0.266500	-3.213367	4.506205
H	-2.008283	-3.115944	5.518513
H	-3.759021	-1.748413	4.416448
H	-3.489733	2.036975	-1.185225
H	-2.733309	1.349340	-2.656788
H	-3.649499	0.295965	-1.640345
H	-0.080094	-3.543586	-2.990363
H	0.683979	-1.952240	-2.875078
H	0.829035	-3.081855	-1.534190
O	-3.016255	-1.148094	-3.624480
C	-3.057338	-0.377813	-4.619361
O	-2.604196	0.798592	-4.684570
C	-3.718193	-0.938721	-5.890871
H	-3.042731	-1.670088	-6.349591
H	-4.638881	-1.468673	-5.631833
H	-3.922904	-0.146684	-6.612916
H	-1.677215	-1.769171	-2.401349

Miscellaneous structures

Methyl amine

Geometry optimization in gas phase, B3LYP/6-311G**

```
 SCF Done:  E(RB3LYP) =  -95.8884404259
 Zero-point correction=                           0.063817 (Hartree/Particle)
 Thermal correction to Energy=                    0.067227
 Thermal correction to Enthalpy=                  0.068171
 Thermal correction to Gibbs Free Energy=         0.040906
 Sum of electronic and zero-point Energies=            -95.824624
 Sum of electronic and thermal Energies=               -95.821214
 Sum of electronic and thermal Enthalpies=             -95.820269
 Sum of electronic and thermal Free Energies=          -95.847535
```

Energy in DMA:

```
 SCF Done:  E(RM06L) =  -95.8810619091
```

Energy in NMP:

```
 SCF Done:  E(RM06L) =  -95.8810290473
```

Cartesian coordinates:

```
 N     0.145531     0.066729     0.081226
 H     0.270509     0.019161     1.087456
 H     1.076966     0.118698    -0.318878
 C    -0.541444    -1.136708    -0.397844
 H    -1.541925    -1.178997     0.040649
 H    -0.037334    -2.091075    -0.177433
 H    -0.670864    -1.070496    -1.481335
```

Methyl ammonium

Geometry optimization in gas phase, B3LYP/6-311G**

```
 SCF Done:  E(RB3LYP) =  -96.2487146337
 Zero-point correction=                           0.079156 (Hartree/Particle)
 Thermal correction to Energy=                    0.082612
 Thermal correction to Enthalpy=                  0.083556
 Thermal correction to Gibbs Free Energy=         0.055971
 Sum of electronic and zero-point Energies=            -96.169558
 Sum of electronic and thermal Energies=               -96.166103
 Sum of electronic and thermal Enthalpies=             -96.165159
 Sum of electronic and thermal Free Energies=          -96.192744
```

Energy in DMA:

```
 SCF Done:  E(RM06L) =  -96.3363740682
```

Energy in NMP:

```
 SCF Done:  E(RM06L) =  -96.3358750962
```

Cartesian coordinates:

```
 C    -0.585415    -1.171625    -0.425450
 N     0.149798     0.052391     0.082873
 H     0.265634     0.025540     1.100947
 H     1.085205     0.127285    -0.329443
 H    -1.566118    -1.198849     0.043990
 H    -0.006297    -2.052176    -0.156704
 H    -0.678740    -1.088313    -1.505854
 H    -0.354568     0.915250    -0.144711
```

Acetic acid

Geometry optimization in gas phase, B3LYP/6-311G**

```
 SCF Done:  E(RB3LYP) =  -229.156430482
 Zero-point correction=                           0.061673 (Hartree/Particle)
 Thermal correction to Energy=                    0.066234
 Thermal correction to Enthalpy=                  0.067179
 Thermal correction to Gibbs Free Energy=         0.034504
 Sum of electronic and zero-point Energies=           -229.094757
 Sum of electronic and thermal Energies=              -229.090196
 Sum of electronic and thermal Enthalpies=            -229.089252
 Sum of electronic and thermal Free Energies=         -229.121927
```

Energy in DMA:

```
 SCF Done:  E(RM06L) =  -229.157074391
```

Energy in NMP:

```
 SCF Done:  E(RM06L) =  -229.157027524
```

Cartesian coordinates:

C	-1.275324	0.000017	-0.703185
C	-0.014810	0.000156	0.120307
O	1.089871	-0.000030	-0.668707
O	0.049064	0.000003	1.321720
H	-2.140000	-0.000069	-0.043721
H	-1.295787	0.880204	-1.349633
H	-1.295616	-0.880174	-1.349634
H	1.852101	-0.000109	-0.070764

Acetate

Geometry optimization in gas phase, B3LYP/6-311G**

```
SCF Done:  E(RB3LYP) =  -228.576047417
Zero-point correction=                           0.047639 (Hartree/Particle)
Thermal correction to Energy=                    0.052125
Thermal correction to Enthalpy=                  0.053069
Thermal correction to Gibbs Free Energy=         0.019716
Sum of electronic and zero-point Energies=           -228.528409
Sum of electronic and thermal Energies=              -228.523922
Sum of electronic and thermal Enthalpies=            -228.522978
Sum of electronic and thermal Free Energies=         -228.556332
```

Energy in DMA:

SCF Done: E(RM06L) = -228.679595314

Energy in NMP:

SCF Done: E(RM06L) = -228.679077367

Cartesian coordinates:

C	0.081190	-0.000001	0.091106
O	-0.016749	0.000000	1.338473
O	1.101633	0.000000	-0.635200
C	-1.281012	0.000000	-0.703230
H	-2.145632	0.000000	-0.031885
H	-1.324464	0.880023	-1.356881
H	-1.324464	-0.880023	-1.356881

(*aR*)-**A** without extra amine

Geometry optimization in gas phase, B3LYP/6-311G**

```
SCF Done:  E(RB3LYP) =  -1283.91389206
Zero-point correction=                           0.390936 (Hartree/Particle)
Thermal correction to Energy=                    0.414639
Thermal correction to Enthalpy=                  0.415583
Thermal correction to Gibbs Free Energy=         0.337009
Sum of electronic and zero-point Energies=          -1283.522956
Sum of electronic and thermal Energies=             -1283.499253
Sum of electronic and thermal Enthalpies=           -1283.498309
Sum of electronic and thermal Free Energies=        -1283.576883
```

Energy in DMA:

SCF Done: E(RM06L) = -1283.91950406

Energy in NMP:

SCF Done: E(RM06L) = -1283.91937704

Cartesian coordinates:

C	8.138140	18.087186	0.133403
C	8.250425	16.773634	-0.209524
C	7.739581	15.683999	0.587158
C	7.146362	16.054640	1.929709
C	6.981285	17.473218	2.187471
C	7.477105	18.422384	1.348926
C	7.750512	14.390775	0.075557
C	8.433012	14.122975	-1.195195
C	8.926995	15.236890	-1.887724
O	8.822188	16.498691	-1.420095
C	9.551697	15.172301	-3.150462
C	9.710029	13.962600	-3.753021
C	9.289875	12.767807	-3.110749
C	8.666906	12.818787	-1.817532
C	8.358777	11.567491	-1.225936
C	8.605682	10.373722	-1.870176
C	9.178919	10.341072	-3.152140

C	9.518925	11.527414	-3.753283
C	6.980965	13.304974	0.763265
C	7.505339	12.635297	1.872528
C	6.763405	11.566006	2.444003
C	5.544983	11.198996	1.938363
C	4.969053	11.877459	0.838626
C	5.701939	12.953570	0.240489
C	3.694593	11.526042	0.327759
C	3.145314	12.199631	-0.735012
C	3.860015	13.264933	-1.327063
C	5.096779	13.633025	-0.854837
N	8.761592	12.953302	2.358643
C	9.179068	12.555403	3.695519
O	6.864907	15.208261	2.778920
H	7.926054	11.530905	-0.243729
H	8.350613	9.446708	-1.369349
H	9.359162	9.395814	-3.650603
H	9.982040	11.536629	-4.734235
H	10.174948	13.894521	-4.730381
H	9.875179	16.099961	-3.604327
H	8.516303	18.847677	-0.536229
H	7.373439	19.472210	1.603537
H	6.495527	17.727654	3.120993
H	5.614445	14.462326	-1.319984
H	3.424196	13.803752	-2.161306
H	2.168872	11.924058	-1.116321
H	3.155855	10.710889	0.800477
H	4.996568	10.381231	2.394565
H	7.165919	11.039335	3.298732
H	9.321221	11.473237	3.754690
H	8.469216	12.862920	4.474123
H	10.143529	13.020403	3.902891
H	9.033944	13.898593	2.134165

Reference

1. F. Torricelli, J. Bosson, C. Besnard, M. Chekini, T. Bürgi and J. Lacour, *Angew. Chem., Int. Ed. Engl.*, 2013, **52**, 1796-1800.
2. G. M. Labrador, J. Bosson, Z. S. Breitbach, Y. Lim, E. R. Francotte, R. Sabia, C. Villani, D. W. Armstrong and J. Lacour, *Chirality*, 2016, **28**, 282-289.
3. A. D. Becke, *J. Chem. Phys.*, 1993, **98**, 5648-5652.
4. C. Lee, W. Yang and R. G. Parr, *Phys. Rev. B: Condens. Matter*, 1988, **37**, 785-789.
5. X. Li and M. J. Frisch, *J. Chem. Theory Comput.*, 2006, **2**, 835-839.
6. S. H. Vosko, L. Wilk and M. Nusair, *Can. J. Phys.*, 1980, **58**, 1200-1211.
7. A. D. McLean and G. S. Chandler, *J. Chem. Phys.*, 1980, **72**, 5639-5648.
8. H. P. Hratchian and H. B. Schlegel, *J. Chem. Theory Comput.*, 2005, **1**, 61-69.
9. H. P. Hratchian and H. B. Schlegel, *J. Chem. Phys.*, 2004, **120**, 9918-9924.
10. K. Fukui, *Acc. Chem. Res.*, 1981, **14**, 363-368.
11. Y. Zhao and D. G. Truhlar, *J. Chem. Phys.*, 2006, **125**, 194101.
12. F. Weigend, *Phys. Chem. Chem. Phys.*, 2006, **8**, 1057-1065.
13. F. Weigend and R. Ahlrichs, *Phys. Chem. Chem. Phys.*, 2005, **7**, 3297-3305.
14. G. Scalmani and M. J. Frisch, *J. Chem. Phys.*, 2010, **132**, 114110.
15. M. J. Frisch, G. W. Trucks, H. B. Schlegel, G. E. Scuseria, M. A. Robb, J. R. Cheeseman, G. Scalmani, V. Barone, B. Mennucci, G. A. Petersson, H. Nakatsuji, M. Caricato, X. Li, H. P. Hratchian, A. F. Izmaylov, J. Bloino, G. Zheng, J. L. Sonnenberg, M. Hada, M. Ehara, K. Toyota, R. Fukuda, J. Hasegawa, M. Ishida, T. Nakajima, Y. Honda, O. Kitao, H. Nakai, T. Vreven, J. A. Montgomery Jr., J. E. Peralta, F. Ogliaro, M. J. Bearpark, J. Heyd, E. N. Brothers, K. N. Kudin, V. N. Staroverov, R. Kobayashi, J. Normand, K. Raghavachari, A. P. Rendell, J. C. Burant, S. S. Iyengar, J. Tomasi, M. Cossi, N. Rega, N. J. Millam, M. Klene, J. E. Knox, J. B. Cross, V. Bakken, C. Adamo, J. Jaramillo, R. Gomperts, R. E. Stratmann, O. Yazyev, A. J. Austin, R. Cammi, C. Pomelli, J. W. Ochterski, R. L. Martin, K. Morokuma, V. G. Zakrzewski, G. A. Voth, P. Salvador, J. J. Dannenberg, S. Dapprich, A. D. Daniels, Ö. Farkas, J. B. Foresman, J. V. Ortiz, J. Cioslowski and D. J. Fox, *Gaussian 09*, Gaussian, Inc., Wallingford, CT, USA, 2009.
